# Ruthenium-Mediated *N*‑Arylation for DNA-Encoded Libraries

**DOI:** 10.1021/jacs.5c11842

**Published:** 2025-09-11

**Authors:** Suraj Kanoo, Eduardo de Pedro Beato, Tim Schulte, Lara Vogelsang, Luca Torkowski, Felix Waldbach, Philipp Hartmann, Riya Kayal, Karl-Josef Dietz, Tobias Ritter

**Affiliations:** † 28314 Max-Planck-Institut für Kohlenforschung, D-45470 Mülheim an der Ruhr, Germany; ‡ Institute of Organic Chemistry, RWTH Aachen University, 52074 Aachen, Germany; § Biochemistry and Physiology of Plants, Faculty of Biology, Bielefeld University, Universitätsstraße 25, 33615 Bielefeld, Germany

## Abstract

C–N cross coupling reactions are widely employed for the construction of carbon–nitrogen bonds. However, control of chemoselectivity in the presence of the amino functionality in oligonucleotides remains a challenge. Here, we report the development of a new ruthenium reagent that enables the chemoselective *N*-arylation of amine–DNA conjugates with distinct chemoselectivity when compared to conventional palladium-based C–N bond-forming catalysts. The ruthenium reagent activates commercially available haloarenes *in situ* via η^6^ π-arene coordination for subsequent S_N_Ar with the amine. The method is compatible with various commercially available haloarenes and aliphatic amines, and the reaction proceeds under mild conditions.

## Graphical abstract

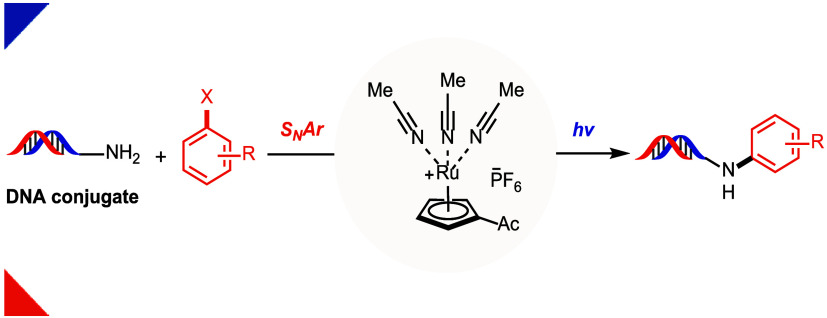

## Introduction

DNA-encoded library (DEL) technology combines combinatorial chemistry and molecular biology to build and evaluate large chemical libraries. Synthesis of DELs relies on readily available, diverse building blocks, and robust reactions to connect them. Cross-coupling reactions, like the Buchwald-Hartwig amination in which aryl (pseudo)­halides linked to DNA reacts with an external amine have been applied to DEL synthesis. However, the reverse approach—DNA-linked amines with external aryl halides—remains unreported. The main challenge for such chemistry lies in the propensity of aryl halides to engage in arylation reactions with the amine groups present in the nucleobases of DNA. We developed an air-stable acetyl-substituted cyclopentadienyl (CpAc) ruthenium complex (**1**) for coupling of diverse aryl halides to DNA-conjugated amines. The ruthenium complex activates aryl halides via π-arene coordination for nucleophilic aromatic substitution (S_N_Ar). The *N*-arylation is operationally simple, no organometallic needs to be isolated, unlocking a large previously inaccessible chemical space for DELs.

The first report that proposed the concept of DNA-encoded libraries (DELs) involved the formation of an amide between an amine-DNA conjugate and an amino acid building block.^1^ Since then, various reactions that exploit the intrinsic nucleophilicity of amines have advanced the field of DEL, including amidation, reductive amination, heterocycle synthesis, and S_N_Ar reactions, among others.^2–4^ Amines are among the most commonly used functional groups in DEL synthesis.^5^ Analogous to small-molecule medicinal chemistry,^6^ C–N cross-coupling reactions, such as Ullmann couplings^7^ and Buchwald–Hartwig aminations,^8–11^ are valuable for DEL synthesis due to robust and orthogonal disconnection, along with the availability of the commercially available aniline and aryl halide building blocks (Figure 1). Yet, no general *N*-arylation reaction has been reported to date, in which the amine functional group is covalently attached to DNA, with the aryl halide as the external coupling partner. Because amines constitute the most commonly found reactive terminus on DNA for DEL, the inability to perform C–N cross-coupling with DNA-conjugated amines presents a severe limitation to structural diversity.^12^ An alternative approach to C–N bond formation for aniline synthesis exploits nucleophilic aromatic substitution (S_N_Ar), which displays chemoselectivity in favor of the nucleophilic amine functional group,^13^ while the less nucleophilic amino groups on the DNA nucleobases remain unreactive. However, a key limitation of conventional S_N_Ar lies in the limited scope of appropriate aryl electrophiles, largely limited to arenes substituted with electron-withdrawing groups (e.g., −NO_2_) and a narrow range of electron-poor heterocycles, such as triazines^14^ and pyrimidines.^15^ This constraint often leads to common core structures in the final library members and reduces the chemical diversity accessible through S_N_Ar.

**Figure 1:**
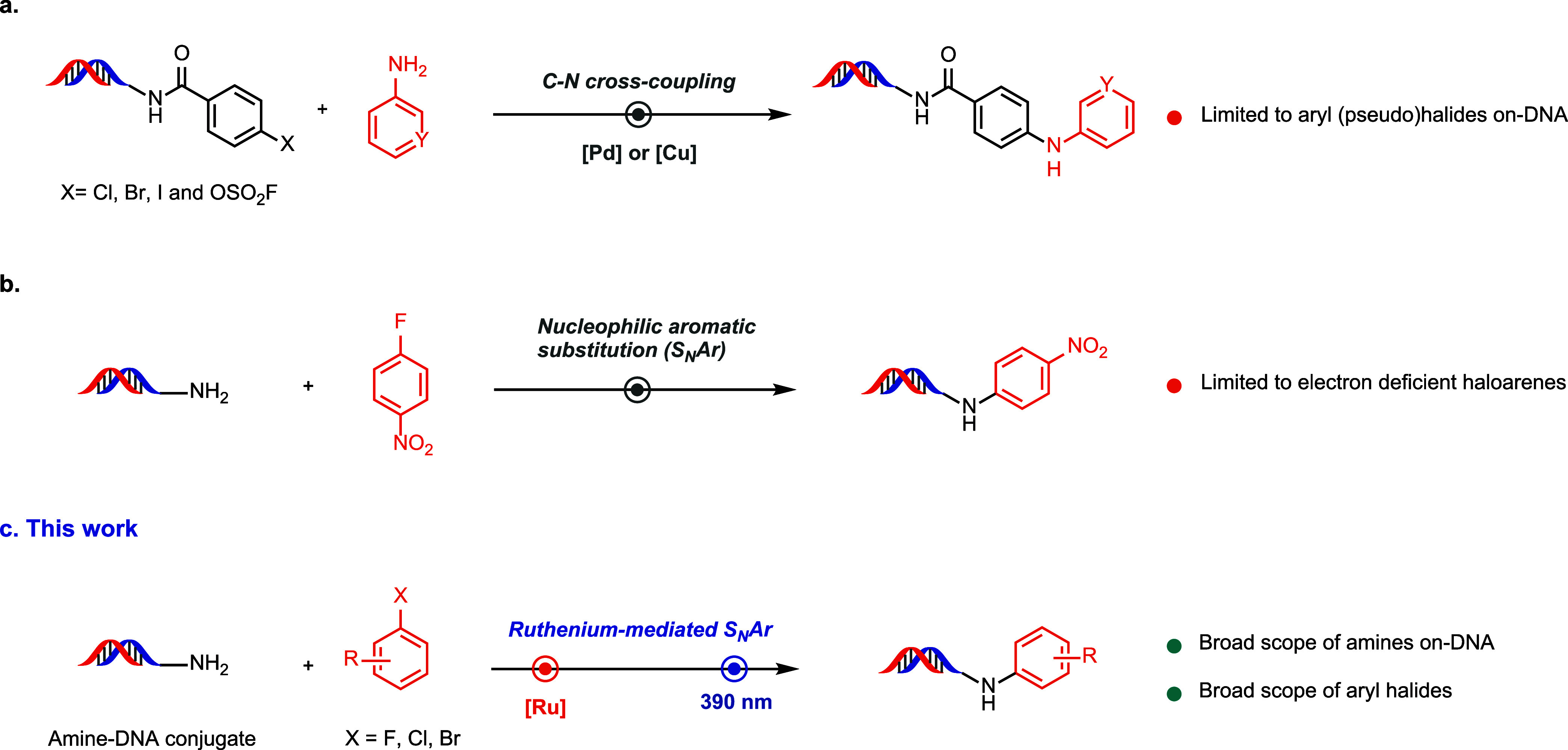
*N*-arylation on-DNA. (a) Palladium and copper-mediated on-DNA C–N cross coupling of DNA-linked aryl (pseudo)­halides with amines as the external reagents. (b) On-DNA nucleophilic aromatic substitution (S_N_Ar) of electron-deficient fluoroarenes. (c) On-DNA ruthenium-mediated S_N_Ar of aliphatic amine-DNA conjugates with various haloarenes activated via π-arene coordination.

The challenge in applying palladium-catalyzed C–N cross-coupling to DNA-conjugated amines, and the potential reason why the reaction remains unreported, is the propensity of aryl halides to undergo undesired arylation with the amino functional groups of the nucleobases of the DNA backbone. The heteroaromatic amino groups in DNA nucleobases generally exhibit lower p*K*_a_ values than aliphatic amines.^16,17^ As a result, reductive elimination from palladium–DNA amido complexes may preferentially lead to arylation of nucleobases rather than the intended aliphatic amine.^18,19^ The Buchwald–Hartwig amination relies on oxidative addition and reductive elimination as the key steps; we envisioned that an S_N_Ar-type reaction mediated by π-arene coordination to a metal center could overcome the current limitations in chemoselectivity. Upon coordination to the metal center, the haloarene forms an η^6^ π-arene complex, which increases the electrophilicity of the arene ring.^20^ A key element of our design was the development of a transition metal reagent (**1**, Figure 2) that could form the π-arene complex *in situ*, so that no organometallic would need to be prepared and isolated. Instead, a single compound that could eventually become commercially available (**1**), can be added to available aryl halides and the DEL to increase synthetic utility and practicality for C–N bond formation (**3**), with subsequent photolysis to afford the desired anilines (**4**).

**Figure 2:**
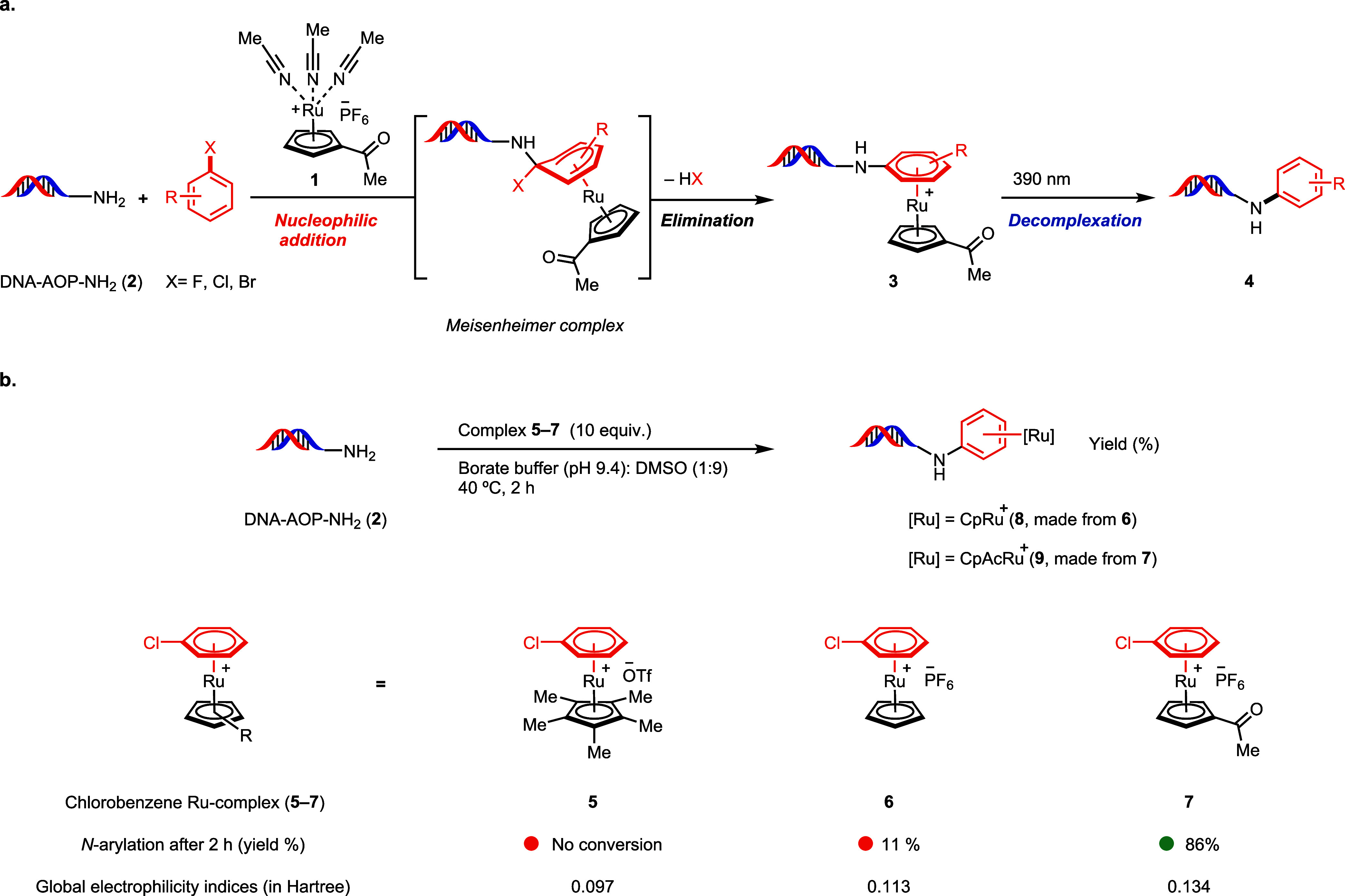
*N*-arylation of DNA-conjugated amines in aqueous media. (a) S_N_Ar of DNA-conjugated aliphatic amines with η^6^ arene complexes. (b) Electrophilicity of Ru π-arene complexes **5**–**7** and *N*-arylation of DNA-conjugate **2** (see Supporting Information page, S234).

## Results and Discussion

Ruthenium is among the most commonly used transition metals in π-arene coordination.^21–23^ We began our investigation with well-known cyclopentadienyl (Cp)-derived ruthenium complexes bearing either a Cp or Cp* ligand (**5** and **6**, respectively) to perform π-arene chemistry due to their availability. While arene coordination to both [RuCp­(MeCN)_3_]­PF_6_ and [RuCp*­(MeCN)_3_]­PF_6_ is well established,^24,25^ the respective η^6^ π-chlorobenzene complexes were unsuitable for amine arylation on DNA. For example, the reaction of DNA-AOP-NH_2_ (**2**) with the Cp* derivative **5** showed no conversion, while the reaction with Cp complex **6** resulted in the desired DNA-conjugate in only 11% yield (Figure 2b), consistent with insufficient electrophilicity of the electron-rich Cp* ligand-based complex, which could only marginally be improved with the slightly less electron-rich Cp-based compound. We rationalized that modifying the Cp ligand with an electron-withdrawing substituent may result in sufficient electrophilicity, and evaluated various ruthenium complexes, which ultimately led to the development of the novel, air-stable, and DNA-compatible acetyl-substituted cyclopentadienyl (CpAc) ruthenium complex **1**. A computational study of the global electrophilicity indices of the three ruthenium complexes **5**–**7** supports the anticipated electrophilicity order (Figure 2b). When complex **1** is mixed with various haloarenes in dimethyl carbonate (DMC) solvent at 80 °C for 2 h, η^6^ ruthenium π-arene complexes like **7** are formed *in situ*. These complexes could be used directly, without isolation or purification, for subsequent reactions with amine-DNA conjugates to achieve *N*-arylation. For example, complex **7** provides *N*-arylated product in 86% yield (Figure 2b) within 2 h. Analogous to amide coupling reagents such as DMT-MM, and HATU, which activate carboxylic acids for acylation reactions,^5,12^ our protocol involves activation of haloarenes by **1**. The protocol is operationally simple, and utilizes a single bench-stable ruthenium reagent. Ruthenium complex **1** can be synthesized on a gram scale as a crystalline yellow solid (see Supporting Information pages, S15–S17).

Ruthenium η^6^ π-arene complexes are known to undergo decomplexation upon irradiation with light.^26–29^ Prior studies have shown that DNA conjugates can be stable toward light irradiation at wavelengths above 360 nm.^30^ On-DNA η^6^-arene ruthenium complex **3** undergoes clean decomplexation to form arylamine **4** (Figure 2a) in water within 2 h of 390 nm irradiation.

To evaluate the regio- and chemoselectivity of ruthenium π-arene complexes in S_N_Ar reactions with aliphatic amines, we synthesized the oligonucleotide mimic **10** (Figure 3a), bearing a single adenine residue and both primary and secondary amine groups. Reaction of amine **10** with ruthenium complex **11** afforded chemoselective arylation at the primary aliphatic amine and resulted in aniline **12** in 83% yield, with no detected modification of the adenine nucleobase. Instead, the Buchwald-type oxidative addition (OAC) complex^31,32^ **13** chemoselectively arylated at the nucleobase and resulted in the formation of product **14** in 87% yield (see Supporting Information pages, S25–S27).

**Figure 3:**
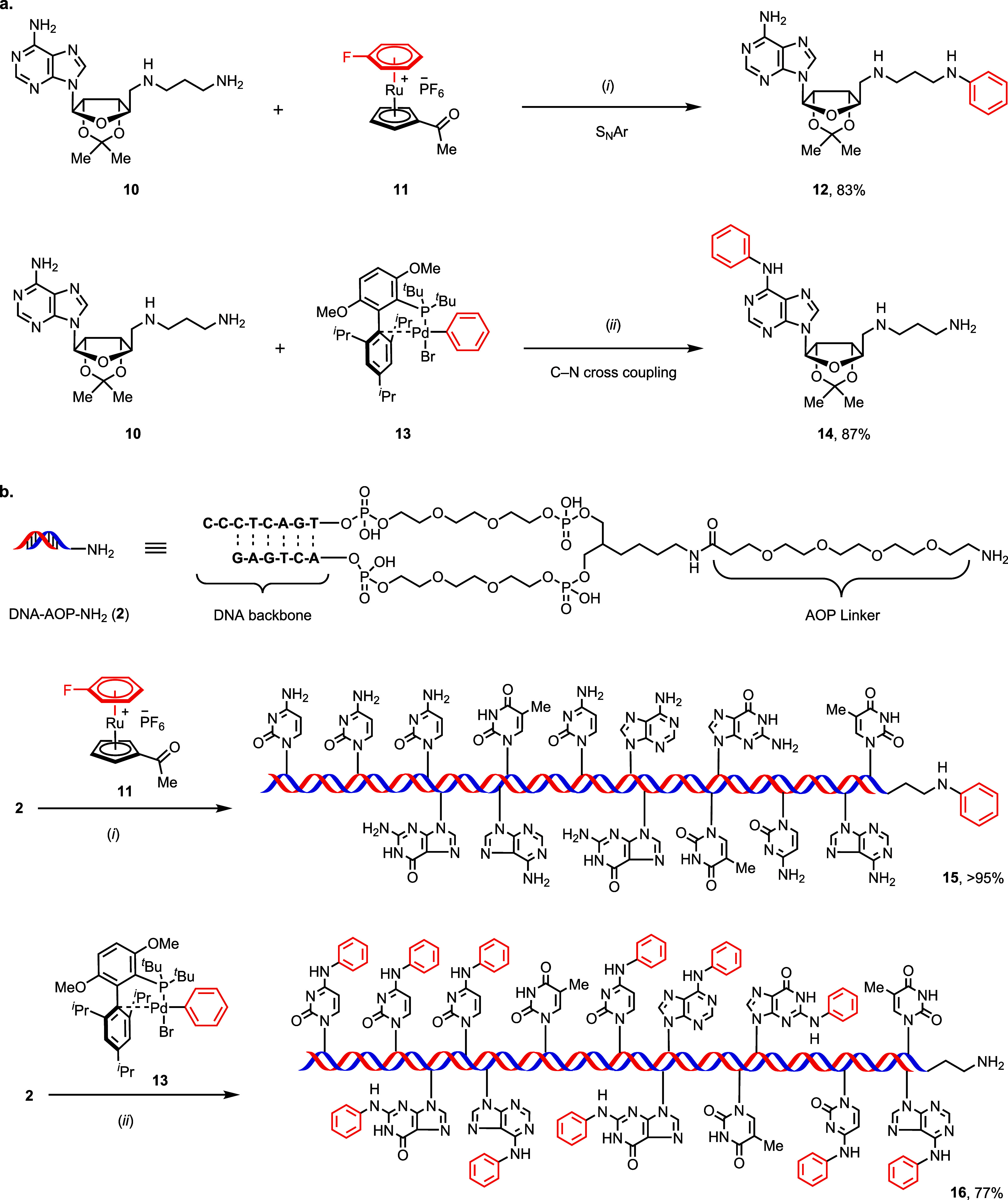
Chemoselectivity of DNA *N*-arylation. (a) Reaction of oligonucleotide mimic **10** for C–N bond formation: (i) 1.0 equiv of **10** (*c* = 0.10 M) and 1.0 equiv of **11** in sodium borate buffer (*c* = 0.50 M, pH 9.4): DMSO (1:9), 40 °C, 16 h; then 390 nm irradiation. (ii) 1.0 equiv of compound **10** (*c* = 0.10 M) and 1.5 equiv of complex **13** in sodium borate buffer (*c* = 0.50 M, pH 9.4): DMSO (1:9), 40 °C, 16 h. (b) *N*-arylation of DNA fragment **2**. (i) Fluorobenzene (*c* = 10 mM) and Ru complex **1** (*c* = 1.0 mM) in DMC, 80 °C, 2 h to form **11** *in situ*; then DNA conjugate (**2**, c = 0.10 mM) in sodium borate buffer (*c* = 0.50 M, pH 9.4): DMSO (1:9), 40 °C, 2–16 h; then 390 nm irradiation in water (*c* = 0.10 mM). (ii) DNA-AOP-NH_2_ (**2**, *c* = 0.10 mM) in sodium borate buffer (*c* = 0.50 M, pH 9.4): DMSO (1:9), 50 equiv of complex **13** in DMSO (*c* = 5.0 mM), 40 °C, 2–16 h and (see Supporting Information pages, S25–S27 and S235). DMSO, dimethyl sulfoxide.

The DNA fragment **2** employed in this study contains all four canonical nucleobases—adenine (A), guanine (G), thymine (T), and cytosine (C), all of which, except thymine contain an NH_2_ group (Figure 3b). The reaction of DNA-conjugate **2** with an excess of the Buchwald OAC **13** (Figure 3b) resulted in clean and efficient 11-fold arylation, as confirmed by LC–MS analysis. DNA-conjugate **2** contains 11 heteroaromatic primary amino groups, located on three adenine, three guanine, and five cytosine nucleobases in the oligonucleotide backbone, respectively. Exhaustive and chemoselective arylation of all A, G, and C nucleobases in the presence of T and the primary aliphatic amine at the terminus is consistent with our LC–MS results to form product **16**. Treatment of **2** with 10-fold excess of the ruthenium complex derived from **1** displayed equally selective but complementary reactivity to afford a single monoarylated product **15**, established by the LC–MS analysis, and consistent with exclusive arylation at the single primary amine, as in **12**. The complementary and orthogonal reactivity of ruthenium-based complex **11** and palladium-based compound **13** may be rationalized by the distinct mechanism pathways by which the C–N bond is formed. *N*-arylation with arylpalladium complexes proceeds through reductive elimination from a Pd­(II) amido complex, in which the amine has been deprotonated.^18^ Because aliphatic amines are less acidic than the amino groups on nucleobases,^16,17^ chemoselective deprotonation occurs on the nucleobases. Amine attack on π-arene-coordinated ruthenium complexes occurs from the neutral amine, hence addition of the more nucleophilic, basic aliphatic amine occurs chemoselectively in preference to the less nucleophilic aminonucleobases.

Evaluation of different aryl halides for *N*-arylation of **2** indicated that all four halides are suitable (Figure 4). Even iodobenzene afforded a 70% yield of the aniline **15**, albeit with a larger excess of the complex and a longer reaction time. The method is compatible with a variety of differently substituted aryl fluorides, aryl chlorides, and aryl bromides. Various haloarenes with *ortho* (**30**–**35**), *meta* (**24**–**29**), and *para* (**17**–**23**) substitutions, with other halides (**17**–**19**, **24**–**26**, **30**–**33**), carbonyls (**21**, **36**–**40**), and sulfonamides (**41**, **42**), resulted in yields over 80%. Additionally, primary amides (**35**, **44**) resulted in product formation above 50% yield. Electron-donating groups on the arene ring, such as alkyl (**20**, **27**, **34**, **43**) and alkoxy (**22**, **29**) substituents are well tolerated, despite their electron-donating nature, which decreases electrophilicity of the coordinated arenes. Thioether **23** also resulted in the formation of the desired product with yields above 50%, with thiol oxidation as the side product. With more than one halide on the arene, substitution occurs in the order *F* > Cl ∼ Br > I (see Supporting Information pages, S254–S255), as expected for an S_N_Ar pathway.

**Figure 4:**
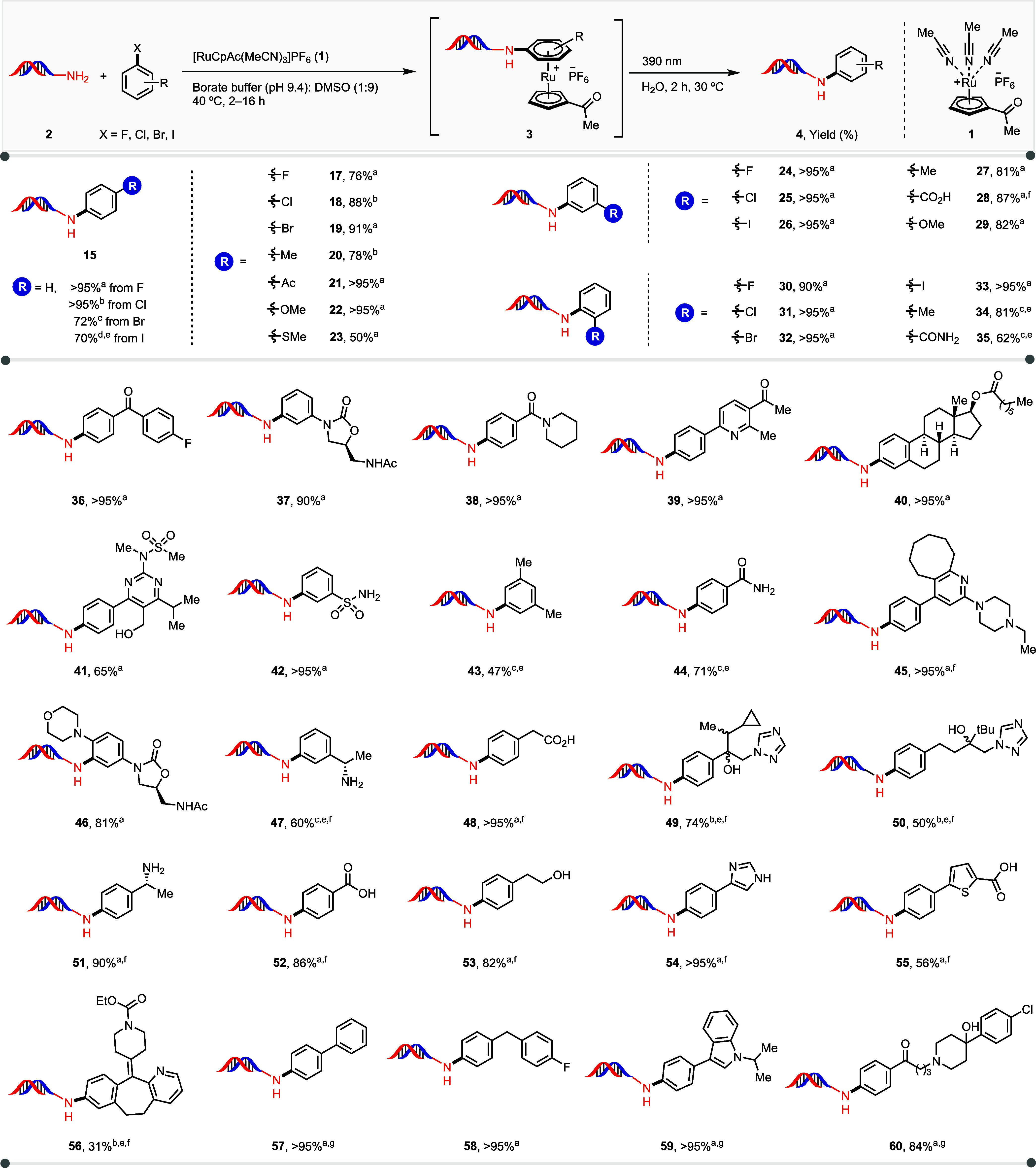
Substrate scope of *N*-arylation of various haloarenes. Aryl halide (*c* = 10 mM) and Ru complex **1** (*c* = 1.0 mM) in DMC, 80 °C, 2 h; then DNA-AOP-NH_2_ (**2**, *c* = 0.10 mM), in sodium borate buffer (*c* = 0.50 M, pH 9.4): DMSO (1:9), 40 °C, 2–16 h; then 390 nm irradiation in water (c = 0.10 mM). ^a^From fluoroarene. ^b^From chloroarene. ^c^From bromoarene. ^d^From iodoarene. ^e^40 equiv of **1**. ^f^HBF_4_·Et_2_O was used for the complexation step. ^g^30 equiv of Ru complex **1** and 10 equiv of arene were used (see Supporting Information pages, S63–S171).

Lewis-basic groups such as amines, pyridines, and others, which can potentially coordinate to the metal center, can inhibit η^6^ π-coordination. We hypothesized that the addition of an acid with a noncoordinating conjugate base could protonate these basic groups, preventing ruthenium coordination and, thereby, broadening the method’s substrate scope. Indeed, 1.3 equiv of HBF_4_·Et_2_O per coordinating group on the arene prevented ruthenium coordination and allowed the haloarene to participate in η^6^ π-coordination. Substrates including primary amines (**47**, **51**), alcohols (**53**), carboxylic acids (**28**, **48**, **52**, **55**), pyridine (**56**), imidazole (**54**), and triazoles (**49**, **50**) all proved compatible with the transformation. Due to the intrinsic properties of η^6^-arene chemistry, the metal center coordinates to the most electron-rich arene in a molecule. For instance, in the case of 4-fluorobiphenyl (**57**), ruthenium predominantly coordinated to the phenyl ring lacking fluorine. Introduction of a second ruthenium fragment, by simply adding an additional equivalent of **1**, resulted in a productive reaction with >95% yield. This strategy is also adaptable to more complex substrates containing more than one coordinating arene within the molecule, including haloperidol (**60**) and an indole derivative **59**, each giving yields over 80%.

The synthesis of DNA-encoded libraries typically relies on a split-and-pool strategy to generate diverse and structurally rich chemical libraries. This approach often requires a bifunctional starting material that can undergo further functionalization in subsequent library cycles. Amidation is one of the most frequently employed reactions in DEL synthesis due to its high efficiency and compatibility with bifunctional building blocks.^5,12^ For instance, Fmoc- or Boc-protected amino acids serve as ideal starting materials because they can be selectively deprotected in later steps, enabling further derivatization through various chemical transformations. We therefore extended our investigation to the *N*-arylation of various primary and secondary amine-DNA conjugates, initially synthesized via amidation (Figure 5). To study the *N*-arylation of amine-DNA conjugates, we employed the fluorobenzene-ruthenium complex **11** as substrate. Both natural (**62**, **64**, **67**–**68**, **74**) and non-natural (**61**, **63**, **65**–**66**) amino acids, yielded the *N*-arylated product in over 90% efficiency in most cases. DNA-conjugated cyclic secondary amines (**69**–**75**) are among the most effective nucleophiles, with yields over 80% in all the cases except for **72** which formed in 69% yield. Chemoselectivity in favor of aliphatic amines over other nucleophilic groups was observed in all cases, such as compared to alcohols (**72**) and phenols (**64**). The selectivity of amine over the phenol functional group in tyrosine, was confirmed by NMR analysis (see Supporting Information page, S28). The method exhibits high functional group tolerance for both the amine-DNA conjugates and the aryl halides; for example, carboxylic acids (**78**), ketones (**81**, **83**), and amines (**82**, **85**) are tolerated in bifunctional aryl halides for potential subsequent library expansion.

**Figure 5:**
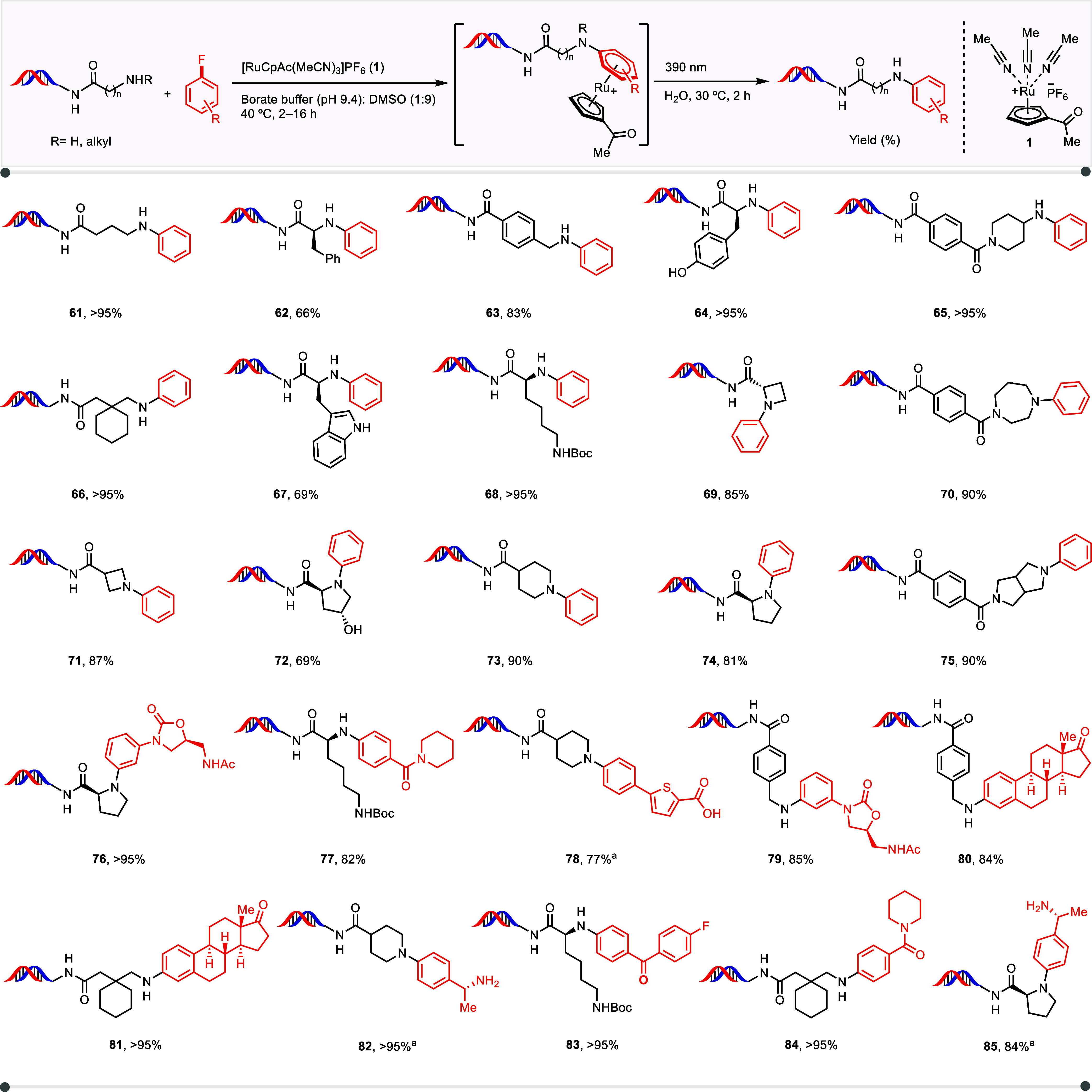
Substrate scope of *N*-arylation for various amine-DNA conjugates. Aryl halide (*c* = 10 mM) and Ru complex **1** (*c* = 1.0 mM) in DMC, 80 °C, 2 h; then amine-DNA conjugate (*c* = 0.10 mM), in sodium borate buffer (*c* = 0.50 M, pH 9.4): DMSO (1:9), 40 °C, 2–16 h; then 390 nm irradiation in water (*c* = 0.10 mM). ^a^HBF_4_·Et_2_O was used for the complexation step (see Supporting Information pages, S171–S221).

As a proof of principle, we synthesized an 18-membered mock library by combining three representative amine-DNA conjugates with six different haloarenes in a split and pool approach. All 18 library members could be identified by mass spectrometry and were formed with full conversion to product in most cases (see Supporting Information, pages S255–S259).

Maintaining DNA integrity during every on-DNA step is essential for accurate hit identification in DEL synthesis because the DNA tag functions as a molecular barcode that links each small molecule to its identity in terms of genetic code. Chemical degradation or mutation of the DNA following on-DNA reactions can result in false positives, false negatives, and unreliable sequencing data.^33^ To ensure compatibility, we assessed DNA integrity by qPCR analysis and confirmed the stability of the DNA throughout the reaction sequence of S_N_Ar (see Supporting Information, page S244). In addition, DNA ligation efficiency after *N*-arylation of an amine-DNA conjugate was confirmed by gel electrophoresis (see Supporting Information pages, S248–S253).

## Conclusions

In conclusion, we report the development of a novel, air-stable ruthenium reagent, [RuCpAc­(MeCN)_3_]­PF_6_ (**1**), for the efficient coupling of various haloarenes to achieve the first ruthenium-mediated on-DNA *N*-arylation of amine-DNA conjugates via S_N_Ar. The ruthenium reagent **1** activates haloarenes for *N*-arylation, analogous to the way amide coupling reagents activate carboxylic acids for acylation reactions. This work addresses a limitation of C–N cross coupling in the presence of oligonucleotides and demonstrates the potential of η^6^ π-arene reactivity in biological systems and synthesis in DNA-encoded libraries in particular.

## Supplementary Material



## Notes

Experimental procedures, spectroscopic data, LC-MS chromatograms, and NMR spectra of all products (PDF)

## References

[1] Brenner S., Lerner R. A. (1992). Encoded combinatorial chemistry. Proc. Natl. Acad. Sci. U.S.A..

[2] Satz A. L., Cai J., Chen Y., Goodnow R., Gruber F., Kowalczyk A., Petersen A., Naderi-Oboodi G., Orzechowski L., Strebel Q. (2015). DNA Compatible Multistep Synthesis and Applications
to DNA Encoded Libraries. Bioconjugate Chem..

[3] Fair R. J., Walsh R. T., Hupp C. D. (2021). The expanding
reaction toolkit for
DNA-encoded libraries. Bioorg. Med. Chem. Lett..

[4] Shi Y., Wu Y.-r., Yu J.-q., Zhang W.-n., Zhuang C.-l. (2021). DNA-encoded
libraries (DELs): a review of on-DNA chemistries and their output. RSC Adv..

[5] Fitzgerald P. R., Paegel B. M. (2021). DNA-Encoded Chemistry: Drug Discovery from a Few Good
Reactions. Chem. Rev..

[6] Brown D. G., Boström J. (2016). Analysis of
Past and Present Synthetic Methodologies
on Medicinal Chemistry: Where Have All the New Reactions Gone?. J. Med. Chem..

[7] Lu X., Roberts S. E., Franklin G. J., Davie C. P. (2017). On-DNA Pd and Cu
promoted C–N cross-coupling reactions. MedChemComm.

[8] de
Pedro Beato E., Priego J., Gironda-Martínez A., González F., Benavides J., Blas J., Martín-Ortega M. D., Toledo M. Á., Ezquerra J., Torrado A. (2019). Mild and Efficient
Palladium-Mediated C–N Cross-Coupling Reaction between DNA-Conjugated
Aryl Bromides and Aromatic Amines. ACS Comb.
Sci..

[9] Chen Y.-C., Faver J. C., Ku A. F., Miklossy G., Riehle K., Bohren K. M., Ucisik M. N., Matzuk M. M., Yu Z., Simmons N. (2020). C–N
Coupling of DNA-Conjugated (Hetero)­aryl
Bromides and Chlorides for DNA-Encoded Chemical Library Synthesis. Bioconjugate Chem..

[10] Chheda P. R., Simmons N., Schuman D. P., Shi Z. (2022). Palladium-Mediated
C–N Coupling of DNA-Conjugated (Hetero)­aryl Halides with Aliphatic
and (Hetero)­aromatic Amines. Org. Lett..

[11] Xu H., Ma F., Wang N., Hou W., Xiong H., Lu F., Li J., Wang S., Ma P., Yang G., Lerner R. A. (2019). DNA-Encoded
Libraries: Aryl Fluorosulfonates as Versatile Electrophiles Enabling
Facile On-DNA Suzuki, Sonogashira, and Buchwald Reactions. Adv. Sci..

[12] Franzini R. M., Randolph C. (2016). Chemical space of DNA-encoded
libraries. J. Med. Chem..

[13] Castan I. F. S. F., Madin A., Pairaudeau G., Waring M. J. (2022). Scope of on-DNA
nucleophilic aromatic substitution on weakly-activated heterocyclic
substrates for the synthesis of DNA-encoded libraries. Bioorg. Med. Chem..

[14] Clark M. A., Acharya R. A., Arico-Muendel C. C. (2009). Design, synthesis and
selection of DNA-encoded small-molecule libraries. Nat. Chem. Biol..

[15] Wang D.-Y., Wen X., Xiong C.-D., Zhao J.-N., Ding C.-Y., Meng Q., Zhou H., Wang C., Uchiyama M., Lu X.-J., Zhang A. (2019). Non-transition metal-mediated diverse aryl–heteroatom bond
formation of arylammonium salts. iScience.

[16] Krishnamurthy R. (2012). Role of pKa
of nucleobases in the origins of chemical evolution. Acc. Chem. Res..

[17] Hall H. K. (1957). Correlation of the base strengths of amines. J. Am. Chem. Soc..

[18] Biscoe M. R., Barder T. E., Buchwald S. L. (2007). Electronic effects on the selectivity
of Pd-catalyzed C–N bond-forming reactions using biarylphosphine
ligands: the competitive roles of amine binding and acidity. Angew. Chem., Int. Ed..

[19] Sunesson Y., Limé E., Nilsson Lill S. O., Meadows R. E., Norrby P.-O. (2014). Role of
the base in Buchwald–Hartwig amination. J. Org. Chem..

[20] Williams L. J., Bhonoah Y., Wilkinson L. A., Walton J. W. (2021). As Nice as π:
Aromatic Reactions Activated by π-Coordination to Transition
Metals. Chem. - Eur. J..

[21] Perekalin D. S., Kudinov A. R. (2014). Cyclopentadienyl ruthenium complexes with naphthalene
and other polycyclic aromatic ligands. Coord.
Chem. Rev..

[22] Beyzavi M. H., Mandal D., Strebl M. G., Neumann C. N., D’Amato E. M., Chen J., Hooker J. M., Ritter T. (2017). ^18^F-Deoxyfluorination
of phenols via Ru π-complexes. ACS Cent.
Sci..

[23] Schulte T., Wang Z., Li C.-C., Hamad A., Waldbach F., Pampel J., Petzold R., Leutzsch M., Bahns F., Ritter T. (2024). Ruthenium phenoxo complexes: an isolobal ligand to
Cp with improved properties. J. Am. Chem. Soc..

[24] Williams L. J., Bhonoah Y., Walton J. W. (2022). Enolate
S_N_Ar of unactivated
arenes via [(η^6^-arene)­RuCp]^+^ intermediates. Chem. Commun..

[25] Dembek A. A., Fagan P. J. (1996). Synthesis of (η^5^-pentamethylcyclopentadienyl)­ruthenium
π-complexes of heterocycles by nucleophilic substitution. Organometallics.

[26] Pearson A. J., Park J. G. (1992). Model studies on the carboxylate-binding pocket analogues
of vancomycin using arene-ruthenium chemistry. J. Org. Chem..

[27] Pearson A. J., Lee K. (1994). A formal total synthesis of the ACE inhibitor K-13: an application
of arene-ruthenium chemistry to complex chemical synthesis. J. Org. Chem..

[28] Janetka J. W., Rich D. H. (1995). Synthesis of peptidyl ruthenium π-arene complexes:
application to the synthesis of cyclic biphenyl ether peptides. J. Am. Chem. Soc..

[29] Janetka J. W., Rich D. H. (1997). Total synthesis of the cyclic biphenyl ether peptides
K-13 and OF4949-III via S_N_Ar macrocyclization of peptidyl
ruthenium π-arene complexes. J. Am. Chem.
Soc..

[30] Bao Y., Xing M., Matthew N., Chen X., Wang X., Lu X. (2024). Macrocyclizing DNA-linked
peptides via three-component cyclization
and photoinduced chemistry. Org. Lett..

[31] Vinogradova E. V., Zhang C., Spokoyny A. M., Pentelute B. L., Buchwald S. L. (2015). Organometallic palladium reagents
for cysteine bioconjugation. Nature.

[32] Mallek A. J., Pentelute B. L., Buchwald S. L. (2021). Selective *N*-arylation
of *p*-aminophenylalanine in unprotected peptides with
organometallic palladium reagents. Angew. Chem.,
Int. Ed..

[33] Wang H., Zhao G., Zhang T., Li Y., Zhang G., Li Y. (2023). Comparative study of DNA barcode integrity evaluation approaches
in the early-stage development of DNA-compatible chemical transformation. ACS Pharmacol. Transl. Sci..

