# Supplementary material for: Ruthenium-Mediated N‑Arylation for DNA-Encoded Libraries
Source: J Am Chem Soc. 2025 Sep 11;147(38):35011–8. doi: 10.1021/jacs.5c11842 (PMC12464987; doi:10.1021/jacs.5c11842)
Supplement: Supplementary file 1 [file ja5c11842_si_001.pdf]

SUPPORTING INFORMATION

Ruthenium-Mediated *N*-Arylation for DNA-Encoded Libraries

Suraj Kanoo[1,2,‡], Eduardo de Pedro Beato[1,‡], Tim Schulte[1], Lara Vogelsang[3], Luca Torkowski[1], Felix Waldbach[1], Philipp Hartmann[1], Riya Kayal[1], Karl-Josef Dietz[3], and Tobias Ritter[1]*

[1] Max-Planck-Institut für Kohlenforschung, Kaiser-Wilhelm-Platz 1, 45470 Mülheim an der Ruhr, Germany.

[2] Institute of Organic Chemistry, RWTH Aachen University, Landoltweg 1, 52074 Aachen, Germany.

[3] Biochemistry and Physiology of Plants, Faculty of Biology, Bielefeld University, Universitätsstraße 25, 33615 Bielefeld, Germany.

‡These authors contributed equally: Suraj Kanoo, Eduardo de Pedro Beato

*E-mail: ritter@kofo.mpg.de

TABLE OF CONTENTS

TABLE OF CONTENTS 1

MATERIALS AND METHODS 8

Starting Materials 8

Solvents 9

Chromatography 9

NMR Spectroscopy 10

Mass spectrometry 10

Liquid chromatography–mass spectrometry (LC–MS) 10

Gel electrophoresis of DNA conjugates 11

Quantitative polymerase chain reaction (qPCR) 11

Melting point 12

Miscellaneous 12

EXPERIMENTAL DATA 13

Preparation and reactions of various ruthenium complexes and small molecules 13

Synthesis of NaCp ligand **S2** 13

Synthesis of NaCpAc ligand **S3** 14

Synthesis of ruthenium CpAc complex **S4** 15

Synthesis of ruthenium CpAc complex **1** 17

Synthesis of ruthenium Cp* complex **5** 18

Synthesis of ruthenium Cp complex **6** 18

Synthesis of ruthenium CpAc complex **7** 19

Synthesis of ruthenium CpAc complex **S7** 20

Synthesis of ruthenium CpAc complex **S8** 21

Synthesis of ruthenium CpAc complex **S9** 21

Synthesis of *N*-arylated ruthenium CpAc complex **S10** 22

Synthesis of ketal **S12** 23

Synthesis of oligonucleotide mimic **10** 24

Synthesis of ruthenium CpAc complex **11** 24

Synthesis of aniline **12** 25

Synthesis of oxidative addition complex (OAC) **13** 26

Synthesis of aniline **14** 27

Synthesis of *N*-arylated tyrosine derivative **S14** 28

ON-DNA REACTIONS 29

Preparation of Stock solutions: 29

Preparation of $HP-NH_2$ stock solution **SR-01** in water 29
Preparation of DMT-MM stock solution **SR-02** in water 29
Preparation of HATU stock solution **SR-03** in DMA 29
Preparation of $MgCl_2$ stock solution **SR-04** in water 29
Preparation of NaOAc stock solution **SR-05** in water 29
Preparation of NaCl stock solution **SR-06** in water 29
Preparation of piperidine stock solution (20% v/v) **SR-07** in DMF 29
Preparation of DIPEA stock solution **SR-08** in DMA 30
Preparation of sodium borate buffer (pH 9.4, c = 0.50 M) 30

Functionalization of DNA conjugates 31

Preparation of DNA–AOP–NH_2 (**2**) stock solution **SD-01** in water 31
General procedures for the preparation of DNA-conjugated substrates 33
General procedure for *N*-arylation of amine-DNA conjugates with aryl fluorides 36

Preparation of DNA-conjugated substrates 37

DNA-conjugated substrate **S15** 37
DNA-conjugated substrate **S16** 39
DNA-conjugated substrate **S17** 40
DNA-conjugated substrate **S18** 42
DNA-conjugated substrate **S19** 43
DNA-conjugated substrate **S20** 45
DNA-conjugated substrate **S21** 46
DNA-conjugated substrate **S22** 48
DNA-conjugated substrate **S23** 50
DNA-conjugated substrate **S24** 51
DNA-conjugated substrate **S25** 53
DNA-conjugated substrate **S26** 54
DNA-conjugated substrate **S27** 56
DNA-conjugated substrate **S28** 57
DNA-conjugated substrate **S29** 59
DNA-conjugated substrate **S30** 61

ON-DNA *N*-ARYLATION 63

General protocol for the preparation of η^6- ruthenium π-arene complex stock solutions (in situ) 63

Complexation method without acid: 63
Complexation method with acid: 64

N-arylation of haloarenes with DNA-AOP-NH_2 (**2**) 65

Synthesis of DNA-conjugate **15** (from fluorobenzene) 65

Synthesis of DNA-conjugate **15** (from chlorobenzene) 67
Synthesis of DNA-conjugate **15** (from bromobenzene) 69
Synthesis of DNA-conjugate **15** (from iodobenzene) 71
Synthesis of DNA-conjugate **17** 73
Synthesis of DNA-conjugate **18** 75
Synthesis of DNA-conjugate **19** 77
Synthesis of DNA-conjugate **20** 79
Synthesis of DNA-conjugate **21** 82
Synthesis of DNA-conjugate **22** 84
Synthesis of DNA-conjugate **23** 86
Synthesis of DNA-conjugate **24** 88
Synthesis of DNA-conjugate **25** 90
Synthesis of DNA-conjugate **26** 92
Synthesis of DNA-conjugate **27** 95
Synthesis of DNA-conjugate **28** 97
Synthesis of DNA-conjugate **29** 99
Synthesis of DNA-conjugate **30** 101
Synthesis of DNA-conjugate **31** 103
Synthesis of DNA-conjugate **32** 106
Synthesis of DNA-conjugate **33** 108
Synthesis of DNA-conjugate **34** 110
Synthesis of DNA-conjugate **35** 112
Synthesis of DNA-conjugate **36** 114
Synthesis of DNA-conjugate **37** 117
Synthesis of DNA-conjugate **38** 119
Synthesis of DNA-conjugate **39** 121
Synthesis of DNA-conjugate **40** 123
Synthesis of DNA-conjugate **41** 126
Synthesis of DNA-conjugate **42** 128
Synthesis of DNA-conjugate **43** 130
Synthesis of DNA-conjugate **44** 132
Synthesis of DNA-conjugate **45** 134
Synthesis of DNA-conjugate **46** 137
Synthesis of DNA-conjugate **47** 139
Synthesis of DNA-conjugate **48** 141
Synthesis of DNA-conjugate **49** 143
Synthesis of DNA-conjugate **50** 146
Synthesis of DNA-conjugate **51** 148

Synthesis of DNA-conjugate **52** 150
Synthesis of DNA-conjugate **53** 153
Synthesis of DNA-conjugate **54** 155
Synthesis of DNA-conjugate **55** 157
Synthesis of DNA-conjugate **56** 160
Synthesis of DNA-conjugate **57** 162
Synthesis of DNA-conjugate **58** 164
Synthesis of DNA-conjugate **59** 166
Synthesis of DNA-conjugate **60** 169
N-arylation of amine-DNA conjugates 171
In situ formation of fluorobenzene-ruthenium stock solution: 171
N-arylation of DNA-conjugate **S15** 172
N-arylation of DNA-conjugate **S16** 174
N-arylation of DNA-conjugate **S17** 176
N-arylation of DNA-conjugate **S18** 178
N-arylation of DNA-conjugate **S20** 180
N-arylation of DNA-conjugate **S21** 182
N-arylation of DNA-conjugate **S22** 184
N-arylation of DNA-conjugate **S23** 186
N-arylation of DNA-conjugate **S24** 188
N-arylation of DNA-conjugate **S25** 190
N-arylation of DNA-conjugate **S26** 192
N-arylation of DNA-conjugate **S27** 194
N-arylation of DNA-conjugate **S28** 196
N-arylation of DNA-conjugate **S29** 198
N-arylation of DNA-conjugate **S30** 200
Synthesis of DNA-conjugate **76** 202
Synthesis of DNA-conjugate **77** 204
Synthesis of DNA-conjugate **78** 206
Synthesis of DNA-conjugate **79** 208
Synthesis of DNA-conjugate **80** 210
Synthesis of DNA-conjugate **81** 212
Synthesis of DNA-conjugate **82** 214
Synthesis of DNA-conjugate **83** 216
Synthesis of DNA-conjugate **84** 217
Synthesis of DNA-conjugate **85** 219
COMPUTATIONAL STUDIES 222

Electrophilicity of ruthenium complexes 222
Computational details 222
Computational data 222
Electrophilicity values 222
Optimized structures with x, y, z coordinates (Å) 224
[η^6(PhCl)RuCpMe$_5$]$^+$ 224
[η^6(PhCl)RuCpCH(Me)(OMe)]$^+$ 225
[η^6(PhCl)RuCp]$^+$ 227
[η^6(PhCl)RuCpCF$_3$]$^+$ 228
[η^6(PhCl)RuCpCN]$^+$ 229
[η^6(PhCl)RuCpAc]$^+$ 230
[η^6(PhCl)RuCpCO$_2$Me]$^+$ 231

EXPERIMENTAL STUDIES 234
Reaction of DNA-AOP-NH$_2$ (**2**) with different complexes 234
Chemoselectivity of DNA-AOP-NH$_2$ (**2**) towards C–N cross coupling reaction 235
Control experiment 237
Co-injection experiment 240
N-arylation of ligated DNA-conjugate **S33** 244
GEL electrophoresis protocol 245
qPCR analysis of DNA conjugates 246
Ligation efficiency 248
NMR reaction monitoring 254
Proof-of-concept mock-library synthesis 255
Quantitative NMR (qNMR) of ruthenium complex **1** 260

X-RAY CRYSTALLOGRAPHIC ANALYSIS 261
Ruthenium complex **1** (CCDC = 2432119) 261

SPECTROSCOPIC DATA 263
^{1}H NMR spectrum of **S4** 263
^{13}C NMR spectrum of **S4** 264
^{19}F NMR spectrum of **S4** 265
^{31}P NMR spectrum of **S4** 266
^{1}H NMR spectrum of **1** 267
^{13}C NMR spectrum of **1** 268
^{19}F NMR spectrum of **1** 269

^{31}P NMR spectrum of **1** ... 270
^{1}H NMR spectrum of **5** ... 271
^{13}C NMR spectrum of **5** ... 272
^{19}F NMR spectrum of **5** ... 273
^{1}H NMR spectrum of **6** ... 274
^{13}C NMR spectrum of **6** ... 275
^{19}F NMR spectrum of **6** ... 276
^{31}P NMR spectrum of **6** ... 277
^{1}H NMR spectrum of **7** ... 278
^{13}C NMR spectrum of **7** ... 279
^{19}F NMR spectrum of **7** ... 280
^{31}P NMR spectrum of **7** ... 281
^{1}H NMR spectrum of **S7** ... 282
^{13}C NMR spectrum of **S7** ... 283
^{19}F NMR spectrum of **S7** ... 284
^{31}P NMR spectrum of **S7** ... 285
^{1}H NMR spectrum of **S8** ... 286
^{13}C NMR spectrum of **S8** ... 287
^{19}F NMR spectrum of **S8** ... 288
^{31}P NMR spectrum of **S8** ... 289
^{1}H NMR spectrum of **S9** ... 290
^{13}C NMR spectrum of **S9** ... 291
^{19}F NMR spectrum of **S9** ... 292
^{31}P NMR spectrum of **S9** ... 293
^{1}H NMR spectrum of **S10** ... 294
^{13}C NMR spectrum of **S10** ... 295
^{19}F NMR spectrum of **S10** ... 296
^{31}P NMR spectrum of **S10** ... 297
^{1}H NMR spectrum of **S14** ... 298
^{13}C NMR spectrum of **S14** ... 299
^{1}H NOESY NMR spectrum of **S14** ... 300
^{1}H {^{15}N} HMBC NMR spectrum of **S14** ... 301
^{1}H NMR spectrum of **S12** ... 302
^{13}C NMR spectrum of **S12** ... 303
^{1}H NMR spectrum of **10** ... 304
^{13}C NMR spectrum of **10** ... 305
^{1}H NMR spectrum of **11** ... 306
^{13}C NMR spectrum of **11** ... 307

^{19}F NMR spectrum of **11** 308
^{31}P NMR spectrum of **11** 309
^{1}H NMR spectrum of **12** 310
^{13}C NMR spectrum of **12** 311
^{1}H NMR spectrum of **12** (Deuterated sample) 312
^{13}C NMR spectrum of **12** (Deuterated sample) 313
^{1}H {^{13}C} HMBC NMR spectrum of **12** 314
^{1}H {^{13}C} HSQC NMR spectrum of **12** 315
^{1}H ROESY NMR spectrum of **12** 316
^{1}H {^{15}N} HMBC NMR spectrum of **12** 317
^{1}H COSY NMR spectrum of **12** 318
^{1}H NMR spectrum of **14** 319
^{13}C NMR spectrum of **14** 320
^{1}H {^{13}C} HSQC NMR spectrum of **14** 321
^{1}H {^{13}C} HMBC NMR spectrum of **14** 322
^{1}H {^{15}N} HMBC NMR spectrum of **14** 323
^{1}H COSY NMR spectrum of **14** 324
^{1}H ROESY NMR spectrum of **14** 325

References 326

MATERIALS AND METHODS

Starting Materials

All substrates were used as received from the commercial suppliers:

Material	Vendor	Purity
N,N-Dimethylacetamide (DMA)	Iris Biotech	>99%
Fmoc-L-Tyr-OH	Iris Biotech	99%
Magnesium sulfate	Fisher Scientific	99%
Piperidine	Iris Biotech	>99%
Sodium chloride	Chemsolute	>99%
Triethylamine (TEA) puriss. p.a., ≥99.5% (GC)	Sigma-Aldrich	>99.5%
Hexafluoroisopropanol (HFIP)	Fluorochem	>99.5%
N-[(Dimethylamino)-1*H*-1,2,3-triazolo-[4,5-*b*]pyridin-1-ylmethylene]-*N*-methylmethanaminium hexafluorophosphate *N*-oxide (HATU)	Sigma-Aldrich	97%
N,N-Diisopropylethylamine (DIPEA)	Sigma-Aldrich	>99%
4-(4,6-Dimethoxy-1,3,5-triazin-2-yl)-4-methylmorpholinium chloride (DMT-MM)	Sigma-Aldrich	97%
Dimethyl sulfoxide (DMSO)	Iris Biotech	99%
Agarose	Bio-Rad	>99.5%
Sodium bicarbonate	Acros	99%
Sodium carbonate	Acros	99%
Sodium diethyldithiocarbamate trihydrate (30.5–32.5 wt.%, Na as Na_2SO_4)	Sigma-Aldrich	n/a
Sodium sulfate (anhydrous)	VWR	>99%
Tetrafluoroboric acid diethyl ether complex (50–55% w/w HBF_4)	Fisher Scientific	n/a
Palladium acetate	In-house	98%
Fmoc-15-amino-4,7,10,13-tetraoxapentadecanoic acid (Fmoc-AOP)	BLDpharm	98%
N-Methylmorpholine (NMM)	Sigma-Aldrich	99%
5'-Chloro-5'-deoxyadenosine	BLDpharm	97%
Fluorobenzene	Sigma-Aldrich	99%
Bromobenzene	TCI Chemicals	>99%
Iodobenzene	BLDpharm	98%
Silver(I) trifluoromethanesulfonate	BLDpharm	98%

(Trimethylsilyl)methylmagnesium chloride solution (1M in diethyl ether)	Sigma-Aldrich	n/a
Di-*tert*-butyl(2',4',6'-triisopropyl-3,6-dimethoxy-[1,1'-biphenyl]-2-yl)phosphine (t-BuBrettPhos)	BLDpharm	98%
2,2-Dimethoxypropane	Alfa Aesar	98%
p-Toluenesulfonic acid monohydrate	Sigma-Aldrich	>98.5%
1,3-Diaminopropane	Sigma-Aldrich	>99%
Acetone	Fisher Scientific	99.8%
Potassium hexafluorophosphate	Fisher Scientific	99%
Methyl acetate	Alfa Aesar	99%
L-Tyrosine methyl ester	Sigma-Aldrich	98%
Dimethylcarbonate (DMC)	Sigma-Aldrich	99%
Ethanol	OQEMA	98%
Boric acid	Sigma-Aldrich	>99.5%
Acetonitrile	Fisher Scientific	>99.9%

DNA headpiece HP–NH_2 (5’- /5Phos/GAGTCA/iSp9/iUniAmM/iSp9/TGACTCCC-3’) was purchased from LGC, Biosearch Technologies.

DNA for ligation including Primer–Tag1–Tag2–Tag3–Tag4–ClosingPrimer (5’- /5Phos/AAATCGATGTGTTC CGCAAGAAGCCTGGTAAGCGGAGAAAGGTCGTTACGATGCCCGGTCTACNNNNNNNNNNNNCTGATGG CGCGAGGGAGGC/GTAGACCGGGCATCGTAACGACCTTTCTCCGCTTACCAGGCTTCTTGCGGAACACA TCGATTTGG-3’) was purchased from Integrated DNA Technologies, Inc.

T4 ligase and 10× ligation buffer were purchased from New England BioLabs Inc.

Solvents

Water used to prepare buffers and as solvent was of ultra-high quality (UHQ) grade (18.2 MΩ·cm^{-1}). Methanol (>99%) was purchased from Sigma-Aldrich, dichloromethane (>99%) and acetonitrile (>99%) were purchased from Fisher Scientific.

Chromatography

Thin layer chromatography (TLC) was performed using EMD TLC plates pre-coated with 250 µm thickness silica gel 60 F254 plates and visualized by fluorescence quenching under UV light and $KMnO_4$ stain. Flash column chromatography was performed using silica gel (40–63 µm particle size) purchased from Geduran®.

NMR Spectroscopy

Chemical shifts are reported in ppm with the solvent residual peak as the internal standard. For ^{1}H NMR: $CDCl_3$, δ 7.26; D_2O, δ 4.79; CD_3OD, δ 3.31; $(CD_3)_2SO$, δ 2.50; CD_3CN, δ 1.94. For ^{13}C NMR: $CDCl_3$, δ 77.2; CD_3OD, δ 41.0; $(CD_3)_2SO$, δ 39.5; CD_3CN, δ 1.3[1]. ^{19}F NMR spectra were referenced using a unified chemical shift scale based on the ^{1}H resonance of tetramethylsilane (1% v/v solution in the respective solvent). Data are reported as follows: s = singlet, d = doublet, t = triplet, q = quartet, quint/p = quintet/pentaplet, m = multiplet, br = broad; coupling constants in Hz.

NMR spectra of small molecules were recorded on the following instruments:

1. Bruker Avance III 500 spectrometer equipped with a BBFO probe head, operating at 500 MHz, 471 MHz, and 126 MHz, for ^{1}H, ^{19}F, and ^{13}C acquisitions, respectively.
2. Bruker Avance III 600 spectrometer equipped with a triple-channel “TCI” cryogenic probehead (Bruker GmbH, Rheinstetten) operating at 600 MHz, 565 MHz, and 151 MHz for ^{1}H, ^{19}F, and ^{13}C respectively. All experiments used standard Bruker pulse sequences with standard parameter sets found in libraries of Topspin 3.6.

Mass spectrometry

High resolution Mass Spectrometry (HRMS) experiments for small molecules were performed on a Thermo Scientific™ Q Exactive Plus or a Thermo Scientific™ Q Exactive GC Orbitrap device.

Liquid chromatography–mass spectrometry (LC–MS)

Analytical LC–MS measurements of DNA conjugates were performed on an Agilent 1290 Infinity II system utilizing an AdvanceBio Oligonucleotide column, 50 × 4.6 mm, 2.7 µm, at 50 °C, and a flow rate = 0.4 mL · min^{-1}. The following HPLC methods were used for all DNA measurements.

HPLC method A: linear gradient from 10:90 v/v (MeOH : 100 mM HFIP, 10 mM triethylamine in H_2O) to 50:50 v/v (MeOH : 100 mM HFIP, 10 mM triethylamine in H_2O) over 4 min, followed by an isocratic run for 5 minutes with 50:50 v/v (MeOH : 100 mM HFIP, 10 mM triethylamine in H_2O), followed by a gradient from 50:50 v/v (MeOH : 100 mM HFIP, 10 mM trimethylamine in water in H_2O) to 10:90 v/v (MeOH : 100 mM HFIP, 10 mM triethylamine in H_2O) over 1 minute.

HPLC method B: linear gradient from 10:90 v/v (MeOH : 100 mM HFIP, 10 mM triethylamine in H_2O) to 50:50 v/v (MeOH : 100 mM HFIP, 10 mM triethylamine in H_2O) over 4 min, followed by an isocratic run for 1 minute with 50:50 v/v (MeOH : 100 mM HFIP, 10 mM triethylamine in H_2O), followed by a gradient from 50:50 v/v (MeOH : 100 mM HFIP, 10 mM triethylamine in H_2O) to 10:90 v/v (MeOH : 100 mM HFIP, 10 mM triethylamine in H_2O) over 1 minute.

Unless otherwise stated, conversions of the DNA conjugates were calculated by measuring the integration of the peaks of the diode array detection (DAD) UV absorbance at 260 nm of the LC–MS traces, assuming complete DNA recovery and identical UV absorbance for all DNA conjugates[2]. All absorbance signals in the total ion current (TIC) chromatogram that originate from DNA conjugates (molecular weight > 1000 g/mol)

were considered. Typically, observable signals in TIC chromatogram include, $[M-H_4]^{4-}$, $[M-H_5]^{5-}$, $[M-H_6]^{6-}$, $[M-H_7]^{7-}$, $[M-H_8]^{8-}$, and $[M-H_9]^{9-}$. In the cases where an absorbance of a small molecule impurity (molecular weight < 1000 g/mol) overlaps with absorbances of DNA conjugates, conversion was calculated by integration of the TIC chromatograms corresponding to the DNA conjugates after the reaction and the DNA conjugate starting material, respectively. Impurities present prior to the analyzed reaction were not considered.

MS-parameters:

MS system name: Agilent infinity Lab LC/MSD single quadrupole (Model: G6125B).

Threshold: 50 and 150

Fragmentor: 70

Drying gas flow (l/min): Actual (12.0 l/min), setpoint (12.0 l/min) and maximum (13.0 l/min).

Drying gas temperature (°C): Actual (333 °C), setpoint (333 °C) and maximum (350 °C).

Nebulizer Pressure (psig): Actual (35 psig), setpoint (35 psig) and maximum (60 psig).

Capillary Voltage (V): Positive (3000 V) and negative (3000 V).

Gel electrophoresis of DNA conjugates

Gel electrophoresis was carried out as instructed in the manual of Bio-Rad utilizing the Bio-Rad Laboratories ChemiDocMP system[3]. Image Lab Version 6.1.0 build 7 software from Bio-Rad Laboratories Inc. was used for analysis and processing of gel electrophoresis data.

Reagents used in the DNA Gel electrophoresis workflow:

Bio-Rad Laboratories 10× TBE Buffer (Tris/boric acid/EDTA).

Bio-Rad Laboratories Certified low range Ultra Agarose.

Bio-Rad Laboratories EZ Load 20 base pairs (bp) Molecular Ruler.

Bio-Rad Laboratories Nucleic Acid sample loading buffer, 5×.

CarlRoth SYBR® Green DNA dye, 11× conc., for electrophoresis, ready-to-use.

Devices used in the DNA Gel electrophoresis workflow:

Bio-Rad Laboratories ChemiDocMP.

Bio-Rad Laboratories Mini-Sub® Cell GT.

Bio-Rad Laboratories PowerPac Basic considered.

Quantitative polymerase chain reaction (qPCR)

Quantitive PCR was performed on a qPCR cycler MyiQTM Optics Module-Thermocycler from Bio-Rad Laboratories. PCR-Mastermix was prepared using KAPA SYBR® FASTqPCR Master Mix.

Primers for qPCR:

- Forward primer (5’ 565 Cla Primer): 5’-TGA CTC CCA AAT CGA TGT G -3’
- Reverse primer (3’ 454 Short Primer): 5’-GCC TCC CTC GCG CCA -3’

Melting point

Melting point measurements were performed using a Büchi M-565 automatic melting point apparatus. To determine the approximate melting range, the temperature was initially increased at a rate of 15 °C min^{-1}. Subsequently, a heating rate of 1 °C min^{-1} was applied near the expected melting range for accurate determination. Three independent measurements were recorded and averaged for accuracy.

Miscellaneous

DNA-conjugated reactions were conducted in an Eppendorf ThermoMixer® C. DNA desalting and rebuffering was performed with AMICON® filter units from Sigma Aldrich. DNA concentrations were determined by A_{260} absorption using a Thermo Scientific™ NanoDrop™ One^{C}.

EXPERIMENTAL DATA

Preparation and reactions of various ruthenium complexes and small molecules

Synthesis of NaCp ligand S2

The NaCp (**S2**) ligand was synthesized using a modified literature procedure[4]:

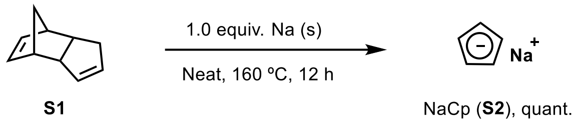

Under an ambient atmosphere, a 250-mL two-necked round-bottomed flask was equipped with a Teflon-coated magnetic stirring bar. To the central neck a reflux condenser was attached, that was connected to the Schlenk line by using a gas adapter. The second neck was closed with a glass stopper. The set up was flame-dried under high-vacuum and refilled with argon three times. Then, the flask was charged with **S1** (100 mL, 94.0 g, 711 mmol, 6.24 equiv.) under a stream of argon. Sodium (2.62 g, 114 mmol, 1.00 equiv.) was transferred to a 250 mL beaker containing hexanes (100 mL) and cut into small pieces (~0.5 cm) under the hexane layer using a knife to prevent oxidation. The Na-pieces were quickly added to the flask under a stream of argon and the hexane was discarded. The Na and **S1** mixture was stirred at 23 °C for 5 min under an argon atmosphere before heating at 160 °C (oil bath temperature) in an oil bath for 12 hours while stirring. After 12 hours, full consumption of the Na and precipitation of a colorless solid was observed. The oil bath was removed and the mixture cooled to 23 °C. Subsequently, the solid was filtered off using an inert filter frit (Fig. S1) and washed with dry pentane (3 × 20 mL). The frit was transferred to a nitrogen-filled glovebox and the off-white solid transferred to a 20 mL borosilicate vial to give NaCp (**S2**) in quant. yield (10.0 g, 114 mmol), which was stored in the glovebox at −20 °C. The obtained NMR spectra of the product are in good accordance with the reported literature[4].

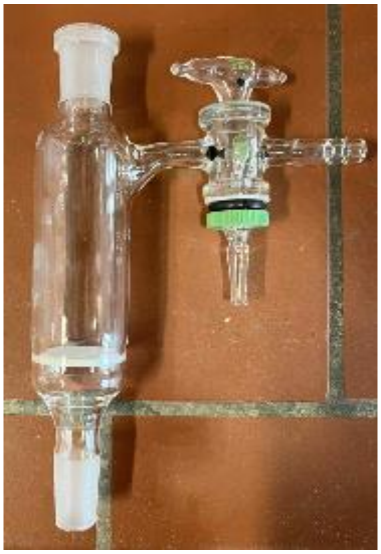

Fig S1. Picture of the inert filter frit used for the filtration of NaCp (**S2**).

NMR Spectroscopy:

^{1}H NMR (600 MHz, DMSO) δ 5.34 (s, 5H).

^{13}C NMR (151 MHz, DMSO) δ 103.0.

Synthesis of NaCpAc ligand S3

The NaCp (**S2**) ligand was synthesized using a modified literature procedure[5]:

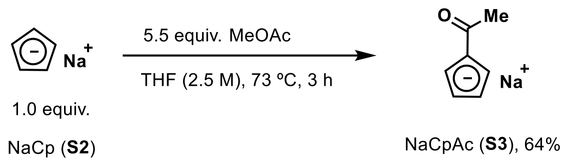

In a nitrogen-filled glovebox, NaCp (**S2**) (3.52 g, 40.0 mmol, 1.00 equiv.) was weighed into a pressure tube with Schlenk line attachment, equipped with a Teflon-coated magnetic stirring bar and dry THF (20 mL, c = 2.0 M) was then added. The vessel was closed, taken out of the glovebox and freshly distilled MeOAc (17.5 mL, 16.3 g, 220 mmol, 5.50 equiv.) was added dropwise over a period of 5 min by syringe under a stream of argon. The vessel was closed and the mixture was heated at 73 °C (oil bath temperature) for 3 hours behind a blast shield. Precipitation of a colorless solid was observed. After cooling to 23 °C, the suspension was decanted to a 100 mL Schlenk flask under a stream of argon and the mixture concentrated to ~10 mL by evaporation through the Schlenk line. Subsequently, dry diethylether (20 mL) was added to induce further precipitation. The resulting colorless solid was filtered off using an inert filter frit with Schlenk line attachment under a stream of argon (Fig S2), washed with dry diethylether (3 × 30 ml), and dried under high vacuum. The inert filter frit was introduced in a nitrogen-filled glovebox and the colorless solid was transferred to a 20 mL borosilicate vial for further storage in the glovebox at −20 °C. NaCpAc (**S3**) was obtained as a colorless solid (5.18 g, 25.6 mmol, 64%) and used for the next step without further purification. The obtained NMR spectra of the product are in good accordance with the reported literature[5].

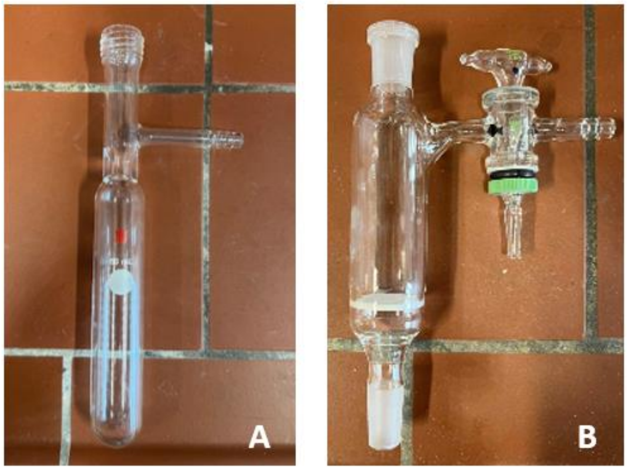

Fig S2. A. Picture of the pressure tube with schlenk line attachment. B. Picture of the inert filter frit used for the filtration of NaCpAc (**S3**).

NMR Spectroscopy:

^{1}H NMR (500 MHz, D_2O) δ 6.72 – 6.60 (m, 2H), 6.23 – 6.15 (m, 2H), 2.33 (s, 3H).

^{13}C NMR (126 MHz, D_2O) δ 188.8, 122.7, 117.3, 117.3, 115.6, 115.5, 23.9.

Synthesis of ruthenium CpAc complex S4

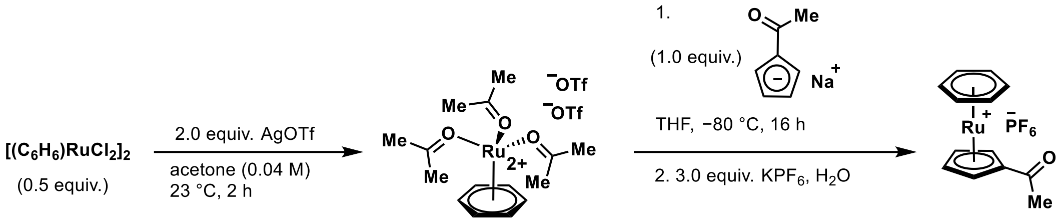

S4, 51%

A flame-dried 100 mL Schlenk flask equipped with a Teflon-coated magnetic stir bar under argon atmosphere was charged with $[(C_6H_6)RuCl_2]_2$ (1.00 g, 2.00 mmol, 0.500 equiv.) and AgOTf (2.06 g, 8.00 mmol, 2.00 equiv.). The flask was evacuated and filled with argon twice. Then, dry acetone (45 mL, c = 44 mM) was added and the suspension was stirred for 2 hours at 23 °C in the dark by covering the flask with aluminum foil. Meanwhile, a 2-necked 250 mL flask was equipped with a Teflon-coated magnetic stir bar and an inert filter frit was connected to the Schlenk line via the second neck (Fig. S3). The set up was flame-dried under vacuum and set under an argon atmosphere. Subsequently the suspension was filtered into the 250 mL flask (Fig. S3, A) by slowly applying vacuum to the collection flask over a period of 5 min. The filter frit was flushed with 10 mL dry acetone and then closed with a glass stopper. Meanwhile, a dropping funnel was flame-dried under an argon atmosphere and flushed with argon for 5 min. The inert filter frit was replaced with the dropping funnel and the dropping funnel was then closed with a septum. In a nitrogen-filled glovebox, NaCpAc (**S3**) (809 mg, 4.00 mmol, 1.00 equiv.) was weighed into a Schlenk flask and THF/DCM 3:1 (30 mL) was added. The Schlenk flask was closed with a septum, taken out of the glovebox and attached to the Schlenk line. Under argon, the NaCpAc suspension was transferred to the dropping funnel via syringe. The 250 mL Schlenk flask was cooled to −80 °C using a dry ice/acetone bath and the flask was covered with aluminum foil. The suspension was added dropwise at a rate of 1 drop every 3 seconds to the ruthenium precursor solution while the solution was stirred (Fig. S3, B). While adding the suspension, more DCM/THF 1:1 (50 mL in total) was added to the dropping funnel and the suspension was mixed thoroughly, until all NaCpAc was added. After the addition was completed, the reaction mixture was stirred in the dark and slowly warmed to 23 °C overnight (bath is not removed) (Fig. S3, C). Subsequently, the brown suspension was filtered into a 250 mL round-bottomed flask and the solution concentrated under reduced pressure. The dark residue was dissolved in Milli-Q water (150 mL), stirred for 5 min. and then filtered into another 250 mL round-bottomed flask. The remaining dark residue was washed with Milli-Q water (3 × 10 mL) and precipitation from the combined aqueous phases was induced by addition of solid KPF_6 while the mixture was stirred (2.21 g, 12.0 mmol, 3.00 equiv.). The flask was placed in a fridge with a set temperature of 4 °C for 3 hours to complete precipitation. The formed faint-yellow solid was filtered off and washed with Milli-Q water

(20 mL). Subsequently, the solid was dissolved in MeCN (7.5 mL), transferred to a 100 mL round-bottomed flask and precipitation induced by the addition of diethylether (80 mL). The formed colorless solid was filtered off, washed with Et_2O (3 × 10 mL) and dried under high vacuum to give the desired complex **S4** (440 mg, 1.02 mmol, 51%) as a colorless solid.

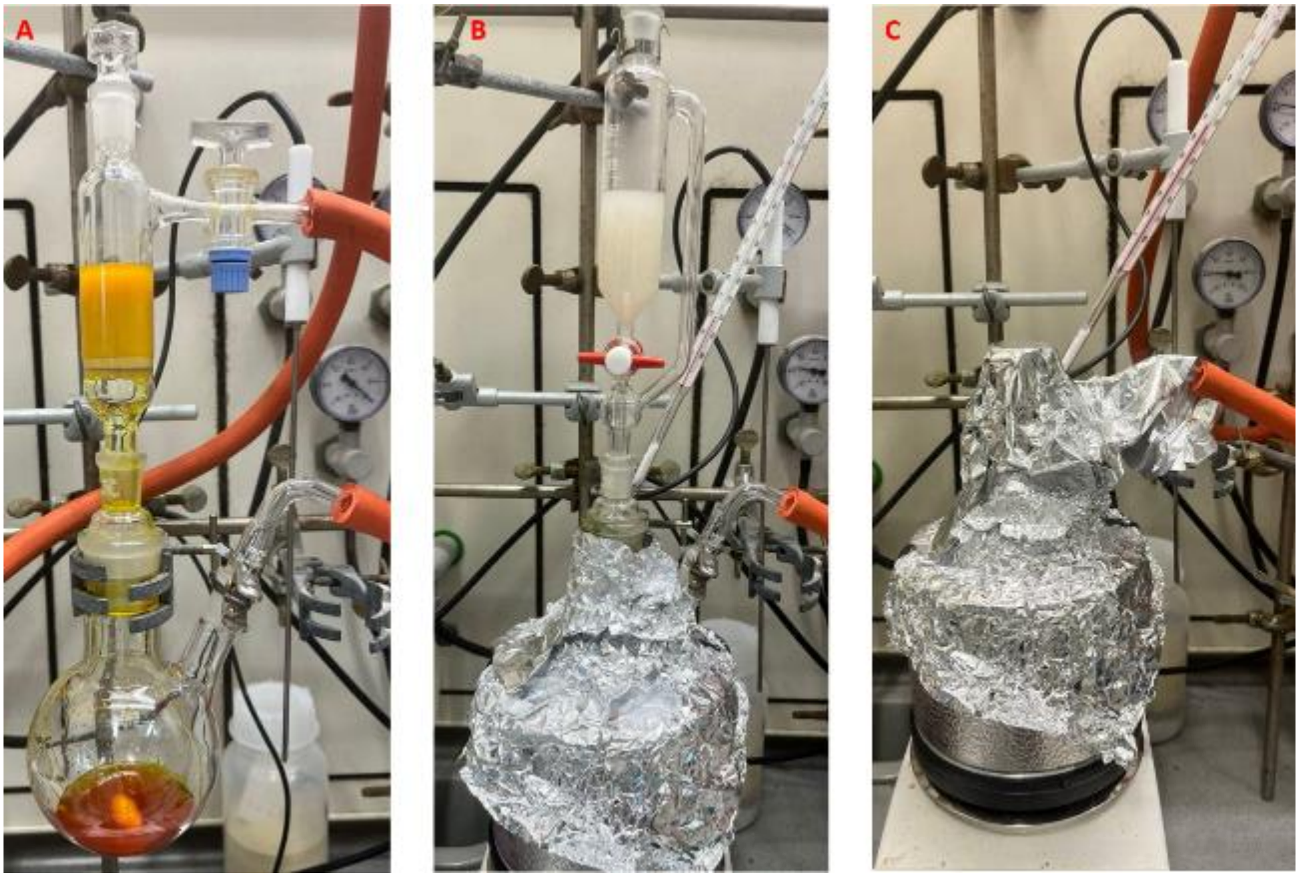

Fig S3. Pictures of the reaction set up. A = Filtration of the ruthenium precursor solution in acetone through an inert filter frit. B = Dropwise addition of NaCpAc (**S3**) suspension. C = Stirring and slowly warming to 23 °C after addition of NaCpAc (**S3**) suspension was completed.

NMR Spectroscopy:

^{1}**H NMR** (600 MHz, CD_3CN) δ 6.16 (s, 6H), 5.79 (t, *J* = 1.9 Hz, 2H), 5.48 (t, *J* = 1.9 Hz, 2H), 2.36 (s, 3H).

^{13}**C NMR** (151 MHz, CD_3CN) δ 197.0, 92.9, 88.3, 83.4, 80.9, 27.9.

^{19}**F NMR** (565 MHz, CD_3CN) δ −72.9 (d, *J* = 706.7 Hz).

^{31}**P NMR** (243 MHz, CD_3CN) δ −144.6 (hept, *J* = 706.5 Hz).

HRMS ESI (m/z) calculated for $C_{13}H_{13}ORu$ [M^+] 287.0004; found 287.0004; deviation: +0.07 ppm.

Synthesis of ruthenium CpAc complex 1

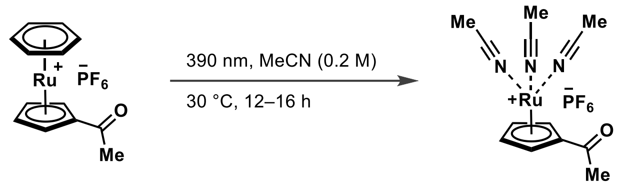

S4

1, 98%
$[RuCpAc(MeCN)_3]PF_6$

Under an ambient atmosphere, a 40 mL borosilicate vial equipped with a Teflon-coated magnetic stirring bar was charged with ruthenium complex **S4** (200 mg, 0.464 mmol, 1.00 equiv.) and acetonitrile (MeCN) (23 mL, c = 20 mM). The reaction mixture was purged with argon using a stainless steel needle for 2 min. The vial was closed with a screw cap, and the reaction mixture was irradiated for 16 hours with a 390 nm Kessil lamp (40 W) with constant stirring (500 rpm) and maintaining the temperature at approximately 30 °C through cooling with a fan. After 16 hours, the solvent was removed under reduced pressure using a rotary evaporator. To the resulting solid, approximately 1 mL of acetonitrile was added, resulting in a yellow solution. The acetonitrile solution was then slowly added dropwise (approximately one drop per second) to a 40 mL vial containing 30 mL of diethyl ether (Et_2O) to induce precipitation. The vial was then transferred to a centrifuge and centrifuged at 3000 × g for 5 min. The supernatant was decanted and 20 mL of pentane was added to the remaining yellow solid. The vial was sonicated for 30 seconds, transferred to a centrifuge and centrifuged at 3000 × g for 5 min. The supernatant was decanted, leaving a yellow solid, which was dried under high vacuum to afford ruthenium complex **1** as a fine crystalline yellow solid (217 mg, 0.456 mmol, 98%).

NMR Spectroscopy:

^{1}H NMR (600 MHz, CD_3CN) δ 5.08 (dd, *J* = 2.1, 1.5 Hz, 2H), 4.53 (dd, *J* = 2.1, 1.6 Hz, 2H), 2.22 (s, 3H), 1.96 (s, 9H).

^{13}C NMR (151 MHz, CD_3CN) δ 199.4, 127.5, 78.3, 72.5, 70.5, 27.9, 1.8.

^{19}F NMR (565 MHz, CD_3CN) δ −73.0 (d, *J* = 706.6 Hz).

^{31}P NMR (243 MHz, CD_3CN) δ −144.7 (hept, *J* = 706.4 Hz).

HRMS ESI (m/z) calculated for $C_{13}H_{16}N_3ORu$ $[M^+]$ 332.0337; found 332.0331; deviation: +0.4 ppm.

Melting point: 141.5 °C (decomposition).

Quantitative NMR (qNMR): 97.4 wt% purity.

Synthesis of ruthenium Cp* complex 5

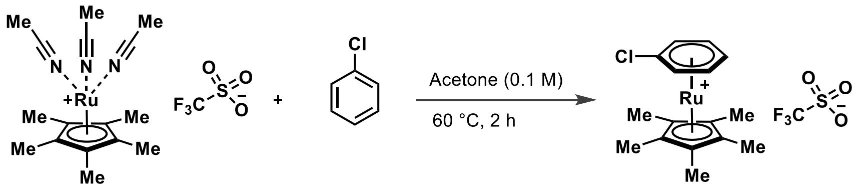

S5 10 equiv. **5**, 84%

[RuCp*(MeCN)$_3$]OTf

Under an ambient atmosphere, a 4 mL vial equipped with a Teflon-coated magnetic stirring bar was charged with the complex [RuCp*(MeCN)$_3$]OTf (**S5**) (20.0 mg, 39.3 µmol, 1.00 equiv.) and acetone (0.4 mL, c = 0.1 M). To the resulting reaction mixture, chlorobenzene (40 µL, 44 mg, 0.39 mmol, 10 equiv.) was added. The vial was closed with a screw cap and heated at 60 °C for 2 hours. After 2 hours, the reaction mixture was cooled to 23 °C, and the solvent was removed under reduced pressure using a rotary evaporator. Then, ~0.2 mL acetonitrile was added to the vial and the contents were mixed thoroughly using a glass Pasteur pipette to result in a solution The acetonitrile solution was then slowly added dropwise (approximately one drop per second) using a glass pipette to a 20 mL vial containing ~10 mL diethyl ether (Et_2O) to induce precipitation while stirring (800 rpm). The vial was then transferred to a centrifuge and centrifuged at 3000 × g for 5 min. The supernatant was decanted and the solid was dried under high vacuum to afford ruthenium Cp* complex **5** as an off-white solid (16.5 mg, 33.1 µmol, 84%).

NMR Spectroscopy:

^{1}H NMR (600 MHz, CD_3CN) δ 6.08 – 6.06 (m, 2H), 5.89 – 5.86 (m, 2H), 5.81 (tt, *J* = 5.7, 0.6 Hz, 1H), 1.95 (s, 15H).

^{13}C NMR (151 MHz, CD_3CN) δ 98.9, 89.4, 88.2, 87.9, 30.8, 10.4.

^{19}F NMR (565 MHz, CD_3CN) δ −79.3.

HRMS ESI (m/z) calculated for $C_{16}H_{20}ClRu$ [M^+] 349.0291; found 349.0289; deviation: +0.5 ppm.

Synthesis of ruthenium Cp complex 6

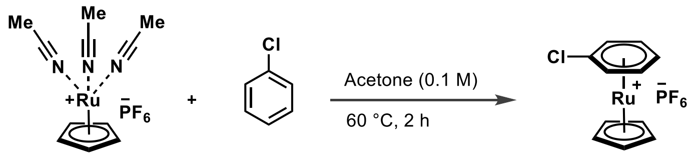

S6 10 equiv. **6**, 90%

[RuCp(MeCN)$_3$]PF$_6$

Under an ambient atmosphere, a 4 mL vial equipped with a Teflon-coated magnetic stirring bar was charged with the complex [RuCp(MeCN)$_3$]PF$_6$ (**S6**) (30.0 mg, 69.1 µmol, 1.00 equiv.) and acetone (0.7 mL, c = 0.1 M). To the resulting reaction mixture, chlorobenzene (70 µL, 78 mg, 0.69 mmol, 10 equiv.) was added. The vial

was closed with a screw cap and heated at 60 °C for 2 hours. After 2 hours, the reaction mixture was cooled to 23 °C, and the solvent was removed under reduced pressure using a rotary evaporator. Then, ~0.2 mL acetonitrile was added to the vial and the contents were mixed thoroughly using a glass Pasteur pipette to result in a solution The acetonitrile solution was then slowly added dropwise (approximately one drop per second) using a glass pipette to a 20 mL vial containing ~10 mL diethyl ether (Et_2O) to induce precipitation while stirring (800 rpm). The vial was then transferred to a centrifuge and centrifuged at 3000 × g for 5 min. The supernatant was decanted and the solid was dried under high vacuum to afford ruthenium Cp complex **6** as a light grey solid (26.3 mg, 62.0 µmol, 90%).

NMR Spectroscopy:

^{1}**H NMR** (500 MHz, CD_3CN) δ 6.55 (d, *J* = 6.2 Hz, 2H), 6.21 (t, *J* = 6.1 Hz, 2H), 6.09 (t, *J* = 5.8 Hz, 1H), 5.45 (s, 5H).

^{13}**C NMR** (151 MHz, CD_3CN) δ 106.5, 88.5, 86.7, 86.3, 83.3.

^{19}**F NMR** (565 MHz, CD_3CN) δ −73.0 (d, *J* = 706.1 Hz).

^{31}**P NMR** (243 MHz, CD_3CN) δ −144.6 (hept, *J* = 706.5 Hz).

HRMS ESI (m/z) calculated for $C_{11}H_{10}C_{11}Ru$ [M^+] 278.9509; found 278.9507; deviation: +0.6 ppm.

Synthesis of ruthenium CpAc complex 7

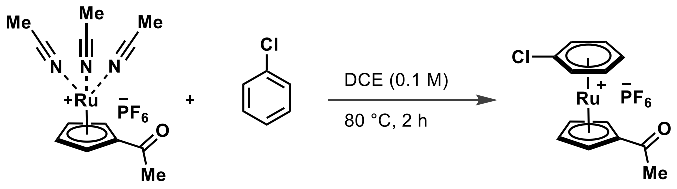

1
$[RuCpAc(MeCN)_3]PF_6$

10 equiv.

7, 98%

Under an ambient atmosphere, a 4 mL vial equipped with a Teflon-coated magnetic stirring bar was charged with the complex $[RuCpAc(MeCN)_3]PF_6$ (**1**) (10.0 mg, 21.0 µmol, 1.00 equiv.) and 1,2-dichloroethane (DCE) (0.2 mL, c = 0.1 M). To the resulting reaction mixture, chlorobenzene (21 µL, 23 mg, 0.21 mmol, 10 equiv.) was added. The vial was closed with a screw cap and heated at 80 °C for 2 hours. After 2 hours, the reaction mixture was cooled to 23 °C, and the solvent was removed under reduced pressure using a rotary evaporator. Then, ~0.2 mL acetonitrile was added to the vial and the contents were mixed thoroughly using a glass Pasteur pipette to result in a solution The acetonitrile solution was then slowly added dropwise (approximately one drop per second) using a glass pipette to a 20 mL vial containing ~10 mL diethyl ether (Et_2O) to induce precipitation while stirring (800 rpm). The vial was then transferred to a centrifuge and centrifuged at 3000 × g for 5 min. The supernatant was decanted and the solid was dried under high vacuum to afford ruthenium CpAc complex **7** as an off-white solid (9.6 mg, 21 µmol, 98%).

NMR Spectroscopy:

^{1}H NMR (600 MHz, CD_3CN) δ 6.56 – 6.54 (m, 2H), 6.28 – 6.25 (m, 2H), 6.15 – 6.12 (m, 1H), 5.87 (dd, *J* = 2.1, 1.8 Hz, 2H), 5.56 – 5.54 (m, 2H), 2.36 (s, 3H).

^{13}C NMR (151 MHz, CD_3CN) δ 196.0, 107.3, 94.4, 89.7, 88.2, 87.4, 85.2, 82.8, 28.2.

^{19}F NMR (565 MHz, CD_3CN) δ −72.9 (d, *J* = 706.3 Hz).

^{31}P NMR (243 MHz, CD_3CN) δ −144.6 (hept, *J* = 706.5 Hz).

HRMS ESI (m/z) calculated for $C_{13}H_{12}ClORu$ [M^+] 320.9620; found 320.9615; deviation: +0.8 ppm.

Synthesis of ruthenium CpAc complex S7

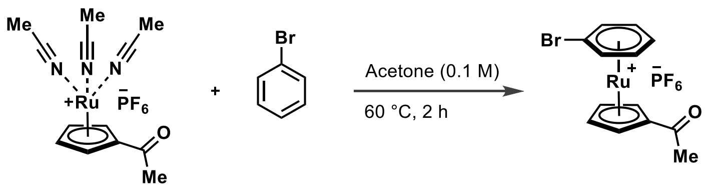

1 10 equiv. **S7**, 95%

Under an ambient atmosphere, a 4 mL vial equipped with a Teflon-coated magnetic stirring bar was charged with the complex [RuCpAc(MeCN)$_3$]PF_6 (**1**) (10.0 mg, 21.0 µmol, 1.00 equiv.) and acetone (0.2 mL, c = 0.1 M). To the resulting reaction mixture, bromobenzene (22 µL, 33 mg, 0.21 mmol, 10 equiv.) was added. The vial was closed with a screw cap and heated at 60 °C for 2 hours. After 2 hours, the reaction mixture was cooled to 23 °C, and the solvent was removed under reduced pressure using a rotary evaporator. Then, ~0.2 mL acetonitrile was added to the vial and the contents were mixed thoroughly using a glass Pasteur pipette to result in a solution The acetonitrile solution was then slowly added dropwise (approximately one drop per second) using a glass pipette to a 20 mL vial containing ~10 mL diethyl ether (Et_2O) to induce precipitation while stirring (800 rpm). The vial was then transferred to a centrifuge and centrifuged at 3000 × g for 5 min. The supernatant was decanted and the solid was dried under high vacuum to afford ruthenium CpAc complex **S7** as an off-white solid (10.2 mg, 20.0 µmol, 95%).

NMR Spectroscopy:

^{1}H NMR (600 MHz, CD_3CN) δ 6.63 – 6.57 (m, 2H), 6.24 – 6.20 (m, 2H), 6.18 – 6.15 (m, 1H), 5.85 (dd, *J* = 2.1, 1.7 Hz, 2H), 5.54 (dd, *J* = 2.2, 1.7 Hz, 2H), 2.37 (s, 3H).

^{13}C NMR (151 MHz, CD_3CN) δ 196.0, 94.6, 92.1, 92.1, 88.6, 87.5, 85.4, 83.0, 28.4.

^{19}F NMR (565 MHz, CD_3CN) δ −73.0 (d, *J* = 706.5 Hz).

^{31}P NMR (243 MHz, CD_3CN) δ −144.6 (hept, *J* = 706.5 Hz).

HRMS ESI (m/z) calculated for $C_{13}H_{12}BrORu$ [M^+] 364.9109; found 364.9105; deviation: +1.0 ppm.

Synthesis of ruthenium CpAc complex S8

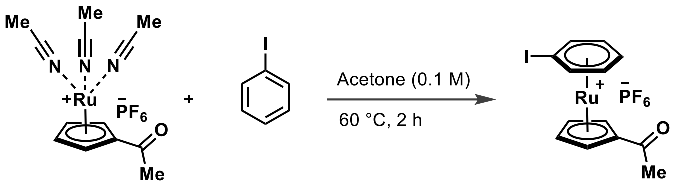

1 10 equiv. **S8,** 68%

Under an ambient atmosphere, a 4 mL vial equipped with a Teflon-coated magnetic stirring bar was charged with the complex [RuCpAc(MeCN)$_3$]PF$_6$ (**1**) (20.0 mg, 42.0 µmol, 1.00 equiv.) and acetone (0.4 mL, c = 0.1 M). To the resulting reaction mixture, iodobenzene (46 µL, 84 mg, 0.42 mmol, 10 equiv.) was added. The vial was closed with a screw cap and heated at 60 °C for 2 hours. After 2 hours, the reaction mixture was cooled to 23 °C, and the solvent was removed under reduced pressure using a rotary evaporator. Then, ~0.2 mL acetonitrile was added to the vial and the contents were mixed thoroughly using a glass Pasteur pipette to result in a solution The acetonitrile solution was then slowly added dropwise (approximately one drop per second) using a glass pipette to a 20 mL vial containing ~10 mL diethyl ether (Et$_2$O) to induce precipitation while stirring (800 rpm). The vial was then transferred to a centrifuge and centrifuged at 3000 × g for 5 min. The supernatant was decanted and the solid was dried under high vacuum to afford ruthenium Cp* complex **S8** as an off-white solid (16.0 mg, 28.7 µmol, 68%).

NMR Spectroscopy:

^{1}**H NMR** (600 MHz, CD$_3$CN) δ 6.65 – 6.56 (m, 2H), 6.27 – 6.16 (m, 2H), 6.14 – 6.07 (m, 1H), 5.80 (dd, *J* = 2.1, 1.7 Hz, 2H), 5.51 (dd, *J* = 2.2, 1.7 Hz, 2H), 2.37 (s, 3H).

^{13}**C NMR** (151 MHz, CD$_3$CN) δ 196.0, 96.7, 94.7, 88.9, 87.4, 85.6, 83.1, 56.8, 28.6.

^{19}**F NMR** (565 MHz, CD$_3$CN) δ −72.9 (d, *J* = 706.2 Hz).

^{31}**P NMR** (243 MHz, CD$_3$CN) δ −144.60 (hept, *J* = 706.5 Hz).

HRMS ESI (m/z) calculated for $C_{13}H_{12}IORu$ [M$^+$] 412.8970; found 412.8972; deviation: −0.3 ppm.

Synthesis of ruthenium CpAc complex S9

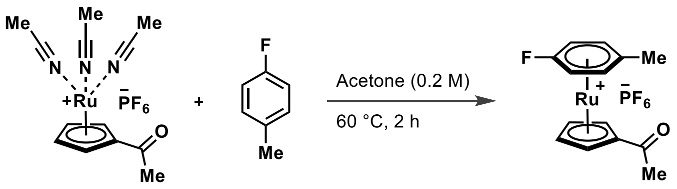

1 10 equiv. **S9,** 94%

Under an ambient atmosphere, a 4 mL vial equipped with a Teflon-coated magnetic stirring bar was charged with the complex [RuCpAc(MeCN)$_3$]PF$_6$ (**1**) (20.0 mg, 42.0 µmol, 1.00 equiv.) and acetone (0.4 mL, c = 0.1 M). To the resulting reaction mixture, 1-fluoro-1-methylbenzene (46 µL, 46 mg, 0.42 mmol, 10 equiv.) was added. The vial was closed with a screw cap and heated at 60 °C for 2 hours. After 2 hours, the reaction

mixture was cooled to 23 °C, and the solvent was removed under reduced pressure using a rotary evaporator. Then, ~0.2 mL acetonitrile was added to the vial and the contents were mixed thoroughly using a glass Pasteur pipette to result in a solution The acetonitrile solution was then slowly added dropwise (approximately one drop per second) using a glass pipette to a 20 mL vial containing ~10 mL diethyl ether (Et_2O) to induce precipitation while stirring (800 rpm). The vial was then transferred to a centrifuge and centrifuged at 3000 × g for 5 min. The supernatant was decanted and the solid was dried under high vacuum to afford ruthenium CpAc complex **S9** as an off-white solid (18.4 mg, 39.7 µmol, 94%).

NMR Spectroscopy:

^{1}**H NMR** (600 MHz, CD_3CN) δ 6.52 – 6.48 (m, 2H), 6.21 – 6.17 (m, 2H), 5.85 (dd, *J* = 2.1, 1.8 Hz, 2H), 5.53 (dd, *J* = 2.2, 1.7 Hz, 2H), 2.36 (s, 3H), 2.19 (s, 3H).

^{13}**C NMR** (151 MHz, CD_3CN) δ 196.5, 136.9 (d, *J* = 276.7 Hz), 103.7, 93.7, 88.1 (d, *J* = 6.5 Hz), 85.1, 82.3, 78.9 (d, *J* = 21.1 Hz), 28.0, 19.1 (d, *J* = 1.8 Hz).

^{19}**F NMR** (565 MHz, CD_3CN) δ −72.9 (d, *J* = 706.3 Hz), −140.7 (p, *J* = 3.2 Hz).

^{31}**P NMR** (243 MHz, CD_3CN) δ −144.6 (hept, *J* = 706.5 Hz).

HRMS ESI (m/z) calculated for $C_{14}H_{14}FORu$ $[M^+]$ 319.0065; found 319.0066; deviation: +0.3 ppm.

Synthesis of *N*-arylated ruthenium CpAc complex S10

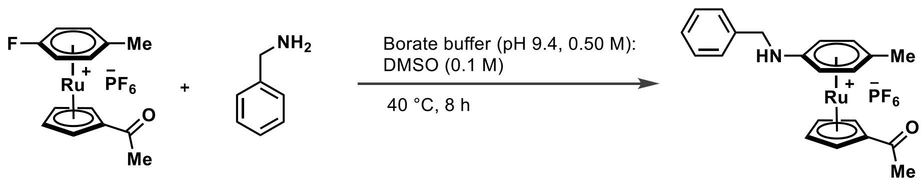

S9 2.2 equiv. **S10**, 56%

Under an ambient atmosphere, a 4 mL vial equipped with a Teflon-coated magnetic stirring bar was charged with ruthenium CpAc complex **S9** (10.0 mg, 21.6 µmol, 1.00 equiv.) and DMSO (0.2 mL, c = 0.1 M), followed by sodium borate buffer (22 µL, pH 9.4, c = 0.50 M). To the resulting reaction mixture added benzylamine (5.2 µL, 5.1 mg, 47 µmol, 2.2 equiv.). The vial was closed with a screw cap and heated at 40 °C for 8 hours. After 8 hours, the solvent was removed using Biotage V-10 evaporator. Then, 0.5 mL DCM was added to the vial and the contents were mixed thoroughly using a glass Pasteur pipette. The insoluble borate salts in the mixture was then filtered using a syringe filter to obtain a clear yellow solution, which was then slowly added dropwise (approximately one drop per second) to 20 mL vial containing approximately 10 mL pentane while stirring (800 rpm) to induce precipitation. The vial was then transferred to a centrifuge and centrifuged at 3000 × g for 5 min. The supernatant was decanted and the solid was dried under high vacuum to afford *N*-arylated ruthenium CpAc complex **S10** as an off-white solid (6.7 mg, 12 µmol, 56%).

NMR Spectroscopy:

^{1}**H NMR** (600 MHz, CD_3CN) δ 7.46 – 7.41 (m, 2H), 7.38 – 7.36 (m, 3H), 5.82 (dt, *J* = 6.8, 0.4 Hz, 2H),

5.64 (d, *J* = 6.8 Hz, 2H), 5.42 (dd, *J* = 2.2, 1.7 Hz, 2H), 5.04 (dd, *J* = 2.1, 1.7 Hz, 2H), 4.23 (d, *J* = 6.0 Hz, 2H), 2.27 (s, 3H), 2.08 (s, 3H).

^{13}C NMR (151 MHz, CD_3CN) δ 197.4, 137.4, 129.9, 128.9, 128.9, 127.5, 98.0, 91.4, 87.0, 82.6, 79.9, 69.4, 47.3, 27.9, 18.9.

^{19}F NMR (471 MHz, CD_3CN) δ −72.9 (d, *J* = 705.9 Hz).

^{31}P NMR (243 MHz, CD_3CN) δ −144.60 (hept, *J* = 706.5 Hz).

HRMS ESI (m/z) calculated for $C_{21}H_{22}NORu$ [M^+] 406.0739; found 406.0740; deviation: −0.2 ppm.

Synthesis of ketal S12

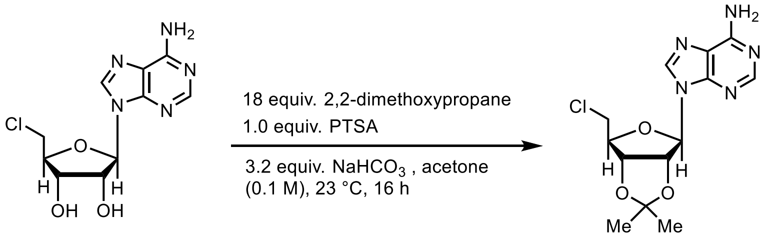

S11 **S12**, 96%

Under an ambient atmosphere, a 50 mL two neck round bottomed flask equipped with a Teflon-coated magnetic stirring bar was charged with 5'-chloro-5'-deoxyadenosine (**S11**) (1.00 g, 3.50 mmol, 1.00 equiv.) and acetone (35 mL, c = 0.1 M). To the above mixture was added 2, 2-dimethoxypropane (7.70 mL, 6.55 g, 63.0 mmol, 18.0 equiv), followed by p-toluenesulfonic acid (666 mg, 3.50 mmol, 1.00 equiv.). The mixture was then stirred at 23 °C for 16 hours. After 16 hours, solid sodium bicarbonate (945 mg, 11.2 mmol, 3.20 equiv.) was added to the above reaction mixture, and the mixture was stirred for another 30 min. The solvent was then removed under reduced pressure using a rotary evaporator. The residue was dissolved in dichloromethane (~50 mL) and transferred to a separatory funnel, followed by the addition of saturated aqueous $NaHCO_3$ solution (~50 mL). The layers were separated and the aqueous layer was extracted with dichloromethane (3 × 150 mL). The combined organic layers were washed with brine (50 mL) and were dried over anhydrous sodium sulfate. The solution was filtered and concentrated under reduced pressure using a rotary evaporator. The residue was purified by flash column chromatography on silica gel, eluting with 5% MeOH in DCM to afford the desired ketal **S12** as a colorless solid (1.10 g, 3.36 mmol, 96%).

R_f = 0.27 (5% MeOH in DCM).

NMR Spectroscopy:

^{1}H NMR (600 MHz, MeOD) δ 8.28 (s, 1H), 8.24 (s, 1H), 6.24 (d, *J* = 2.5 Hz, 1H), 5.51 (ddd, *J* = 6.3, 2.5, 0.5 Hz, 1H), 5.15 (ddd, *J* = 6.3, 2.9, 0.5 Hz, 1H), 4.48 – 4.40 (m, 1H), 3.83 (dd, *J* = 11.3, 7.1 Hz, 1H), 3.69 (dd, *J* = 11.3, 5.5 Hz, 1H), 1.62 (d, *J* = 0.7 Hz, 3H), 1.41 (d, *J* = 0.7 Hz, 3H).

^{13}C NMR (151 MHz, MeOD) δ 157.5, 154.1, 150.2, 141.8, 120.6, 115.6, 91.9, 87.9, 85.3, 84.1, 44.7,

27.4, 25.4.

HRMS ESI (m/z) calculated for $C_{13}H_{17}Cl_1N_5O_3$ [M+H$^+$] 326.1014; found 326.1013; deviation: +0.3 ppm.

Synthesis of oligonucleotide mimic 10

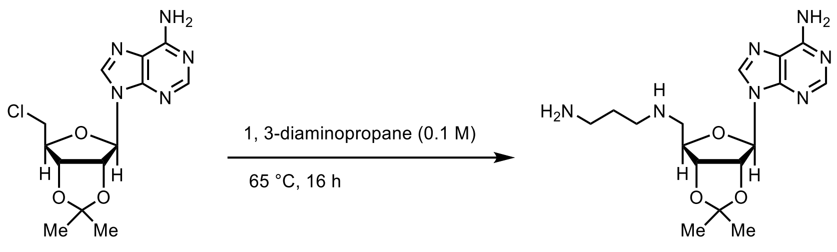

S12 **10**, 80%

Under an ambient atmosphere, a 20 mL vial equipped with a Teflon-coated magnetic stirring bar was charged with the compound **S12** (300 mg, 0.921 mmol, 1.00 equiv.), followed by dry 1,3-diaminopropane (9.2 mL, 8.1 g, 0.11 mol, c = 0.10 M). The resulting reaction mixture was heated at 65 °C for 16 hours. Next, the solvent was removed using Biotage V-10 evaporator. The residue was purified by flash column chromatography on silica gel, eluting with DCM/ NH_3, ca. 7M solution in methanol using a gradient from 1:0–85:15 to afford the desired oligonucleotide mimic **10** as a pale yellow oil (267 mg, 0.735 mmol, 80%). The adenosine derivative **10** when kept under vacuum for 16 hours, it turned into a colorless solid. The compound is hygroscopic in nature.

R$_f$ = 0.25 (DCM/ NH_3, ca. 7M solution in methanol, 85:15).

NMR Spectroscopy:

^{1}H NMR (600 MHz, MeOD) δ 8.28 (s, 1H), 8.22 (s, 1H), 6.16 (d, *J* = 2.8 Hz, 1H), 5.50 (dd, *J* = 6.4, 2.8 Hz, 1H), 5.01 (dd, *J* = 6.4, 3.4 Hz, 1H), 4.34 (td, *J* = 6.2, 3.4 Hz, 1H), 2.85 (d, *J* = 6.5 Hz, 2H), 2.66 – 2.50 (m, 4H), 1.60 (s, 3H), 1.59 – 1.54 (m, 2H), 1.38 (d, *J* = 0.6 Hz, 3H).

^{13}C NMR (151 MHz, MeOD) δ 157.5, 154.0, 150.4, 142.0, 120.7, 115.6, 91.6, 86.8, 84.9, 83.9, 52.5, 48.3, 40.5, 33.4, 27.5, 25.6.

HRMS ESI (m/z) calculated for $C_{16}H_{26}N_7O_3$ [M+H$^+$] 364.2091; found 364.2090; deviation: +0.4 ppm.

Synthesis of ruthenium CpAc complex 11

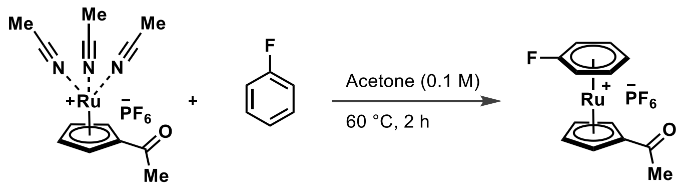

1 10 equiv. **11**, 94%

Under an ambient atmosphere, a 4 mL vial equipped with a Teflon-coated magnetic stirring bar was charged

with the complex [RuCpAc(MeCN)$_3$]PF$_6$ (**1**) (20.0 mg, 42.0 µmol, 1.00 equiv.) and acetone (0.4 mL, c = 0.1 M). To the resulting reaction mixture, fluorobenzene (40 µL, 41 mg, 0.42 mmol, 10 equiv.) was added. The vial was closed with a screw cap and heated at 60 °C for 2 hours. After 2 hours, the reaction mixture was cooled to 23 °C, and the solvent was removed under reduced pressure using a rotary evaporator. Then, ~0.2 mL acetonitrile was added to the vial and the contents were mixed thoroughly using a glass Pasteur pipette to result in a solution The acetonitrile solution was then slowly added dropwise (approximately one drop per second) using a glass pipette to a 20 mL vial containing ~10 mL diethyl ether (Et$_2$O) to induce precipitation while stirring (800 rpm). The vial was then transferred to a centrifuge and centrifuged at 3000 × g for 5 min. The supernatant was decanted and the solid was dried under high vacuum to afford ruthenium CpAc complex **11** as an off-white solid (17.8 mg, 39.6 µmol, 94%).

NMR Spectroscopy:

^{1}H NMR (600 MHz, CD$_3$CN) δ 6.58 – 6.52 (m, 2H), 6.26 – 6.22 (m, 2H), 6.06 – 6.02 (m, 1H), 5.88 (dd, *J* = 2.1, 1.7 Hz, 2H), 5.56 (dd, *J* = 2.2, 1.7 Hz, 2H), 2.37 (s, 3H).

^{13}C NMR (151 MHz, CD$_3$CN) δ 196.5, 137.7 (d, *J* = 277.8 Hz), 93.7, 87.3 (d, *J* = 6.4 Hz), 87.1, 84.7, 82.2, 79.5 (d, *J* = 20.9 Hz), 27.9.

^{19}F NMR (565 MHz, CD$_3$CN) δ −72.9 (d, *J* = 706.3 Hz), −137.3 (hept, *J* = 3.5 Hz).

^{31}P NMR (243 MHz, CD$_3$CN) δ −144.7 (hept, *J* = 706.3 Hz).

HRMS ESI (m/z) calculated for $C_{13}H_{12}F_1O_1Ru_1$ [M$^+$] 304.9910; found 304.9911; deviation: −0.2 ppm.

Synthesis of aniline 12

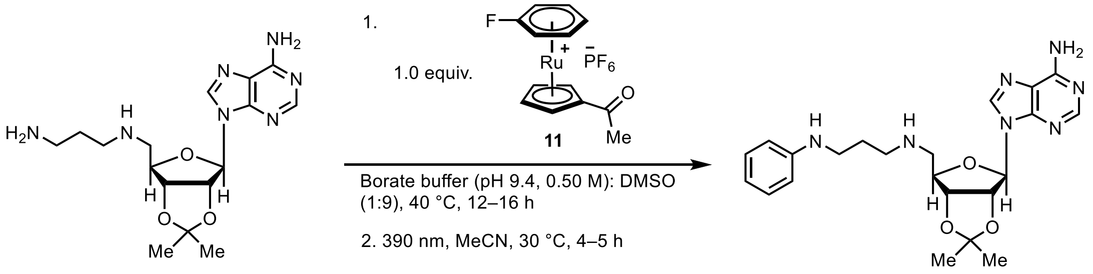

10 **12**, 83%

Under an ambient atmosphere, a 4 mL vial equipped with a Teflon-coated magnetic stirring bar was charged with adenosine derivative **10** (30.0 mg, 82.6 µmol, 1.00 equiv.) and fluorobenzene ruthenium complex **11** (37.1 mg, 82.6 µmol, 1.00 equiv.). Then, sodium borate buffer (83 µL, pH 9.4, c = 0.50 M) and DMSO (743 µL; final concentration c = 0.10 M, buffer/DMSO = 1:9 v/v) were added to the reaction vial. The vial was closed with a screw cap, and the mixture was stirred at 40 °C for 16 hours. After 16 hours, the reaction mixture was diluted with 0.8 mL of water and irradiated with a 390 nm Kessil lamp (40 W) for 2 hours with continuous stirring (500 rpm); the temperature was kept at approximately 30 °C through cooling with a fan. The solvent was removed using a Biotage V-10 evaporator. The residue was dissolved in ~7 mL of acetonitrile and irradiated with a 390 nm Kessil lamp (40 W) with constant stirring and maintaining the

temperature at approximately 30 °C by cooling with a fan for 2.5 hours. After irradiation, the solvent was removed under reduced pressure using a rotary evaporator. The residue was purified by flash column chromatography on silica gel, eluting with DCM/NH_3, ca. 7M solution in methanol, using a gradient from 99:1–90:10 to afford the *N*-arylated product **12** as a yellow oil (30.1 mg, 68.5 µmol, 83%).

R*f* = 0.14 (DCM/ NH_3, ca. 7M solution in methanol, 9:1).

NMR Spectroscopy:

^{1}H NMR (600 MHz, DMSO) δ 8.36 (s, 1H), 8.16 (s, 1H), 7.33 (s, 2H), 7.05 – 7.01 (m, 2H), 6.51 (dd, *J* = 8.7, 1.1 Hz, 2H), 6.48 (tt, *J* = 7.2, 1.1 Hz, 1H), 6.09 (d, *J* = 3.1 Hz, 1H), 5.53 (br, 1H), 5.47 (dd, *J* = 6.3, 3.1 Hz, 1H), 4.98 (dd, *J* = 6.3, 2.8 Hz, 1H), 4.22 (td, *J* = 5.9, 2.8 Hz, 1H), 3.33 (s, 2H), 3.06 – 2.91 (m, 2H), 2.73 (dd, *J* = 12.3, 5.9 Hz, 1H), 2.66 (dd, *J* = 12.3, 6.1 Hz, 1H), 2.58 – 2.55 (m, 2H), 1.66 – 1.60 (m, 2H), 1.54 (s, 3H), 1.32 (s, 3H).

^{13}C NMR (151 MHz, DMSO) δ 156.1, 152.7, 149.0, 148.9, 139.9, 128.8, 119.2, 115.3, 113.2, 111.9, 89.2, 85.0, 82.7, 82.2, 51.1, 47.3, 41.1, 28.9, 27.0, 25.2.

HRMS ESI (m/z) calculated for $C_{22}H_{30}N_7O_3$ [M+H$^+$] 440.2404; found 440.2408; deviation: −0.8 ppm.

Synthesis of oxidative addition complex (OAC) 13

The oxidative addition complex **13** was synthesized using a modified literature procedure[6]:

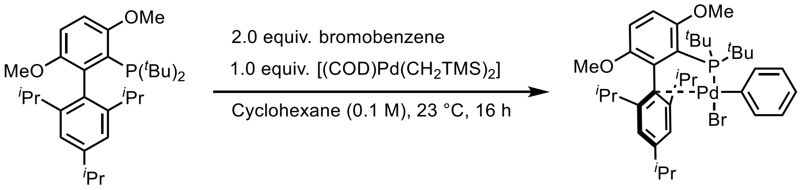

S13 **13**, 49%

Under an ambient atmosphere, an oven-dried 50 mL round bottomed flask equipped with a Teflon coated magnetic stirring bar was charged with the 2-(di-*tert*-butylphosphin)-2’, 4’, 6’-triisopropyl-3, 6-dimethoxy-1, 1’-biphenyl (**S13**) (113 mg, 0.233 mmol, 1.00 equiv.). The round bottomed flask was then brought into a nitrogen-filled glovebox and added bromobenzene (49 µL, 73 mg, 0.46 mmol, 2.0 equiv.), followed by cyclohexane (2.0 mL, c = 0.1 M). The reaction mixture was stirred vigorously for 1 min. to dissolve the ligand **S13**. Next, (COD)Pd(CH_2TMS)$_2$ (90.1 mg, 0.233 mmol, 1.00 equiv) was added to the reaction tube in one portion, which resulted in an immediate color change. The flask was then closed with a septum cap, removed from the glovebox, and the content was stirred for 16 hours at 23 °C. After 16 hours, pentane (6 mL) was added to the reaction mixture using a 10 mL syringe to induce precipitation. The reaction mixture was then transferred to a 50 mL Falcon tube and centrifuged at 3000 × g for 5 min. Next, the solvent was decanted into a separate 20 mL borosilicate vial, and the remaining solid was washed with pentane (~6 mL). The centrifugation and decantation process was repeated two additional times. The solid was dried under high-vacuum for 16 hours to afford the desired oxidative addition complex **13** as a yellow solid (86.3 mg, 0.115

mmol, 49%). The complex was then stored in a nitrogen filled glovebox. The obtained NMR spectra of the product are in good agreement with the reported literature[6].

NMR Spectroscopy:

^{1}H NMR (600 MHz, CD_2Cl_2) δ 7.08 – 7.04 (m, 4H), 6.94 (ddd, *J* = 9.0, 7.0, 2.5 Hz, 1H), 6.87 (d, *J* = 8.9 Hz, 1H), 6.78 – 6.74 (m, 2H), 6.67 (t, *J* = 7.1 Hz, 1H), 3.79 (s, 3H), 3.33 (s, 3H), 3.02 (p, 1H), 2.58 (hept, *J* = 6.8 Hz, 2H), 1.59 (d, *J* = 6.8 Hz, 6H), 1.38 (d, *J* = 14.9 Hz, 18H), 1.34 (d, *J* = 6.9 Hz, 6H), 0.82 (d, *J* = 6.6 Hz, 6H).

^{31}P NMR (243 MHz, CD_2Cl_2) δ 69.6.

Synthesis of aniline 14

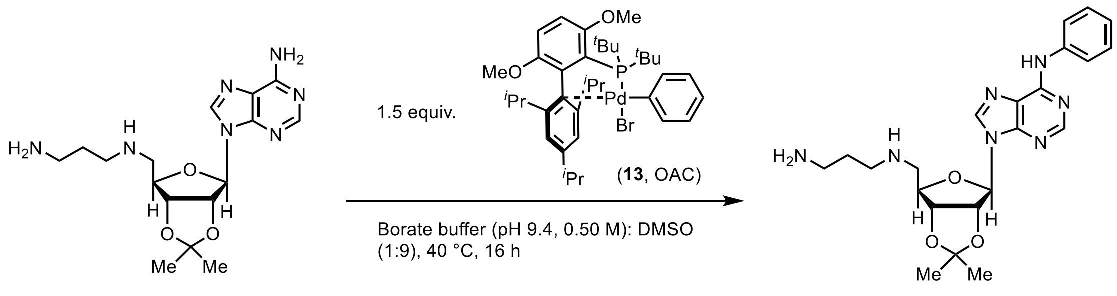

10 **14**, 87%

Under an ambient atmosphere, a 4 mL vial equipped with a Teflon-coated magnetic stirring bar was charged with OAC **13** (74.1 mg, 99.0 µmol, 1.50 equiv.) and adenosine derivative **10** (24.0 mg, 66.0 µmol, 1.00 equiv.). Then, sodium borate (66 µL, pH 9.4, c = 0.50 M) and DMSO (594 µL; final concentration c = 0.10 M, buffer/DMSO = 1:9 v/v) were added to the reaction vial. The vial was closed with a screw cap, and the mixture was stirred at 40 °C for 16 hours. After 16 hours, the solvent was removed using a Biotage V-10 evaporator. The residue was purified by flash column chromatography on silica gel, eluting with DCM/NH_3, ca. 7M solution in methanol, using a gradient from 98:2–90:10 to afford the desired *N*-arylated product **14** as a light brown solid (25.4 mg, 57.8 µmol, 87%).

R_f = 0.14 (DCM/ NH_3, ca. 7M solution in methanol, 9:1).

NMR Spectroscopy:

^{1}H NMR (600 MHz, DMSO) δ 8.55 (s, 1H), 8.42 (s, 1H), 7.94 (dd, *J* = 8.7, 1.2 Hz, 2H), 7.33 (dd, *J* = 8.6, 7.3 Hz, 2H), 7.05 (tt, *J* = 7.4, 1.2 Hz, 1H), 6.17 (d, *J* = 2.9 Hz, 1H), 5.50 (dd, *J* = 6.3, 2.9 Hz, 1H), 4.98 (dd, *J* = 6.3, 2.7 Hz, 1H), 4.24 (td, *J* = 6.0, 2.6 Hz, 1H), 2.76 – 2.63 (m, 2H), 2.58 – 2.52 (m, 2H), 2.49 – 2.40 (m, 2H), 1.55 (s, 3H), 1.47 – 1.40 (m, 2H), 1.34 (s, 3H).

^{13}C NMR (151 MHz, DMSO) δ 152.1, 152.1 149.1, 140.8, 139.5, 128.4, 122.7, 120.9, 120.3, 113.1, 89.5, 85.30, 82.9, 82.2, 51.2, 47.3, 40.1, 33.3, 27.0, 25.2.

HRMS ESI (m/z) calculated for $C_{22}H_{30}N_7O_3$ [M+H$^+$] 440.2404; found 440.2407; deviation: −0.6 ppm.

Synthesis of *N*-arylated tyrosine derivative S14

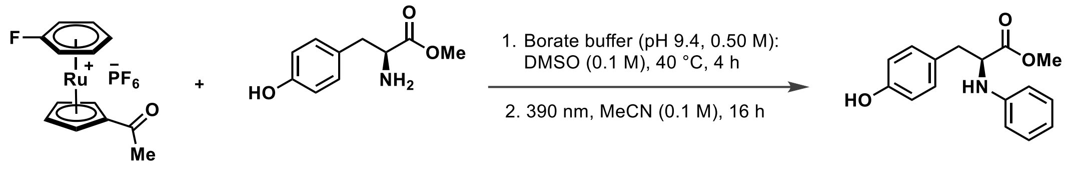

11 1.0 equiv. **S14**, 82%

Under an ambient atmosphere, a 4 mL vial equipped with a Teflon-coated magnetic stirring bar was charged with the ruthenium complex **11** (23.0 mg, 51.2 µmol, 1.00 equiv.) and DMSO (461 µL, c = 0.1 M), followed by sodium borate buffer (51 µL, pH 9.4, c = 0.50 M). To the resulting reaction mixture added the tyrosine derivative (10.0 mg, 51.2 µmol, 1.00 equiv.). The vial was closed using a screw cap and heated at 40 °C for 4 hours. After 4 hours, the solvent was removed using Biotage V-10 evaporator. Next, the residue was dissolved in MeCN (0.5 mL, c= 0.1 M). The acetonitrile reaction mixture was then irradiated with 390 nm Kessil lamp (40 W) for 16 hours while stirring and maintaining the temperature at approximately ~30 °C through cooling with a fan. Next, the solvent was removed under reduced pressure using a rotary evaporator. The residue was purified by flash column chromatography on silica gel, eluting with DCM/MeOH, using a gradient from 1:0–95:5 to afford the desired *N*-arylated product **S14** as a colorless solid (11.4 mg, 42.1 µmol, 82%).

R_f = 0.47 (DCM/ MeOH. 95:5).

NMR Spectroscopy:

^{1}H NMR (600 MHz, CD_3CN) δ 7.13 – 7.09 (m, 2H), 7.07 – 7.04 (m, 2H), 6.79 (s, 1H), 6.75 – 6.70 (m, 2H), 6.66 (tt, *J* = 7.3, 1.1 Hz, 1H), 6.59 (dd, *J* = 8.7, 1.1 Hz, 2H), 4.62 (d, *J* = 9.0 Hz, 1H), 4.26 (ddd, *J* = 9.0, 7.1, 6.3 Hz, 1H), 3.60 (s, 3H), 3.02 (dd, *J* = 13.8, 6.3 Hz, 1H), 2.95 (dd, *J* = 13.8, 7.1 Hz, 1H).

^{13}C NMR (151 MHz, CD_3CN) δ 174.8, 156.7, 148.2, 131.4, 130.2, 129.2, 118.6, 116.0, 114.0, 58.9, 52.4, 38.7.

HRMS ESI (m/z) calculated for $C_{16}H_{17}NO_3$ [M+Na$^+$] 294.1100; found 294.1098; deviation: +0.9 ppm.

ON-DNA REACTIONS

Preparation of Stock solutions:

Preparation of HP-NH_2 stock solution SR-01 in water

The HP-NH_2 stock solution **SR-01** in water (c = 5.0 mM, 10 mL) was prepared by dissolving in an ambient atmosphere the commercially available HP-NH_2 (50,000 nmol, MW = 4937.23 Da, purchased from LGC, Biosearch Technologies in a 15 mL Falcon tube) in 10 mL Milli-Q water.

Preparation of DMT-MM stock solution SR-02 in water

Under an ambient atmosphere, a 1.5 mL Eppendorf tube was charged with 4-(4,6-dimethoxy-1,3,5-triazin-2-yl)-4-methylmorpholinium chloride (DMT-MM) (110 mg, 0.397 mmol). To the Eppendorf tube was added 1.0 mL of Milli-Q water, resulting in the DMT-MM stock solution **SR-02** in water (c = 0.40 M).

Preparation of HATU stock solution SR-03 in DMA

Under an ambient atmosphere, a 1.5 mL Eppendorf tube was charged with *O*-(7-azabenzotriazol-1-yl)-*N*,*N*,*N′*,*N′*-tetramethyluronium hexafluorophosphate (HATU) (95 mg, 0.25 mmol). To the Eppendorf tube was added 500 µL of DMA, resulting in the HATU stock solution **SR-03** in DMA (c = 0.50 M).

Preparation of $MgCl_2$ stock solution SR-04 in water

Under an ambient atmosphere, $MgCl_2$ (4.0 mg, 40 µmol) was weighed on a piece of weighing paper and then transferred to a 20 mL vial. Next, 16.8 mL of Milli-Q water was added to the vial. The mixture was shaken and subsequently sonicated for 10 seconds to ensure complete dissolution, which resulted in the $MgCl_2$ stock solution **SR-04** in water (c = 2.5 mM).

Preparation of NaOAc stock solution SR-05 in water

Under an ambient atmosphere, a 20 mL borosilicate vial was charged with sodium acetate (NaOAc) (100 mg, 1.22 mmol). Next, 16.3 mL of Milli-Q water was added to the vial. The mixture was shaken and subsequently sonicated for 10 seconds to ensure complete dissolution, which resulted in the NaOAc stock solution **SR-05** in water (c = 75 mM).

Preparation of NaCl stock solution SR-06 in water

Under an ambient atmosphere, a 20 mL vial was charged with NaCl (5.00 g, 85.6 mmol). Next, 17.1 mL of Milli-Q water was added to the vial. The mixture was shaken and subsequently sonicated for 10 seconds to ensure complete dissolution, which resulted in the NaCl stock solution **SR-06** in water (c = 5.0 M).

Preparation of piperidine stock solution (20% v/v) SR-07 in DMF

Under an ambient atmosphere, piperidine (2.0 mL, 1.7 g, 20 mmol) was added to a 20 mL vial using a 5 mL syringe. Next, 8.0 mL of anhydrous DMF was added to the vial, which resulted in a total volume of 10 mL. Next, the solution was vortexed for 30 seconds, which resulted in the 20% piperidine (v/v) stock solution **SR-**

07 in DMF. The stock solution **SR-07** was then stored in a fridge at 4 °C.

Preparation of DIPEA stock solution SR-08 in DMA

Under an ambient atmosphere, a 1.5 mL Eppendorf tube was charged with diisopropylethylamine (DIPEA) (44 µL, 32 mg, 25 µmol). Next, 456 µL of DMA was added to the 1.5 mL Eppendorf tube and the mixture was vortexed for 10 seconds, which resulted in the DIPEA stock solution **SR-08** in DMA (c = 0.50 M).

Preparation of sodium borate buffer (pH 9.4, c = 0.50 M)

Under ambient atmosphere, boric acid (3.09 g, 50.0 mmol) was weighed onto weighing paper and transferred to a 250 mL plastic Kautex bottle equipped with a Teflon-coated magnetic stirring bar. Approximately 60 mL of Milli-Q water was added, and the mixture was stirred until the boric acid was completely dissolved. The pH of the solution was adjusted to 9.4 by the dropwise addition (approximately one drop every five seconds) of a sodium hydroxide stock solution in Milli-Q water (c = 1.0 M), under continuous stirring. The pH was continuously monitored using a calibrated pH meter. Once the pH reached 9.4, the addition of NaOH was stopped. The volume of the solution was then adjusted to 100 mL by the addition of Milli-Q water to the above solution, resulting in a sodium borate buffer stock solution (100 mL, pH 9.4, c = 0.50 M).

Functionalization of DNA conjugates

Preparation of DNA–AOP–NH_2 (2) stock solution SD-01 in water

The linker-elongated-headpiece DNA–AOP–NH_2 (**2**) was prepared using the procedure described below:

a. Structure of HP-NH_2

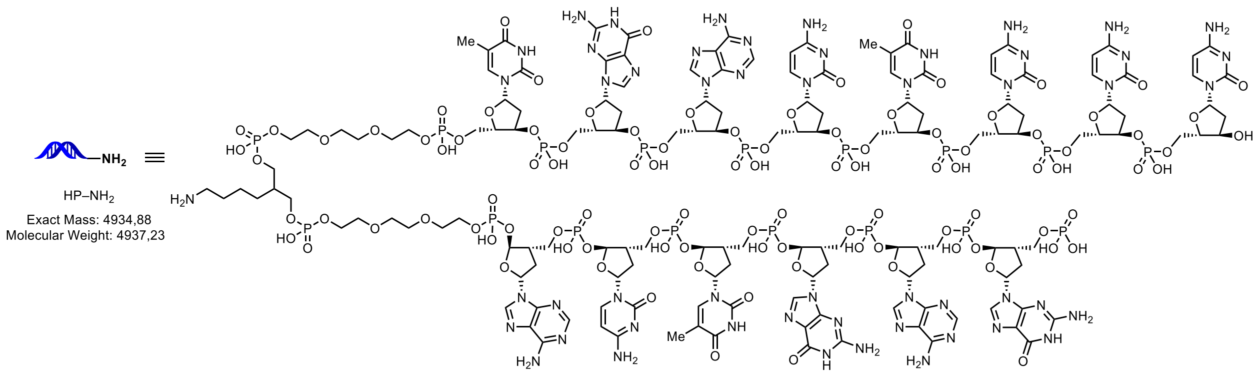

b. Preparation of DNA-AOP-NH_2 (**2**)

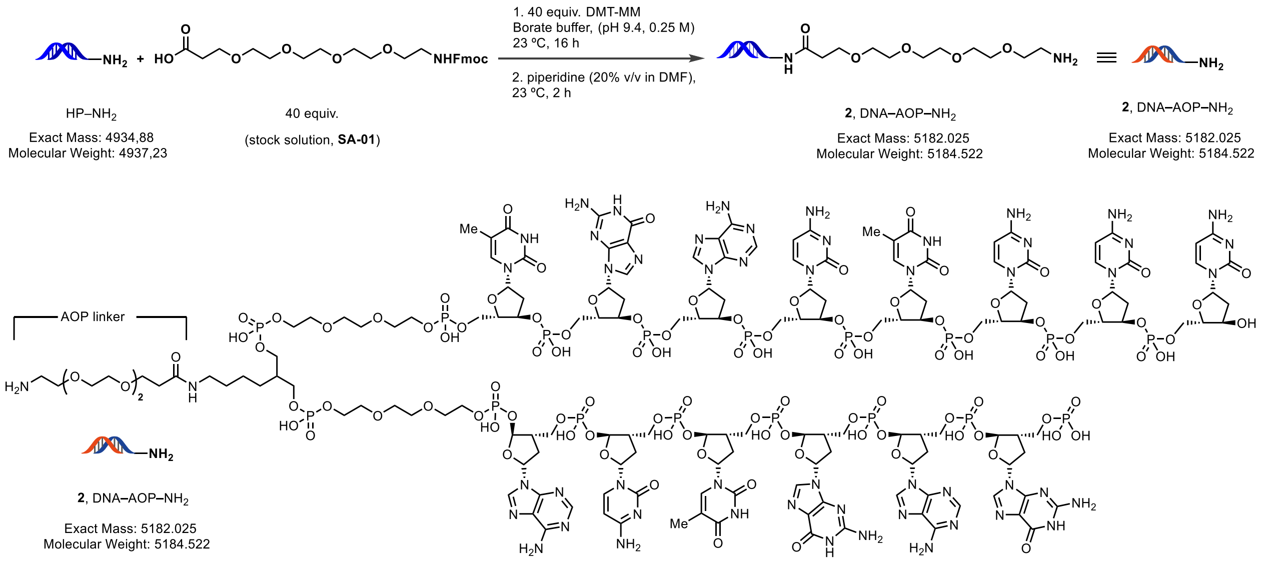

Preparation of Fmoc-AOP-COOH stock solution SA-01: Under an ambient atmosphere, a 1.5 mL Eppendorf tube was charged with Fmoc-15-amino-4, 7, 10, 13-tetraoxapentadecanoic acid (Fmoc-AOP-COOH) (195 mg, 0.400 mmol). Next, 1.0 mL of DMA was added to the above 1.5 mL Eppendorf tube and the mixture was vortexed for 10 seconds. Next, the Eppendorf tube was sonicated for 10 seconds to ensure that the Fmoc-AOP-COOH is completely dissolved, which resulted in the Fmoc-AOP-COOH stock solution **SA-01** (c = 0.40 M) in DMA.

Preparation of HP-NH_2 stock solution SB-01 in sodium borate buffer: Under an ambient atmosphere, the HP-NH_2 stock solution **SR-01** in water (2.0 mL, c = 5.0 mM) was added to a 15 mL Falcon tube and diluted with 3.0 mL of Milli-Q water, resulting in a stock solution of HP-NH_2 in water (5.0 mL, c = 2.0 mM). To the above Falcon tube was added, 5.0 mL of sodium borate buffer (pH 9.4, c = 0.50 M), resulting in a stock

solution **SB-01** of HP-NH_2 (10 mL, c = 1.0 mM) in sodium borate buffer (c = 0.25 M, pH 9.4).

Under an ambient atmosphere, the stock solution of HP-NH_2 (**SB-01**) (6.25 mL, c = 1.0 mM, 6.3 µmol, 1.0 equiv.) in sodium borate buffer (c = 0.25 M, pH 9.4) was added to a 15 mL Falcon tube. Next, the stock solution of Fmoc-15-amino-4, 7, 10, 13-tetraoxapentadecanoic acid (**SA-01**) (625 µL, c = 0.40 M, 250 µmol, 40 equiv.) in DMA was added. The reaction mixture was vortexed for 5 seconds. Then, the stock solution of DMT-MM (**SR-02**) (625 µL, c = 0.40 M, 250 µmol, 40 equiv.) in water was added. The mixture was vortexed for 5 seconds and then left standing at 23 °C for 16 hours. After 16 hours, an aliquot of 1 µL of the reaction mixture was diluted to 40 µL with water for LC–MS analysis. Next, the total reaction volume was roughly equally divided in four different portions of approximately 1.8 mL into four 15 mL Falcon tubes. Next, to each of the Falcon tubes were added the stock solution of NaCl in water (**SR-06**, 187 µL, c = 5.0 M, 10% volume of the total reaction volume), followed by cold ethanol (−20 °C, 6.2 mL) to precipitate the *N*-Fmoc amine DNA conjugate. The four Falcon tubes were placed in a freezer (−20 °C) for at least 1 hour, and each Falcon tube was centrifuged at 4 °C and 11000 × g for 45 minutes. Next, the supernatant was decanted, the pellet was dried under air, and then 781 µL of water were added in each of the Falcon tubes (the approximate concentration of the DNA-conjugate is c = ~2 mM). Next, to each Falcon tube containing the *N*-Fmoc amine DNA conjugate stock solution in water (781 µL, c = ~2 mM), was added the piperidine stock solution **SR-07** (781 µL, 20% v/v in DMF). The mixture was then left standing at 23 °C for 2 hours. After 2 hours, to each of the Falcon tubes was added the stock solution of NaCl in water (**SR-06**, 156 µL, c = 5.0 M, 10% volume of the total reaction volume), followed by cold ethanol (−20 °C, 5.2 mL) to precipitate the DNA conjugate. The four Falcon tubes were placed in a freezer (−20 °C) for at least 1 hour, and then each Falcon tube was centrifuged at 4 °C and 11000 × g for 1.5 hours. The supernatants were decanted, and the four pellets were dissolved (combined) in a total of 1.0 mL Milli-Q water. The resulting mixture was transferred to an Amicon ultra centrifugal filter (15 mL filter, 3 kDa MWCO). The mixture was then diluted to 12 mL with Milli-Q water. The solution was centrifuged at 11000 × g to a total volume of <500 µL. Next, 12 mL of Milli-Q water were added to the Amicon filter. The solution was centrifuged at 11000 × g to a total volume of <500 µL. The procedure for dilution with water and then centrifugation was repeated and additional time for a total of 3 three repetitions. The remaining DNA-conjugate **2** solution in the Amicon filter was then transferred to a 15 mL falcon tube. The Amicon filter was then washed with Milli-Q water (approximately 600 µL) and then transferred to the Falcon tube to make sure that the all the DNA conjugate **2** was collected from the filter. The concentration of the DNA conjugate **2** solution in water was measured by A_{260} absorption using a Thermo Scientific™ NanoDrop™ One^C. The concentration of the DNA-AOP-NH_2 (**2**) in water was then adjusted to c = 2.0 mM with Milli-Q water. The resulting stock solution **SD-01** (c = 2.0 mM in water) was used for performing all the on-DNA reactions.

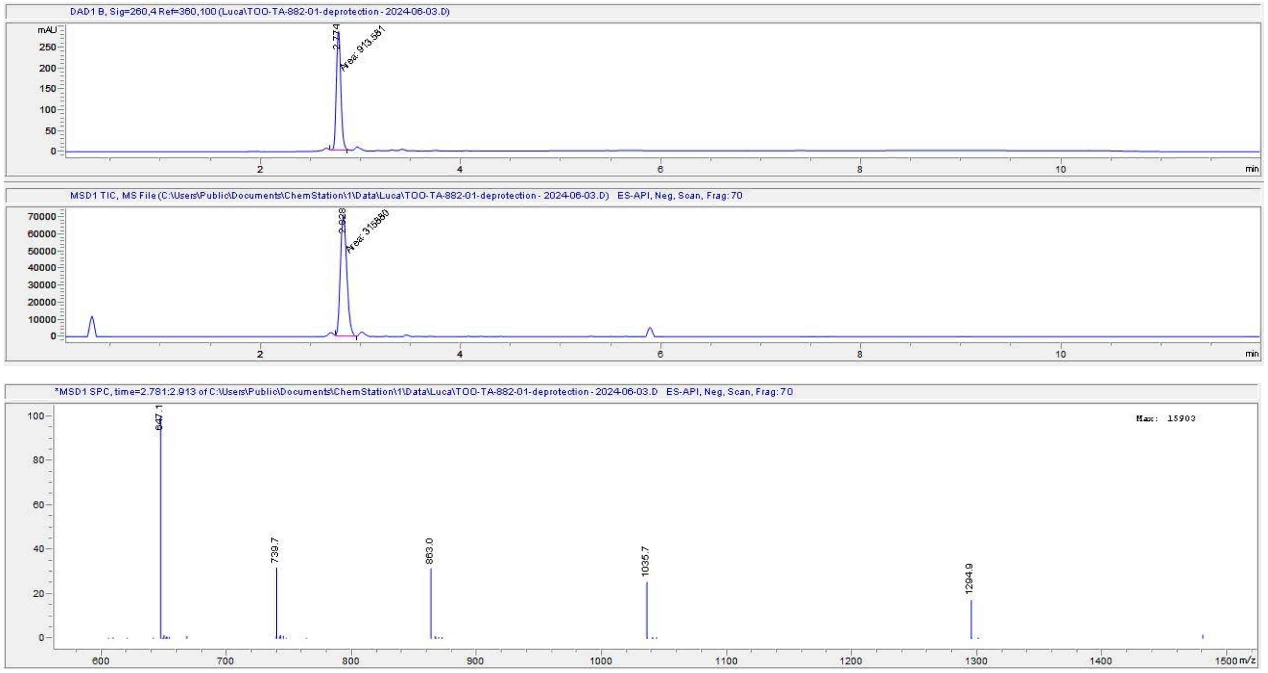

Figure S4. Analytical HPLC trace of DNA-AOP-NH_2 (**2**) with HPLC method A. (Top) DAD chromatogram at 260 nm. (Middle) TIC chromatogram. (Bottom) Ionization of peak at 2.83 min. containing reaction product **2**.

General procedures for the preparation of DNA-conjugated substrates

General procedure for synthesis of DNA-conjugated arenes with HATU

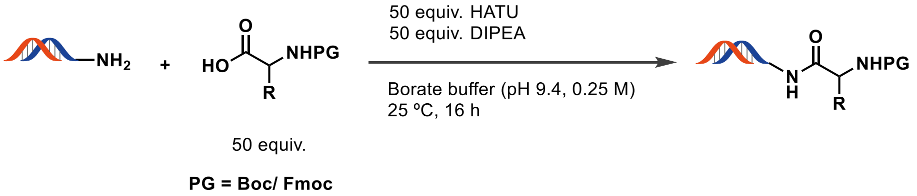

Under an ambient atmosphere, a stock solution of carboxylic acid in DMA (2.0 µL, c = 0.50 M, 1.0 µmol, 50 equiv.) was added to a 1.5 mL Eppendorf tube, followed by the stock solution of HATU in DMA (**SR-03**, 2.0 µL, c = 0.50 M, 1.0 µmol, 50 equiv.). The mixture was vortexed for 5 seconds. Next, a stock solution of DIPEA in DMA (**SR-08**, 2.0 µL, c = 0.50 M, 1.0 µmol, 50 equiv.) was added. The mixture was vortexed for 5 seconds and left standing at 23 °C for 20 min.

In a separate 1.5 mL Eppendorf tube was added the stock solution **SD-01** of DNA-AOP-NH_2 (**2**) in water (10 µL, c = 2.0 mM, 20 nmol, 1.0 equiv.), followed by sodium borate buffer (10 µL, pH 9.4, c = 0.50 M). The solution of the first Eppendorf tube containing acid, HATU and DIPEA (6.0 µL DMA) was then added to above DNA-conjugate **2** solution. The reaction mixture was vortexed for 5 seconds, transferred to a thermocycler at 25 °C, and incubated for 16 hours at 600 rpm. After 16 hours, an aliquot of 1 µL of the reaction mixture was diluted to 40 µL with water for LC–MS analysis. Next, to the above reaction mixture was added the stock solution of NaCl in water (**SR-06**, 2.6 µL, c = 5.0 M, 10% volume of the total reaction volume), followed by

cold ethanol (−20 °C, 86 µL) to precipitate the DNA conjugate. The Eppendorf tube was placed in a freezer (−20 °C) for at least 1 hour, and then it was centrifuged at 4 °C and 11000 × g for at least 30 minutes. The supernatant was removed and the pellet was dried under air, then dissolved in 10 µL water (c = 2.0 mM).

General procedure for synthesis of DNA-conjugated arenes with DMT-MM

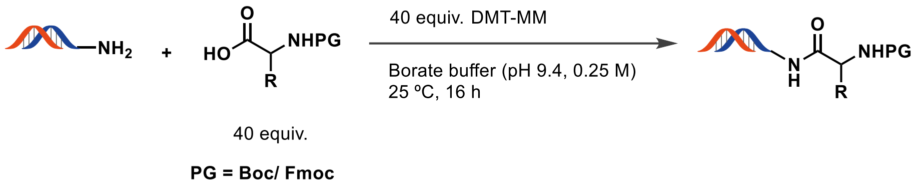

Under an ambient atmosphere, the stock solution **SD-01** of DNA-AOP-NH_2 (**2**) (10 µL, c = 2.0 mM, 20 nmol, 1.0 equiv.) in water was added to a 1.5 mL Eppendorf tube, followed by sodium borate buffer (10 µL, pH 9.4 c = 0.50 M). Next, a stock solution of the acid in DMA (2.0 µL, c = 0.40 M, 0.80 µmol, 40 equiv.) was added. The reaction mixture was vortexed for 5 seconds. Then, the stock solution of DMT-MM in water (**SR-02**, 2.0 µL, c = 0.40 M, 0.80 µmol, 40 equiv.) was added. The mixture was vortexed for 5 seconds, transferred to a thermocycler at 25 °C, and incubated for 16 hours at 600 rpm. After 16 hours, an aliquot of 1.0 µL of the reaction mixture was diluted to 40 µL with water for LC–MS analysis. Next, to the above reaction mixture was added the stock solution of NaCl in water (**SR-06**, 2.4 µL, c = 5.0 M, 10% volume of the total reaction volume), followed by cold ethanol (−20 °C, 80 µL) to precipitate the DNA conjugate. The Eppendorf tube was placed in a freezer (−20 °C) for at least 1 hour, and then it was centrifuged at 4 °C and 11000 × g for at least 30 minutes. The supernatant was removed and the pellet was dried under air, then dissolved in 10 µL water (c = 2.0 mM).

General procedure for synthesis of DNA-conjugated arenes by reverse amidation

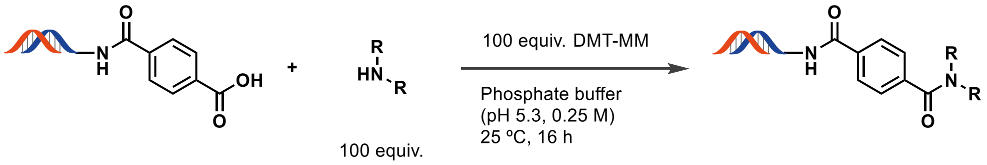

Under an ambient atmosphere, a stock solution of DNA-conjugated carboxylic acid (10 µL, c = 2.0 mM, 20 nmol, 1.0 equiv.) in water was added to a 1.5 mL Eppendorf tube, followed by NaPi buffer (10 µL, pH 5.3, c = 0.50 M). Next, a stock solution of the amine in DMA (2.0 µL, c = 1.0 M, 2.0 µmol, 100 equiv.) was added. The reaction mixture was vortexed for 5 seconds. Then, a stock solution of DMT-MM in water (2.0 µL, c = 1.0 M, 2.0 µmol, 100 equiv.) was added. The mixture was vortexed for 5 seconds, transferred to a thermocycler at 25 °C, and incubated for 16 hours at 600 rpm. After 16 hours, an aliquot of 1.0 µL of the reaction mixture was diluted to 40 µL with water for LC–MS analysis. Next, to the above reaction mixture was added the stock solution of NaCl in water (**SR-06**, 2.4 µL, c = 5.0 M, 10% volume of the total reaction volume), followed by cold ethanol (−20 °C, 80 µL) to precipitate the DNA conjugate. The Eppendorf tube was placed in a freezer (−20 °C) for at least 1 hour, and then it was centrifuged at 4 °C and 11000 × g for at least 30 minutes. The

supernatant was removed and the pellet was dried under air, then dissolved in 10 µL water (c = 2.0 mM).

General procedure for on-DNA *N*-Fmoc deprotection

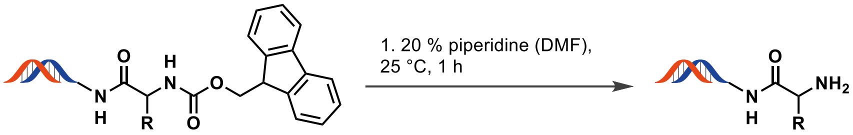

The piperidine stock solution **SR-07** (20 µL, 20% v/v, in DMF) was added to a 1.5 mL Eppendorf tube containing the *N*-Fmoc protected DNA conjugate (20 µL, c = 1.0 mM in water) and the resulting solution was vortexed for 5 seconds. The mixture was allowed to stand at 25 °C for 2 hours. Afterwards, an aliquot of 1 µL of the reaction mixture was diluted to 40 µL with water for LC–MS analysis. Next, to the above reaction mixture was added the stock solution of NaCl in water (**SR-06**, 4.0 µL, c = 5.0 M, 10% volume of the total reaction volume), followed by cold ethanol (−20 °C, 120 µL) to precipitate the *N*-Fmoc deprotected DNA-conjugate. The Eppendorf tube was placed in a freezer (−20 °C) for at least 1 hour and then it was centrifuged at 4 °C and 11000 × g for at least 30 minutes. The supernatant was decanted and the pellet was dried under air, then dissolved in 10 µL of water to obtain the *N*-Fmoc deprotected DNA-conjugate (10 µL, c = 2.0 mM).

General procedure for on-DNA *N*-Boc deprotection

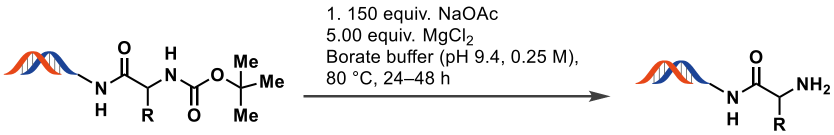

To a 1.5 mL Eppendorf tube containing the *N*-Boc protected DNA-conjugate (20 µL, c = 1.0 mM) in water, was added sodium borate buffer (100 µL, pH 9.4, c = 0.50 M), followed by stock solutions of NaOAc in water (**SR-05**, 40 µL, c = 75 mM) and $MgCl_2$ in water (**SR-04**, 40 µL, c = 2.5 mM). This resulted in the final reaction volume of 200 µL. The reaction mixture was then incubated in a thermocycler at 80 °C for 16–24 hours at 800 rpm. The progress of the reaction was monitored by LC–MS analysis. After the deprotection (approximately 24–48h), the reaction mixture was cooled to 23 °C and the mixture was then filtered using an Amicon ultra centrifugal filter (3 kDa MWCO). The retained DNA conjugate was dissolved in 8–10 µL of water to afford the *N*-Boc deprotected DNA-conjugate. The DNA concentration was determined by measuring the A_{260} absorption using a Thermo Scientific™ NanoDrop™ One^{C}). The concentration was then adjusted by addition of Milli Q water to c = 2.0 mM.

General procedure for *N*-arylation of amine-DNA conjugates with aryl fluorides

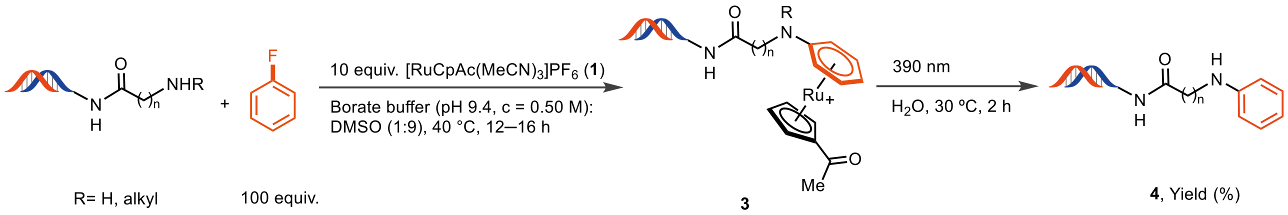

Preparation of η[6] ruthenium complex: Under an ambient atmosphere, a 1 mL glass GC vial equipped with a 6 mm Teflon-coated stirring bar was charged with ruthenium complex **1** (1.4 mg, 2.9 µmol, 1.0 equiv.). Next, a stock solution of fluorobenzene in DMC (294 µL, c = 0.10 M, 29 µmol, 10 equiv.) was added. The vial was then closed with a screw cap and reaction mixture was heated at 80 °C for 2 hours. After 2 hours, the reaction mixture was cooled to 23 °C. Next, the DMC was removed under a gentle stream of argon and 294 µL of DMSO were added to result an in situ formed stock solution of arene-ruthenium complex **C1** (294 µL, c = 0.01 M, assuming quantitative arene coordination to ruthenium). The stock solution **C1** was used for performing *N*-arylation of all the amine DNA-conjugates.

On-DNA S_NAr reaction: At 20–25 °C, a stock solution of amine DNA-conjugate (1.0 µL, c = 2.0 mM, 2.0 nmol, 1.0 equiv.) in water was added to a 1.5 mL Eppendorf tube, followed by sodium borate buffer (1.0 µL, c = 0.50 M, pH 9.4). To this mixture, 16 µL of DMSO was added and the solution was vortexed for 5 seconds. Next, the freshly prepared stock solution **C1** (2.0 µL, c = 0.01 M, 0.02 µmol, 10 equiv.) in DMSO was added. The resulting reaction mixture was vortexed for 5 seconds, transferred to a thermocycler at 40 °C, and incubated for 16 hours at 800 rpm to yield the DNA-conjugate **3**. The reaction mixture was diluted with 10 µL of Milli-Q water. To the reaction mixture was added the stock solution of NaCl in water (**SR-06** , 3.0 µL, c = 5.0 M, 10% volume of the total reaction volume), followed by cold ethanol (−20 °C, 99 µL) to precipitate the *N*-arylated ruthenium DNA conjugate **3**. The Eppendorf tube was placed in a freezer (−20 °C) for at least 1 hour, and then it was centrifuged at 4 °C and 11000 × g for at least 30 minutes. The supernatant was removed and the pellet was dried under air, then dissolved in 20 µL water to obtain the DNA-conjugate **3** (20 µL, c = 0.10 mM). Then, 1.0 µL of the above solution was diluted to 40 µL with water for LC–MS analysis.

Decomplexation of the DNA-conjugated ruthenium η[6] complex: Under an ambient atmosphere, the DNA-conjugate **3** stock solution in water (c = 0.10 mM, 20 µL) was irradiated with a 390 nm (40 W) Kessil lamp for 2 hours, while maintaining the temperature at approximately 30 °C through cooling with a fan. To the reaction mixture was added the stock solution of NaCl in water (**SR-06**, 2.0 µL, c = 5.0 M, 10% volume of the total reaction volume), followed by cold ethanol (−20 °C, 66 µL) to precipitate the DNA conjugate **4**. The Eppendorf tube was placed in the freezer (−20 °C) for at least 1 hour, and then it was centrifuged at 4 °C and 11000 × g for at least 30 minutes. The supernatant was removed, the pellet dried under air and dissolved in Milli-Q water to obtain the purified DNA-conjugate **4**. Then, 1 µL of the above solution was diluted to 40 µL with water for LC–MS analysis.

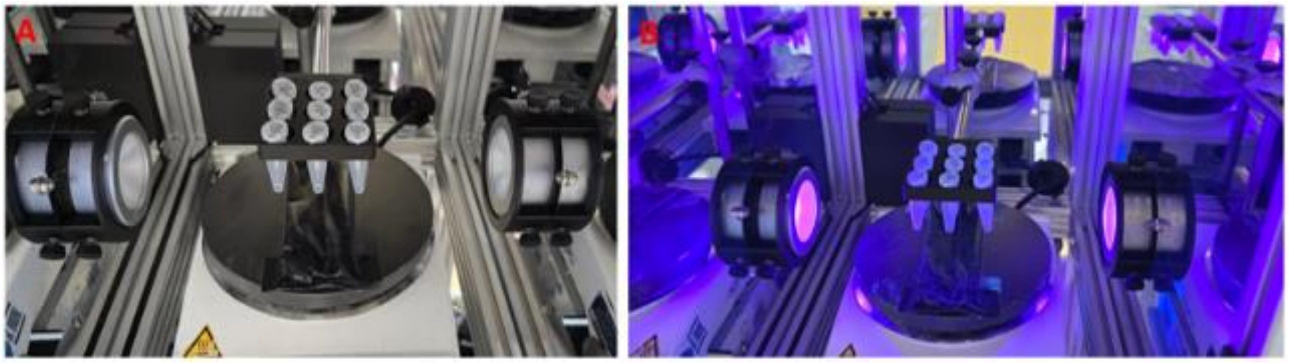

Figure S5. Pictures of the decomplexation reaction set up. A = 1.5 mL Eppendorf tubes placed vertically in the vial holder, perpendicular to the direction of light. B = Decomplexation by the irradiation of 390 nm Kessil lamp (40 W).

Preparation of DNA-conjugated substrates

DNA-conjugated substrate S15

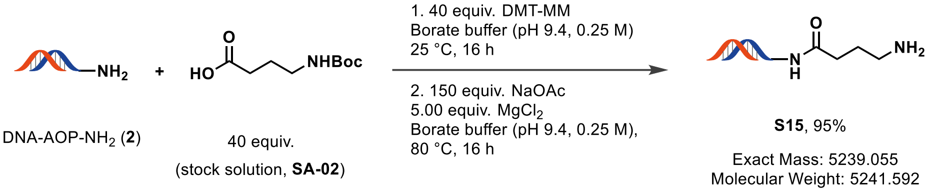

Preparation of γ-(Boc-amino) butyric acid stock solution (SA-02): At 20–25 °C, a 1.5 mL Eppendorf tube was charged with 4-γ-(Boc-amino) butyric acid (8.1 mg, 40 µmol). Next, 100 µL of DMA was added to the above 1.5 mL Eppendorf tube and the mixture was vortexed for 10 seconds. Next, the Eppendorf vial was sonicated for 10 seconds to make sure that the *N*-Boc acid is completely soluble. This resulted in the formation of the 4-γ-(Boc-amino) butyric acid stock solution in DMA (**SA-02**, c = 0.40 M). This stock solution was used for performing on-DNA reactions.

At 20–25 °C, the stock solution **SD-01** of DNA-AOP-NH_2 (**2**) (10 µL, c = 2.0 mM, 20 nmol, 1.0 equiv.) in water was added to a 1.5 mL Eppendorf tube, followed by sodium borate buffer (10 µL, pH 9.4, c = 0.50 M). Next, the stock solution of 4-γ-(Boc-amino) butyric acid stock solution (**SA-02**, 2.0 µL, c = 0.40 M, 0.80 µmol, 40 equiv.) in DMA was added. The reaction mixture was vortexed for 5 seconds. Then, the stock solution of DMT-MM (**SR-02**, 2.0 µL, c = 0.40 M, 0.80 µmol, 40 equiv.) in water was added. The mixture was vortexed for 5 seconds, transferred to a thermocycler at 25 °C, and incubated for 16 hours at 600 rpm. After 16 hours, an aliquot of 1.0 µL of the reaction mixture was diluted to 40 µL with water for LC–MS analysis. Next, to the above reaction mixture was added the stock solution of NaCl in water (**SR-06**, 2.4 µL, c = 5.0 M, 10% volume of the total reaction volume), followed by cold ethanol (−20 °C, 80 µL) to precipitate the *N*-Boc amine DNA conjugate. The Eppendorf tube was placed in a freezer (−20 °C) for at least 1 hour, and then it was centrifuged at 4 °C and 11000 × g for at least 30 minutes. The supernatant was removed and the pellet was dried under air, then dissolved in 20 µL water (c = 1.0 mM).

Boc deprotection: To the above 1.5 mL Eppendorf tube containing the *N*-Boc amine DNA-conjugate (20 µL, c = 1.0 mM) in water, was added sodium borate buffer (100 µL, pH 9.4, c = 0.50 M), followed by stock solutions of NaOAc in water (**SR-05**, 40 µL, c = 75 mM) and $MgCl_2$ in water (**SR-04**, 40 µL, c = 2.5 mM). This resulted in the final reaction volume of 200 µL. The reaction mixture was then incubated in a thermocycler at 80 °C for 16–24 hours at 800 rpm. The progress of the reaction was monitored by LC–MS analysis. After the deprotection (approximately 24–48h), the reaction mixture was cooled to 23 °C and the mixture was then filtered using an Amicon ultra centrifugal filter (3 kDa MWCO). The retained DNA conjugate was dissolved in 8–10 µL of water to afford DNA conjugate **S15**. The DNA concentration was determined by measuring the A_{260} absorption using a Thermo Scientific™ NanoDrop™ One^{C}). The concentration was then adjusted by addition of Milli Q water to c = 2.0 mM. The yield of the DNA conjugate was calculated by measuring the integration of the peaks of the diode array detection (DAD) UV absorbance at 260 nm of the LC–MS trace, assuming complete DNA recovery and identical UV absorbance.

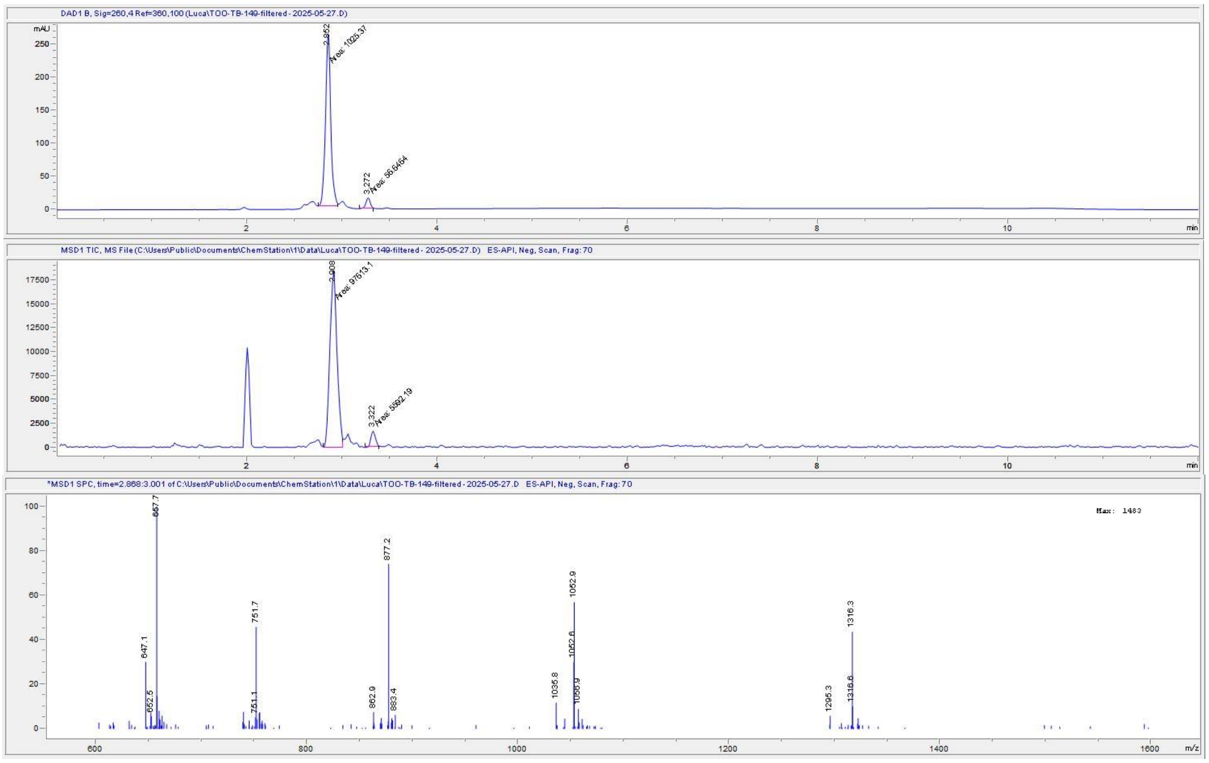

Figure S6. Analytical HPLC trace of **S15** with HPLC method A. (Top) DAD chromatogram at 260 nm. (Middle) TIC chromatogram. (Bottom) Ionization of peak at 2.91 min. containing reaction product.

DNA-conjugated substrate S16

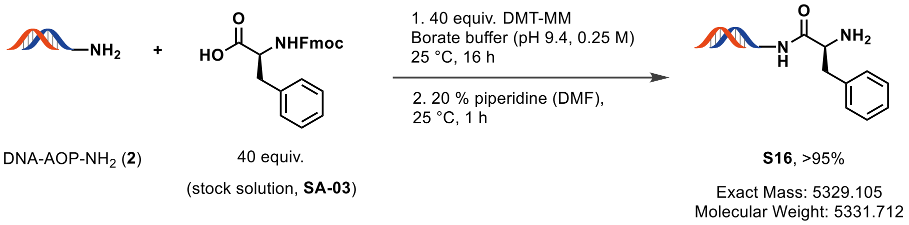

Preparation of *N*-Fmoc-*L*-phenylalanine stock solution (SA-03): Under an ambient atmosphere, a 1.5 mL Eppendorf tube was charged with (((9H-fluoren-9-yl)methoxy)carbonyl)-*L*-phenylalanine (31 mg, 80 µmol). Next, 200 µL of DMA was added to the above 1.5 mL Eppendorf tube and the mixture was vortexed for 10 seconds. Next, the Eppendorf tube was sonicated for 10 seconds to ensure that the *N*-Fmoc amino acid is completely dissolved resulting in the formation of (((9H-fluoren-9-yl)methoxy)carbonyl)-*L*-phenylalanine stock solution in DMA (**SA-03**, c = 0.40 M).

Under an ambient atmosphere, the stock solution **SD-01** of DNA-AOP-NH_2 (**2**) in water (10 µL, c = 2.0 mM, 20 nmol, 1.0 equiv.) was added to a 1.5 mL Eppendorf tube, followed by sodium borate buffer (10 µL, pH 9.4, c = 0.50 M). Next, the stock solution of (((9H-fluoren-9-yl)methoxy)carbonyl)-*L*-phenylalanine in DMA (**SA-03**, 2.0 µL, c = 0.40 M, 0.80 µmol, 40 equiv.) was added. The reaction mixture was vortexed for 5 seconds. Then, the stock solution of DMT-MM in water (**SR-03**, 2.0 µL, c = 0.40 M, 0.80 µmol, 40 equiv.) was added. The mixture was vortexed for 5 seconds, transferred to a thermocycler at 25 °C, and incubated for 16 hours at 600 rpm. After 16 hours, an aliquot of 1.0 µL of the reaction mixture was diluted to 40 µL with water for LC–MS analysis. Next, to the above reaction mixture was added the stock solution of NaCl in water (**SR-06**, 2.4 µL, c = 5.0 M, 10% volume of the total reaction volume), followed by cold ethanol (−20 °C, 80 µL) to precipitate the *N*-Fmoc phenylalanine DNA conjugate. The Eppendorf tube was placed in a freezer (−20 °C) for at least 1 hour, and then it was centrifuged at 4 °C and 11000 × g for at least 30 minutes. The supernatant was removed and the pellet was dried under air, then dissolved in 20 µL water (c = 1.0 mM).

Fmoc deprotection: The piperidine stock solution **SR-07** (20 µL, 20% v/v, in DMF) was added to the above 1.5 mL Eppendorf tube containing the *N*-Fmoc phenylalanine DNA conjugate (20 µL, c = 1.0 mM in water) and the resulting solution was vortexed for 5 seconds. The mixture was allowed to stand at 25 °C for 2 hours. Afterwards, an aliquot of 1 µL of the reaction mixture was diluted to 40 µL with water for LC–MS analysis. Next, to the above reaction mixture was added the stock solution of NaCl in water (**SR-06**, 4.0 µL, c = 5.0 M, 10% volume of the total reaction volume), followed by cold ethanol (−20 °C, 120 µL) to precipitate the DNA conjugate **S16**. The Eppendorf tube was placed in a freezer (−20 °C) for at least 1 hour and then it was centrifuged at 4 °C and 11000 × g for at least 30 minutes. The supernatant was decanted and the pellet was dried under air, then dissolved in 10 µL of water to obtain the DNA-phenylalanine conjugate **S16** (10 µL, c = 2.0 mM). The yield of the DNA conjugate was calculated by measuring the integration of the peaks of the diode array detection (DAD) UV absorbance at 260 nm of the LC–MS trace, assuming complete DNA recovery and identical UV absorbance.

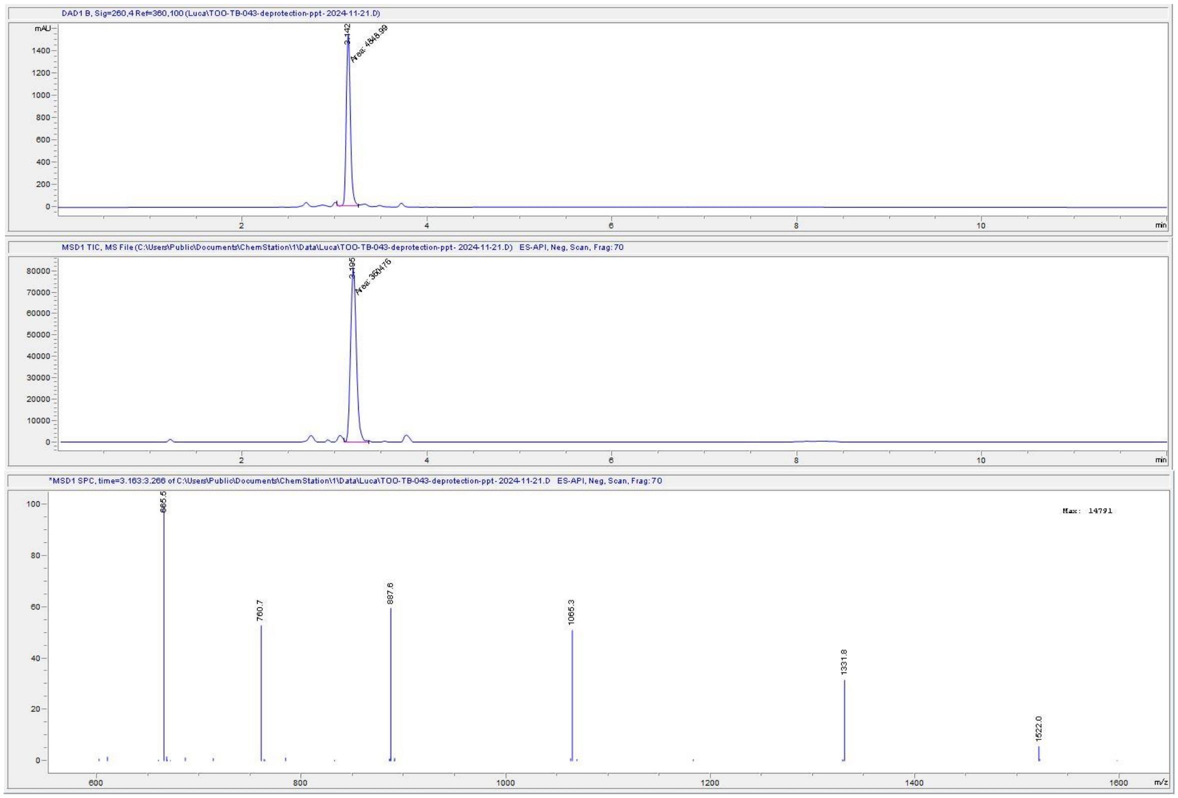

Figure S7. Analytical HPLC trace of **S16** with HPLC method A. (Top) DAD chromatogram at 260 nm. (Middle) TIC chromatogram. (Bottom) Ionization of peak at 3.19 min. containing reaction product.

DNA-conjugated substrate S17

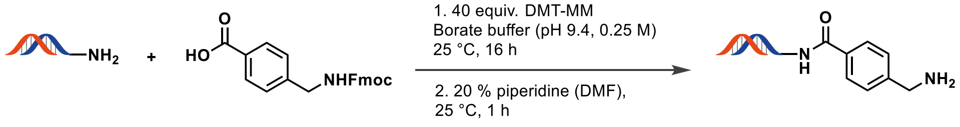

DNA-AOP-NH_2 (**2**) 40 equiv. (stock solution**, SA-04**) **S17**, >95%

Exact Mass: 5315.085
Molecular Weight: 5317.692

Preparation of 4-(Fmoc-aminomethyl)benzoic acid stock solution (SA-04): Under an ambient atmosphere, a 1.5 mL Eppendorf tube was charged with 4-(Fmoc-aminomethyl)benzoic acid (14.9 mg, 39.9 µmol). Next, 100 µL of DMA was added to the above 1.5 mL Eppendorf tube and the mixture was vortexed for 10 seconds. Next, the Eppendorf tube was sonicated for 10 seconds to ensure that the *N*-Fmoc amino acid is completely dissolved, resulting in the formation of 4-(Fmoc-aminomethyl)benzoic acid stock solution **SA-04** in DMA (c = 0.40 M).

Under an ambient atmosphere, the stock solution **SD-01** of DNA-AOP-NH_2 (**2**) in water (10 µL, c = 2.0 mM, 20 nmol, 1.0 equiv.) was added to a 1.5 mL Eppendorf tube, followed by sodium borate buffer (10 µL, pH 9.4, c = 0.50 M). Next, the stock solution of 4-(Fmoc-aminomethyl)benzoic acid in DMA (**SA-04**, 2.0 µL , c = 0.40 M, 0.80 µmol, 40 equiv.) was added. The reaction mixture was vortexed for 5 seconds. Then, the stock solution of DMT-MM in water (**SR-02**, 2.0 µL, c = 0.40 M, 0.80 µmol, 40 equiv.) was added. The mixture was

vortexed for 5 seconds, transferred to a thermocycler at 25 °C, and incubated for 16 hours at 600 rpm. After 16 hours, an aliquot of 1.0 µL of the reaction mixture was diluted to 40 µL with water for LC–MS analysis. Next, to the above reaction mixture was added the stock solution of NaCl in water (**SR-06**, 2.4 µL, c = 5.0 M, 10% volume of the total reaction volume), followed by cold ethanol (−20 °C, 80 µL) to precipitate the *N*-Fmoc amine DNA conjugate. The Eppendorf tube was placed in a freezer (−20 °C) for at least 1 hour, and then it was centrifuged at 4 °C and 11000 × g for at least 30 minutes. The supernatant was removed and the pellet was dried under air, then dissolved in 20 µL water (c = 1.0 mM).

Fmoc deprotection: The piperidine stock solution **SR-07** (20 µL, 20% v/v, in DMF) was added to the above 1.5 mL Eppendorf tube containing the DNA conjugate (20 µL, c = 1.0 mM in water) and the resulting solution was vortexed for 5 seconds. The mixture was allowed to stand at 25 °C for 2 hours. Afterwards, an aliquot of 1 µL of the reaction mixture was diluted to 40 µL with water for LC–MS analysis. Next, to the above reaction mixture was added the stock solution of NaCl in water (**SR-06**, 4.0 µL, c = 5.0 M, 10% volume of the total reaction volume), followed by cold ethanol (−20 °C, 120 µL) to precipitate the DNA conjugate **S17**. The Eppendorf tube was placed in a freezer (−20 °C) for at least 1 hour and then it was centrifuged at 4 °C and 11000 × g for at least 30 minutes. The supernatant was decanted and the pellet was dried under air, then dissolved in 10 µL of water to obtain the DNA-conjugate **S17** (10 µL, c = 2.0 mM). The yield of the DNA conjugate was calculated by measuring the integration of the peaks of the diode array detection (DAD) UV absorbance at 260 nm of the LC–MS trace, assuming complete DNA recovery and identical UV absorbance.

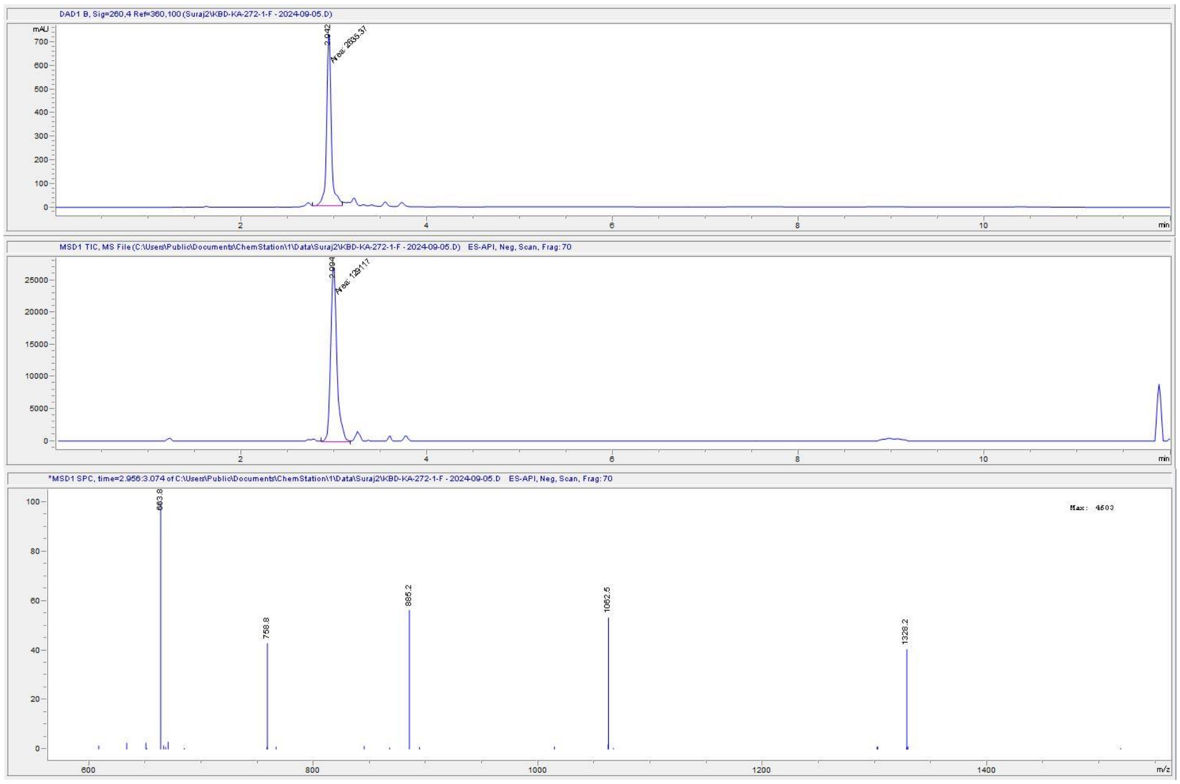

Figure S8. Analytical HPLC trace of **S17** with HPLC method A. (Top) DAD chromatogram at 260 nm. (Middle) TIC chromatogram. (Bottom) Ionization of peak at 2.99 min. containing reaction product.

DNA-conjugated substrate S18

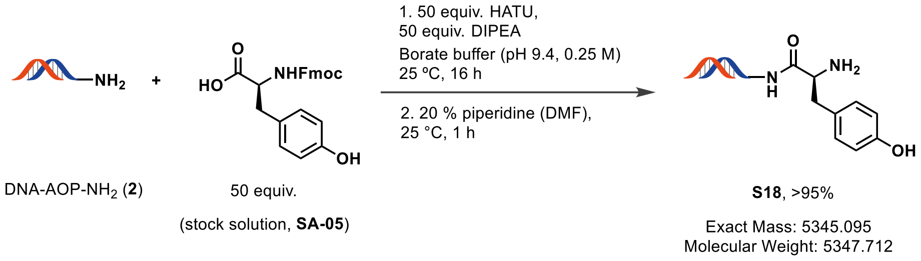

Preparation of *N*-Fmoc-*L*-tyrosine stock solution (SA-05): At 20–25 °C, a 1.5 mL Eppendorf tube was charged with *N*-Fmoc-*L*-tyrosine (20.1 mg, 49.8 µmol). Next, 100 µL of DMA was added to the above 1.5 mL Eppendorf tube and the mixture was vortexed for 10 seconds. Next, the Eppendorf vial was sonicated for 10 seconds to ensure that the *N*-Fmoc acid is completely dissolved, resulting in the formation of *N*-Fmoc-*L*-tyrosine stock solution in DMA (**SA-05**, c = 0.50 M).

Under an ambient atmosphere, the stock solution of *N*-Fmoc-*L*-tyrosine in DMA (**SA-05**, 2.0 µL, c = 0.50 M, 1.0 µmol, 50 equiv.) was added to a 1.5 mL Eppendorf tube, followed by the stock solution of HATU in DMA (**SR-03**, 2.0 µL, c = 0.50 M, 1.0 µmol, 50 equiv.). The mixture was vortexed for 5 seconds. Next, the stock solution of DIPEA in DMA (**SR-08**, 2.0 µL, c = 0.50 M, 1.0 µmol, 50 equiv.) was added. The mixture was vortexed for 5 seconds and left standing at 23 °C for 20 min.

In a separate 1.5 mL Eppendorf tube was added the stock solution **SD-01** of DNA-AOP-NH_2 (**2**) in water (10 µL, c = 2.0 mM, 20 nmol, 1.0 equiv.), followed by sodium borate buffer (10 µL, pH 9.4, c = 0.50 M). The solution of the first Eppendorf tube containing acid, HATU and DIPEA (6.0 µL DMA) was then added to above DNA-conjugate **2** solution. The reaction mixture was vortexed for 5 seconds, transferred to a thermocycler at 25 °C, and incubated for 16 hours at 600 rpm. After 16 hours, an aliquot of 1 µL of the reaction mixture was diluted to 40 µL with water for LC–MS analysis. Next, to the above reaction mixture was added the stock solution of NaCl in water (**SR-06**, 2.6 µL, c = 5.0 M, 10% volume of the total reaction volume), followed by cold ethanol (−20 °C, 86 µL) to precipitate the DNA conjugate. The Eppendorf tube was placed in a freezer (−20 °C) for at least 1 hour, and then it was centrifuged at 4 °C and 11000 × g for at least 30 minutes. The supernatant was removed and the pellet was dried under air, then dissolved in 20 µL water (c = 1.0 mM).

Fmoc deprotection: Fmoc deprotection: The piperidine stock solution **SR-07** (20 µL, 20% v/v, in DMF) was added to the above 1.5 mL Eppendorf tube containing the *N*-Fmoc tyrosine DNA conjugate (20 µL, c = 1.0 mM in water) and the resulting solution was vortexed for 5 seconds. The mixture was allowed to stand at 25 °C for 2 hours. Afterwards, an aliquot of 1 µL of the reaction mixture was diluted to 40 µL with water for LC–MS analysis. Next, to the above reaction mixture was added the stock solution of NaCl in water (**SR-06**, 4.0 µL, c = 5.0 M, 10% volume of the total reaction volume), followed by cold ethanol (−20 °C, 120 µL) to precipitate the DNA conjugate **S18**. The Eppendorf tube was placed in a freezer (−20 °C) for at least 1 hour and then it was centrifuged at 4 °C and 11000 × g for at least 30 minutes. The supernatant was decanted and the pellet was dried under air, then dissolved in 10 µL of water to obtain the DNA-conjugate **S18** (10 µL, c = 2.0 mM). The yield of the DNA conjugate was calculated by measuring the integration of the peaks of the

diode array detection (DAD) UV absorbance at 260 nm of the LC–MS trace, assuming complete DNA recovery and identical UV absorbance.

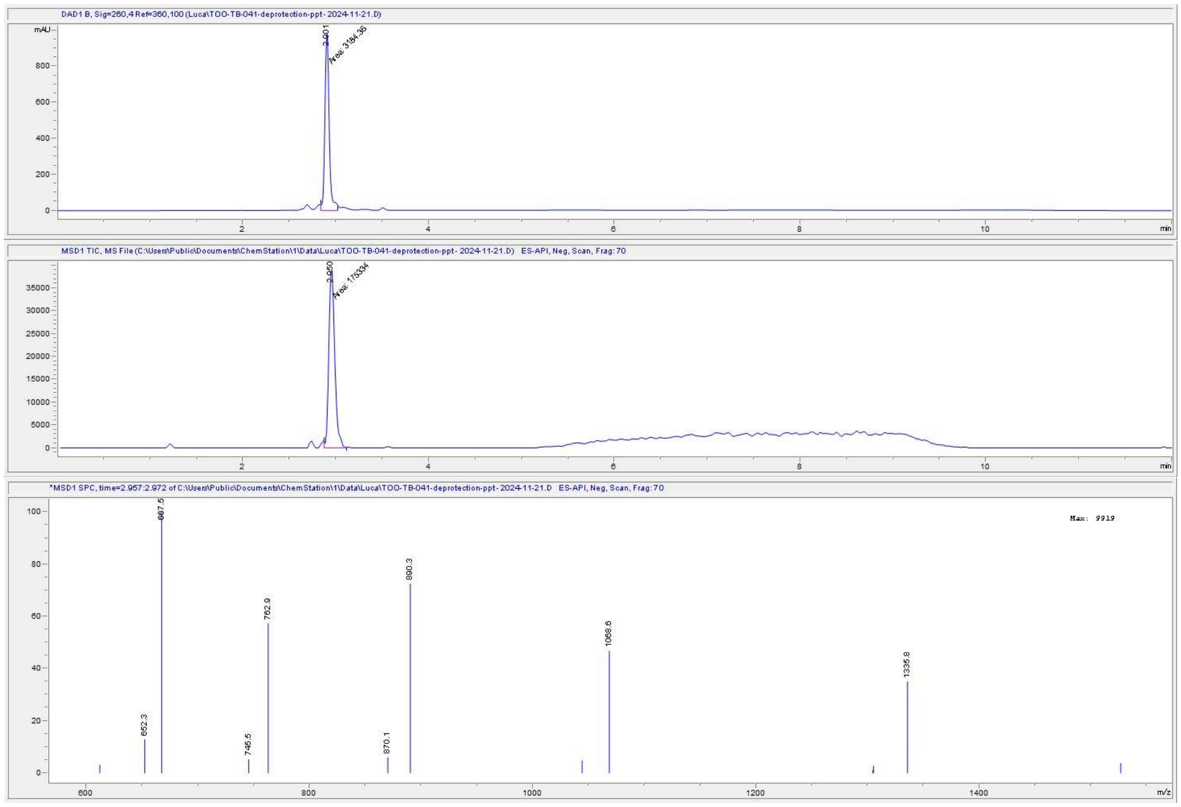

Figure S9. Analytical HPLC trace of **S18** with HPLC method A. (Top) DAD chromatogram at 260 nm. (Middle) TIC chromatogram. (Bottom) Ionization of peak at 2.95 min. containing reaction product.

DNA-conjugated substrate S19

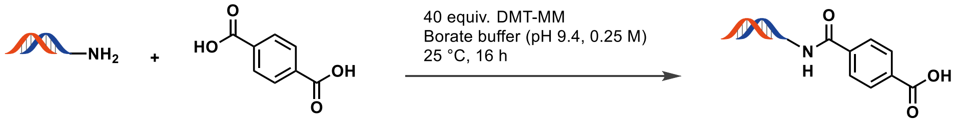

DNA-AOP-NH$_2$ (**2**)   40 equiv. (stock solution, **SA-06**)   **S19**, 88%

Exact Mass: 5330.041
Molecular Weight: 5332.639

Preparation of terephthalic acid stock solution (SA-06): Under an ambient atmosphere, a 1.5 mL Eppendorf tube was charged with terephthalic acid (13.3 mg, 80.0 µmol). Next, 200 µL of DMA was added to the above 1.5 mL Eppendorf tube and the mixture was vortexed for 10 seconds. Next, the Eppendorf tube was sonicated for 10 seconds to ensure that the terephthalic acid is completely dissolved, resulting in the formation of terephthalic acid stock solution **SA-06** in DMA (c = 0.40 M).

At 20–25 °C, the stock solution **SD-01** of DNA-AOP-NH$_2$ (**2**) (10 µL, c = 2.0 mM, 20 nmol, 1.0 equiv.) in water was added to a 1.5 mL Eppendorf tube, followed by sodium borate buffer (10 µL, pH 9.4, c = 0.50 M). Next, the stock solution of terephthalic acid (**SA-06**, 2.0 µL, c = 0.40 M, 0.80 µmol, 40 equiv.) in DMA was added.

The reaction mixture was vortexed for 5 seconds. Then, the stock solution of DMT-MM (**SR-02**, 2.0 µL, c = 0.40 M, 0.80 µmol, 40 equiv.) in water was added. The mixture was vortexed for 5 seconds, transferred to a thermocycler at 25 °C, and incubated for 16 hours at 600 rpm. After 16 hours, an aliquot of 1.0 µL of the reaction mixture was diluted to 40 µL with water for LC–MS analysis. Next, to the above reaction mixture was added the stock solution of NaCl in water (**SR-06**, 2.4 µL, c = 5.0 M, 10% volume of the total reaction volume), followed by cold ethanol (−20 °C, 80 µL) to precipitate the DNA conjugate. The Eppendorf tube was placed in a freezer (−20 °C) for at least 1 hour, and then it was centrifuged at 4 °C and 11000 × g for at least 30 minutes. The supernatant was removed and the pellet was dried under air, then dissolved in 10 µL water (c = 2.0 mM). The yield of the DNA conjugate was calculated by measuring the integration of the peaks of the diode array detection (DAD) UV absorbance at 260 nm of the LC–MS trace, assuming complete DNA recovery and identical UV absorbance.

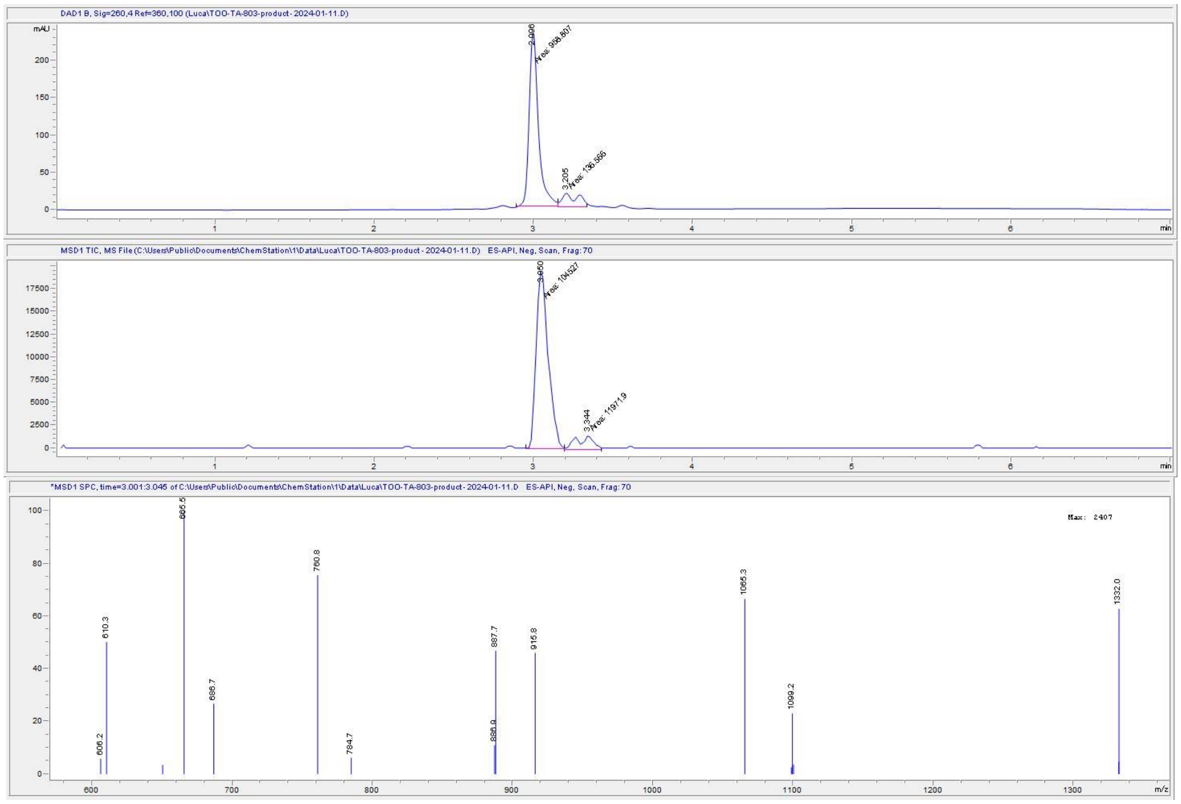

Figure S10. Analytical HPLC trace of **S19** with HPLC method B. (Top) DAD chromatogram at 260 nm. (Middle) TIC chromatogram. (Bottom) Ionization of peak at 3.05 min. containing reaction product.

DNA-conjugated substrate S20

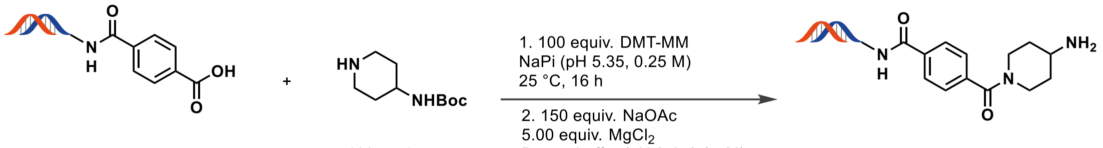

Preparation of *tert*-butyl piperidin-4-ylcarbamate stock solution (SA-07): At 20–25 °C, a 1.5 mL Eppendorf tube was charged with *tert*-butyl piperidin-4-ylcarbamate (20 mg, 0.10 mmol). Next, 100 µL of DMA was added to the above 1.5 mL Eppendorf tube and the mixture was vortexed for 10 seconds. Next, the Eppendorf vial was sonicated for 10 seconds to make sure that the *N*-Boc protected amine is completely dissolved, resulting in the formation of *tert*-butyl piperidin-4-ylcarbamate stock solution in DMA (**SA-07**, c = 1.0 M).

Under an ambient atmosphere, the stock solution of DNA-conjugated carboxylic acid **S19** (10 µL, c = 2.0 mM, 20 nmol, 1.0 equiv.) in water was added to a 1.5 mL Eppendorf tube, followed by NaPi buffer (10 µL, pH 5.3, c = 0.50 M). Next, the stock solution of the amine in DMA (**SA-07**, 2.0 µL, c = 1.0 M, 2.0 µmol, 100 equiv.) was added. The reaction mixture was vortexed for 5 seconds. Then, the stock solution of DMT-MM in water (**SR-02**, 2.0 µL, c = 1.0 M, 2.0 µmol, 100 equiv.) was added. The mixture was vortexed for 5 seconds, transferred to a thermocycler at 25 °C, and incubated for 16 hours at 600 rpm. After 16 hours, an aliquot of 1.0 µL of the reaction mixture was diluted to 40 µL with water for LC–MS analysis. Next, to the above reaction mixture was added the stock solution of NaCl in water (**SR-06**, 2.4 µL, c = 5.0 M, 10% volume of the total reaction volume), followed by cold ethanol (−20 °C, 80 µL) to precipitate the DNA conjugate. The Eppendorf tube was placed in a freezer (−20 °C) for at least 1 hour, and then it was centrifuged at 4 °C and 11000 × g for at least 30 minutes. The supernatant was removed and the pellet was dried under air, then dissolved in 20 µL water (c = 1.0 mM).

Boc deprotection: To the above 1.5 mL Eppendorf tube containing the *N*-Boc amine DNA-conjugate (20 µL, c = 1.0 mM) in water, was added sodium borate buffer (100 µL, pH 9.4, c = 0.50 M), followed by stock solutions of NaOAc in water (**SR-05**, 40 µL, c = 75 mM) and $MgCl_2$ in water (**SR-04**, 40 µL, c = 2.5 mM). This resulted in the final reaction volume of 200 µL. The reaction mixture was then incubated in a thermocycler at 80 °C for 16–24 hours at 800 rpm. The progress of the reaction was monitored by LC–MS analysis. After the deprotection (approximately 24–48h), the reaction mixture was cooled to 23 °C and the mixture was then filtered using an Amicon ultra centrifugal filter (3 kDa MWCO). The retained DNA conjugate was dissolved in 8–10 µL of water to afford DNA conjugate **S20**. The DNA concentration was determined by measuring the A_{260} absorption using a Thermo Scientific™ NanoDrop™ One^{C}). The concentration was then adjusted by addition of Milli Q water to c = 2.0 mM. The yield of the DNA conjugate was calculated by measuring the integration of the peaks of the diode array detection (DAD) UV absorbance at 260 nm of the LC–MS trace, assuming complete DNA recovery and identical UV absorbance.

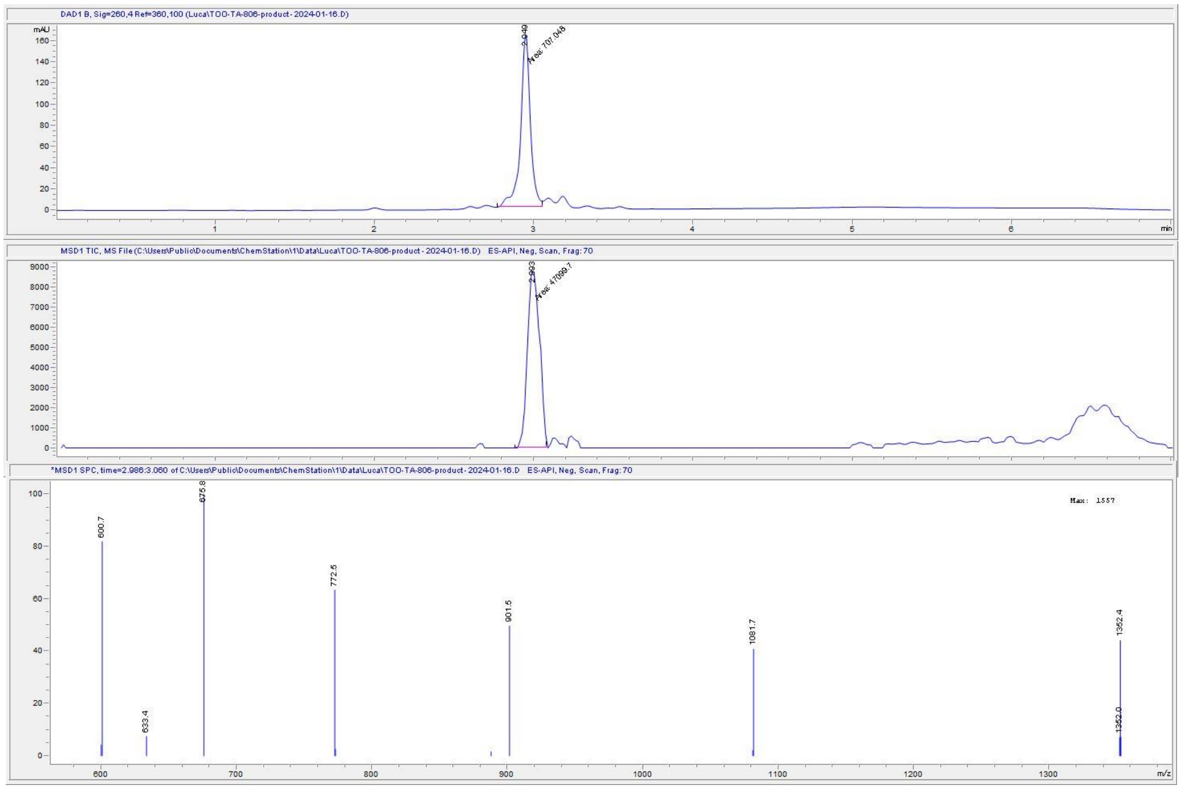

Figure S11. Analytical HPLC trace of **S20** with HPLC method B. (Top) DAD chromatogram at 260 nm. (Middle) TIC chromatogram. (Bottom) Ionization of peak at 2.99 min. containing reaction product.

DNA-conjugated substrate S21

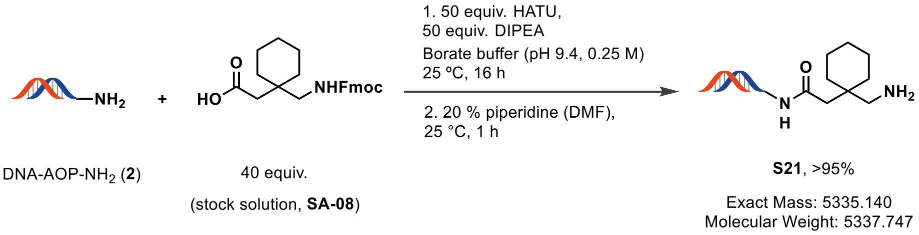

Preparation of *N*-Fmoc-Gabapentin stock solution: At 20–25 °C, a 1.5 mL Eppendorf tube was charged with *N*-Fmoc-Gabapentin (19.7 mg, 50.0 µmol). Next, 100 µL of DMA was added to the above 1.5 mL Eppendorf tube and the mixture was vortexed for 10 seconds. Next, the Eppendorf vial was sonicated for 10 seconds to ensure that the *N*-Fmoc acid is completely dissolved, resulting in the formation of *N*-Fmoc-Gabapentin stock solution **SA-08** in DMA (c = 0.50 M).

Under an ambient atmosphere, the stock solution of *N*-Fmoc-Gabapentin in DMA (**SA-08**, 2.0 µL, c = 0.50 M, 1.0 µmol, 50 equiv.) was added to a 1.5 mL Eppendorf tube, followed by the stock solution of HATU in DMA (**SR-03**, 2.0 µL, c = 0.50 M, 1.0 µmol, 50 equiv.). The mixture was vortexed for 5 seconds. Next, the stock solution of DIPEA in DMA (**SR-08**, 2.0 µL, c = 0.50 M, 1.0 µmol, 50 equiv.) was added. The mixture was vortexed for 5 seconds and left standing at 23 °C for 20 min.

In a separate 1.5 mL Eppendorf tube was added the stock solution **SD-01** of DNA-AOP-NH_2 (**2**) in water (10 µL, c = 2.0 mM, 20 nmol, 1.0 equiv.), followed by sodium borate buffer (10 µL, pH 9.4, c = 0.50 M). The solution of the first Eppendorf tube containing acid, HATU and DIPEA (6.0 µL DMA) was then added to above DNA-conjugate **2** solution. The reaction mixture was vortexed for 5 seconds, transferred to a thermocycler at 25 °C, and incubated for 16 hours at 600 rpm. After 16 hours, an aliquot of 1 µL of the reaction mixture was diluted to 40 µL with water for LC–MS analysis. Next, to the above reaction mixture was added the stock solution of NaCl in water (**SR-06**, 2.6 µL, c = 5.0 M, 10% volume of the total reaction volume), followed by cold ethanol (−20 °C, 86 µL) to precipitate the DNA conjugate. The Eppendorf tube was placed in a freezer (−20 °C) for at least 1 hour, and then it was centrifuged at 4 °C and 11000 × g for at least 30 minutes. The supernatant was removed and the pellet was dried under air, then dissolved in 20 µL water (c = 1.0 mM).

Fmoc deprotection: The piperidine stock solution **SR-07** (20 µL, 20% v/v, in DMF) was added to the above 1.5 mL Eppendorf tube containing the *N*-Fmoc-Gabapentin DNA conjugate (20 µL, c = 1.0 mM in water) and the resulting solution was vortexed for 5 seconds. The mixture was allowed to stand at 25 °C for 2 hours. Afterwards, an aliquot of 1 µL of the reaction mixture was diluted to 40 µL with water for LC–MS analysis. Next, to the above reaction mixture was added the stock solution of NaCl in water (**SR-06**, 4.0 µL, c = 5.0 M, 10% volume of the total reaction volume), followed by cold ethanol (−20 °C, 120 µL) to precipitate the DNA conjugate **S21**. The Eppendorf tube was placed in a freezer (−20 °C) for at least 1 hour and then it was centrifuged at 4 °C and 11000 × g for at least 30 minutes. The supernatant was decanted and the pellet was dried under air, then dissolved in 10 µL of water to obtain the DNA-conjugate **S21** (10 µL, c = 2.0 mM). The yield of the DNA conjugate was calculated by measuring the integration of the peaks of the diode array detection (DAD) UV absorbance at 260 nm of the LC–MS trace, assuming complete DNA recovery and identical UV absorbance.

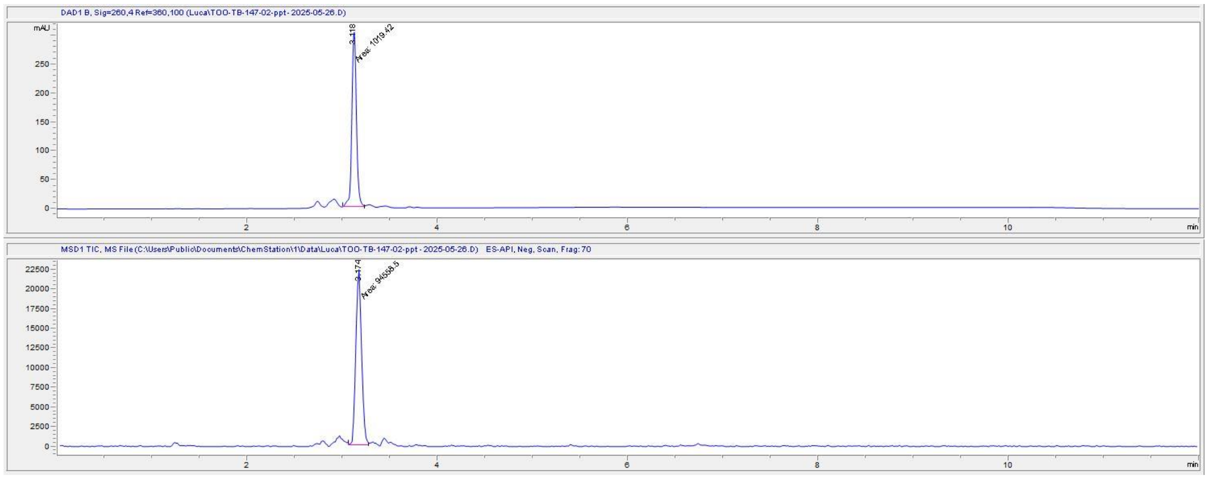

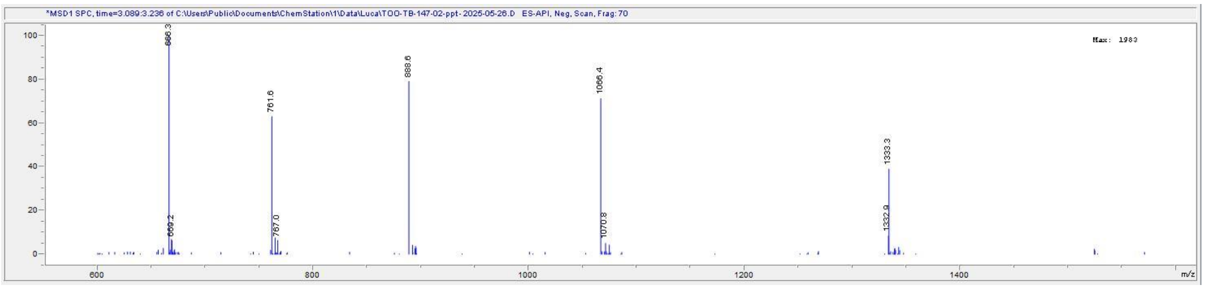

Figure S12. Analytical HPLC trace of **S21** with HPLC method A. (Top) DAD chromatogram at 260 nm. (Middle) TIC chromatogram. (Bottom) Ionization of peak at 3.17 min. containing reaction product.

DNA-conjugated substrate S22

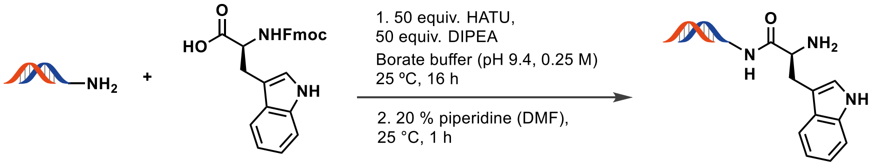

DNA-AOP-NH$_2$ (**2**)

50 equiv.

(stock solution, **SA-09**)

S22, >95%

Exact Mass: 5368.104
Molecular Weight: 5370.736

Preparation of *N*-Fmoc-*L*-tryptophan stock solution: At 20–25 °C, a 1.5 mL Eppendorf tube was charged with *N*-Fmoc-*L*-tryptophan (21.3 mg, 49.9 µmol). Next, 100 µL of DMA was added to the above 1.5 mL Eppendorf tube and the mixture was vortexed for 10 seconds. Next, the Eppendorf vial was sonicated for 10 seconds to ensure that the *N*-Fmoc-*L*-tryptophan is completely dissolved, resulting in the formation of *N*-Fmoc-*L*-tryptophan stock solution **SA-09** in DMA (c = 0.50 M).

Under an ambient atmosphere, the stock solution of *N*-Fmoc-*L*-tryptophan in DMA (**SA-09**, 2.0 µL, c = 0.50 M, 1.0 µmol, 50 equiv.) was added to a 1.5 mL Eppendorf tube, followed by the stock solution of HATU in DMA (**SR-03**, 2.0 µL, c = 0.50 M, 1.0 µmol, 50 equiv.). The mixture was vortexed for 5 seconds. Next, the stock solution of DIPEA in DMA (**SR-08**, 2.0 µL, c = 0.50 M, 1.0 µmol, 50 equiv.) was added. The mixture was vortexed for 5 seconds and left standing at 23 °C for 20 min.

In a separate 1.5 mL Eppendorf tube was added the stock solution **SD-01** of DNA-AOP-NH$_2$ (**2**) in water (10 µL, c = 2.0 mM, 20 nmol, 1.0 equiv.), followed by sodium borate buffer (10 µL, pH 9.4, c = 0.50 M). The solution of the first Eppendorf tube containing acid, HATU and DIPEA (6.0 µL DMA) was then added to above DNA-conjugate **2** solution. The reaction mixture was vortexed for 5 seconds, transferred to a thermocycler at 25 °C, and incubated for 16 hours at 600 rpm. After 16 hours, an aliquot of 1 µL of the reaction mixture was diluted to 40 µL with water for LC–MS analysis. Next, to the above reaction mixture was added the stock solution of NaCl in water (**SR-06**, 2.6 µL, c = 5.0 M, 10% volume of the total reaction volume), followed by cold ethanol (−20 °C, 86 µL) to precipitate the DNA conjugate. The Eppendorf tube was placed in a freezer (−20 °C) for at least 1 hour, and then it was centrifuged at 4 °C and 11000 × g for at least 30 minutes. The supernatant was removed and the pellet was dried under air, then dissolved in 20 µL water (c = 1.0 mM).

Fmoc deprotection: Fmoc deprotection: The piperidine stock solution **SR-07** (20 µL, 20% v/v, in DMF) was added to the above 1.5 mL Eppendorf tube containing the *N*-Fmoc tryptophan DNA conjugate (20 µL, c = 1.0 mM in water) and the resulting solution was vortexed for 5 seconds. The mixture was allowed to stand at 25 °C for 2 hours. Afterwards, an aliquot of 1 µL of the reaction mixture was diluted to 40 µL with water for LC–MS analysis. Next, to the above reaction mixture was added the stock solution of NaCl in water (**SR-06**, 4.0 µL, c = 5.0 M, 10% volume of the total reaction volume), followed by cold ethanol (−20 °C, 120 µL) to precipitate the DNA conjugate **S22**. The Eppendorf tube was placed in a freezer (−20 °C) for at least 1 hour and then it was centrifuged at 4 °C and 11000 × g for at least 30 minutes. The supernatant was decanted and the pellet was dried under air, then dissolved in 10 µL of water to obtain the DNA-conjugate **S22** (10 µL, c = 2.0 mM). The yield of the DNA conjugate was calculated by measuring the integration of the peaks of the diode array detection (DAD) UV absorbance at 260 nm of the LC–MS trace, assuming complete DNA recovery and identical UV absorbance.

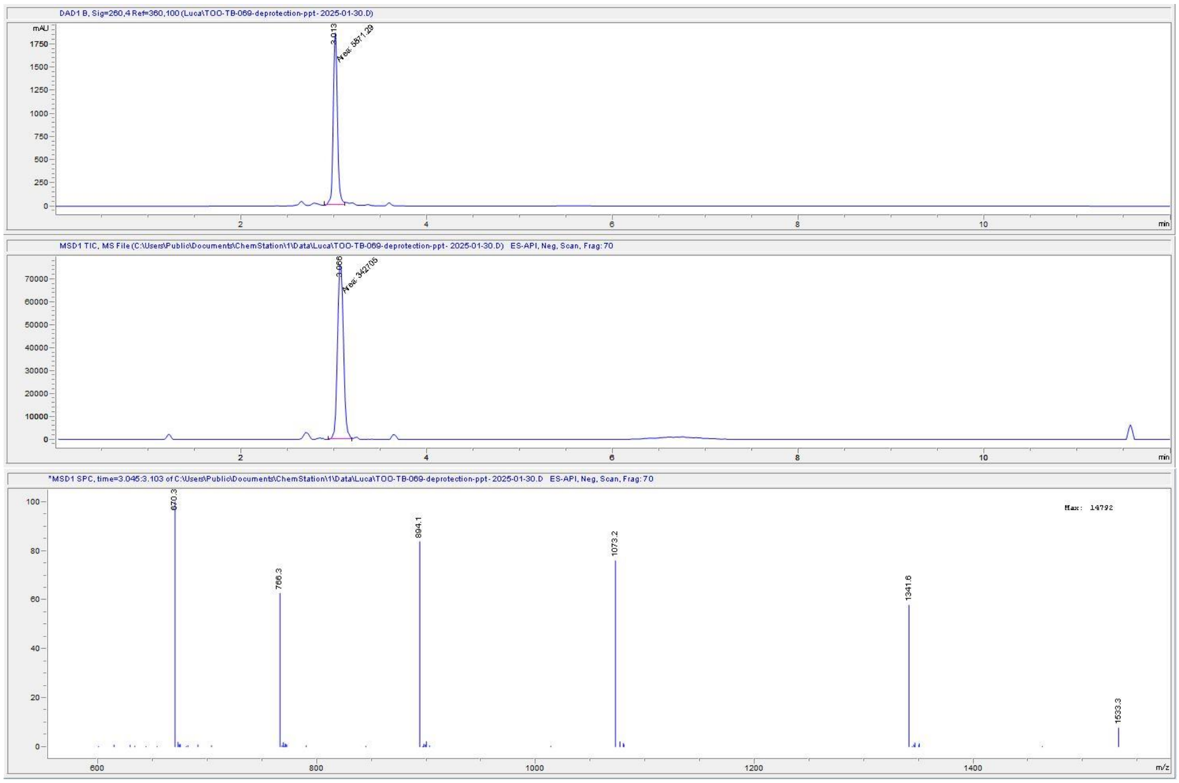

Figure S13. Analytical HPLC trace of **S22** with HPLC method A. (Top) DAD chromatogram at 260 nm. (Middle) TIC chromatogram. (Bottom) Ionization of peak at 3.06 min. containing reaction product.

DNA-conjugated substrate S23

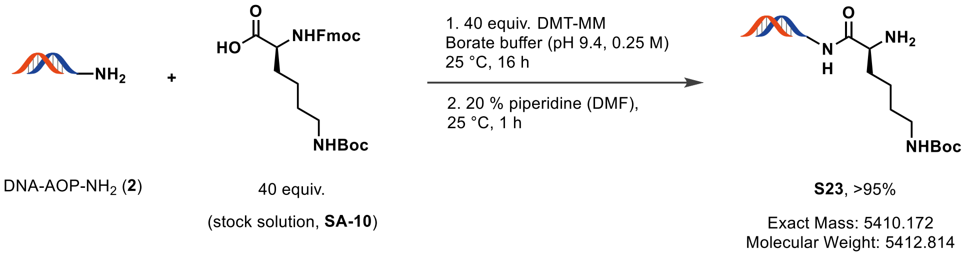

Preparation of *N*-Fmoc-*N-Boc*-lysine stock solution (SA-10): Under an ambient atmosphere, a 1.5 mL Eppendorf tube was charged with *N*-Fmoc-*N*-Boc-lysine (18.7 mg, 39.9 µmol). Next, 100 µL of DMA was added to the above 1.5 mL Eppendorf tube and the mixture was vortexed for 10 seconds. Next, the Eppendorf tube was sonicated for 10 seconds to ensure that the *N*-Fmoc amino acid is completely dissolved, resulting in the formation of *N*-Fmoc-*N*-Boc-lysine stock solution **SA-10** in DMA (c = 0.40 M).

Under an ambient atmosphere, the stock solution **SD-01** of DNA-AOP-NH_2 (**2**) in water (10 µL, c = 2.0 mM, 20 nmol, 1.0 equiv.) was added to a 1.5 mL Eppendorf tube, followed by sodium borate buffer (10 µL, pH 9.4, c = 0.50 M). Next, the stock solution of *N*-Fmoc-*N*-Boc-lysine in DMA (**SA-10**, 2.0 µL, c = 0.40 M, 0.80 µmol, 40 equiv.) was added. The reaction mixture was vortexed for 5 seconds. Then, the stock solution of DMT-MM in water (**SR-02**, 2.0 µL, c = 0.40 M, 0.80 µmol, 40 equiv.) was added. The mixture was vortexed for 5 seconds, transferred to a thermocycler at 25 °C, and incubated for 16 hours at 600 rpm. After 16 hours, an aliquot of 1.0 µL of the reaction mixture was diluted to 40 µL with water for LC–MS analysis. Next, to the above reaction mixture was added the stock solution of NaCl in water (**SR-06**, 2.4 µL, c = 5.0 M, 10% volume of the total reaction volume), followed by cold ethanol (−20 °C, 80 µL) to precipitate the *N*-Fmoc-*N*-Boc lysine DNA conjugate. The Eppendorf tube was placed in a freezer (−20 °C) for at least 1 hour, and then it was centrifuged at 4 °C and 11000 × g for at least 30 minutes. The supernatant was removed and the pellet was dried under air, then dissolved in 20 µL water (c = 1.0 mM).

Fmoc deprotection: The piperidine stock solution **SR-07** (20 µL, 20% v/v, in DMF) was added to the above 1.5 mL Eppendorf tube containing the *N*-Fmoc-*N*-Boc lysine DNA conjugate (20 µL, c = 1.0 mM in water) and the resulting solution was vortexed for 5 seconds. The mixture was allowed to stand at 25 °C for 2 hours. Afterwards, an aliquot of 1 µL of the reaction mixture was diluted to 40 µL with water for LC–MS analysis. Next, to the above reaction mixture was added the stock solution of NaCl in water (**SR-06**, 4.0 µL, c = 5.0 M, 10% volume of the total reaction volume), followed by cold ethanol (−20 °C, 120 µL) to precipitate the DNA conjugate **S23**. The Eppendorf tube was placed in a freezer (−20 °C) for at least 1 hour and then it was centrifuged at 4 °C and 11000 × g for at least 30 minutes. The supernatant was decanted and the pellet was dried under air, then dissolved in 10 µL of water to obtain the DNA- conjugate **S23** (10 µL, c = 2.0 mM). The yield of the DNA conjugate was calculated by measuring the integration of the peaks of the diode array detection (DAD) UV absorbance at 260 nm of the LC–MS trace, assuming complete DNA recovery and identical UV absorbance.

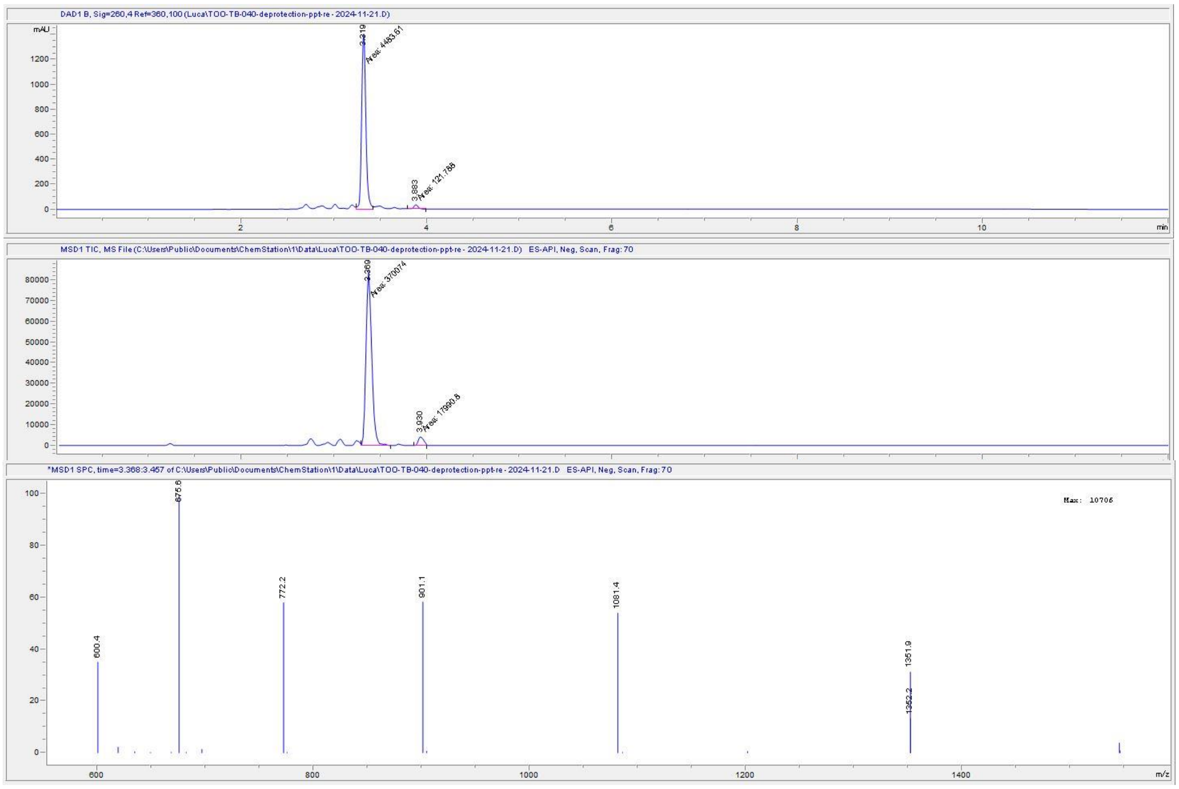

Figure S14. Analytical HPLC trace of **S23** with HPLC method A. (Top) DAD chromatogram at 260 nm. (Middle) TIC chromatogram. (Bottom) Ionization of peak at 3.37 min. containing reaction product.

DNA-conjugated substrate S24

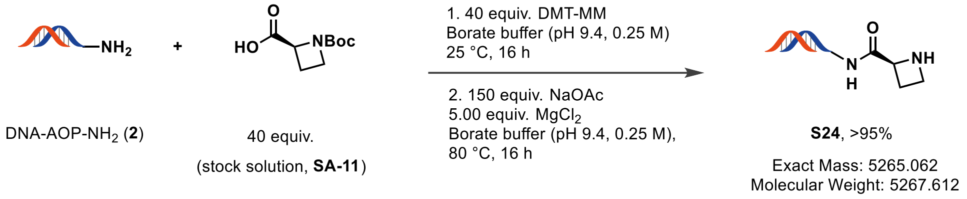

Preparation of (*S*)-1-(*tert*-butoxycarbonyl)azetidine-2-carboxylic acid stock solution (SA-11): At 20–25 °C, a 1.5 mL Eppendorf tube was charged with (*S*)-1-(*tert*-butoxycarbonyl)azetidine-2-carboxylic acid (16.1 mg, 80.0 µmol). Next, 200 µL of DMA was added to the above 1.5 mL Eppendorf tube and the mixture was vortexed for 10 seconds. Next, the Eppendorf vial was sonicated for 10 seconds to make sure that the *N*-Boc acid is completely dissolved, which resulted in the formation of the (*S*)-1-(*tert*-butoxycarbonyl)azetidine-2-carboxylic acid stock solution (**SA-11**) (c = 0.40 M) in DMA.

At 20–25 °C, the stock solution **SD-01** of DNA-AOP-NH_2 (**2**) (10 µL, c = 2.0 mM, 20 nmol, 1.0 equiv.) in water was added to a 1.5 mL Eppendorf tube, followed by sodium borate buffer (10 µL, pH 9.4, c = 0.50 M). Next, the stock solution of (*S*)-1-(*tert*-butoxycarbonyl)azetidine-2-carboxylic acid (**SA-11**, 2.0 µL , c = 0.40 M, 0.80 µmol, 40 equiv.) in DMA was added. The reaction mixture was vortexed for 5 seconds. Then, the stock solution of DMT-MM (**SR-02**, 2.0 µL, c = 0.40 M, 0.80 µmol, 40 equiv.) in water was added. The mixture was

vortexed for 5 seconds, transferred to a thermocycler at 25 °C, and incubated for 16 hours at 600 rpm. After 16 hours, an aliquot of 1.0 µL of the reaction mixture was diluted to 40 µL with water for LC–MS analysis. Next, to the above reaction mixture was added the stock solution of NaCl in water (**SR-06**, 2.4 µL, c = 5.0 M, 10% volume of the total reaction volume), followed by cold ethanol (−20 °C, 80 µL) to precipitate the *N*-Boc DNA conjugate. The Eppendorf tube was placed in a freezer (−20 °C) for at least 1 hour, and then it was centrifuged at 4 °C and 11000 × g for at least 30 minutes. The supernatant was removed and the pellet was dried under air, then dissolved in 20 µL water (c = 1.0 mM).

Boc deprotection: To the above 1.5 mL Eppendorf tube containing the *N*-Boc DNA-conjugate (20 µL, c = 1.0 mM) in water, was added sodium borate buffer (100 µL, pH 9.4, c = 0.50 M), followed by stock solutions of NaOAc in water (**SR-05**, 40 µL, c = 75 mM) and $MgCl_2$ in water (**SR-04**, 40 µL, c = 2.5 mM). This resulted in the final reaction volume of 200 µL. The reaction mixture was then incubated in a thermocycler at 80 °C for 16–24 hours at 800 rpm. The progress of the reaction was monitored by LC–MS analysis. After the deprotection (approximately 24–48h), the reaction mixture was cooled to 23 °C and the mixture was then filtered using an Amicon ultra centrifugal filter (3 kDa MWCO). The retained DNA conjugate was dissolved in 8–10 µL of water to afford DNA conjugate **S24**. The DNA concentration was determined by measuring the A_{260} absorption using a Thermo Scientific™ NanoDrop™ One^{C}). The concentration was then adjusted by addition of Milli Q water to c = 2.0 mM. The yield of the DNA conjugate was calculated by measuring the integration of the peaks of the diode array detection (DAD) UV absorbance at 260 nm of the LC–MS trace, assuming complete DNA recovery and identical UV absorbance.

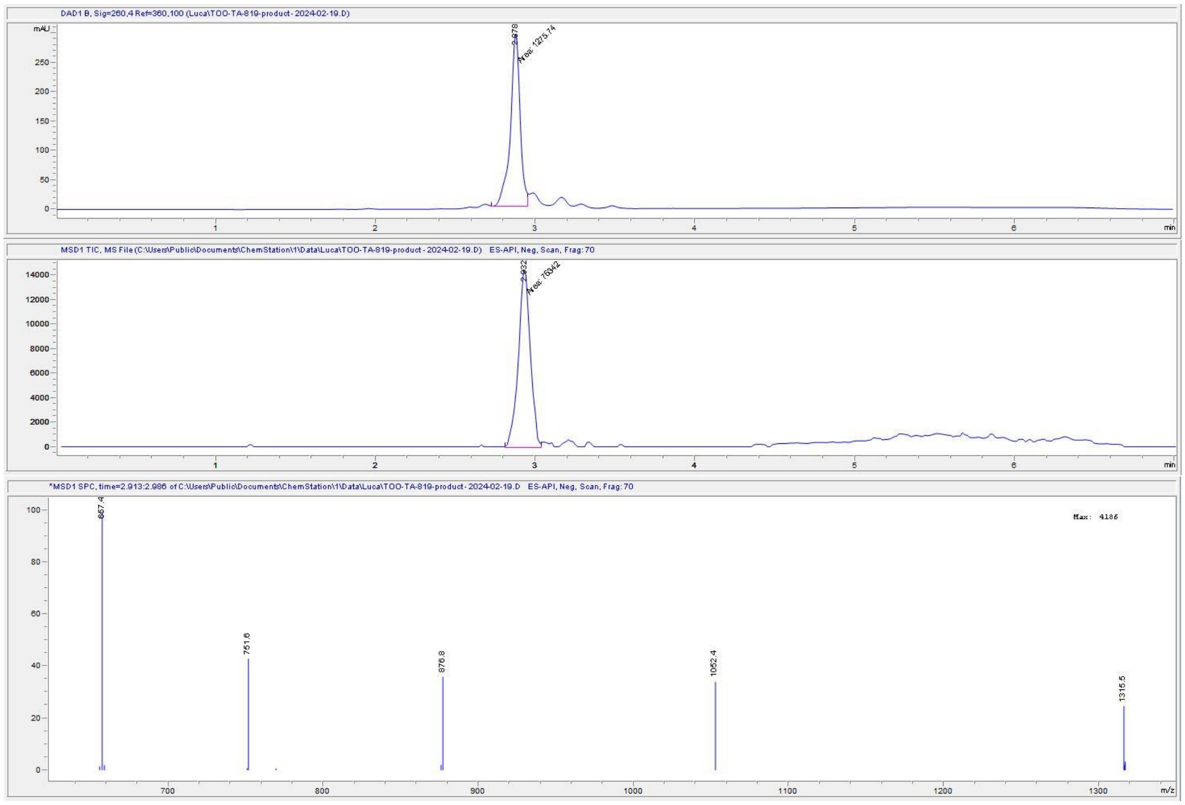

Figure S15. Analytical HPLC trace of **S24** with HPLC method B. (Top) DAD chromatogram at 260 nm. (Middle) TIC chromatogram. (Bottom) Ionization of peak at 2.93 min. containing reaction product.

DNA-conjugated substrate S25

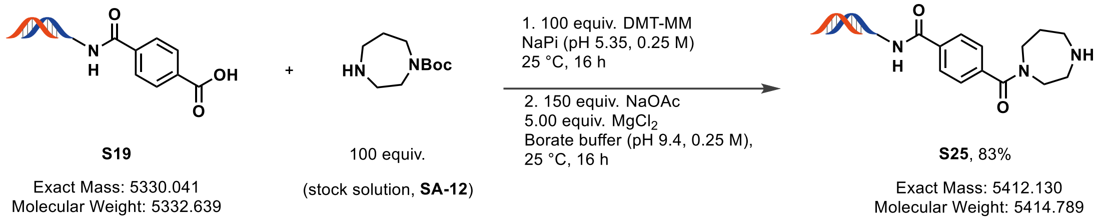

Preparation of *tert*-butyl 1,4-diazepane-1-carboxylate stock solution (SA-12): At 20–25 °C, a 1.5 mL Eppendorf tube was charged with *tert*-butyl 1,4-diazepane-1-carboxylate (16.1 mg, 80.0 µmol). Next, 80 µL of DMA was added to the above 1.5 mL Eppendorf tube and the mixture was vortexed for 10 seconds. Next, the Eppendorf vial was sonicated for 10 seconds to make sure that the *N*-Boc protected amine is completely dissolved, resulting in the formation of *tert*-butyl 1,4-diazepane-1-carboxylate stock solution in DMA (**SA-12**, c = 1.0 M).

Under an ambient atmosphere, the stock solution of DNA-conjugated carboxylic acid **S19** (10 µL, c = 2.0 mM, 20 nmol, 1.0 equiv.) in water was added to a 1.5 mL Eppendorf tube, followed by NaPi buffer (10 µL, pH 5.3, c = 0.50 M). Next, the stock solution of the amine in DMA (**SA-12**, 2.0 µL, c = 1.0 M, 2.0 µmol, 100 equiv.) was added. The reaction mixture was vortexed for 5 seconds. Then, the stock solution of DMT-MM in water (**SR-02**, 2.0 µL, c = 1.0 M, 2.0 µmol, 100 equiv.) was added. The mixture was vortexed for 5 seconds, transferred to a thermocycler at 25 °C, and incubated for 16 hours at 600 rpm. After 16 hours, an aliquot of 1.0 µL of the reaction mixture was diluted to 40 µL with water for LC–MS analysis. Next, to the above reaction mixture was added the stock solution of NaCl in water (**SR-06**, 2.4 µL, c = 5.0 M, 10% volume of the total reaction volume), followed by cold ethanol (−20 °C, 80 µL) to precipitate the *N*-Boc DNA conjugate. The Eppendorf tube was placed in a freezer (−20 °C) for at least 1 hour, and then it was centrifuged at 4 °C and 11000 × g for at least 30 minutes. The supernatant was removed and the pellet was dried under air, then dissolved in 20 µL water (c = 1.0 mM).

Boc deprotection: To the above 1.5 mL Eppendorf tube containing the *N*-Boc DNA-conjugate (20 µL, c = 1.0 mM) in water, was added sodium borate buffer (100 µL, pH 9.4, c = 0.50 M), followed by stock solutions of NaOAc in water (**SR-05**, 40 µL, c = 75 mM) and $MgCl_2$ in water (**SR-04**, 40 µL, c = 2.5 mM). This resulted in the final reaction volume of 200 µL. The reaction mixture was then incubated in a thermocycler at 80 °C for 16–24 hours at 800 rpm. The progress of the reaction was monitored by LC–MS analysis. After the deprotection (approximately 24–48h), the reaction mixture was cooled to 23 °C and the mixture was then filtered using an Amicon ultra centrifugal filter (3 kDa MWCO). The retained DNA conjugate was dissolved in 8–10 µL of water to afford DNA conjugate **S25**. The DNA concentration was determined by measuring the A_{260} absorption using a Thermo Scientific™ NanoDrop™ One^{C}). The concentration was then adjusted by addition of Milli Q water to c = 2.0 mM. The yield of the DNA conjugate was calculated by measuring the

integration of the peaks of the diode array detection (DAD) UV absorbance at 260 nm of the LC–MS trace, assuming complete DNA recovery and identical UV absorbance.

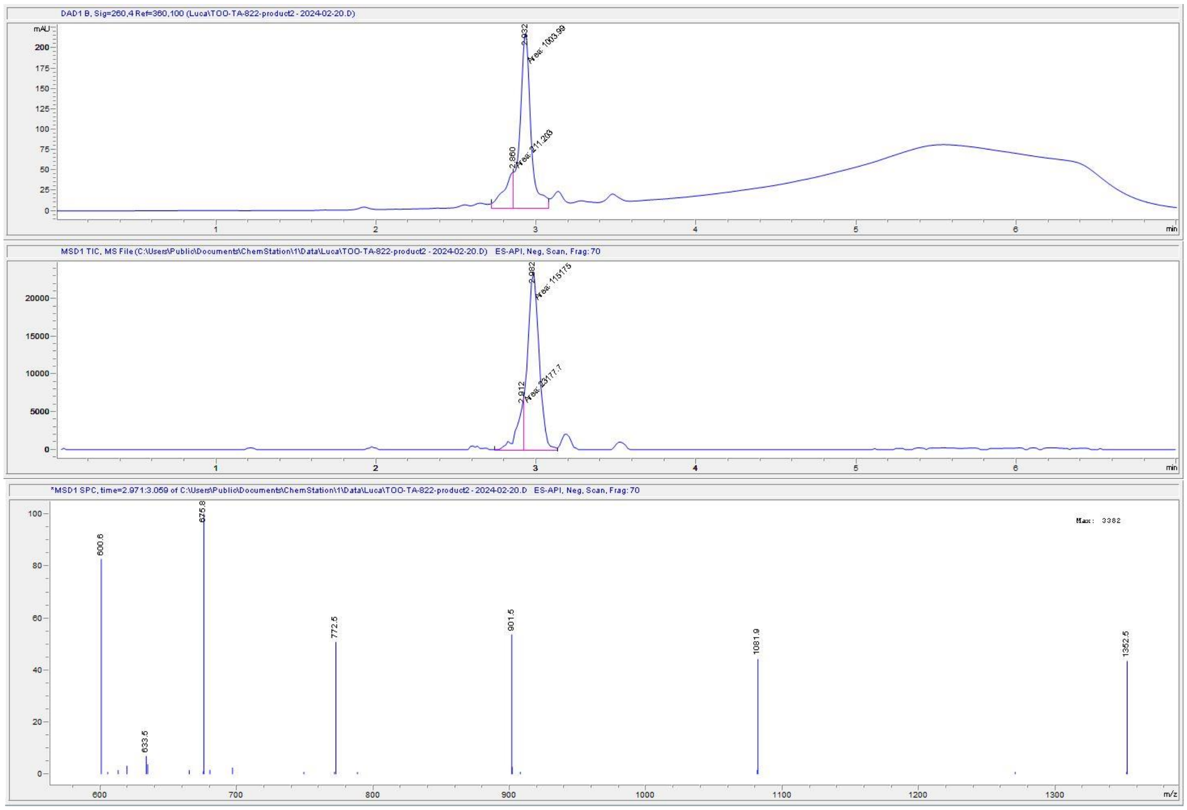

Figure S16. Analytical HPLC trace of **S25** with HPLC method B. (Top) DAD chromatogram at 260 nm. (Middle) TIC chromatogram. (Bottom) Ionization of peak at 2.98 min. containing reaction product.

DNA-conjugated substrate S26

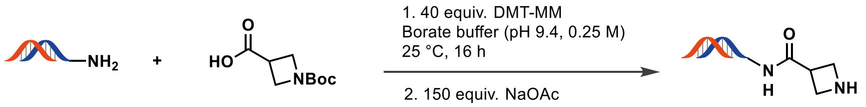

DNA-AOP-NH2 (**2**)

40 equiv.

(stock solution, **SA-13**)

S26, >95%

Exact Mass: 5265.062
Molecular Weight: 5267.612

Preparation of 1-(*tert*-butoxycarbonyl)azetidine-3-carboxylic acid stock solution (SA-13): At 20–25 °C, a 1.5 mL Eppendorf tube was charged with 1-(*tert*-butoxycarbonyl)azetidine-3-carboxylic acid (16.1 mg, 80.0 µmol). Next, 200 µL of DMA was added to the above 1.5 mL Eppendorf tube and the mixture was vortexed for 10 seconds. Next, the Eppendorf vial was sonicated for 10 seconds to make sure that the *N*-Boc acid is completely dissolved, which resulted in the formation of the 1-(*tert*-butoxycarbonyl)azetidine-3-carboxylic acid stock solution (**SA-13**) (c = 0.40 M) in DMA.

At 20–25 °C, the stock solution **SD-01** of DNA-AOP-NH2 (**2**) (10 µL, c = 2.0 mM, 20 nmol, 1.0 equiv.) in water was added to a 1.5 mL Eppendorf tube, followed by sodium borate buffer (10 µL, pH 9.4, c = 0.50 M). Next,

the stock solution of 1-(*tert*-butoxycarbonyl)azetidine-3-carboxylic acid (**SA-13**, 2.0 µL , c = 0.40 M, 0.80 µmol, 40 equiv.) in DMA was added. The reaction mixture was vortexed for 5 seconds. Then, the stock solution of DMT-MM (**SR-02**, 2.0 µL, c = 0.40 M, 0.80 µmol, 40 equiv.) in water was added. The mixture was vortexed for 5 seconds, transferred to a thermocycler at 25 °C, and incubated for 16 hours at 600 rpm. After 16 hours, an aliquot of 1.0 µL of the reaction mixture was diluted to 40 µL with water for LC–MS analysis. Next, to the above reaction mixture was added the stock solution of NaCl in water (**SR-06**, 2.4 µL, c = 5.0 M, 10% volume of the total reaction volume), followed by cold ethanol (−20 °C, 80 µL) to precipitate the *N*-Boc DNA conjugate. The Eppendorf tube was placed in a freezer (−20 °C) for at least 1 hour, and then it was centrifuged at 4 °C and 11000 × g for at least 30 minutes. The supernatant was removed and the pellet was dried under air, then dissolved in 20 µL water (c = 1.0 mM).

Boc deprotection: To the above 1.5 mL Eppendorf tube containing the *N*-Boc DNA-conjugate (20 µL, c = 1.0 mM) in water, was added sodium borate buffer (100 µL, pH 9.4, c = 0.50 M), followed by stock solutions of NaOAc in water (**SR-05**, 40 µL, c = 75 mM) and $MgCl_2$ in water (**SR-04**, 40 µL, c = 2.5 mM). This resulted in the final reaction volume of 200 µL. The reaction mixture was then incubated in a thermocycler at 80 °C for 16–24 hours at 800 rpm. The progress of the reaction was monitored by LC–MS analysis. After the deprotection (approximately 24–48h), the reaction mixture was cooled to 23 °C and the mixture was then filtered using an Amicon ultra centrifugal filter (3 kDa MWCO). The retained DNA conjugate was dissolved in 8–10 µL of water to afford DNA conjugate **S26**. The DNA concentration was determined by measuring the A_{260} absorption using a Thermo Scientific™ NanoDrop™ One^{C}). The concentration was then adjusted by addition of Milli Q water to c = 2.0 mM. The yield of the DNA conjugate was calculated by measuring the integration of the peaks of the diode array detection (DAD) UV absorbance at 260 nm of the LC–MS trace, assuming complete DNA recovery and identical UV absorbance.

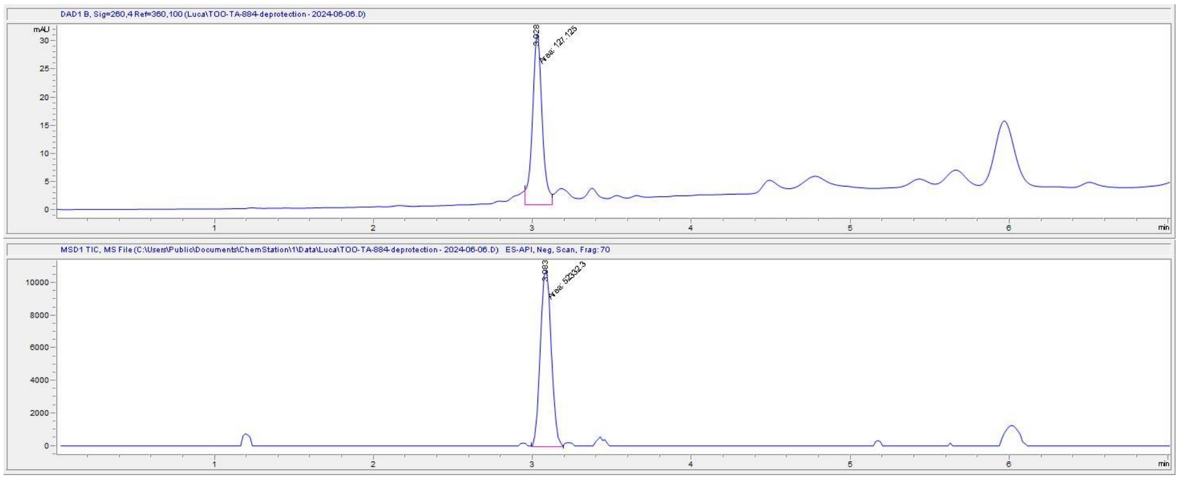

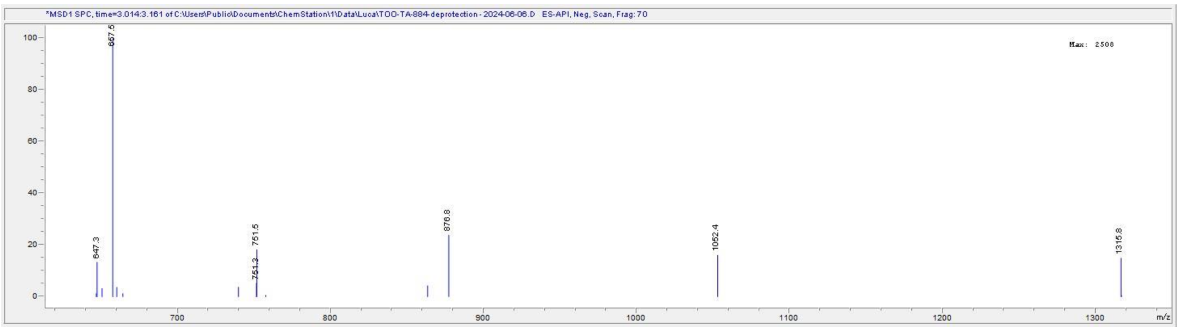

Figure S17. Analytical HPLC trace of **S26** with HPLC method B. (Top) DAD chromatogram at 260 nm. (Middle) TIC chromatogram. (Bottom) Ionization of peak at 3.08 min. containing reaction product.

DNA-conjugated substrate S27

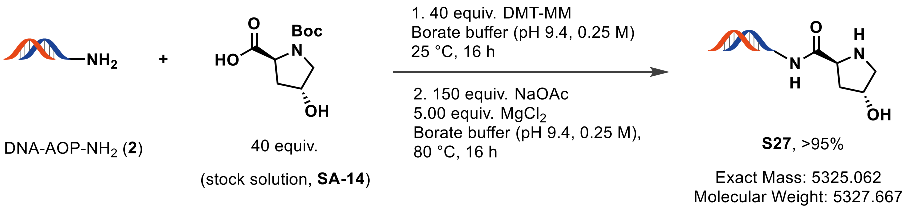

Preparation of (*2S*,*4R*)-1-(*tert*-butoxycarbonyl)-4-hydroxypyrrolidine-2-carboxylic acid stock solution (SA-14): At 20–25 °C, a 1.5 mL Eppendorf tube was charged with (*2S*,*4R*)-1-(*tert*-butoxycarbonyl)-4-hydroxypyrrolidine-2-carboxylic acid (9.2 mg, 39 µmol). Next, 100 µL of DMA was added to the above 1.5 mL Eppendorf tube and the mixture was vortexed for 10 seconds. Next, the Eppendorf vial was sonicated for 10 seconds to make sure that the *N*-Boc acid is completely dissolved, which resulted in the formation of the (*2S*,*4R*)-1-(*tert*-butoxycarbonyl)-4-hydroxypyrrolidine-2-carboxylic acid stock solution (**SA-14**) (c = 0.40 M) in DMA.

At 20–25 °C, the stock solution **SD-01** of DNA-AOP-NH_2 (**2**) (10 µL, c = 2.0 mM, 20 nmol, 1.0 equiv.) in water was added to a 1.5 mL Eppendorf tube, followed by sodium borate buffer (10 µL, pH 9.4, c = 0.50 M). Next, the stock solution of (*2S*,*4R*)-1-(*tert*-butoxycarbonyl)-4-hydroxypyrrolidine-2-carboxylic acid (**SA-14**, 2.0 µL, c = 0.40 M, 0.80 µmol, 40 equiv.) in DMA was added. The reaction mixture was vortexed for 5 seconds. Then, the stock solution of DMT-MM (**SR-02**, 2.0 µL, c = 0.40 M, 0.80 µmol, 40 equiv.) in water was added. The mixture was vortexed for 5 seconds, transferred to a thermocycler at 25 °C, and incubated for 16 hours at 600 rpm. After 16 hours, an aliquot of 1.0 µL of the reaction mixture was diluted to 40 µL with water for LC–MS analysis. Next, to the above reaction mixture was added the stock solution of NaCl in water (**SR-06**, 2.4 µL, c = 5.0 M, 10% volume of the total reaction volume), followed by cold ethanol (−20 °C, 80 µL) to precipitate the *N*-Boc DNA conjugate. The Eppendorf tube was placed in a freezer (−20 °C) for at least 1 hour, and then it was centrifuged at 4 °C and 11000 × g for at least 30 minutes. The supernatant was removed and the pellet was dried under air, then dissolved in 20 µL water (c = 1.0 mM).

Boc deprotection: To the above 1.5 mL Eppendorf tube containing the *N*-Boc DNA-conjugate (20 µL, c = 1.0 mM) in water, was added sodium borate buffer (100 µL, pH 9.4, c = 0.50 M), followed by stock

solutions of NaOAc in water (**SR-05**, 40 µL, c = 75 mM) and $MgCl_2$ in water (**SR-04**, 40 µL, c = 2.5 mM). This resulted in the final reaction volume of 200 µL. The reaction mixture was then incubated in a thermocycler at 80 °C for 16–24 hours at 800 rpm. The progress of the reaction was monitored by LC–MS analysis. After the deprotection (approximately 24–48h), the reaction mixture was cooled to 23 °C and the mixture was then filtered using an Amicon ultra centrifugal filter (3 kDa MWCO). The retained DNA conjugate was dissolved in 8–10 µL of water to afford DNA conjugate **S27**. The DNA concentration was determined by measuring the A_{260} absorption using a Thermo Scientific™ NanoDrop™ One^{C}). The concentration was then adjusted by addition of Milli Q water to c = 2.0 mM. The yield of the DNA conjugate was calculated by measuring the integration of the peaks of the diode array detection (DAD) UV absorbance at 260 nm of the LC–MS trace, assuming complete DNA recovery and identical UV absorbance.

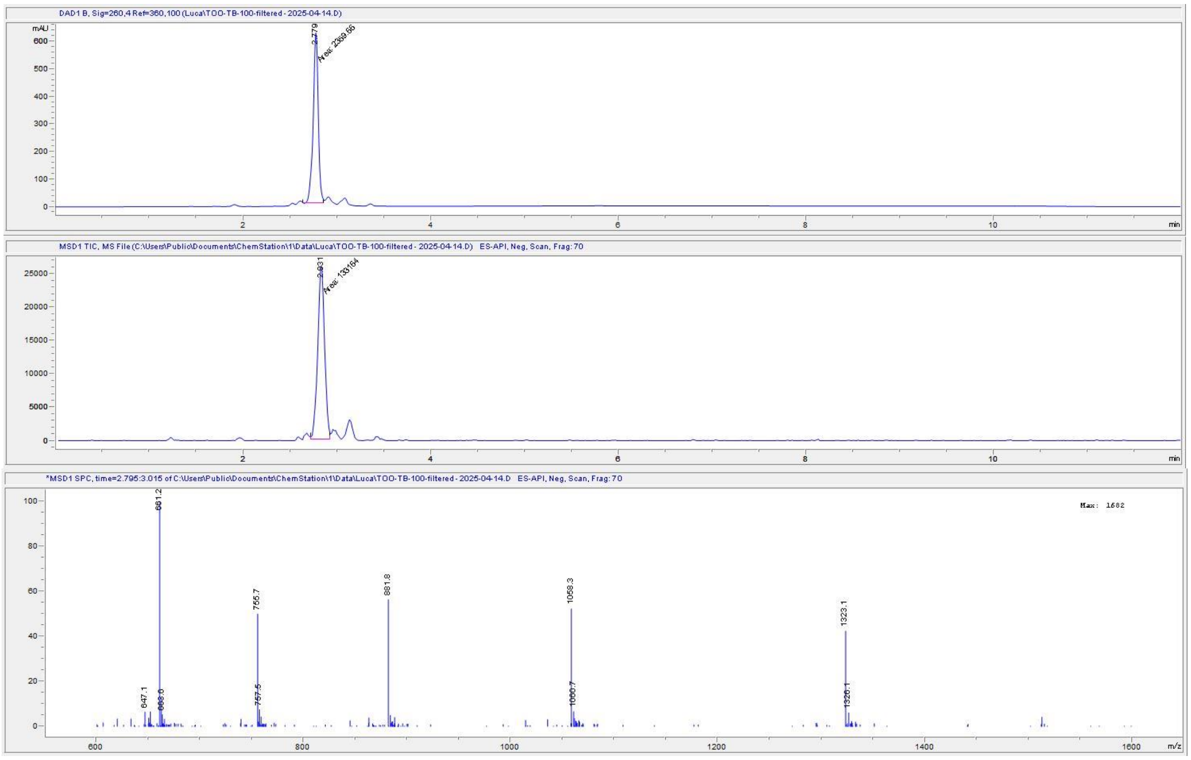

Figure S18. Analytical HPLC trace of **S27** with HPLC method A. (Top) DAD chromatogram at 260 nm. (Middle) TIC chromatogram. (Bottom) Ionization of peak at 2.83 min. containing reaction product.

DNA-conjugated substrate S28

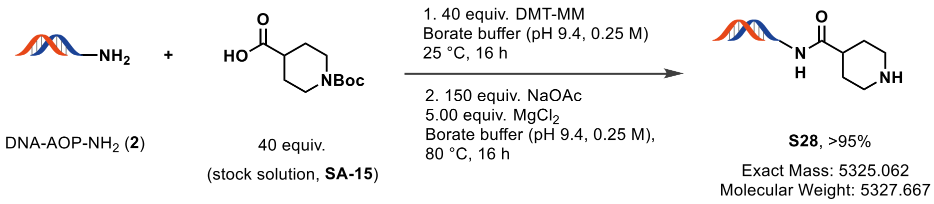

Preparation of *N*-Boc-piperidine-4-carboxylic acid stock solution (SA-15): At 20–25 °C, a 1.5 mL Eppendorf tube was charged with 1-(*tert*-butoxycarbonyl)piperidine-4-carboxylic acid (9.1 mg, 39 µmol). Next,

100 µL of DMA was added to the above 1.5 mL Eppendorf tube and the mixture was vortexed for 10 seconds. Next, the Eppendorf vial was sonicated for 10 seconds to make sure that the *N*-Boc acid is completely dissolved, which resulted in the formation of the 1-(*tert*-butoxycarbonyl)piperidine-4-carboxylic acid stock solution (**SA-15**) (c = 0.40 M) in DMA.

At 20–25 °C, the stock solution **SD-01** of DNA-AOP-NH_2 (**2**) (10 µL, c = 2.0 mM, 20 nmol, 1.0 equiv.) in water was added to a 1.5 mL Eppendorf tube, followed by sodium borate buffer (10 µL, pH 9.4, c = 0.50 M). Next, the stock solution of 1-(*tert*-butoxycarbonyl)piperidine-4-carboxylic acid (**SA-15**, 2.0 µL , c = 0.40 M, 0.80 µmol, 40 equiv.) in DMA was added. The reaction mixture was vortexed for 5 seconds. Then, the stock solution of DMT-MM (**SR-02**, 2.0 µL, c = 0.40 M, 0.80 µmol, 40 equiv.) in water was added. The mixture was vortexed for 5 seconds, transferred to a thermocycler at 25 °C, and incubated for 16 hours at 600 rpm. After 16 hours, an aliquot of 1.0 µL of the reaction mixture was diluted to 40 µL with water for LC–MS analysis. Next, to the above reaction mixture was added the stock solution of NaCl in water (**SR-06**, 2.4 µL, c = 5.0 M, 10% volume of the total reaction volume), followed by cold ethanol (−20 °C, 80 µL) to precipitate the *N*-Boc piperidine DNA conjugate. The Eppendorf tube was placed in a freezer (−20 °C) for at least 1 hour, and then it was centrifuged at 4 °C and 11000 × g for at least 30 minutes. The supernatant was removed and the pellet was dried under air, then dissolved in 20 µL water (c = 1.0 mM).

Boc deprotection: To the above 1.5 mL Eppendorf tube containing the *N*-Boc piperidine DNA-conjugate (20 µL, c = 1.0 mM) in water, was added sodium borate buffer (100 µL, pH 9.4, c = 0.50 M), followed by stock solutions of NaOAc in water (**SR-05**, 40 µL, c = 75 mM) and $MgCl_2$ in water (**SR-04**, 40 µL, **c** = 2.5 mM). This resulted in the final reaction volume of 200 µL. The reaction mixture was then incubated in a thermocycler at 80 °C for 16–24 hours at 800 rpm. The progress of the reaction was monitored by LC–MS analysis. After the deprotection (approximately 24–48h), the reaction mixture was cooled to 23 °C and the mixture was then filtered using an Amicon ultra centrifugal filter (3 kDa MWCO). The retained DNA conjugate was dissolved in 8–10 µL of water to afford DNA conjugate **S28**. The DNA concentration was determined by measuring the A_{260} absorption using a Thermo Scientific™ NanoDrop™ One^{C}). The concentration was then adjusted by addition of Milli Q water to c = 2.0 mM. The yield of the DNA conjugate was calculated by measuring the integration of the peaks of the diode array detection (DAD) UV absorbance at 260 nm of the LC–MS trace, assuming complete DNA recovery and identical UV absorbance.

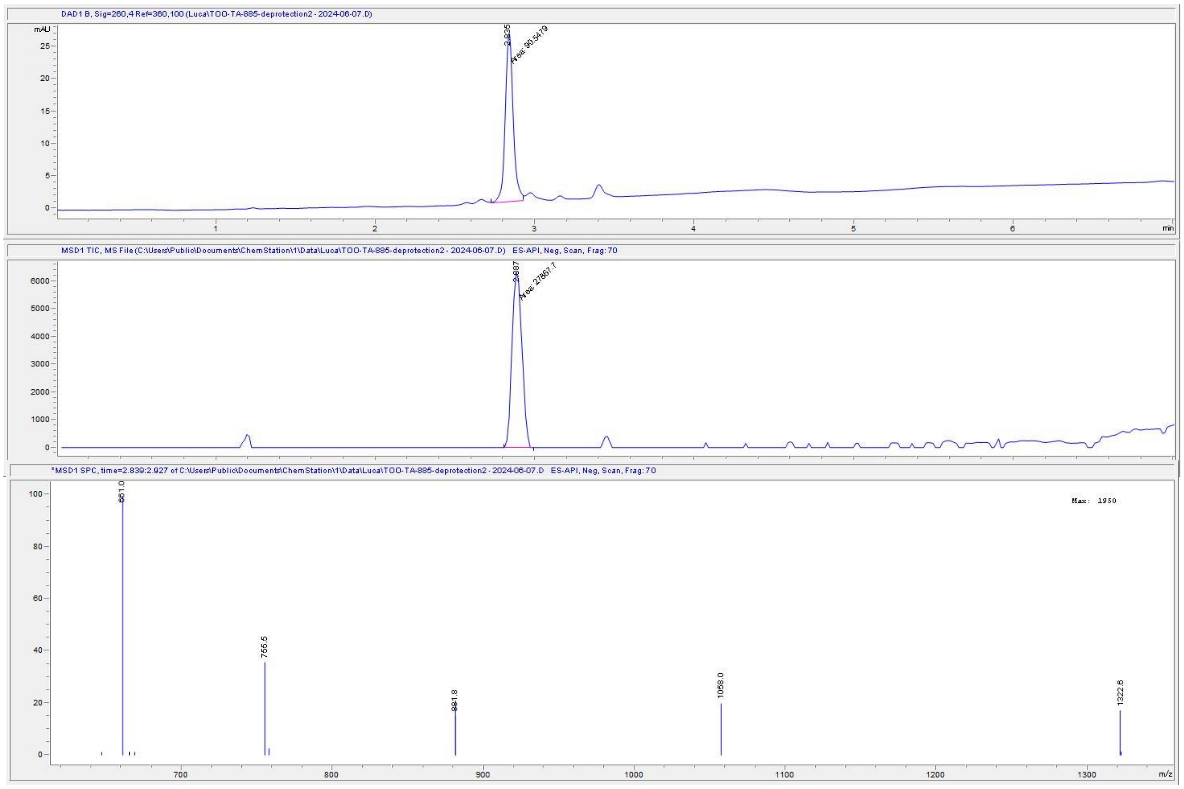

Figure S19. Analytical HPLC trace of **S28** with HPLC method B. (Top) DAD chromatogram at 260 nm. (Middle) TIC chromatogram. (Bottom) Ionization of peak at 2.89 min. containing reaction product.

DNA-conjugated substrate S29

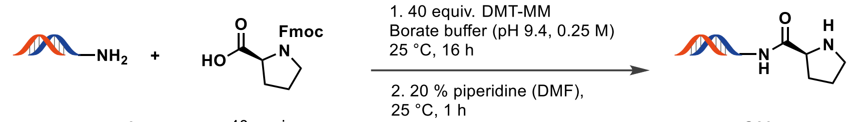

DNA-AOP-NH_2 (**2**) 40 equiv. (stock solution, **SA-16**) **S29**, >95%

Exact Mass: 5325.062
Molecular Weight: 5327.667

Preparation of *N*-Fmoc-*L*-proline stock solution (SA-16): Under an ambient atmosphere, a 1.5 mL Eppendorf tube was charged with *N*-Fmoc -*L*-proline (27 mg, 80 µmol). Next, 200 µL of DMA was added to the above 1.5 mL Eppendorf tube and the mixture was vortexed for 10 seconds. Next, the Eppendorf tube was sonicated for 10 seconds to ensure that the *N*-Fmoc -*L*-proline is completely dissolved, resulting in the formation of *N*-Fmoc -*L*-proline stock solution **SA-16** in DMA (c = 0.40 M).

Under an ambient atmosphere, the stock solution **SD-01** of DNA-AOP-NH_2 (**2**) in water (10 µL, c = 2.0 mM, 20 nmol, 1.0 equiv.) was added to a 1.5 mL Eppendorf tube, followed by sodium borate buffer (10 µL, pH 9.4, c = 0.50 M). Next, the stock solution of *N*-Fmoc -*L*-proline in DMA (**SA-16**, 2.0 µL, c = 0.40 M, 0.80 µmol, 40 equiv.) was added. The reaction mixture was vortexed for 5 seconds. Then, the stock solution of DMT-MM in water (**SR-02**, 2.0 µL, c = 0.40 M, 0.80 µmol, 40 equiv.) was added. The mixture was vortexed for 5 seconds, transferred to a thermocycler at 25 °C, and incubated for 16 hours at 600 rpm. After 16 hours, an aliquot of

1.0 µL of the reaction mixture was diluted to 40 µL with water for LC–MS analysis. Next, to the above reaction mixture was added the stock solution of NaCl in water (**SR-06**, 2.4 µL, c = 5.0 M, 10% volume of the total reaction volume), followed by cold ethanol (−20 °C, 80 µL) to precipitate the *N*-Fmoc proline DNA conjugate. The Eppendorf tube was placed in a freezer (−20 °C) for at least 1 hour, and then it was centrifuged at 4 °C and 11000 × g for at least 30 minutes. The supernatant was removed and the pellet was dried under air, then dissolved in 20 µL water (c = 1.0 mM).

Fmoc deprotection: The piperidine stock solution **SR-07** (20 µL, 20% v/v, in DMF) was added to the above 1.5 mL Eppendorf tube containing the *N*-Fmoc proline DNA conjugate (20 µL, c = 1.0 mM in water) and the resulting solution was vortexed for 5 seconds. The mixture was allowed to stand at 25 °C for 2 hours. Afterwards, an aliquot of 1 µL of the reaction mixture was diluted to 40 µL with water for LC–MS analysis. Next, to the above reaction mixture was added the stock solution of NaCl in water (**SR-06**, 4.0 µL, c = 5.0 M, 10% volume of the total reaction volume), followed by cold ethanol (−20 °C, 120 µL) to precipitate the DNA conjugate **S29**. The Eppendorf tube was placed in a freezer (−20 °C) for at least 1 hour and then it was centrifuged at 4 °C and 11000 × g for at least 30 minutes. The supernatant was decanted and the pellet was dried under air, then dissolved in 10 µL of water to obtain the DNA-conjugate **S29** (10 µL, c = 2.0 mM). The yield of the DNA conjugate was calculated by measuring the integration of the peaks of the diode array detection (DAD) UV absorbance at 260 nm of the LC–MS trace, assuming complete DNA recovery and identical UV absorbance.

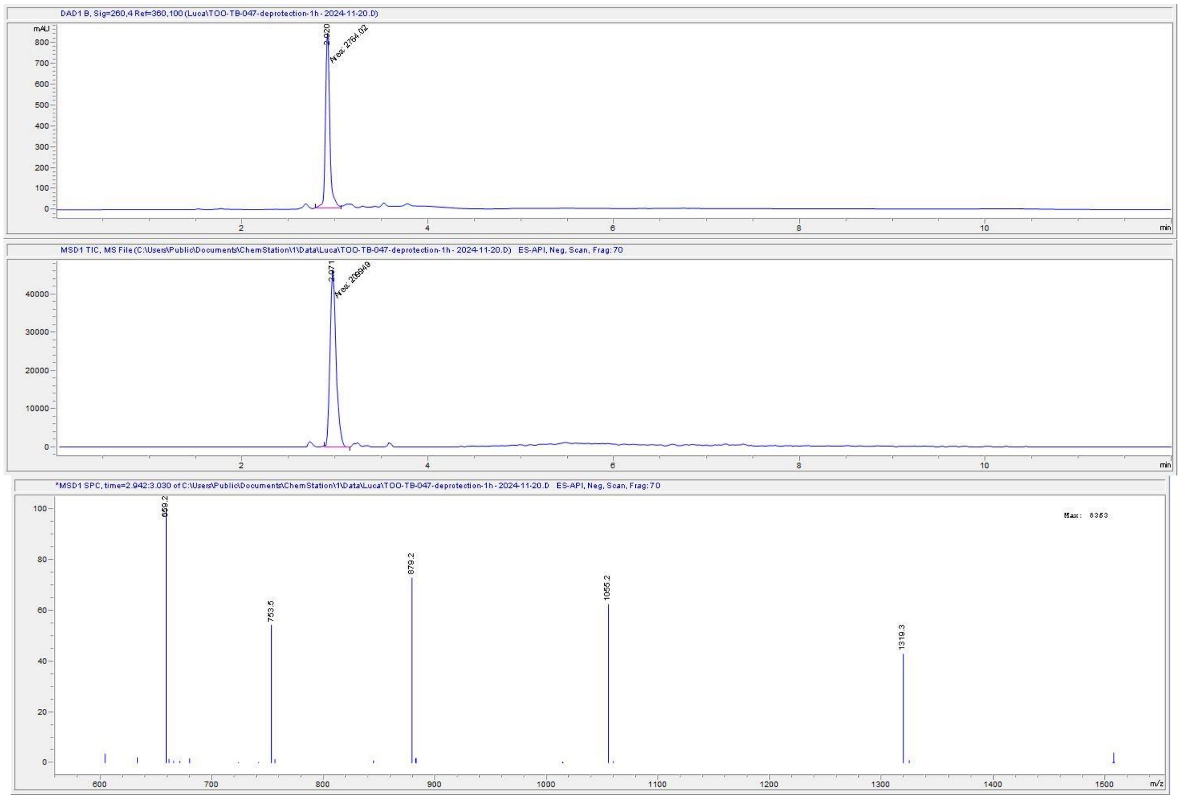

Figure S20. Analytical HPLC trace of **S29** with HPLC method A. (Top) DAD chromatogram at 260 nm. (Middle) TIC chromatogram. (Bottom) Ionization of peak at 3.07 min. containing reaction product.

DNA-conjugated substrate S30

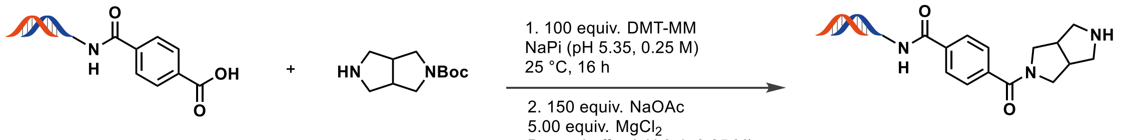

S19

Exact Mass: 5330.041
Molecular Weight: 5332.639

100 equiv.
(stock solution, **SA-17**)

S30, 80%

Exact Mass: 5424.130
Molecular Weight: 5426.800

Preparation of *tert*-butyl hexahydropyrrolo[3,4-*c*]pyrrole-2(*1H*)-carboxylate stock solution (SA-17): At 20–25 °C, a 1.5 mL Eppendorf tube was charged with *tert*-butyl hexahydropyrrolo[3,4-*c*]pyrrole-2(*1H*)-carboxylate stock solution (42.5 mg, 200 µmol). Next, 200 µL of DMA was added to the above 1.5 mL Eppendorf tube and the mixture was vortexed for 10 seconds. Next, the Eppendorf vial was sonicated for 10 seconds to make sure that the *N*-Boc protected amine is completely dissolved, resulting in the formation of *tert*-butyl piperidin-4-ylcarbamate stock solution in DMA (**SA-17**, c = 1.0 M).

Under an ambient atmosphere, the stock solution of DNA-conjugated carboxylic acid **S12** (10 µL, c = 2.0 mM, 20 nmol, 1.0 equiv.) in water was added to a 1.5 mL Eppendorf tube, followed by NaPi buffer (10 µL, pH 5.3, c = 0.50 M). Next, the stock solution of the amine in DMA (**SA-17**, 2.0 µL, c = 1.0 M, 2.0 µmol, 100 equiv.) was added. The reaction mixture was vortexed for 5 seconds. Then, the stock solution of DMT-MM in water (**SR-02**, 2.0 µL, c = 1.0 M, 2.0 µmol, 100 equiv.) was added. The mixture was vortexed for 5 seconds, transferred to a thermocycler at 25 °C, and incubated for 16 hours at 600 rpm. After 16 hours, an aliquot of 1.0 µL of the reaction mixture was diluted to 40 µL with water for LC–MS analysis. Next, to the above reaction mixture was added the stock solution of NaCl in water (**SR-06**, 2.4 µL, c = 5.0 M, 10% volume of the total reaction volume), followed by cold ethanol (−20 °C, 80 µL) to precipitate the DNA conjugate. The Eppendorf tube was placed in a freezer (−20 °C) for at least 1 hour, and then it was centrifuged at 4 °C and 11000 × g for at least 30 minutes. The supernatant was removed and the pellet was dried under air, then dissolved in 20 µL water (c = 1.0 mM).

Boc deprotection: To the above 1.5 mL Eppendorf tube containing the *N*-Boc amine DNA-conjugate (20 µL, c = 1.0 mM) in water, was added sodium borate buffer (100 µL, pH 9.4, c = 0.50 M), followed by stock solutions of NaOAc in water (**SR-05**, 40 µL, c = 75 mM) and $MgCl_2$ in water (**SR-04**, 40 µL, c = 2.5 mM). This resulted in the final reaction volume of 200 µL. The reaction mixture was then incubated in a thermocycler at 80 °C for 16–24 hours at 800 rpm. The progress of the reaction was monitored by LC–MS analysis. After the deprotection (approximately 24–48h), the reaction mixture was cooled to 23 °C and the mixture was then filtered using an Amicon ultra centrifugal filter (3 kDa MWCO). The retained DNA conjugate was dissolved in 8–10 µL of water to afford DNA conjugate **S30**. The DNA concentration was determined by measuring the A_{260} absorption using a Thermo Scientific™ NanoDrop™ One^{C}). The concentration was then adjusted by addition of Milli Q water to c = 2.0 mM. The yield of the DNA conjugate was calculated by measuring the integration of the peaks of the diode array detection (DAD) UV absorbance at 260 nm of the LC–MS trace, assuming complete DNA recovery and identical UV absorbance.

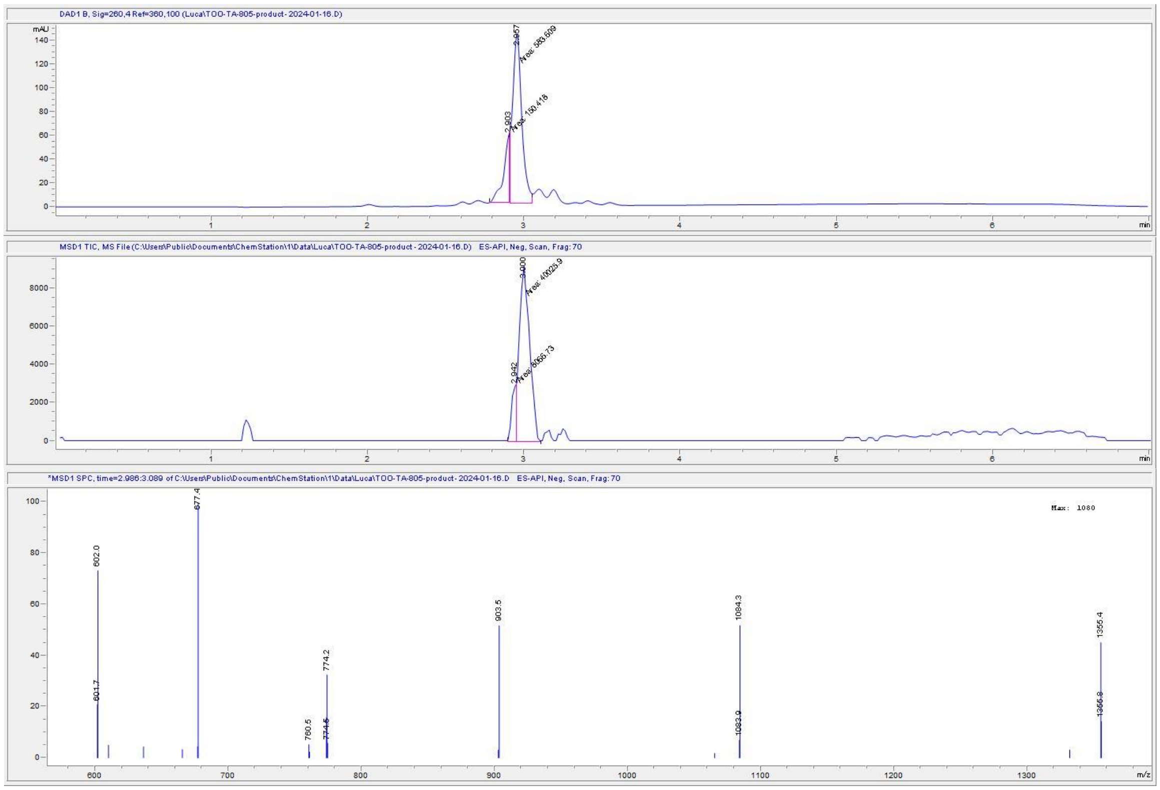

Figure S21. Analytical HPLC trace of **S30** with HPLC method B. (Top) DAD chromatogram at 260 nm. (Middle) TIC chromatogram. (Bottom) Ionization of peak at 3.00 min. containing reaction product.

ON-DNA *N*-ARYLATION

General protocol for the preparation of η⁶- ruthenium π-arene complex stock solutions (in situ)

Complexation method without acid:

It is important to note that the concentrations of the stock solutions were calculated based on the assumption that ruthenium complex **1** undergoes quantitative arene coordination to form the corresponding arene–ruthenium complexes *in situ*. This assumption eliminates the need for isolation or purification of the complexes, allowing them to be generated directly as stock solutions for subsequent use in the reactions.

Method A:

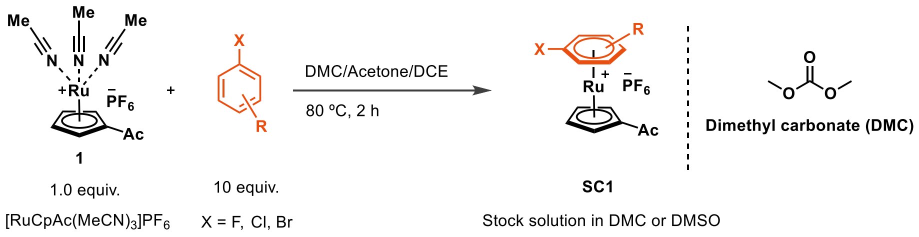

Under an ambient atmosphere, a 1 mL glass GC vial equipped with a 6 mm Teflon-coated stirring bar was charged with 50 µL stock solution of [RuCpAc(MeCN)$_3$]PF$_6$ (**1**, c = 40 mM, 1.0 equiv.) in dimethylcarbonate (DMC)/Acetone/DCE (1,2-dichloroethane) and mixed with 50 µL stock solution of aryl halide (c = 0.40 M, 10 equiv.) in DMC/Acetone/DCE. The vial was closed with a screw and the resulting reaction mixture was stirred at 80 °C for 2 hours. Subsequently, DMC/Acetone/DCE was removed under a gentle flow of argon and the residue was dissolved in 100 µL DMSO to obtain a stock solution of the arene ruthenium complex (c = 0.02 M, 100 µL, assuming quantitative arene coordination to ruthenium) in DMSO. The resulting stock solution was then used directly for performing on-DNA reactions without purification.

Note: Complex stock solution after the reaction in DMC (c = 0.02 M, 100 µL, assuming quantitative arene coordination to ruthenium), can also be used directly for performing on-DNA reaction without any purification.

Method B:

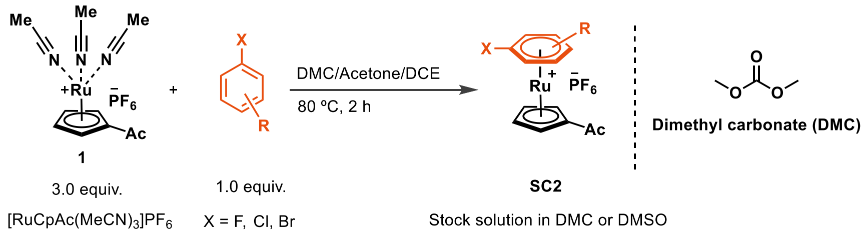

Under an ambient atmosphere, a 1 mL glass GC vial equipped with a 6 mm Teflon-coated stirring bar was charged with 50 µL stock solution of [RuCpAc(MeCN)$_3$]PF$_6$ (**1**, c = 60 mM, 3.0 equiv.) in DMC/Acetone/DCE and mixed with 50 µL stock solution of aryl halide (c = 20 mM, 1.0 equiv.) DMC/Acetone/DCE. The vial was closed with a screw cap and the resulting reaction mixture was stirred at 80 °C for 2 hours. Subsequently,

DMC/Acetone/DCE was removed under a gentle stream of argon and the residue was dissolved in 100 µL DMSO to obtain the stock solution of the arene ruthenium complex (c = 0.01 M, 100 µL, assuming quantitative arene coordination to ruthenium) in DMSO. The resulting stock solution was then used directly for performing on-DNA reactions without purification.

Complexation method with acid:

Method C:

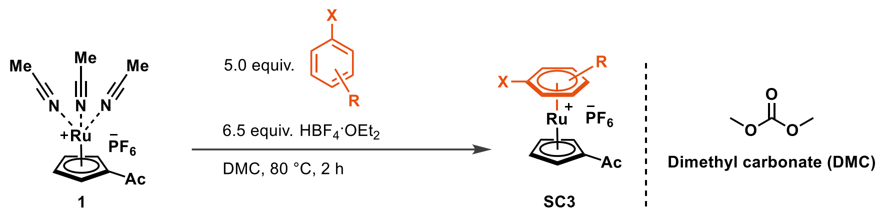

[RuCpAc(MeCN)$_3$]PF$_6$ Stock solution in DMC or DMSO

Under an ambient atmosphere, a 1 mL glass GC vial equipped with a 6 mm Teflon-coated stirring bar was charged with 25 µL stock solution of aryl halide (c = 0.20 M, 5.0 equiv.) in DMC and mixed with 25 µL stock solution of $HBF_4{\cdot}OEt_2$ (c = 0.26 M, 6.5 equiv.) in DMC. To this mixture was added 50 µL stock solution of [RuCpAc(MeCN)$_3$]PF$_6$ (**1**, c = 20 mM, 1.0 equiv.) in DMC. The vial was closed with a screw cap and the resulting reaction mixture was stirred at 80 °C for 2 hours. Subsequently, DMC was removed under a gentle stream of argon and the residue was dissolved in 100 µL DMSO to obtain the stock solution of arene ruthenium complex (c = 0.01 M, 100 µL, assuming quantitative arene coordination to ruthenium) in DMSO. The resulting stock solution was then used directly for performing on-DNA reactions without any purification.

Method D:

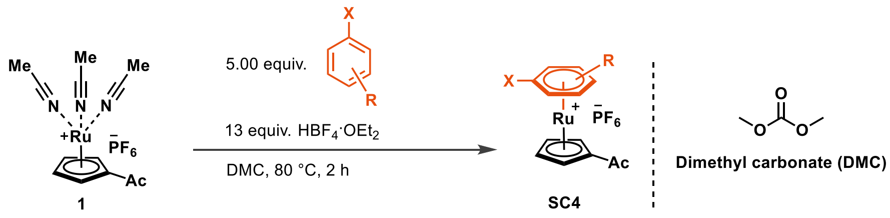

[RuCpAc(MeCN)$_3$]PF$_6$ Stock solution in DMC or DMSO

Under ambient atmosphere, a 1 mL glass GC vial equipped with a 6 mm Teflon-coated stirring bar was charged with 25 µL stock solution of aryl halide (c = 0.40 M, 5.0 equiv.) in DMC and mixed with 25 µL stock solution of $HBF_4{\cdot}OEt_2$ (c = 1.0 M, 13 equiv.) in DMC. To this reaction mixture was added 50 µL stock solution of [RuCpAc(MeCN)$_3$]PF$_6$ (**1**, c = 40 mM, 1.0 equiv.) in DMC. The vial was closed and the resulting reaction mixture was stirred at 80 °C for 2 hours. Subsequently, DMC was removed under a gentle stream of argon and the residue was dissolved in 100 µL DMSO to obtain the stock solution of arene ruthenium complex (c = 0.02 M, 100 µL, assuming quantitative arene coordination to ruthenium) in DMSO. The resulting stock solution was then used directly for performing on-DNA reactions without any purification.

Note: Complex stock solution after the reaction in DMC (c = 0.02 M, 100 µL, assuming quantitative arene coordination to ruthenium), can also be used directly for performing on-DNA reaction without any purification.

Preparation of $HBF_4 \cdot OEt_2$ stock solution: Under an ambient atmosphere, a 4 mL borosilicate vial was charged with $HBF_4 \cdot OEt_2$ (0.50 mL, 0.60 g, 3.7 mmol, 50–55% w/w). Next, 1.5 mL of DMC was added to the vial and the mixture was vortexed for 10 seconds, resulting in an $HBF_4 \cdot OEt_2$ stock solution in DMC (c = 1.0 M).

N-arylation of haloarenes with DNA-AOP-NH_2 (2)

Synthesis of DNA-conjugate 15 (from fluorobenzene)

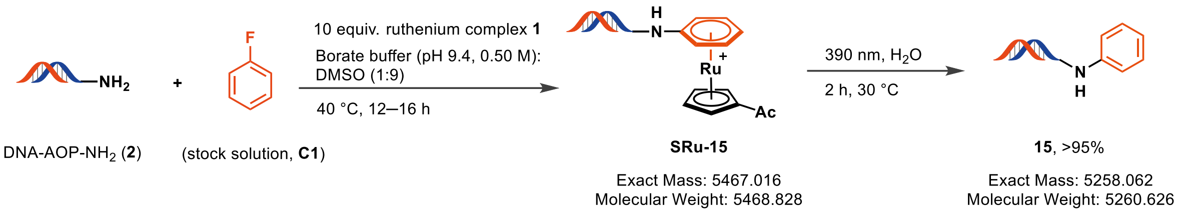

In situ formation of arene-ruthenium stock solution: Under an ambient atmosphere, a 1 mL glass GC vial equipped with a 6 mm Teflon-coated stirring bar was charged with ruthenium complex **1** (1.4 mg, 2.9 µmol, 1.0 equiv.). Next, a stock solution of fluorobenzene in DMC (294 µL, c = 0.10 M, 29 µmol, 10 equiv.) was added. The resulting reaction mixture was heated at 80 °C for 2 hours. After 2 hours, the reaction mixture was cooled to 23 °C. Next, the DMC was removed under a gentle stream of argon and 294 µL of DMSO were added to result an in situ formed stock solution of arene-ruthenium complex **C1** (294 µL, c = 0.01 M, assuming quantitative arene coordination to ruthenium).

Under an ambient atmosphere, the stock solution **SD-01** of DNA-AOP-NH_2 (**2**) in water (1.0 µL, c = 2.0 mM, 2.0 nmol, 1.0 equiv.) was added to a 1.5 mL Eppendorf tube, followed by sodium borate buffer (1.0 µL, pH 9.4, c = 0.50 M). To this mixture, 16 µL of DMSO was added and the solution was vortexed for 5 seconds. Next, the freshly prepared stock solution **C1** (2.0 µL, c = 0.01 M, 0.02 µmol, 10 equiv.) in DMSO was added. The resulting reaction mixture was vortexed for 5 seconds, transferred to a thermocycler at 40 °C, and incubated for 16 hours at 800 rpm to yield the DNA-conjugate **SRu-15**. Next, the reaction mixture was diluted with 10 µL of Milli-Q water. To the reaction mixture was added the stock solution of NaCl in water (**SR-06** , 3.0 µL, c = 5.0 M, 10% volume of the total reaction volume), followed by cold ethanol (−20 °C, 99 µL) to precipitate the *N*-arylated ruthenium DNA conjugate **SRu-15**. The Eppendorf tube was placed in a freezer (−20 °C) for at least 1 hour, and then it was centrifuged at 4 °C and 11000 × g for at least 30 minutes. The supernatant was removed and the pellet was dried under air, then dissolved in 20 µL water to obtain the DNA-conjugate **SRu-15** (20 µL, c = 0.10 mM). Then, 1.0 µL of the above solution was diluted to 40 µL with water for LC–MS analysis.

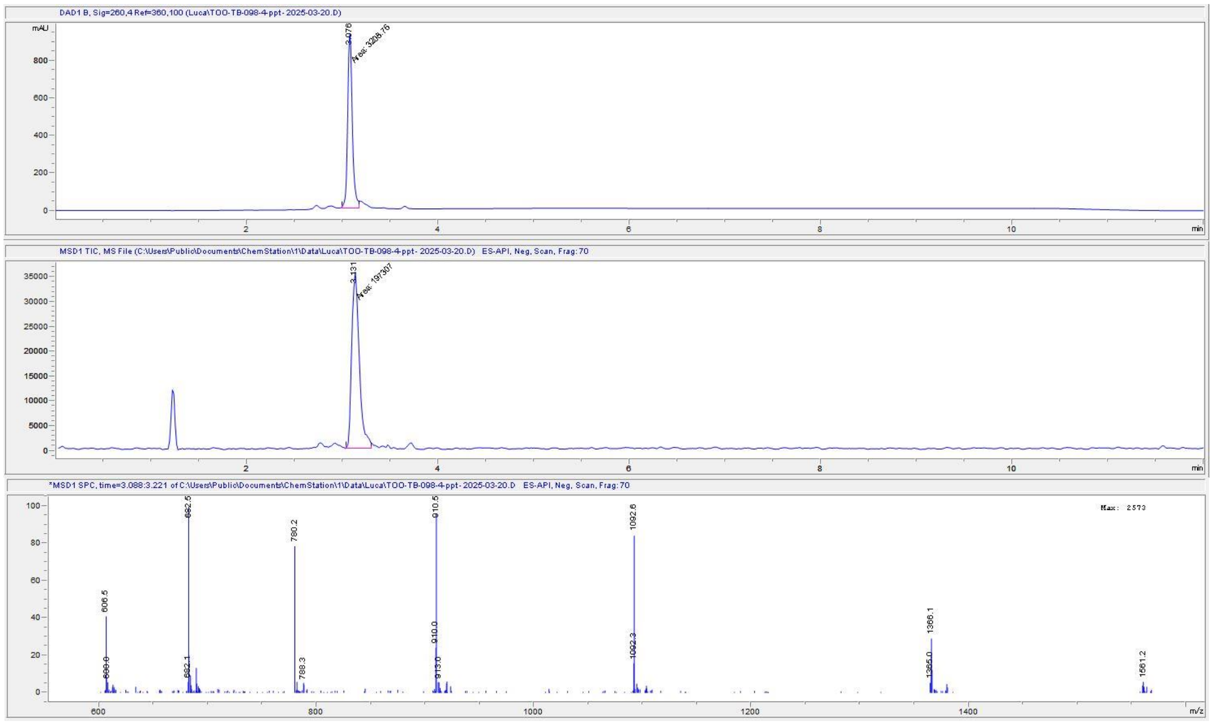

Figure S22. Analytical HPLC trace of **SRu-15** with HPLC method A. (Top) DAD chromatogram at 260 nm. (Middle) TIC chromatogram. (Bottom) Ionization of peak at 3.13 min containing reaction product.

Decomplexation of SRu-15 to obtain product 15: Under an ambient atmosphere, the DNA-conjugate **SRu-15** stock solution in water (c = 0.10 mM, 20 µL) was irradiated with a 390 nm (40 W) Kessil lamp for 2 hours, while maintaining the temperature at approximately 30 °C through cooling with a fan. To the reaction mixture was added the stock solution of NaCl in water (**SR-06**, 2.0 µL, c = 5.0 M, 10% volume of the total reaction volume), followed by cold ethanol (−20 °C, 66 µL) to precipitate the DNA conjugate **15**. The Eppendorf tube was placed in the freezer (−20 °C) for at least 1 hour, and then it was centrifuged at 4 °C and 11000 × g for at least 30 minutes. The supernatant was removed, the pellet dried under air and dissolved in Milli-Q water to obtain the purified DNA-conjugate **15**. Then, 1 µL of the above solution was diluted to 40 µL with water for LC–MS analysis. The yield of the DNA conjugate was calculated by measuring the integration of the peaks of the diode array detection (DAD) UV absorbance at 260 nm of the LC–MS trace, assuming complete DNA recovery and identical UV absorbance.

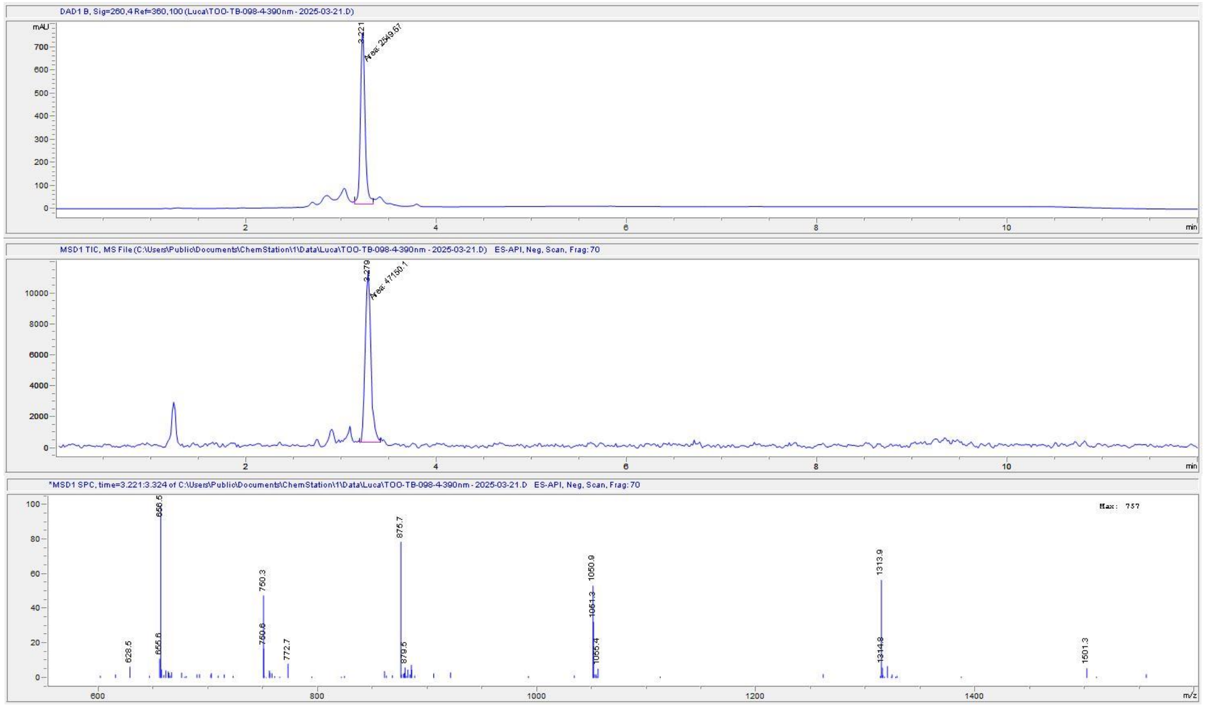

Figure S23. Analytical HPLC trace of **15** with HPLC method A. (Top) DAD chromatogram at 260 nm. (Middle) TIC chromatogram. (Bottom) Ionization of peak at 3.28 min. containing reaction product.

Synthesis of DNA-conjugate 15 (from chlorobenzene)

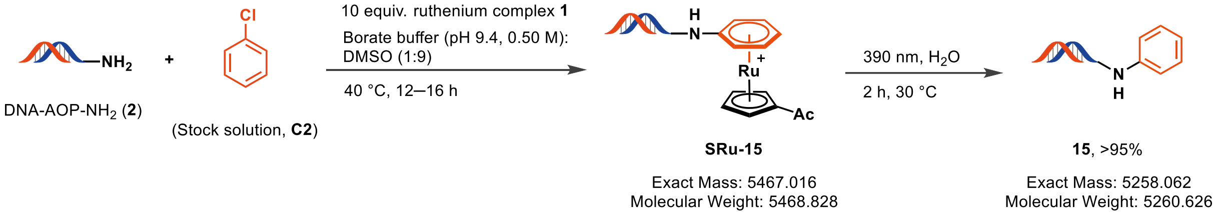

In situ formation of arene-ruthenium stock solution: Under an ambient atmosphere, a 1 mL glass GC vial equipped with a 6 mm Teflon-coated stirring bar was charged with ruthenium complex **1** (1.4 mg, 2.9 µmol, 1.0 equiv.). Next, a stock solution of chlorobenzene in DMC (294 µL, c = 0.10 M, 10 equiv.) was added. The resulting reaction mixture was heated at 80 °C for 2 hours. After 2 hours, the reaction mixture was cooled to 23 °C. Next, the DMC was removed under a gentle stream of argon and 294 µL of DMSO were added to result an in situ formed stock solution of arene-ruthenium complex **C2** (294 µL, c = 0.01 M, assuming quantitative arene coordination to ruthenium).

Under an ambient atmosphere, the stock solution **SD-01** of DNA-AOP-NH_2 (**2**) in water (1.0 µL, c = 2.0 mM, 2.0 nmol, 1.0 equiv.) was added to a 1.5 mL Eppendorf tube, followed by sodium borate buffer (1.0 µL, pH 9.4, c = 0.50 M). To this mixture, 16 µL of DMSO was added and the solution was vortexed for 5 seconds. Next, the freshly prepared stock solution **C2** (2.0 µL, c = 0.01 M, 0.02 µmol, 10 equiv.) in DMSO was added. The resulting reaction mixture was vortexed for 5 seconds, transferred to a thermocycler at 40 °C, and incubated for 16 hours at 800 rpm to yield the DNA-conjugate **SRu-15**. Next, the reaction mixture was diluted

with 10 µL of Milli-Q water. To the reaction mixture was added the stock solution of NaCl in water (**SR-06** , 3.0 µL, c = 5.0 M, 10% volume of the total reaction volume), followed by cold ethanol (−20 °C, 99 µL) to precipitate the *N*-arylated ruthenium DNA conjugate **SRu-15**. The Eppendorf tube was placed in a freezer (−20 °C) for at least 1 hour, and then it was centrifuged at 4 °C and 11000 × g for at least 30 minutes. The supernatant was removed and the pellet was dried under air, then dissolved in 20 µL water to obtain the DNA-conjugate **SRu-15** (20 µL, c = 0.10 mM). Then, 1.0 µL of the above solution was diluted to 40 µL with water for LC–MS analysis.

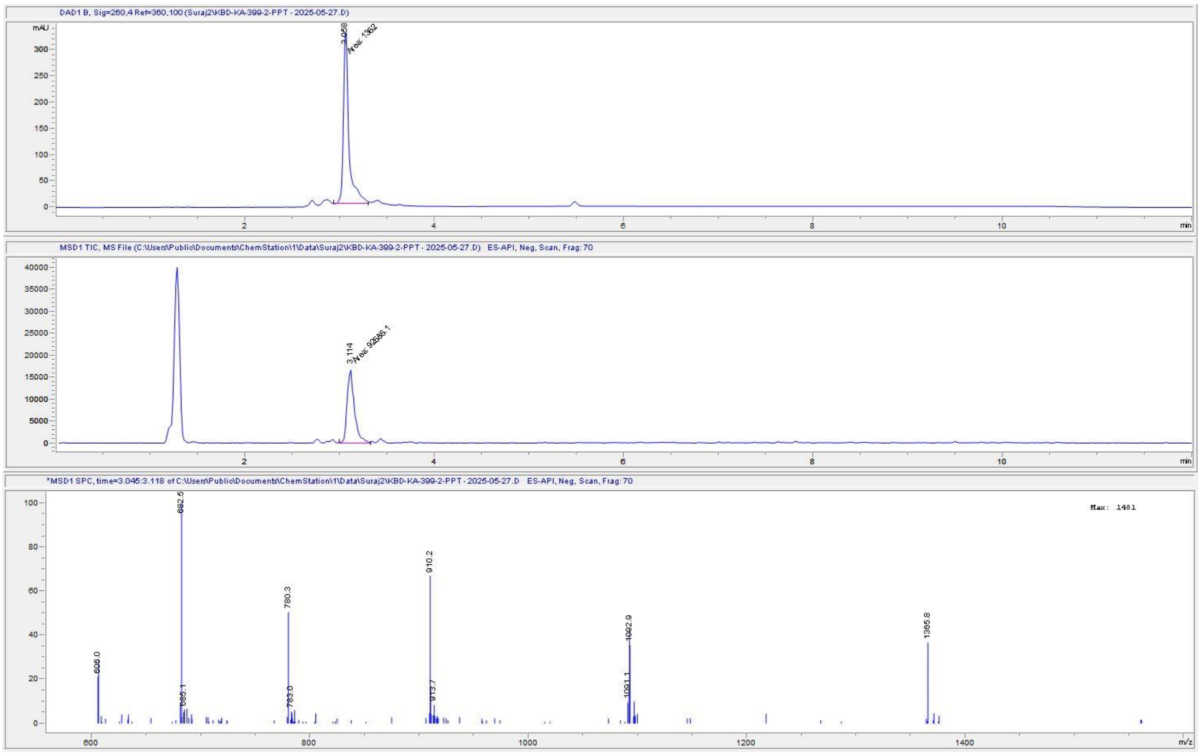

Figure S24. Analytical HPLC trace of **SRu-15** with HPLC method A. (Top) DAD chromatogram at 260 nm. (Middle) TIC chromatogram. (Bottom) Ionization of peak at 3.11 min containing reaction product.

Decomplexation of SRu-15 to obtain product 15: Under an ambient atmosphere, the DNA-conjugate **SRu-15** stock solution in water (c = 0.10 mM, 20 µL) was irradiated with a 390 nm (40 W) Kessil lamp for 2 hours, while maintaining the temperature at approximately 30 °C through cooling with a fan. To the reaction mixture was added the stock solution of NaCl in water (**SR-06**, 2.0 µL, c = 5.0 M, 10% volume of the total reaction volume), followed by cold ethanol (−20 °C, 66 µL) to precipitate the DNA conjugate **15**. The Eppendorf tube was placed in the freezer (−20 °C) for at least 1 hour, and then it was centrifuged at 4 °C and 11000 × g for at least 30 minutes. The supernatant was removed, the pellet dried under air and dissolved in Milli-Q water to obtain the purified DNA-conjugate **15**. Then, 1 µL of the above solution was diluted to 40 µL with water for LC–MS analysis. The yield of the DNA conjugate was calculated by measuring the integration of the peaks of the diode array detection (DAD) UV absorbance at 260 nm of the LC–MS trace, assuming complete DNA recovery and identical UV absorbance.

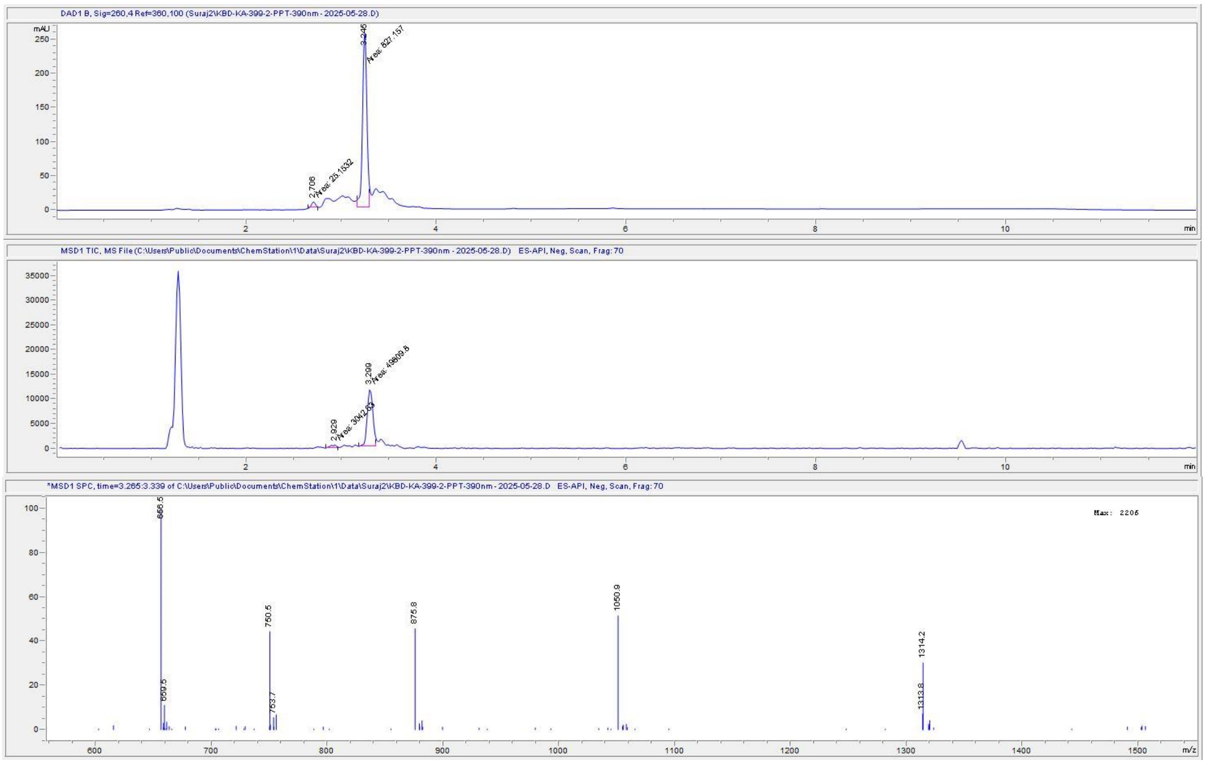

Figure S25. Analytical HPLC trace of **15** with HPLC method A. (Top) DAD chromatogram at 260 nm. (Middle) TIC chromatogram. (Bottom) Ionization of peak at 3.30 min. containing reaction product.

Synthesis of DNA-conjugate 15 (from bromobenzene)

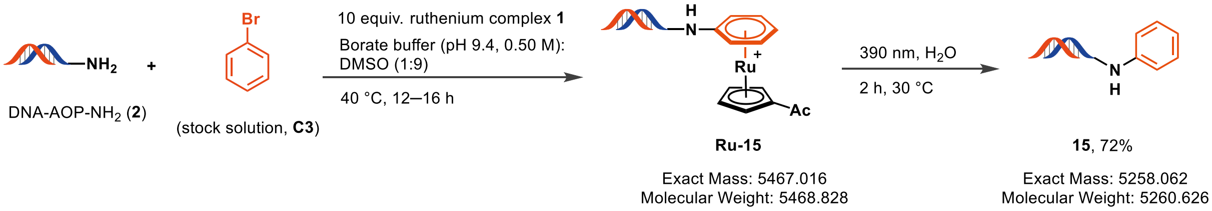

In situ formation of arene-ruthenium stock solution: Under an ambient atmosphere, a 1 mL glass GC vial equipped with a 6 mm Teflon-coated stirring bar was charged with ruthenium complex **1** (1.4 mg, 2.9 µmol, 1.0 equiv.). Next, a stock solution of bromobenzene in DMC (294 µL, c = 0.10 M, 29 µmol, 10 equiv.) was added. The vial was closed with a screw cap and the resulting reaction mixture was heated at 80 °C for 2 hours. After 2 hours, the reaction mixture was cooled to 23 °C. Next, the DMC was removed under a gentle stream of argon and 294 µL of DMSO were added to result an in situ formed stock solution of arene-ruthenium complex **C3** (294 µL, c = 0.01 M, assuming quantitative arene coordination to ruthenium).

Under an ambient atmosphere, the stock solution **SD-01** of DNA-AOP-NH_2 (**2**) in water (1.0 µL, c = 2.0 mM, 2.0 nmol, 1.0 equiv.) was added to a 1.5 mL Eppendorf tube, followed by sodium borate buffer (1.0 µL, pH 9, c = 0.50 M). To this mixture, 16 µL of DMSO was added and the solution was vortexed for 5 seconds. Next, the freshly prepared stock solution **C3** (2.0 µL, c = 0.01 M, 0.02 µmol, 10 equiv.) in DMSO was added. The resulting reaction mixture was vortexed for 5 seconds, transferred to a thermocycler at 40 °C, and incubated

for 16 hours at 800 rpm to yield the DNA-conjugate **SRu-15**. Next, the reaction mixture was diluted with 10 µL of Milli-Q water. To the reaction mixture was added the stock solution of NaCl in water (**SR-06** , 3.0 µL, c = 5.0 M, 10% volume of the total reaction volume), followed by cold ethanol (−20 °C, 99 µL) to precipitate the *N*-arylated ruthenium DNA conjugate **SRu-15**. The Eppendorf tube was placed in a freezer (−20 °C) for at least 1 hour, and then it was centrifuged at 4 °C and 11000 × g for at least 30 minutes. The supernatant was removed and the pellet was dried under air, then dissolved in 20 µL water to obtain the DNA-conjugate **SRu-15** (20 µL, c = 0.10 mM). Then, 1.0 µL of the above solution was diluted to 40 µL with water for LC–MS analysis. It is important to note that, in the case of bromobenzene-ruthenium complex bi-arylation as the side product was observed.

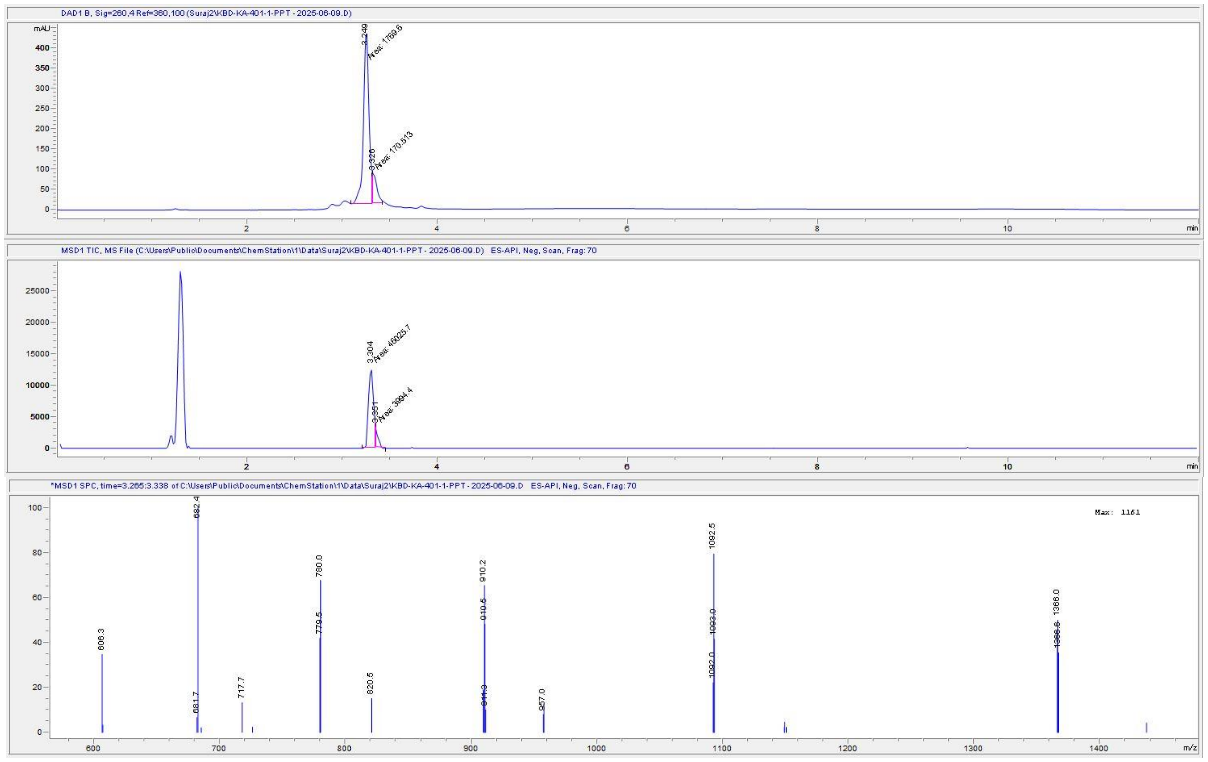

Figure S26. Analytical HPLC trace of **Ru-15** with HPLC method A. (Top) DAD chromatogram at 260 nm. (Middle) TIC chromatogram. (Bottom) Ionization of peak at 3.30 min containing reaction product.

Decomplexation of SRu-15 to obtain product 15: Under an ambient atmosphere, the DNA-conjugate **SRu-15** stock solution in water (c = 0.10 mM, 20 µL) was irradiated with a 390 nm (40 W) Kessil lamp for 2 hours, while maintaining the temperature at approximately 30 °C through cooling with a fan. To the reaction mixture was added the stock solution of NaCl in water (**SR-06**, 2.0 µL, c = 5.0 M, 10% volume of the total reaction volume), followed by cold ethanol (−20 °C, 66 µL) to precipitate the DNA conjugate **15**. The Eppendorf tube was placed in the freezer (−20 °C) for at least 1 hour, and then it was centrifuged at 4 °C and 11000 × g for at least 30 minutes. The supernatant was removed, the pellet dried under air and dissolved in Milli-Q water to obtain the purified DNA-conjugate **15**. Then, 1 µL of the above solution was diluted to 40 µL with water for LC–MS analysis. The yield of the DNA conjugate was calculated by measuring the integration of the peaks of

the diode array detection (DAD) UV absorbance at 260 nm of the LC–MS trace, assuming complete DNA recovery and identical UV absorbance.

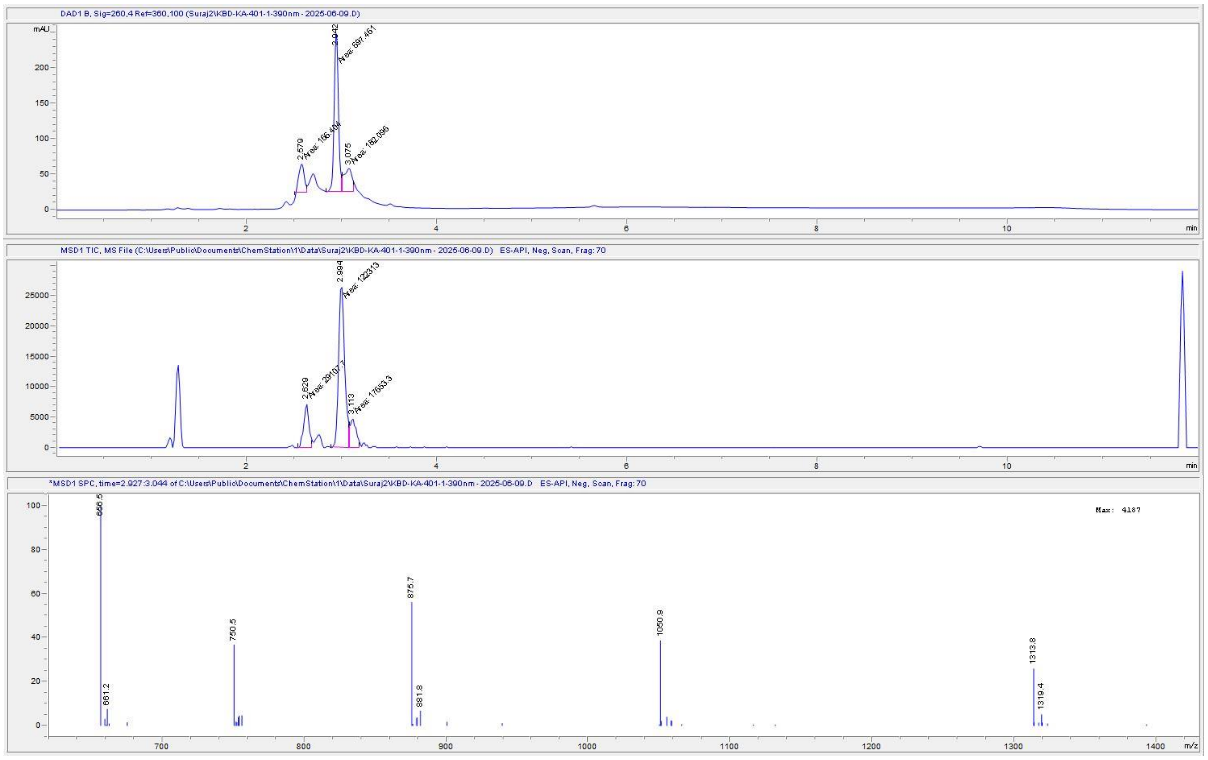

Figure S27. Analytical HPLC trace of **15** with HPLC method A. (Top) DAD chromatogram at 260 nm. (Middle) TIC chromatogram. (Bottom) Ionization of peak at 2.99 min. containing reaction product.

Synthesis of DNA-conjugate 15 (from iodobenzene)

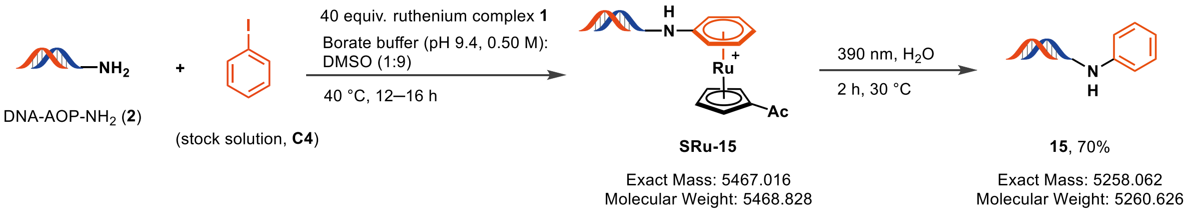

In situ formation of arene-ruthenium stock solution: Under an ambient atmosphere, a 1 mL glass GC vial equipped with a 6 mm Teflon-coated stirring bar was charged with ruthenium complex **1** (1.4 mg, 2.9 µmol, 1.0 equiv.). Next, a stock solution of iodobenzene in DMC (294 µL, c = 0.10 M, 29 µmol, 10 equiv.) was added. The resulting reaction mixture was heated at 80 °C for 2 hours. After 2 hours, the reaction mixture was cooled to 23 °C. Next, the DMC was removed under a gentle stream of argon and 294 µL of DMSO were added to result an in situ formed stock solution of arene-ruthenium complex **C4** (294 µL, c = 0.01 M, assuming quantitative arene coordination to ruthenium).

Under an ambient atmosphere, the stock solution **SD-01** of DNA-AOP-NH2 (**2**) in water (1.0 µL, c = 2.0 mM, 2.0 nmol, 1.0 equiv.) was added to a 1.5 mL Eppendorf tube, followed by sodium borate buffer (1.0 µL, pH

9.4, c = 0.50 M). To this mixture, 10 µL of DMSO was added and the solution was vortexed for 5 seconds. Next, the freshly prepared stock solution **C4** (8.0 µL, c = 0.01 M, 0.08 µmol, 40 equiv.) in DMSO was added. The resulting reaction mixture was vortexed for 5 seconds, transferred to a thermocycler at 40 °C, and incubated for 16 hours at 800 rpm to yield the DNA-conjugate **SRu-15**. Next, the reaction mixture was diluted with 10 µL of Milli-Q water. To the reaction mixture was added the stock solution of NaCl in water (**SR-06** , 3.0 µL, c = 5.0 M, 10% volume of the total reaction volume), followed by cold ethanol (−20 °C, 99 µL) to precipitate the *N*-arylated ruthenium DNA conjugate **SRu-15**. The Eppendorf tube was placed in a freezer (−20 °C) for at least 1 hour, and then it was centrifuged at 4 °C and 11000 × g for at least 30 minutes. The supernatant was removed and the pellet was dried under air, then dissolved in 20 µL water to obtain the DNA-conjugate **SRu-15** (20 µL, c = 0.10 mM). Then, 1.0 µL of the above solution was diluted to 40 µL with water for LC–MS analysis.

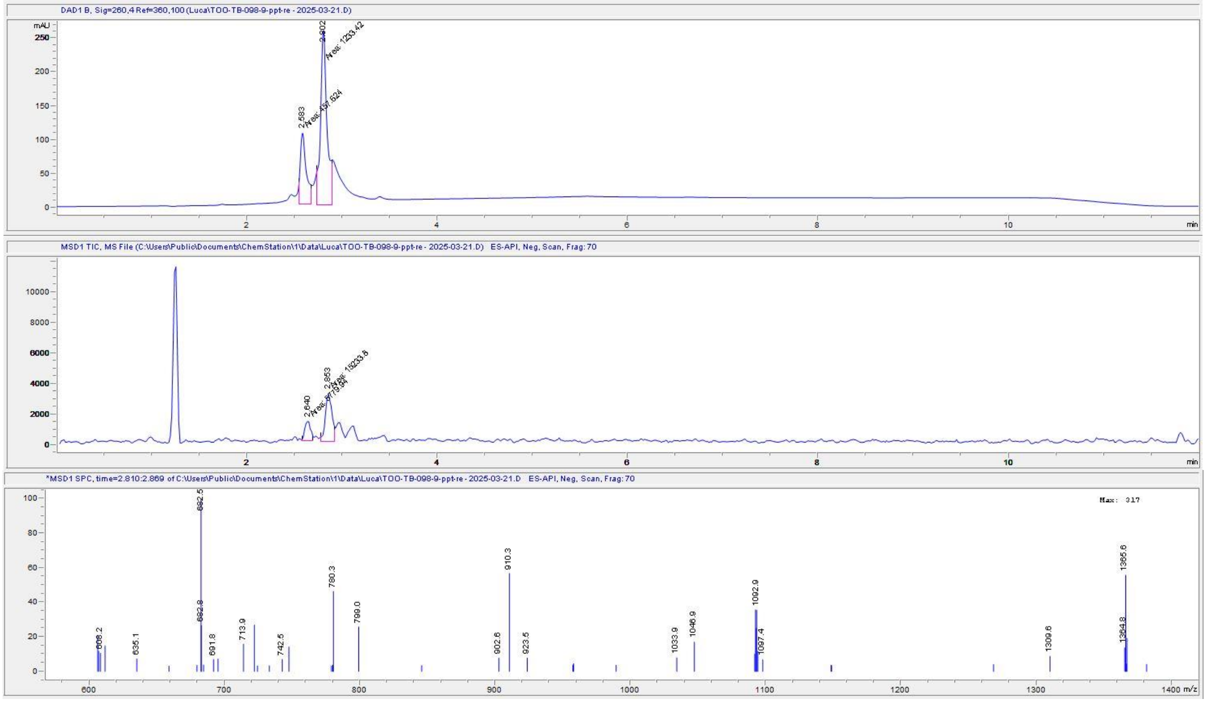

Figure S28. Analytical HPLC trace of **Ru-15** with HPLC method A. (Top) DAD chromatogram at 260 nm. (Middle) TIC chromatogram. (Bottom) Ionization of peak at 2.85 min containing reaction product.

Decomplexation of SRu-15 to obtain product 15: Under an ambient atmosphere, the DNA-conjugate **SRu-15** stock solution in water (c = 0.10 mM, 20 µL) was irradiated with a 390 nm (40 W) Kessil lamp for 2 hours, while maintaining the temperature at approximately 30 °C through cooling with a fan. To the reaction mixture was added the stock solution of NaCl in water (**SR-06**, 2.0 µL, c = 5.0 M, 10% volume of the total reaction volume), followed by cold ethanol (−20 °C, 66 µL) to precipitate the DNA conjugate 15. The Eppendorf tube was placed in the freezer (−20 °C) for at least 1 hour, and then it was centrifuged at 4 °C and 11000 × g for at least 30 minutes. The supernatant was removed, the pellet dried under air and dissolved in Milli-Q water to obtain the purified DNA-conjugate 15. Then, 1 µL of the above solution was diluted to 40 µL with water for

LC–MS analysis. The yield of the DNA conjugate was calculated by measuring the integration of the peaks of the diode array detection (DAD) UV absorbance at 260 nm of the LC–MS trace, assuming complete DNA recovery and identical UV absorbance.

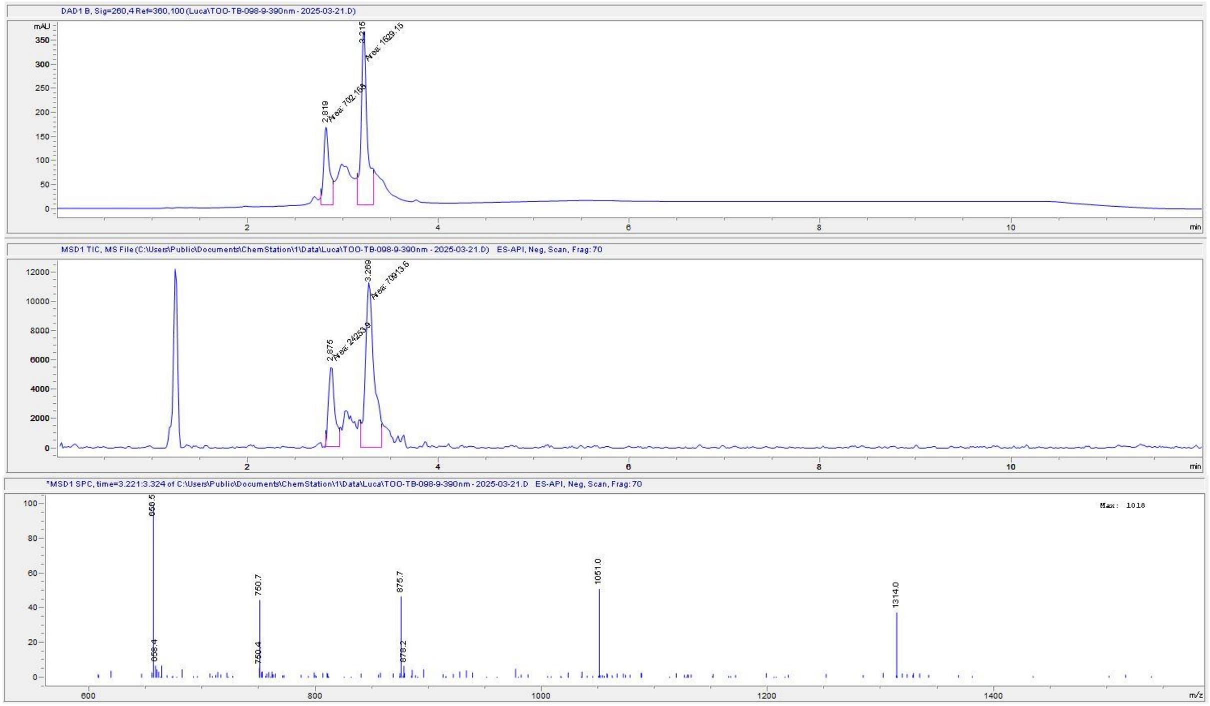

Figure S29. Analytical HPLC trace of **15** with HPLC method A. (Top) DAD chromatogram at 260 nm. (Middle) TIC chromatogram. (Bottom) Ionization of peak at 3.27 min. containing reaction product.

Synthesis of DNA-conjugate 17

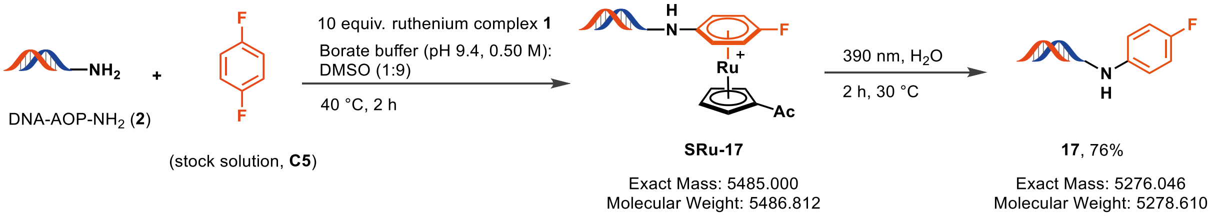

In situ formation of arene-ruthenium stock solution: Under an ambient atmosphere, a 1 mL glass GC vial equipped with a 6 mm Teflon-coated stirring bar was charged with ruthenium complex **1** (1.4 mg, 2.9 µmol, 1.0 equiv.). Next, a stock solution of 1,4-difluorobenzene in DMC (294 µL, c = 0.10 M, 29 µmol, 10 equiv.) was added. The resulting reaction mixture was heated at 80 °C for 2 hours. After 2 hours, the reaction mixture was cooled to 23 °C. Next, the DMC was removed under a gentle stream of argon and 294 µL of DMSO were added to result an in situ formed stock solution of arene-ruthenium complex **C5** (294 µL, c = 0.01 M, assuming quantitative arene coordination to ruthenium).

Under an ambient atmosphere, the stock solution **SD-01** of DNA-AOP-NH_2 (**2**) in water (1.0 µL, c = 2.0 mM, 2.0 nmol, 1.0 equiv.) was added to a 1.5 mL Eppendorf tube, followed by sodium borate buffer (1.0 µL, pH

9.4, c = 0.50 M). To this mixture, 16 µL of DMSO was added and the solution was vortexed for 5 seconds. Next, the freshly prepared stock solution **C5** (2.0 µL, c = 0.01 M, 0.02 µmol, 10 equiv.) in DMSO was added. The resulting reaction mixture was vortexed for 5 seconds, transferred to a thermocycler at 40 °C, and incubated for 2 hours at 800 rpm to yield the DNA-conjugate **SRu-17**. Next, the reaction mixture was diluted with 10 µL of Milli-Q water. To the reaction mixture was added the stock solution of NaCl in water (**SR-06** , 3.0 µL, c = 5.0 M, 10% volume of the total reaction volume), followed by cold ethanol (−20 °C, 99 µL) to precipitate the *N*-arylated ruthenium DNA conjugate **SRu-17**. The Eppendorf tube was placed in a freezer (−20 °C) for at least 1 hour, and then it was centrifuged at 4 °C and 11000 × g for at least 30 minutes. The supernatant was removed and the pellet was dried under air, then dissolved in 20 µL water to obtain the DNA-conjugate **SRu-17** (20 µL, c = 0.10 mM). Then, 1.0 µL of the above solution was diluted to 40 µL with water for LC–MS analysis.

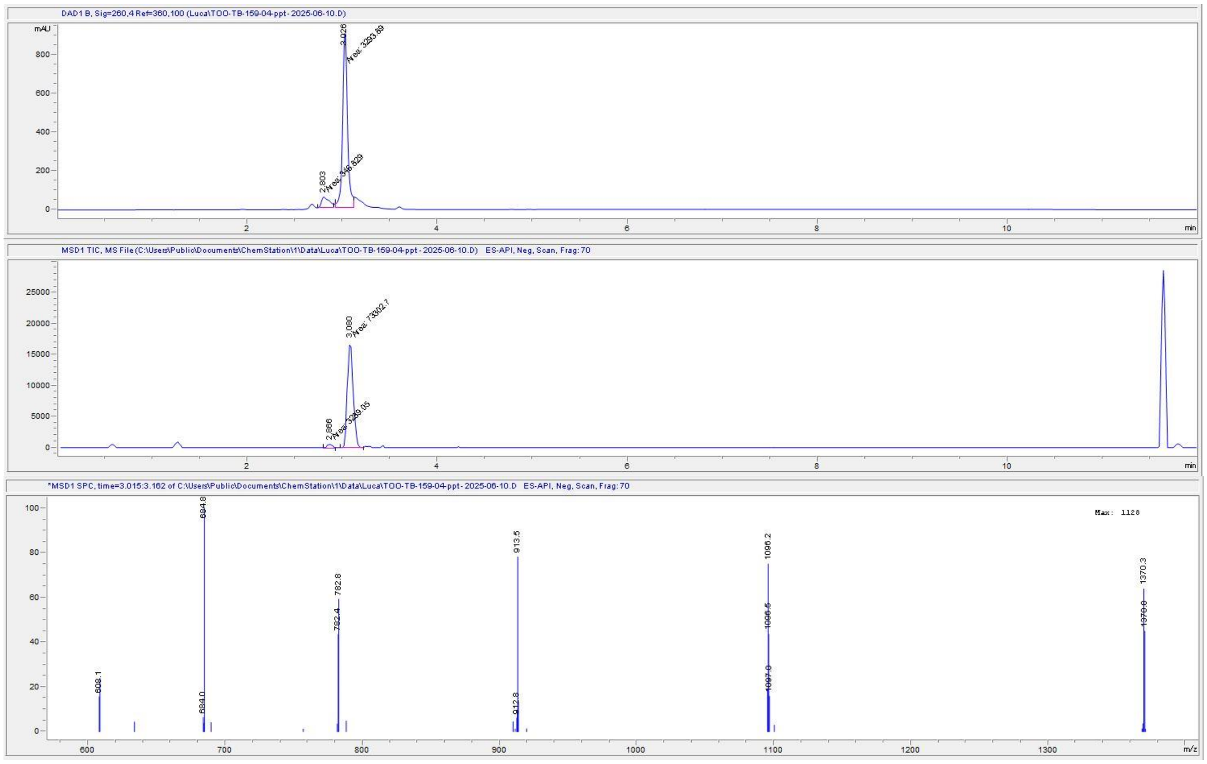

Figure S30. Analytical HPLC trace of **Ru-17** with HPLC method A. (Up) DAD chromatogram at 260 nm. (Middle) TIC chromatogram. (Bottom) Ionization of peak at 3.08 min containing reaction product.

Decomplexation of SRu-17 to obtain product 17: Under an ambient atmosphere, the DNA-conjugate **SRu-17** stock solution in water (c = 0.10 mM, 20 µL) was irradiated with a 390 nm (40 W) Kessil lamp for 2 hours, while maintaining the temperature at approximately 30 °C through cooling with a fan. To the reaction mixture was added the stock solution of NaCl in water (**SR-06**, 2.0 µL, c = 5.0 M, 10% volume of the total reaction volume), followed by cold ethanol (−20 °C, 66 µL) to precipitate the DNA conjugate **17**. The Eppendorf tube was placed in the freezer (−20 °C) for at least 1 hour, and then it was centrifuged at 4 °C and 11000 × g for at least 30 minutes. The supernatant was removed, the pellet dried under air and dissolved in Milli-Q water to

obtain the purified DNA-conjugate **17**. Then, 1 µL of the above solution was diluted to 40 µL with water for LC–MS analysis. The yield of the DNA conjugate was calculated by measuring the integration of the peaks of the diode array detection (DAD) UV absorbance at 260 nm of the LC–MS trace, assuming complete DNA recovery and identical UV absorbance.

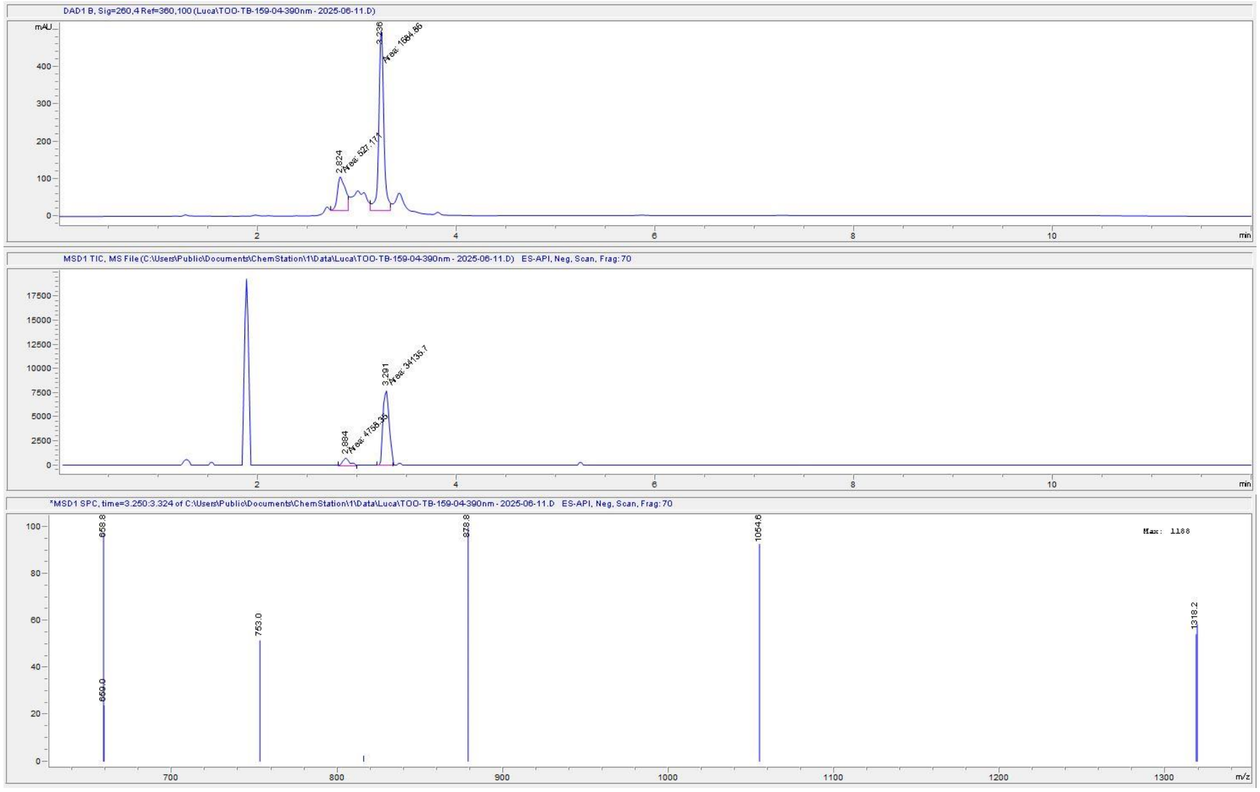

Figure S29. Analytical HPLC trace of **17** with HPLC method A. (Up) DAD chromatogram at 260 nm. (Middle) TIC chromatogram. (Bottom) Ionization of peak at 3.29 min. containing reaction product.

Synthesis of DNA-conjugate 18

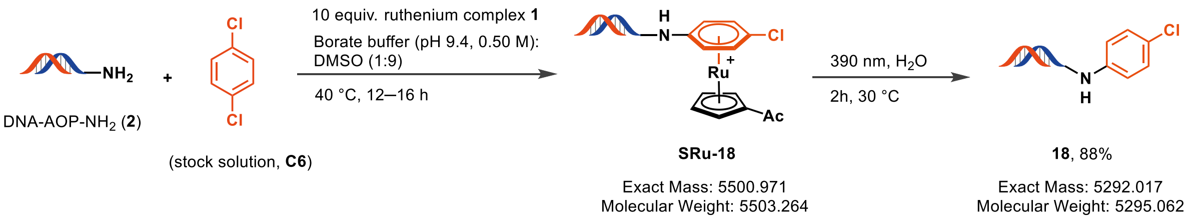

In situ formation of arene-ruthenium stock solution: Under an ambient atmosphere, a 1 mL glass GC vial equipped with a 6 mm Teflon-coated stirring bar was charged with ruthenium complex **1** (1.4 mg, 2.9 µmol, 1.0 equiv.). Next, a stock solution of 1,4-dichlorobenzene in DMC (294 µL, c = 0.10 M, 29 µmol, 10 equiv.) was added. The resulting reaction mixture was heated at 80 °C for 2 hours. After 2 hours, the reaction mixture was cooled to 23 °C. Next, the DMC was removed under a gentle stream of argon and 294 µL of DMSO were added to result an in situ formed stock solution of arene-ruthenium complex **C6** (294 µL, c =

0.01 M, assuming quantitative arene coordination to ruthenium).

Under an ambient atmosphere, the stock solution **SD-01** of DNA-AOP-NH_2 (**2**) in water (1.0 µL, c = 2.0 mM, 2.0 nmol, 1.0 equiv.) was added to a 1.5 mL Eppendorf tube, followed by sodium borate buffer (1.0 µL, pH 9.4, c = 0.50 M). To this mixture, 16 µL of DMSO was added and the solution was vortexed for 5 seconds. Next, the freshly prepared stock solution **C6** (2.0 µL, c = 0.01 M, 0.02 µmol, 10 equiv.) in DMSO was added. The resulting reaction mixture was vortexed for 5 seconds, transferred to a thermocycler at 40 °C, and incubated for 16 hours at 800 rpm to yield the DNA-conjugate **SRu-18**. Next, the reaction mixture was diluted with 10 µL of Milli-Q water. To the reaction mixture was added the stock solution of NaCl in water (**SR-06** , 3.0 µL, c = 5.0 M, 10% volume of the total reaction volume), followed by cold ethanol (−20 °C, 99 µL) to precipitate the *N*-arylated ruthenium DNA conjugate **SRu-18**. The Eppendorf tube was placed in a freezer (−20 °C) for at least 1 hour, and then it was centrifuged at 4 °C and 11000 × g for at least 30 minutes. The supernatant was removed and the pellet was dried under air, then dissolved in 20 µL water to obtain the DNA-conjugate **SRu-18** (20 µL, c = 0.10 mM). Then, 1.0 µL of the above solution was diluted to 40 µL with water for LC–MS analysis.

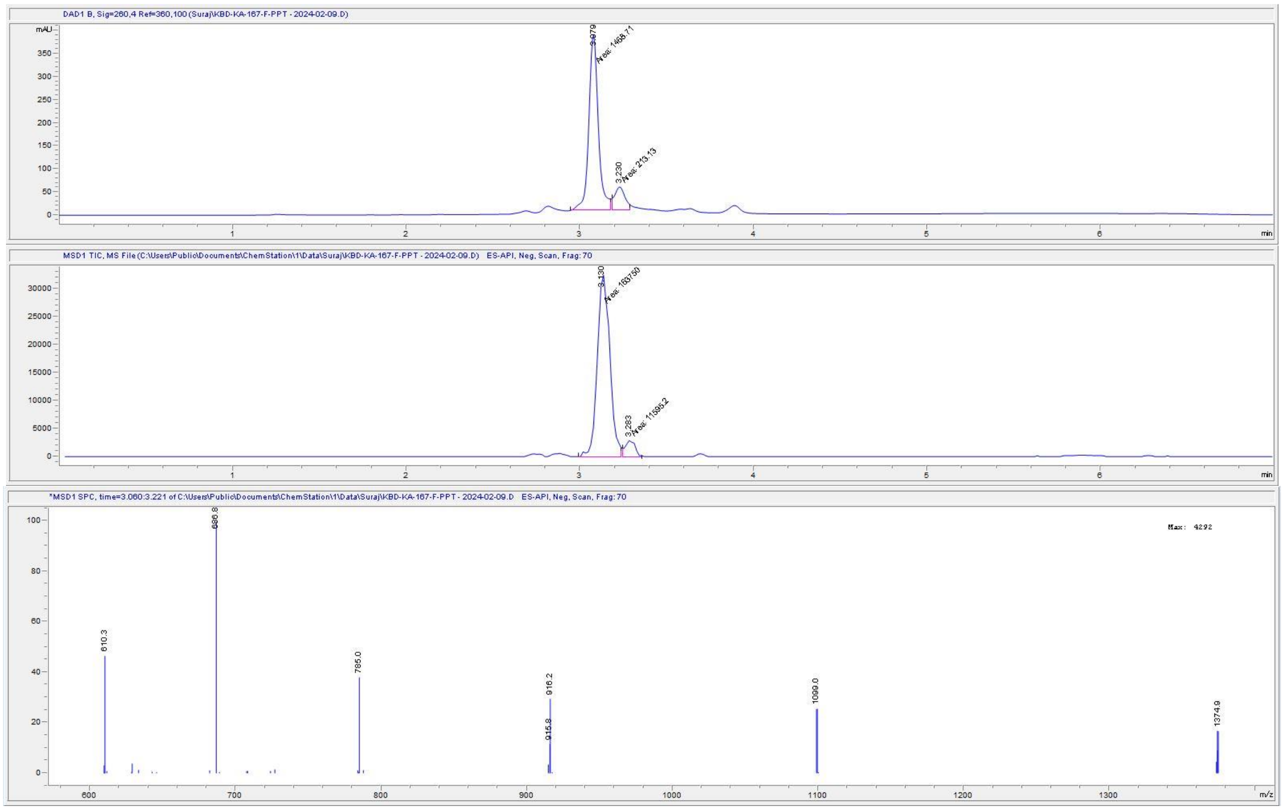

Figure S31. Analytical HPLC trace of **SRu-18** with HPLC method B. (Top) DAD chromatogram at 260 nm. (Middle) TIC chromatogram. (Bottom) Ionization of peak at 3.13 min containing reaction product.

Decomplexation of SRu-18 to obtain product 18: Under an ambient atmosphere, the DNA-conjugate **SRu-18** stock solution in water (c = 0.10 mM, 20 µL) was irradiated with a 390 nm (40 W) Kessil lamp for 2 hours, while maintaining the temperature at approximately 30 °C through cooling with a fan. To the reaction mixture

was added the stock solution of NaCl in water (**SR-06**, 2.0 µL, c = 5.0 M, 10% volume of the total reaction volume), followed by cold ethanol (−20 °C, 66 µL) to precipitate the DNA conjugate **18**. The Eppendorf tube was placed in the freezer (−20 °C) for at least 1 hour, and then it was centrifuged at 4 °C and 11000 × g for at least 30 minutes. The supernatant was removed, the pellet dried under air and dissolved in Milli-Q water to obtain the purified DNA-conjugate **18**. Then, 1 µL of the above solution was diluted to 40 µL with water for LC–MS analysis. The yield of the DNA conjugate was calculated by measuring the integration of the peaks of the diode array detection (DAD) UV absorbance at 260 nm of the LC–MS trace, assuming complete DNA recovery and identical UV absorbance.

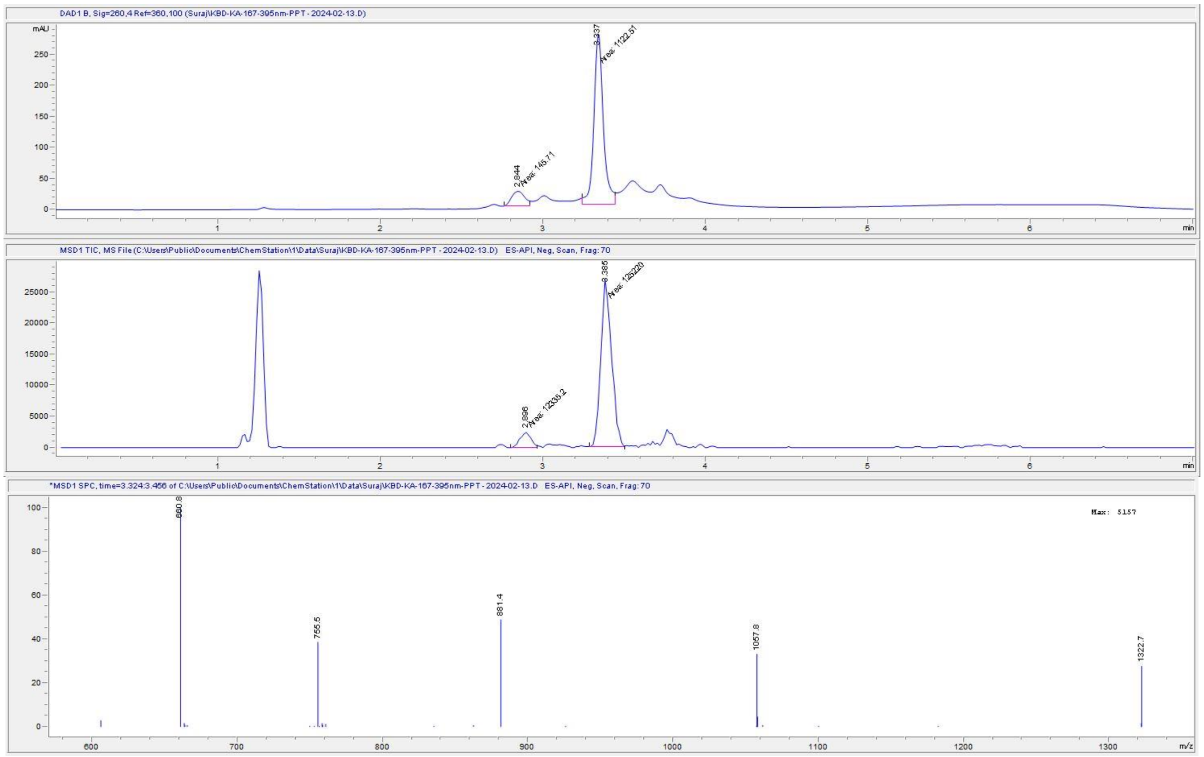

Figure S32. Analytical HPLC trace of **18** with HPLC method B. (Top) DAD chromatogram at 260 nm. (Middle) TIC chromatogram. (Bottom) Ionization of peak at 3.38 min. containing reaction product.

Synthesis of DNA-conjugate 19

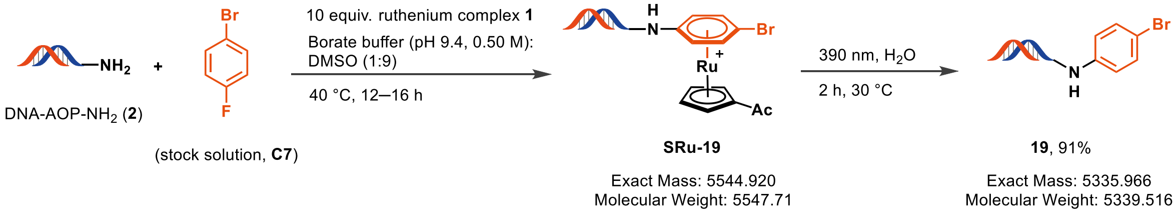

In situ formation of arene-ruthenium stock solution: Under an ambient atmosphere, a 1 mL glass GC vial equipped with a 6 mm Teflon-coated stirring bar was charged with ruthenium complex **1** (1.4 mg, 2.9 µmol, 1.0 equiv.). Next, a stock solution of 1-bromo-4-fluorobenzene in DMC (294 µL, c = 0.10 M, 29 µmol, 10

equiv.) was added. The resulting reaction mixture was heated at 80 °C for 2 hours. After 2 hours, the reaction mixture was cooled to 23 °C. Next, the DMC was removed under a gentle stream of argon and 294 µL of DMSO were added to result an in situ formed stock solution of arene-ruthenium complex **C7** (294 µL, c = 0.01 M, assuming quantitative arene coordination to ruthenium).

Under an ambient atmosphere, the stock solution **SD-01** of DNA-AOP-NH_2 (**2**) in water (1.0 µL, c = 2.0 mM, 2.0 nmol, 1.0 equiv.) was added to a 1.5 mL Eppendorf tube, followed by sodium borate buffer (1.0 µL, pH 9.4, c = 0.50 M). To this mixture, 16 µL of DMSO was added and the solution was vortexed for 5 seconds. Next, the freshly prepared stock solution **C7** (2.0 µL, c = 0.01 M, 0.02 µmol, 10 equiv.) in DMSO was added. The resulting reaction mixture was vortexed for 5 seconds, transferred to a thermocycler at 40 °C, and incubated for 16 hours at 800 rpm to yield the DNA-conjugate **SRu-19**. Next, the reaction mixture was diluted with 10 µL of Milli-Q water. To the reaction mixture was added the stock solution of NaCl in water (**SR-06** , 3.0 µL, c = 5.0 M, 10% volume of the total reaction volume), followed by cold ethanol (−20 °C, 99 µL) to precipitate the *N*-arylated ruthenium DNA conjugate **SRu-19**. The Eppendorf tube was placed in a freezer (−20 °C) for at least 1 hour, and then it was centrifuged at 4 °C and 11000 × g for at least 30 minutes. The supernatant was removed and the pellet was dried under air, then dissolved in 20 µL water to obtain the DNA-conjugate **SRu-19** (20 µL, c = 0.10 mM). Then, 1.0 µL of the above solution was diluted to 40 µL with water for LC–MS analysis.

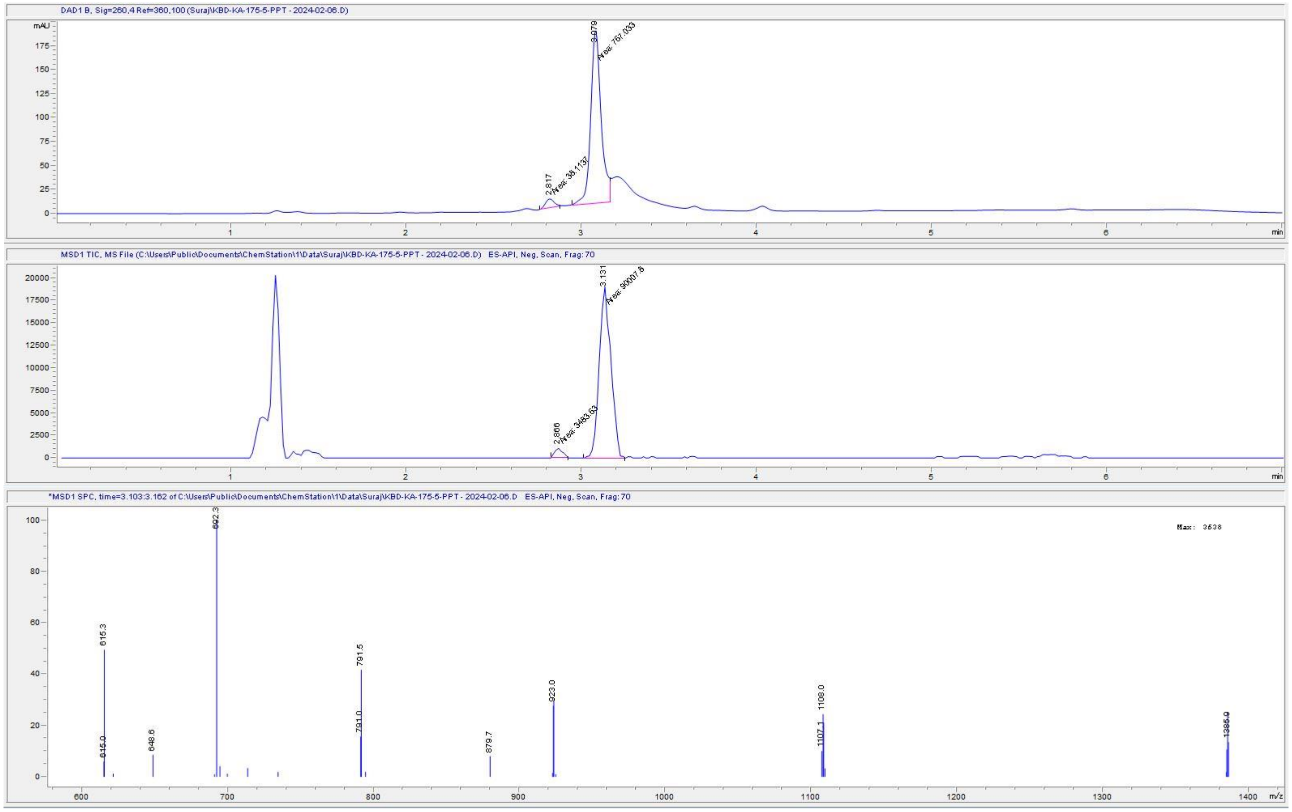

Figure S33. Analytical HPLC trace of **SRu-19** with HPLC method B. (Top) DAD chromatogram at 260 nm. (Middle) TIC chromatogram. (Bottom) Ionization of peak at 3.13 min. containing reaction product.

Decomplexation of SRu-19 to obtain product 19: Under an ambient atmosphere, the DNA-conjugate **SRu-19** stock solution in water (c = 0.10 mM, 20 µL) was irradiated with a 390 nm (40 W) Kessil lamp for 2 hours, while maintaining the temperature at approximately 30 °C through cooling with a fan. To the reaction mixture was added the stock solution of NaCl in water (**SR-06**, 2.0 µL, c = 5.0 M, 10% volume of the total reaction volume), followed by cold ethanol (−20 °C, 66 µL) to precipitate the DNA conjugate **19**. The Eppendorf tube was placed in the freezer (−20 °C) for at least 1 hour, and then it was centrifuged at 4 °C and 11000 × g for at least 30 minutes. The supernatant was removed, the pellet dried under air and dissolved in Milli-Q water to obtain the purified DNA-conjugate **19**. Then, 1 µL of the above solution was diluted to 40 µL with water for LC–MS analysis. The yield of the DNA conjugate was calculated by measuring the integration of the peaks of the diode array detection (DAD) UV absorbance at 260 nm of the LC–MS trace, assuming complete DNA recovery and identical UV absorbance.

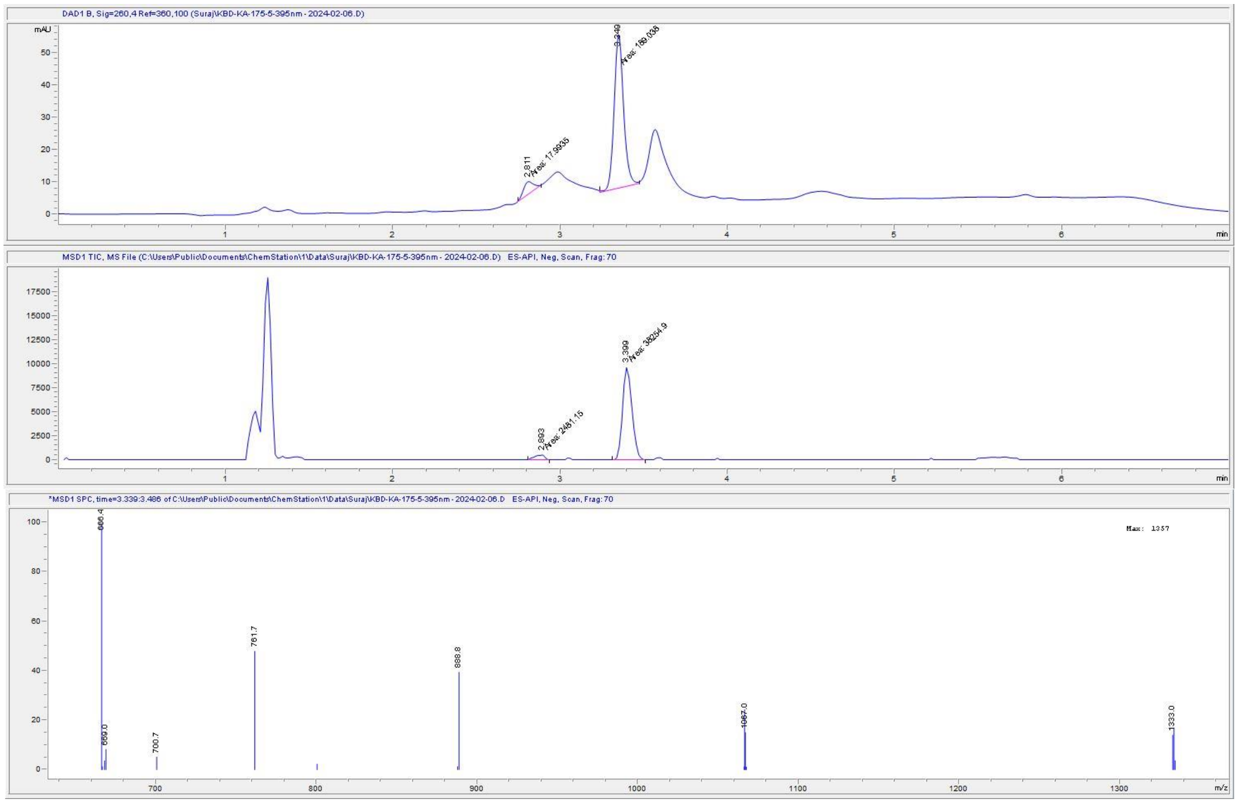

Figure S34. Analytical HPLC trace of **19** with HPLC method B. (Top) DAD chromatogram at 260 nm. (Middle) TIC chromatogram. (Bottom) Ionization of peak at 3.40 min. containing reaction product.

Synthesis of DNA-conjugate 20

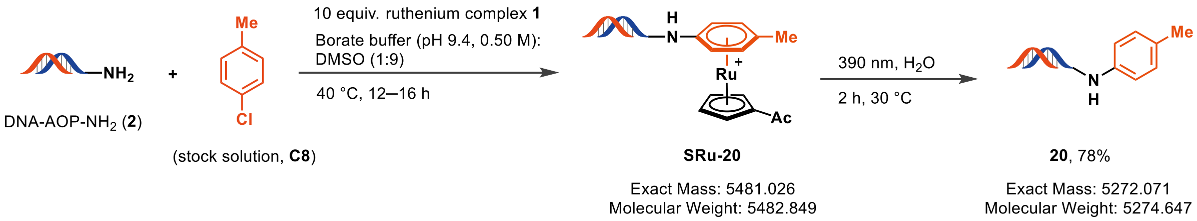

In situ formation of arene-ruthenium stock solution: Under an ambient atmosphere, a 1 mL glass GC vial equipped with a 6 mm Teflon-coated stirring bar was charged with ruthenium complex **1** (1.4 mg, 2.9 µmol, 1.0 equiv.). Next, a stock solution of 1-chloro-4-methylbenzene in DMC (294 µL, c = 0.10 M, 29 µmol, 10 equiv.) was added. The resulting reaction mixture was heated at 80 °C for 2 hours. After 2 hours, the reaction mixture was cooled to 23 °C. Next, the DMC was removed under a gentle stream of argon and 294 µL of DMSO were added to result an in situ formed stock solution of arene-ruthenium complex **C8** (294 µL, c = 0.01 M, assuming quantitative arene coordination to ruthenium).

Under an ambient atmosphere, the stock solution **SD-01** of DNA-AOP-NH_2 (**2**) in water (1.0 µL, c = 2.0 mM, 2.0 nmol, 1.0 equiv.) was added to a 1.5 mL Eppendorf tube, followed by sodium borate buffer (1.0 µL, pH 9.4, c = 0.50 M). To this mixture, 16 µL of DMSO was added and the solution was vortexed for 5 seconds. Next, the freshly prepared stock solution **C8** (2.0 µL, c = 0.01 M, 0.02 µmol, 10 equiv.) in DMSO was added. The resulting reaction mixture was vortexed for 5 seconds, transferred to a thermocycler at 40 °C, and incubated for 16 hours at 800 rpm to yield the DNA-conjugate **SRu-20**. Next, the reaction mixture was diluted with 10 µL of Milli-Q water. To the reaction mixture was added the stock solution of NaCl in water (**SR-06** , 3.0 µL, c = 5.0 M, 10% volume of the total reaction volume), followed by cold ethanol (−20 °C, 99 µL) to precipitate the *N*-arylated ruthenium DNA conjugate **SRu-20**. The Eppendorf tube was placed in a freezer (−20 °C) for at least 1 hour, and then it was centrifuged at 4 °C and 11000 × g for at least 30 minutes. The supernatant was removed and the pellet was dried under air, then dissolved in 20 µL water to obtain the DNA-conjugate **SRu-20** (20 µL, c = 0.10 mM). Then, 1.0 µL of the above solution was diluted to 40 µL with water for LC–MS analysis.

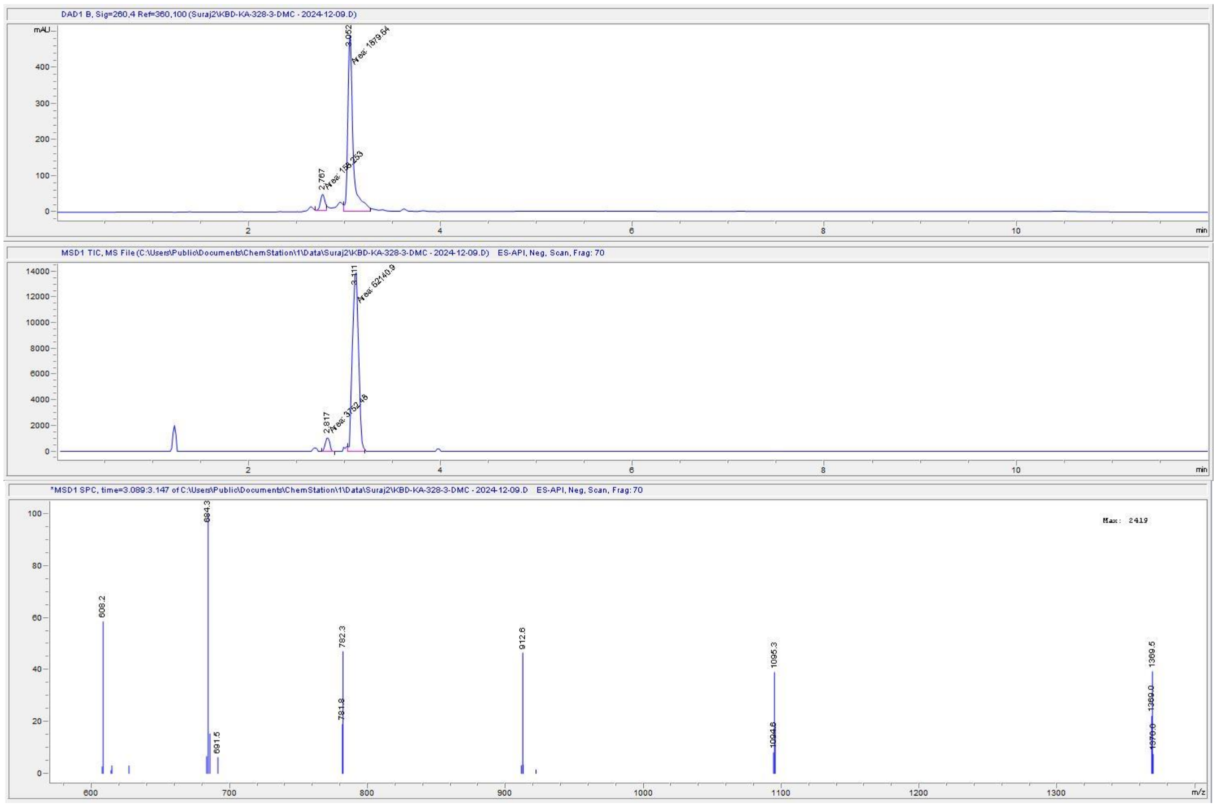

Figure S35. Analytical HPLC trace of **SRu-20** with HPLC method A. (Top) DAD chromatogram at 260 nm. (Middle) TIC chromatogram. (Bottom) Ionization of peak at 3.11 min. containing reaction product. Conversion was calculated using the TIC chromatogram.

Decomplexation of SRu-20 to obtain product 20: Under an ambient atmosphere, the DNA-conjugate **SRu-20** stock solution in water (c = 0.10 mM, 20 µL) was irradiated with a 390 nm (40 W) Kessil lamp for 2 hours, while maintaining the temperature at approximately 30 °C through cooling with a fan. To the reaction mixture was added the stock solution of NaCl in water (**SR-06**, 2.0 µL, c = 5.0 M, 10% volume of the total reaction volume), followed by cold ethanol (−20 °C, 66 µL) to precipitate the DNA conjugate **20**. The Eppendorf tube was placed in the freezer (−20 °C) for at least 1 hour, and then it was centrifuged at 4 °C and 11000 × g for at least 30 minutes. The supernatant was removed, the pellet dried under air and dissolved in Milli-Q water to obtain the purified DNA-conjugate **20**. Then, 1 µL of the above solution was diluted to 40 µL with water for LC–MS analysis. The yield of the DNA conjugate was calculated by measuring the integration of the peaks of the diode array detection (DAD) UV absorbance at 260 nm of the LC–MS trace, assuming complete DNA recovery and identical UV absorbance.

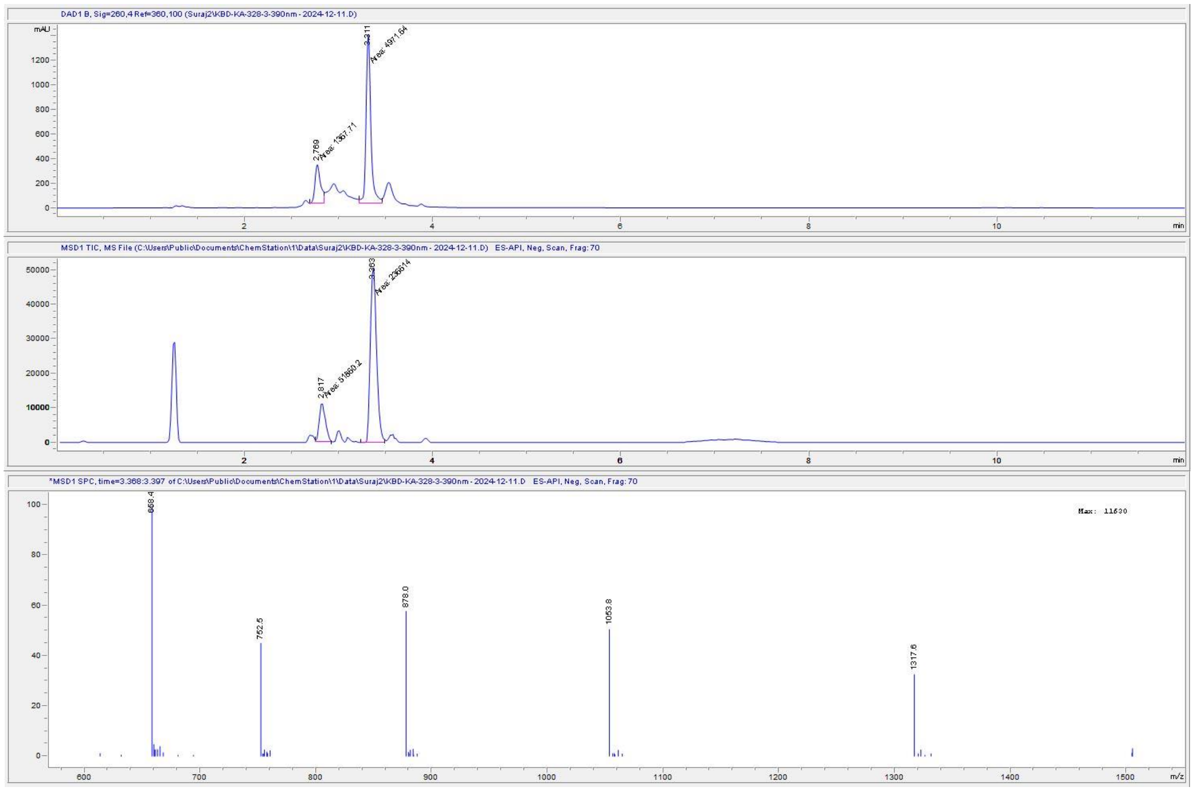

Figure S36. Analytical HPLC trace of **20** with HPLC method A. (Top) DAD chromatogram at 260 nm. (Middle) TIC chromatogram. (Bottom) Ionization of peak at 3.36 min. containing reaction product.

Synthesis of DNA-conjugate 21

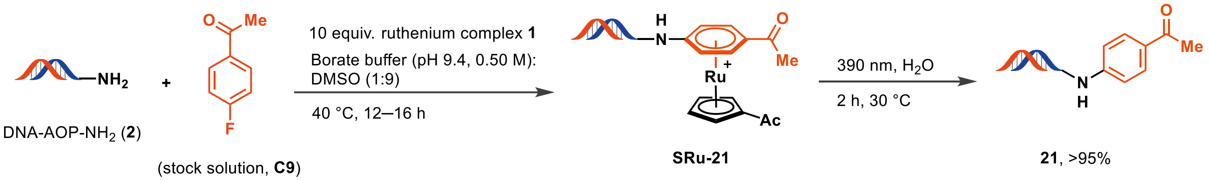

Exact Mass: 5509.020
Molecular Weight: 5510.859

Exact Mass: 5300.066
Molecular Weight: 5302.657

In situ formation of arene-ruthenium stock solution: Under an ambient atmosphere, a 1 mL glass GC vial equipped with a 6 mm Teflon-coated stirring bar was charged with ruthenium complex **1** (1.4 mg, 2.9 µmol, 1.0 equiv.). Next, a stock solution of 1-(4-fluorophenyl)ethan-1-one in DMC (294 µL, c = 0.10 M, 29 µmol, 10 equiv.) was added. The resulting reaction mixture was heated at 80 °C for 2 hours. After 2 hours, the reaction mixture was cooled to 23 °C. Next, the DMC was removed under a gentle stream of argon and 294 µL of DMSO were added to result an in situ formed stock solution of arene-ruthenium complex **C9** (294 µL, c = 0.01 M, assuming quantitative arene coordination to ruthenium).

Under an ambient atmosphere, the stock solution **SD-01** of DNA-AOP-NH_2 (**2**) in water (1.0 µL, c = 2.0 mM, 2.0 nmol, 1.0 equiv.) was added to a 1.5 mL Eppendorf tube, followed by sodium borate buffer (1.0 µL, pH 9.4, c = 0.50 M). To this mixture, 16 µL of DMSO was added and the solution was vortexed for 5 seconds. Next, the freshly prepared stock solution **C9** (2.0 µL, c = 0.01 M, 0.02 µmol, 10 equiv.) in DMSO was added. The resulting reaction mixture was vortexed for 5 seconds, transferred to a thermocycler at 40 °C, and incubated for 16 hours at 800 rpm to yield the DNA-conjugate **SRu-21**. Next, the reaction mixture was diluted with 10 µL of Milli-Q water. To the reaction mixture was added the stock solution of NaCl in water (**SR-06** , 3.0 µL, c = 5.0 M, 10% volume of the total reaction volume), followed by cold ethanol (−20 °C, 99 µL) to precipitate the *N*-arylated ruthenium DNA conjugate **SRu-21**. The Eppendorf tube was placed in a freezer (−20 °C) for at least 1 hour, and then it was centrifuged at 4 °C and 11000 × g for at least 30 minutes. The supernatant was removed and the pellet was dried under air, then dissolved in 20 µL water to obtain the DNA-conjuagte **SRu-21** (20 µL, c = 0.10 mM). Then, 1.0 µL of the above solution was diluted to 40 µL with water for LC–MS analysis.

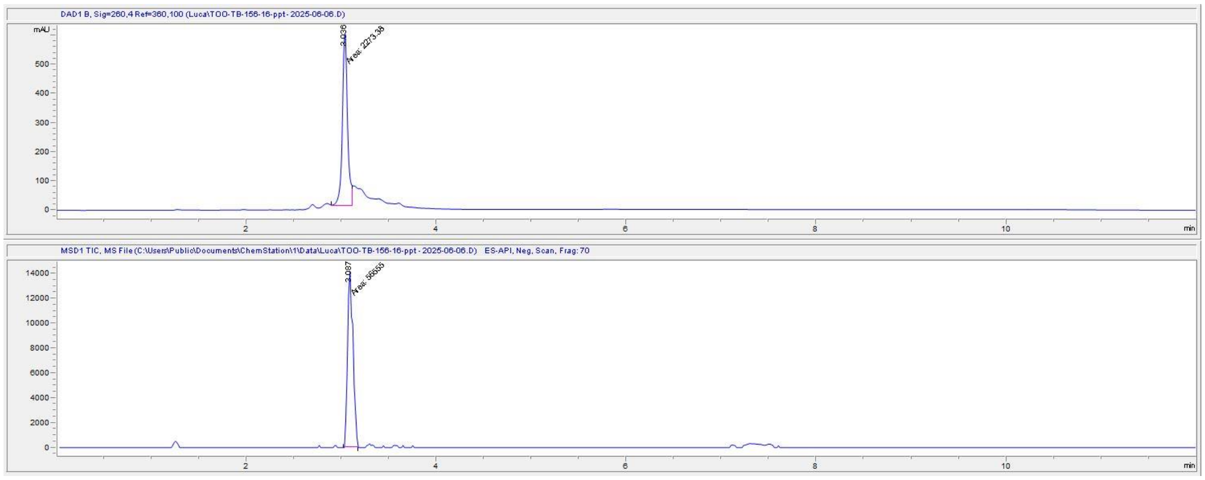

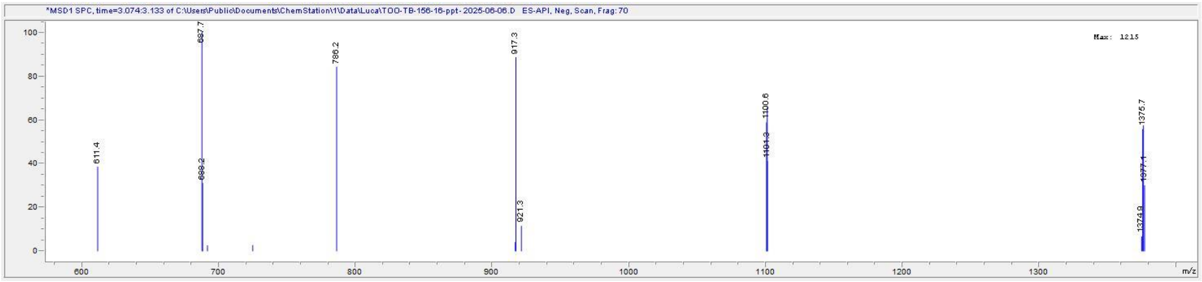

Figure S37. Analytical HPLC trace of **SRu-21** with HPLC method A. (Top) DAD chromatogram at 260 nm. (Middle) TIC chromatogram. (Bottom) Ionization of peak at 3.09 min. containing reaction product.

Decomplexation of SRu-21 to obtain product 21: Under an ambient atmosphere, the DNA-conjugate **SRu-21** stock solution in water (c = 0.10 mM, 20 µL) was irradiated with a 390 nm (40 W) Kessil lamp for 2 hours, while maintaining the temperature at approximately 30 °C through cooling with a fan. To the reaction mixture was added the stock solution of NaCl in water (**SR-06**, 2.0 µL, c = 5.0 M, 10% volume of the total reaction volume), followed by cold ethanol (−20 °C, 66 µL) to precipitate the DNA conjugate **21**. The Eppendorf tube was placed in the freezer (−20 °C) for at least 1 hour, and then it was centrifuged at 4 °C and 11000 × g for at least 30 minutes. The supernatant was removed, the pellet dried under air and dissolved in Milli-Q water to obtain the purified DNA-conjugate **21**. Then, 1 µL of the above solution was diluted to 40 µL with water for LC–MS analysis. The yield of the DNA conjugate was calculated by measuring the integration of the peaks of the diode array detection (DAD) UV absorbance at 260 nm of the LC–MS trace, assuming complete DNA recovery and identical UV absorbance.

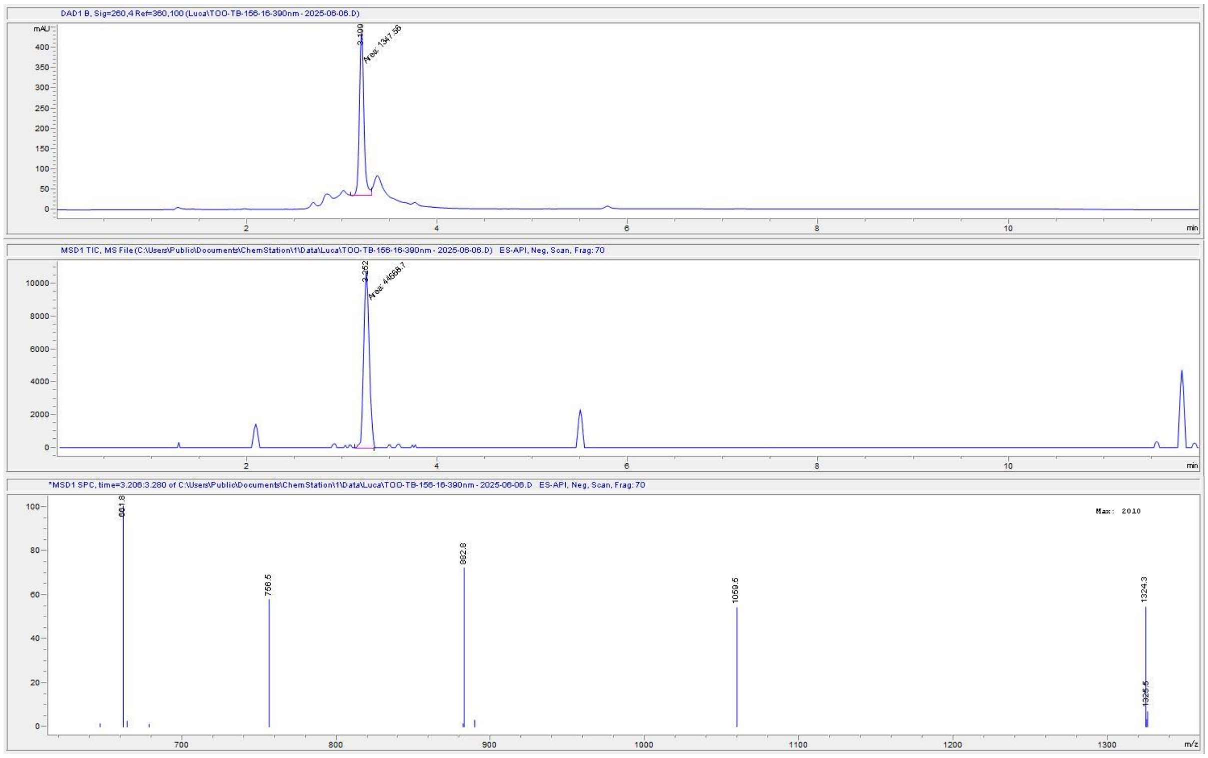

Figure S38. Analytical HPLC trace of **21** with HPLC method A. (Top) DAD chromatogram at 260 nm. (Middle) TIC chromatogram. (Bottom) Ionization of peak at 3.25 min. containing reaction product.

Synthesis of DNA-conjugate 22

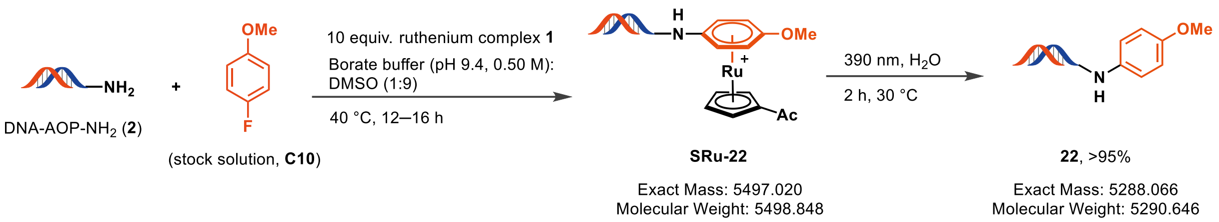

In situ formation of arene-ruthenium stock solution: Under an ambient atmosphere, a 1 mL glass GC vial equipped with a 6 mm Teflon-coated stirring bar was charged with ruthenium complex **1** (1.4 mg, 2.9 µmol, 1.0 equiv.). Next, a stock solution of 1-fluoro-4-methoxybenzene in DMC (294 µL, c = 0.10 M, 29 µmol, 10 equiv.) was added. The resulting reaction mixture was heated at 80 °C for 2 hours. After 2 hours, the reaction mixture was cooled to 23 °C. Next, the DMC was removed under a gentle stream of argon and 294 µL of DMSO were added to result an in situ formed stock solution of arene-ruthenium complex **C10** (294 µL, c = 0.01 M, assuming quantitative arene coordination to ruthenium).

Under an ambient atmosphere, the stock solution **SD-01** of DNA-AOP-NH_2 (**2**) in water (1.0 µL, c = 2.0 mM, 2.0 nmol, 1.0 equiv.) was added to a 1.5 mL Eppendorf tube, followed by sodium borate buffer (1.0 µL, pH 9.4, c = 0.50 M). To this mixture, 16 µL of DMSO was added and the solution was vortexed for 5 seconds. Next, the freshly prepared stock solution **C10** (2.0 µL, c = 0.01 M, 0.02 µmol, 10 equiv.) in DMSO was added. The resulting reaction mixture was vortexed for 5 seconds, transferred to a thermocycler at 40 °C, and incubated for 16 hours at 800 rpm to yield the DNA-conjugate **SRu-22**. Next, the reaction mixture was diluted with 10 µL of Milli-Q water. To the reaction mixture was added the stock solution of NaCl in water (**SR-06** , 3.0 µL, c = 5.0 M, 10% volume of the total reaction volume), followed by cold ethanol (−20 °C, 99 µL) to precipitate the *N*-arylated ruthenium DNA conjugate **SRu-22**. The Eppendorf tube was placed in a freezer (−20 °C) for at least 1 hour, and then it was centrifuged at 4 °C and 11000 × g for at least 30 minutes. The supernatant was removed and the pellet was dried under air, then dissolved in 20 µL water to obtain the DNA-conjugate **SRu-22** (20 µL, c = 0.10 mM). Then, 1.0 µL of the above solution was diluted to 40 µL with water for LC–MS analysis.

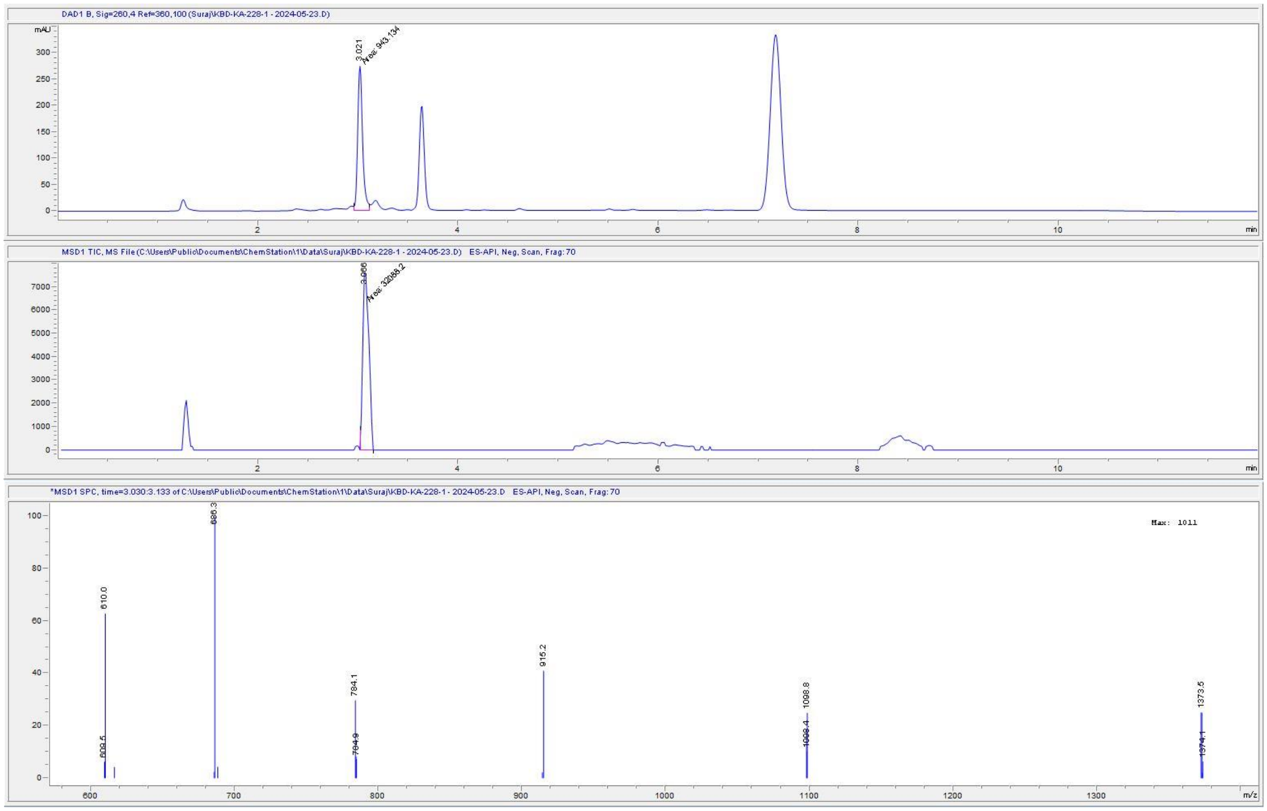

Figure S39. Analytical HPLC trace of **SRu-22** with HPLC method A. (Top) DAD chromatogram at 260 nm. (Middle) TIC chromatogram. (Bottom) Ionization of peak at 3.07 min. containing reaction product.

Decomplexation of SRu-22 to obtain product 22: Under an ambient atmosphere, the DNA-conjugate **SRu-22** stock solution in water (c = 0.10 mM, 20 µL) was irradiated with a 390 nm (40 W) Kessil lamp for 2 hours, while maintaining the temperature at approximately 30 °C through cooling with a fan. To the reaction mixture was added the stock solution of NaCl in water (**SR-06**, 2.0 µL, c = 5.0 M, 10% volume of the total reaction volume), followed by cold ethanol (−20 °C, 66 µL) to precipitate the DNA conjugate **22**. The Eppendorf tube was placed in the freezer (−20 °C) for at least 1 hour, and then it was centrifuged at 4 °C and 11000 × g for at least 30 minutes. The supernatant was removed, the pellet dried under air and dissolved in Milli-Q water to obtain the purified DNA-conjugate **22**. Then, 1 µL of the above solution was diluted to 40 µL with water for LC–MS analysis. The yield of the DNA conjugate was calculated by measuring the integration of the peaks of the diode array detection (DAD) UV absorbance at 260 nm of the LC–MS trace, assuming complete DNA recovery and identical UV absorbance.

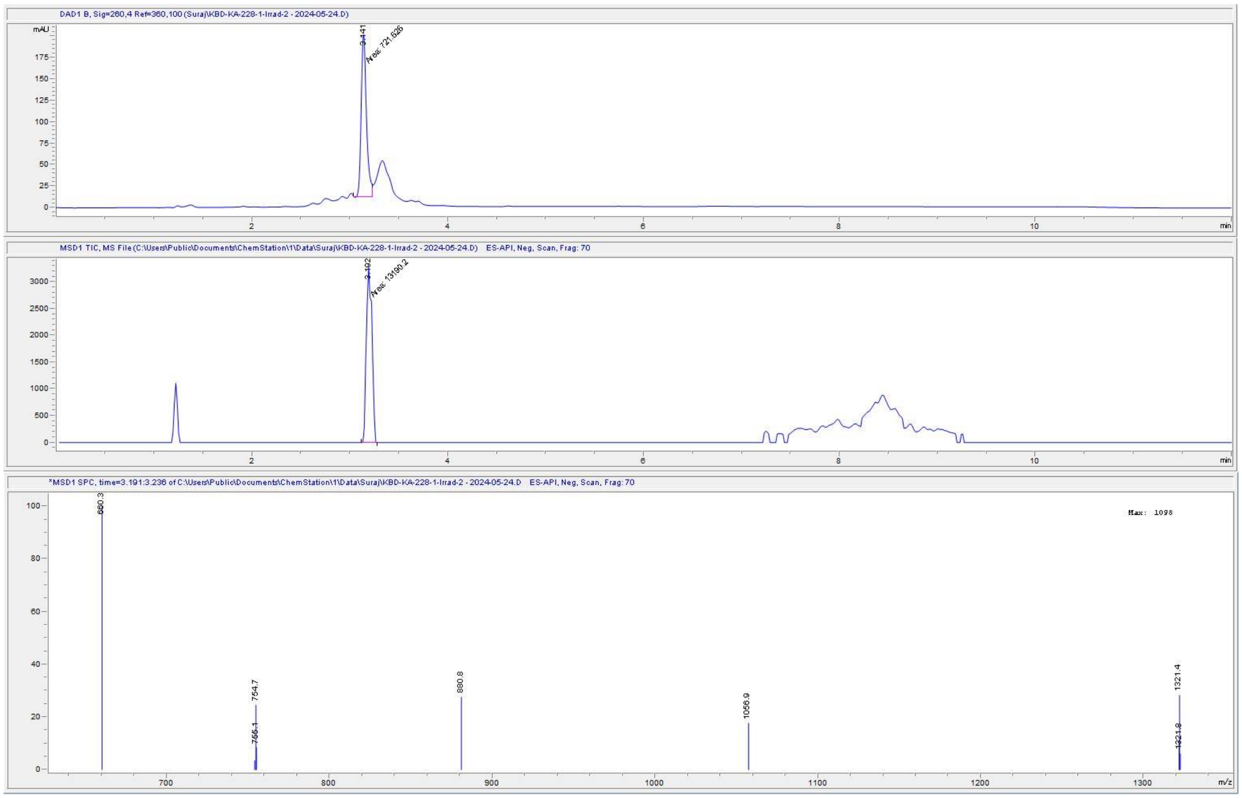

Figure S40. Analytical HPLC trace of **22** with HPLC method A. (Top) DAD chromatogram at 260 nm. (Middle) TIC chromatogram. (Bottom) Ionization of peak at 3.19 min. containing reaction product.

Synthesis of DNA-conjugate 23

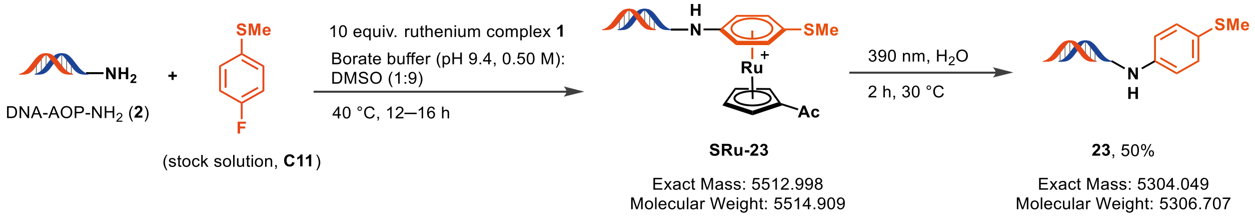

In situ formation of arene-ruthenium stock solution: Under an ambient atmosphere, a 1 mL glass GC vial equipped with a 6 mm Teflon-coated stirring bar was charged with ruthenium complex **1** (1.4 mg, 2.9 µmol, 1.0 equiv.). Next, a stock solution of (4-fluorophenyl)(methyl)sulfane in DMC (294 µL, c = 0.10 M, 29 µmol, 10 equiv.) was added. The resulting reaction mixture was heated at 80 °C for 2 hours. After 2 hours, the reaction mixture was cooled to 23 °C. Next, the DMC was removed under a gentle stream of argon and 294 µL of DMSO were added to result an in situ formed stock solution of arene-ruthenium complex **C11** (294 µL, c= 0.01 M, assuming quantitative arene coordination to ruthenium).

Under an ambient atmosphere, the stock solution **SD-01** of DNA-AOP-NH2 (**2**) in water (1.0 µL, c = 2.0 mM, 2.0 nmol, 1.0 equiv.) was added to a 1.5 mL Eppendorf tube, followed by sodium borate buffer (1.0 µL, pH 9.4, c = 0.50 M). To this mixture, 16 µL of DMSO was added and the solution was vortexed for 5 seconds. Next, the freshly prepared stock solution **C11** (2.0 µL, c = 0.01 M, 0.02 µmol, 10 equiv.) in DMSO was added.

The resulting reaction mixture was vortexed for 5 seconds, transferred to a thermocycler at 40 °C, and incubated for 16 hours at 800 rpm to yield the DNA-conjugate **SRu-23**. Next, the reaction mixture was diluted with 10 µL of Milli-Q water. To the reaction mixture was added the stock solution of NaCl in water (**SR-06** , 3.0 µL, c = 5.0 M, 10% volume of the total reaction volume), followed by cold ethanol (−20 °C, 99 µL) to precipitate the *N*-arylated ruthenium DNA conjugate **SRu-23**. The Eppendorf tube was placed in a freezer (−20 °C) for at least 1 hour, and then it was centrifuged at 4 °C and 11000 × g for at least 30 minutes. The supernatant was removed and the pellet was dried under air, then dissolved in 20 µL water to obtain the DNA-conjugate **SRu-23** (20 µL, c = 0.10 mM). Then, 1.0 µL of the above solution was diluted to 40 µL with water for LC–MS analysis.

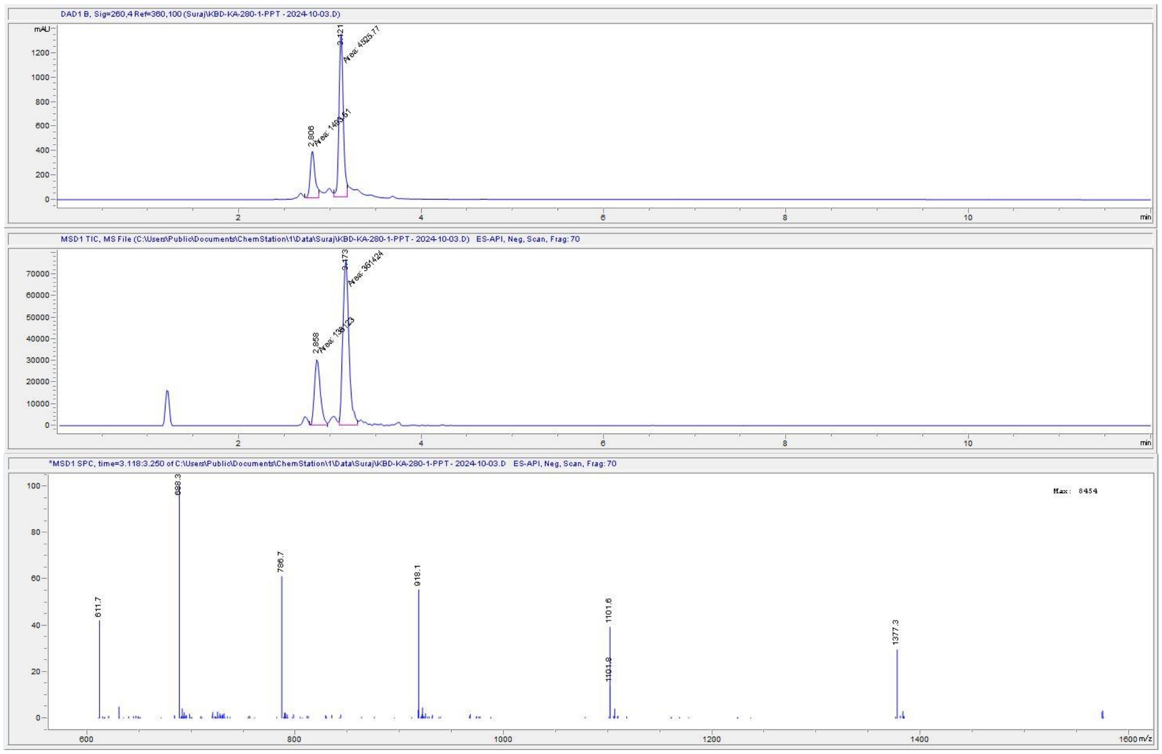

Figure S41. Analytical HPLC trace of **SRu-23** with HPLC method B. (Top) DAD chromatogram at 260 nm. (Middle) TIC chromatogram. (Bottom) Ionization of peak at 3.17 min. containing reaction product.

Decomplexation of SRu-23 to obtain product 23: Under an ambient atmosphere, the DNA-conjugate **SRu-23** stock solution in water (c = 0.10 mM, 20 µL) was irradiated with a 390 nm (40 W) Kessil lamp for 2 hours, while maintaining the temperature at approximately 30 °C through cooling with a fan. To the reaction mixture was added the stock solution of NaCl in water (**SR-06**, 2.0 µL, c = 5.0 M, 10% volume of the total reaction volume), followed by cold ethanol (−20 °C, 66 µL) to precipitate the DNA conjugate **23**. The Eppendorf tube was placed in the freezer (−20 °C) for at least 1 hour, and then it was centrifuged at 4 °C and 11000 × g for at least 30 minutes. The supernatant was removed, the pellet dried under air and dissolved in Milli-Q water to obtain the purified DNA-conjugate **23**. Then, 1 µL of the above solution was diluted to 40 µL with water for LC–MS analysis. The yield of the DNA conjugate was calculated by measuring the integration of the peaks of

the diode array detection (DAD) UV absorbance at 260 nm of the LC–MS trace, assuming complete DNA recovery and identical UV absorbance.

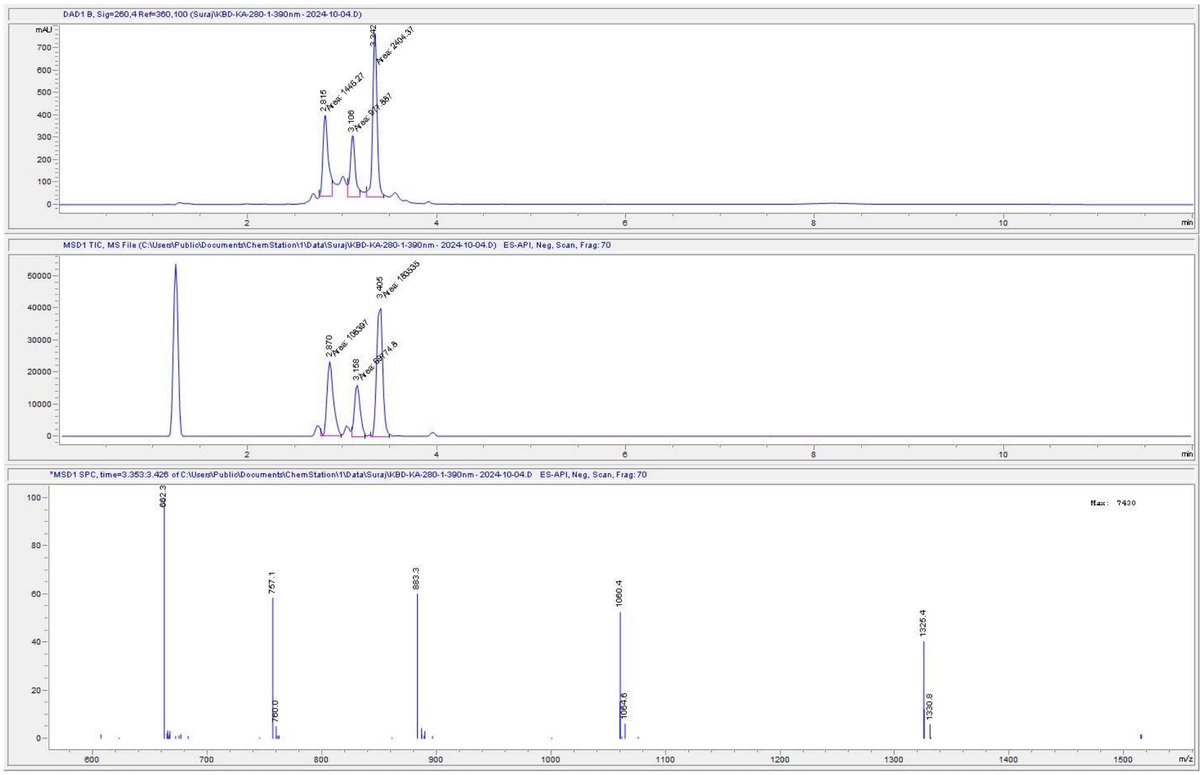

Figure S42. Analytical HPLC trace of **23** with HPLC method A. (Top) DAD chromatogram at 260 nm. (Middle) TIC chromatogram. (Bottom) Ionization of peak at 3.40 min. containing reaction product.

Synthesis of DNA-conjugate 24

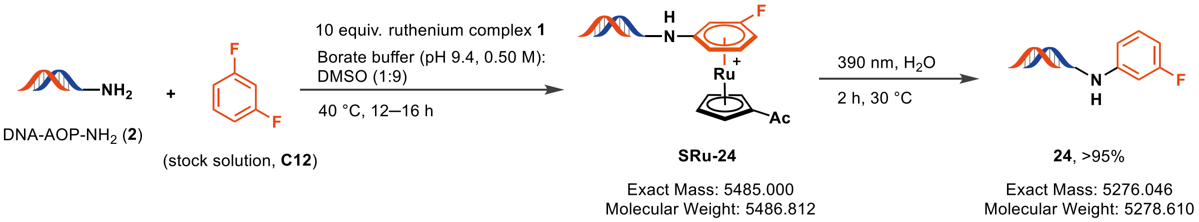

In situ formation of arene-ruthenium stock solution: Under an ambient atmosphere, a 1 mL glass GC vial equipped with a 6 mm Teflon-coated stirring bar was charged with ruthenium complex **1** (1.4 mg, 2.9 µmol, 1.0 equiv.). Next, a stock solution of 1,3-fluorobenzene in DMC (294 µL, c = 0.10 M, 29 µmol, 10 equiv.) was added. The resulting reaction mixture was heated at 80 °C for 2 hours. After 2 hours, the reaction mixture was cooled to 23 °C. Next, the DMC was removed under a gentle stream of argon and 294 µL of DMSO were added to result an in situ formed stock solution of arene-ruthenium complex **C12** (294 µL, c = 0.01 M, assuming quantitative arene coordination to ruthenium).

Under an ambient atmosphere, the stock solution **SD-01** of DNA-AOP-NH2 (**2**) in water (1.0 µL, c = 2.0 mM,

2.0 nmol, 1.0 equiv.) was added to a 1.5 mL Eppendorf tube, followed by sodium borate buffer (1.0 µL, pH 9.4, c = 0.50 M). To this mixture, 16 µL of DMSO was added and the solution was vortexed for 5 seconds. Next, the freshly prepared stock solution **C12** (2.0 µL, c = 0.01 M, 0.02 µmol, 10 equiv.) in DMSO was added. The resulting reaction mixture was vortexed for 5 seconds, transferred to a thermocycler at 40 °C, and incubated for 16 hours at 800 rpm to yield the DNA-conjugate **SRu-24**. Next, the reaction mixture was diluted with 10 µL of Milli-Q water. To the reaction mixture was added the stock solution of NaCl in water (**SR-06** , 3.0 µL, c = 5.0 M, 10% volume of the total reaction volume), followed by cold ethanol (−20 °C, 99 µL) to precipitate the *N*-arylated ruthenium DNA conjugate **SRu-24**. The Eppendorf tube was placed in a freezer (−20 °C) for at least 1 hour, and then it was centrifuged at 4 °C and 11000 × g for at least 30 minutes. The supernatant was removed and the pellet was dried under air, then dissolved in 20 µL water to obtain the DNA-conjugate **SRu-24** (20 µL, c = 0.10 mM). Then, 1.0 µL of the above solution was diluted to 40 µL with water for LC–MS analysis.

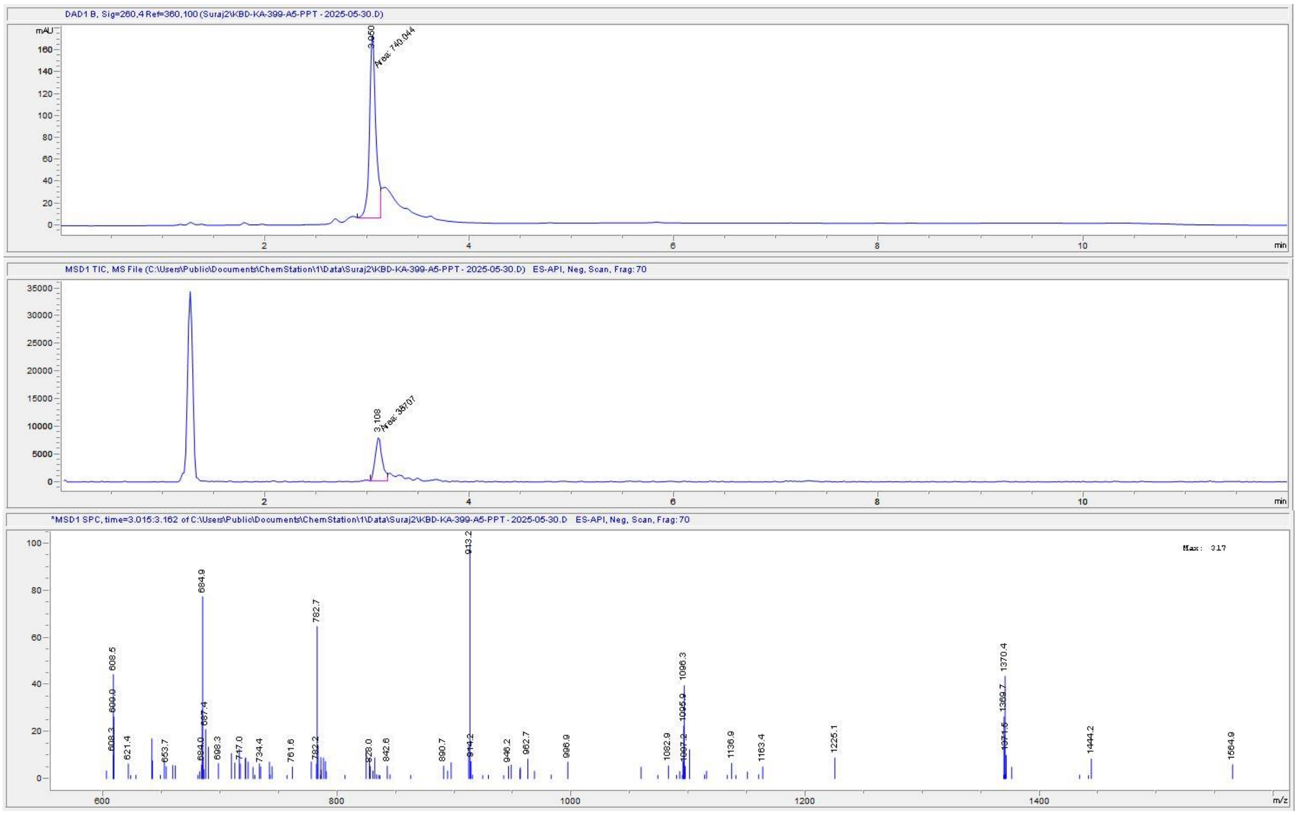

Figure S43. Analytical HPLC trace of **SRu-24** with HPLC method A. (Top) DAD chromatogram at 260 nm. (Middle) TIC chromatogram. (Bottom) Ionization of peak at 3.11 min containing reaction product.

Decomplexation of SRu-24 to obtain product 24: Under an ambient atmosphere, the DNA-conjugate **SRu-24** stock solution in water (c = 0.10 mM, 20 µL) was irradiated with a 390 nm (40 W) Kessil lamp for 2 hours, while maintaining the temperature at approximately 30 °C through cooling with a fan. To the reaction mixture was added the stock solution of NaCl in water (**SR-06**, 2.0 µL, c = 5.0 M, 10% volume of the total reaction volume), followed by cold ethanol (−20 °C, 66 µL) to precipitate the DNA conjugate **24**. The Eppendorf tube

was placed in the freezer (−20 °C) for at least 1 hour, and then it was centrifuged at 4 °C and 11000 × g for at least 30 minutes. The supernatant was removed, the pellet dried under air and dissolved in Milli-Q water to obtain the purified DNA-conjugate **24**. Then, 1 µL of the above solution was diluted to 40 µL with water for LC–MS analysis. The yield of the DNA conjugate was calculated by measuring the integration of the peaks of the diode array detection (DAD) UV absorbance at 260 nm of the LC–MS trace, assuming complete DNA recovery and identical UV absorbance.

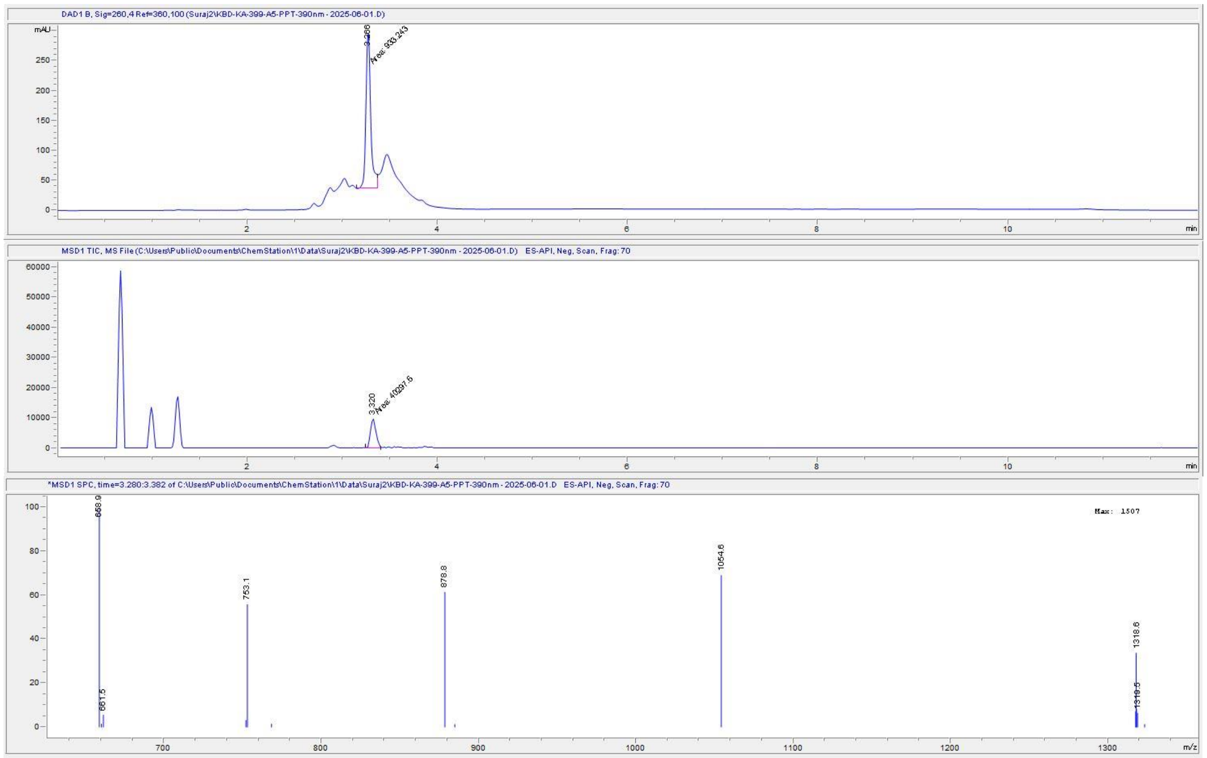

Figure S44. Analytical HPLC trace of **24** with HPLC method A. (Top) DAD chromatogram at 260 nm. (Middle) TIC chromatogram. (Bottom) Ionization of peak at 3.32 min. containing reaction product.

Synthesis of DNA-conjugate 25

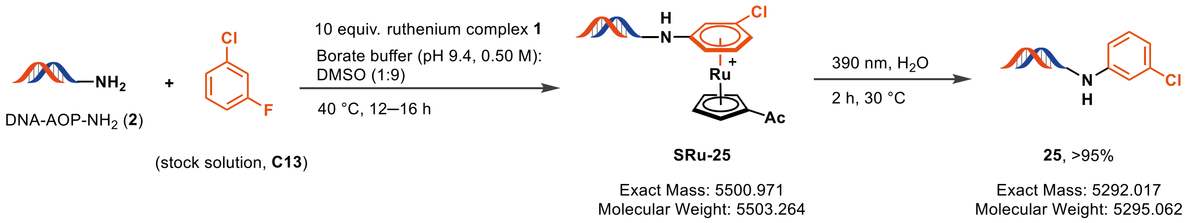

In situ formation of arene-ruthenium stock solution: Under an ambient atmosphere, a 1 mL glass GC vial equipped with a 6 mm Teflon-coated stirring bar was charged with ruthenium complex **1** (1.4 mg, 2.9 µmol, 1.0 equiv.). Next, a stock solution of 1-chloro-3-fluorobenzene in DMC (294 µL, c = 0.10 M, 29 µmol, 10 equiv.) was added. The resulting reaction mixture was heated at 80 °C for 2 hours. After 2 hours, the reaction mixture was cooled to 23 °C. Next, the DMC was removed under a gentle stream of argon and 294 µL of

DMSO were added to result an in situ formed stock solution of arene-ruthenium complex **C13** (294 µL, c = 0.01 M, assuming quantitative arene coordination to ruthenium).

Under an ambient atmosphere, the stock solution **SD-01** of DNA-AOP-NH_2 (**2**) in water (1.0 µL, c = 2.0 mM, 2.0 nmol, 1.0 equiv.) was added to a 1.5 mL Eppendorf tube, followed by sodium borate buffer (1.0 µL, pH 9.4, c = 0.50 M). To this mixture, 16 µL of DMSO was added and the solution was vortexed for 5 seconds. Next, the freshly prepared stock solution **C13** (2.0 µL, c = 0.01 M, 0.02 µmol, 10 equiv.) in DMSO was added. The resulting reaction mixture was vortexed for 5 seconds, transferred to a thermocycler at 40 °C, and incubated for 16 hours at 800 rpm to yield the DNA-conjugate **SRu-25**. Next, the reaction mixture was diluted with 10 µL of Milli-Q water. To the reaction mixture was added the stock solution of NaCl in water (**SR-06** , 3.0 µL, c = 5.0 M, 10% volume of the total reaction volume), followed by cold ethanol (−20 °C, 99 µL) to precipitate the *N*-arylated ruthenium DNA conjugate **SRu-25**. The Eppendorf tube was placed in a freezer (−20 °C) for at least 1 hour, and then it was centrifuged at 4 °C and 11000 × g for at least 30 minutes. The supernatant was removed and the pellet was dried under air, then dissolved in 20 µL water to obtain the DNA-conjugate **SRu-25** (20 µL, c = 0.10 mM). Then, 1.0 µL of the above solution was diluted to 40 µL with water for LC–MS analysis.

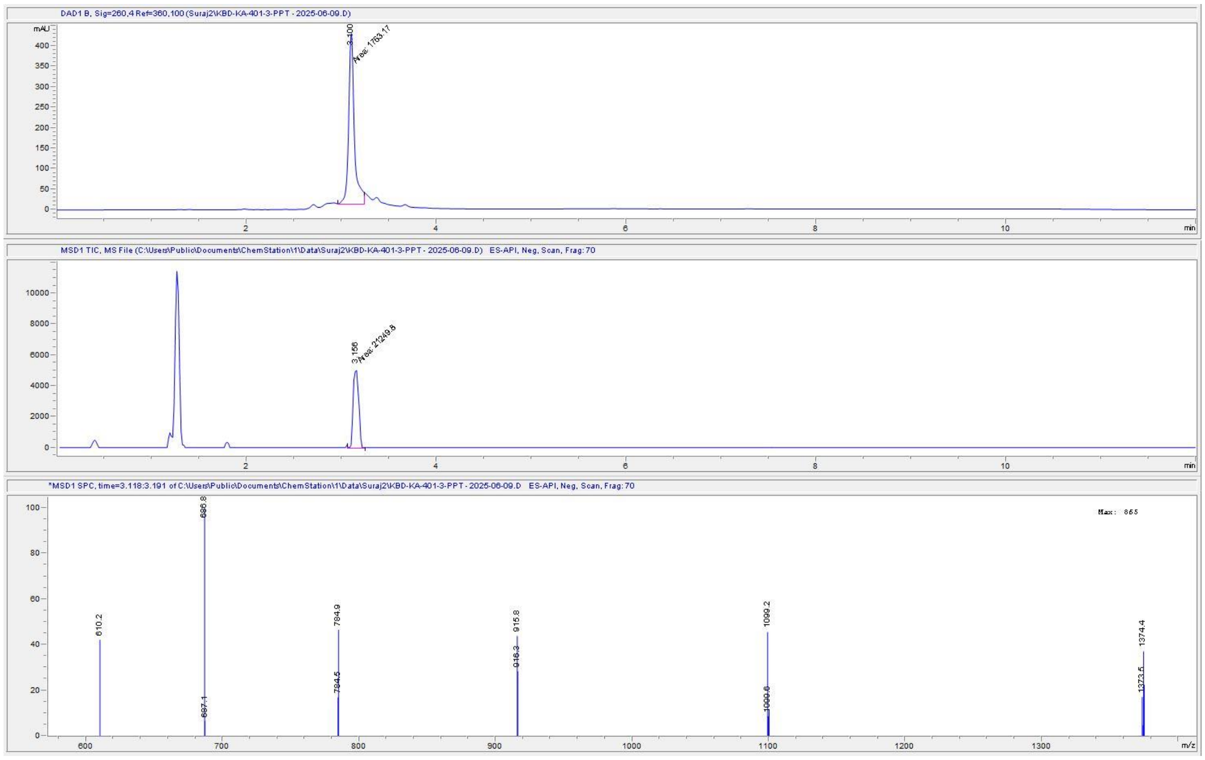

Figure S45. Analytical HPLC trace of **Ru-25** with HPLC method A. (Top) DAD chromatogram at 260 nm. (Middle) TIC chromatogram. (Bottom) Ionization of peak at 3.16 min. containing reaction product.

Decomplexation of SRu-25 to obtain product 25: Under an ambient atmosphere, the DNA-conjugate **SRu-25** stock solution in water (c = 0.10 mM, 20 µL) was irradiated with a 390 nm (40 W) Kessil lamp for 2 hours, while maintaining the temperature at approximately 30 °C through cooling with a fan. To the reaction mixture

was added the stock solution of NaCl in water (**SR-06**, 2.0 µL, c = 5.0 M, 10% volume of the total reaction volume), followed by cold ethanol (−20 °C, 66 µL) to precipitate the DNA conjugate **25**. The Eppendorf tube was placed in the freezer (−20 °C) for at least 1 hour, and then it was centrifuged at 4 °C and 11000 × g for at least 30 minutes. The supernatant was removed, the pellet dried under air and dissolved in Milli-Q water to obtain the purified DNA-conjugate **25**. Then, 1 µL of the above solution was diluted to 40 µL with water for LC–MS analysis. The yield of the DNA conjugate was calculated by measuring the integration of the peaks of the diode array detection (DAD) UV absorbance at 260 nm of the LC–MS trace, assuming complete DNA recovery and identical UV absorbance.

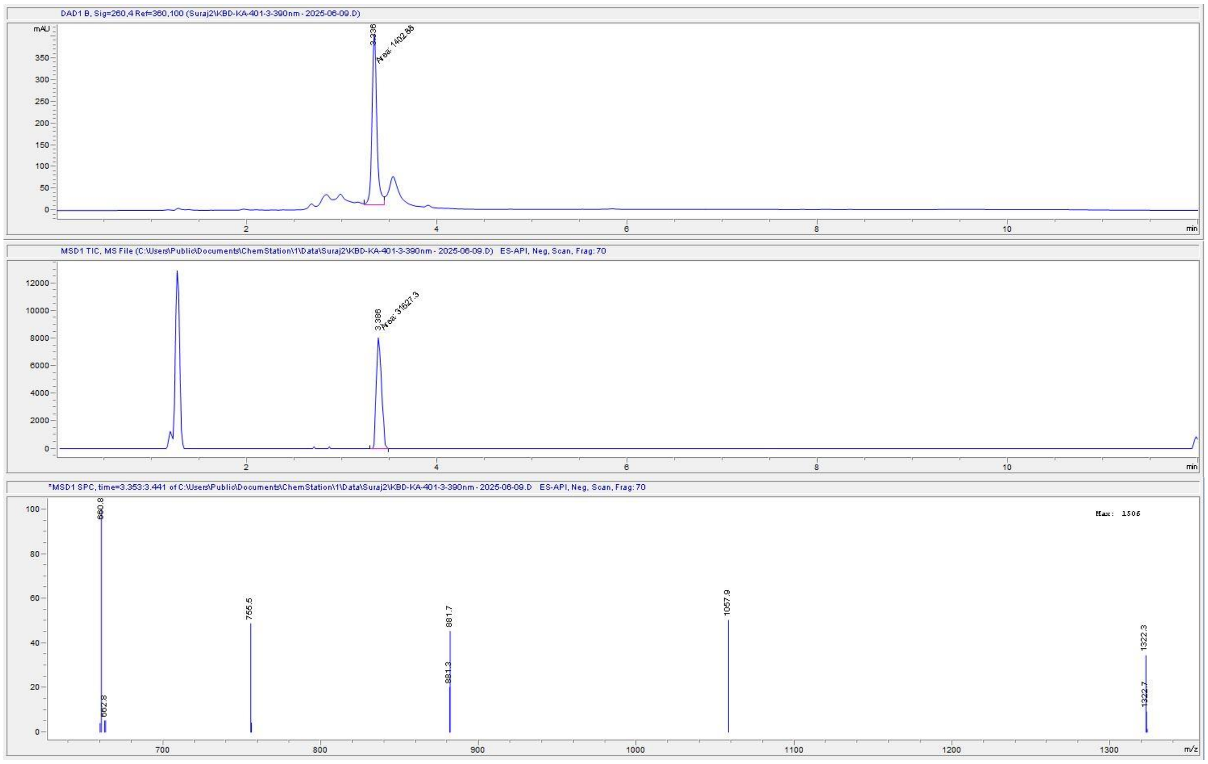

Figure S46. Analytical HPLC trace of **25** with HPLC method B. (Top) DAD chromatogram at 260 nm. (Middle) TIC chromatogram. (Bottom) Ionization of peak at 3.39 min. containing reaction product.

Synthesis of DNA-conjugate 26

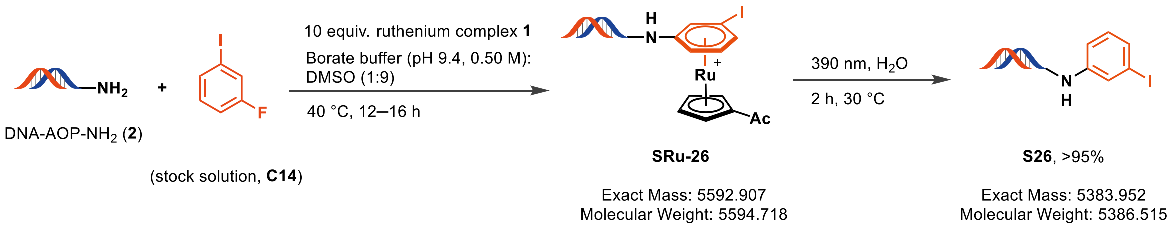

In situ formation of arene-ruthenium stock solution: Under an ambient atmosphere, a 1 mL glass GC vial equipped with a 6 mm Teflon-coated stirring bar was charged with ruthenium complex **1** (1.4 mg, 2.9 µmol, 1.0 equiv.). Next, a stock solution of 1-fluoro-3-iodobenzene in DMC (294 µL, c = 0.10 M, 29 µmol, 10 equiv.)

was added. The resulting reaction mixture was heated at 80 °C for 2 hours. After 2 hours, the reaction mixture was cooled to 23 °C. Next, the DMC was removed under a gentle stream of argon and 294 µL of DMSO were added to result an in situ formed stock solution of arene-ruthenium complex **C14** (294 µL, c = 0.01 M, assuming quantitative arene coordination to ruthenium).

Under an ambient atmosphere, the stock solution **SD-01** of DNA-AOP-NH_2 (**2**) in water (1.0 µL, c = 2.0 mM, 2.0 nmol, 1.0 equiv.) was added to a 1.5 mL Eppendorf tube, followed by sodium borate buffer (1.0 µL, pH 9.4, c = 0.50 M). To this mixture, 16 µL of DMSO was added and the solution was vortexed for 5 seconds. Next, the freshly prepared stock solution **C14** (2.0 µL, c = 0.01 M, 0.02 µmol, 10 equiv.) in DMSO was added. The resulting reaction mixture was vortexed for 5 seconds, transferred to a thermocycler at 40 °C, and incubated for 16 hours at 800 rpm to yield the DNA-conjugate **SRu-26**. Next, the reaction mixture was diluted with 10 µL of Milli-Q water. To the reaction mixture was added the stock solution of NaCl in water (**SR-06** , 3.0 µL, c = 5.0 M, 10% volume of the total reaction volume), followed by cold ethanol (−20 °C, 99 µL) to precipitate the *N*-arylated ruthenium DNA conjugate **SRu-26**. The Eppendorf tube was placed in a freezer (−20 °C) for at least 1 hour, and then it was centrifuged at 4 °C and 11000 × g for at least 30 minutes. The supernatant was removed and the pellet was dried under air, then dissolved in 20 µL water to obtain the DNA-conjugate **SRu-26** (20 µL, c = 0.10 mM). Then, 1.0 µL of the above solution was diluted to 40 µL with water for LC–MS analysis.

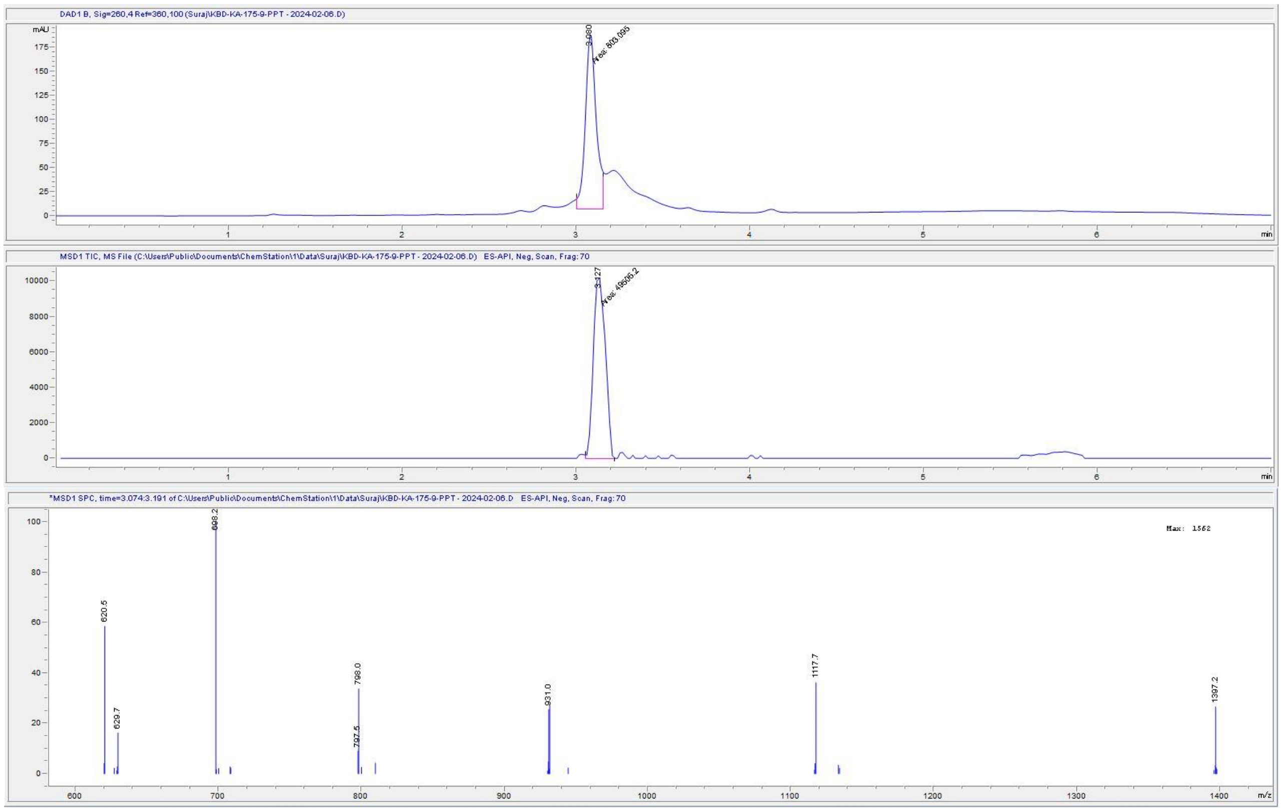

Figure S47. Analytical HPLC trace of **SRu-26** with HPLC method B. (Top) DAD chromatogram at 260 nm. (Middle) TIC chromatogram. (Bottom) Ionization of peak at 3.13 min. containing reaction product.

Decomplexation of SRu-26 to obtain product 26: Under an ambient atmosphere, the DNA-conjugate **SRu-26** stock solution in water (c = 0.10 mM, 20 µL) was irradiated with a 390 nm (40 W) Kessil lamp for 2 hours, while maintaining the temperature at approximately 30 °C through cooling with a fan. To the reaction mixture was added the stock solution of NaCl in water (**SR-06**, 2.0 µL, c = 5.0 M, 10% volume of the total reaction volume), followed by cold ethanol (−20 °C, 66 µL) to precipitate the DNA conjugate **26**. The Eppendorf tube was placed in the freezer (−20 °C) for at least 1 hour, and then it was centrifuged at 4 °C and 11000 × g for at least 30 minutes. The supernatant was removed, the pellet dried under air and dissolved in Milli-Q water to obtain the purified DNA-conjugate **26**. Then, 1 µL of the above solution was diluted to 40 µL with water for LC–MS analysis. The yield of the DNA conjugate was calculated by measuring the integration of the peaks of the diode array detection (DAD) UV absorbance at 260 nm of the LC–MS trace, assuming complete DNA recovery and identical UV absorbance.

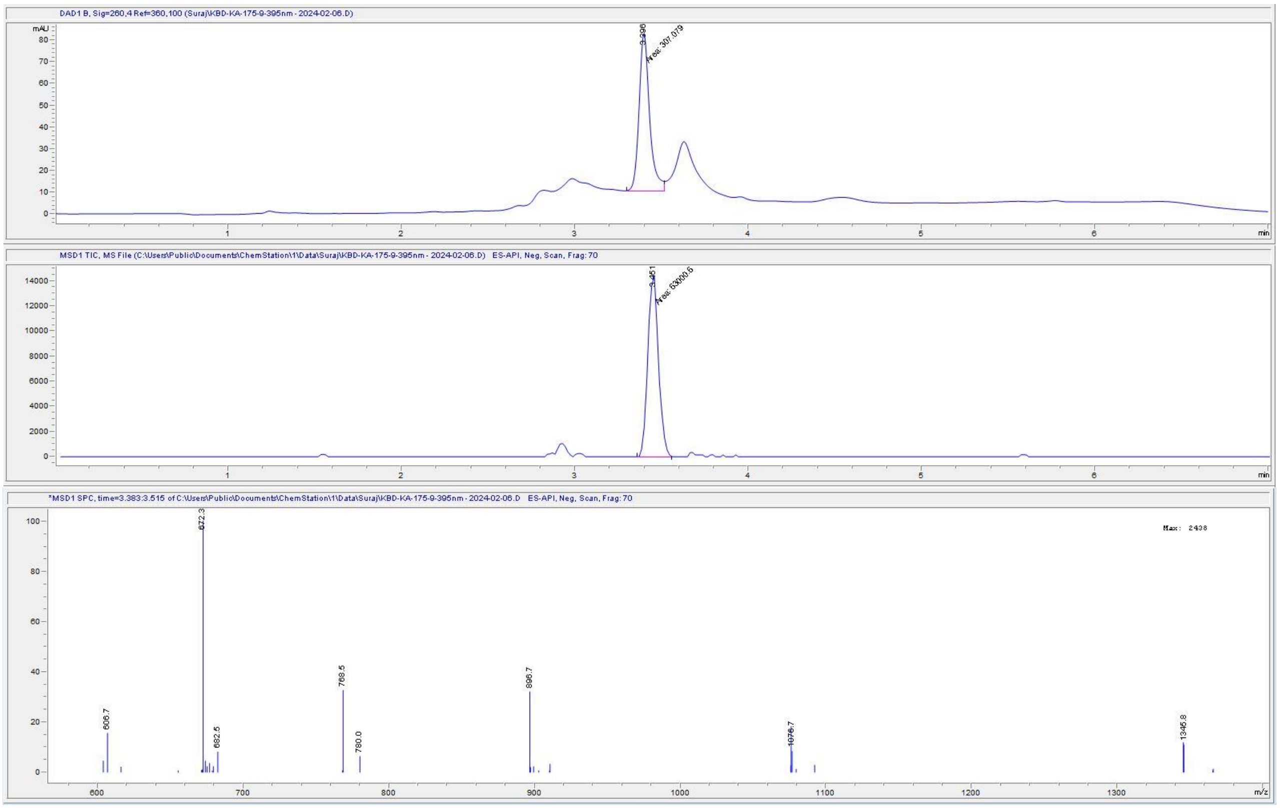

Figure S48. Analytical HPLC trace of **26** with HPLC method B. (Top) DAD chromatogram at 260 nm. (Middle) TIC chromatogram. (Bottom) Ionization of peak at 3.45 min. containing reaction product.

Synthesis of DNA-conjugate 27

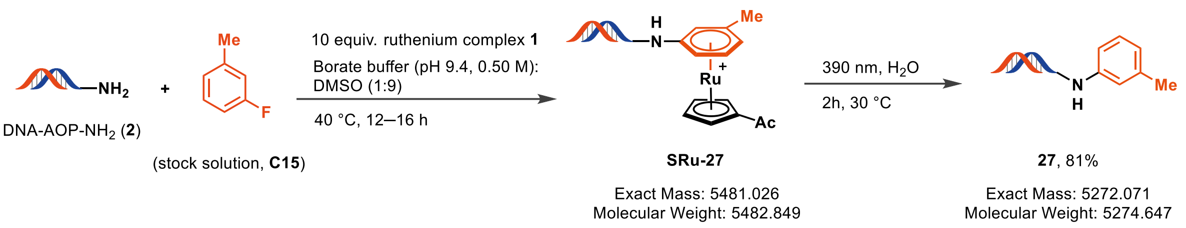

In situ formation of arene-ruthenium stock solution: Under an ambient atmosphere, a 1 mL glass GC vial equipped with a 6 mm Teflon-coated stirring bar was charged with ruthenium complex **1** (1.4 mg, 2.9 µmol, 1.0 equiv.). Next, a stock solution of 1-fluoro-3-methylbenzene in DMC (294 µL, c = 0.10 M, 29 µmol, 10 equiv.) was added. The resulting reaction mixture was heated at 80 °C for 2 hours. After 2 hours, the reaction mixture was cooled to 23 °C. Next, the DMC was removed under a gentle stream of argon and 294 µL of DMSO were added to result an in situ formed stock solution of arene-ruthenium complex **C15** (294 µL, c = 0.01 M, assuming quantitative arene coordination to ruthenium).

Under an ambient atmosphere, the stock solution **SD-01** of DNA-AOP-NH_2 (**2**) in water (1.0 µL, c = 2.0 mM, 2.0 nmol, 1.0 equiv.) was added to a 1.5 mL Eppendorf tube, followed by sodium borate buffer (1.0 µL, pH 9.4, c = 0.50 M). To this mixture, 16 µL of DMSO was added and the solution was vortexed for 5 seconds. Next, the freshly prepared stock solution **C15** (2.0 µL, c = 0.01 M, 0.02 µmol, 10 equiv.) in DMSO was added. The resulting reaction mixture was vortexed for 5 seconds, transferred to a thermocycler at 40 °C, and incubated for 16 hours at 800 rpm to yield the DNA-conjugate **SRu-27**. Next, the reaction mixture was diluted with 10 µL of Milli-Q water. To the reaction mixture was added the stock solution of NaCl in water (**SR-06** , 3.0 µL, c = 5.0 M, 10% volume of the total reaction volume), followed by cold ethanol (−20 °C, 99 µL) to precipitate the *N*-arylated ruthenium DNA conjugate **SRu-27**. The Eppendorf tube was placed in a freezer (−20 °C) for at least 1 hour, and then it was centrifuged at 4 °C and 11000 × g for at least 30 minutes. The supernatant was removed and the pellet was dried under air, then dissolved in 20 µL water to obtain the DNA-conjugate **SRu-27** (20 µL, c = 0.10 mM). Then, 1.0 µL of the above solution was diluted to 40 µL with water for LC–MS analysis.

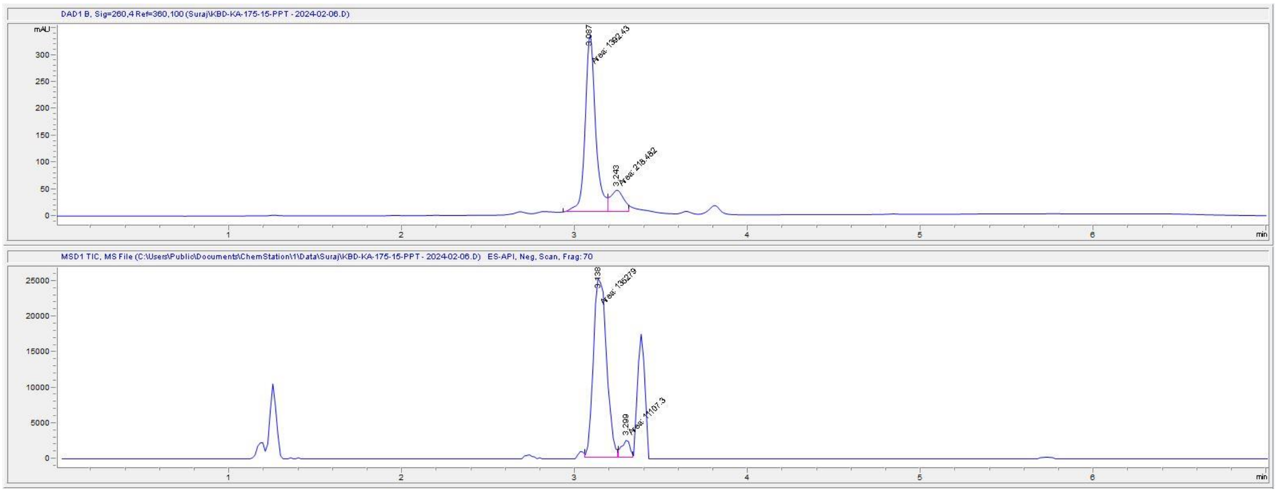

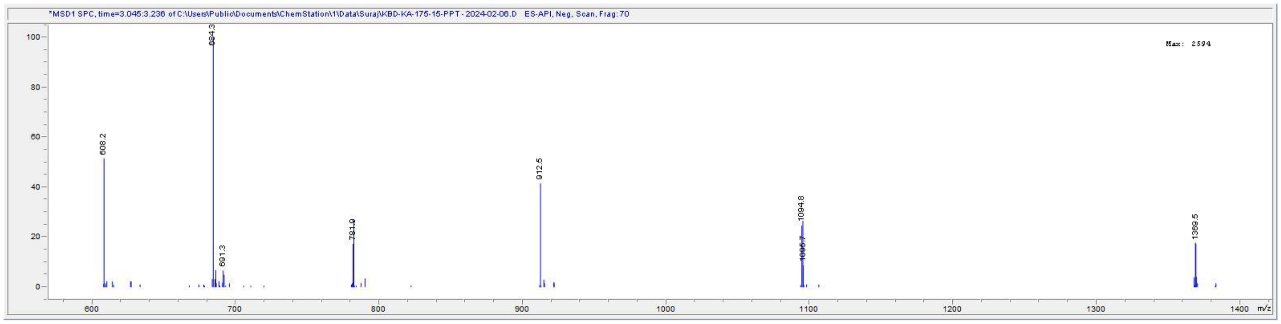

Figure S49. Analytical HPLC trace of **SRu-27** with HPLC method B. (Top) DAD chromatogram at 260 nm. (Middle) TIC chromatogram. (Bottom) Ionization of peak at 3.14 min. containing reaction product.

Decomplexation of SRu-27 to obtain product 27: Under an ambient atmosphere, the DNA-conjugate **SRu-27** stock solution in water (c = 0.10 mM, 20 µL) was irradiated with a 390 nm (40 W) Kessil lamp for 2 hours, while maintaining the temperature at approximately 30 °C through cooling with a fan. To the reaction mixture was added the stock solution of NaCl in water (**SR-06**, 2.0 µL, c = 5.0 M, 10% volume of the total reaction volume), followed by cold ethanol (−20 °C, 66 µL) to precipitate the DNA conjugate **27**. The Eppendorf tube was placed in the freezer (−20 °C) for at least 1 hour, and then it was centrifuged at 4 °C and 11000 × g for at least 30 minutes. The supernatant was removed, the pellet dried under air and dissolved in Milli-Q water to obtain the purified DNA-conjugate **27**. Then, 1 µL of the above solution was diluted to 40 µL with water for LC–MS analysis. The yield of the DNA conjugate was calculated by measuring the integration of the peaks of the diode array detection (DAD) UV absorbance at 260 nm of the LC–MS trace, assuming complete DNA recovery and identical UV absorbance.

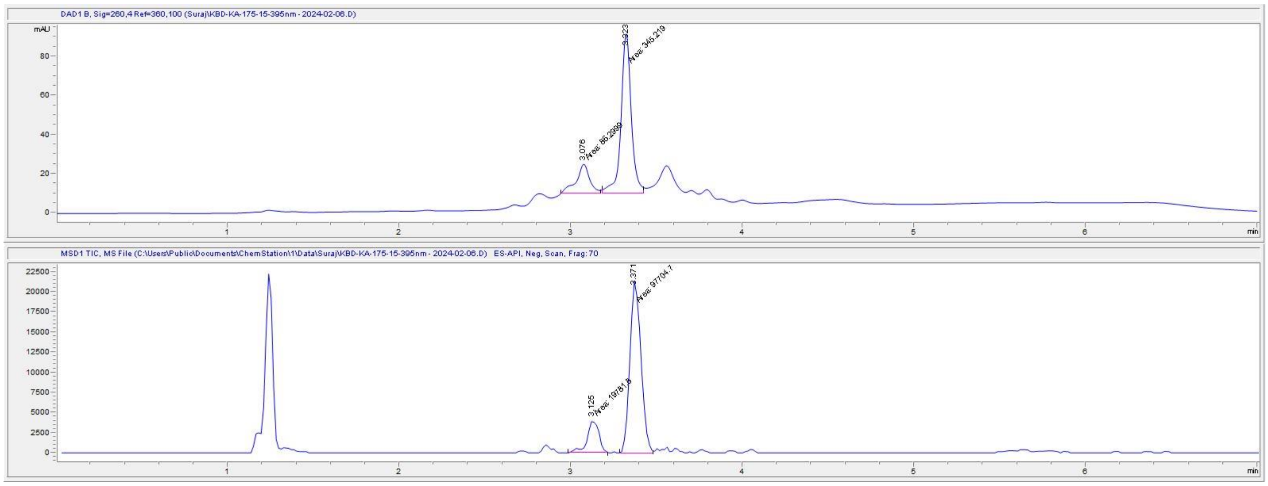

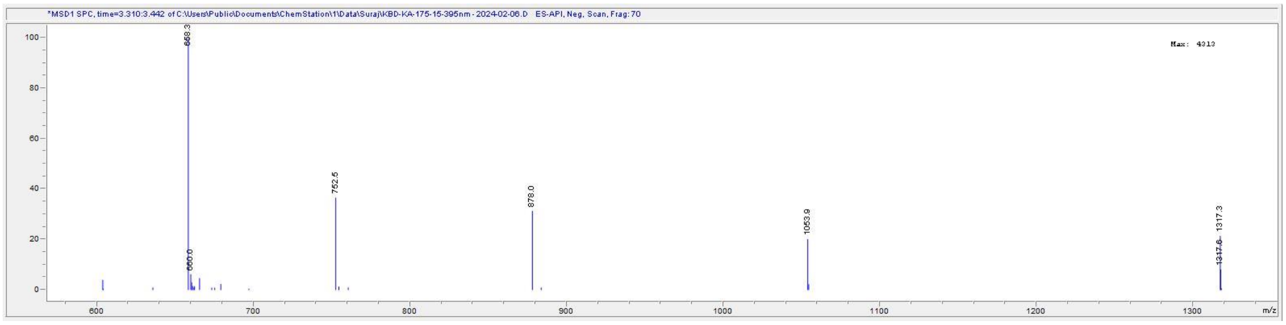

Figure S50. Analytical HPLC trace of **27** with HPLC method B. (Top) DAD chromatogram at 260 nm. (Middle) TIC chromatogram. (Bottom) Ionization of peak at 3.37 min. containing reaction product.

Synthesis of DNA-conjugate 28

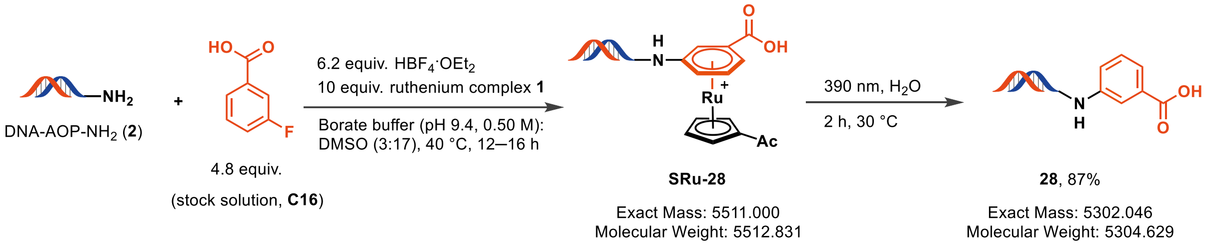

SRu-28

Exact Mass: 5511.000
Molecular Weight: 5512.831

28, 87%

Exact Mass: 5302.046
Molecular Weight: 5304.629

In situ formation of fluoroarene-ruthenium stock solution: Under an ambient atmosphere, a 1 mL glass GC vial equipped with a 6 mm Teflon-coated stirring bar was charged with ruthenium complex **1** (1.0 mg, 2.1 µmol, 1.0 equiv.) and then 100 µL of DMC was added to the reaction vial. In a separate Eppendorf tube was added a stock solution of $HBF_4 \cdot OEt_2$ (50 µL, c = 0.26 M, 13 µmol, 6.2 equiv.), followed by a stock solution of 3-fluorobenzoic acid in DMC (50 µL, c = 0.20 M, 10 µmol, 4.8 equiv.). The contents of the Eppendorf tube was then transferred to the reaction vial. The vial was then closed with a screw cap and the resulting reaction mixture was heated at 80 °C for 2 hours. After 2 hours, the reaction mixture was cooled to 23 °C and the DMC was removed under a gentle stream of argon and 200 µL of DMSO was added to result in an in situ formed stock solution of arene-ruthenium complex **C16** in DMSO (200 µL, c = 0.01 M, assuming quantitative arene coordination to ruthenium).

Under an ambient atmosphere, the stock solution **SD-01** of DNA-AOP-NH_2 (**2**) (1.0 µL, c = 2.0 mM, 2.0 nmol, 1.0 equiv.) in water was added to a 1.5 mL Eppendorf tube, followed by sodium borate buffer (2.0 µL, c = 0.50 M). To this mixture, 15 µL of DMSO was added and the solution was vortexed for 5 seconds. Next, the freshly prepared stock solution **C16** (2.0 µL, c = 0.01 M, 0.02 µmol, 10 equiv.) in DMSO was added. The resulting reaction mixture was vortexed for 5 seconds, transferred to a thermocycler at 40 °C, and incubated for 16 hours at 800 rpm to yield the DNA-conjugate **SRu-28**. To the reaction mixture was added the stock solution of NaCl in water (**SR-06** , 2.0 µL, c = 5.0 M, 10% volume of the total reaction volume), followed by cold ethanol (−20 °C, 66 µL) to precipitate the *N*-arylated ruthenium DNA conjugate **SRu-28**. The Eppendorf tube was placed in a freezer (−20 °C) for at least 1 hour, and then it was centrifuged at 4 °C and 11000 × g for at least 30 minutes. The supernatant was removed and the pellet was dried under air, then dissolved in 20

µL water to obtain the DNA-conjugate **SRu-28** (10 µL, c = 0.10 mM). Then, 1.0 µL of the above solution was diluted to 40 µL with water for LC–MS analysis.

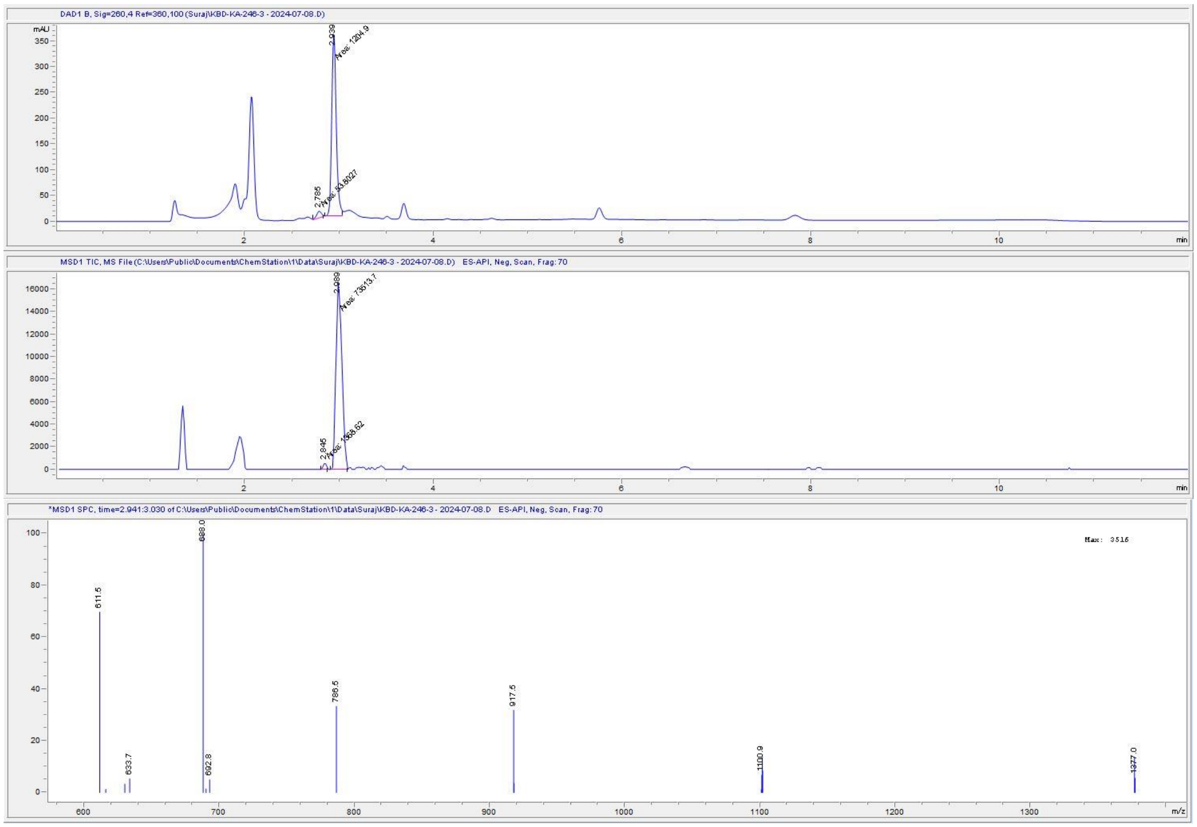

Figure S51. Analytical HPLC trace of **SRu-28** with HPLC method A. (Top) DAD chromatogram at 260 nm. (Middle) TIC chromatogram. (Bottom) Ionization of peak at 2.99 min containing reaction product.

Decomplexation of SRu-28 to obtain product 28: Under an ambient atmosphere, the DNA-conjugate **SRu-28** stock solution in water (c = 0.10 mM, 20 µL) was irradiated with a 390 nm (40 W) Kessil lamp for 2 hours, while maintaining the temperature at approximately 30 °C through cooling with a fan. To the reaction mixture was added the stock solution of NaCl in water (**SR-06**, 2.0 µL, c = 5.0 M, 10% volume of the total reaction volume), followed by cold ethanol (−20 °C, 66 µL) to precipitate the DNA conjugate **28**. The Eppendorf tube was placed in the freezer (−20 °C) for at least 1 hour, and then it was centrifuged at 4 °C and 11000 × g for at least 30 minutes. The supernatant was removed, the pellet dried under air and dissolved in Milli-Q water to obtain the purified DNA-conjugate **28**. Then, 1 µL of the above solution was diluted to 40 µL with water for LC–MS analysis. The yield of the DNA conjugate was calculated by measuring the integration of the peaks of the diode array detection (DAD) UV absorbance at 260 nm of the LC–MS trace, assuming complete DNA recovery and identical UV absorbance.

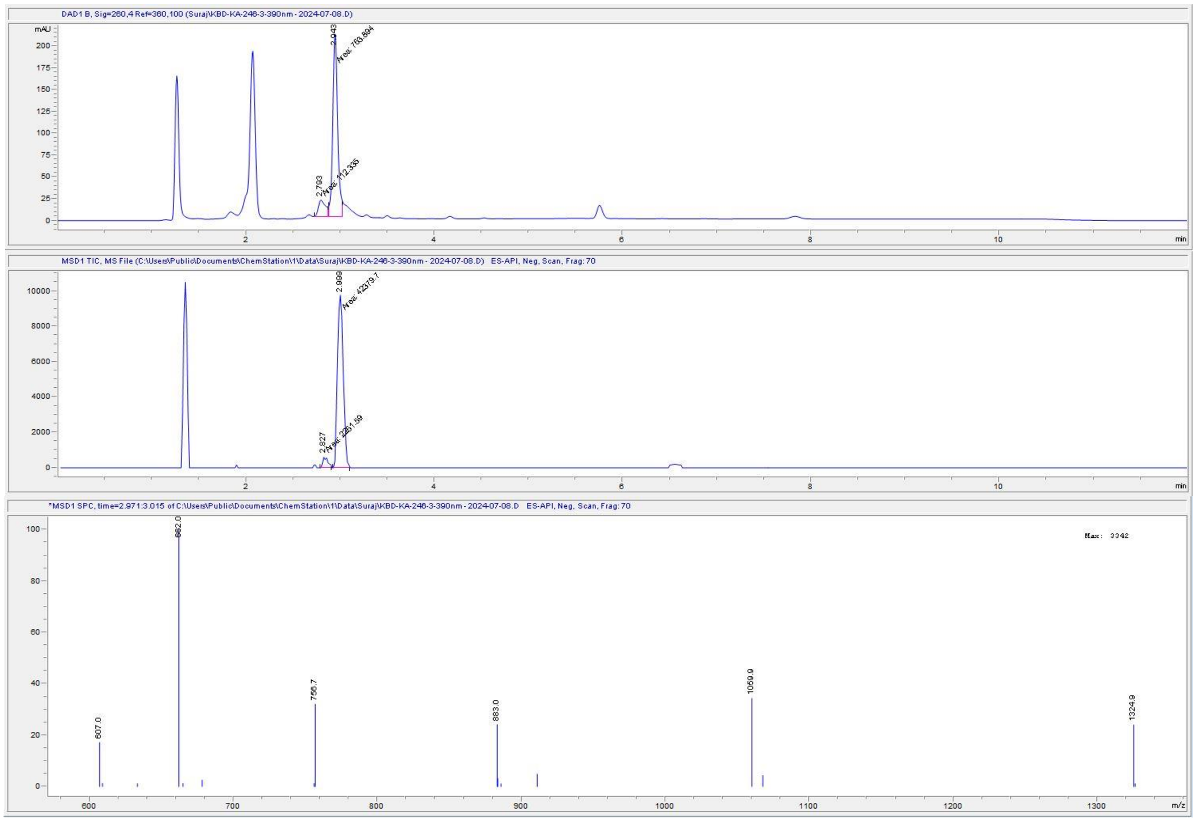

Figure S52. Analytical HPLC trace of **28** with HPLC method A. (Top) DAD chromatogram at 260 nm. (Middle) TIC chromatogram. (Bottom) Ionization of peak at 3.00 min. containing reaction product.

Synthesis of DNA-conjugate 29

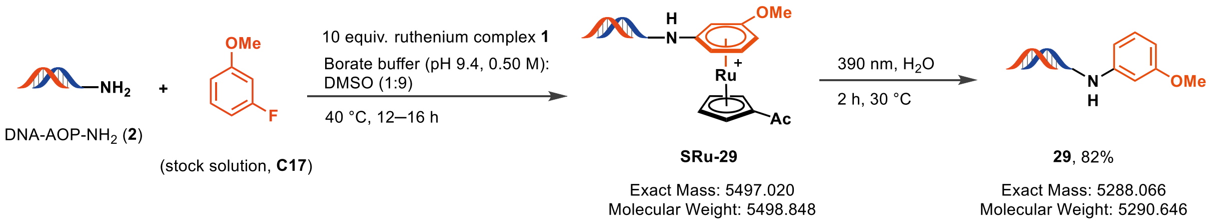

In situ formation of arene-ruthenium stock solution: Under an ambient atmosphere, a 1 mL glass GC vial equipped with a 6 mm Teflon-coated stirring bar was charged with ruthenium complex **1** (1.4 mg, 2.9 µmol, 1.0 equiv.). Next, a stock solution of 1-fluoro-3-methoxybenzene in DMC (294 µL, c = 0.10 M, 29 µmol, 10 equiv.) was added. The resulting reaction mixture was heated at 80 °C for 2 hours. After 2 hours, the reaction mixture was cooled to 23 °C. Next, the DMC was removed under a gentle stream of argon and 294 µL of DMSO were added to result an in situ formed stock solution of arene-ruthenium complex **C17** (294 µL, c = 0.01 M, assuming quantitative arene coordination to ruthenium).

Under an ambient atmosphere, the stock solution **SD-01** of DNA-AOP-NH$_2$ (**2**) in water (1.0 µL, c = 2.0 mM, 2.0 nmol, 1.0 equiv.) was added to a 1.5 mL Eppendorf tube, followed by sodium borate buffer (1.0 µL, pH 9.4, c = 0.50 M). To this mixture, 16 µL of DMSO was added and the solution was vortexed for 5 seconds.

Next, the freshly prepared stock solution **C17** (2.0 µL, c = 0.01 M, 0.02 µmol, 10 equiv.) in DMSO was added. The resulting reaction mixture was vortexed for 5 seconds, transferred to a thermocycler at 40 °C, and incubated for 16 hours at 800 rpm to yield the DNA-conjugate **SRu-29**. Next, the reaction mixture was diluted with 10 µL of Milli-Q water. To the reaction mixture was added the stock solution of NaCl in water (**SR-06** , 3.0 µL, c = 5.0 M, 10% volume of the total reaction volume), followed by cold ethanol (−20 °C, 99 µL) to precipitate the *N*-arylated ruthenium DNA conjugate **SRu-29**. The Eppendorf tube was placed in a freezer (−20 °C) for at least 1 hour, and then it was centrifuged at 4 °C and 11000 × g for at least 30 minutes. The supernatant was removed and the pellet was dried under air, then dissolved in 20 µL water to obtain the DNA-conjugate **SRu-29** (20 µL, c = 0.10 mM). Then, 1.0 µL of the above solution was diluted to 40 µL with water for LC–MS analysis.

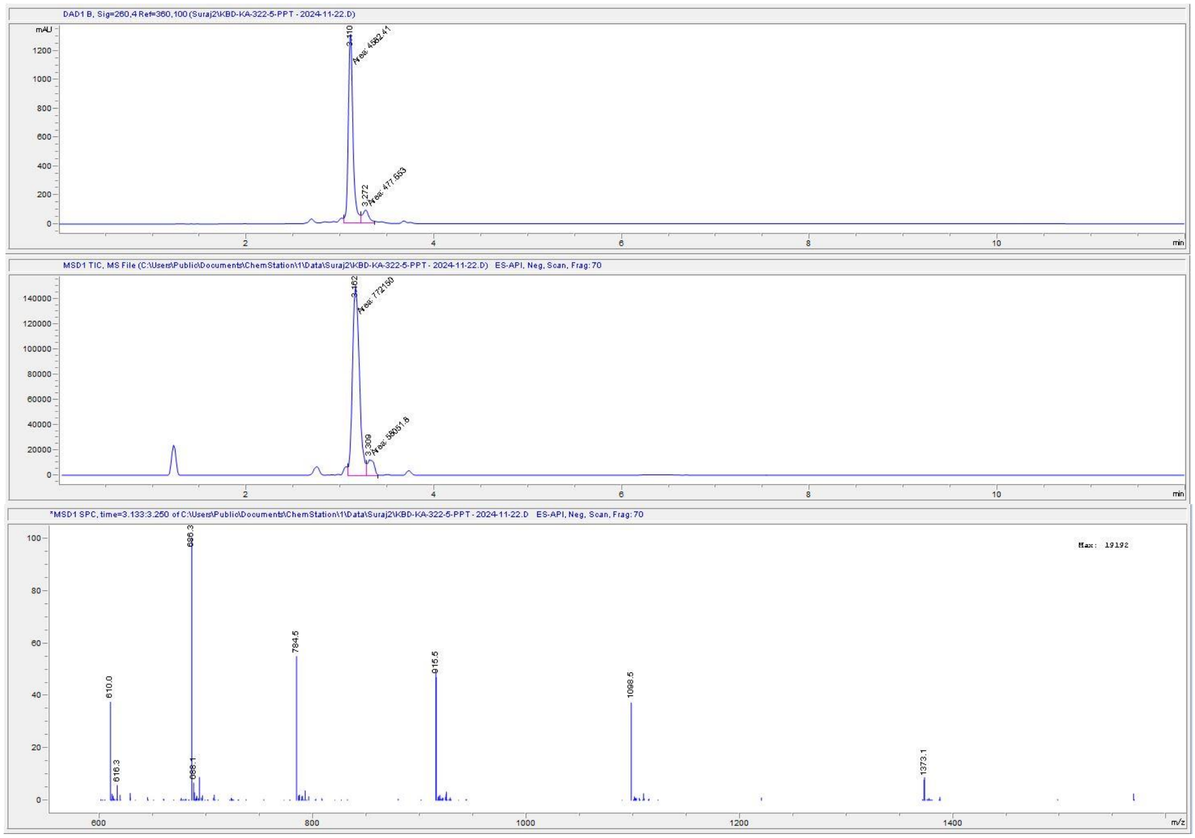

Figure S53. Analytical HPLC trace of **SRu-29** with HPLC method A. (Top) DAD chromatogram at 260 nm. (Middle) TIC chromatogram. (Bottom) Ionization of peak at 3.16 min. containing reaction product.

Decomplexation of SRu-29 to obtain product 29: Under an ambient atmosphere, the DNA-conjugate **SRu-29** stock solution in water (c = 0.10 mM, 20 µL) was irradiated with a 390 nm (40 W) Kessil lamp for 2 hours, while maintaining the temperature at approximately 30 °C through cooling with a fan. To the reaction mixture was added the stock solution of NaCl in water (**SR-06**, 2.0 µL, c = 5.0 M, 10% volume of the total reaction volume), followed by cold ethanol (−20 °C, 66 µL) to precipitate the DNA conjugate **29**. The Eppendorf tube was placed in the freezer (−20 °C) for at least 1 hour, and then it was centrifuged at 4 °C and 11000 × g for at

least 30 minutes. The supernatant was removed, the pellet dried under air and dissolved in Milli-Q water to obtain the purified DNA-conjugate **29**. Then, 1 µL of the above solution was diluted to 40 µL with water for LC–MS analysis. The yield of the DNA conjugate was calculated by measuring the integration of the peaks of the diode array detection (DAD) UV absorbance at 260 nm of the LC–MS trace, assuming complete DNA recovery and identical UV absorbance.

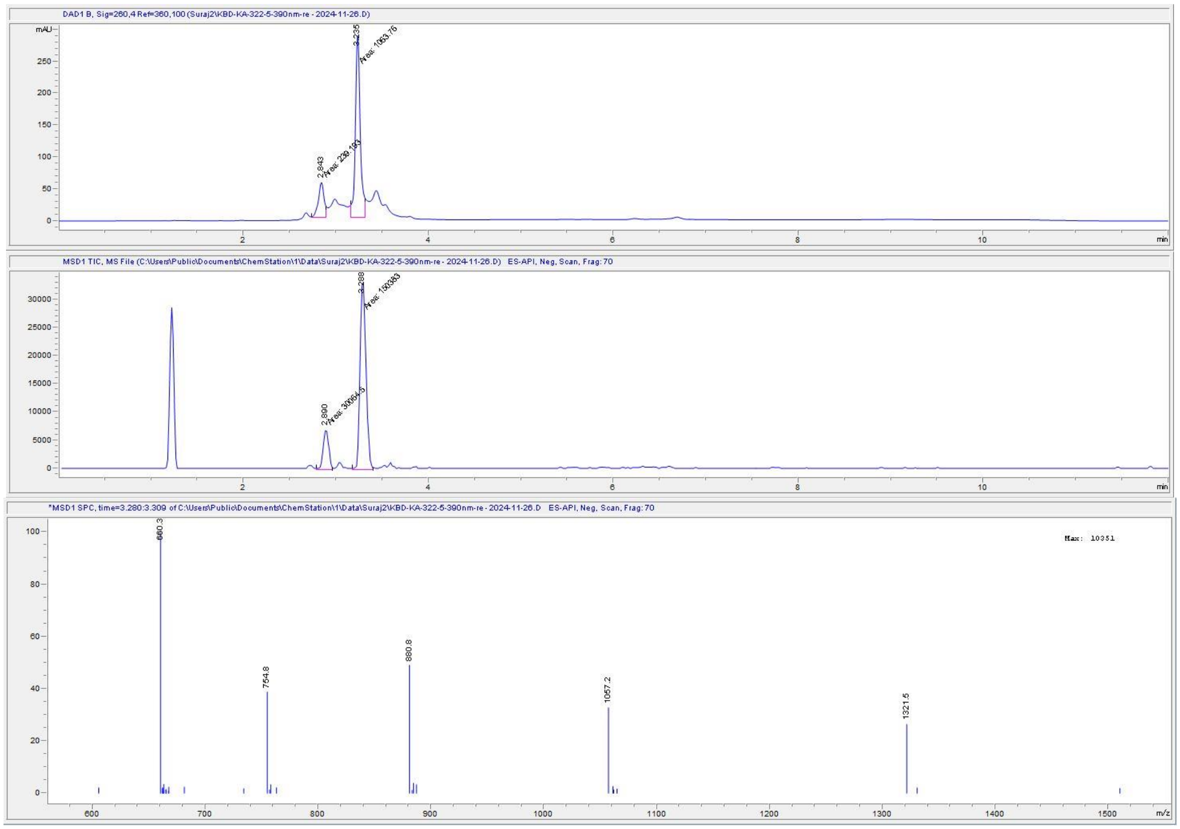

Figure S54. Analytical HPLC trace of **29** with HPLC method A. (Top) DAD chromatogram at 260 nm. (Middle) TIC chromatogram. (Bottom) Ionization of peak at 3.29 min. containing reaction product.

Synthesis of DNA-conjugate 30

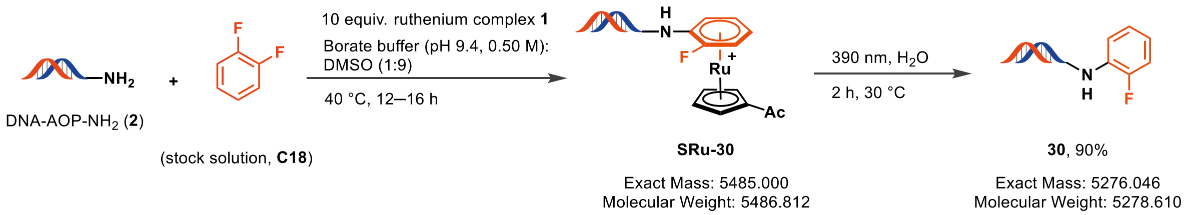

In situ formation of arene-ruthenium stock solution: Under an ambient atmosphere, a 1 mL glass GC vial equipped with a 6 mm Teflon-coated stirring bar was charged with ruthenium complex **1** (1.4 mg, 2.9 µmol, 1.0 equiv.). Next, a stock solution of 1,2-difluorobenzene in DMC (294 µL, c = 0.10 M, 29 µmol, 10 equiv.) was added. The resulting reaction mixture was heated at 80 °C for 2 hours. After 2 hours, the reaction mixture was cooled to 23 °C. Next, the DMC was removed under a gentle stream of argon and 294 µL of

DMSO were added to result an in situ formed stock solution of arene-ruthenium complex **C18** (294 µL, c = 0.01 M, assuming quantitative arene coordination to ruthenium).

Under an ambient atmosphere, the stock solution **SD-01** of DNA-AOP-NH_2 (**2**) in water (1.0 µL, c = 2.0 mM, 2.0 nmol, 1.0 equiv.) was added to a 1.5 mL Eppendorf tube, followed by sodium borate buffer (1.0 µL, pH 9.4, c = 0.50 M). To this mixture, 16 µL of DMSO was added and the solution was vortexed for 5 seconds. Next, the freshly prepared stock solution **C18** (2.0 µL, c = 0.01 M, 0.02 µmol, 10 equiv.) in DMSO was added. The resulting reaction mixture was vortexed for 5 seconds, transferred to a thermocycler at 40 °C, and incubated for 16 hours at 800 rpm to yield the DNA-conjugate **SRu-30**. Next, the reaction mixture was diluted with 10 µL of Milli-Q water. To the reaction mixture was added the stock solution of NaCl in water (**SR-06** , 3.0 µL, c = 5.0 M, 10% volume of the total reaction volume), followed by cold ethanol (−20 °C, 99 µL) to precipitate the *N*-arylated ruthenium DNA conjugate **SRu-30**. The Eppendorf tube was placed in a freezer (−20 °C) for at least 1 hour, and then it was centrifuged at 4 °C and 11000 × g for at least 30 minutes. The supernatant was removed and the pellet was dried under air, then dissolved in 20 µL water to obtain the DNA-conjugate **SRu-30** (20 µL, c = 0.10 mM). Then, 1.0 µL of the above solution was diluted to 40 µL with water for LC–MS analysis.

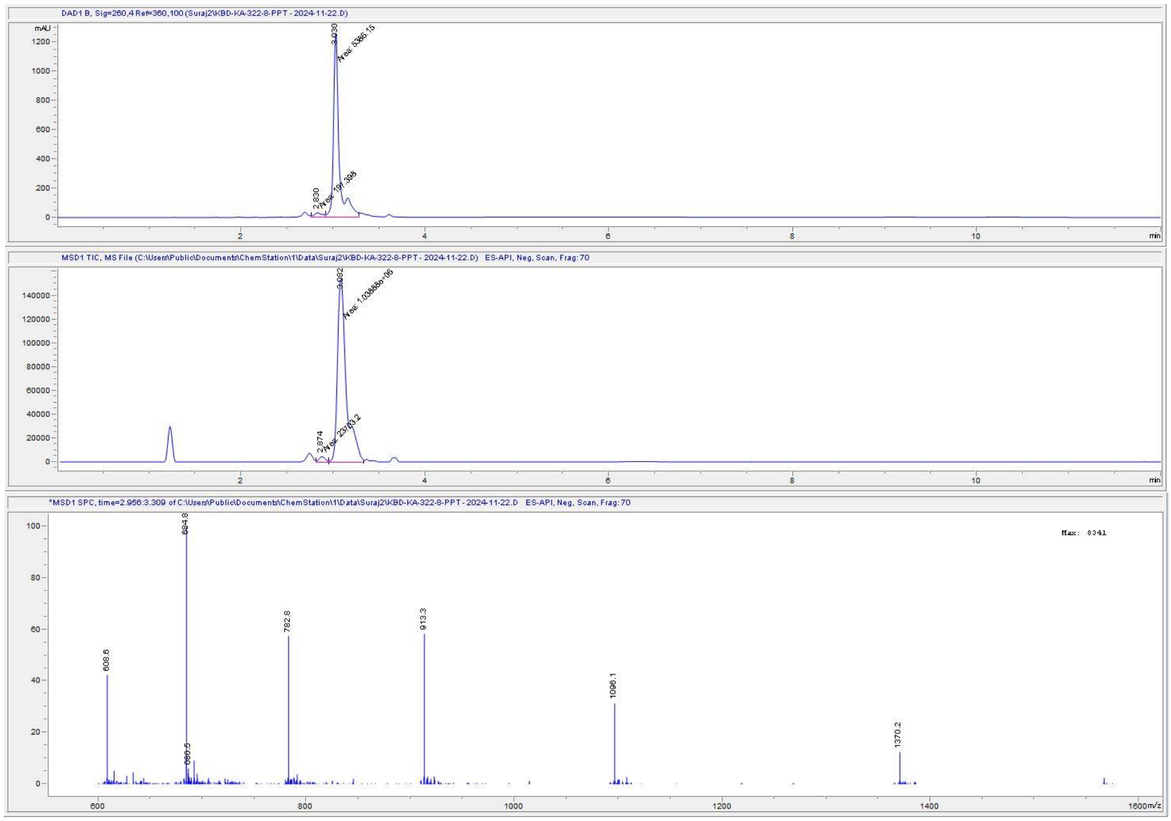

Figure S55. Analytical HPLC trace of **SRu-30** with HPLC method B. (Top) DAD chromatogram at 260 nm. (Middle) TIC chromatogram. (Bottom) Ionization of peak at 3.27 min. containing reaction product.

Decomplexation of SRu-30 to obtain product 30: Under an ambient atmosphere, the DNA-conjugate **SRu-**

30 stock solution in water (c = 0.10 mM, 20 µL) was irradiated with a 390 nm (40 W) Kessil lamp for 2 hours, while maintaining the temperature at approximately 30 °C through cooling with a fan. To the reaction mixture was added the stock solution of NaCl in water (**SR-06**, 2.0 µL, c = 5.0 M, 10% volume of the total reaction volume), followed by cold ethanol (−20 °C, 66 µL) to precipitate the DNA conjugate **30**. The Eppendorf tube was placed in the freezer (−20 °C) for at least 1 hour, and then it was centrifuged at 4 °C and 11000 × g for at least 30 minutes. The supernatant was removed, the pellet dried under air and dissolved in Milli-Q water to obtain the purified DNA-conjugate **30**. Then, 1 µL of the above solution was diluted to 40 µL with water for LC–MS analysis. The yield of the DNA conjugate was calculated by measuring the integration of the peaks of the diode array detection (DAD) UV absorbance at 260 nm of the LC–MS trace, assuming complete DNA recovery and identical UV absorbance.

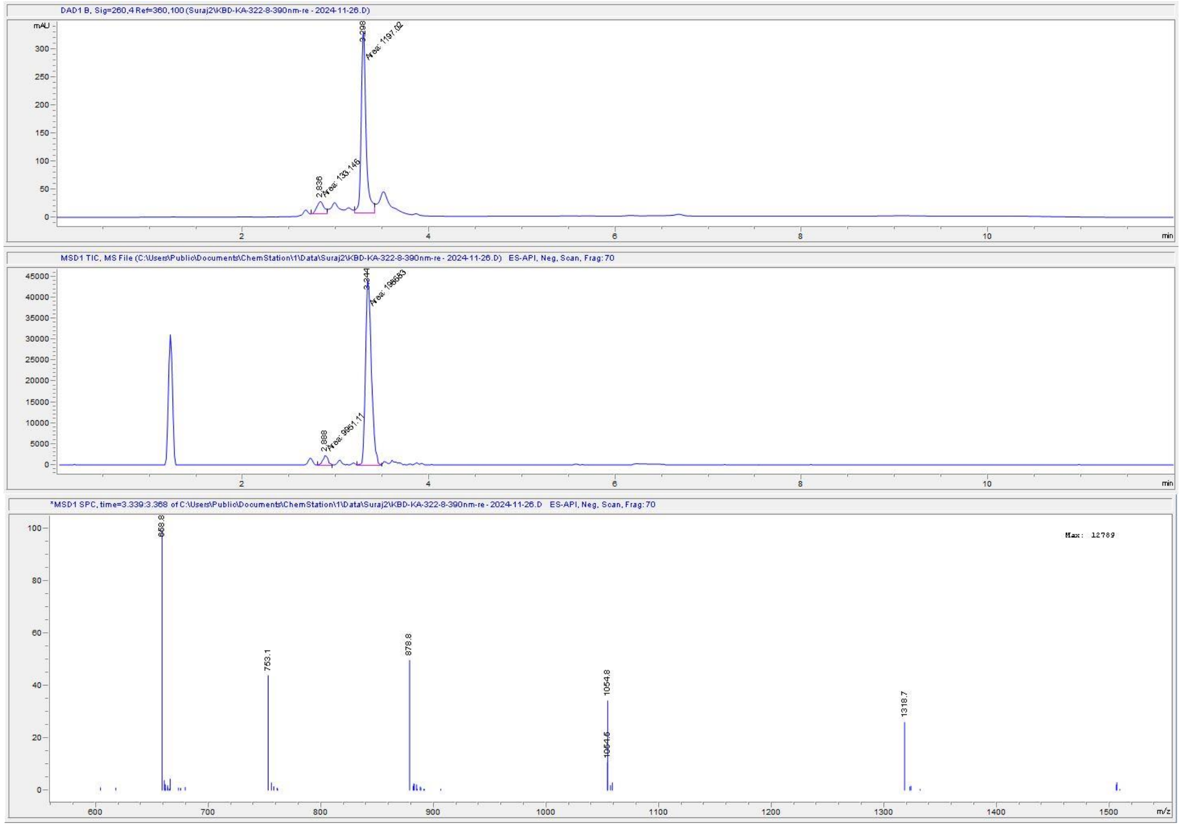

Figure S56. Analytical HPLC trace of **30** with HPLC method A. (Top) DAD chromatogram at 260 nm. (Middle) TIC chromatogram. (Bottom) Ionization of peak at 3.34 min. containing reaction product.

Synthesis of DNA-conjugate 31

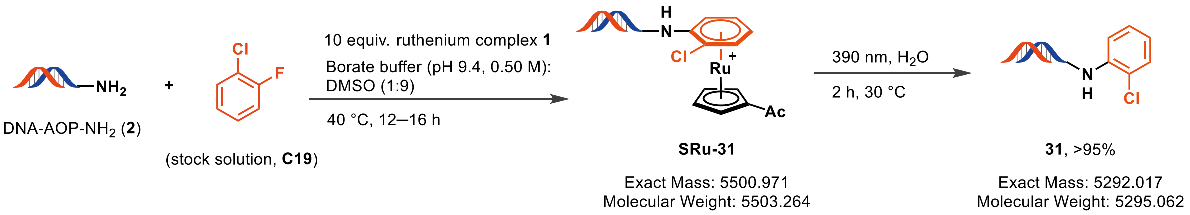

In situ formation of arene-ruthenium stock solution: Under an ambient atmosphere, a 1 mL glass GC vial equipped with a 6 mm Teflon-coated stirring bar was charged with ruthenium complex **1** (1.4 mg, 2.9 µmol, 1.0 equiv.). Next, a stock solution of 1-chloro-2-fluorobenzene in DMC (294 µL, c = 0.10 M, 29 µmol, 10 equiv.) was added. The resulting reaction mixture was heated at 80 °C for 2 hours. After 2 hours, the reaction mixture was cooled to 23 °C. Next, the DMC was removed under a gentle stream of argon and 294 µL of DMSO were added to result an in situ formed stock solution of arene-ruthenium complex **C19** (294 µL, c = 0.01 M, assuming quantitative arene coordination to ruthenium).

Under an ambient atmosphere, the stock solution **SD-01** of DNA-AOP-NH_2 (**2**) in water (1.0 µL, c = 2.0 mM, 2.0 nmol, 1.0 equiv.) was added to a 1.5 mL Eppendorf tube, followed by sodium borate buffer (1.0 µL, pH 9.4, c = 0.50 M). To this mixture, 16 µL of DMSO was added and the solution was vortexed for 5 seconds. Next, the freshly prepared stock solution **C19** (2.0 µL, c = 0.01 M, 0.02 µmol, 10 equiv.) in DMSO was added. The resulting reaction mixture was vortexed for 5 seconds, transferred to a thermocycler at 40 °C, and incubated for 16 hours at 800 rpm to yield the DNA-conjugate **SRu-31**. Next, the reaction mixture was diluted with 10 µL of Milli-Q water. To the reaction mixture was added the stock solution of NaCl in water (**SR-06** , 3.0 µL, c = 5.0 M, 10% volume of the total reaction volume), followed by cold ethanol (−20 °C, 99 µL) to precipitate the *N*-arylated ruthenium DNA conjugate **SRu-31**. The Eppendorf tube was placed in a freezer (−20 °C) for at least 1 hour, and then it was centrifuged at 4 °C and 11000 × g for at least 30 minutes. The supernatant was removed and the pellet was dried under air, then dissolved in 20 µL water to obtain the DNA-conjugate **SRu-31** (20 µL, c = 0.10 mM). Then, 1.0 µL of the above solution was diluted to 40 µL with water for LC–MS analysis.

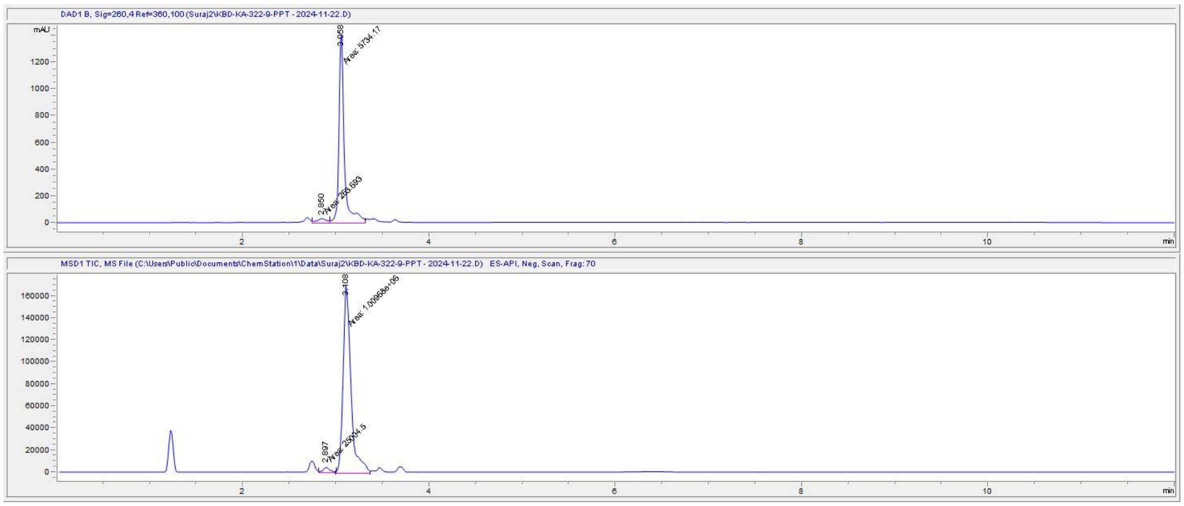

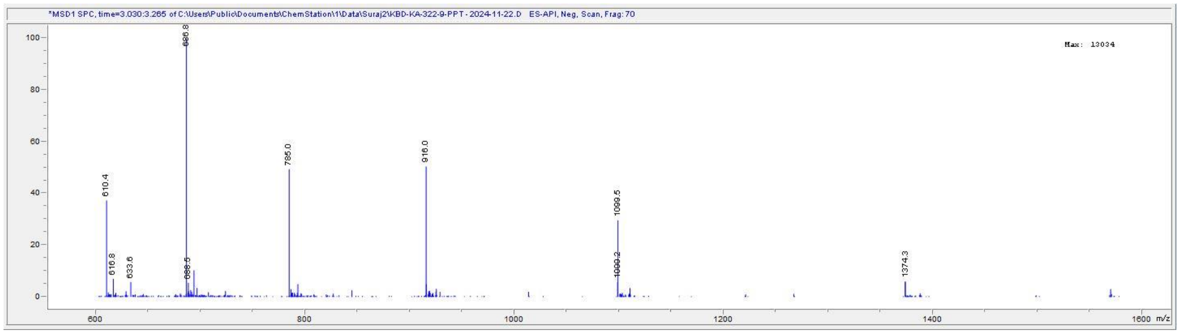

Figure S57. Analytical HPLC trace of **SRu-31** with HPLC method A. (Top) DAD chromatogram at 260 nm. (Middle) TIC chromatogram. (Bottom) Ionization of peak at 3.11 min. containing reaction product.

Decomplexation of SRu-31 to obtain product 31: Under an ambient atmosphere, the DNA-conjugate **SRu-31** stock solution in water (c = 0.10 mM, 20 µL) was irradiated with a 390 nm (40 W) Kessil lamp for 2 hours, while maintaining the temperature at approximately 30 °C through cooling with a fan. To the reaction mixture was added the stock solution of NaCl in water (**SR-06**, 2.0 µL, c = 5.0 M, 10% volume of the total reaction volume), followed by cold ethanol (−20 °C, 66 µL) to precipitate the DNA conjugate **31**. The Eppendorf tube was placed in the freezer (−20 °C) for at least 1 hour, and then it was centrifuged at 4 °C and 11000 × g for at least 30 minutes. The supernatant was removed, the pellet dried under air and dissolved in Milli-Q water to obtain the purified DNA-conjugate **31**. Then, 1 µL of the above solution was diluted to 40 µL with water for LC–MS analysis. The yield of the DNA conjugate was calculated by measuring the integration of the peaks of the diode array detection (DAD) UV absorbance at 260 nm of the LC–MS trace, assuming complete DNA recovery and identical UV absorbance.

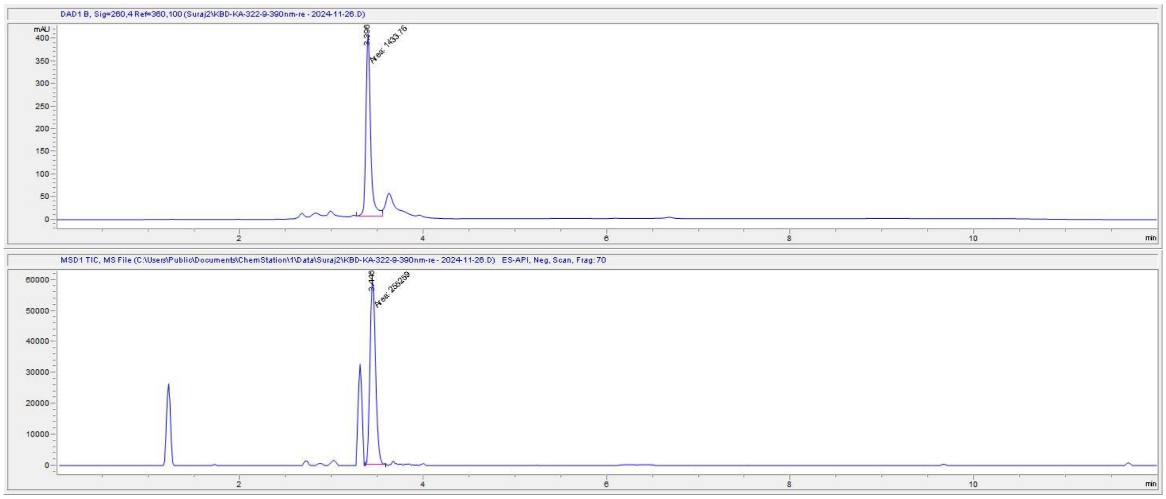

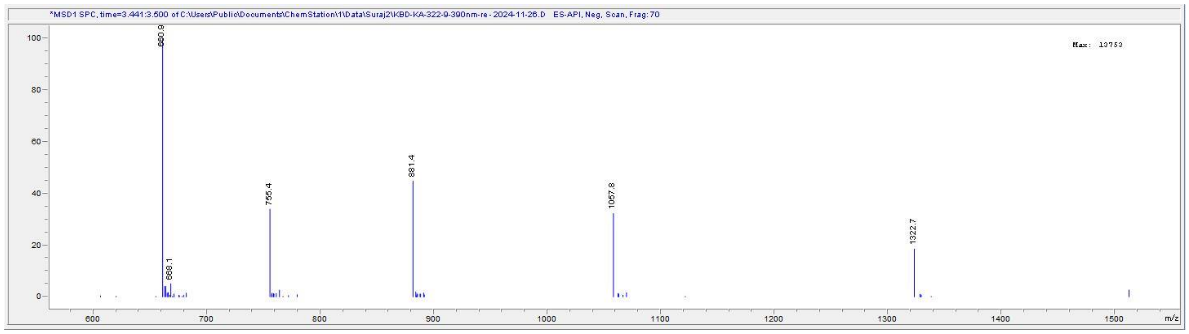

Figure S58. Analytical HPLC trace of **31** with HPLC method A. (Top) DAD chromatogram at 260 nm. (Middle) TIC chromatogram. (Bottom) Ionization of peak at 3.45 min. containing reaction product.

Synthesis of DNA-conjugate 32

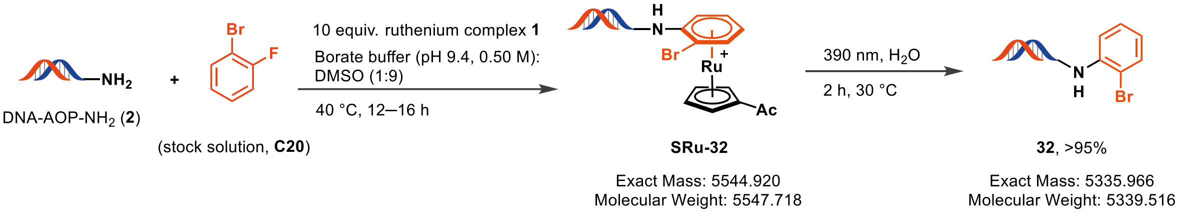

In situ formation of arene-ruthenium stock solution: Under an ambient atmosphere, a 1 mL glass GC vial equipped with a 6 mm Teflon-coated stirring bar was charged with ruthenium complex **1** (1.4 mg, 2.9 µmol, 1.0 equiv.). Next, a stock solution of 1-bromo-2-fluorobenzene in DMC (294 µL, c = 0.10 M, 29 µmol, 10 equiv.) was added. The resulting reaction mixture was heated at 80 °C for 2 hours. After 2 hours, the reaction mixture was cooled to 23 °C. Next, the DMC was removed under a gentle stream of argon and 294 µL of DMSO were added to result an in situ formed stock solution of arene-ruthenium complex **C20** (294 µL, c = 0.01 M, assuming quantitative arene coordination to ruthenium).

Under an ambient atmosphere, the stock solution **SD-01** of DNA-AOP-NH_2 (**2**) in water (1.0 µL, c = 2.0 mM, 2.0 nmol, 1.0 equiv.) was added to a 1.5 mL Eppendorf tube, followed by sodium borate buffer (1.0 µL, pH 9.4, c = 0.50 M). To this mixture, 16 µL of DMSO was added and the solution was vortexed for 5 seconds. Next, the freshly prepared stock solution **C20** (2.0 µL, c = 0.01 M, 0.02 µmol, 10 equiv.) in DMSO was added. The resulting reaction mixture was vortexed for 5 seconds, transferred to a thermocycler at 40 °C, and incubated for 16 hours at 800 rpm to yield the DNA-conjugate **SRu-32**. Next, the reaction mixture was diluted with 10 µL of Milli-Q water. To the reaction mixture was added the stock solution of NaCl in water (**SR-06** , 3.0 µL, c = 5.0 M, 10% volume of the total reaction volume), followed by cold ethanol (−20 °C, 99 µL) to precipitate the *N*-arylated ruthenium DNA conjugate **SRu-32**. The Eppendorf tube was placed in a freezer (−20 °C) for at least 1 hour, and then it was centrifuged at 4 °C and 11000 × g for at least 30 minutes. The supernatant was removed and the pellet was dried under air, then dissolved in 20 µL water to obtain the DNA-conjugate **SRu-32** (20 µL, c = 0.10 mM). Then, 1.0 µL of the above solution was diluted to 40 µL with water for LC–MS analysis.

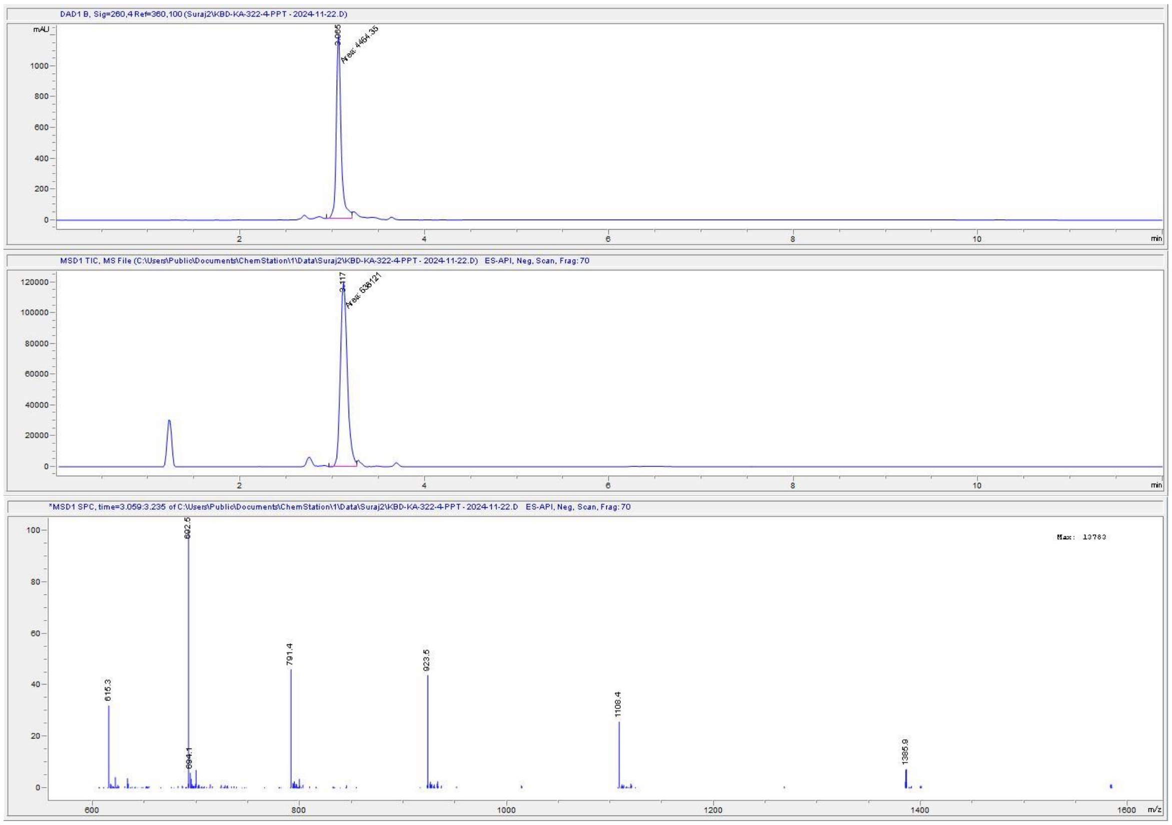

Figure S59. Analytical HPLC trace of **SRu-32** with HPLC method A. (Top) DAD chromatogram at 260 nm. (Middle) TIC chromatogram. (Bottom) Ionization of peak at 3.12 min. containing reaction product.

Decomplexation of SRu-32 to obtain product 32: Under an ambient atmosphere, the DNA-conjugate **SRu-32** stock solution in water (c = 0.10 mM, 20 µL) was irradiated with a 390 nm (40 W) Kessil lamp for 2 hours, while maintaining the temperature at approximately 30 °C through cooling with a fan. To the reaction mixture was added the stock solution of NaCl in water (**SR-06**, 2.0 µL, c = 5.0 M, 10% volume of the total reaction volume), followed by cold ethanol (−20 °C, 66 µL) to precipitate the DNA conjugate **32**. The Eppendorf tube was placed in the freezer (−20 °C) for at least 1 hour, and then it was centrifuged at 4 °C and 11000 × g for at least 30 minutes. The supernatant was removed, the pellet dried under air and dissolved in Milli-Q water to obtain the purified DNA-conjugate **32**. Then, 1 µL of the above solution was diluted to 40 µL with water for LC–MS analysis. The yield of the DNA conjugate was calculated by measuring the integration of the peaks of the diode array detection (DAD) UV absorbance at 260 nm of the LC–MS trace, assuming complete DNA recovery and identical UV absorbance.

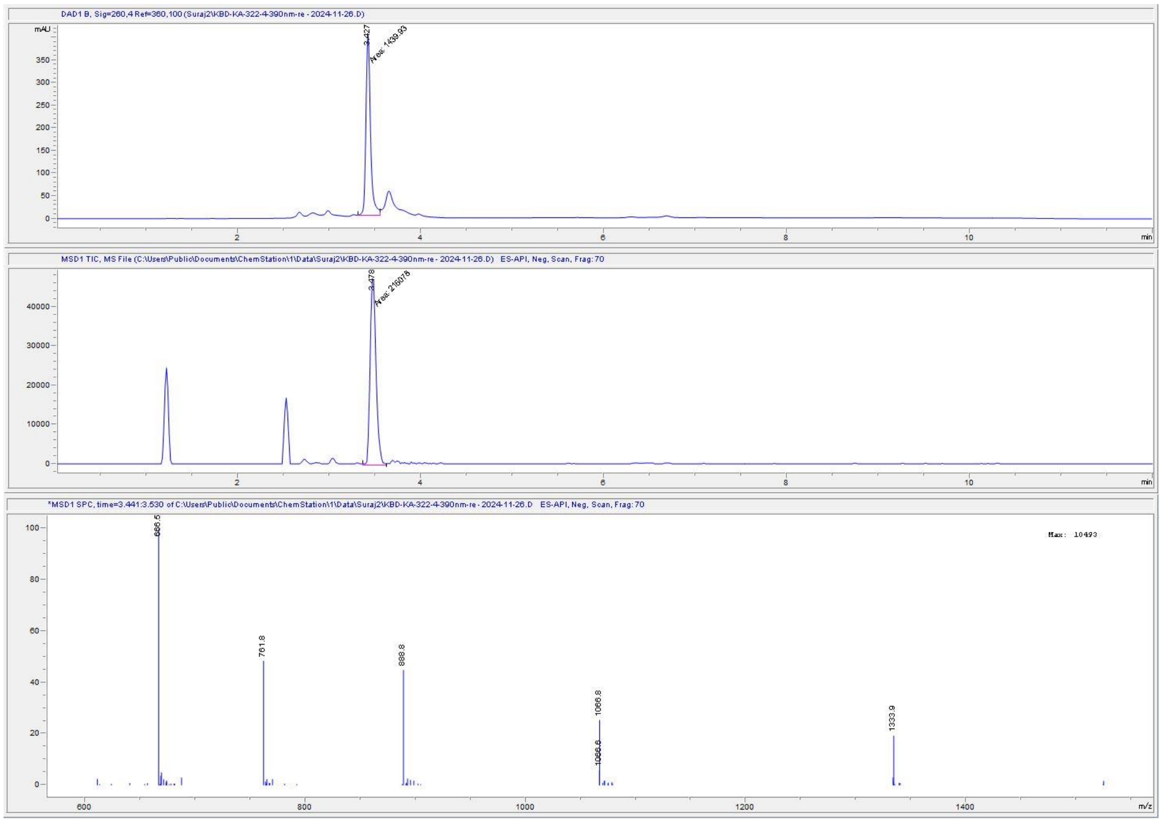

Figure S60. Analytical HPLC trace of **32** with HPLC method A. (Top) DAD chromatogram at 260 nm. (Middle) TIC chromatogram. (Bottom) Ionization of peak at 3.48 min. containing reaction product.

Synthesis of DNA-conjugate 33

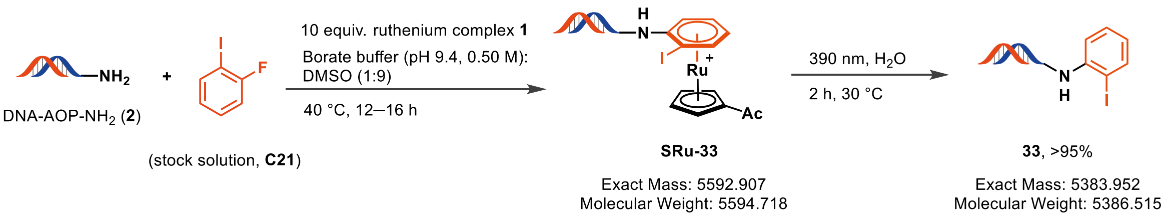

In situ formation of arene-ruthenium stock solution: Under an ambient atmosphere, a 1 mL glass GC vial equipped with a 6 mm Teflon-coated stirring bar was charged with ruthenium complex **1** (1.4 mg, 2.9 µmol, 1.0 equiv.). Next, a stock solution of 1-fluoro-2-iodobenzene in DMC (294 µL, c = 0.10 M, 29 µmol, 10 equiv.) was added. The resulting reaction mixture was heated at 80 °C for 2 hours. After 2 hours, the reaction mixture was cooled to 23 °C. Next, the DMC was removed under a gentle stream of argon and 294 µL of DMSO were added to result an in situ formed stock solution of arene-ruthenium complex **C21** (294 µL, c = 0.01 M, assuming quantitative arene coordination to ruthenium).

Under an ambient atmosphere, the stock solution **SD-01** of DNA-AOP-NH_2 (**2**) in water (1.0 µL, c = 2.0 mM, 2.0 nmol, 1.0 equiv.) was added to a 1.5 mL Eppendorf tube, followed by sodium borate buffer (1.0 µL, pH 9.4, c = 0.50 M). To this mixture, 16 µL of DMSO was added and the solution was vortexed for 5 seconds.

Next, the freshly prepared stock solution **C21** (2.0 µL, c = 0.01 M, 0.02 µmol, 10 equiv.) in DMSO was added. The resulting reaction mixture was vortexed for 5 seconds, transferred to a thermocycler at 40 °C, and incubated for 16 hours at 800 rpm to yield the DNA-conjugate **SRu-33**. Next, the reaction mixture was diluted with 10 µL of Milli-Q water. To the reaction mixture was added the stock solution of NaCl in water (**SR-06** , 3.0 µL, c = 5.0 M, 10% volume of the total reaction volume), followed by cold ethanol (−20 °C, 99 µL) to precipitate the *N*-arylated ruthenium DNA conjugate **SRu-33**. The Eppendorf tube was placed in a freezer (−20 °C) for at least 1 hour, and then it was centrifuged at 4 °C and 11000 × g for at least 30 minutes. The supernatant was removed and the pellet was dried under air, then dissolved in 20 µL water to obtain the DNA-conjugate **SRu-33** (20 µL, c = 0.10 mM). Then, 1.0 µL of the above solution was diluted to 40 µL with water for LC–MS analysis.

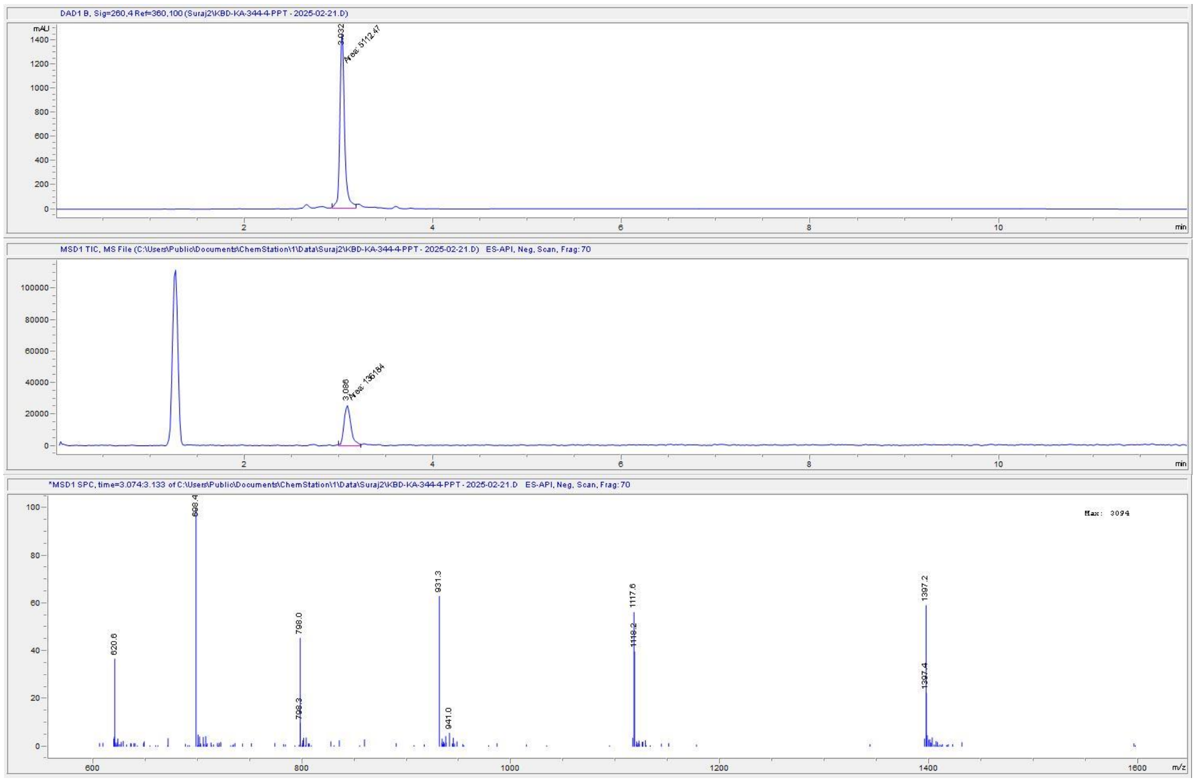

Figure S61. Analytical HPLC trace of **SRu-33** with HPLC method A. (Top) DAD chromatogram at 260 nm. (Middle) TIC chromatogram. (Bottom) Ionization of peak at 3.08 min. containing reaction product.

Decomplexation of SRu-33 to obtain product 33: Under an ambient atmosphere, the DNA-conjugate **SRu-33** stock solution in water (c = 0.10 mM, 20 µL) was irradiated with a 390 nm (40 W) Kessil lamp for 2 hours, while maintaining the temperature at approximately 30 °C through cooling with a fan. To the reaction mixture was added the stock solution of NaCl in water (**SR-06**, 2.0 µL, c = 5.0 M, 10% volume of the total reaction volume), followed by cold ethanol (−20 °C, 66 µL) to precipitate the DNA conjugate **33**. The Eppendorf tube was placed in the freezer (−20 °C) for at least 1 hour, and then it was centrifuged at 4 °C and 11000 × g for at least 30 minutes. The supernatant was removed, the pellet dried under air and dissolved in Milli-Q water to obtain the purified DNA-conjugate **33**. Then, 1 µL of the above solution was diluted to 40 µL with water for

LC–MS analysis. The yield of the DNA conjugate was calculated by measuring the integration of the peaks of the diode array detection (DAD) UV absorbance at 260 nm of the LC–MS trace, assuming complete DNA recovery and identical UV absorbance.

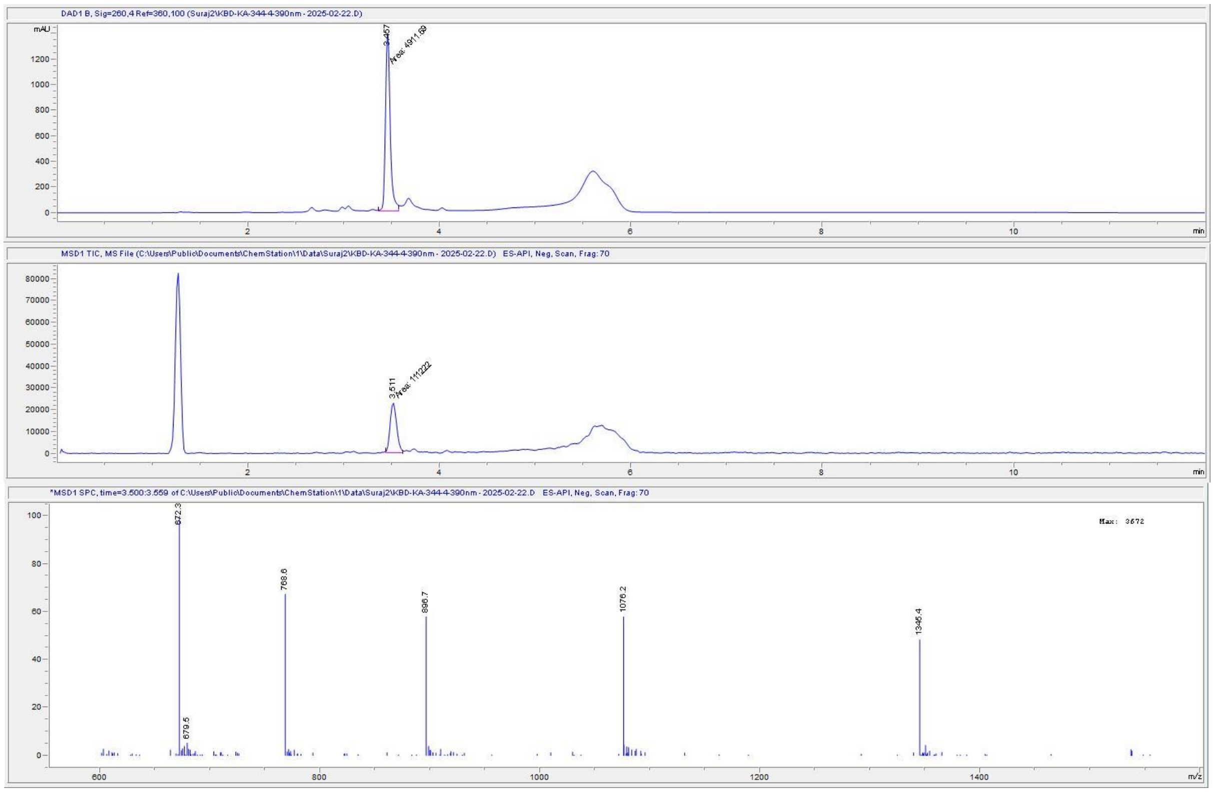

Figure S62. Analytical HPLC trace of **33** with HPLC method A. (Top) DAD chromatogram at 260 nm. (Middle) TIC chromatogram. (Bottom) Ionization of peak at 3.51 min. containing reaction product.

Synthesis of DNA-conjugate 34

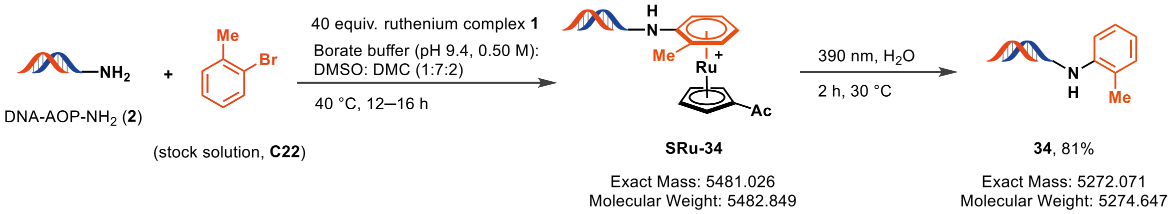

In situ formation of arene-ruthenium stock solution: Under an ambient atmosphere, a 1 mL glass GC vial equipped with a 6 mm Teflon-coated stirring bar was charged with a stock solution of ruthenium complex **1** in DMC (50 µL, c = 40 mM, 2.0 µmol). Next, a stock solution of 1-bromo-2-methylbenzene in DMC (50 µL, c = 0.20 M, 10 µmol, 5.0 equiv.) was added. The resulting reaction mixture was heated at 80 °C for 2 hours. After 2 hours, the reaction mixture was cooled to 23 °C, to result an in situ formed stock solution of arene-ruthenium complex **C22** in DMC (100 µL, c = 0.02 M, assuming quantitative arene coordination to ruthenium).

Under an ambient atmosphere, a stock solution of DNA-AOP-NH_2 (**2**) (1.0 µL, c = 1.0 mM, 1.0 nmol, 1.0

equiv.) in sodium borate buffer (pH 9.4, c = 0.25 M) was added to a 1.5 mL Eppendorf tube. To this mixture, 7 µL of DMSO was added and the solution was vortexed for 5 seconds. The freshly prepared stock solution **C22** (2.0 µL, c = 0.02 M, 0.04 µmol, 40 equiv.) in DMC was then added. The resulting reaction mixture was vortexed for 5 seconds, transferred to a thermocycler at 40 °C, and incubated for 16 hours at 800 rpm to yield the DNA-conjugate **SRu-34**. Next, the reaction mixture was diluted with 10 µL of Milli-Q water. To the reaction mixture was added the stock solution of NaCl in water (**SR-06** , 2.0 µL, c = 5.0 M, 10% volume of the total reaction volume), followed by cold ethanol (−20 °C, 66 µL) to precipitate the *N*-arylated ruthenium DNA conjugate **SRu-34**. The Eppendorf tube was placed in a freezer (−20 °C) for at least 1 hour, and then it was centrifuged at 4 °C and 11000 × g for at least 30 minutes. The supernatant was removed and the pellet was dried under air, then dissolved in 10 µL water to obtain the DNA-conjugate **SRu-34** (10 µL, c = 0.10 mM). Then, 1.0 µL of the above solution was diluted to 40 µL with water for LC–MS analysis.

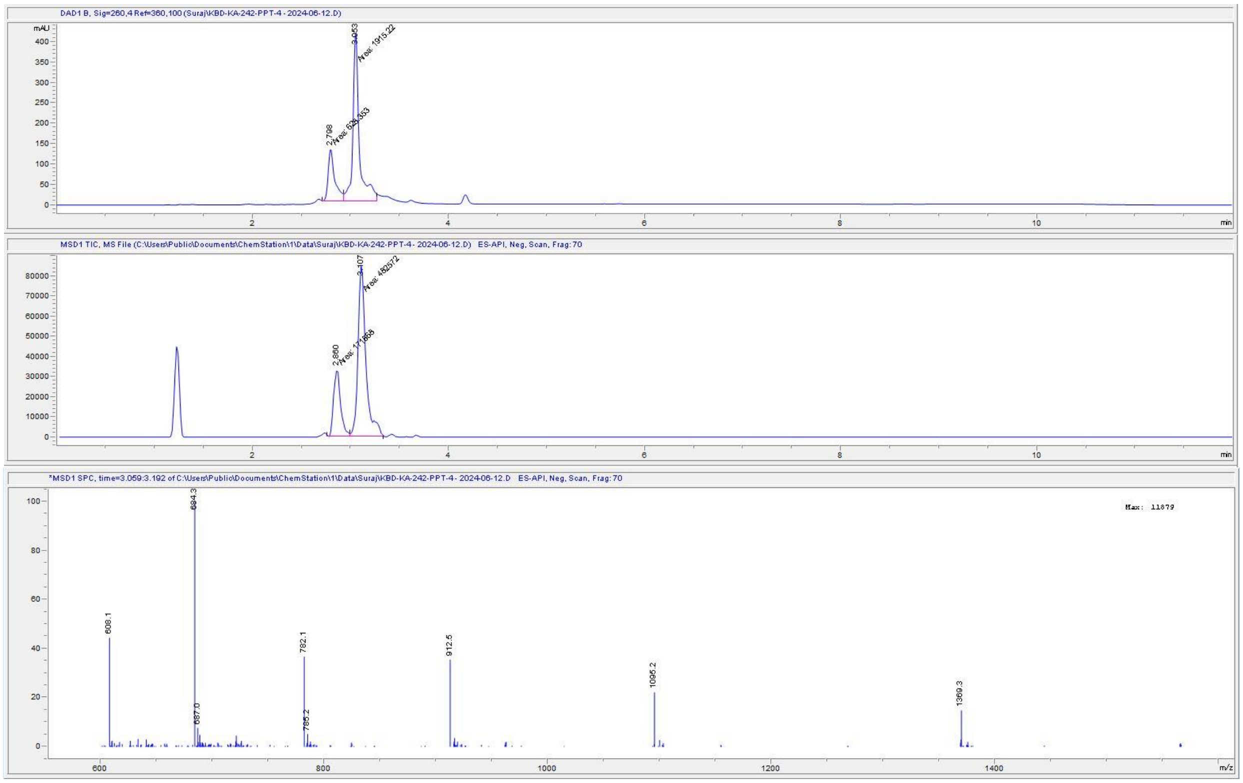

Figure S63. Analytical HPLC trace of **SRu-34** with HPLC method A. (Top) DAD chromatogram at 260 nm. (Middle) TIC chromatogram. (Bottom) Ionization of peak at 3.11 min. containing reaction product.

Decomplexation of SRu-34 to obtain product 34: Under an ambient atmosphere, the DNA-conjugate **SRu-34** stock solution in water (c = 0.10 mM, 10 µL) was irradiated with a 390 nm (40 W) Kessil lamp for 2 hours, while maintaining the temperature at approximately 30 °C through cooling with a fan. To the reaction mixture was added the stock solution of NaCl in water (**SR-06**, 1.0 µL, c = 5.0 M, 10% volume of the total reaction volume), followed by cold ethanol (−20 °C, 33 µL) to precipitate the DNA conjugate **34**. The Eppendorf tube was placed in the freezer (−20 °C) for at least 1 hour, and then it was centrifuged at 4 °C and 11000 × g for at least 30 minutes. The supernatant was removed, the pellet dried under air and dissolved in Milli-Q water to

obtain the purified DNA-conjugate **34**. Then, 1 µL of the above solution was diluted to 40 µL with water for LC–MS analysis. The yield of the DNA conjugate was calculated by measuring the integration of the peaks of the TIC chromatogram.

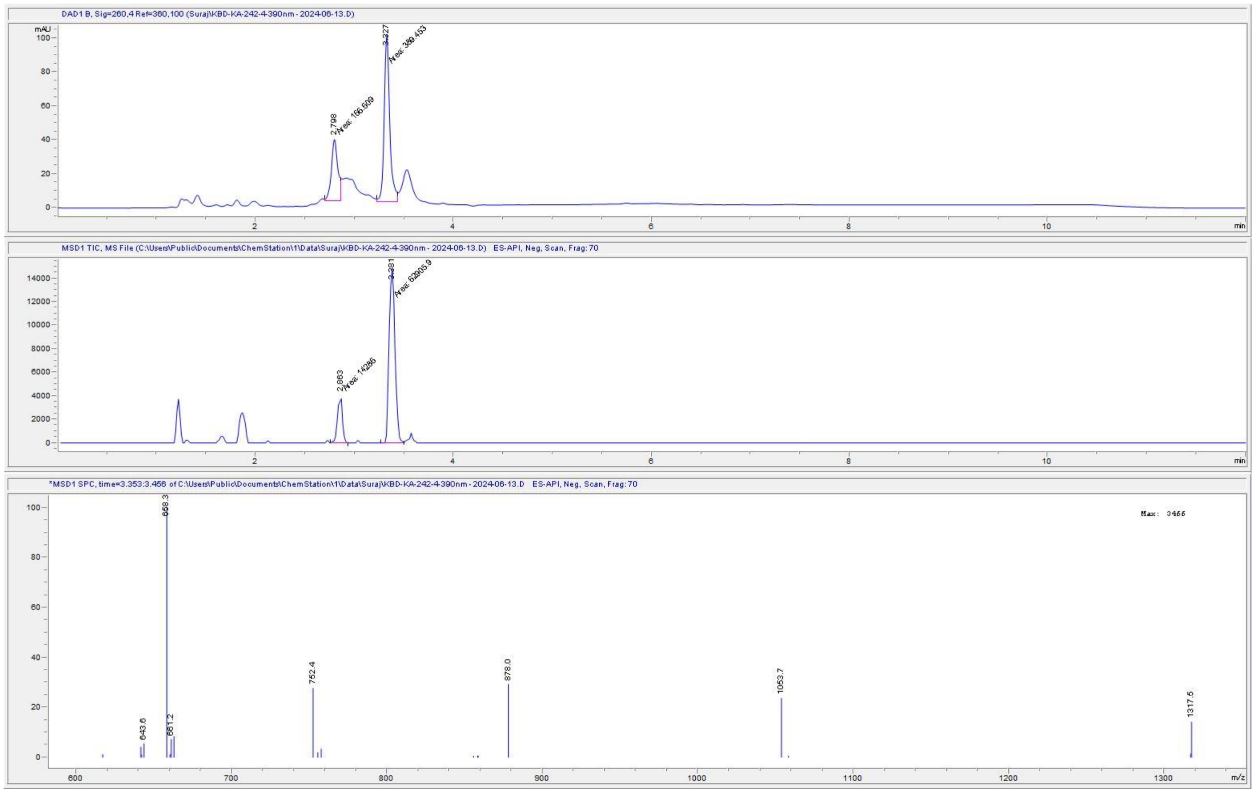

Figure S64. Analytical HPLC trace of **34** with HPLC method A. (Top) DAD chromatogram at 260 nm. (Middle) TIC chromatogram. (Bottom) Ionization of peak at 3.38 min. containing reaction product.

Synthesis of DNA-conjugate 35

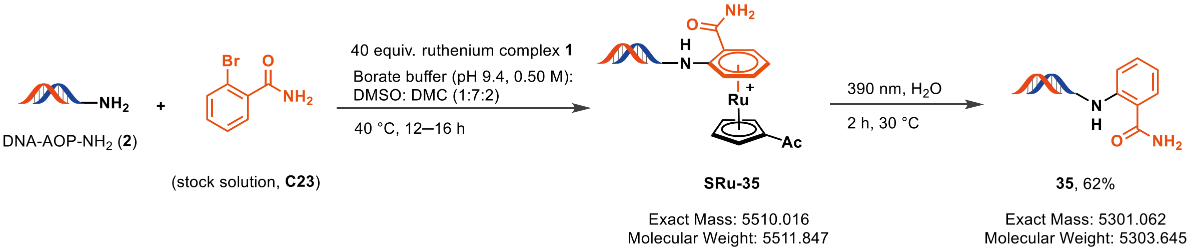

In situ formation of arene-ruthenium stock solution: Under an ambient atmosphere, a 1 mL glass GC vial equipped with a 6 mm Teflon-coated stirring bar was charged with a stock solution of ruthenium complex **1** in DMC (50 µL, c = 40 mM, 2.0 µmol). Next, a stock solution of 2-bromobenzamine in DMC (50 µL, c = 0.20 M, 10 µmol, 5.0 equiv.) was added. The resulting reaction mixture was heated at 80 °C for 2 hours. After 2 hours, the reaction mixture was cooled to 23 °C, to result an in situ formed stock solution of arene-ruthenium complex **C23** in DMC (100 µL, c = 0.02 M, assuming quantitative arene coordination to ruthenium).

Under an ambient atmosphere, a stock solution of DNA-AOP-NH_2 (**2**) (1.0 µL, c = 1.0 mM, 1.0 nmol, 1.0 equiv.) in sodium borate buffer (pH 9.4, c = 0.25 M) was added to a 1.5 mL Eppendorf tube. To this mixture, 7 µL of DMSO was added and the solution was vortexed for 5 seconds. The freshly prepared stock solution **C23** (2.0 µL, c = 0.02 M, 0.04 µmol, 40 equiv.) in DMC was then added. The resulting reaction mixture was vortexed for 5 seconds, transferred to a thermocycler at 40 °C, and incubated for 16 hours at 800 rpm to yield the DNA-conjugate **SRu-35**. Next, the reaction mixture was diluted with 10 µL of Milli-Q water. To the reaction mixture was added the stock solution of NaCl in water (**SR-06** , 2.0 µL, c = 5.0 M, 10% volume of the total reaction volume), followed by cold ethanol (−20 °C, 66 µL) to precipitate the *N*-arylated ruthenium DNA conjugate **SRu-35**. The Eppendorf tube was placed in a freezer (−20 °C) for at least 1 hour, and then it was centrifuged at 4 °C and 11000 × g for at least 30 minutes. The supernatant was removed and the pellet was dried under air, then dissolved in 10 µL water to obtain the DNA-conjugate **SRu-35** (10 µL, c = 0.10 mM). Then, 1.0 µL of the above solution was diluted to 40 µL with water for LC–MS analysis.

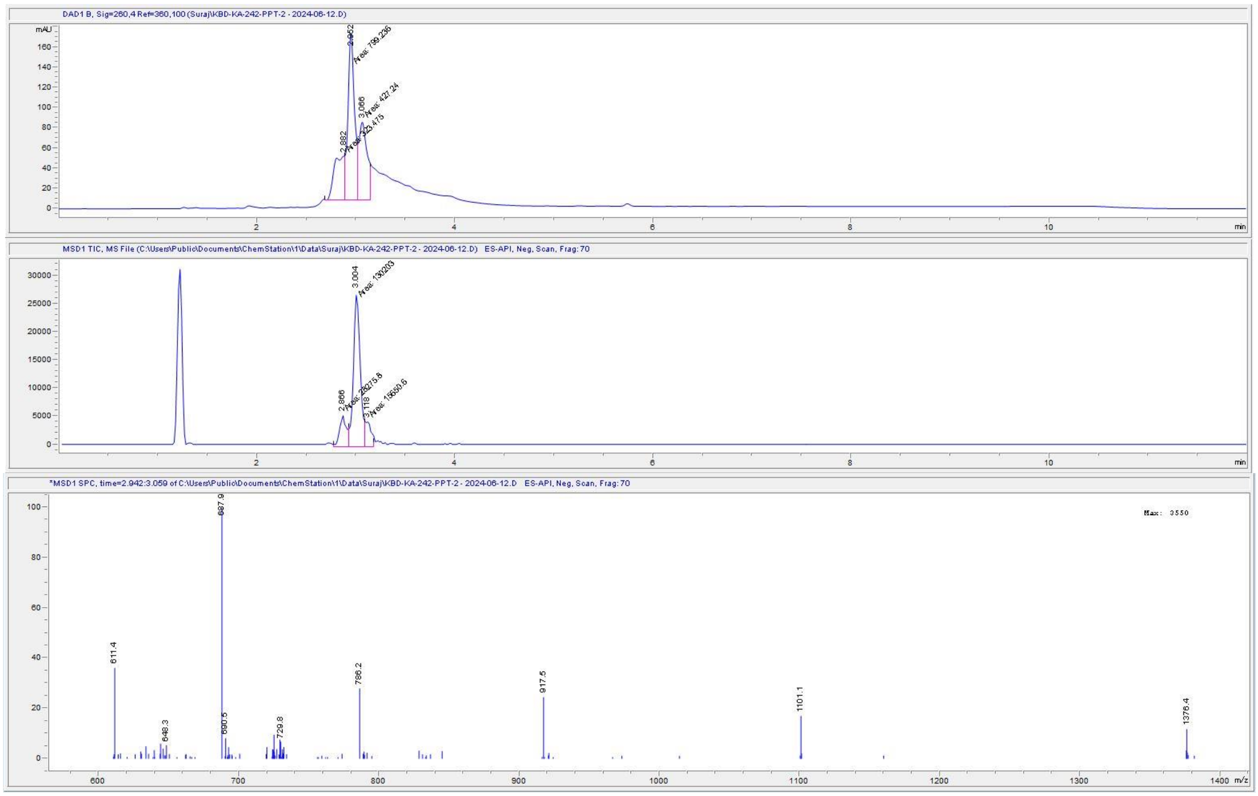

Figure S65. Analytical HPLC trace of **SRu-35** with HPLC method A. (Top) DAD chromatogram at 260 nm. (Middle) TIC chromatogram. (Bottom) Ionization of peak at 3.00 min. containing reaction product.

Decomplexation of SRu-35 to obtain product 35: Under an ambient atmosphere, the DNA-conjugate **SRu-35** stock solution in water (c = 0.10 mM, 10 µL) was irradiated with a 390 nm (40 W) Kessil lamp for 2 hours, while maintaining the temperature at approximately 30 °C through cooling with a fan. To the reaction mixture was added the stock solution of NaCl in water (**SR-06**, 1.0 µL, c = 5.0 M, 10% volume of the total reaction volume), followed by cold ethanol (−20 °C, 33 µL) to precipitate the DNA conjugate **35**. The Eppendorf tube

was placed in the freezer (−20 °C) for at least 1 hour, and then it was centrifuged at 4 °C and 11000 × g for at least 30 minutes. The supernatant was removed, the pellet dried under air and dissolved in Milli-Q water to obtain the purified DNA-conjugate **35**. Then, 1 µL of the above solution was diluted to 40 µL with water for LC–MS analysis. The yield of the DNA conjugate was calculated by measuring the integration of the peaks of the diode array detection (DAD) UV absorbance at 260 nm of the LC–MS trace, assuming complete DNA recovery and identical UV absorbance.

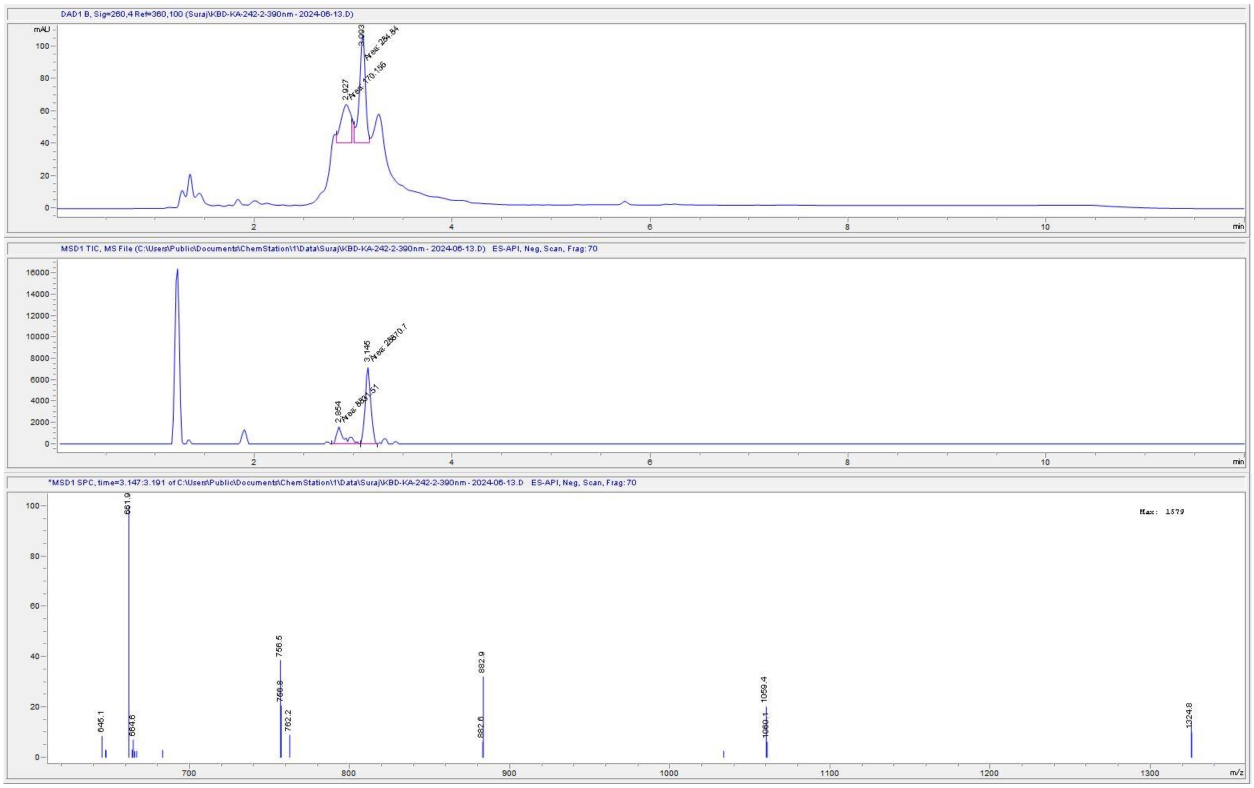

Figure S66. Analytical HPLC trace of **35** with HPLC method A. (Top) DAD chromatogram at 260 nm. (Middle) TIC chromatogram. (Bottom) Ionization of peak at 3.14 min. containing reaction product.

Synthesis of DNA-conjugate 36

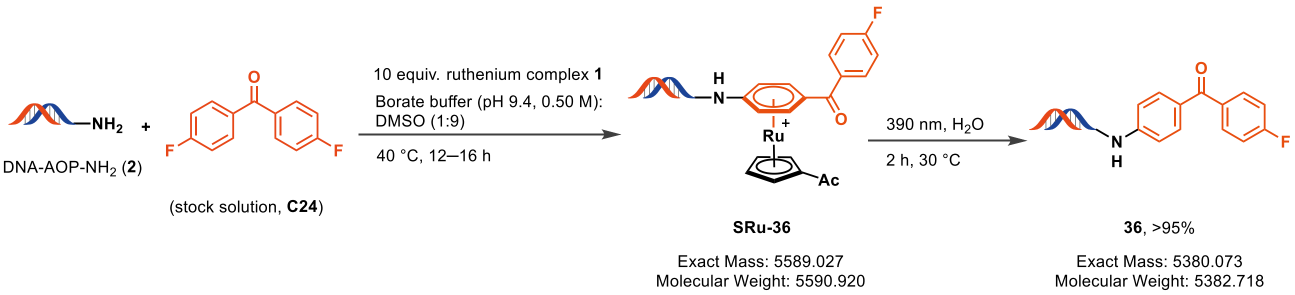

In situ formation of arene-ruthenium stock solution: Under an ambient atmosphere, a 1 mL glass GC vial equipped with a 6 mm Teflon-coated stirring bar was charged with ruthenium complex **1** (1.4 mg, 2.9 µmol, 1.0 equiv.). Next, a stock solution of bis(4-fluorophenyl)methanone in DMC (294 µL, c = 0.10 M, 29 µmol, 10

equiv.) was added. The resulting reaction mixture was heated at 80 °C for 2 hours. After 2 hours, the reaction mixture was cooled to 23 °C. Next, the DMC was removed under a gentle stream of argon and 294 µL of DMSO were added to result an in situ formed stock solution of arene-ruthenium complex **C24** (294 µL, c = 0.01 M, assuming quantitative arene coordination to ruthenium).

Under an ambient atmosphere, the stock solution **SD-01** of DNA-AOP-NH_2 (**2**) in water (1.0 µL, c = 2.0 mM, 2.0 nmol, 1.0 equiv.) was added to a 1.5 mL Eppendorf tube, followed by sodium borate buffer (1.0 µL, pH 9.4, c = 0.50 M). To this mixture, 16 µL of DMSO was added and the solution was vortexed for 5 seconds. Next, the freshly prepared stock solution **C24** (2.0 µL, c = 0.01 M, 0.02 µmol, 10 equiv.) in DMSO was added. The resulting reaction mixture was vortexed for 5 seconds, transferred to a thermocycler at 40 °C, and incubated for 16 hours at 800 rpm to yield the DNA-conjugate **SRu-36**. Next, the reaction mixture was diluted with 10 µL of Milli-Q water. To the reaction mixture was added the stock solution of NaCl in water (**SR-06** , 3.0 µL, c = 5.0 M, 10% volume of the total reaction volume), followed by cold ethanol (−20 °C, 99 µL) to precipitate the *N*-arylated ruthenium DNA conjugate **SRu-36**. The Eppendorf tube was placed in a freezer (−20 °C) for at least 1 hour, and then it was centrifuged at 4 °C and 11000 × g for at least 30 minutes. The supernatant was removed and the pellet was dried under air, then dissolved in 20 µL water to obtain the DNA-conjugate **SRu-36** (20 µL, c = 0.10 mM). Then, 1.0 µL of the above solution was diluted to 40 µL with water for LC–MS analysis.

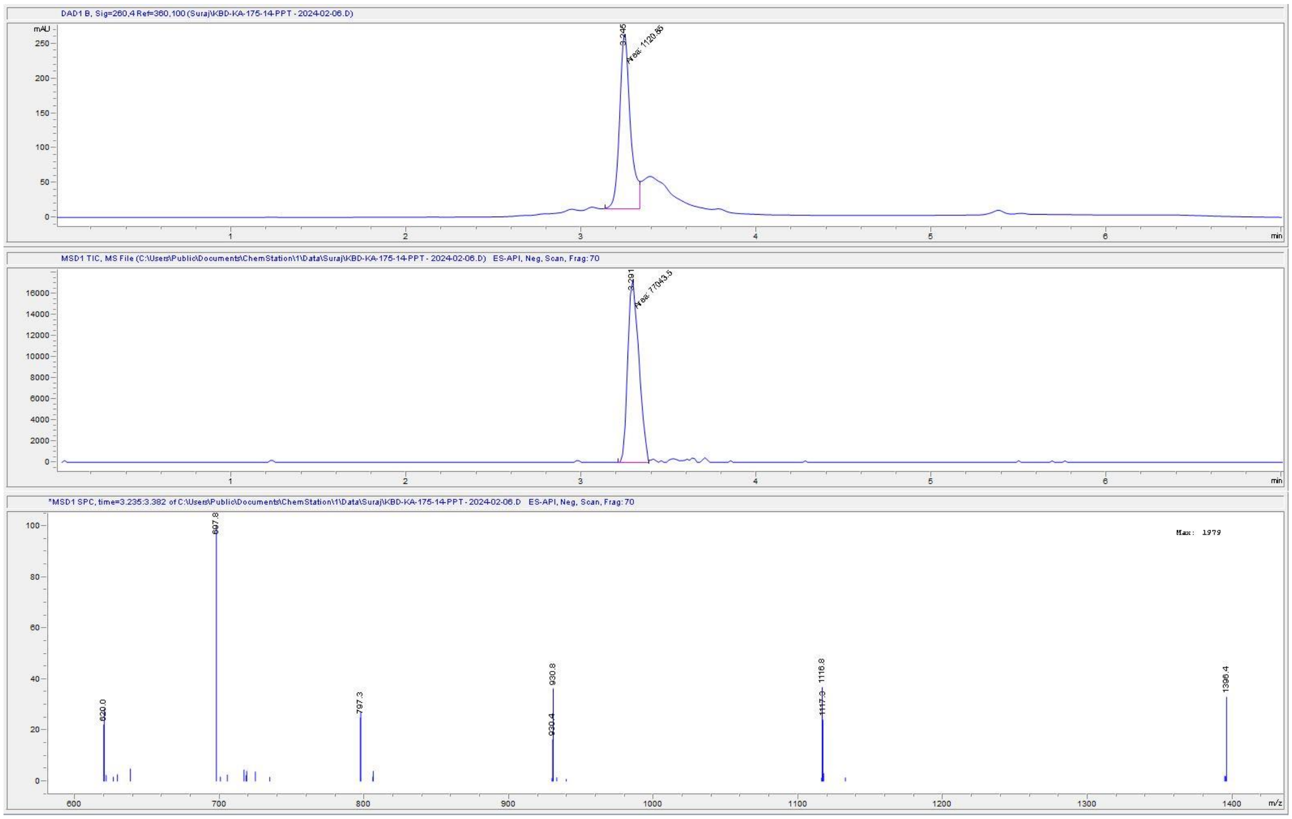

Figure S67. Analytical HPLC trace of **SRu-36** with HPLC method B. (Top) DAD chromatogram at 260 nm. (Middle) TIC chromatogram. (Bottom) Ionization of peak at 3.29 min. containing reaction product.

Decomplexation of SRu-36 to obtain product 36: Under an ambient atmosphere, the DNA-conjugate **SRu-36** stock solution in water (c = 0.10 mM, 20 µL) was irradiated with a 390 nm (40 W) Kessil lamp for 2 hours, while maintaining the temperature at approximately 30 °C through cooling with a fan. To the reaction mixture was added the stock solution of NaCl in water (**SR-06**, 2.0 µL, c = 5.0 M, 10% volume of the total reaction volume), followed by cold ethanol (−20 °C, 66 µL) to precipitate the DNA conjugate **36**. The Eppendorf tube was placed in the freezer (−20 °C) for at least 1 hour, and then it was centrifuged at 4 °C and 11000 × g for at least 30 minutes. The supernatant was removed, the pellet dried under air and dissolved in Milli-Q water to obtain the purified DNA-conjugate **36**. Then, 1 µL of the above solution was diluted to 40 µL with water for LC–MS analysis. The yield of the DNA conjugate was calculated by measuring the integration of the peaks of the diode array detection (DAD) UV absorbance at 260 nm of the LC–MS trace, assuming complete DNA recovery and identical UV absorbance.

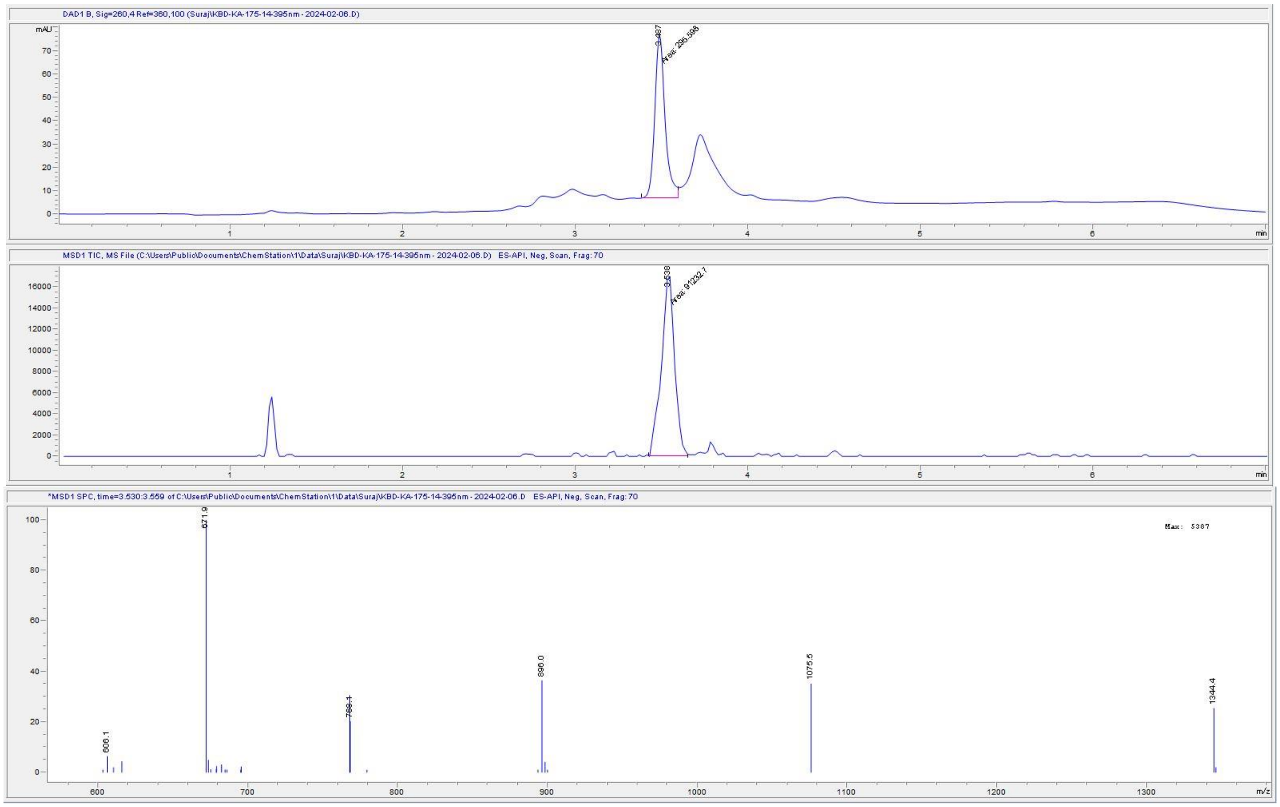

Figure S68. Analytical HPLC trace of **36** with HPLC method B. (Top) DAD chromatogram at 260 nm. (Middle) TIC chromatogram. (Bottom) Ionization of peak at 3.54 min. containing reaction product.

Synthesis of DNA-conjugate 37

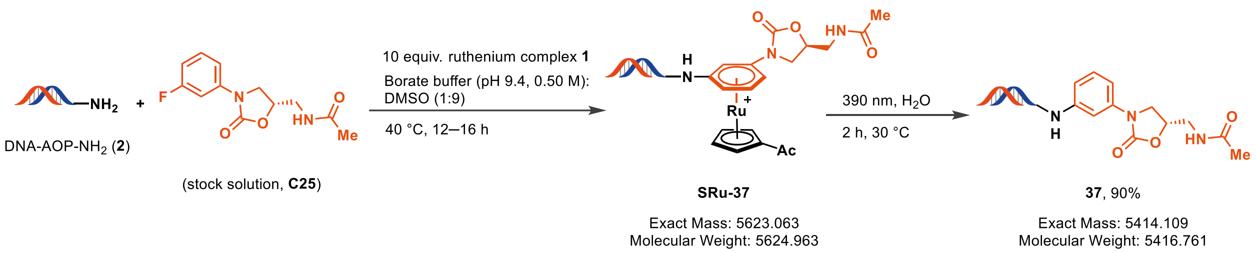

In situ formation of arene-ruthenium stock solution: Under an ambient atmosphere, a 1 mL glass GC vial equipped with a 6 mm Teflon-coated stirring bar was charged with ruthenium complex **1** (1.4 mg, 2.9 µmol, 1.0 equiv.). Next, a stock solution of (*S*)-N-((3-(3-fluorophenyl)-2-oxooxazolidin-5-yl)methyl)acetamide in DMC (294 µL, c = 0.10 M, 29 µmol, 10 equiv.) was added. The resulting reaction mixture was heated at 80 °C for 2 hours. After 2 hours, the reaction mixture was cooled to 23 °C. Next, the DMC was removed under a gentle stream of argon and 294 µL of DMSO were added to result an in situ formed stock solution of arene-ruthenium complex **C25** (294 µL, c = 0.01 M, assuming quantitative arene coordination to ruthenium).

Under an ambient atmosphere, the stock solution **SD-01** of DNA-AOP-NH_2 (**2**) in water (1.0 µL, c = 2.0 mM, 2.0 nmol, 1.0 equiv.) was added to a 1.5 mL Eppendorf tube, followed by sodium borate buffer (1.0 µL, pH 9.4, c = 0.50 M). To this mixture, 16 µL of DMSO was added and the solution was vortexed for 5 seconds. Next, the freshly prepared stock solution **C25** (2.0 µL, c = 0.01 M, 0.02 µmol, 10 equiv.) in DMSO was added. The resulting reaction mixture was vortexed for 5 seconds, transferred to a thermocycler at 40 °C, and incubated for 16 hours at 800 rpm to yield the DNA-conjugate **SRu-37**. Next, the reaction mixture was diluted with 10 µL of Milli-Q water. To the reaction mixture was added the stock solution of NaCl in water (**SR-06** , 3.0 µL, c = 5.0 M, 10% volume of the total reaction volume), followed by cold ethanol (−20 °C, 99 µL) to precipitate the *N*-arylated ruthenium DNA conjugate **SRu-37**. The Eppendorf tube was placed in a freezer (−20 °C) for at least 1 hour, and then it was centrifuged at 4 °C and 11000 × g for at least 30 minutes. The supernatant was removed and the pellet was dried under air, then dissolved in 20 µL water to obtain the DNA-conjugate **SRu-37** (20 µL, c = 0.10 mM). Then, 1.0 µL of the above solution was diluted to 40 µL with water for LC–MS analysis.

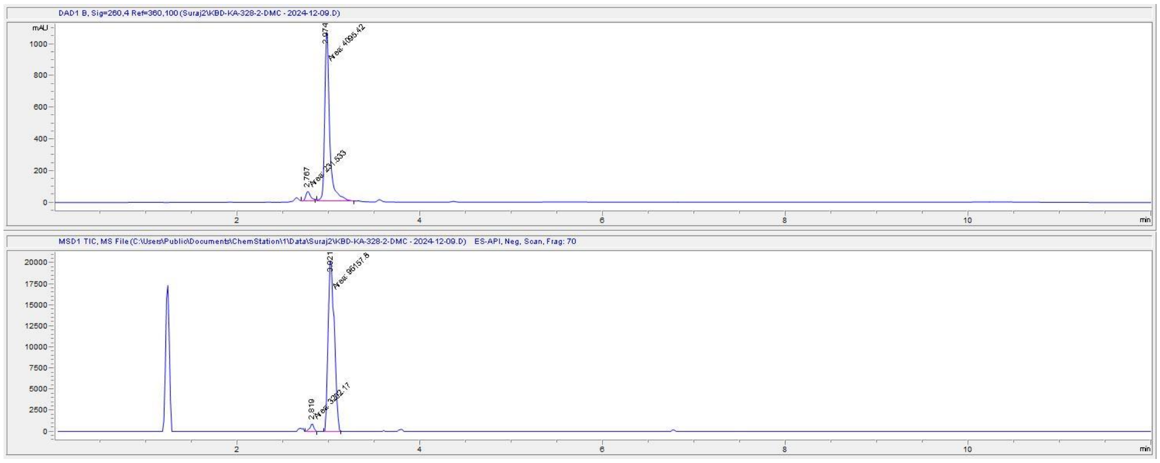

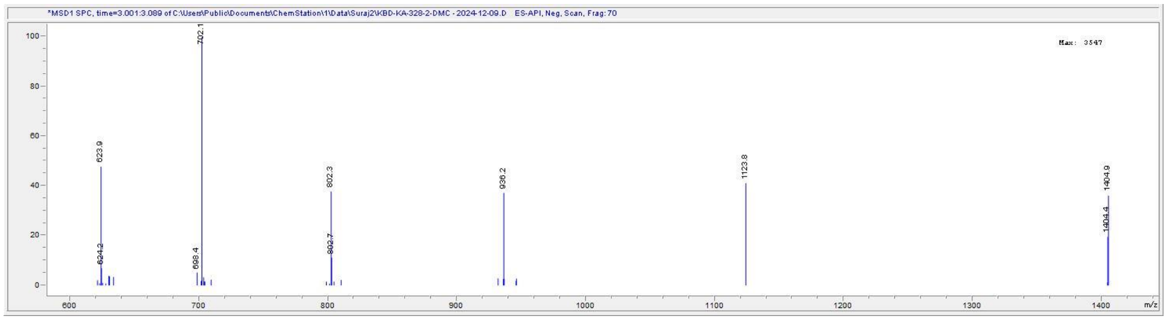

Figure S69. Analytical HPLC trace of **SRu-37** with HPLC method B. (Top) DAD chromatogram at 260 nm. (Middle) TIC chromatogram. (Bottom) Ionization of peak at 3.0 min. containing reaction product.

Decomplexation of SRu-37 to obtain product 37: Under an ambient atmosphere, the DNA-conjugate **SRu-37** stock solution in water (c = 0.10 mM, 20 µL) was irradiated with a 390 nm (40 W) Kessil lamp for 2 hours, while maintaining the temperature at approximately 30 °C through cooling with a fan. To the reaction mixture was added the stock solution of NaCl in water (**SR-06**, 2.0 µL, c = 5.0 M, 10% volume of the total reaction volume), followed by cold ethanol (−20 °C, 66 µL) to precipitate the DNA conjugate **37**. The Eppendorf tube was placed in the freezer (−20 °C) for at least 1 hour, and then it was centrifuged at 4 °C and 11000 × g for at least 30 minutes. The supernatant was removed, the pellet dried under air and dissolved in Milli-Q water to obtain the purified DNA-conjugate **37**. Then, 1 µL of the above solution was diluted to 40 µL with water for LC–MS analysis. The yield of the DNA conjugate was calculated by measuring the integration of the peaks of the diode array detection (DAD) UV absorbance at 260 nm of the LC–MS trace, assuming complete DNA recovery and identical UV absorbance.

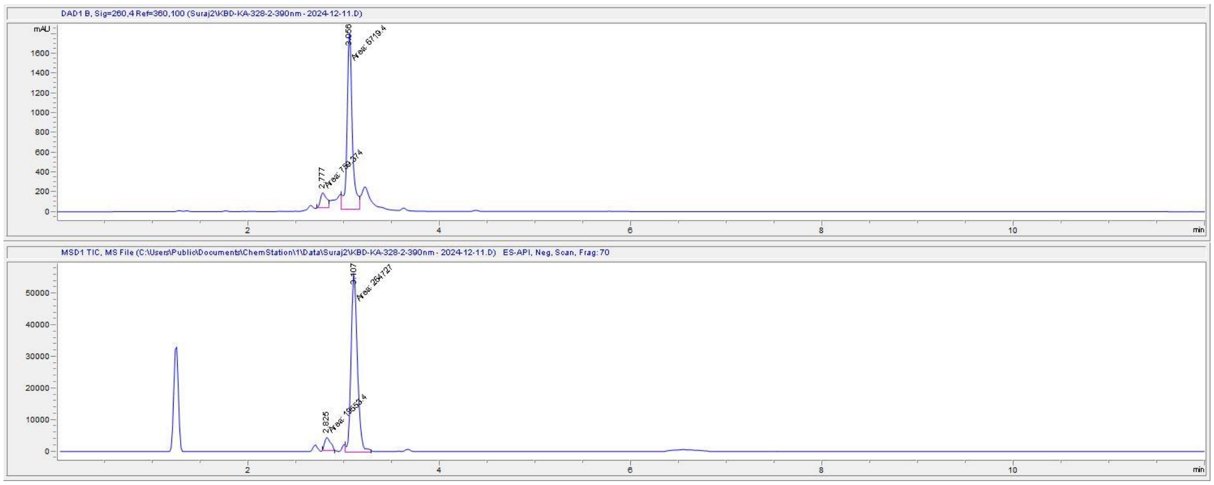

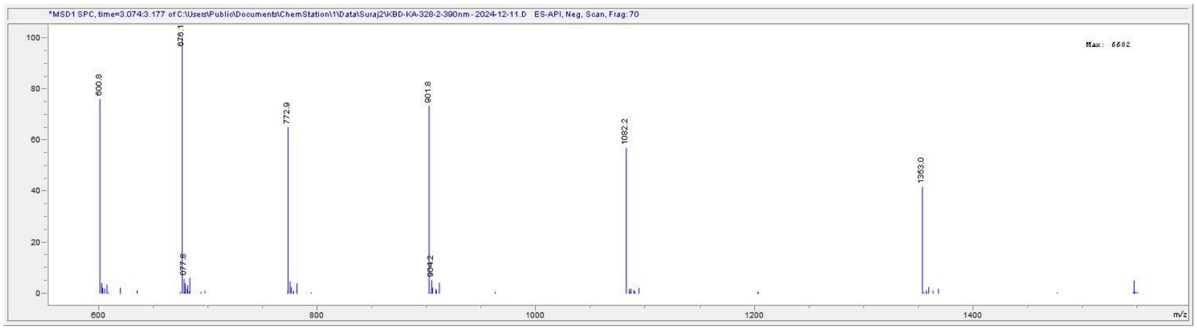

Figure S70. Analytical HPLC trace of **37** with HPLC method A. (Top) DAD chromatogram at 260 nm. (Middle) TIC chromatogram. (Bottom) Ionization of peak at 3.11 min. containing reaction product.

Synthesis of DNA-conjugate 38

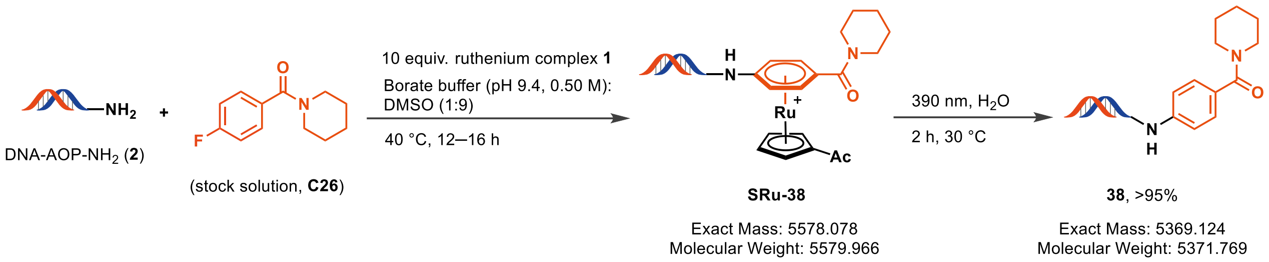

In situ formation of arene-ruthenium stock solution: Under an ambient atmosphere, a 1 mL glass GC vial equipped with a 6 mm Teflon-coated stirring bar was charged with ruthenium complex **1** (1.4 mg, 2.9 µmol, 1.0 equiv.). Next, a stock solution of (4-fluorophenyl)(piperidin-1-yl)methanone in DMC (294 µL, c = 0.10 M, 29 µmol, 10 equiv.) was added. The resulting reaction mixture was heated at 80 °C for 2 hours. After 2 hours, the reaction mixture was cooled to 23 °C. Next, the DMC was removed under a gentle stream of argon and 294 µL of DMSO were added to result an in situ formed stock solution of arene-ruthenium complex **C26** (294 µL, c = 0.01 M, assuming quantitative arene coordination to ruthenium).

Under an ambient atmosphere, the stock solution **SD-01** of DNA-AOP-NH_2 (**2**) in water (1.0 µL, c = 2.0 mM, 2.0 nmol, 1.0 equiv.) was added to a 1.5 mL Eppendorf tube, followed by sodium borate buffer (1.0 µL, pH 9.4, c = 0.50 M). To this mixture, 16 µL of DMSO was added and the solution was vortexed for 5 seconds. Next, the freshly prepared stock solution **C26** (2.0 µL, c = 0.01 M, 0.02 µmol, 10 equiv.) in DMSO was added. The resulting reaction mixture was vortexed for 5 seconds, transferred to a thermocycler at 40 °C, and incubated for 16 hours at 800 rpm to yield the DNA-conjugate **SRu-38**. Next, the reaction mixture was diluted with 10 µL of Milli-Q water. To the reaction mixture was added the stock solution of NaCl in water (**SR-06** , 3.0 µL, c = 5.0 M, 10% volume of the total reaction volume), followed by cold ethanol (−20 °C, 99 µL) to precipitate the *N*-arylated ruthenium DNA conjugate **SRu-38**. The Eppendorf tube was placed in a freezer (−20 °C) for at least 1 hour, and then it was centrifuged at 4 °C and 11000 × g for at least 30 minutes. The supernatant was removed and the pellet was dried under air, then dissolved in 20 µL water to obtain the DNA-conjugate **SRu-38** (20 µL, c = 0.10 mM). Then, 1.0 µL of the above solution was diluted to 40 µL with water for LC–MS analysis.

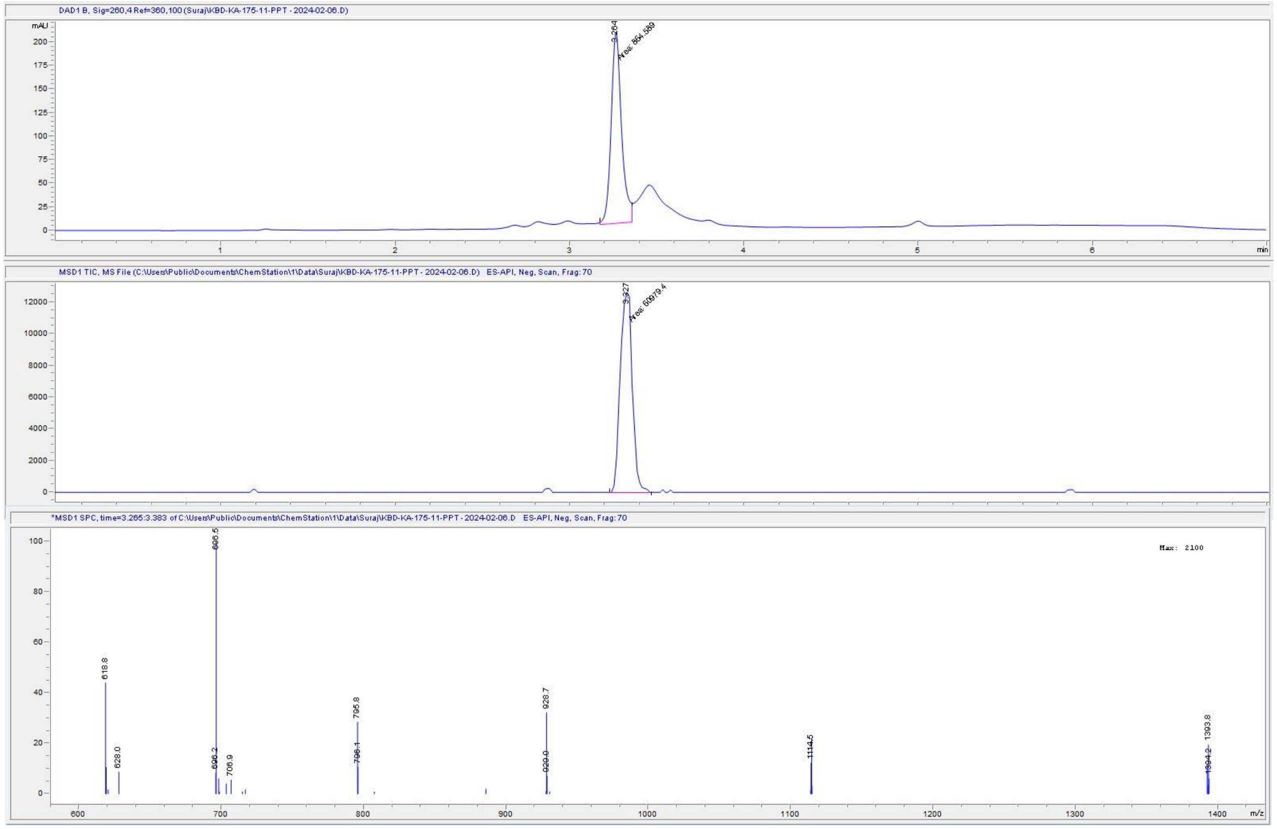

Figure S71. Analytical HPLC trace of **SRu-38** with HPLC method B. (Top) DAD chromatogram at 260 nm. (Middle) TIC chromatogram. (Bottom) Ionization of peak at 3.33 min. containing reaction product.

Decomplexation of SRu-38 to obtain product 38: Under an ambient atmosphere, the DNA-conjugate **SRu-38** stock solution in water (c = 0.10 mM, 20 µL) was irradiated with a 390 nm (40 W) Kessil lamp for 2 hours, while maintaining the temperature at approximately 30 °C through cooling with a fan. To the reaction mixture was added the stock solution of NaCl in water (**SR-06**, 2.0 µL, c = 5.0 M, 10% volume of the total reaction volume), followed by cold ethanol (−20 °C, 66 µL) to precipitate the DNA conjugate **38**. The Eppendorf tube was placed in the freezer (−20 °C) for at least 1 hour, and then it was centrifuged at 4 °C and 11000 × g for at least 30 minutes. The supernatant was removed, the pellet dried under air and dissolved in Milli-Q water to obtain the purified DNA-conjugate **38**. Then, 1 µL of the above solution was diluted to 40 µL with water for LC–MS analysis. The yield of the DNA conjugate was calculated by measuring the integration of the peaks of the diode array detection (DAD) UV absorbance at 260 nm of the LC–MS trace, assuming complete DNA recovery and identical UV absorbance.

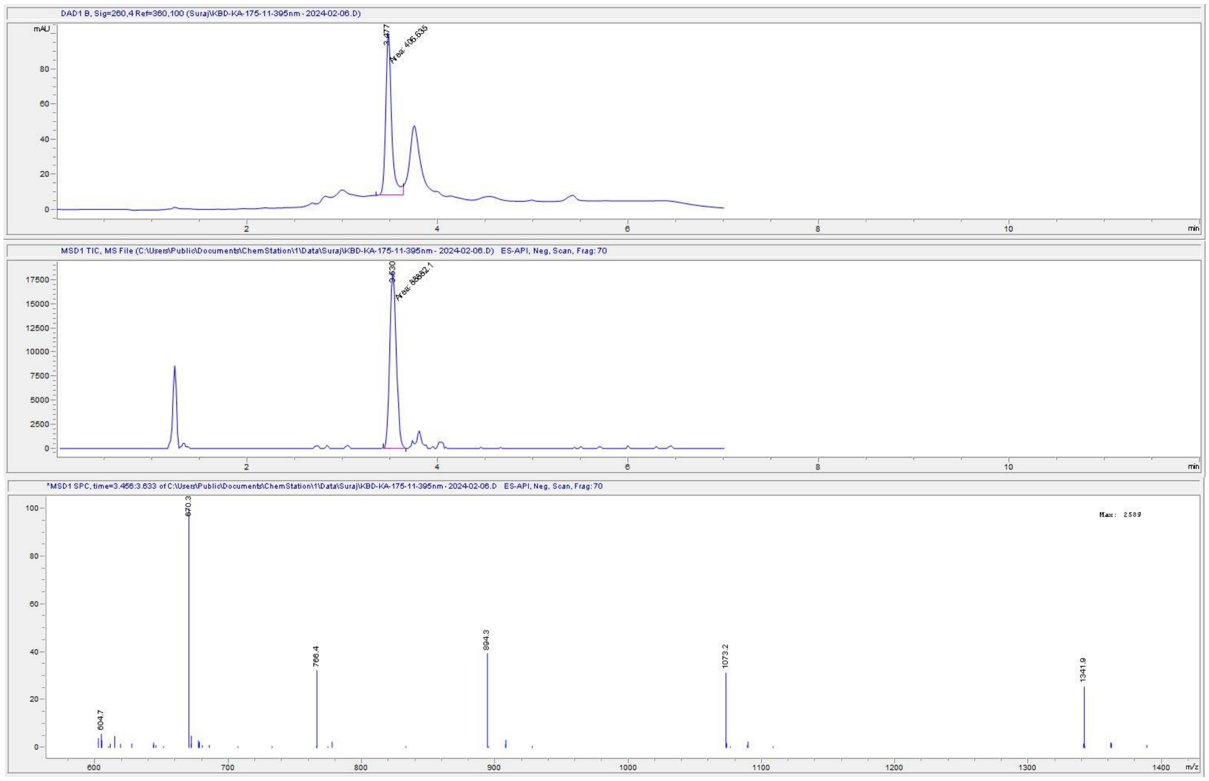

Figure S72. Analytical HPLC trace of **38** with HPLC method B. (Top) DAD chromatogram at 260 nm. (Middle) TIC chromatogram. (Bottom) Ionization of peak at 3.5 min. containing reaction product.

Synthesis of DNA-conjugate 39

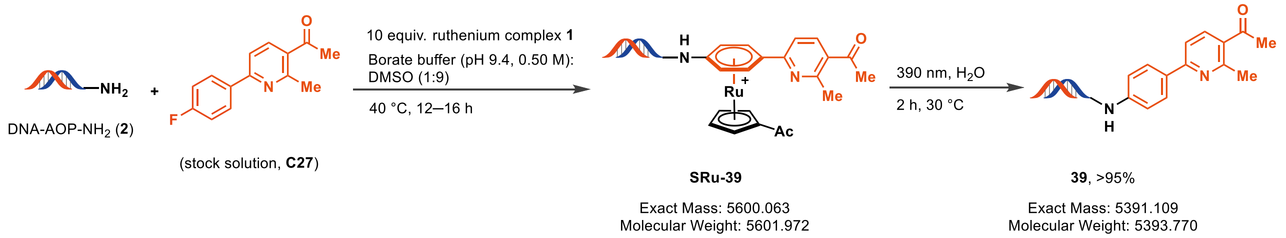

In situ formation of arene-ruthenium stock solution: Under an ambient atmosphere, a 1 mL glass GC vial equipped with a 6 mm Teflon-coated stirring bar was charged with ruthenium complex **1** (1.4 mg, 2.9 µmol, 1.0 equiv.). Next, a stock solution of 1-(6-(4-fluorophenyl)-2-methylpyridin-3-yl)ethan-1-one in DMC (294 µL, c = 0.10 M, 29 µmol, 10 equiv.) was added. The resulting reaction mixture was heated at 80 °C for 2 hours. After 2 hours, the reaction mixture was cooled to 23 °C. Next, the DMC was removed under a gentle stream of argon and 294 µL of DMSO were added to result an in situ formed stock solution of arene-ruthenium complex **C27** (294 µL, c = 0.01 M, assuming quantitative arene coordination to ruthenium).

Under an ambient atmosphere, the stock solution **SD-01** of DNA-AOP-NH_2 (**2**) in water (1.0 µL, c = 2.0 mM, 2.0 nmol, 1.0 equiv.) was added to a 1.5 mL Eppendorf tube, followed by sodium borate buffer (1.0 µL, pH 9.4, c = 0.50 M). To this mixture, 16 µL of DMSO was added and the solution was vortexed for 5 seconds. Next, the freshly prepared stock solution **C27** (2.0 µL, c = 0.01 M, 0.02 µmol, 10 equiv.) in DMSO was added.

The resulting reaction mixture was vortexed for 5 seconds, transferred to a thermocycler at 40 °C, and incubated for 16 hours at 800 rpm to yield the DNA-conjugate **SRu-39**. Next, the reaction mixture was diluted with 10 µL of Milli-Q water. To the reaction mixture was added the stock solution of NaCl in water (**SR-06** , 3.0 µL, c = 5.0 M, 10% volume of the total reaction volume), followed by cold ethanol (−20 °C, 99 µL) to precipitate the *N*-arylated ruthenium DNA conjugate **SRu-39**. The Eppendorf tube was placed in a freezer (−20 °C) for at least 1 hour, and then it was centrifuged at 4 °C and 11000 × g for at least 30 minutes. The supernatant was removed and the pellet was dried under air, then dissolved in 20 µL water to obtain the DNA-conjugate **SRu-39** (20 µL, c = 0.10 mM). Then, 1.0 µL of the above solution was diluted to 40 µL with water for LC–MS analysis.

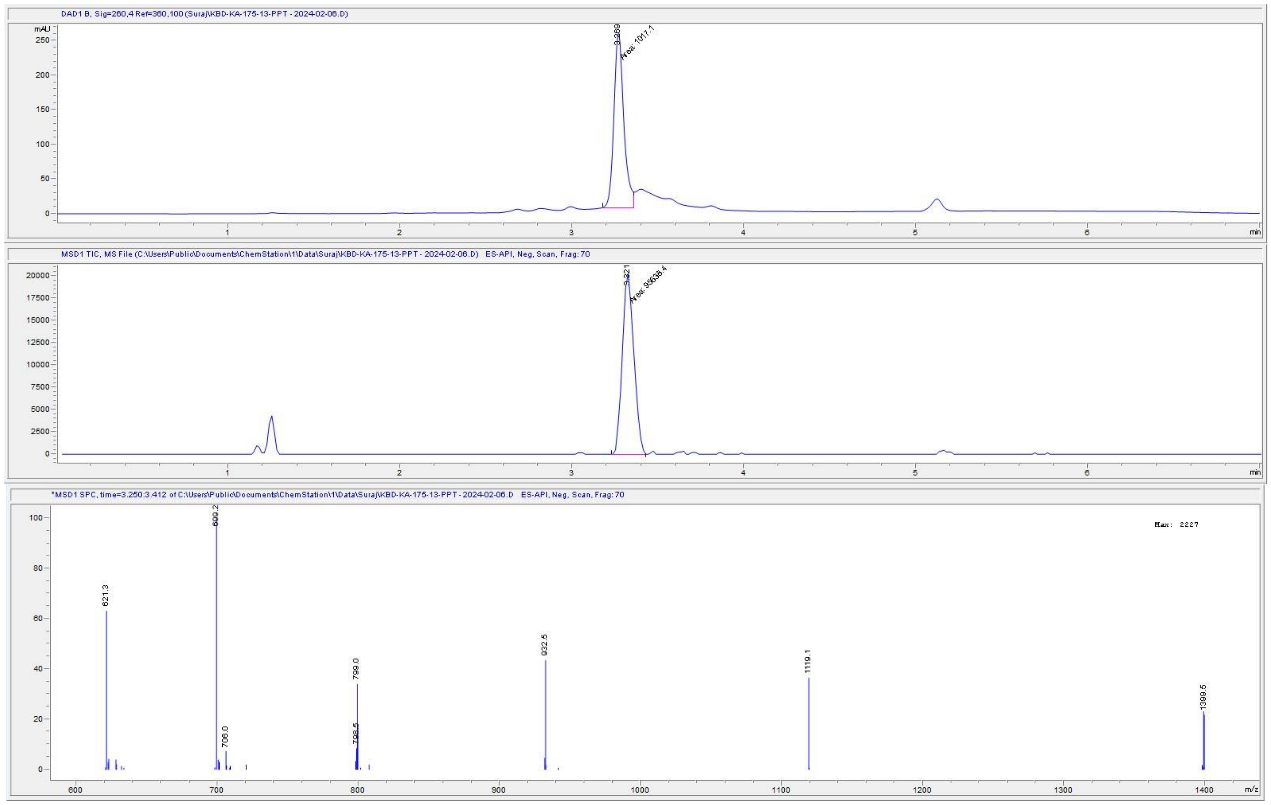

Figure S73. Analytical HPLC trace of **SRu-39** with HPLC method B. (Top) DAD chromatogram at 260 nm. (Middle) TIC chromatogram. (Bottom) Ionization of peak at 3.32 min. containing reaction product.

Decomplexation of SRu-39 to obtain product 39: Under an ambient atmosphere, the DNA-conjugate **SRu-39** stock solution in water (c = 0.10 mM, 20 µL) was irradiated with a 390 nm (40 W) Kessil lamp for 2 hours, while maintaining the temperature at approximately 30 °C through cooling with a fan. To the reaction mixture was added the stock solution of NaCl in water (**SR-06**, 2.0 µL, c = 5.0 M, 10% volume of the total reaction volume), followed by cold ethanol (−20 °C, 66 µL) to precipitate the DNA conjugate **39**. The Eppendorf tube was placed in the freezer (−20 °C) for at least 1 hour, and then it was centrifuged at 4 °C and 11000 × g for at least 30 minutes. The supernatant was removed, the pellet dried under air and dissolved in Milli-Q water to obtain the purified DNA-conjugate **39**. Then, 1 µL of the above solution was diluted to 40 µL with water for

LC–MS analysis. The yield of the DNA conjugate was calculated by measuring the integration of the peaks of the diode array detection (DAD) UV absorbance at 260 nm of the LC–MS trace, assuming complete DNA recovery and identical UV absorbance.

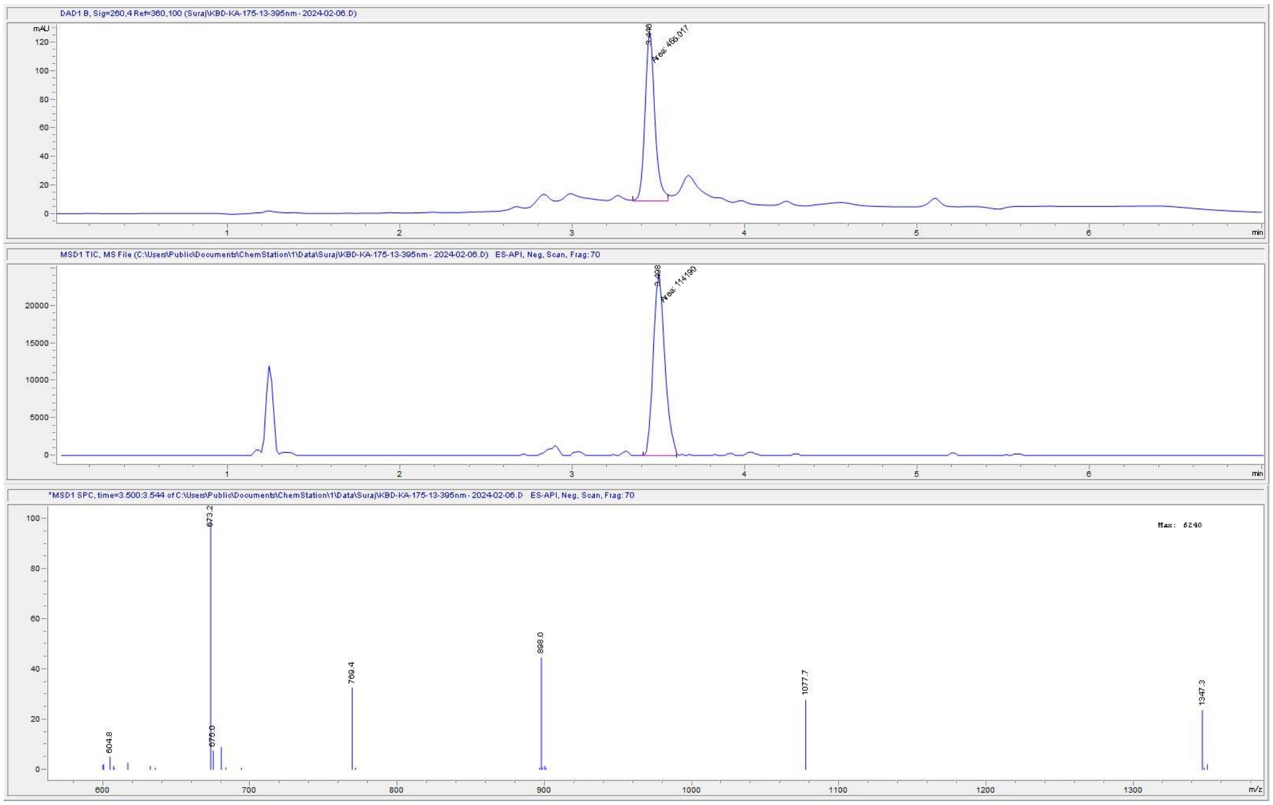

Figure S74. Analytical HPLC trace of **39** with HPLC method B. (Top) DAD chromatogram at 260 nm. (Middle) TIC chromatogram. (Bottom) Ionization of peak at 3.41 min. containing reaction product.

Synthesis of DNA-conjugate 40

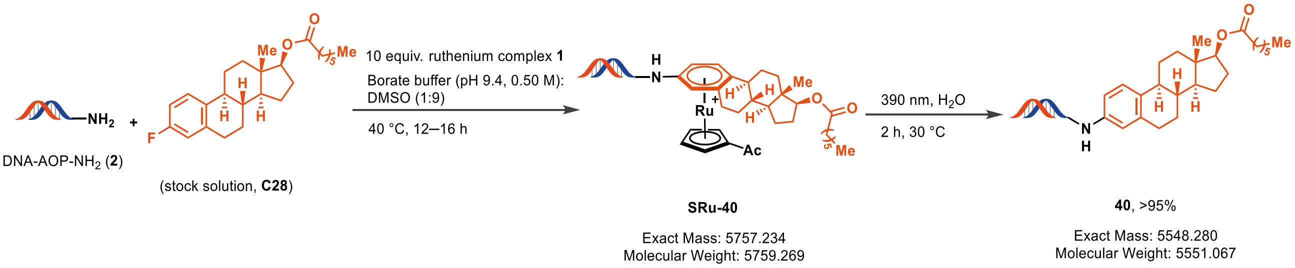

In situ formation of arene-ruthenium stock solution: Under an ambient atmosphere, a 1 mL glass GC vial equipped with a 6 mm Teflon-coated stirring bar was charged with ruthenium complex **1** (1.4 mg, 2.9 µmol, 1.0 equiv.). Next, a stock solution of the steroid derivative in DMC (294 µL, c = 0.10 M, 29 µmol, 10 equiv.) was added. The resulting reaction mixture was heated at 80 °C for 2 hours. After 2 hours, the reaction mixture was cooled to 23 °C. Next, the DMC was removed under a gentle stream of argon and 294 µL of DMSO were added to result an in situ formed stock solution of arene-ruthenium complex **C28** (294 µL, c =

0.01 M, assuming quantitative arene coordination to ruthenium).

Under an ambient atmosphere, the stock solution **SD-01** of DNA-AOP-NH_2 (**2**) in water (1.0 µL, c = 2.0 mM, 2.0 nmol, 1.0 equiv.) was added to a 1.5 mL Eppendorf tube, followed by sodium borate buffer (1.0 µL, pH 9.4, c = 0.50 M). To this mixture, 16 µL of DMSO was added and the solution was vortexed for 5 seconds. Next, the freshly prepared stock solution **C28** (2.0 µL, c = 0.01 M, 0.02 µmol, 10 equiv.) in DMSO was added. The resulting reaction mixture was vortexed for 5 seconds, transferred to a thermocycler at 40 °C, and incubated for 16 hours at 800 rpm to yield the DNA-conjugate **SRu-40**. Next, the reaction mixture was diluted with 10 µL of Milli-Q water. To the reaction mixture was added the stock solution of NaCl in water (**SR-06** , 3.0 µL, c = 5.0 M, 10% volume of the total reaction volume), followed by cold ethanol (−20 °C, 99 µL) to precipitate the *N*-arylated ruthenium DNA conjugate **SRu-40**. The Eppendorf tube was placed in a freezer (−20 °C) for at least 1 hour, and then it was centrifuged at 4 °C and 11000 × g for at least 30 minutes. The supernatant was removed and the pellet was dried under air, then dissolved in 20 µL water to obtain the DNA-conjugate **SRu-40** (20 µL, c = 0.10 mM). Then, 1.0 µL of the above solution was diluted to 40 µL with water for LC–MS analysis.

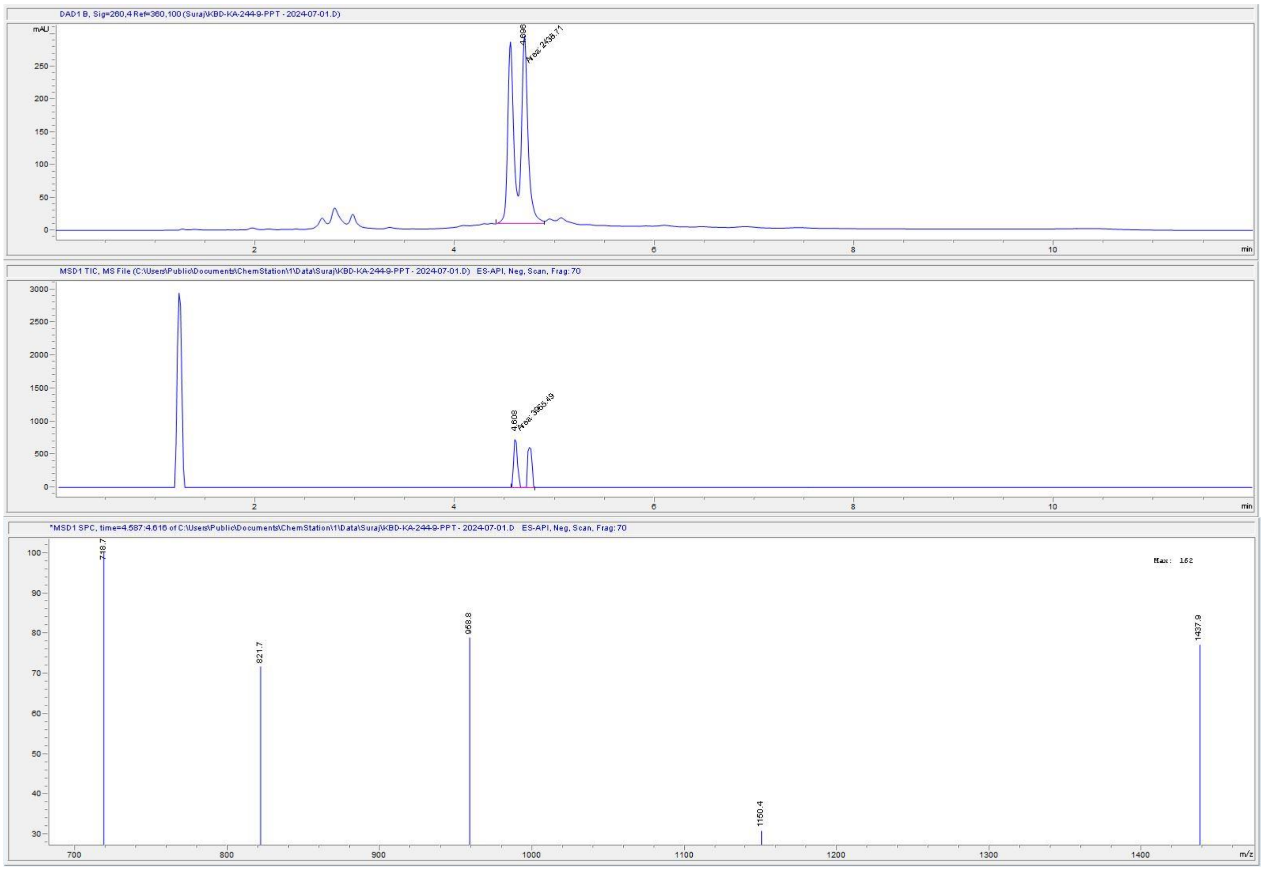

Figure S75. Analytical HPLC trace of **SRu-40** with HPLC method A. (Top) DAD chromatogram at 260 nm. (Middle) TIC chromatogram. (Bottom) Ionization of peak at 4.6–4.8 min. containing reaction product.

Decomplexation of SRu-40 to obtain product 40: Under an ambient atmosphere, the DNA-conjugate **SRu-**

40 stock solution in water (c = 0.10 mM, 20 µL) was irradiated with a 390 nm (40 W) Kessil lamp for 2 hours, while maintaining the temperature at approximately 30 °C through cooling with a fan. To the reaction mixture was added the stock solution of NaCl in water (**SR-06**, 2.0 µL, c = 5.0 M, 10% volume of the total reaction volume), followed by cold ethanol (−20 °C, 66 µL) to precipitate the DNA conjugate **40**. The Eppendorf tube was placed in the freezer (−20 °C) for at least 1 hour, and then it was centrifuged at 4 °C and 11000 × g for at least 30 minutes. The supernatant was removed, the pellet dried under air and dissolved in Milli-Q water to obtain the purified DNA-conjugate **40**. Then, 1 µL of the above solution was diluted to 40 µL with water for LC–MS analysis. The yield of the DNA conjugate was calculated by measuring the integration of the peaks of the diode array detection (DAD) UV absorbance at 260 nm of the LC–MS trace, assuming complete DNA recovery and identical UV absorbance.

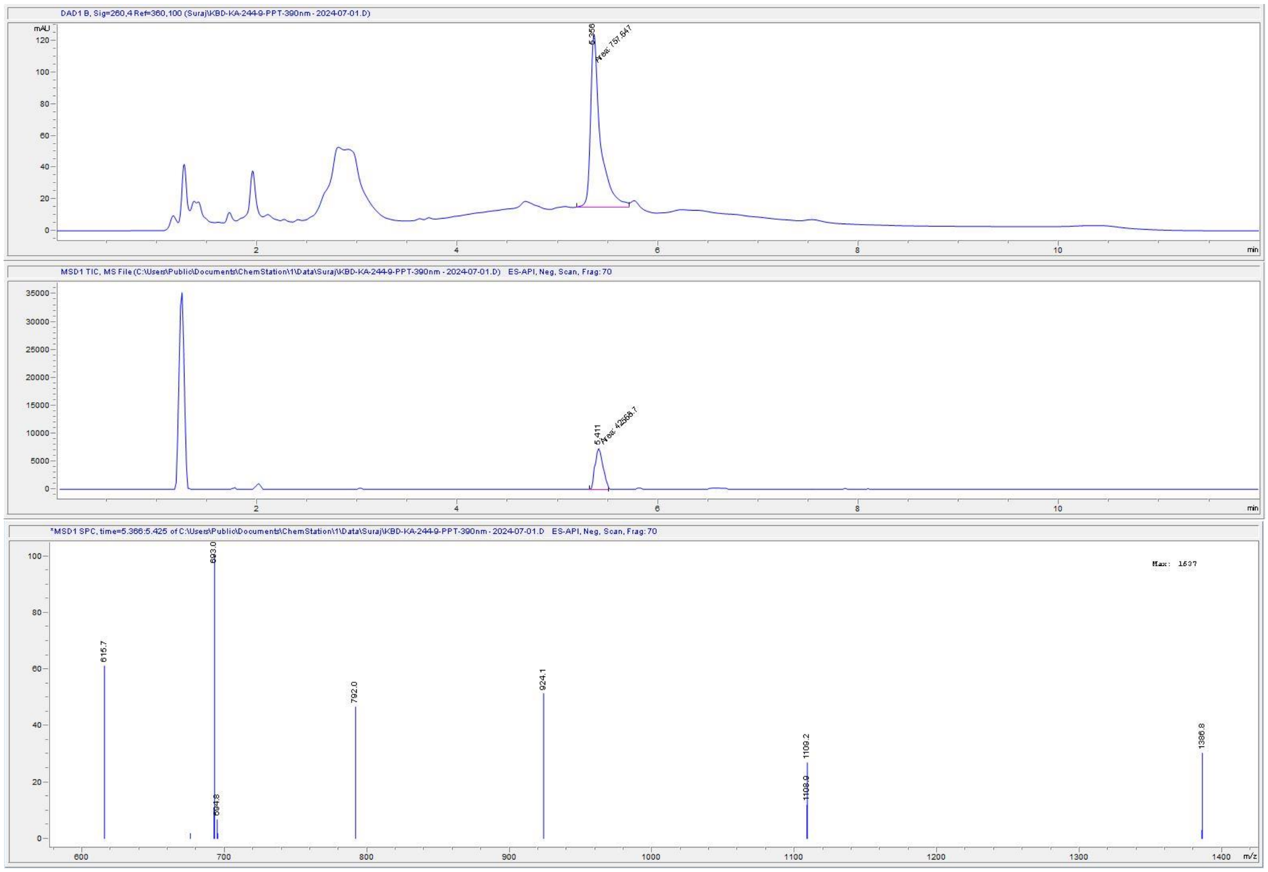

Figure S76. Analytical HPLC trace of **40** with HPLC method A. (Top) DAD chromatogram at 260 nm. (Middle) TIC chromatogram. (Bottom) Ionization of peak at 5.41 min. containing reaction product.

Synthesis of DNA-conjugate 41

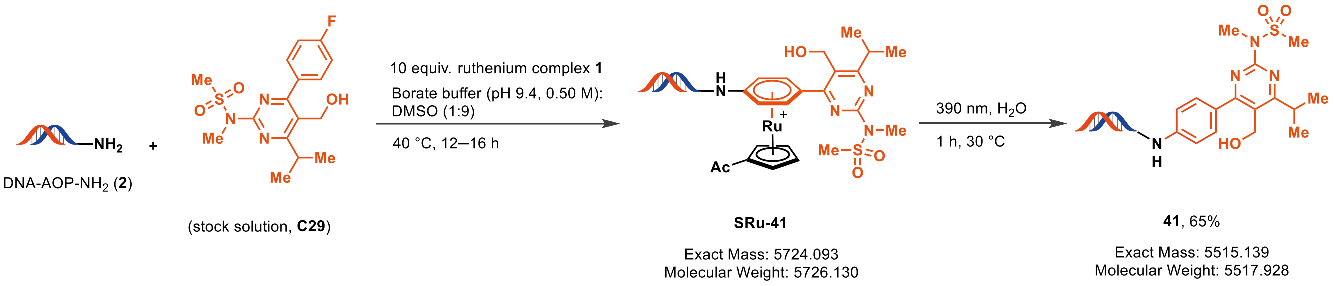

In situ formation of arene-ruthenium stock solution: Under an ambient atmosphere, a 1 mL glass GC vial equipped with a 6 mm Teflon-coated stirring bar was charged with ruthenium complex **1** (1.4 mg, 2.9 µmol, 1.0 equiv.). Next, a stock solution of *N*-(4-(4-fluorophenyl)-5-(hydroxymethyl)-6-isopropylpyrimidin-2-yl)-*N*-methylmethanesulfonamide in DMC (294 µL, c = 0.10 M, 29 µmol, 10 equiv.) was added. The resulting reaction mixture was heated at 80 °C for 2 hours. After 2 hours, the reaction mixture was cooled to 23 °C. Next, the DMC was removed under a gentle stream of argon and 294 µL of DMSO were added to result an in situ formed stock solution of arene-ruthenium complex **C29** (294 µL, c = 0.01 M, assuming quantitative arene coordination to ruthenium).

Under an ambient atmosphere, the stock solution **SD-01** of DNA-AOP-NH_2 (**2**) in water (1.0 µL, c = 2.0 mM, 2.0 nmol, 1.0 equiv.) was added to a 1.5 mL Eppendorf tube, followed by sodium borate buffer (1.0 µL, pH 9.4, c = 0.50 M). To this mixture, 16 µL of DMSO was added and the solution was vortexed for 5 seconds. Next, the freshly prepared stock solution **C29** (2.0 µL, c = 0.01 M, 0.02 µmol, 10 equiv.) in DMSO was added. The resulting reaction mixture was vortexed for 5 seconds, transferred to a thermocycler at 40 °C, and incubated for 16 hours at 800 rpm to yield the DNA-conjugate **SRu-41**. Next, the reaction mixture was diluted with 10 µL of Milli-Q water. To the reaction mixture was added the stock solution of NaCl in water (**SR-06** , 3.0 µL, c = 5.0 M, 10% volume of the total reaction volume), followed by cold ethanol (−20 °C, 99 µL) to precipitate the *N*-arylated ruthenium DNA conjugate **SRu-41**. The Eppendorf tube was placed in a freezer (−20 °C) for at least 1 hour, and then it was centrifuged at 4 °C and 11000 × g for at least 30 minutes. The supernatant was removed and the pellet was dried under air, then dissolved in 20 µL water to obtain the DNA-conjugate **SRu-41** (20 µL, c = 0.10 mM). Then, 1.0 µL of the above solution was diluted to 40 µL with water for LC–MS analysis.

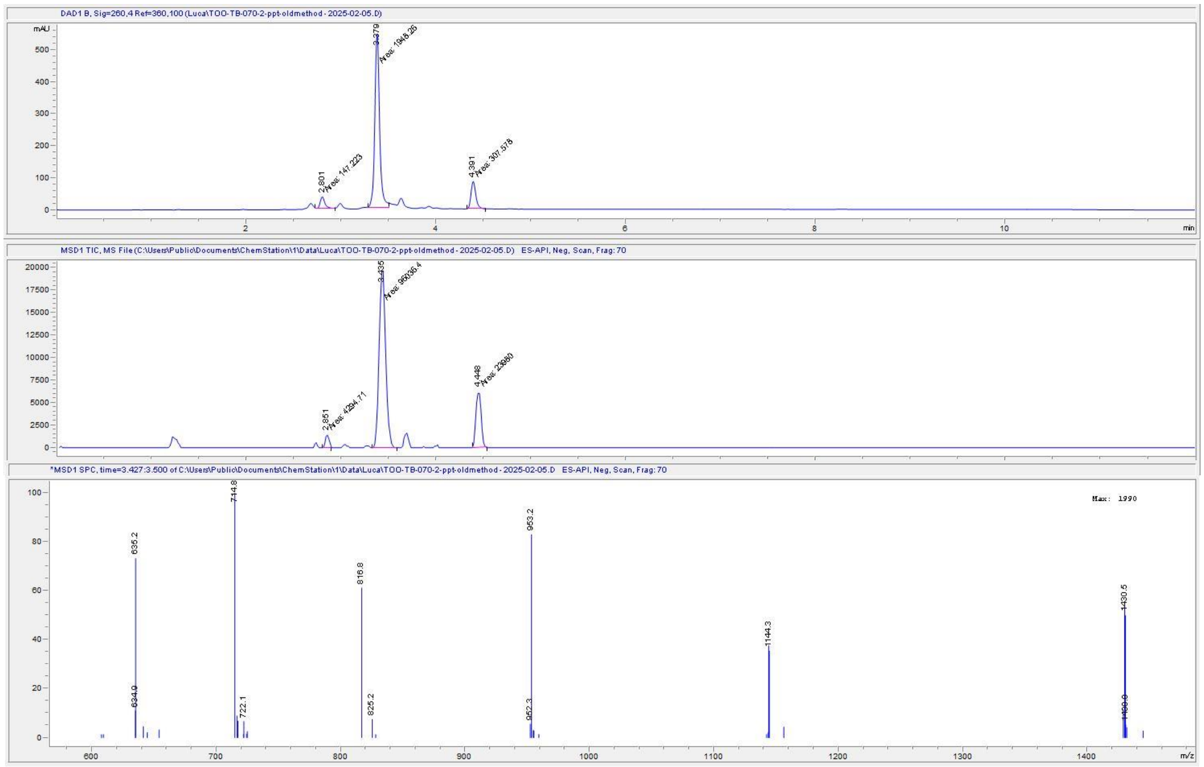

Figure S77. Analytical HPLC trace of **Ru-29** with HPLC method A. (Top) DAD chromatogram at 260 nm. (Middle) TIC chromatogram. (Bottom) Ionization of peak at 3.43 min. containing reaction product.

Decomplexation of SRu-41 to obtain product 41: Under an ambient atmosphere, the DNA-conjugate **SRu-41** stock solution in water (c = 0.10 mM, 20 µL) was irradiated with a 390 nm (40 W) Kessil lamp for 1 hours, while maintaining the temperature at approximately 30 °C through cooling with a fan. To the reaction mixture was added the stock solution of NaCl in water (**SR-06**, 2.0 µL, c = 5.0 M, 10% volume of the total reaction volume), followed by cold ethanol (−20 °C, 66 µL) to precipitate the DNA conjugate **41**. The Eppendorf tube was placed in the freezer (−20 °C) for at least 1 hour, and then it was centrifuged at 4 °C and 11000 × g for at least 30 minutes. The supernatant was removed, the pellet dried under air and dissolved in Milli-Q water to obtain the purified DNA-conjugate **41**. Then, 1 µL of the above solution was diluted to 40 µL with water for LC–MS analysis. The yield of the DNA conjugate was calculated by measuring the integration of the peaks of the diode array detection (DAD) UV absorbance at 260 nm of the LC–MS trace, assuming complete DNA recovery and identical UV absorbance.

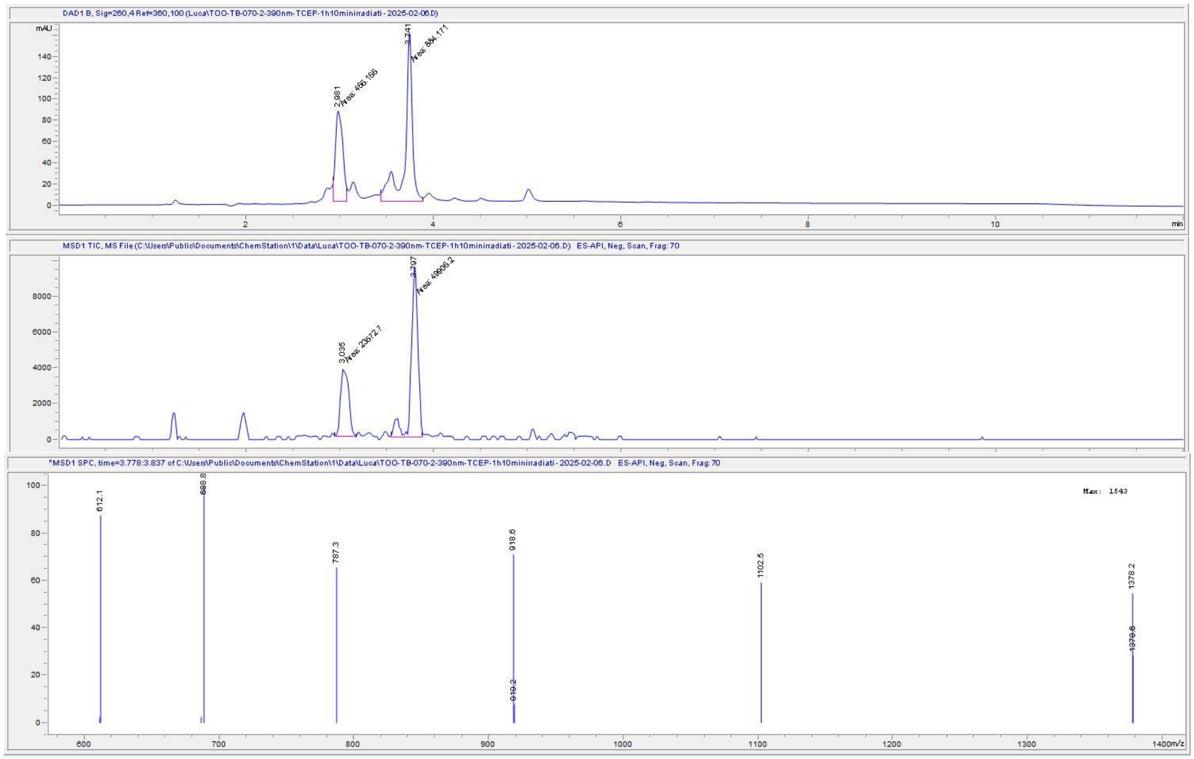

Figure S78. Analytical HPLC trace of **41** with HPLC method A. (Top) DAD chromatogram at 260 nm. (Middle) TIC chromatogram. (Bottom) Ionization of peak at 3.80 min. containing reaction product (The small decomplexed peak of **SRu-41** ~3.50 min. is also considered in the yield determination).

Synthesis of DNA-conjugate 42

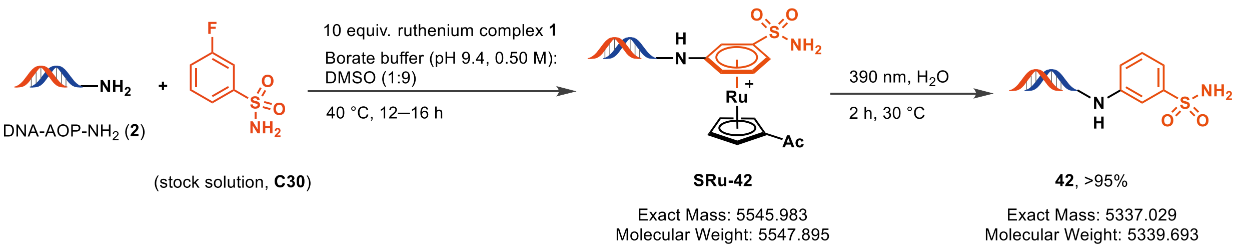

In situ formation of arene-ruthenium stock solution: Under an ambient atmosphere, a 1 mL glass GC vial equipped with a 6 mm Teflon-coated stirring bar was charged with ruthenium complex **1** (1.4 mg, 2.9 µmol, 1.0 equiv.). Next, a stock solution of 3-fluorobenzenesulfonamide in DMC (294 µL, c = 0.10 M, 29 µmol, 10 equiv.) was added. The resulting reaction mixture was heated at 80 °C for 2 hours. After 2 hours, the reaction mixture was cooled to 23 °C. Next, the DMC was removed under a gentle stream of argon and 294 µL of DMSO were added to result an in situ formed stock solution of arene-ruthenium complex **C30** (294 µL, c = 0.01 M, assuming quantitative arene coordination to ruthenium).

Under an ambient atmosphere, the stock solution **SD-01** of DNA-AOP-NH_2 (**2**) in water (1.0 µL, c = 2.0 mM, 2.0 nmol, 1.0 equiv.) was added to a 1.5 mL Eppendorf tube, followed by sodium borate buffer (1.0 µL, pH 9.4, c = 0.50 M). To this mixture, 16 µL of DMSO was added and the solution was vortexed for 5 seconds.

Next, the freshly prepared stock solution **C30** (2.0 µL, c = 0.01 M, 0.02 µmol, 10 equiv.) in DMSO was added. The resulting reaction mixture was vortexed for 5 seconds, transferred to a thermocycler at 40 °C, and incubated for 16 hours at 800 rpm to yield the DNA-conjugate **SRu-42**. Next, the reaction mixture was diluted with 10 µL of Milli-Q water. To the reaction mixture was added the stock solution of NaCl in water (**SR-06** , 3.0 µL, c = 5.0 M, 10% volume of the total reaction volume), followed by cold ethanol (−20 °C, 99 µL) to precipitate the *N*-arylated ruthenium DNA conjugate **SRu-42**. The Eppendorf tube was placed in a freezer (−20 °C) for at least 1 hour, and then it was centrifuged at 4 °C and 11000 × g for at least 30 minutes. The supernatant was removed and the pellet was dried under air, then dissolved in 20 µL water to obtain the DNA-conjugate **SRu-42** (20 µL, c = 0.10 mM). Then, 1.0 µL of the above solution was diluted to 40 µL with water for LC–MS analysis.

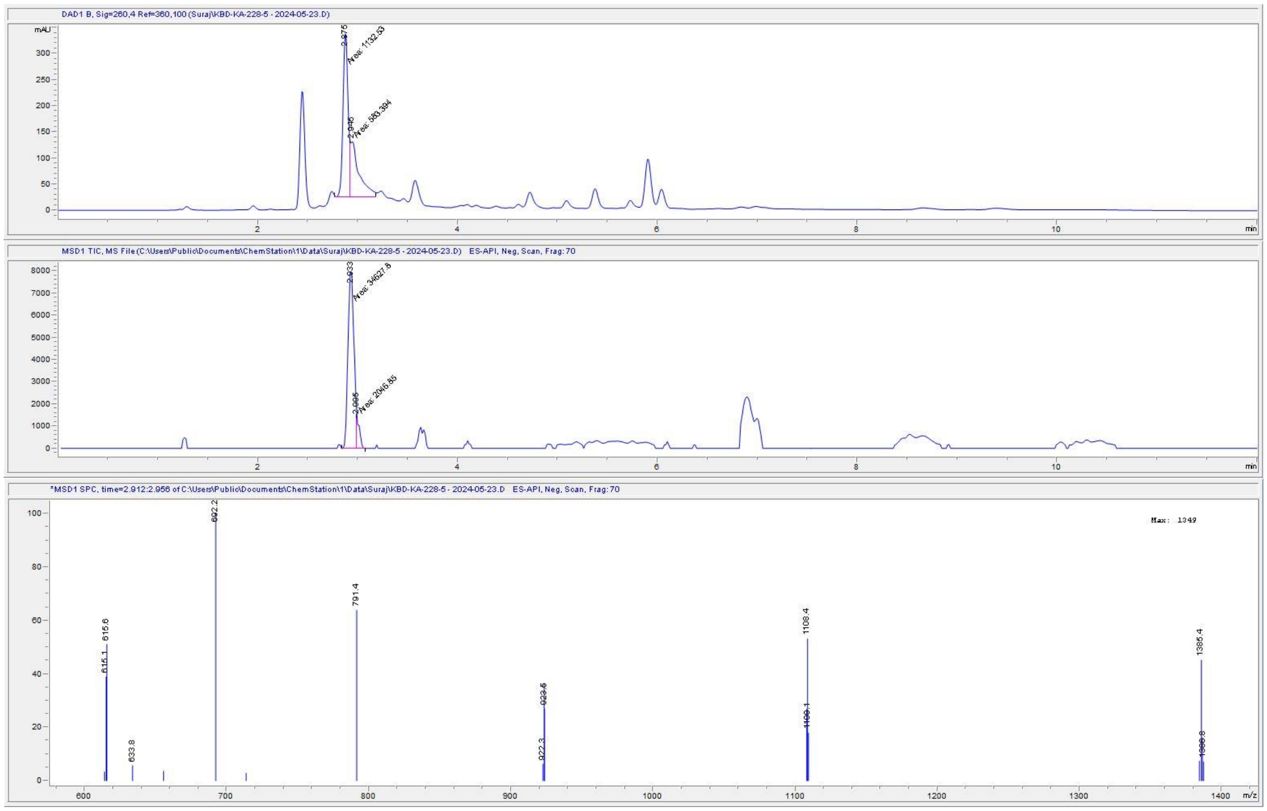

Figure S79. Analytical HPLC trace of **SRu-42** with HPLC method A. (Top) DAD chromatogram at 260 nm. (Middle) TIC chromatogram. (Bottom) Ionization of peak at 2.93 min. containing reaction product.

Decomplexation of SRu-42 to obtain product 42: Under an ambient atmosphere, the DNA-conjugate **SRu-42** stock solution in water (c = 0.10 mM, 20 µL) was irradiated with a 390 nm (40 W) Kessil lamp for 2 hours, while maintaining the temperature at approximately 30 °C through cooling with a fan. To the reaction mixture was added the stock solution of NaCl in water (**SR-06**, 2.0 µL, c = 5.0 M, 10% volume of the total reaction volume), followed by cold ethanol (−20 °C, 66 µL) to precipitate the DNA conjugate **42**. The Eppendorf tube was placed in the freezer (−20 °C) for at least 1 hour, and then it was centrifuged at 4 °C and 11000 × g for at least 30 minutes. The supernatant was removed, the pellet dried under air and dissolved in Milli-Q water to

obtain the purified DNA-conjugate **42**. Then, 1 µL of the above solution was diluted to 40 µL with water for LC–MS analysis. The yield of the DNA conjugate was calculated by measuring the integration of the peaks of the diode array detection (DAD) UV absorbance at 260 nm of the LC–MS trace, assuming complete DNA recovery and identical UV absorbance.

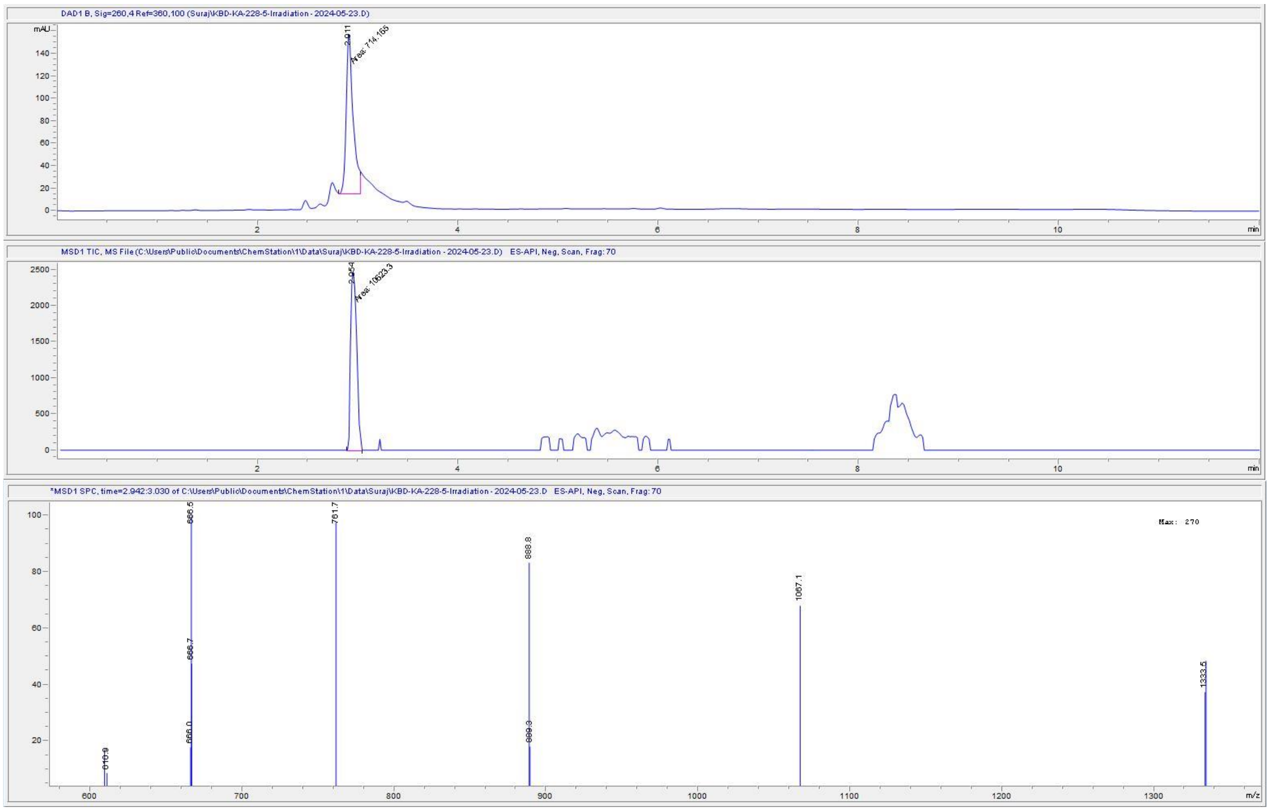

Figure S80. Analytical HPLC trace of **42** with HPLC method A. (Top) DAD chromatogram at 260 nm. (Middle) TIC chromatogram. (Bottom) Ionization of peak at 2.95 min. containing reaction product.

Synthesis of DNA-conjugate 43

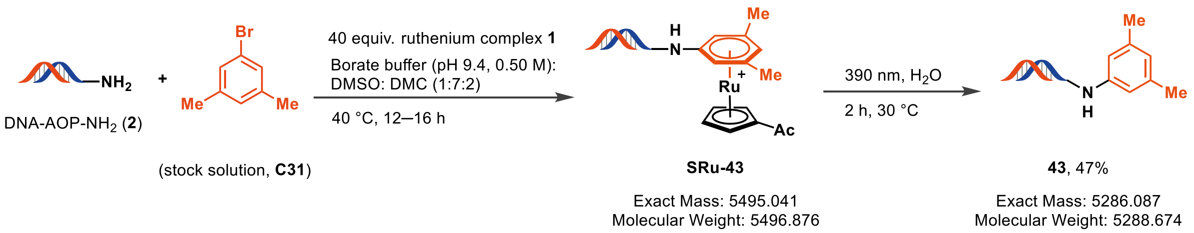

In situ formation of arene-ruthenium stock solution: Under an ambient atmosphere, a 1 mL glass GC vial equipped with a 6 mm Teflon-coated stirring bar was charged with a stock solution of ruthenium complex **1** in DMC (50 µL, c = 40 mM, 2.0 µmol). Next, a stock solution of 1-bromo-3,5-dimethylbenzene in DMC (50 µL, c = 0.20 M, 10 µmol, 5.0 equiv.) was added. The resulting reaction mixture was heated at 80 °C for 2 hours. After 2 hours, the reaction mixture was cooled to 23 °C, to result an in situ formed stock solution of arene-ruthenium complex **C31** in DMC (100 µL, c = 0.02 M, assuming quantitative arene coordination to ruthenium).

Under an ambient atmosphere, a stock solution of DNA-AOP-NH_2 (**2**) (1.0 µL, c = 1.0 mM, 1.0 nmol, 1.0 equiv.) in sodium borate buffer (pH 9.4, c = 0.25 M) was added to a 1.5 mL Eppendorf tube. To this mixture, 7 µL of DMSO was added and the solution was vortexed for 5 seconds. The freshly prepared stock solution **C31** (2.0 µL, c = 0.02 M, 0.04 µmol, 40 equiv.) in DMC was then added. The resulting reaction mixture was vortexed for 5 seconds, transferred to a thermocycler at 40 °C, and incubated for 16 hours at 800 rpm to yield the DNA-conjugate **SRu-43**. Next, the reaction mixture was diluted with 10 µL of Milli-Q water. To the reaction mixture was added the stock solution of NaCl in water (**SR-06** , 2.0 µL, c = 5.0 M, 10% volume of the total reaction volume), followed by cold ethanol (−20 °C, 66 µL) to precipitate the *N*-arylated ruthenium DNA conjugate **SRu-43**. The Eppendorf tube was placed in a freezer (−20 °C) for at least 1 hour, and then it was centrifuged at 4 °C and 11000 × g for at least 30 minutes. The supernatant was removed and the pellet was dried under air, then dissolved in 10 µL water to obtain the DNA-conjugate **SRu-43** (10 µL, c = 0.10 mM). Then, 1.0 µL of the above solution was diluted to 40 µL with water for LC–MS analysis.

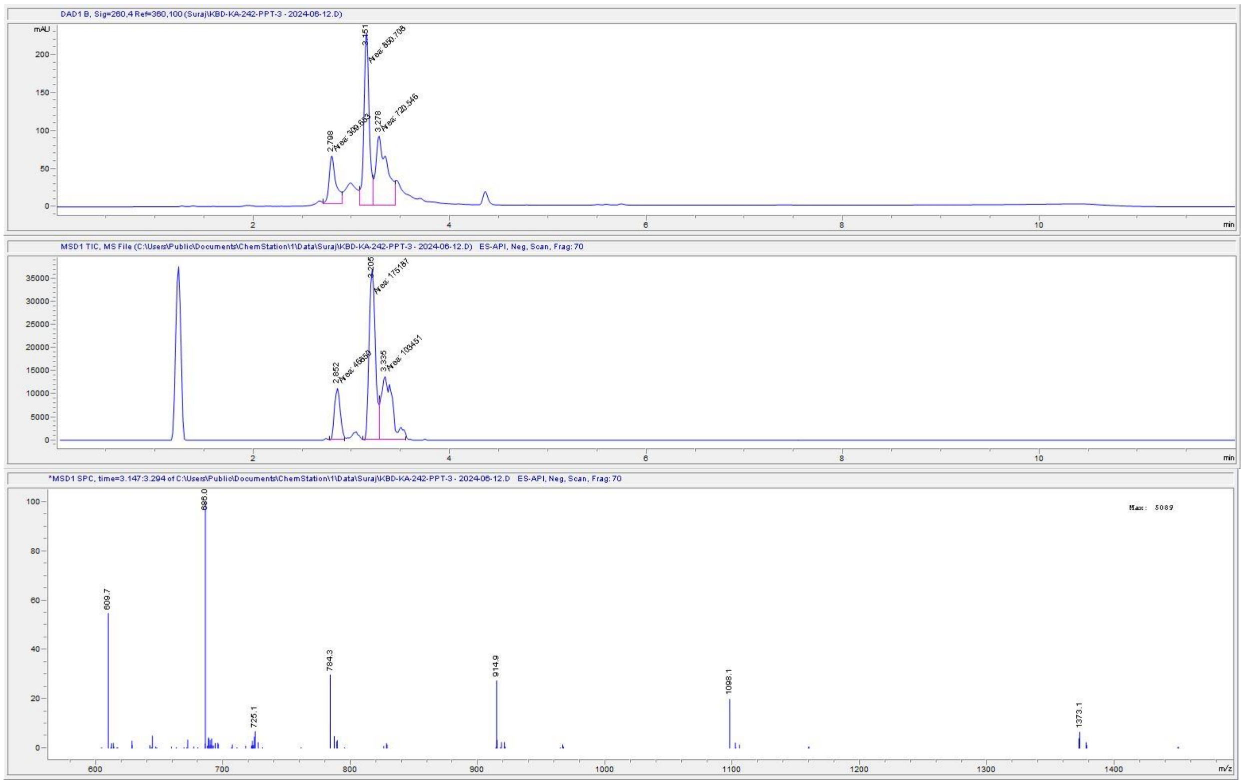

Figure S81. Analytical HPLC trace of **SRu-43** with HPLC method A. (Top) DAD chromatogram at 260 nm. (Middle) TIC chromatogram. (Bottom) Ionization of peak at 3.20 min. containing reaction product.

Decomplexation of SRu-43 to obtain product 43: Under an ambient atmosphere, the DNA-conjugate **SRu-43** stock solution in water (c = 0.10 mM, 10 µL) was irradiated with a 390 nm (40 W) Kessil lamp for 2 hours, while maintaining the temperature at approximately 30 °C through cooling with a fan. To the reaction mixture was added the stock solution of NaCl in water (**SR-06**, 1.0 µL, c = 5.0 M, 10% volume of the total reaction volume), followed by cold ethanol (−20 °C, 33 µL) to precipitate the DNA conjugate **43**. The Eppendorf tube was placed in the freezer (−20 °C) for at least 1 hour, and then it was centrifuged at 4 °C and 11000 × g for at

least 30 minutes. The supernatant was removed, the pellet dried under air and dissolved in Milli-Q water to obtain the purified DNA-conjugate **43**. Then, 1 µL of the above solution was diluted to 40 µL with water for LC–MS analysis. The yield of the DNA conjugate was calculated by measuring the integration of the peaks of the diode array detection (DAD) UV absorbance at 260 nm of the LC–MS trace, assuming complete DNA recovery and identical UV absorbance.

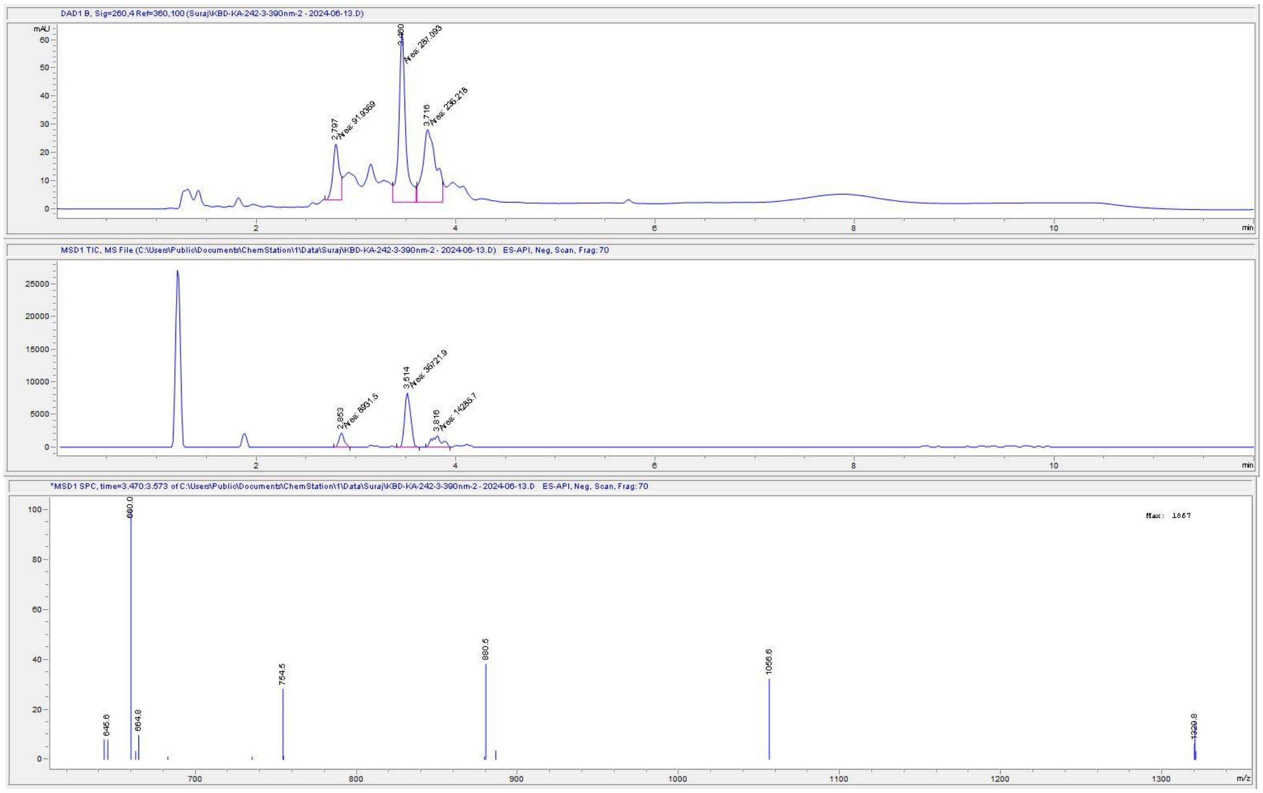

Figure S82. Analytical HPLC trace of **43** with HPLC method A. (Top) DAD chromatogram at 260 nm. (Middle) TIC chromatogram. (Bottom) Ionization of peak at 3.45 min. containing reaction product.

Synthesis of DNA-conjugate 44

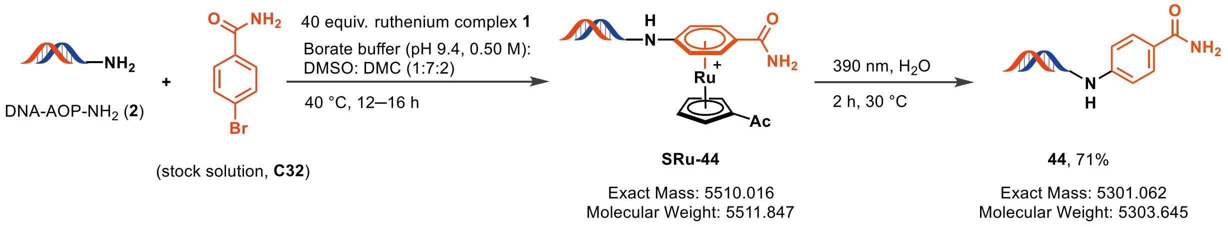

In situ formation of arene-ruthenium stock solution: Under an ambient atmosphere, a 1 mL glass GC vial equipped with a 6 mm Teflon-coated stirring bar was charged with a stock solution of ruthenium complex **1** in DMC (50 µL, c = 40 mM, 2.0 µmol). Next, a stock solution of 4-bromobenzamide in DMC (50 µL, c = 0.20 M, 10 µmol, 5.0 equiv.) was added. The resulting reaction mixture was heated at 80 °C for 2 hours. After 2 hours, the reaction mixture was cooled to 23 °C, to result an in situ formed stock solution of arene-ruthenium

complex **C32** in DMC (100 µL, c = 0.02 M, assuming quantitative arene coordination to ruthenium).

Under an ambient atmosphere, a stock solution of DNA-AOP-NH_2 (**2**) (1.0 µL, c = 1.0 mM, 1.0 nmol, 1.0 equiv.) in sodium borate buffer (pH 9.4, c = 0.25 M) was added to a 1.5 mL Eppendorf tube. To this mixture, 7 µL of DMSO was added and the solution was vortexed for 5 seconds. The freshly prepared stock solution **C32** (2.0 µL, c = 0.02 M, 0.04 µmol, 40 equiv.) in DMC was then added. The resulting reaction mixture was vortexed for 5 seconds, transferred to a thermocycler at 40 °C, and incubated for 16 hours at 800 rpm to yield the DNA-conjugate **SRu-44**. Next, the reaction mixture was diluted with 10 µL of Milli-Q water. To the reaction mixture was added the stock solution of NaCl in water (**SR-06** , 2.0 µL, c = 5.0 M, 10% volume of the total reaction volume), followed by cold ethanol (−20 °C, 66 µL) to precipitate the *N*-arylated ruthenium DNA conjugate **SRu-44**. The Eppendorf tube was placed in a freezer (−20 °C) for at least 1 hour, and then it was centrifuged at 4 °C and 11000 × g for at least 30 minutes. The supernatant was removed and the pellet was dried under air, then dissolved in 10 µL water to obtain the DNA-conjugate **SRu-44** (10 µL, c = 0.10 mM). Then, 1.0 µL of the above solution was diluted to 40 µL with water for LC–MS analysis.

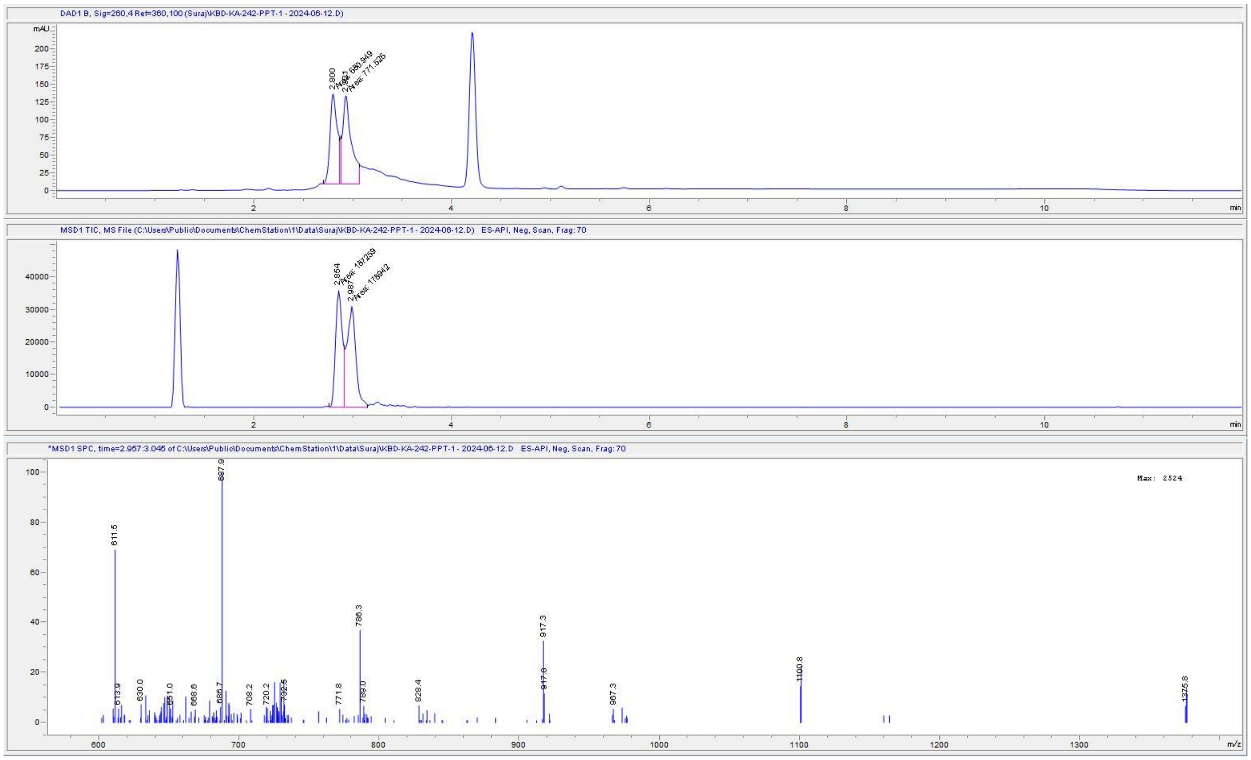

Figure S83. Analytical HPLC trace of **SRu-44** with HPLC method A. (Top) DAD chromatogram at 260 nm. (Middle) TIC chromatogram. (Bottom) Ionization of peak at 2.98 min. containing reaction product.

Decomplexation of SRu-44 to obtain product 44: Under an ambient atmosphere, the DNA-conjugate **SRu-44** stock solution in water (c = 0.10 mM, 10 µL) was irradiated with a 390 nm (40 W) Kessil lamp for 2 hours, while maintaining the temperature at approximately 30 °C through cooling with a fan. To the reaction mixture was added the stock solution of NaCl in water (**SR-06**, 1.0 µL, c = 5.0 M, 10% volume of the total reaction volume), followed by cold ethanol (−20 °C, 33 µL) to precipitate the DNA conjugate **44**. The Eppendorf tube

was placed in the freezer (−20 °C) for at least 1 hour, and then it was centrifuged at 4 °C and 11000 × g for at least 30 minutes. The supernatant was removed, the pellet dried under air and dissolved in Milli-Q water to obtain the purified DNA-conjugate **44**. Then, 1 µL of the above solution was diluted to 40 µL with water for LC–MS analysis. The yield of the DNA conjugate was calculated by measuring the integration of the peaks of the diode array detection (DAD) UV absorbance at 260 nm of the LC–MS trace, assuming complete DNA recovery and identical UV absorbance.

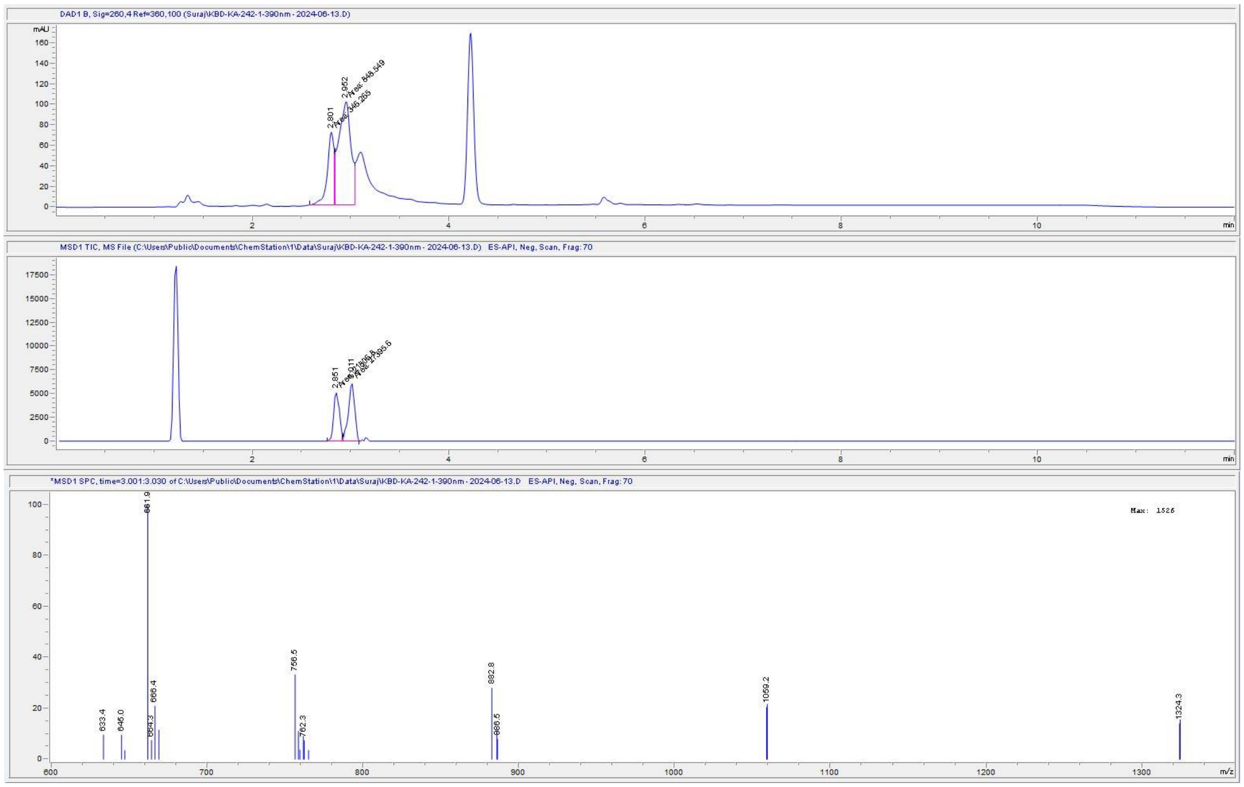

Figure S84. Analytical HPLC trace of **44** with HPLC method A. (Top) DAD chromatogram at 260 nm. (Middle) TIC chromatogram. (Bottom) Ionization of peak at 3.01 min. containing reaction product.

Synthesis of DNA-conjugate 45

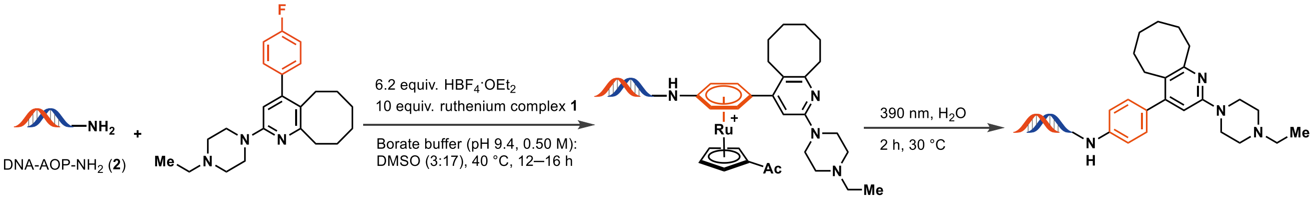

4.8 equiv.
(stock solution, **C33**)

SRu-45
Exact Mass: 5738.215
Molecular Weight: 5740.230

45, >95%
Exact Mass: 5529.261
Molecular Weight: 5532.028

In situ formation of arene-ruthenium stock solution: Under an ambient atmosphere, a 1 mL glass GC vial equipped with a 6 mm Teflon-coated stirring bar was charged with ruthenium complex **1** (1.0 mg, 2.1 µmol, 1.0 equiv.) and then 100 µL of DMC was added to the reaction vial. In a separate Eppendorf tube was added

a stock solution of $HBF_4 \cdot OEt_2$ (50 µL, c = 0.26 M, 13 µmol, 6.2 equiv.), followed by a stock solution of 2-(4-ethylpiperazin-1-yl)-4-(4-fluorophenyl)-5, 6, 7, 8, 9, 10-hexahydrocycloocta[*b*]pyridine in DMC (50 µL, c = 0.20 M, 10 µmol, 4.8 equiv.). The contents of the Eppendorf tube was then transferred to the reaction vial. The vial was then closed with a screw cap and the resulting reaction mixture was heated at 80 °C for 2 hours. After 2 hours, the reaction mixture was cooled to 23 °C and the DMC was removed under a gentle stream of argon and 200 µL of DMSO was added to result in an in situ formed stock solution of arene-ruthenium complex **C33** in DMSO (200 µL, c = 0.01 M, assuming quantitative arene coordination to ruthenium).

Under an ambient atmosphere, the stock solution **SD-01** of DNA-AOP-NH_2 (**2**) in water (1.0 µL, c = 2.0 mM, 2.0 nmol, 1.0 equiv.) was added to a 1.5 mL Eppendorf tube, followed by sodium borate buffer (2.0 µL, pH 9.4, c = 0.50 M). To this mixture, 15 µL of DMSO was added and the solution was vortexed for 5 seconds. Next, the freshly prepared stock solution **C33** (2.0 µL, c = 0.01 M, 0.02 µmol, 10 equiv.) in DMSO was added. The resulting reaction mixture was vortexed for 5 seconds, transferred to a thermocycler at 40 °C, and incubated for 16 hours at 800 rpm to yield the DNA-conjugate **SRu-45**. Next, the reaction mixture was diluted with 10 µL of Milli-Q water. To the reaction mixture was added the stock solution of NaCl in water (**SR-06** , 3.0 µL, c = 5.0 M, 10% volume of the total reaction volume), followed by cold ethanol (−20 °C, 99 µL) to precipitate the *N*-arylated ruthenium DNA conjugate **SRu-45**. The Eppendorf tube was placed in a freezer (−20 °C) for at least 1 hour, and then it was centrifuged at 4 °C and 11000 × g for at least 30 minutes. The supernatant was removed and the pellet was dried under air, then dissolved in 20 µL water to obtain the DNA-conjugate **SRu-45** (20 µL, c = 0.10 mM). Then, 1.0 µL of the above solution was diluted to 40 µL with water for LC–MS analysis.

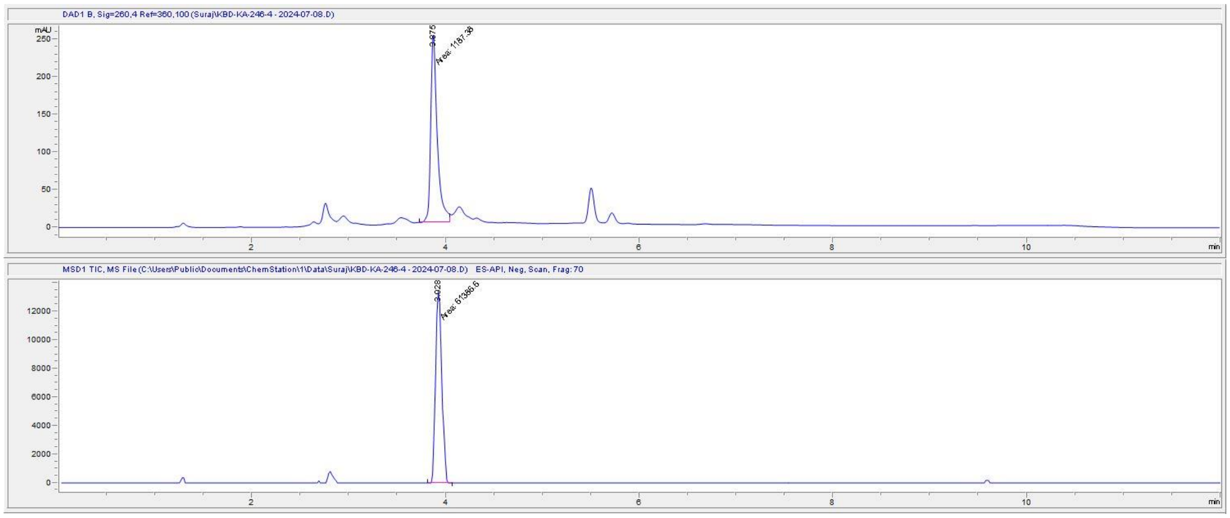

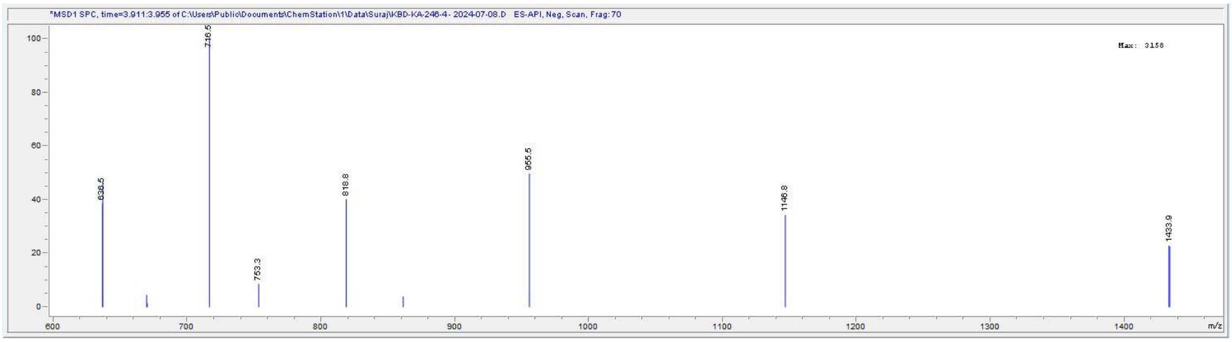

Figure S85. Analytical HPLC trace of **SRu-45** with HPLC method A. (Top) DAD chromatogram at 260 nm. (Middle) TIC chromatogram. (Bottom) Ionization of peak at 3.93 min. containing reaction product.

Decomplexation of SRu-45 to obtain product 45: Under an ambient atmosphere, the DNA-conjugate **SRu-45** stock solution in water (c = 0.10 mM, 20 µL) was irradiated with a 390 nm (40 W) Kessil lamp for 2 hours, while maintaining the temperature at approximately 30 °C through cooling with a fan. To the reaction mixture was added the stock solution of NaCl in water (**SR-06**, 2.0 µL, c = 5.0 M, 10% volume of the total reaction volume), followed by cold ethanol (−20 °C, 66 µL) to precipitate the DNA conjugate **45**. The Eppendorf tube was placed in the freezer (−20 °C) for at least 1 hour, and then it was centrifuged at 4 °C and 11000 × g for at least 30 minutes. The supernatant was removed, the pellet dried under air and dissolved in Milli-Q water to obtain the purified DNA-conjugate **45**. Then, 1 µL of the above solution was diluted to 40 µL with water for LC–MS analysis. The yield of the DNA conjugate was calculated by measuring the integration of the peaks of the diode array detection (DAD) UV absorbance at 260 nm of the LC–MS trace, assuming complete DNA recovery and identical UV absorbance.

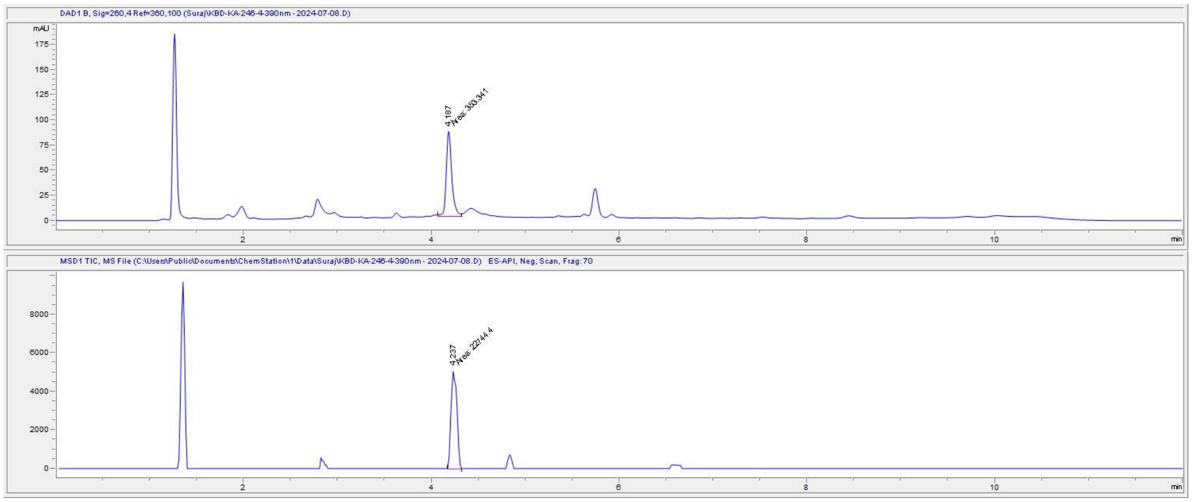

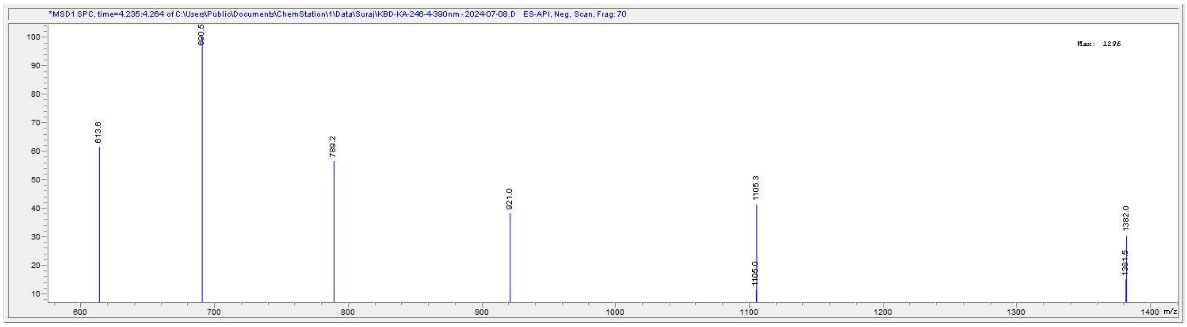

Figure S86. Analytical HPLC trace of **45** with HPLC method A. (Top) DAD chromatogram at 260 nm. (Middle) TIC chromatogram. (Bottom) Ionization of peak at 4.24 min. containing reaction product.

Synthesis of DNA-conjugate 46

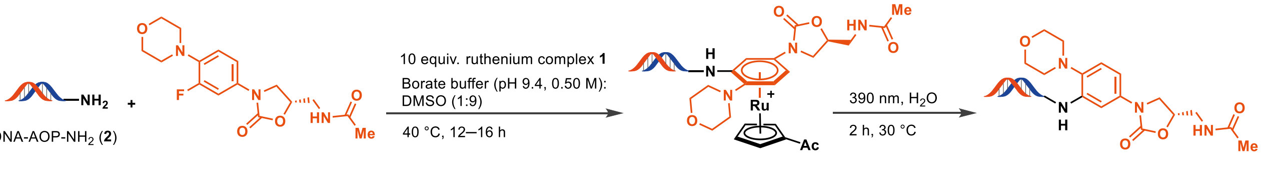

In situ formation of arene-ruthenium stock solution: Under an ambient atmosphere, a 1 mL glass GC vial equipped with a 6 mm Teflon-coated stirring bar was charged with ruthenium complex **1** (1.4 mg, 2.9 µmol, 1.0 equiv.). Next, a stock solution of (*S*)-*N*-((3-(3-fluoro-4-morpholinophenyl)-2-oxooxazolidin-5-yl)methyl)acetamide in DMC (294 µL, c = 0.10 M, 29 µmol, 10 equiv.) was added. The resulting reaction mixture was heated at 80 °C for 2 hours. After 2 hours, the reaction mixture was cooled to 23 °C. Next, the DMC was removed under a gentle stream of argon and 294 µL of DMSO were added to result an in situ formed stock solution of arene-ruthenium complex **C34** (294 µL, c = 0.01 M, assuming quantitative arene coordination to ruthenium).

Under an ambient atmosphere, the stock solution **SD-01** of DNA-AOP-NH_2 (**2**) in water (1.0 µL, c = 2.0 mM, 2.0 nmol, 1.0 equiv.) was added to a 1.5 mL Eppendorf tube, followed by sodium borate buffer (1.0 µL, pH 9.4, c = 0.50 M). To this mixture, 16 µL of DMSO was added and the solution was vortexed for 5 seconds. Next, the freshly prepared stock solution **C34** (2.0 µL, c = 0.01 M, 0.02 µmol, 10 equiv.) in DMSO was added. The resulting reaction mixture was vortexed for 5 seconds, transferred to a thermocycler at 40 °C, and incubated for 16 hours at 800 rpm to yield the DNA-conjugate **SRu-46**. Next, the reaction mixture was diluted with 10 µL of Milli-Q water. To the reaction mixture was added the stock solution of NaCl in water (**SR-06** , 3.0 µL, c = 5.0 M, 10% volume of the total reaction volume), followed by cold ethanol (−20 °C, 99 µL) to precipitate the *N*-arylated ruthenium DNA conjugate **SRu-46**. The Eppendorf tube was placed in a freezer (−20 °C) for at least 1 hour, and then it was centrifuged at 4 °C and 11000 × g for at least 30 minutes. The supernatant was removed and the pellet was dried under air, then dissolved in 20 µL water to obtain the DNA-conjugate **SRu-46** (20 µL, c = 0.10 mM). Then, 1.0 µL of the above solution was diluted to 40 µL with

water for LC–MS analysis.

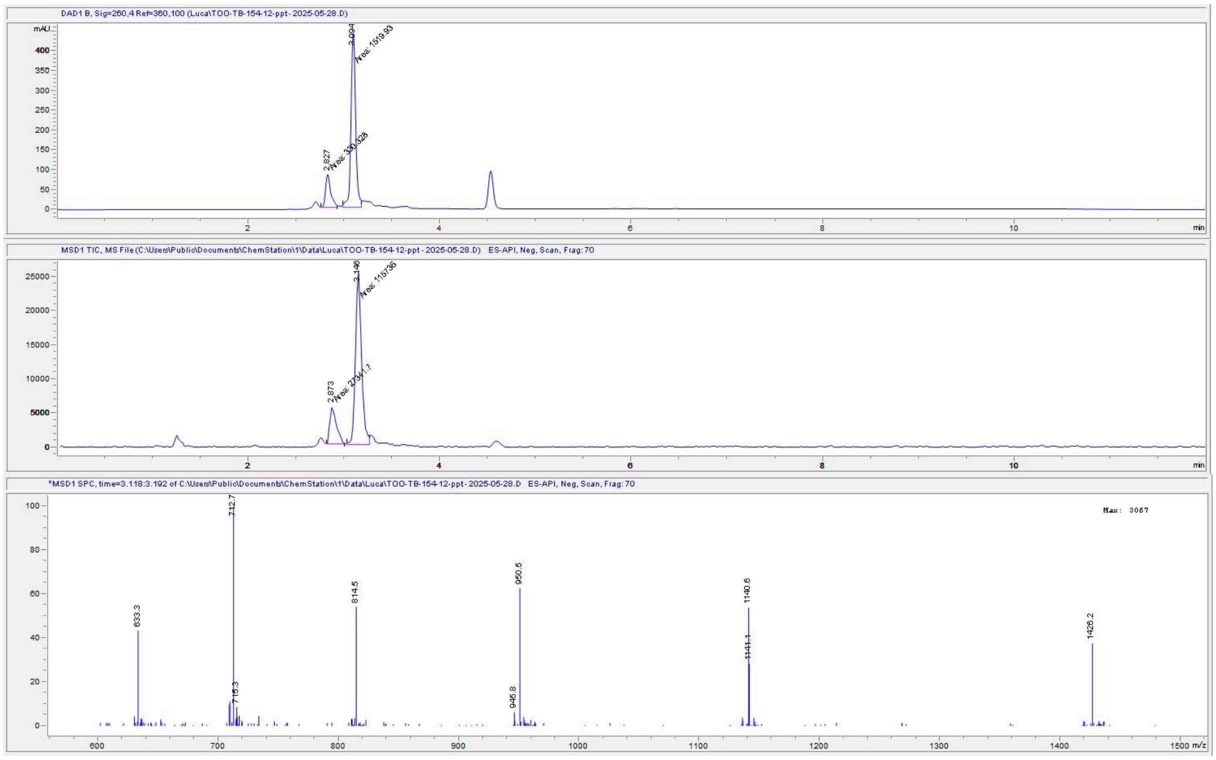

Figure S87. Analytical HPLC trace of **SRu-46** with HPLC method A. (Top) DAD chromatogram at 260 nm. (Middle) TIC chromatogram. (Bottom) Ionization of peak at 3.15 min. containing reaction product.

Decomplexation of SRu-46 to obtain product 46: Under an ambient atmosphere, the DNA-conjugate **SRu-46** stock solution in water (c = 0.10 mM, 20 µL) was irradiated with a 390 nm (40 W) Kessil lamp for 2 hours, while maintaining the temperature at approximately 30 °C through cooling with a fan. To the reaction mixture was added the stock solution of NaCl in water (**SR-06**, 2.0 µL, c = 5.0 M, 10% volume of the total reaction volume), followed by cold ethanol (−20 °C, 66 µL) to precipitate the DNA conjugate **46**. The Eppendorf tube was placed in the freezer (−20 °C) for at least 1 hour, and then it was centrifuged at 4 °C and 11000 × g for at least 30 minutes. The supernatant was removed, the pellet dried under air and dissolved in Milli-Q water to obtain the purified DNA-conjugate **46**. Then, 1 µL of the above solution was diluted to 40 µL with water for LC–MS analysis. The yield of the DNA conjugate was calculated by measuring the integration of the peaks of the diode array detection (DAD) UV absorbance at 260 nm of the LC–MS trace, assuming complete DNA recovery and identical UV absorbance.

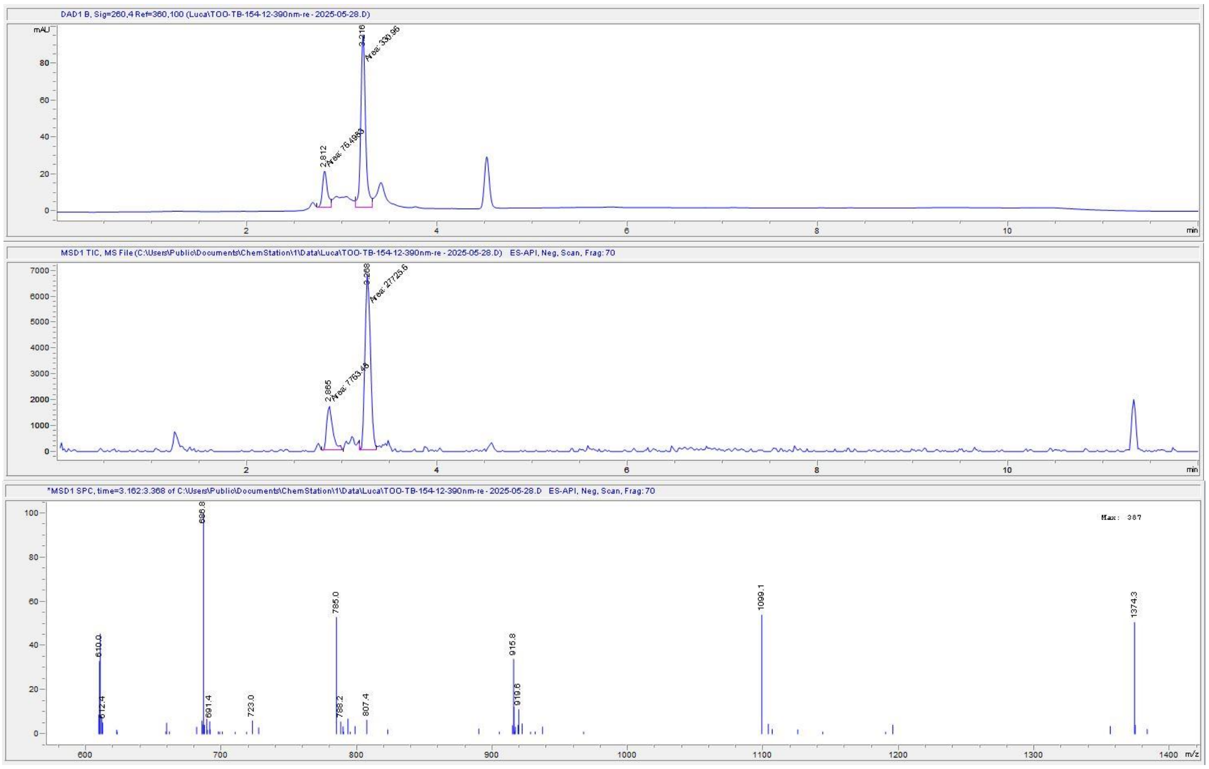

Figure S88. Analytical HPLC trace of **46** with HPLC method A. (Top) DAD chromatogram at 260 nm. (Middle) TIC chromatogram. (Bottom) Ionization of peak at 3.27 min. containing reaction product.

Synthesis of DNA-conjugate 47

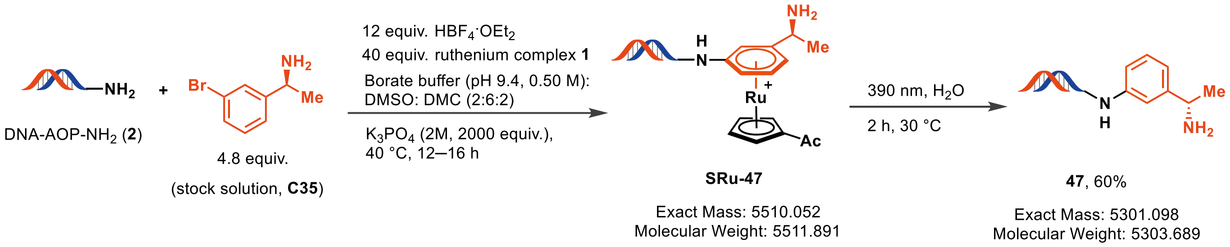

In situ formation of bromoarene-ruthenium stock solution: Under an ambient atmosphere, a 1 mL glass GC vial equipped with a 6 mm Teflon-coated stirring bar was charged with ruthenium complex **1** (1.0 mg, 2.1 µmol, 1.0 equiv.) and then 52 µL of DMC was added to the reaction vial. In a separate Eppendorf tube was added a stock solution of $HBF_4 \cdot OEt_2$ (25 µL, c = 1.0 M, 25 µmol, 12 equiv.), followed by a stock solution of (*S*)-1-(3-bromophenyl)ethan-1-amine in DMC (25 µL, c = 0.40 M, 10 µmol, 4.8 equiv.). The contents of the Eppendorf tube was then transferred to the reaction vial. The vial was then closed with a screw cap and resulting reaction mixture was heated at 80 °C for 2 hours. After 2 hours, the reaction mixture was cooled to 23 °C, resulting in an in situ formed stock solution of arene-ruthenium complex **C35** in DMC (105 µL, c= 0.02 M, assuming quantitative arene coordination to ruthenium).

Under an ambient atmosphere, a stock solution of DNA-AOP-NH_2 (**2**) (1.0 µL, c = 1.0 mM, 1.0 nmol, 1.0 equiv.) in sodium borate buffer (pH 9.5, c = 0.25 M) was added to a 1.5 mL Eppendorf tube, followed by a

stock solution of K_3PO_4 in water (1.0 µL, c = 2.0 M). To this mixture, 6 µL of DMSO was added and the solution was vortexed for 5 seconds. Next, the freshly prepared stock solution **C35** (2.0 µL, c = 0.02 M, 0.04 µmol, 40 equiv.) in DMC was then added. The resulting reaction mixture was vortexed for 5 seconds, transferred to a thermocycler at 40 °C, and incubated for 16 hours at 800 rpm to yield the DNA-conjugate **SRu-47**. Next, the reaction mixture was diluted with 10 µL of Milli-Q water. To the reaction mixture was added the stock solution of NaCl in water (**SR-06** , 2.0 µL, c = 5.0 M, 10% volume of the total reaction volume), followed by cold ethanol (−20 °C, 66 µL) to precipitate the *N*-arylated ruthenium DNA conjugate **SRu-47**. The Eppendorf tube was placed in a freezer (−20 °C) for at least 1 hour, and then it was centrifuged at 4 °C and 11000 × g for at least 30 minutes. The supernatant was removed and the pellet was dried under air, then dissolved in 20 µL water to obtain the DNA-conjugate **SRu-47** (10 µL, c = 0.10 mM). Then, 1.0 µL of the above solution was diluted to 40 µL with water for LC–MS analysis.

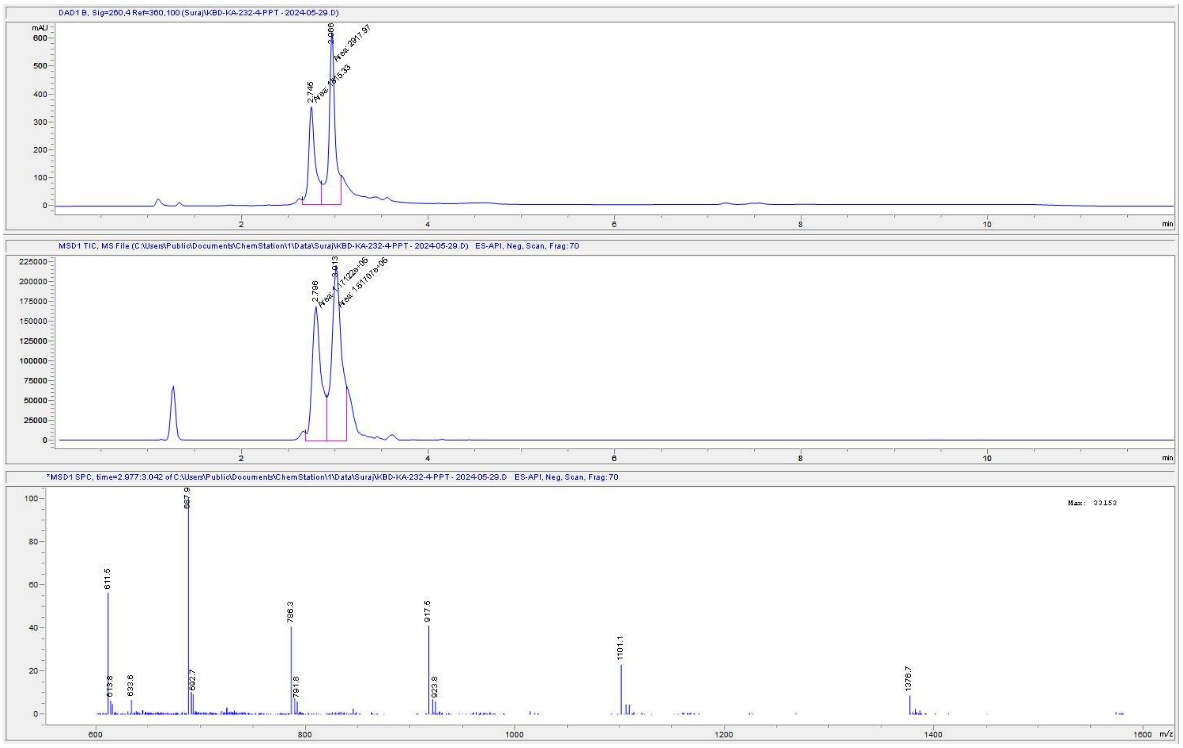

Figure S89. Analytical HPLC trace of **SRu-47** with HPLC method A. (Top) DAD chromatogram at 260 nm. (Middle) TIC chromatogram. (Bottom) Ionization of peak at 3.01 min containing reaction product.

Decomplexation of SRu-47 to obtain product 47: Under an ambient atmosphere, the DNA-conjugate **SRu-47** stock solution in water (c = 0.10 mM, 10 µL) was irradiated with a 390 nm (40 W) Kessil lamp for 2 hours, while maintaining the temperature at approximately 30 °C through cooling with a fan. To the reaction mixture was added the stock solution of NaCl in water (**SR-06**, 1.0 µL, c = 5.0 M, 10% volume of the total reaction volume), followed by cold ethanol (−20 °C, 33 µL) to precipitate the DNA conjugate **47**. The Eppendorf tube was placed in the freezer (−20 °C) for at least 1 hour, and then it was centrifuged at 4 °C and 11000 × g for at least 30 minutes. The supernatant was removed, the pellet dried under air and dissolved in Milli-Q water to

obtain the purified DNA-conjugate **47**. Then, 1 µL of the above solution was diluted to 40 µL with water for LC–MS analysis. The yield of the DNA conjugate was calculated by measuring the integration of the peaks of the diode array detection (DAD) UV absorbance at 260 nm of the LC–MS trace, assuming complete DNA recovery and identical UV absorbance.

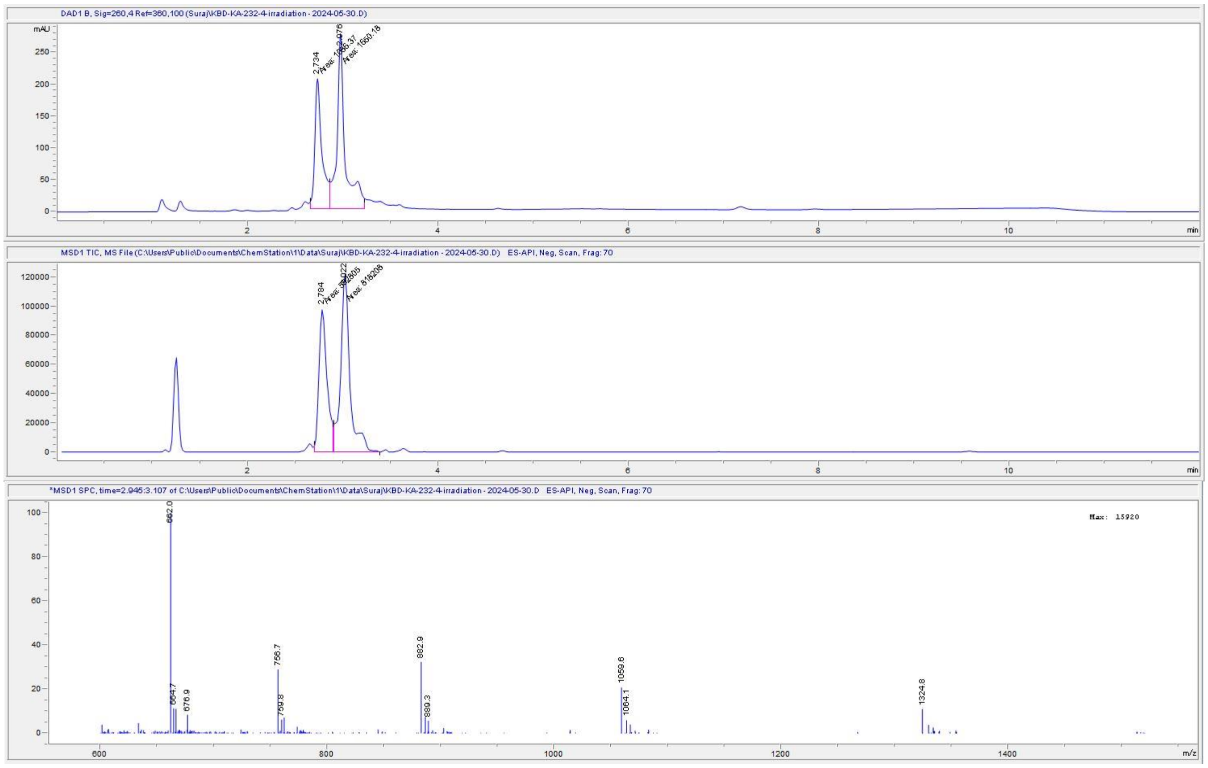

Figure S90. Analytical HPLC trace of **47** with HPLC method A. (Top) DAD chromatogram at 260 nm. (Middle) TIC chromatogram. (Bottom) Ionization of peak at 3.02 min. containing reaction product.

Synthesis of DNA-conjugate 48

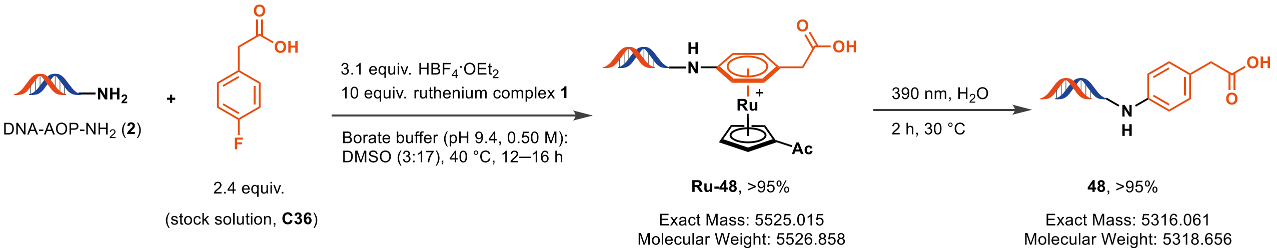

In situ formation of fluoroarene-ruthenium stock solution: Under an ambient atmosphere, a 1 mL glass GC vial equipped with a 6 mm Teflon-coated stirring bar was charged with ruthenium complex **1** (1.0 mg, 2.1 µmol, 1.0 equiv.) and then 100 µL of DMC was added to the reaction vial. In a separate Eppendorf tube was added a stock solution of $HBF_4{\cdot}OEt_2$ (50 µL, c = 0.13 M, 6.5 µmol, 3.1 equiv.), followed by a stock solution of 2-(4-fluorophenyl)acetic acid in DMC (50 µL, c = 0.10 M, 5.0 µmol, 2.4 equiv.). The contents of the Eppendorf tube was then transferred to the reaction vial. The vial was then closed with a screw cap and the resulting

reaction mixture was heated at 80 °C for 2 hours. After 2 hours, the reaction mixture was cooled to 23 °C and the DMC was removed under a gentle stream of argon and 200 µL of DMSO was added to result in an in situ formed stock solution of arene-ruthenium complex **C36** in DMSO (200 µL, c = 0.01 M, assuming quantitative arene coordination to ruthenium).

Under an ambient atmosphere, the stock solution **SD-01** of DNA-AOP-NH_2 (**2**) (1.0 µL, c = 2.0 mM, 2.0 nmol, 1.0 equiv.) in water was added to a 1.5 mL Eppendorf tube, followed by sodium borate buffer (2.0 µL, pH 9.4, c = 0.50 M). To this mixture, 15 µL of DMSO was added and the solution was vortexed for 5 seconds. Next, the freshly prepared stock solution **C39** (2.0 µL, c = 0.01 M, 0.02 µmol, 10 equiv.) in DMSO was added. The resulting reaction mixture was vortexed for 5 seconds, transferred to a thermocycler at 40 °C, and incubated for 16 hours at 800 rpm to yield the DNA-conjugate **SRu-48**. To the reaction mixture was added the stock solution of NaCl in water (**SR-06** , 2.0 µL, c = 5.0 M, 10% volume of the total reaction volume), followed by cold ethanol (−20 °C, 66 µL) to precipitate the *N*-arylated ruthenium DNA conjugate **SRu-48**. The Eppendorf tube was placed in a freezer (−20 °C) for at least 1 hour, and then it was centrifuged at 4 °C and 11000 × g for at least 30 minutes. The supernatant was removed and the pellet was dried under air, then dissolved in 20 µL water to obtain the DNA-conjugate **SRu-48** (10 µL, c = 0.10 mM). Then, 1.0 µL of the above solution was diluted to 40 µL with water for LC–MS analysis.

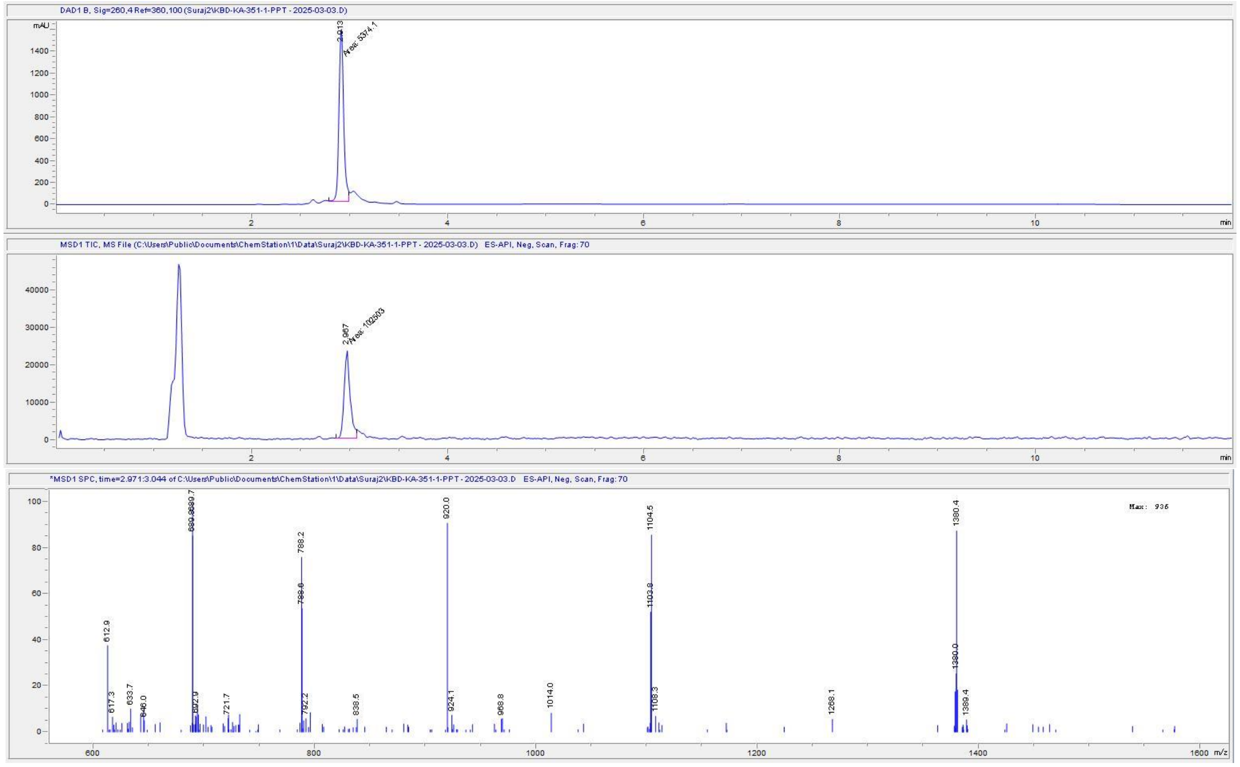

Figure S91. Analytical HPLC trace of **SRu-48** with HPLC method A. (Top) DAD chromatogram at 260 nm. (Middle) TIC chromatogram. (Bottom) Ionization of peak at 2.97 min. containing reaction product.

Decomplexation of SRu-48 to obtain product 48: Under an ambient atmosphere, the DNA-conjugate **SRu-48** stock solution in water (c = 0.10 mM, 20 µL) was irradiated with a 390 nm (40 W) Kessil lamp for 2 hours,

while maintaining the temperature at approximately 30 °C through cooling with a fan. To the reaction mixture was added the stock solution of NaCl in water (**SR-06**, 2.0 µL, c = 5.0 M, 10% volume of the total reaction volume), followed by cold ethanol (−20 °C, 66 µL) to precipitate the DNA conjugate **48**. The Eppendorf tube was placed in the freezer (−20 °C) for at least 1 hour, and then it was centrifuged at 4 °C and 11000 × g for at least 30 minutes. The supernatant was removed, the pellet dried under air and dissolved in Milli-Q water to obtain the purified DNA-conjugate **48**. Then, 1 µL of the above solution was diluted to 40 µL with water for LC–MS analysis. The yield of the DNA conjugate was calculated by measuring the integration of the peaks of the diode array detection (DAD) UV absorbance at 260 nm of the LC–MS trace, assuming complete DNA recovery and identical UV absorbance.

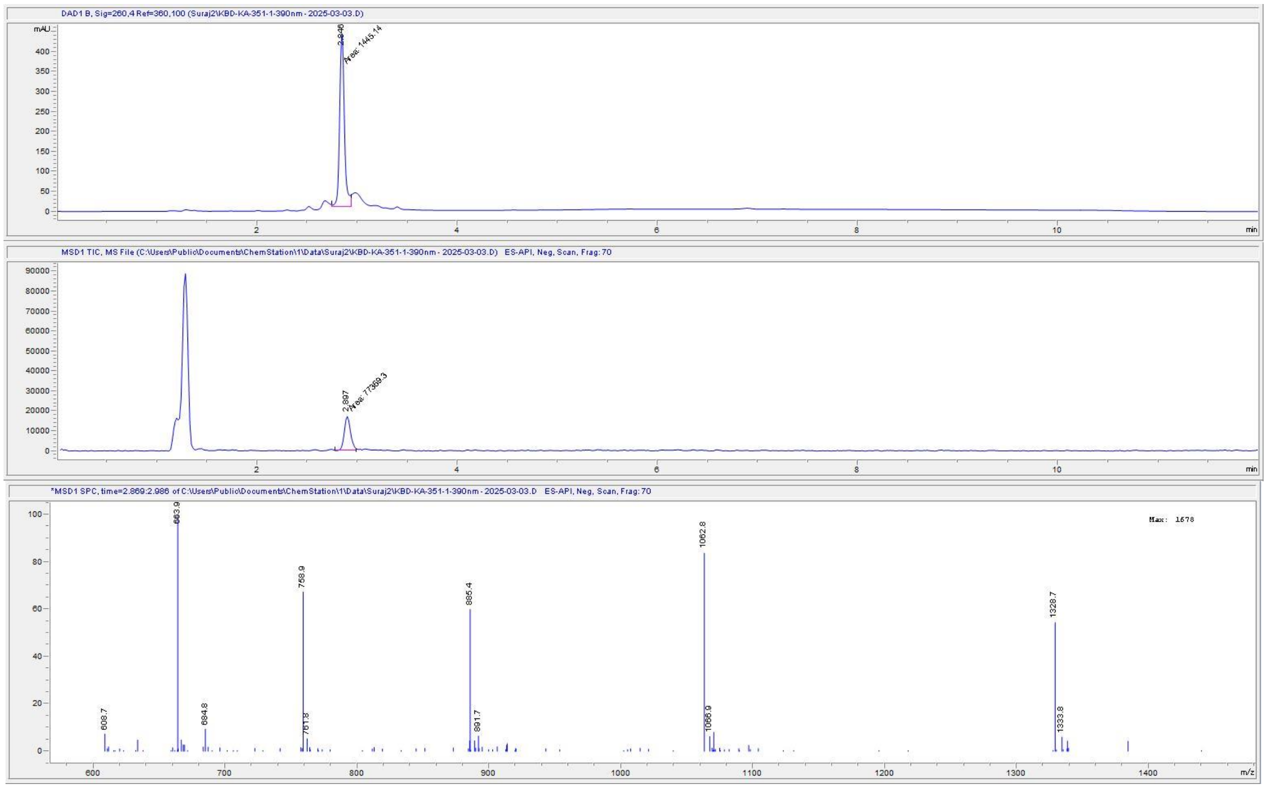

Figure S92. Analytical HPLC trace of **48** with HPLC method A. (Top) DAD chromatogram at 260 nm. (Middle) TIC chromatogram. (Bottom) Ionization of peak at 2.90 min. containing reaction product.

Synthesis of DNA-conjugate 49

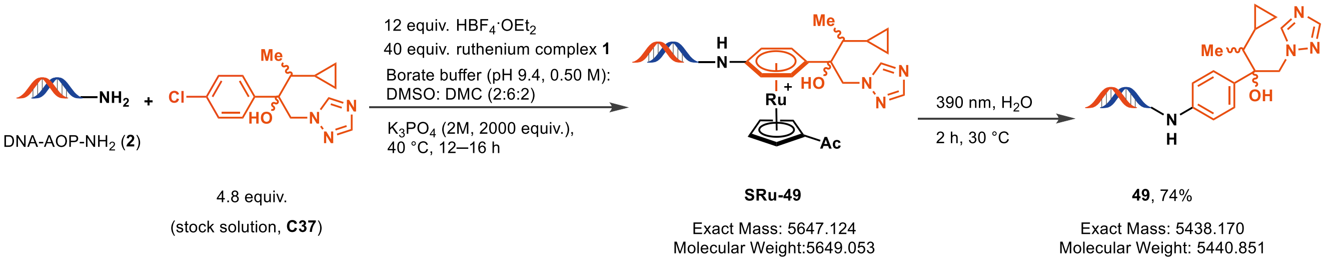

In situ formation of chloroarene-ruthenium stock solution: Under an ambient atmosphere, a 1 mL glass

GC vial equipped with a 6 mm Teflon-coated stirring bar was charged with ruthenium complex **1** (1.0 mg, 2.1 µmol, 1.0 equiv.) and then 52 µL of DMC was added to the reaction vial. In a separate Eppendorf tube was added a stock solution of $HBF_4{\cdot}OEt_2$ (25 µL, c = 1.0 M, 25 µmol, 12 equiv.), followed by a stock solution of Cyproconazole in DMC (25 µL, c= 0.40 M, 10 µmol, 4.8 equiv.). The contents of the Eppendorf tube was then transferred to the reaction vial. The vial was then closed with a screw cap and the resulting reaction mixture was heated at 80 °C for 2 hours. After 2 hours, the reaction mixture was cooled to 23 °C, resulting in an in situ formed stock solution of arene-ruthenium complex **C37** in DMC (105 µL, c = 0.02 M, assuming quantitative arene coordination to ruthenium).

Under an ambient atmosphere, a stock solution of DNA-AOP-NH_2 (**2**) (1.0 µL, c = 1.0 mM, 1.0 nmol, 1.0 equiv.) in sodium borate buffer was added to a 1.5 mL Eppendorf tube, followed by a stock solution of K_3PO_4 in water (1.0 µL, c = 2.0 M). To this mixture, 6 µL of DMSO was added and the solution was vortexed for 5 seconds. Next, the freshly prepared stock solution **C37** (2.0 µL, c = 0.02 M, 0.04 µmol, 40 equiv.) in DMC was then added. The resulting reaction mixture was vortexed for 5 seconds, transferred to a thermocycler at 40 °C, and incubated for 16 hours at 800 rpm to yield the DNA-conjugate **SRu-49**. Next, the reaction mixture was diluted with 10 µL of Milli-Q water. To the reaction mixture was added the stock solution of NaCl in water (**SR-06** , 2.0 µL, c = 5.0 M, 10% volume of the total reaction volume), followed by cold ethanol (−20 °C, 66 µL) to precipitate the *N*-arylated ruthenium DNA conjugate **SRu-49**. The Eppendorf tube was placed in a freezer (−20 °C) for at least 1 hour, and then it was centrifuged at 4 °C and 11000 × g for at least 30 minutes. The supernatant was removed and the pellet was dried under air, then dissolved in 20 µL water to obtain the DNA-conjugate **SRu-49** (10 µL, c = 0.10 mM). Then, 1.0 µL of the above solution was diluted to 40 µL with water for LC–MS analysis.

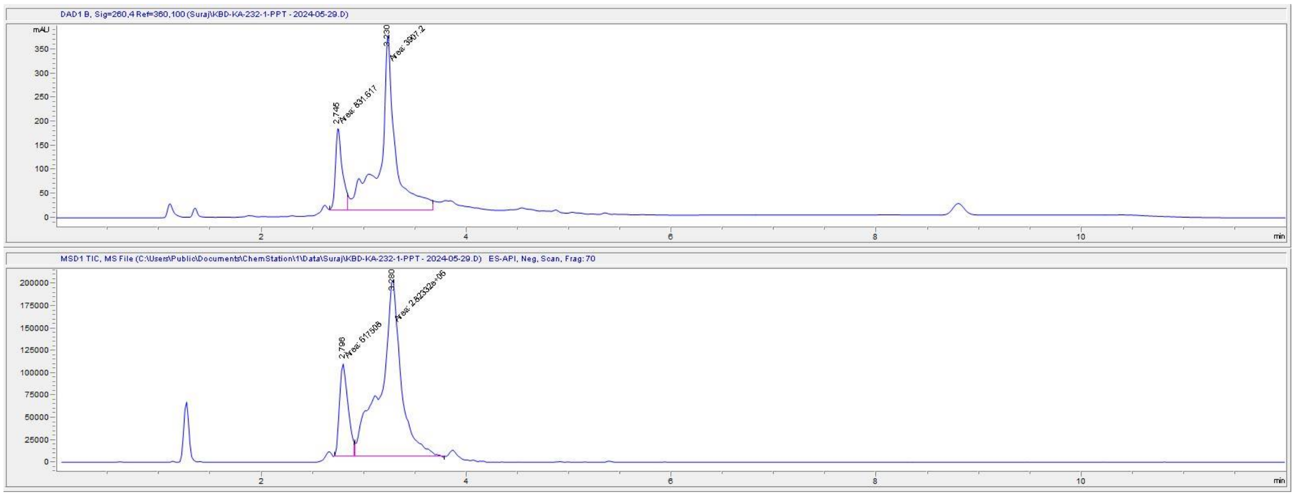

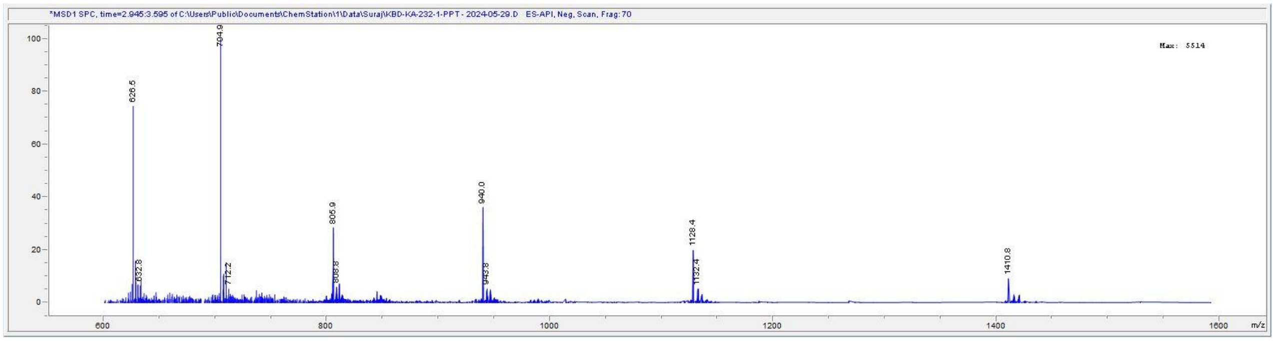

Figure S93. Analytical HPLC trace of **SRu-49** with HPLC method A. (Top) DAD chromatogram at 260 nm. (Middle) TIC chromatogram. (Bottom) Ionization of peak at 3.00–3.75 min. containing reaction product.

Decomplexation of SRu-49 to obtain product 49: Under an ambient atmosphere, the DNA-conjugate **SRu-49** stock solution in water (c = 0.10 mM, 10 µL) was irradiated with a 390 nm (40 W) Kessil lamp for 2 hours, while maintaining the temperature at approximately 30 °C through cooling with a fan. To the reaction mixture was added the stock solution of NaCl in water (**SR-06**, 2.0 µL, c = 5.0 M, 10% volume of the total reaction volume), followed by cold ethanol (−20 °C, 66 µL) to precipitate the DNA conjugate **49**. The Eppendorf tube was placed in the freezer (−20 °C) for at least 1 hour, and then it was centrifuged at 4 °C and 11000 × g for at least 30 minutes. The supernatant was removed, the pellet dried under air and dissolved in Milli-Q water to obtain the purified DNA-conjugate **49**. Then, 1 µL of the above solution was diluted to 40 µL with water for LC–MS analysis. The yield of the DNA conjugate was calculated by measuring the integration of the peaks of the diode array detection (DAD) UV absorbance at 260 nm of the LC–MS trace, assuming complete DNA recovery and identical UV absorbance.

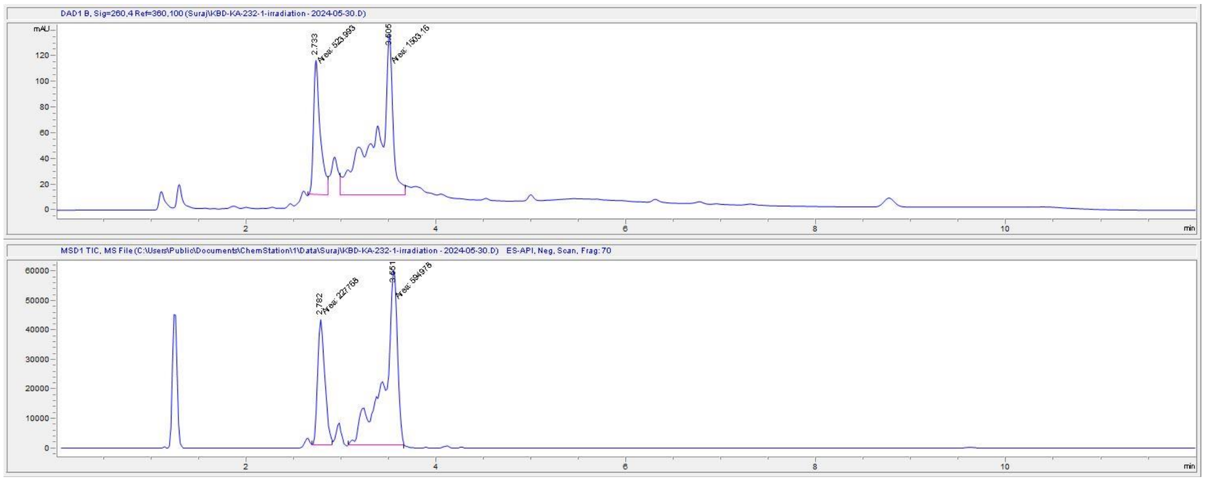

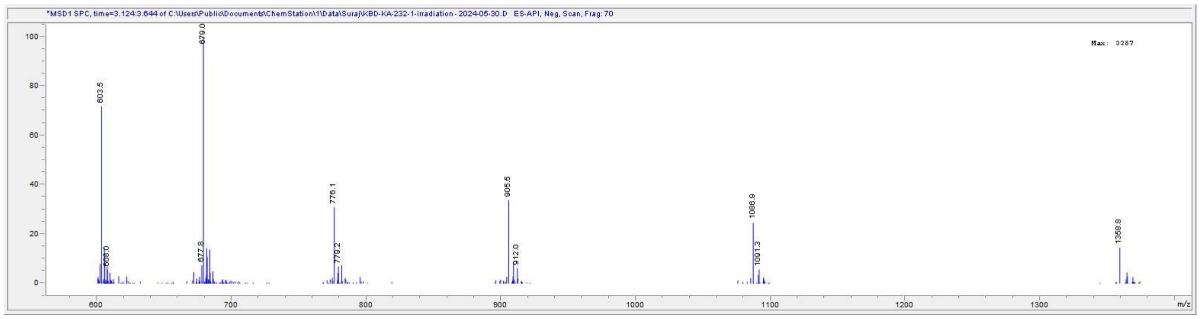

Figure S94. Analytical HPLC trace of **49** with HPLC method A. (Top) DAD chromatogram at 260 nm. (Middle) TIC chromatogram. (Bottom) Ionization of peak at 3.10–3.75 min. containing reaction product.

Synthesis of DNA-conjugate 50

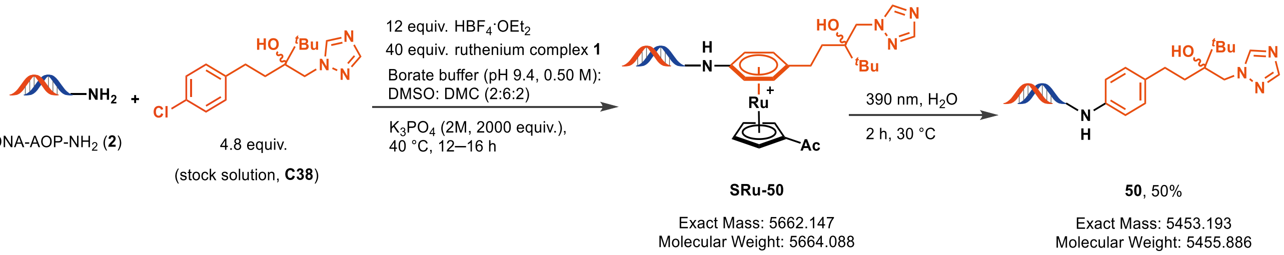

In situ formation of bromoarene-ruthenium stock solution: Under an ambient atmosphere, a 1 mL glass GC vial equipped with a 6 mm Teflon-coated stirring bar was charged with ruthenium complex **1** (1.0 mg, 2.1 µmol, 1.0 equiv.) and then 52 µL of DMC was added to the reaction vial. In a separate Eppendorf tube was added a stock solution of $HBF_4 \cdot OEt_2$ (25 µL, c = 1.0 M, 25 µmol, 12 equiv.), followed by a stock solution of Tebuconazole in DMC (25 µL, c= 0.40 M, 10 µmol, 4.8 equiv.). The contents of the Eppendorf tube was then transferred to the reaction vial. The vial was then closed with a screw cap and resulting reaction mixture was heated at 80 °C for 2 hours. After 2 hours, the reaction mixture was cooled to 23 °C, resulting in an in situ formed stock solution of arene-ruthenium complex **C38** in DMC (105 µL, c= 0.02 M, assuming quantitative arene coordination to ruthenium).

Under an ambient atmosphere, a stock solution of DNA-AOP-NH_2 (**2**) (1.0 µL, c = 1.0 mM, 1.0 nmol, 1.0 equiv.) in sodium borate buffer was added to a 1.5 mL Eppendorf tube, followed by a stock solution of K_3PO_4 in water (1.0 µL, c = 2.0 M). To this mixture, 6 µL of DMSO was added and the solution was vortexed for 5 seconds. Next, the freshly prepared stock solution **C38** (2.0 µL, c = 0.02 M, 0.04 µmol, 40 equiv.) in DMC was then added. The resulting reaction mixture was vortexed for 5 seconds, transferred to a thermocycler at 40 °C, and incubated for 16 hours at 800 rpm to yield the DNA-conjugate **SRu-50**. Next, the reaction mixture was diluted with 10 µL of Milli-Q water. To the reaction mixture was added the stock solution of NaCl in water (**SR-06** , 2.0 µL, c = 5.0 M, 10% volume of the total reaction volume), followed by cold ethanol (−20 °C, 66 µL) to precipitate the *N*-arylated ruthenium DNA conjugate **SRu-50**. The Eppendorf tube was placed in a freezer (−20 °C) for at least 1 hour, and then it was centrifuged at 4 °C and 11000 × g for at least 30 minutes. The supernatant was removed and the pellet was dried under air, then dissolved in 20 µL water to obtain the

DNA-conjugate **SRu-50** (10 µL, c = 0.10 mM). Then, 1.0 µL of the above solution was diluted to 40 µL with water for LC–MS analysis.

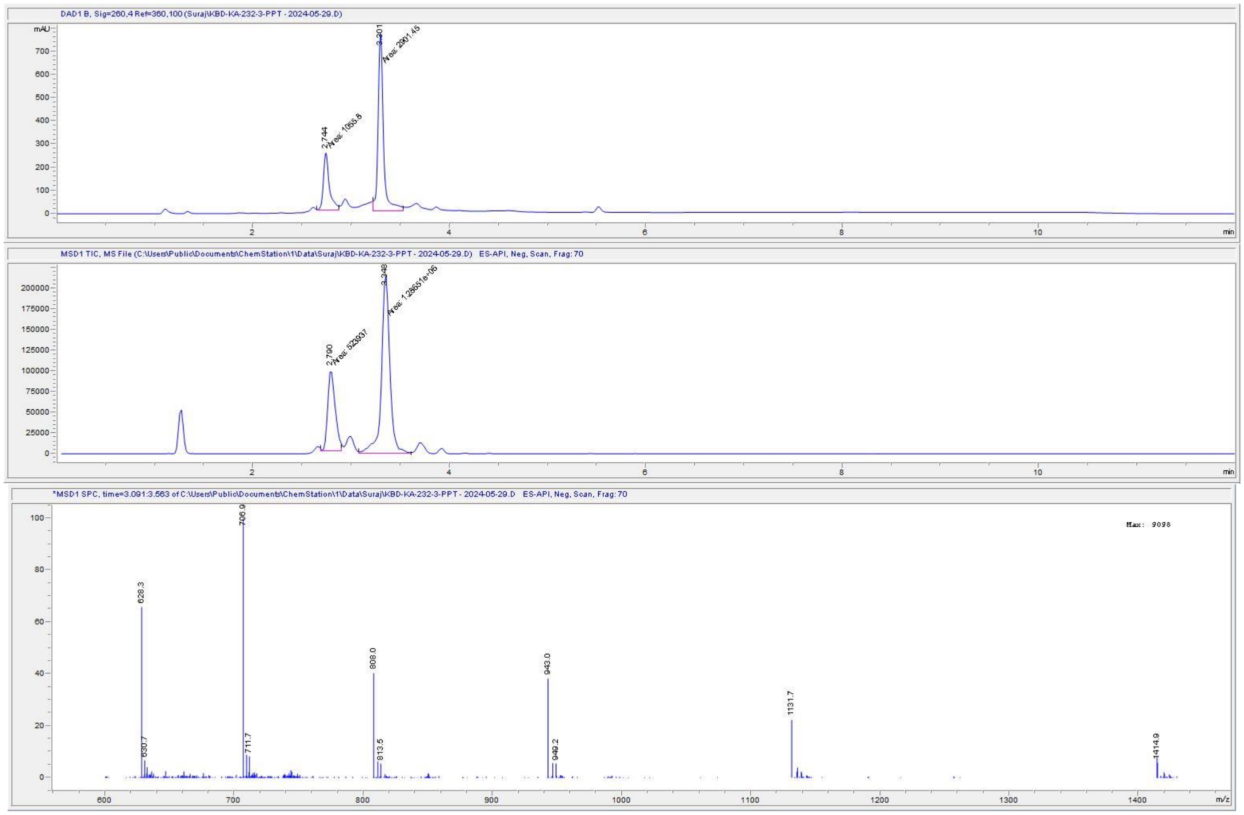

Figure S95. Analytical HPLC trace of **SRu-50** with HPLC method A. (Top) DAD chromatogram at 260 nm. (Middle) TIC chromatogram. (Bottom) Ionization of peak at 3.35 min. containing reaction product.

Decomplexation of SRu-50 to obtain product 50: Under an ambient atmosphere, the DNA-conjugate **SRu-50** stock solution in water (c = 0.10 mM, 10 µL) was irradiated with a 390 nm (40 W) Kessil lamp for 2 hours, while maintaining the temperature at approximately 30 °C through cooling with a fan. To the reaction mixture was added the stock solution of NaCl in water (**SR-06**, 2.0 µL, c = 5.0 M, 10% volume of the total reaction volume), followed by cold ethanol (−20 °C, 66 µL) to precipitate the DNA conjugate **50**. The Eppendorf tube was placed in the freezer (−20 °C) for at least 1 hour, and then it was centrifuged at 4 °C and 11000 × g for at least 30 minutes. The supernatant was removed, the pellet dried under air and dissolved in Milli-Q water to obtain the purified DNA-conjugate **50**. Then, 1 µL of the above solution was diluted to 40 µL with water for LC–MS analysis. The yield of the DNA conjugate was calculated by measuring the integration of the peaks of the diode array detection (DAD) UV absorbance at 260 nm of the LC–MS trace, assuming complete DNA recovery and identical UV absorbance.

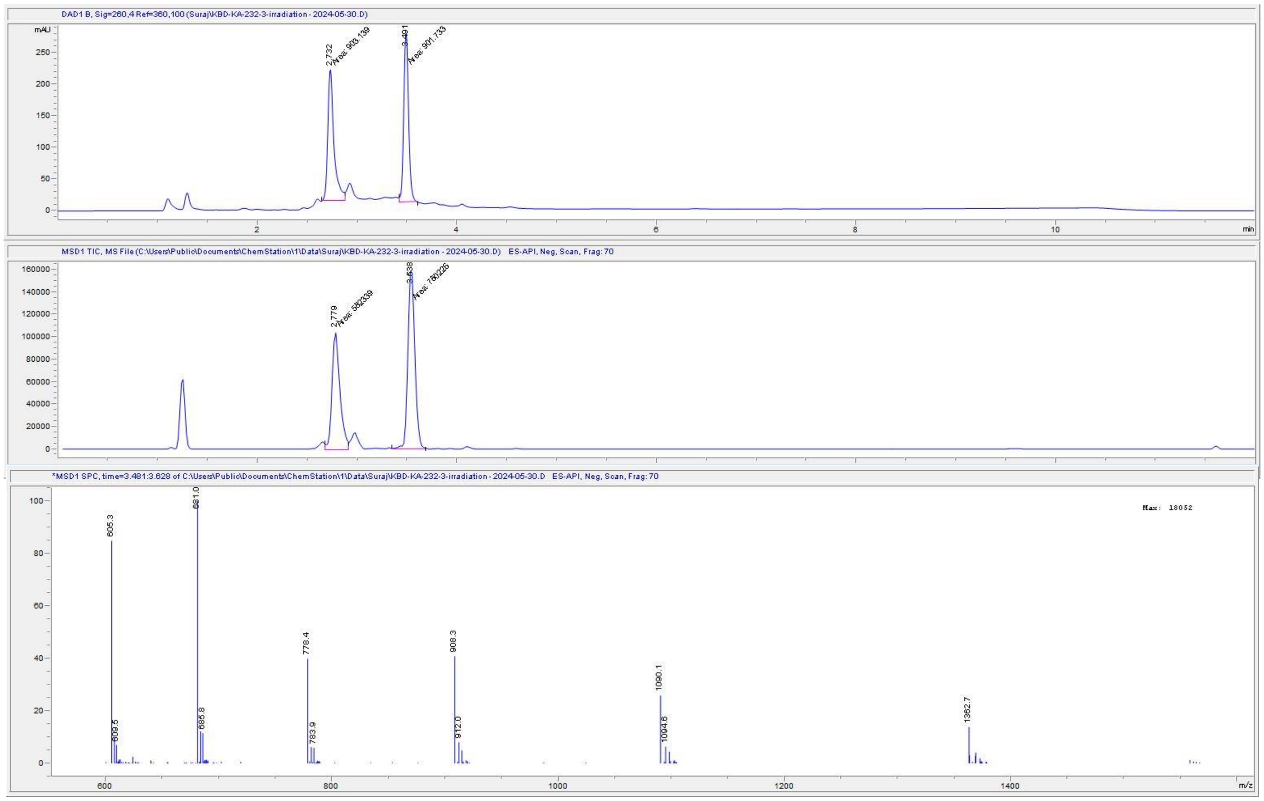

Figure S96. Analytical HPLC trace of **50** with HPLC method A. (Top) DAD chromatogram at 260 nm. (Middle) TIC chromatogram. (Bottom) Ionization of peak at 3.54 min. containing reaction product.

Synthesis of DNA-conjugate 51

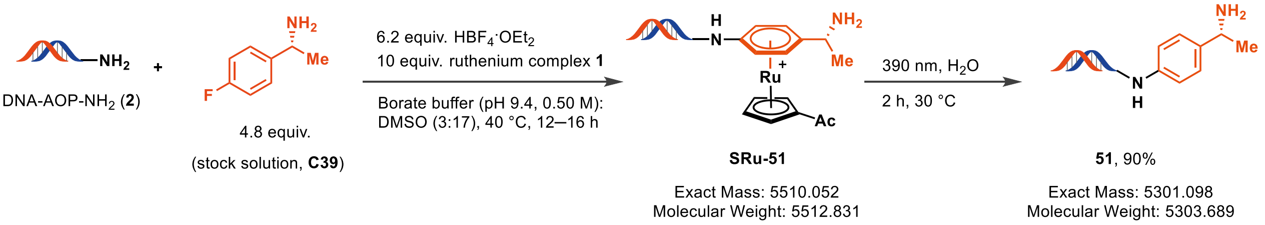

In situ formation of fluoroarene-ruthenium stock solution: Under an ambient atmosphere, a 1 mL glass GC vial equipped with a 6 mm Teflon-coated stirring bar was charged with ruthenium complex **1** (1.0 mg, 2.1 µmol, 1.0 equiv.) and then 100 µL of DMC was added to the reaction vial. In a separate Eppendorf tube was added a stock solution of $HBF_4{\cdot}OEt_2$ (50 µL, c = 0.26 M, 13 µmol, 6.2 equiv.), followed by a stock solution of (*R*)-1-(4-fluorophenyl)ethan-1-amine in DMC (50 µL, c = 0.20 M, 10 µmol, 4.8 equiv.). The contents of the Eppendorf tube was then transferred to the reaction vial. The vial was then closed with a screw cap and the resulting reaction mixture was heated at 80 °C for 2 hours. After 2 hours, the reaction mixture was cooled to 23 °C and the DMC was removed under a gentle stream of argon and 200 µL of DMSO was added to result in an in situ formed stock solution of arene-ruthenium complex **C39** in DMSO (200 µL, c = 0.01 M, assuming quantitative arene coordination to ruthenium).

Under an ambient atmosphere, the stock solution **SD-01** of DNA-AOP-NH_2 (**2**) (1.0 µL, c = 2.0 mM, 2.0 nmol, 1.0 equiv.) in water was added to a 1.5 mL Eppendorf tube, followed by sodium borate buffer (2.0 µL, pH 9.4, c = 0.50 M). To this mixture, 15 µL of DMSO was added and the solution was vortexed for 5 seconds. Next, the freshly prepared stock solution **C39** (2.0 µL, c = 0.01 M, 0.02 µmol, 10 equiv.) in DMSO was added. The resulting reaction mixture was vortexed for 5 seconds, transferred to a thermocycler at 40 °C, and incubated for 16 hours at 800 rpm to yield the DNA-conjugate **SRu-51**. To the reaction mixture was added the stock solution of NaCl in water (**SR-06** , 2.0 µL, c = 5.0 M, 10% volume of the total reaction volume), followed by cold ethanol (−20 °C, 66 µL) to precipitate the *N*-arylated ruthenium DNA conjugate **SRu-51**. The Eppendorf tube was placed in a freezer (−20 °C) for at least 1 hour, and then it was centrifuged at 4 °C and 11000 × g for at least 30 minutes. The supernatant was removed and the pellet was dried under air, then dissolved in 20 µL water to obtain the DNA-conjugate **SRu-51** (10 µL, c = 0.10 mM). Then, 1.0 µL of the above solution was diluted to 40 µL with water for LC–MS analysis.

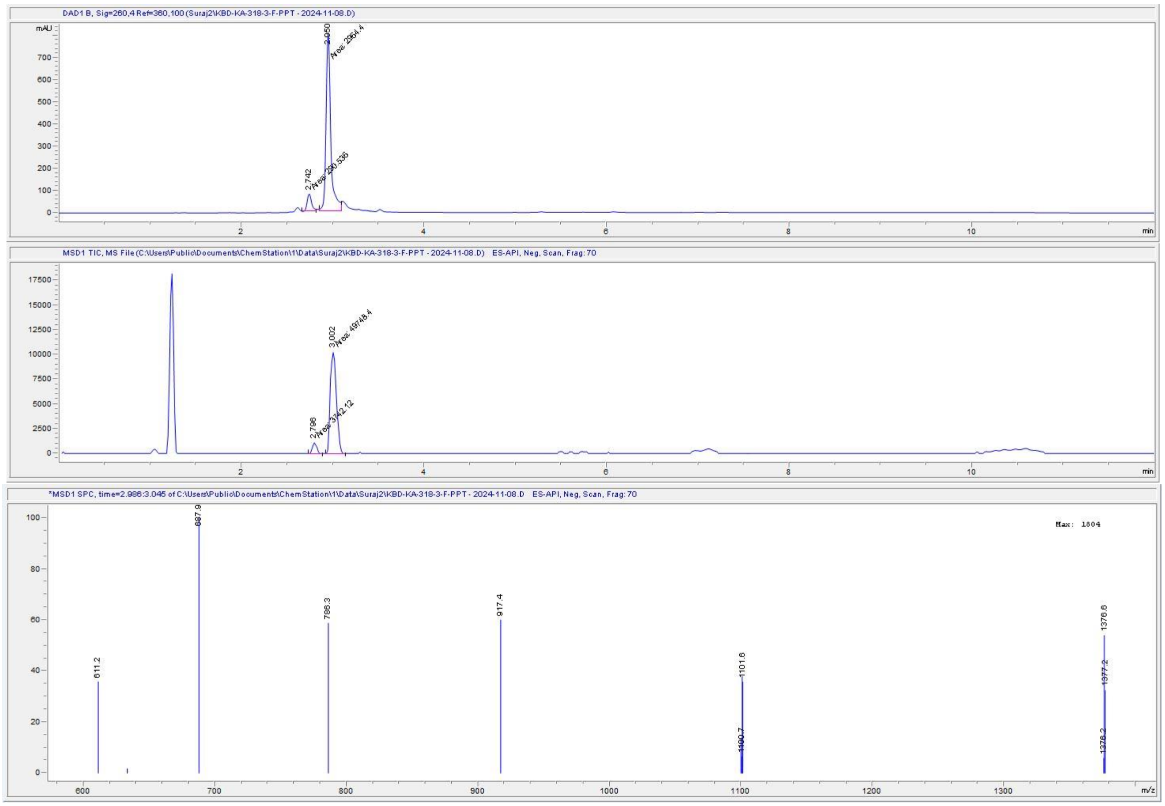

Figure S97. Analytical HPLC trace of **SRu-51** with HPLC method A. (Top) DAD chromatogram at 260 nm. (Middle) TIC chromatogram. (Bottom) Ionization of peak at 3.00 min. containing reaction product.

Decomplexation of SRu-51 to obtain product 51: Under an ambient atmosphere, the DNA-conjugate **SRu-51** stock solution in water (c = 0.10 mM, 20 µL) was irradiated with a 390 nm (40 W) Kessil lamp for 2 hours, while maintaining the temperature at approximately 30 °C through cooling with a fan. To the reaction mixture was added the stock solution of NaCl in water (**SR-06**, 2.0 µL, c = 5.0 M, 10% volume of the total reaction volume), followed by cold ethanol (−20 °C, 66 µL) to precipitate the DNA conjugate **51**. The Eppendorf tube

was placed in the freezer (−20 °C) for at least 1 hour, and then it was centrifuged at 4 °C and 11000 × g for at least 30 minutes. The supernatant was removed, the pellet dried under air and dissolved in Milli-Q water to obtain the purified DNA-conjugate **51**. Then, 1 µL of the above solution was diluted to 40 µL with water for LC–MS analysis. The yield of the DNA conjugate was calculated by measuring the integration of the peaks of the diode array detection (DAD) UV absorbance at 260 nm of the LC–MS trace, assuming complete DNA recovery and identical UV absorbance.

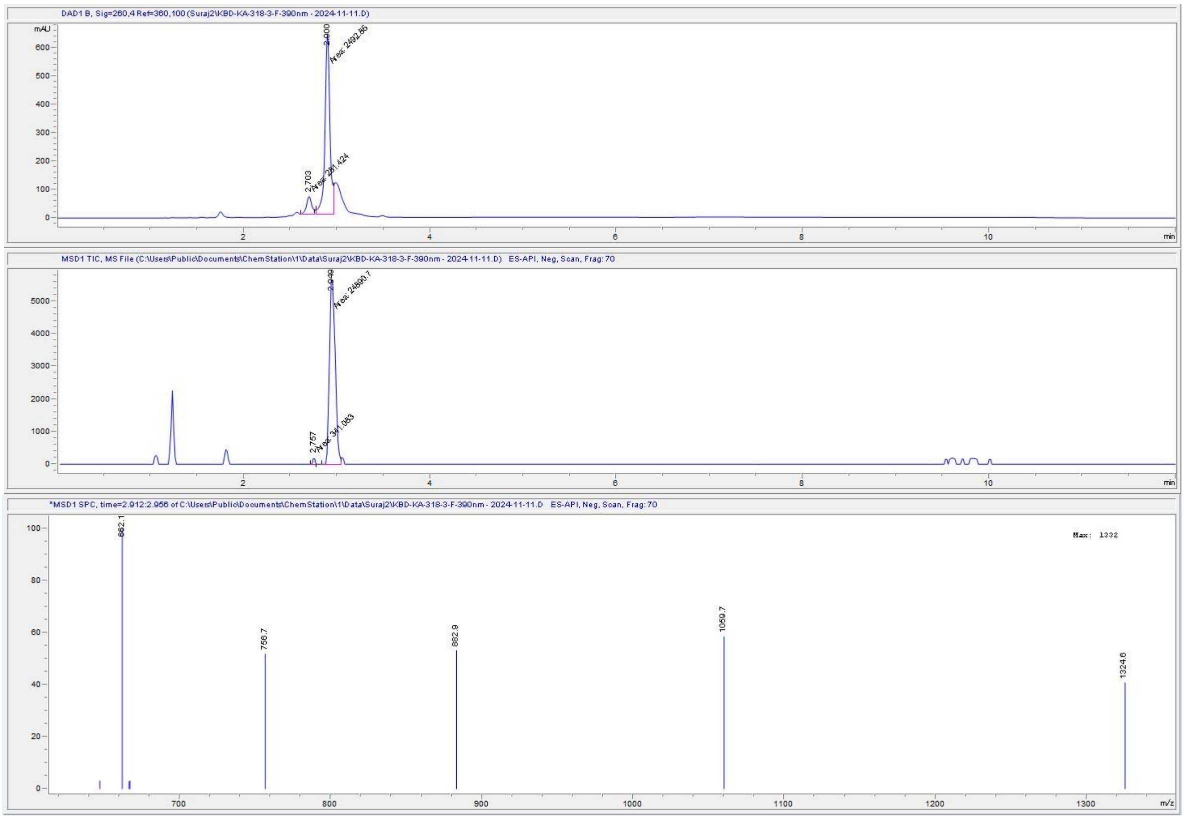

Figure S98. Analytical HPLC trace of **51** with HPLC method A. (Top) DAD chromatogram at 260 nm. (Middle) TIC chromatogram. (Bottom) Ionization of peak at 2.95 min. containing reaction product.

Synthesis of DNA-conjugate 52

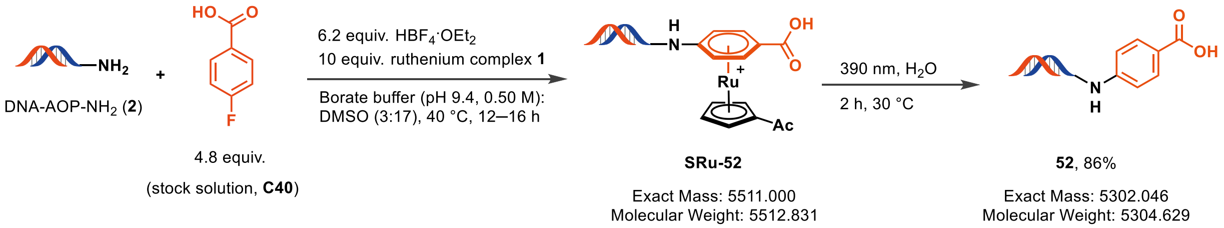

In situ formation of arene-ruthenium stock solution: Under an ambient atmosphere, a 1 mL glass GC vial equipped with a 6 mm Teflon-coated stirring bar was charged with ruthenium complex **1** (1.0 mg, 2.1 µmol, 1.0 equiv.) and then 100 µL of DMC was added to the reaction vial. In a separate Eppendorf tube was added a stock solution of $HBF_4 \cdot OEt_2$ (50 µL, c = 0.26 M, 13 µmol, 6.2 equiv.), followed by a stock solution of 4-

fluorobenzoic acid in DMC (50 µL, c = 0.20 M, 10 µmol, 4.8 equiv.). The contents of the Eppendorf tube was then transferred to the reaction vial. The vial was then closed with a screw cap and the resulting reaction mixture was heated at 80 °C for 2 hours. After 2 hours, the reaction mixture was cooled to 23 °C and the DMC was removed under a gentle stream of argon and 200 µL of DMSO was added to result in an in situ formed stock solution of arene-ruthenium complex **C40** in DMSO (200 µL, c = 0.01 M, assuming quantitative arene coordination to ruthenium).

Under an ambient atmosphere, the stock solution **SD-01** of DNA-AOP-NH_2 (**2**) (1.0 µL, c = 2.0 mM, 2.0 nmol, 1.0 equiv.) in water was added to a 1.5 mL Eppendorf tube, followed by sodium borate buffer (2.0 µL, pH 9.4, c = 0.50 M). To this mixture, 15 µL of DMSO was added and the solution was vortexed for 5 seconds. Next, the freshly prepared stock solution **C40** (2.0 µL, c = 0.01 M, 0.02 µmol, 10 equiv.) in DMSO was added. The resulting reaction mixture was vortexed for 5 seconds, transferred to a thermocycler at 40 °C, and incubated for 16 hours at 800 rpm to yield the DNA-conjugate **SRu-52**. To the reaction mixture was added the stock solution of NaCl in water (**SR-06** , 2.0 µL, c = 5.0 M, 10% volume of the total reaction volume), followed by cold ethanol (−20 °C, 66 µL) to precipitate the *N*-arylated ruthenium DNA conjugate **SRu-52**. The Eppendorf tube was placed in a freezer (−20 °C) for at least 1 hour, and then it was centrifuged at 4 °C and 11000 × g for at least 30 minutes. The supernatant was removed and the pellet was dried under air, then dissolved in 20 µL water to obtain the DNA-conjugate **SRu-52** (10 µL, c = 0.10 mM). Then, 1.0 µL of the above solution was diluted to 40 µL with water for LC–MS analysis.

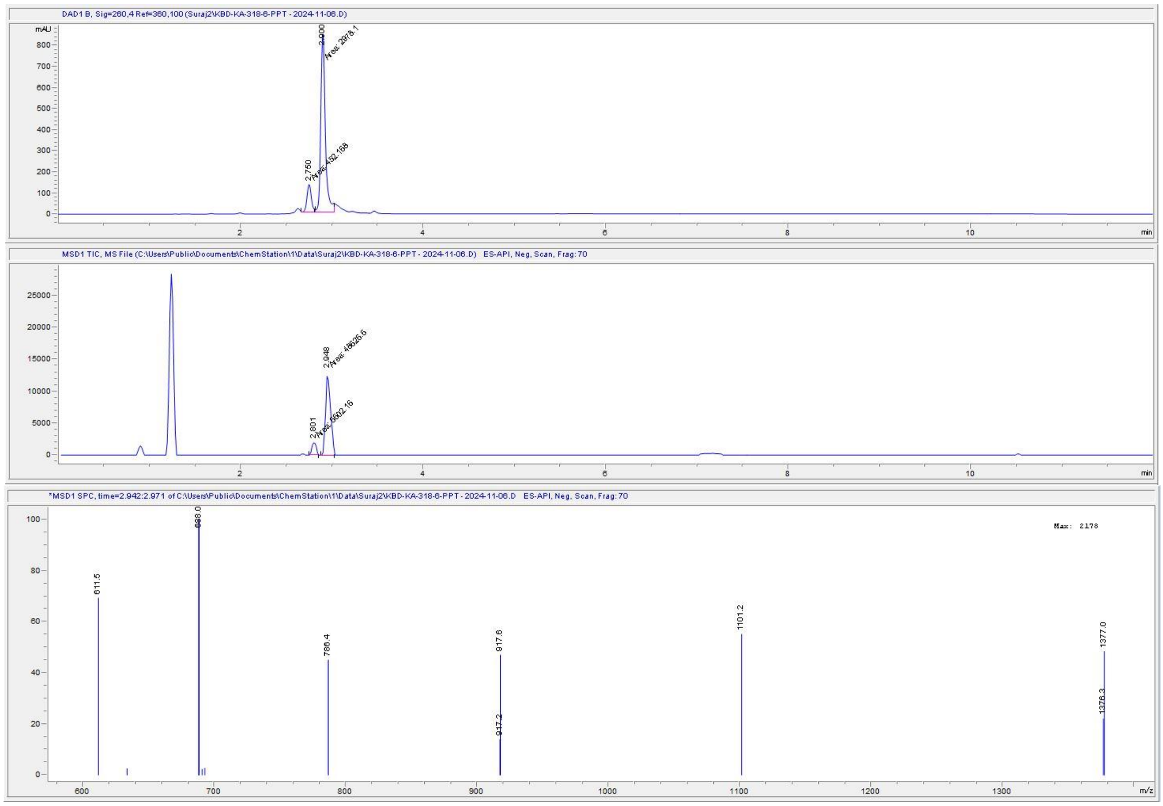

Figure S99. Analytical HPLC trace of **SRu-52** with HPLC method A. (Top) DAD chromatogram at 260 nm.

(Middle) TIC chromatogram. (Bottom) Ionization of peak at 2.95 min. containing reaction product.

Decomplexation of SRu-52 to obtain product 52: Under an ambient atmosphere, the DNA-conjugate **SRu-52** stock solution in water (c = 0.10 mM, 20 µL) was irradiated with a 390 nm (40 W) Kessil lamp for 2 hours, while maintaining the temperature at approximately 30 °C through cooling with a fan. To the reaction mixture was added the stock solution of NaCl in water (**SR-06**, 2.0 µL, c = 5.0 M, 10% volume of the total reaction volume), followed by cold ethanol (−20 °C, 66 µL) to precipitate the DNA conjugate **52**. The Eppendorf tube was placed in the freezer (−20 °C) for at least 1 hour, and then it was centrifuged at 4 °C and 11000 × g for at least 30 minutes. The supernatant was removed, the pellet dried under air and dissolved in Milli-Q water to obtain the purified DNA-conjugate **52**. Then, 1 µL of the above solution was diluted to 40 µL with water for LC–MS analysis. The yield of the DNA conjugate was calculated by measuring the integration of the peaks of the diode array detection (DAD) UV absorbance at 260 nm of the LC–MS trace, assuming complete DNA recovery and identical UV absorbance.

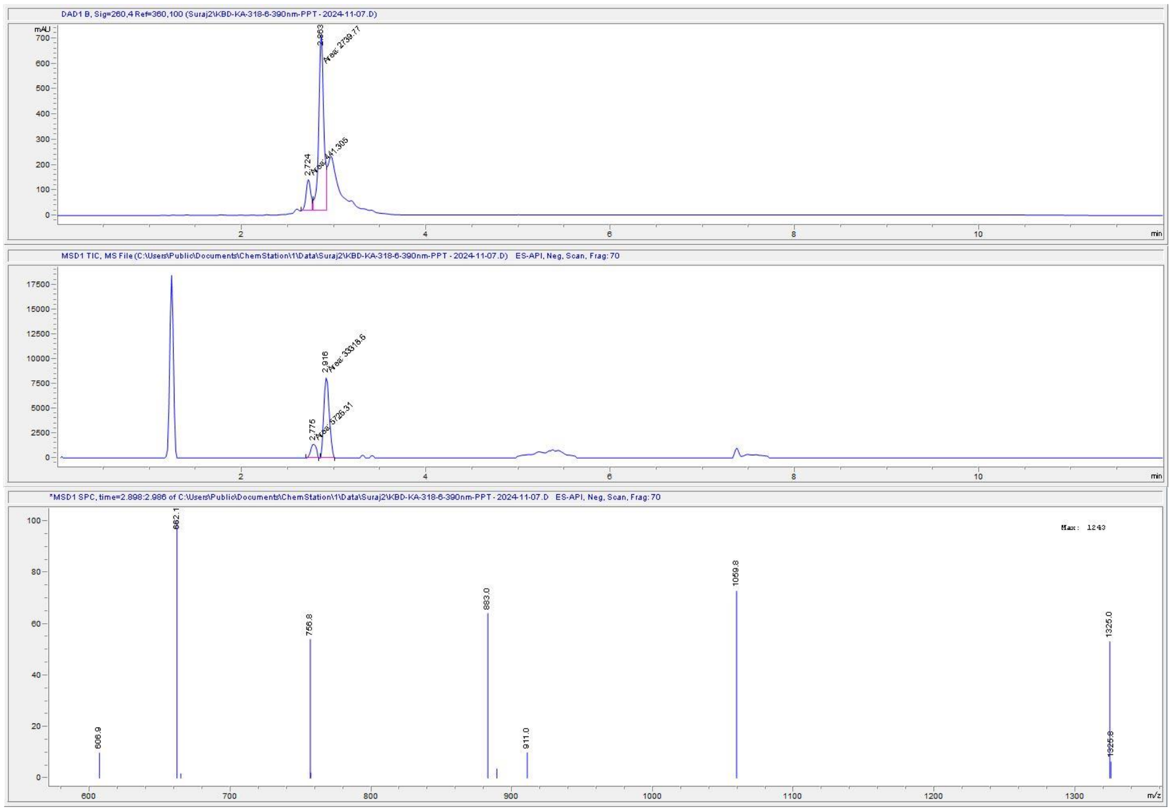

Figure S100. Analytical HPLC trace of **52** with HPLC method A. (Top) DAD chromatogram at 260 nm. (Middle) TIC chromatogram. (Bottom) Ionization of peak at 2.92 min. containing reaction product.

Synthesis of DNA-conjugate 53

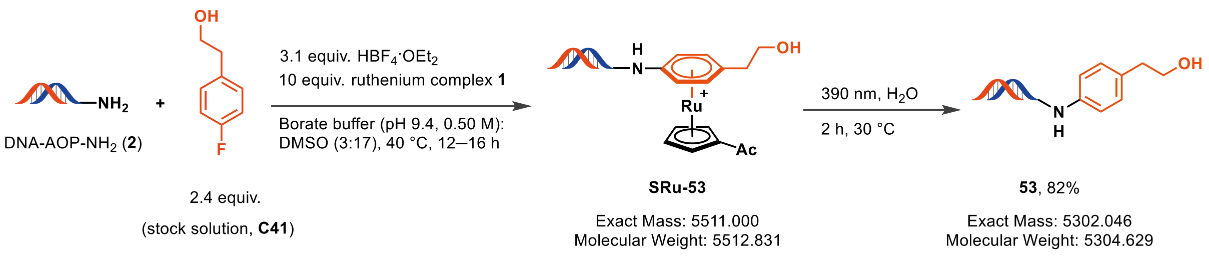

In situ formation of fluoroarene-ruthenium stock solution: Under an ambient atmosphere, a 1 mL glass GC vial equipped with a 6 mm Teflon-coated stirring bar was charged with ruthenium complex **1** (1.0 mg, 2.1 µmol, 1.0 equiv.) and then 100 µL of DMC was added to the reaction vial. In a separate Eppendorf tube was added a stock solution of $HBF_4 \cdot OEt_2$ (50 µL, c = 0.13 M, 6.5 µmol, 3.1 equiv.), followed by a stock solution of 2-(4-fluorophenyl)ethan-1-ol in DMC (50 µL, c = 0.10 M, 5.0 µmol, 2.4 equiv.). The contents of the Eppendorf tube was then transferred to the reaction vial. The vial was then closed with a screw cap and the resulting reaction mixture was heated at 80 °C for 2 hours. After 2 hours, the reaction mixture was cooled to 23 °C and the DMC was removed under a gentle stream of argon and 200 µL of DMSO was added to result in an in situ formed stock solution of arene-ruthenium complex **C41** in DMSO (200 µL, c = 0.01 M, assuming quantitative arene coordination to ruthenium).

Under an ambient atmosphere, the stock solution **SD-01** of DNA-AOP-NH_2 (**2**) (1.0 µL, c = 2.0 mM, 2.0 nmol, 1.0 equiv.) in water was added to a 1.5 mL Eppendorf tube, followed by sodium borate buffer (2.0 µL, pH 9.4, c = 0.50 M). To this mixture, 15 µL of DMSO was added and the solution was vortexed for 5 seconds. Next, the freshly prepared stock solution **C41** (2.0 µL, c = 0.01 M, 0.02 µmol, 10 equiv.) in DMSO was added. The resulting reaction mixture was vortexed for 5 seconds, transferred to a thermocycler at 40 °C, and incubated for 16 hours at 800 rpm to yield the DNA-conjugate **SRu-53**. To the reaction mixture was added the stock solution of NaCl in water (**SR-06** , 2.0 µL, c = 5.0 M, 10% volume of the total reaction volume), followed by cold ethanol (−20 °C, 66 µL) to precipitate the *N*-arylated ruthenium DNA conjugate **SRu-53**. The Eppendorf tube was placed in a freezer (−20 °C) for at least 1 hour, and then it was centrifuged at 4 °C and 11000 × g for at least 30 minutes. The supernatant was removed and the pellet was dried under air, then dissolved in 20 µL water to obtain the DNA-conjugate **SRu-53** (10 µL, c = 0.10 mM). Then, 1.0 µL of the above solution was diluted to 40 µL with water for LC–MS analysis.

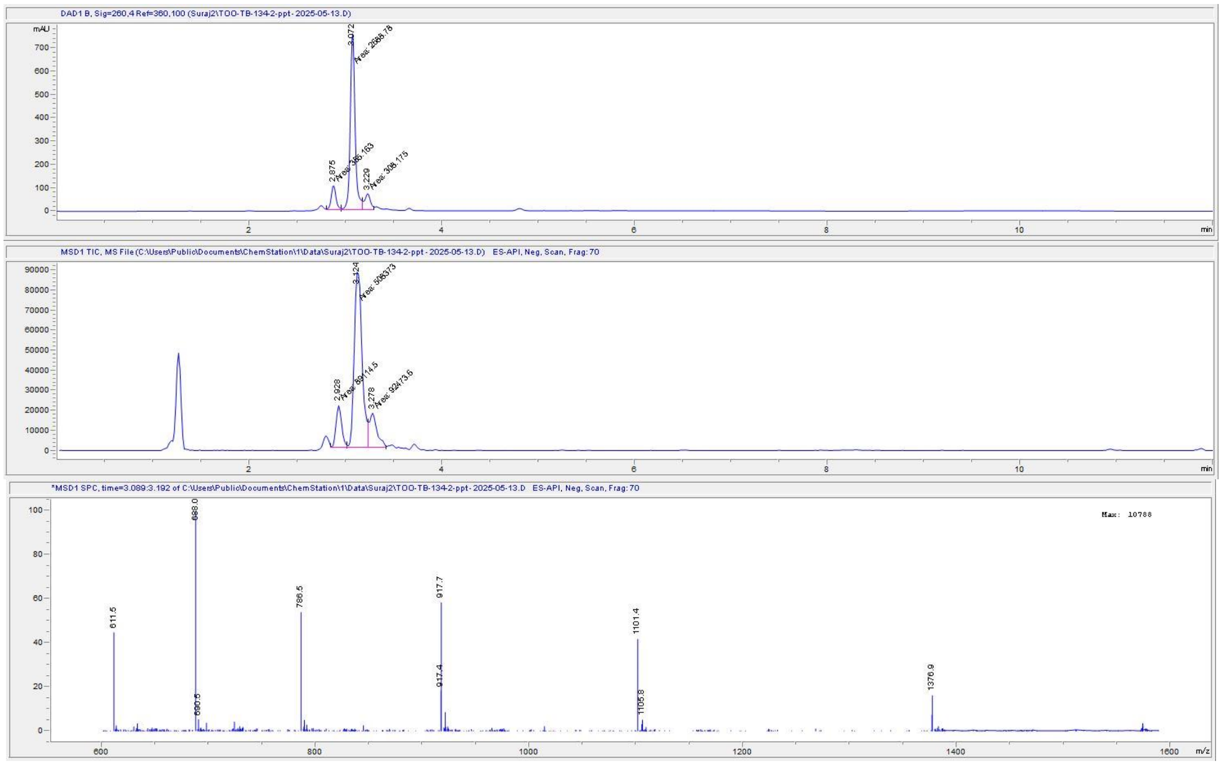

Figure S101. Analytical HPLC trace of **SRu-53** with HPLC method A. (Top) DAD chromatogram at 260 nm. (Middle) TIC chromatogram. (Bottom) Ionization of peak at 3.12 min. containing reaction product.

Decomplexation of SRu-53 to obtain product 53: Under an ambient atmosphere, the DNA-conjugate **SRu-53** stock solution in water (c = 0.10 mM, 20 µL) was irradiated with a 390 nm (40 W) Kessil lamp for 2 hours, while maintaining the temperature at approximately 30 °C through cooling with a fan. To the reaction mixture was added the stock solution of NaCl in water (**SR-06**, 2.0 µL, c = 5.0 M, 10% volume of the total reaction volume), followed by cold ethanol (−20 °C, 66 µL) to precipitate the DNA conjugate **53**. The Eppendorf tube was placed in the freezer (−20 °C) for at least 1 hour, and then it was centrifuged at 4 °C and 11000 × g for at least 30 minutes. The supernatant was removed, the pellet dried under air and dissolved in Milli-Q water to obtain the purified DNA-conjugate **53**. Then, 1 µL of the above solution was diluted to 40 µL with water for LC–MS analysis. The yield of the DNA conjugate was calculated by measuring the integration of the peaks of the diode array detection (DAD) UV absorbance at 260 nm of the LC–MS trace, assuming complete DNA recovery and identical UV absorbance.

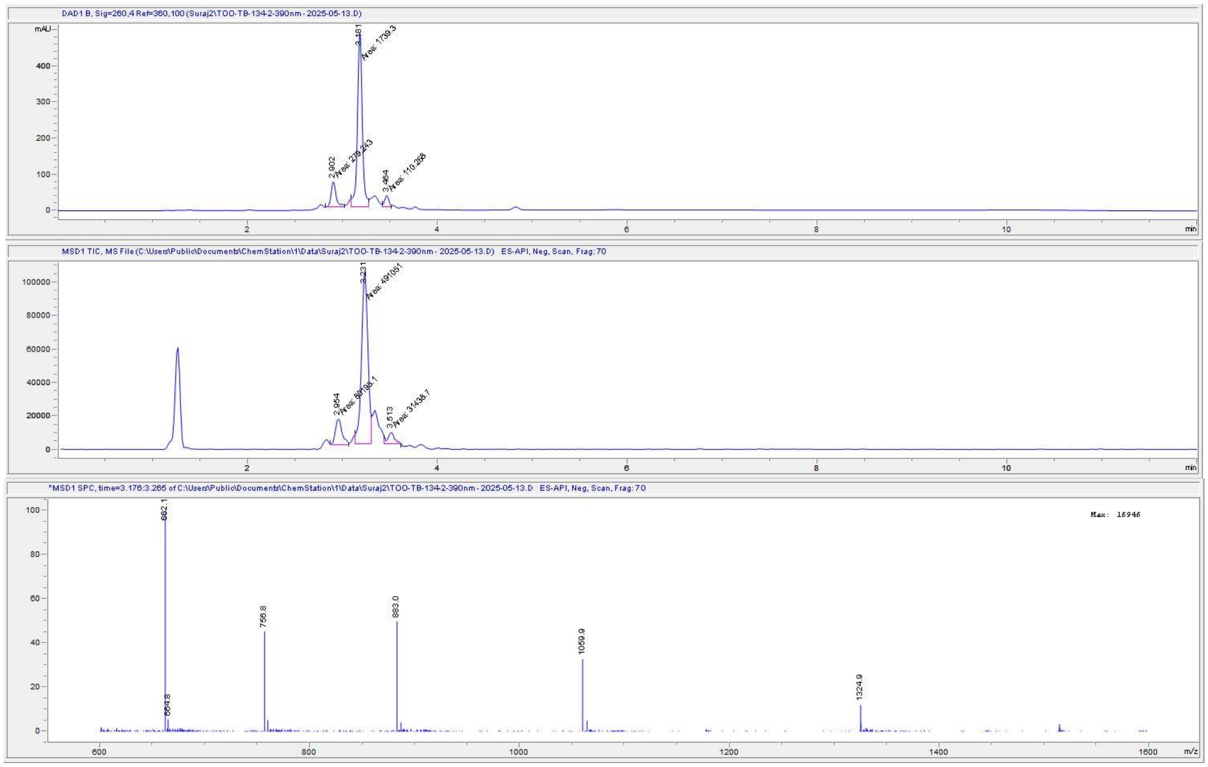

Figure S102. Analytical HPLC trace of **53** with HPLC method A. (Top) DAD chromatogram at 260 nm. (Middle) TIC chromatogram. (Bottom) Ionization of peak at 3.23 min. containing reaction product.

Synthesis of DNA-conjugate 54

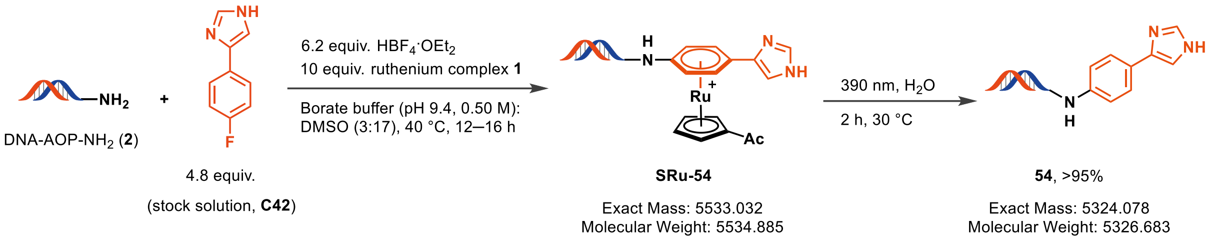

In situ formation of arene-ruthenium stock solution: Under an ambient atmosphere, a 1 mL glass GC vial equipped with a 6 mm Teflon-coated stirring bar was charged with ruthenium complex **1** (1.0 mg, 2.1 µmol, 1.0 equiv.) and then 100 µL of DMC was added to the reaction vial. In a separate Eppendorf tube was added a stock solution of $HBF_4{\cdot}OEt_2$ (50 µL, c = 0.26 M, 13 µmol, 6.2 equiv.), followed by a stock solution of 4-(4-fluorophenyl)-1*H*-imidazole in DMC (50 µL, c = 0.20 M, 10 µmol, 4.8 equiv.). The contents of the Eppendorf tube was then transferred to the reaction vial. The vial was then closed with a screw cap and the resulting reaction mixture was heated at 80 °C for 2 hours. After 2 hours, the reaction mixture was cooled to 23 °C and the DMC was removed under a gentle stream of argon and 200 µL of DMSO was added to result in an in situ formed stock solution of arene-ruthenium complex **C42** in DMSO (200 µL, c = 0.01 M, assuming quantitative arene coordination to ruthenium).

Under an ambient atmosphere, the stock solution **SD-01** of DNA-AOP-NH_2 (**2**) (1.0 µL, c = 2.0 mM, 2.0 nmol,

1.0 equiv.) in water was added to a 1.5 mL Eppendorf tube, followed by sodium borate buffer (2.0 µL, pH 9.4, c = 0.50 M). To this mixture, 15 µL of DMSO was added and the solution was vortexed for 5 seconds. Next, the freshly prepared stock solution **C42** (2.0 µL, c = 0.01 M, 0.02 µmol, 10 equiv.) in DMSO was added. The resulting reaction mixture was vortexed for 5 seconds, transferred to a thermocycler at 40 °C, and incubated for 16 hours at 800 rpm to yield the DNA-conjugate **SRu-54**. To the reaction mixture was added the stock solution of NaCl in water (**SR-06** , 2.0 µL, c = 5.0 M, 10% volume of the total reaction volume), followed by cold ethanol (−20 °C, 66 µL) to precipitate the *N*-arylated ruthenium DNA conjugate **SRu-54**. The Eppendorf tube was placed in a freezer (−20 °C) for at least 1 hour, and then it was centrifuged at 4 °C and 11000 × g for at least 30 minutes. The supernatant was removed and the pellet was dried under air, then dissolved in 20 µL water to obtain the DNA-conjugate **SRu-54** (10 µL, c = 0.10 mM). Then, 1.0 µL of the above solution was diluted to 40 µL with water for LC–MS analysis.

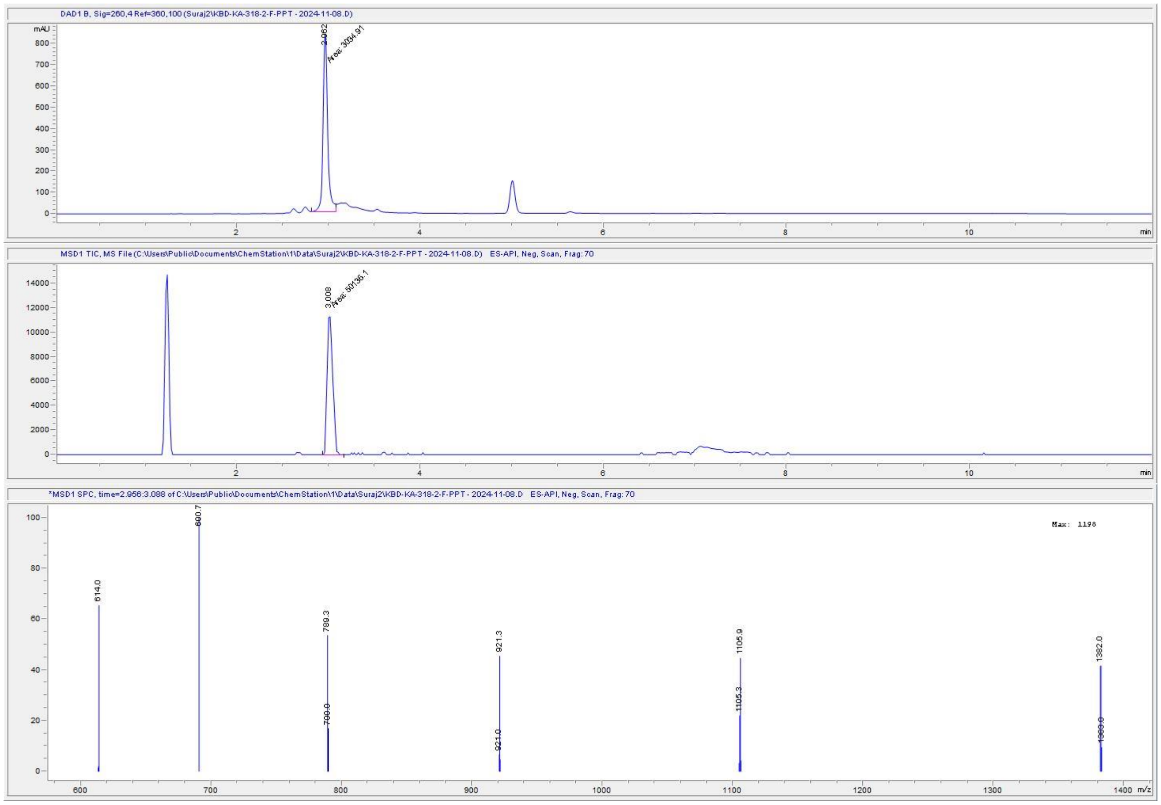

Figure S103. Analytical HPLC trace of **SRu-54** with HPLC method A. (Top) DAD chromatogram at 260 nm. (Middle) TIC chromatogram. (Bottom) Ionization of peak at 3.00 min. containing reaction product.

Decomplexation of SRu-54 to obtain product 54: Under an ambient atmosphere, the DNA-conjugate **SRu-54** stock solution in water (c = 0.10 mM, 20 µL) was irradiated with a 390 nm (40 W) Kessil lamp for 2 hours, while maintaining the temperature at approximately 30 °C through cooling with a fan. To the reaction mixture was added the stock solution of NaCl in water (**SR-06**, 2.0 µL, c = 5.0 M, 10% volume of the total reaction volume), followed by cold ethanol (−20 °C, 66 µL) to precipitate the DNA conjugate **54**. The Eppendorf tube was placed in the freezer (−20 °C) for at least 1 hour, and then it was centrifuged at 4 °C and 11000 × g for at

least 30 minutes. The supernatant was removed, the pellet dried under air and dissolved in Milli-Q water to obtain the purified DNA-conjugate **54**. Then, 1 µL of the above solution was diluted to 40 µL with water for LC–MS analysis. The yield of the DNA conjugate was calculated by measuring the integration of the peaks of the diode array detection (DAD) UV absorbance at 260 nm of the LC–MS trace, assuming complete DNA recovery and identical UV absorbance.

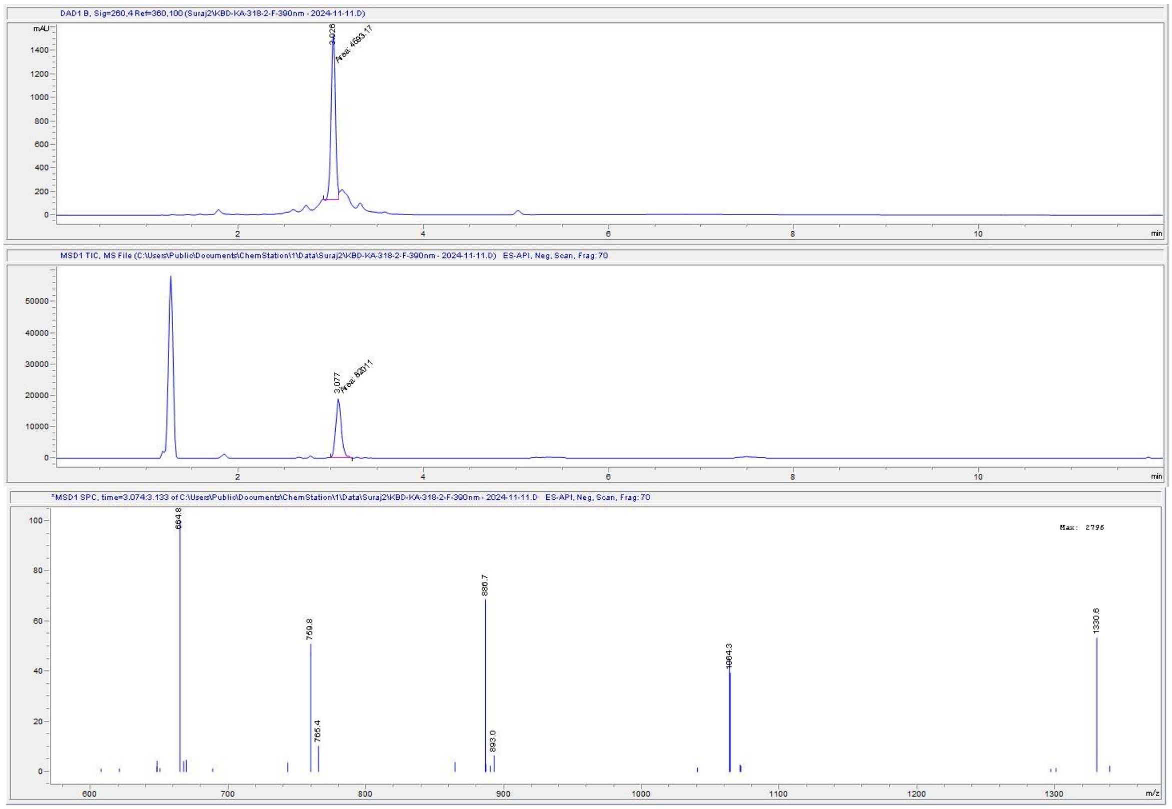

Figure S104. Analytical HPLC trace of **54** with HPLC method A. (Top) DAD chromatogram at 260 nm. (Middle) TIC chromatogram. (Bottom) Ionization of peak at 3.08 min. containing reaction product.

Synthesis of DNA-conjugate 55

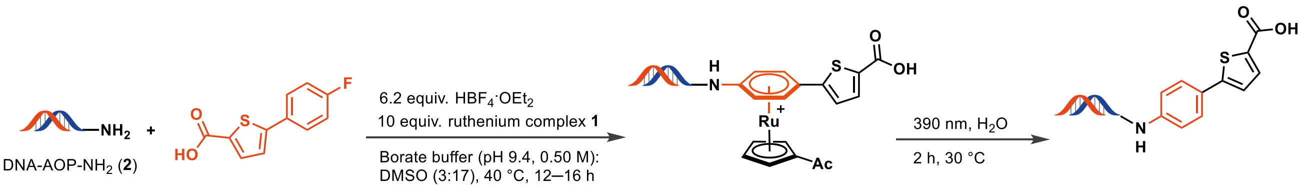

In situ formation of fluoroarene-ruthenium stock solution: Under an ambient atmosphere, a 1 mL glass GC vial equipped with a 6 mm Teflon-coated stirring bar was charged with ruthenium complex **1** (1.0 mg, 2.1 µmol, 1.0 equiv.) and then 100 µL of DMC was added to the reaction vial. In a separate Eppendorf tube was added a stock solution of $HBF_4{\cdot}OEt_2$ (50 µL, c = 0.26 M, 13 µmol, 6.2 equiv.), followed by a stock solution of

5-(4-fluorophenyl)thiophene-2-carboxylic acid in DMC (50 µL, c = 0.20 M, 10 µmol, 4.8 equiv.). The contents of the Eppendorf tube was then transferred to the reaction vial. The vial was then closed with a screw cap and the resulting reaction mixture was heated at 80 °C for 2 hours. After 2 hours, the reaction mixture was cooled to 23 °C and the DMC was removed under a gentle stream of argon and 200 µL of DMSO was added to result in an in situ formed stock solution of arene-ruthenium complex **C43** in DMSO (200 µL, c = 0.01 M, assuming quantitative arene coordination to ruthenium).

Under an ambient atmosphere, the stock solution **SD-01** of DNA-AOP-NH_2 (**2**) (1.0 µL, c = 2.0 mM, 2.0 nmol, 1.0 equiv.) in water was added to a 1.5 mL Eppendorf tube, followed by sodium borate buffer (2.0 µL, pH 9.4, c = 0.50 M). To this mixture, 15 µL of DMSO was added and the solution was vortexed for 5 seconds. Next, the freshly prepared stock solution **C43** (2.0 µL, c = 0.01 M, 0.02 µmol, 10 equiv.) in DMSO was added. The resulting reaction mixture was vortexed for 5 seconds, transferred to a thermocycler at 40 °C, and incubated for 16 hours at 800 rpm to yield the DNA-conjugate **SRu-55**. To the reaction mixture was added the stock solution of NaCl in water (**SR-06** , 2.0 µL, c = 5.0 M, 10% volume of the total reaction volume), followed by cold ethanol (−20 °C, 66 µL) to precipitate the *N*-arylated ruthenium DNA conjugate **SRu-55**. The Eppendorf tube was placed in a freezer (−20 °C) for at least 1 hour, and then it was centrifuged at 4 °C and 11000 × g for at least 30 minutes. The supernatant was removed and the pellet was dried under air, then dissolved in 20 µL water to obtain the DNA-conjugate **SRu-55** (10 µL, c = 0.10 mM). Then, 1.0 µL of the above solution was diluted to 40 µL with water for LC–MS analysis.

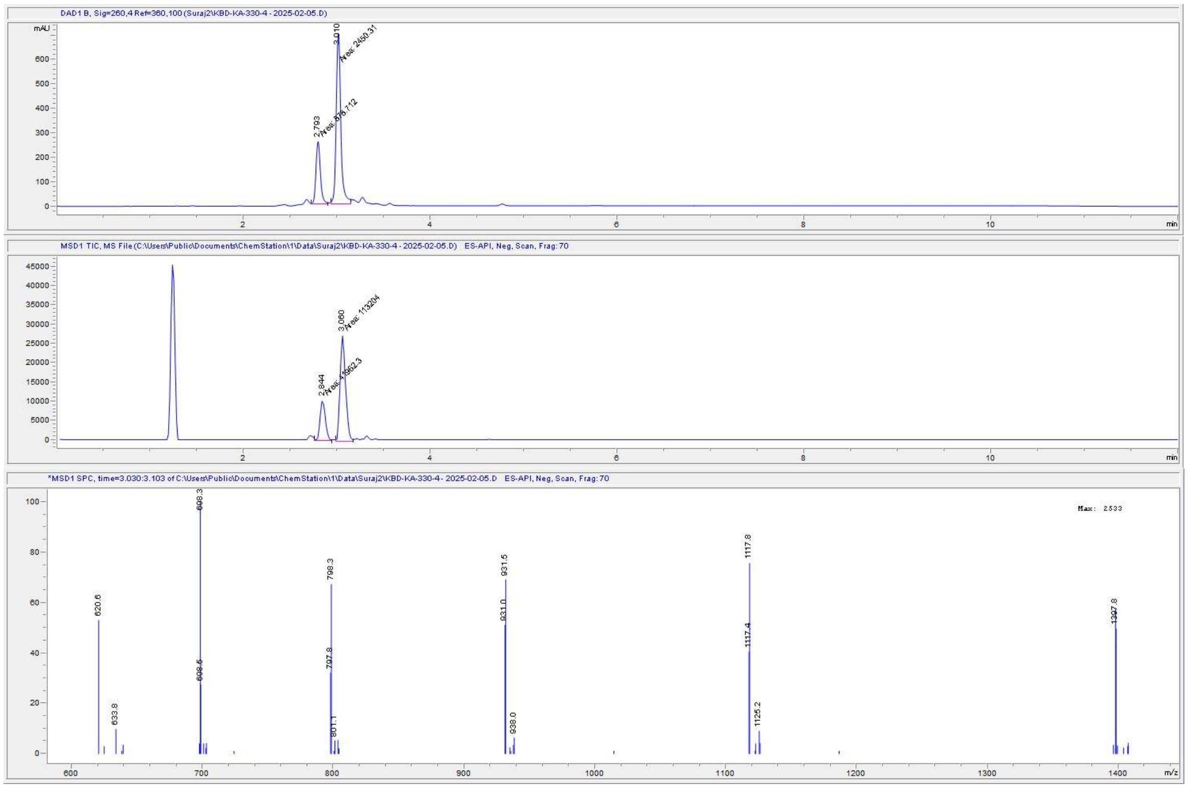

Figure S105. Analytical HPLC trace of **SRu-55** with HPLC method A. (Top) DAD chromatogram at 260 nm. (Middle) TIC chromatogram. (Bottom) Ionization of peak at 3.06 min. containing reaction product.

Decomplexation of SRu-55 to obtain product 55: Under an ambient atmosphere, the DNA-conjugate **SRu-55** stock solution in water (c = 0.10 mM, 20 µL) was irradiated with a 390 nm (40 W) Kessil lamp for 2 hours, while maintaining the temperature at approximately 30 °C through cooling with a fan. To the reaction mixture was added the stock solution of NaCl in water (**SR-06**, 2.0 µL, c = 5.0 M, 10% volume of the total reaction volume), followed by cold ethanol (−20 °C, 66 µL) to precipitate the DNA conjugate **55**. The Eppendorf tube was placed in the freezer (−20 °C) for at least 1 hour, and then it was centrifuged at 4 °C and 11000 × g for at least 30 minutes. The supernatant was removed, the pellet dried under air and dissolved in Milli-Q water to obtain the purified DNA-conjugate **55**. Then, 1 µL of the above solution was diluted to 40 µL with water for LC–MS analysis. The yield of the DNA conjugate was calculated by measuring the integration of the peaks of the diode array detection (DAD) UV absorbance at 260 nm of the LC–MS trace, assuming complete DNA recovery and identical UV absorbance. In this case, HFIP adduct the product was also observed.

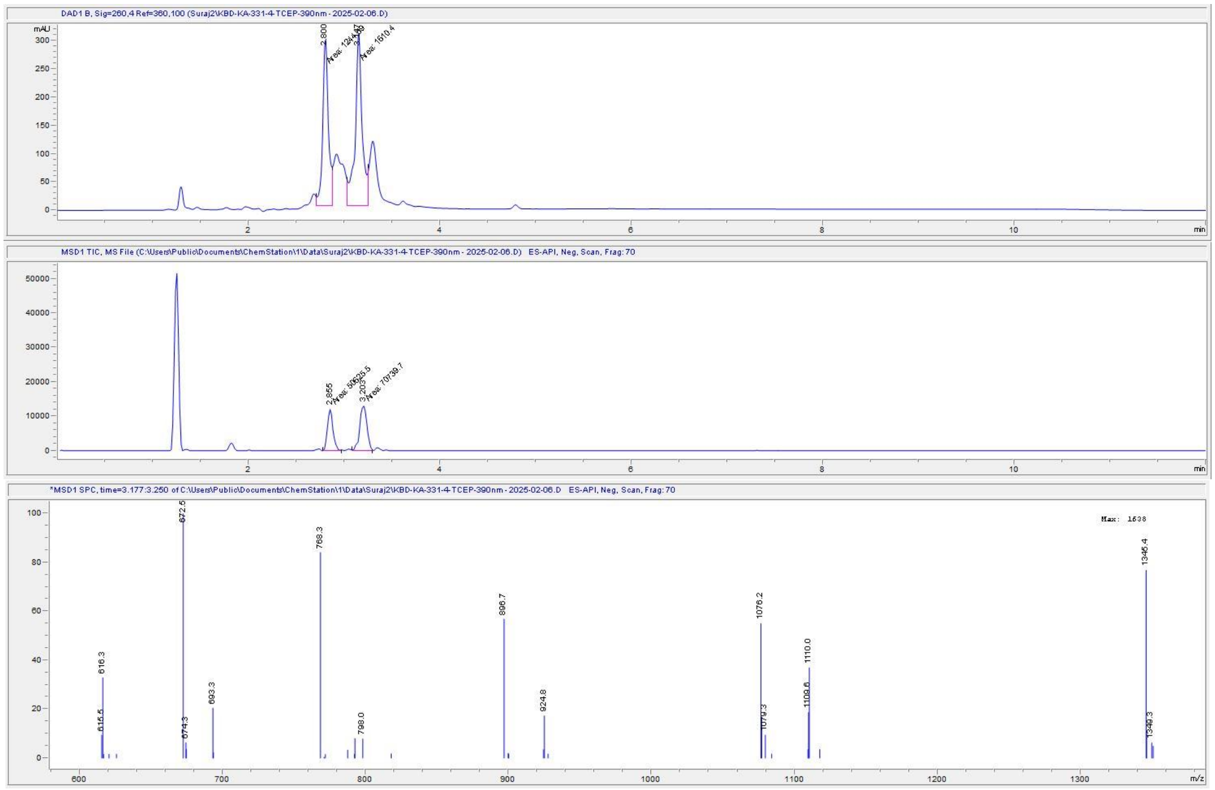

Figure S106. Analytical HPLC trace of **55** with HPLC method A. (Top) DAD chromatogram at 260 nm. (Middle) TIC chromatogram. (Bottom) Ionization of peak at 3.20 min. containing reaction product.

Synthesis of DNA-conjugate 56

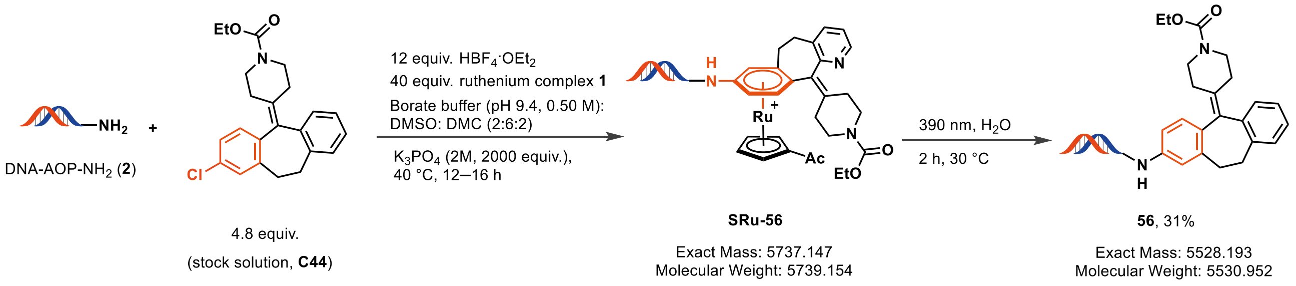

In situ formation of chloroarene-ruthenium stock solution: Under an ambient atmosphere, a 1 mL glass GC vial equipped with a 6 mm Teflon-coated stirring bar was charged with ruthenium complex **1** (1.0 mg, 2.1 µmol, 1.0 equiv.) and then 50 µL of DMC was added to the reaction vial. In a separate Eppendorf tube was added a stock solution of $HBF_4·OEt_2$ (25 µL, c = 1.0 M, 25 µmol, 12 equiv.), followed by a stock solution of Loratadine in DMC (25 µL, c = 0.40 M, 10 µmol, 4.8 equiv.). The contents of the Eppendorf tube was then transferred to the reaction vial. The vial was then closed with a screw cap and resulting reaction mixture was heated at 80 °C for 2 hours. After 2 hours, the reaction mixture was cooled to 23 °C, resulting in an in situ formed stock solution of arene-ruthenium complex **C44** in DMC (105 µL, c = 0.02 M, assuming quantitative arene coordination to ruthenium).

Under an ambient atmosphere, a stock solution of DNA-AOP-NH_2 (**2**) (1.0 µL, c = 1.0 mM, 1.0 nmol, 1.0 equiv.) in sodium borate buffer was added to a 1.5 mL Eppendorf tube, followed by a stock solution of K_3PO_4 in water (1.0 µL, c = 2.0 M). To this mixture, 6 µL of DMSO was added and the solution was vortexed for 5 seconds. Next, the freshly prepared stock solution **C44** (2.0 µL, c = 0.02 M, 0.04 µmol, 40 equiv.) in DMC was then added. The resulting reaction mixture was vortexed for 5 seconds, transferred to a thermocycler at 40 °C, and incubated for 16 hours at 800 rpm to yield the DNA-conjugate **SRu-56**. Next, the reaction mixture was diluted with 10 µL of Milli-Q water. To the reaction mixture was added the stock solution of NaCl in water (**SR-06** , 2.0 µL, c = 5.0 M, 10% volume of the total reaction volume), followed by cold ethanol (−20 °C, 66 µL) to precipitate the *N*-arylated ruthenium DNA conjugate **SRu-56**. The Eppendorf tube was placed in a freezer (−20 °C) for at least 1 hour, and then it was centrifuged at 4 °C and 11000 × g for at least 30 minutes. The supernatant was removed and the pellet was dried under air, then dissolved in 20 µL water to obtain the DNA-conjugate **SRu-56** (10 µL, c = 0.10 mM). Then, 1.0 µL of the above solution was diluted to 40 µL with water for LC–MS analysis.

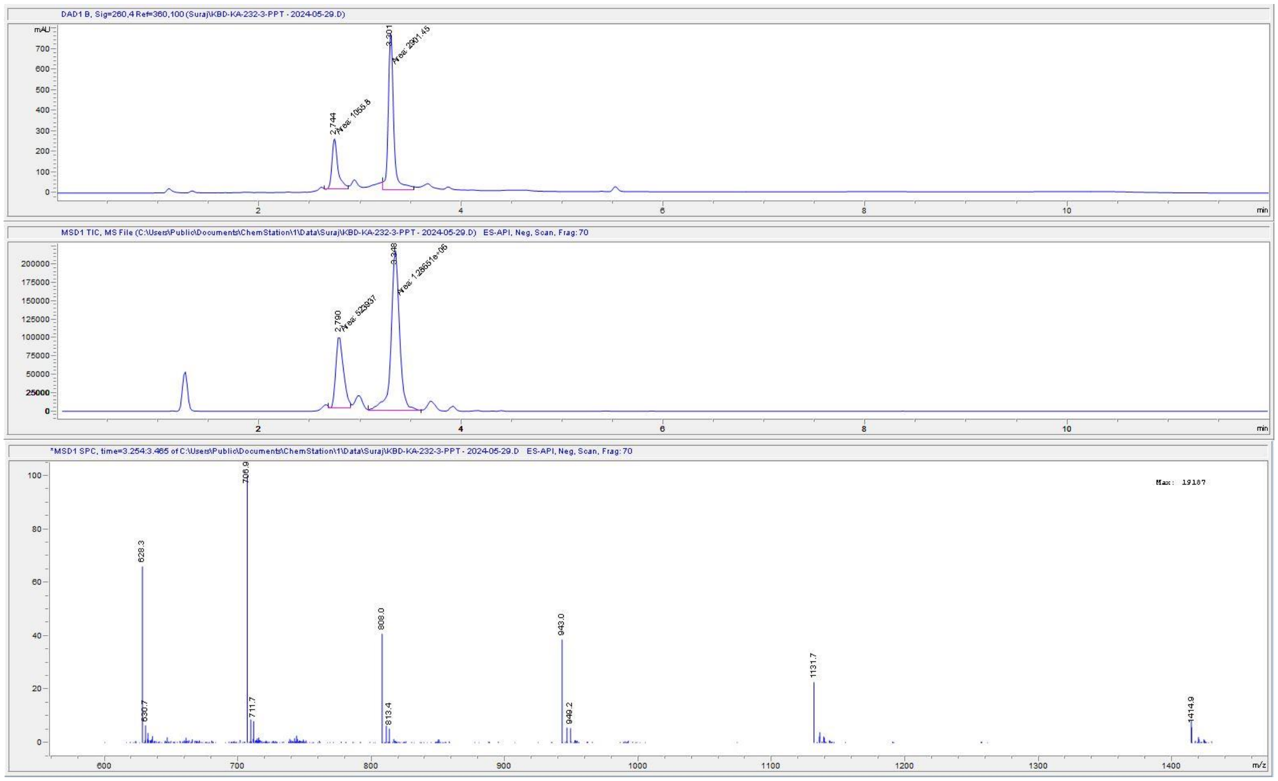

Figure S107. Analytical HPLC trace of **SRu-56** with HPLC method A. (Top) DAD chromatogram at 260 nm. (Middle) TIC chromatogram. (Bottom) Ionization of peak at 3.35 min. containing reaction product.

Decomplexation of SRu-56 to obtain product 56: Under an ambient atmosphere, the DNA-conjugate **SRu-56** stock solution in water (c = 0.10 mM, 20 µL) was irradiated with a 390 nm (40 W) Kessil lamp for 2 hours, while maintaining the temperature at approximately 30 °C through cooling with a fan. To the reaction mixture was added the stock solution of NaCl in water (**SR-06**, 2.0 µL, c = 5.0 M, 10% volume of the total reaction volume), followed by cold ethanol (−20 °C, 66 µL) to precipitate the DNA conjugate **56**. The Eppendorf tube was placed in the freezer (−20 °C) for at least 1 hour, and then it was centrifuged at 4 °C and 11000 × g for at least 30 minutes. The supernatant was removed, the pellet dried under air and dissolved in Milli-Q water to obtain the purified DNA-conjugate **56**. Then, 1 µL of the above solution was diluted to 40 µL with water for LC–MS analysis. The yield of the DNA conjugate was calculated by measuring the integration of the peaks of the diode array detection (DAD) UV absorbance at 260 nm of the LC–MS trace, assuming complete DNA recovery and identical UV absorbance.

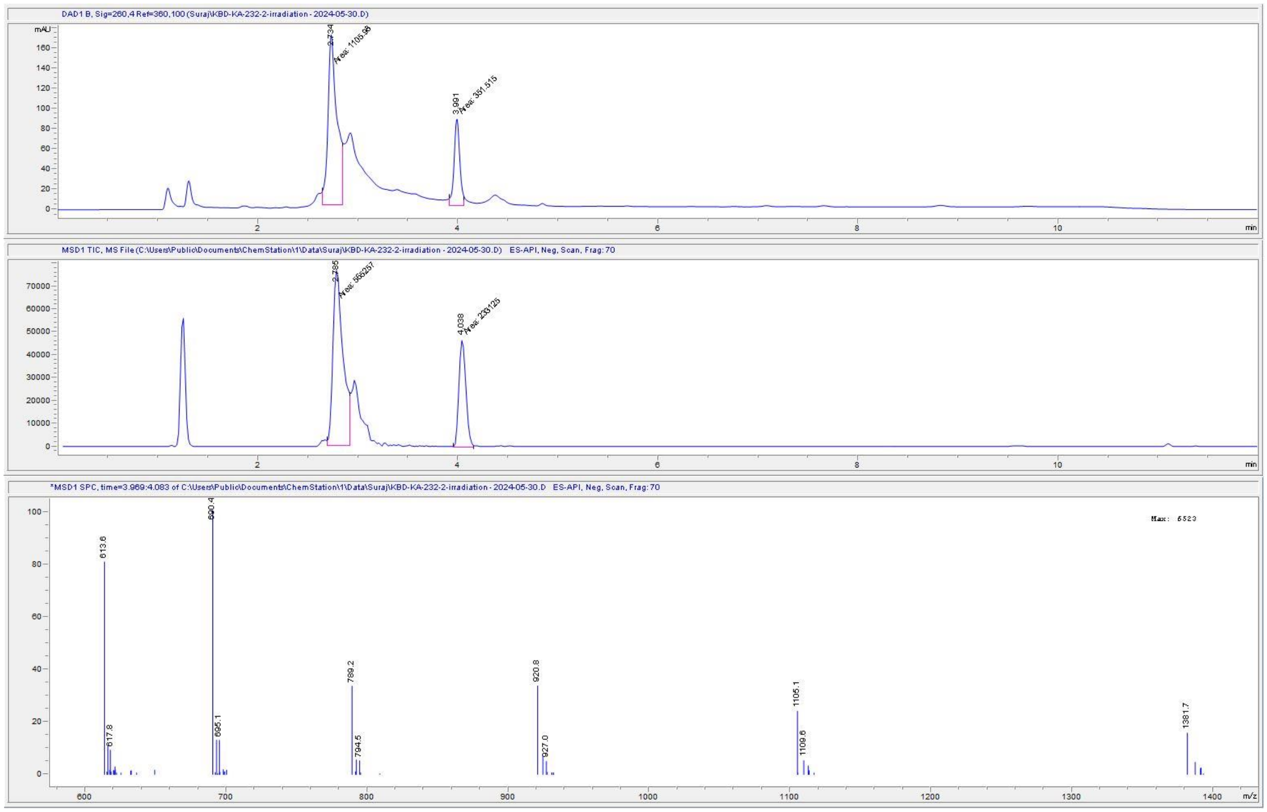

Figure S108. Analytical HPLC trace of **56** with HPLC method A. (Top) DAD chromatogram at 260 nm. (Middle) TIC chromatogram. (Bottom) Ionization of peak at 4.04 min. containing reaction product.

Synthesis of DNA-conjugate 57

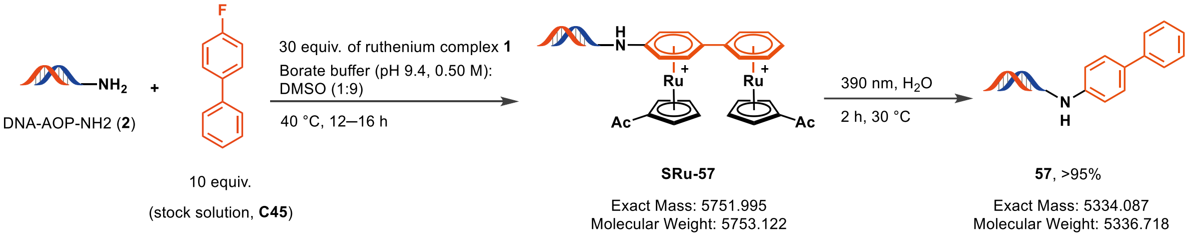

In situ formation of arene-ruthenium stock solution: Under an ambient atmosphere, a 1 mL glass GC vial equipped with a 6 mm Teflon-coated stirring bar was charged with ruthenium complex **1** (2.0 mg, 4.2 µmol, 3.0 equiv.), followed by 70 µL DMC. Next, a stock solution of 4-fluoro-1,1'-biphenyl in DMC (70 µL, c = 0.020 M, 1.0 equiv.) was added to the glass vial. The vial was closed with a screw cap. The resulting reaction mixture was heated at 80 °C for 2 hours. After 2 hours, the reaction mixture was cooled to 23 °C and the DMC was removed under a gentle stream of argon and 140 µL of DMSO were added to result an in situ formed stock solution of arene-ruthenium complex **C45** (140 µL, c = 0.01 M, assuming quantitative arene coordination to ruthenium).

Under an ambient atmosphere, the stock solution **SD-01** of DNA-AOP-NH2 (**2**) in water (1.0 µL, c = 2.0 mM, 2.0 nmol, 1.0 equiv.) was added to a 1.5 mL Eppendorf tube, followed by sodium borate buffer (1.0 µL, pH

9.4, c = 0.50 M). To this mixture, 16 µL of DMSO was added and the solution was vortexed for 5 seconds. Next, the freshly prepared stock solution **C45** (2.0 µL, c = 0.01 M, 0.02 µmol, 10 equiv.) in DMSO was added. The resulting reaction mixture was vortexed for 5 seconds, transferred to a thermocycler at 40 °C, and incubated for 16 hours at 800 rpm to yield the DNA-conjugate **SRu-57**. Next, the reaction mixture was diluted with 10 µL of Milli-Q water. To the reaction mixture was added the stock solution of NaCl in water (**SR-06** , 3.0 µL, c = 5.0 M, 10% volume of the total reaction volume), followed by cold ethanol (−20 °C, 99 µL) to precipitate the *N*-arylated ruthenium DNA conjugate **SRu-57**. The Eppendorf tube was placed in a freezer (−20 °C) for at least 1 hour, and then it was centrifuged at 4 °C and 11000 × g for at least 30 minutes. The supernatant was removed and the pellet was dried under air, then dissolved in 20 µL water to obtain the DNA-conjugate **SRu-57** (20 µL, c = 0.10 mM). Then, 1.0 µL of the above solution was diluted to 40 µL with water for LC–MS analysis.

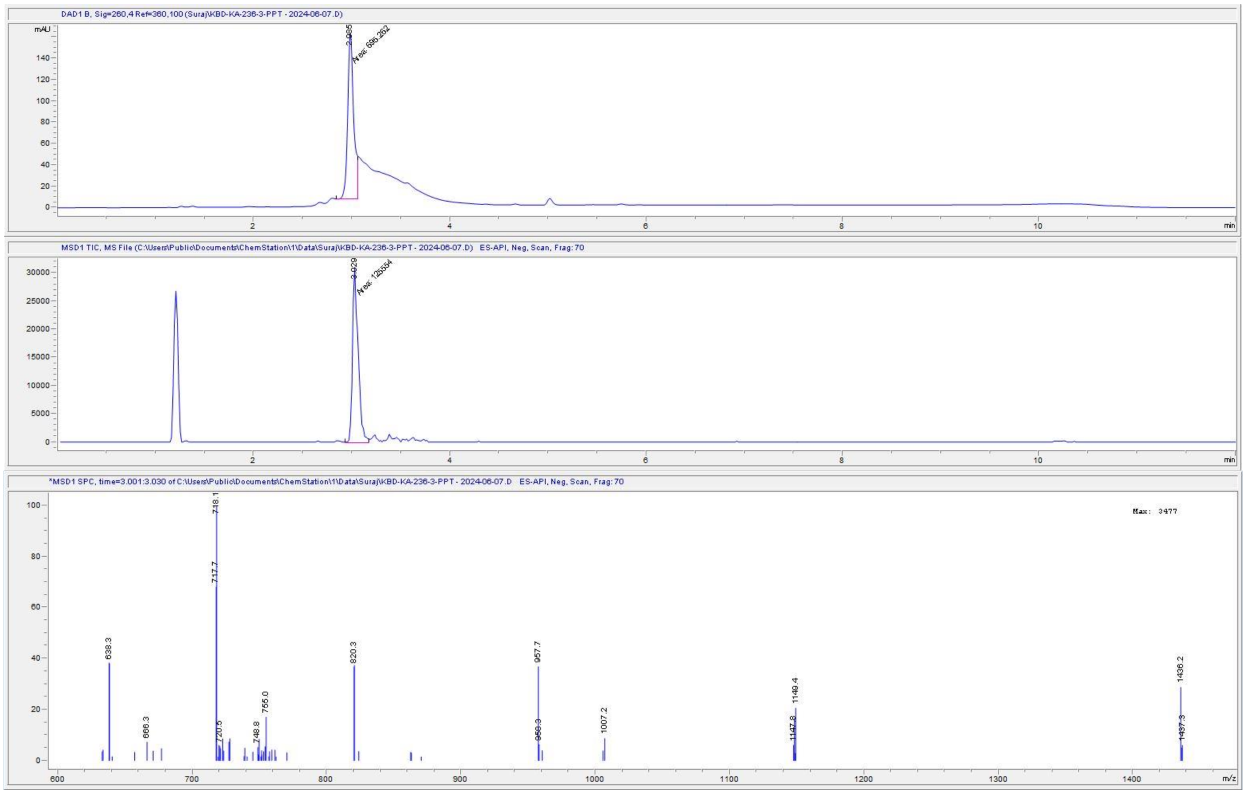

Figure S109. Analytical HPLC trace of **SRu-57** with HPLC method A. (Top) DAD chromatogram at 260 nm. (Middle) TIC chromatogram. (Bottom) Ionization of peak at 3.03 min. containing reaction product.

Decomplexation of SRu-57 to obtain product 57: Under an ambient atmosphere, the DNA-conjugate **SRu-57** stock solution in water (c = 0.10 mM, 20 µL) was irradiated with a 390 nm (40 W) Kessil lamp for 2 hours, while maintaining the temperature at approximately 30 °C through cooling with a fan. To the reaction mixture was added the stock solution of NaCl in water (**SR-06**, 2.0 µL, c = 5.0 M, 10% volume of the total reaction volume), followed by cold ethanol (−20 °C, 66 µL) to precipitate the DNA conjugate **57**. The Eppendorf tube was placed in the freezer (−20 °C) for at least 1 hour, and then it was centrifuged at 4 °C and 11000 × g for at

least 30 minutes. The supernatant was removed, the pellet dried under air and dissolved in Milli-Q water to obtain the purified DNA-conjugate **57**. Then, 1 µL of the above solution was diluted to 40 µL with water for LC–MS analysis. The yield of the DNA conjugate was calculated by measuring the integration of the peaks of the diode array detection (DAD) UV absorbance at 260 nm of the LC–MS trace, assuming complete DNA recovery and identical UV absorbance.

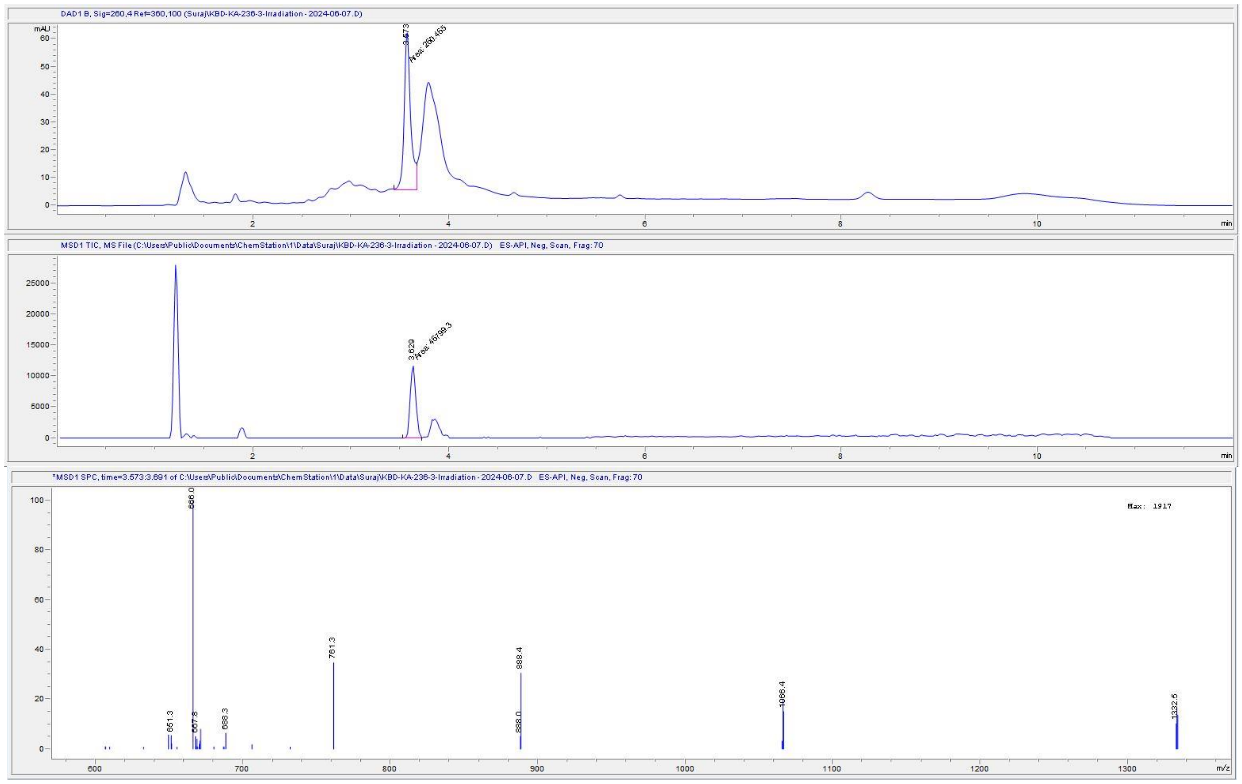

Figure S110. Analytical HPLC trace of **57** with HPLC method A. (Top) DAD chromatogram at 260 nm. (Middle) TIC chromatogram. (Bottom) Ionization of peak at 3.63 min. containing reaction product.

Synthesis of DNA-conjugate 58

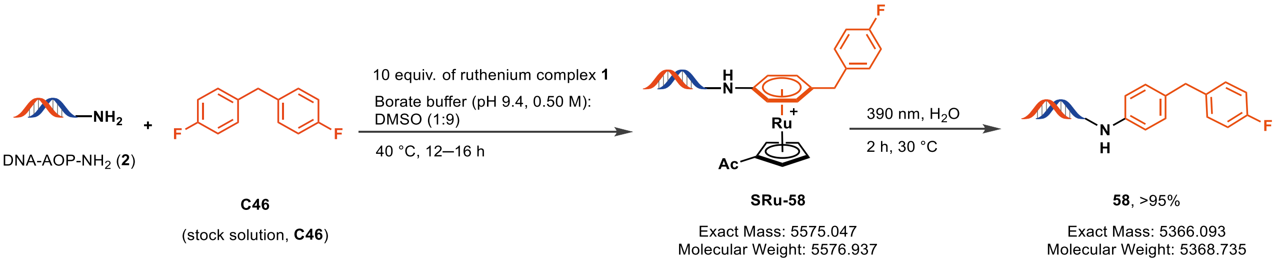

In situ formation of arene-ruthenium stock solution: Under an ambient atmosphere, a 1 mL glass GC vial equipped with a 6 mm Teflon-coated stirring bar was charged with ruthenium complex **1** (1.4 mg, 2.9 µmol, 1.0 equiv.). Next, a stock solution of bis(4-fluorophenyl)methane in DMC (294 µL, c = 0.10 M, 29 µmol, 10 equiv.) was added. The resulting reaction mixture was heated at 80 °C for 2 hours. After 2 hours, the reaction mixture was cooled to 23 °C. Next, the DMC was removed under a gentle stream of argon and 294 µL of

DMSO were added to result an in situ formed stock solution of arene-ruthenium complex **C46** (294 µL, c = 0.01 M, assuming quantitative arene coordination to ruthenium).

Under an ambient atmosphere, the stock solution **SD-01** of DNA-AOP-NH_2 (**2**) in water (1.0 µL, c = 2.0 mM, 2.0 nmol, 1.0 equiv.) was added to a 1.5 mL Eppendorf tube, followed by sodium borate buffer (1.0 µL, pH 9.4, c = 0.50 M). To this mixture, 16 µL of DMSO was added and the solution was vortexed for 5 seconds. The freshly prepared stock solution **C46** (2.0 µL, c = 0.01 M, 0.02 µmol, 10 equiv.) in DMSO was added. The resulting reaction mixture was vortexed for 5 seconds, transferred to a thermocycler at 40 °C, and incubated for 16 hours at 800 rpm to yield the DNA-conjugate **SRu-58**. Next, the reaction mixture was diluted with 10 µL of Milli-Q water. To the reaction mixture was added the stock solution of NaCl in water (**SR-06** , 3.0 µL, c = 5.0 M, 10% volume of the total reaction volume), followed by cold ethanol (−20 °C, 99 µL) to precipitate the *N*-arylated ruthenium DNA conjugate **SRu-58**. The Eppendorf tube was placed in a freezer (−20 °C) for at least 1 hour, and then it was centrifuged at 4 °C and 11000 × g for at least 30 minutes. The supernatant was removed and the pellet was dried under air, then dissolved in 20 µL water to obtain the DNA-conjugate **SRu-58** (20 µL, c = 0.10 mM). Then, 1.0 µL of the above solution was diluted to 40 µL with water for LC–MS analysis.

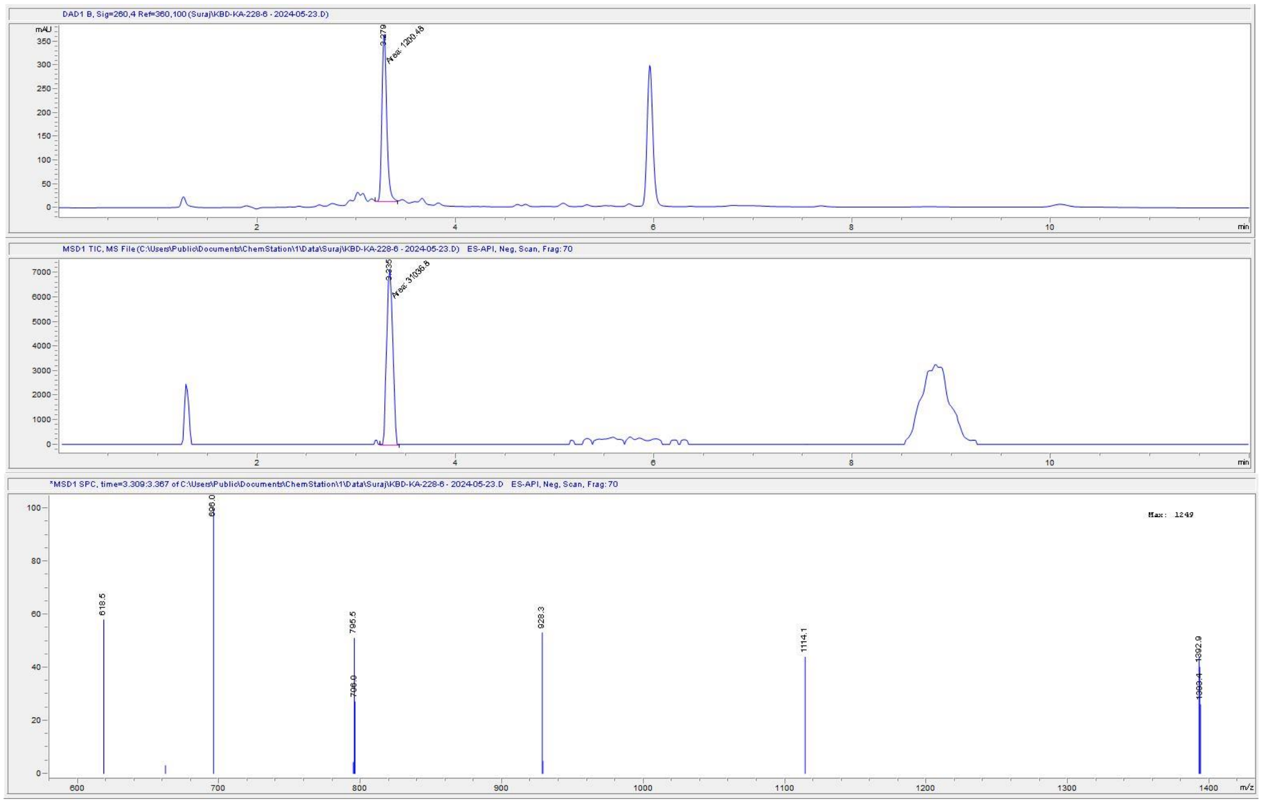

Figure S111. Analytical HPLC trace of **SRu-58** with HPLC method A. (Top) DAD chromatogram at 260 nm. (Middle) TIC chromatogram. (Bottom) Ionization of peak at 3.34 min. containing reaction product.

Decomplexation of SRu-58 to obtain product 58: Under an ambient atmosphere, the DNA-conjugate **SRu-58** stock solution in water (c = 0.10 mM, 20 µL) was irradiated with a 390 nm (40 W) Kessil lamp for 2 hours,

while maintaining the temperature at approximately 30 °C through cooling with a fan. To the reaction mixture was added the stock solution of NaCl in water (**SR-06**, 2.0 µL, c = 5.0 M, 10% volume of the total reaction volume), followed by cold ethanol (−20 °C, 66 µL) to precipitate the DNA conjugate **58**. The Eppendorf tube was placed in the freezer (−20 °C) for at least 1 hour, and then it was centrifuged at 4 °C and 11000 × g for at least 30 minutes. The supernatant was removed, the pellet dried under air and dissolved in Milli-Q water to obtain the purified DNA-conjugate **58**. Then, 1 µL of the above solution was diluted to 40 µL with water for LC–MS analysis. The yield of the DNA conjugate was calculated by measuring the integration of the peaks of the diode array detection (DAD) UV absorbance at 260 nm of the LC–MS trace, assuming complete DNA recovery and identical UV absorbance.

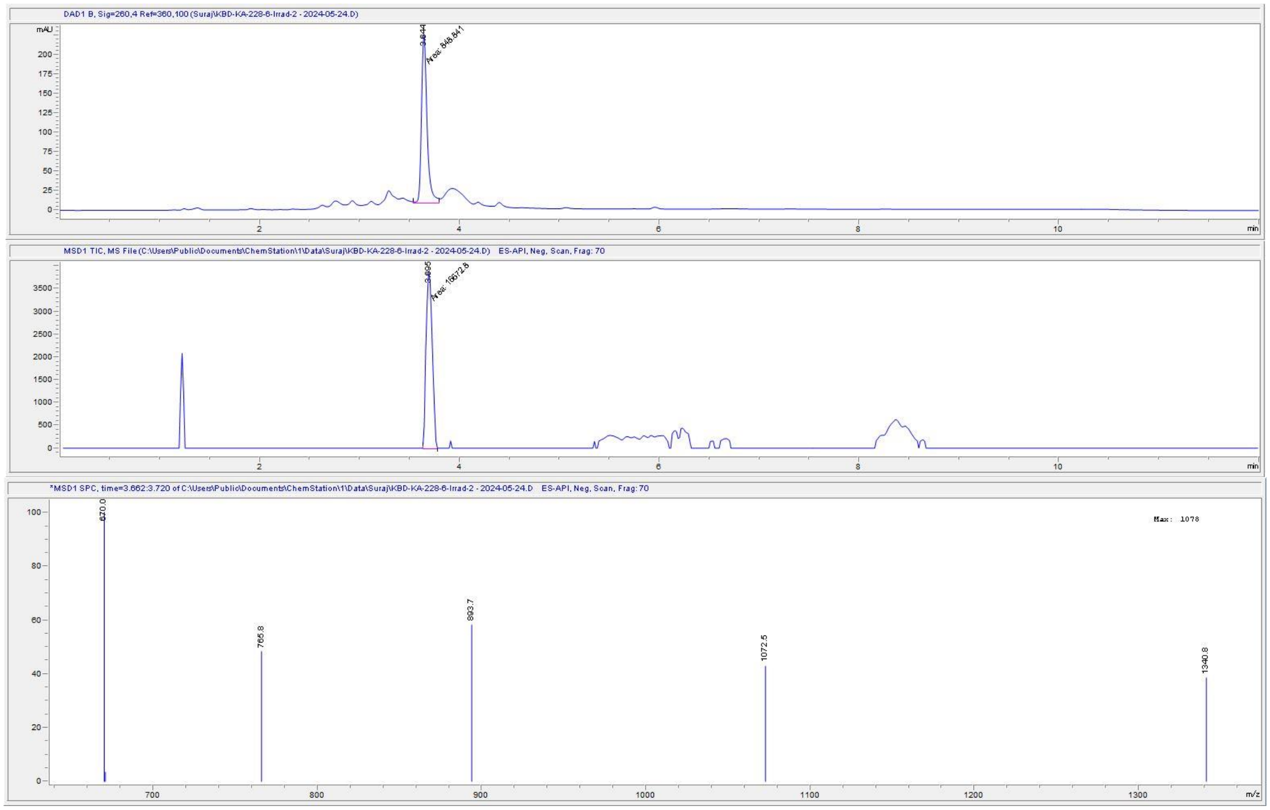

Figure S112. Analytical HPLC trace of **58** with HPLC method A. (Top) DAD chromatogram at 260 nm. (Middle) TIC chromatogram. (Bottom) Ionization of peak at 3.89 min. containing reaction product.

Synthesis of DNA-conjugate 59

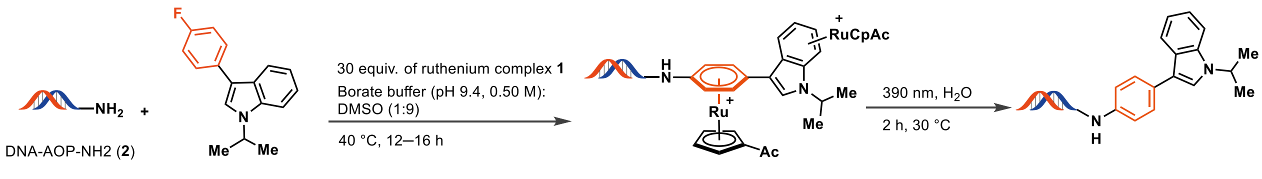

DNA-AOP-NH2 (**2**)

10 equiv.

(stock oslution, **C47**)

SRu-59

Exact Mass: 5833.053
Molecular Weight: 5834.240

59, >95%

Exact Mass: 5415.145
Molecular Weight: 5417.836

In situ formation of arene-ruthenium stock solution: Under an ambient atmosphere, a 1 mL glass GC vial equipped with a 6 mm Teflon-coated stirring bar was charged with ruthenium complex **1** (2.0 mg, 4.2 µmol, 3.0 equiv.), followed by 70 µL DMC. Next, a stock solution of 3-(4-fluorophenyl)-1-isopropyl-1*H*-indole DMC (70 µL, c = 0.020 M, 1.0 equiv.) was added to the glass vial. The vial was closed with a screw cap. The resulting reaction mixture was heated at 80 °C for 2 hours. After 2 hours, the reaction mixture was cooled to 23 °C and the DMC was removed under a gentle stream of argon and 140 µL of DMSO were added to result an in situ formed stock solution of arene-ruthenium complex **C47** (140 µL, c = 0.01 M, assuming quantitative arene coordination to ruthenium).

Under an ambient atmosphere, the stock solution **SD-01** of DNA-AOP-NH_2 (**2**) in water (1.0 µL, c = 2.0 mM, 2.0 nmol, 1.0 equiv.) was added to a 1.5 mL Eppendorf tube, followed by sodium borate buffer (1.0 µL, pH 9.4, c = 0.50 M). To this mixture, 16 µL of DMSO was added and the solution was vortexed for 5 seconds. Next, the freshly prepared stock solution **C47** (2.0 µL, c = 0.01 M, 0.02 µmol, 10 equiv.) in DMSO was added. The resulting reaction mixture was vortexed for 5 seconds, transferred to a thermocycler at 40 °C, and incubated for 16 hours at 800 rpm to yield the DNA-conjugate **SRu-59**. Next, the reaction mixture was diluted with 10 µL of Milli-Q water. To the reaction mixture was added the stock solution of NaCl in water (**SR-06** , 3.0 µL, c = 5.0 M, 10% volume of the total reaction volume), followed by cold ethanol (−20 °C, 99 µL) to precipitate the *N*-arylated ruthenium DNA conjugate **SRu-59**. The Eppendorf tube was placed in a freezer (−20 °C) for at least 1 hour, and then it was centrifuged at 4 °C and 11000 × g for at least 30 minutes. The supernatant was removed and the pellet was dried under air, then dissolved in 20 µL water to obtain the DNA-conjugate **SRu-59** (20 µL, c = 0.10 mM). Then, 1.0 µL of the above solution was diluted to 40 µL with water for LC–MS analysis. The LC–MS analysis showed the solution was consistent of a mixture of DNA conjugates **SRu-59** and **SRu-59’** (3:2).

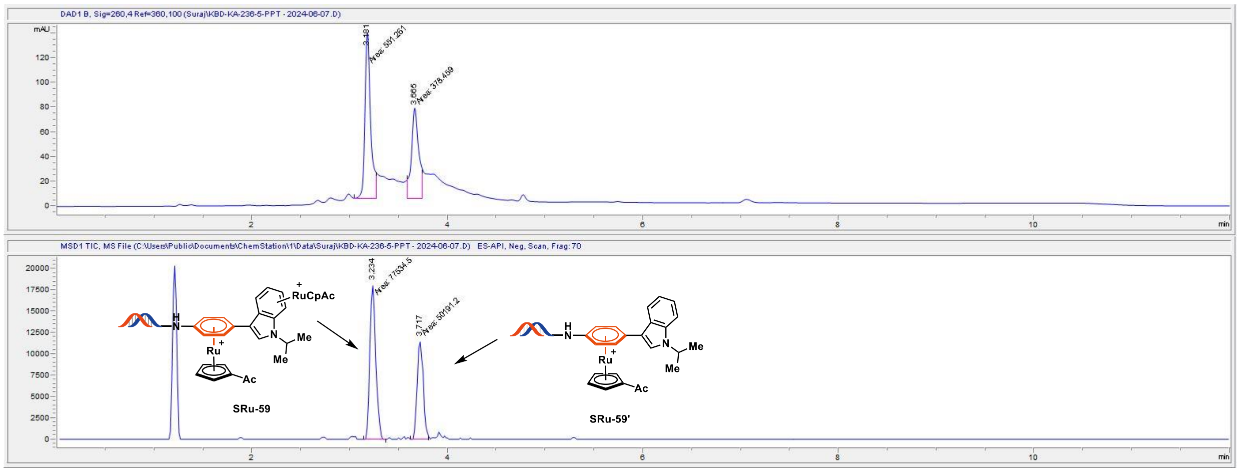

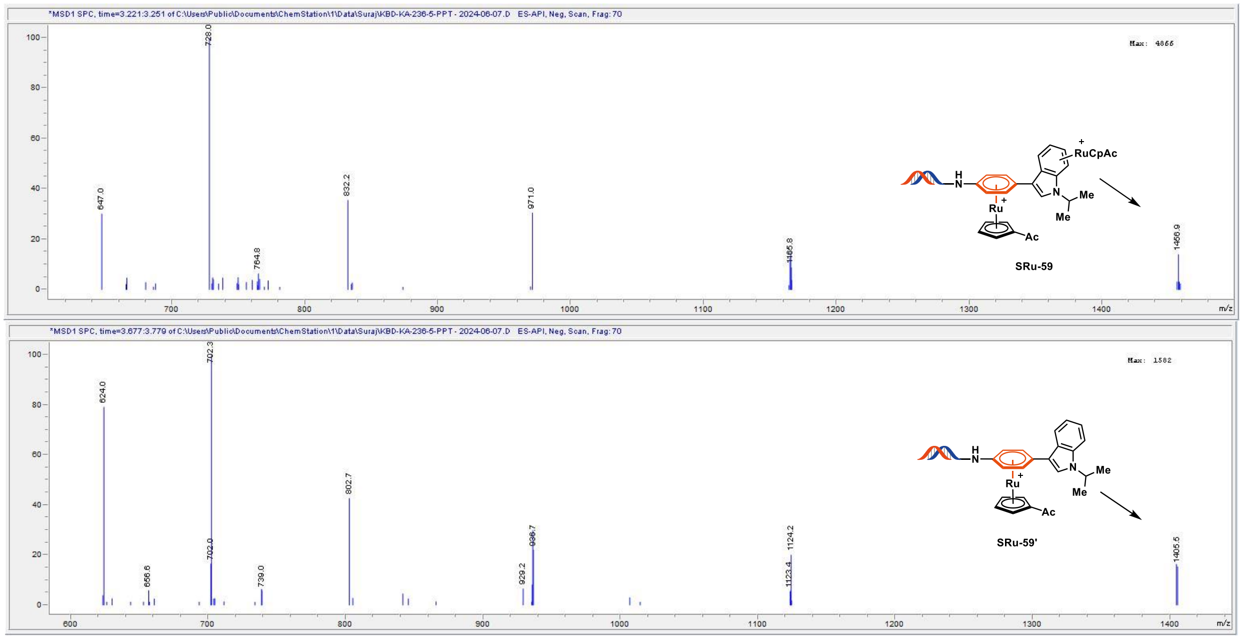

Figure S113. Analytical HPLC trace of **SRu-59** and **SRu-59'** with HPLC method A. (Top) DAD chromatogram at 260 nm. (Middle top) TIC chromatogram. (Middle bottom) Ionization of peak at 3.23 containing DNA-conjugate **SRu-59**. (Bottom) Ionization of peak at 3.72 min. containing DNA-conjugate **SRu-59'**.

Decomplexation of SRu-59 and SRu-59' to obtain product 59: Under an ambient atmosphere, the solution containing the mixture of DNA-conjugates **SRu-59** and **SRu-59'** in water (c = 0.10 mM, 20 µL) was irradiated with a 390 nm (40 W) Kessil lamp for 2 hours, while maintaining the temperature at approximately 30 °C through cooling with a fan. To the reaction mixture was added the stock solution of NaCl in water (**SR-06**, 2.0 µL, c = 5.0 M, 10% volume of the total reaction volume), followed by cold ethanol (−20 °C, 66 µL) to precipitate the DNA conjugate **59**. The Eppendorf tube was placed in the freezer (−20 °C) for at least 1 hour, and then it was centrifuged at 4 °C and 11000 × g for at least 30 minutes. The supernatant was removed, the pellet dried under air and dissolved in Milli-Q water to obtain the purified DNA-conjugate **59.** Then, 1 µL of the above solution was diluted to 40 µL with water for LC–MS analysis. The yield of the DNA conjugate was calculated by measuring the integration of the peaks of the diode array detection (DAD) UV absorbance at 260 nm of the LC–MS trace, assuming complete DNA recovery and identical UV absorbance.

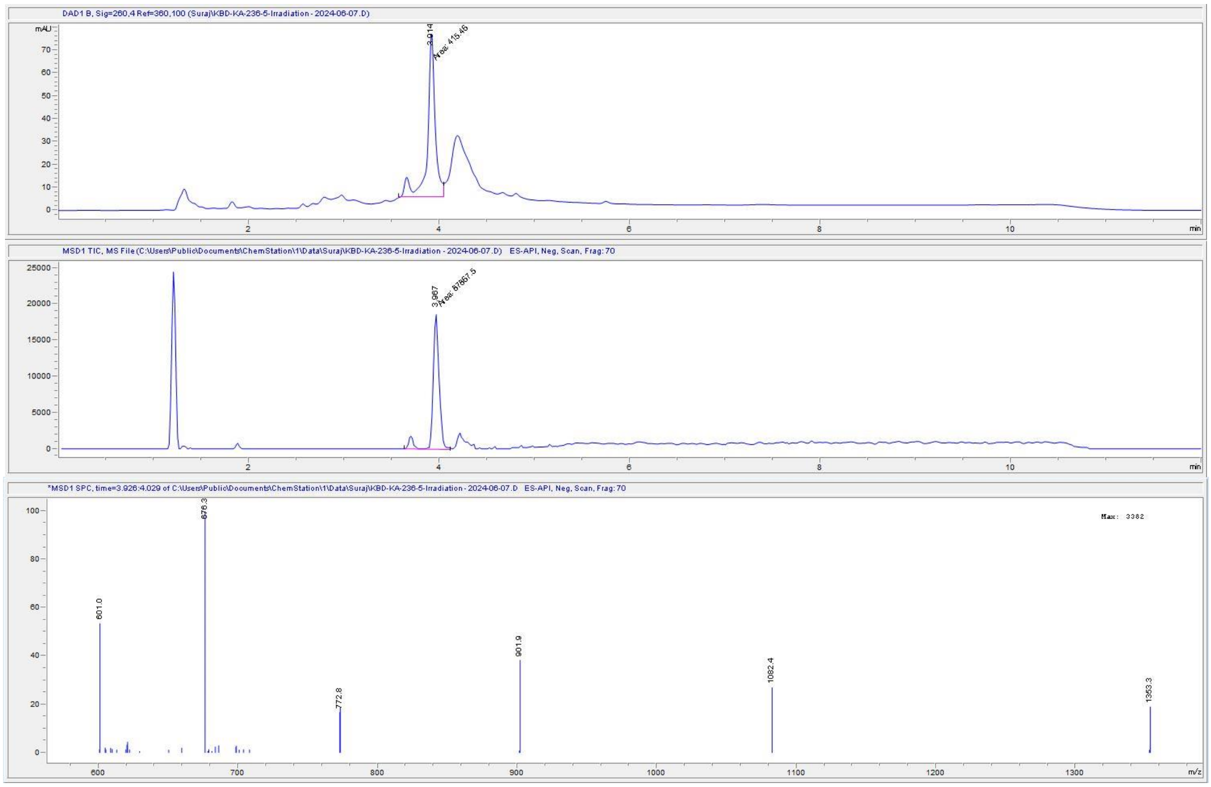

Figure S114. Analytical HPLC trace of **59** with HPLC method A. (Top) DAD chromatogram at 260 nm. (Middle) TIC chromatogram. (Bottom) Ionization of peak at 3.97 min. containing reaction product (peak at ~3.70 min corresponds to the Ru-arene complexed product **SRu-59'**, which is considered in yield determination).

Synthesis of DNA-conjugate 60

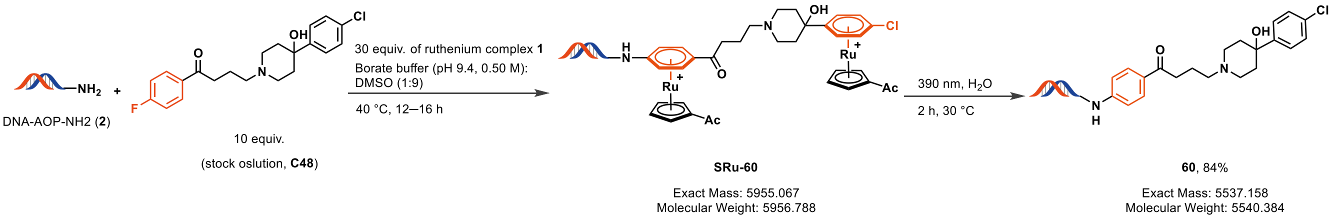

In situ formation of arene-ruthenium stock solution: Under an ambient atmosphere, a 1 mL glass GC vial equipped with a 6 mm Teflon-coated stirring bar was charged with ruthenium complex **1** (2.0 mg, 4.2 µmol, 3.0 equiv.), followed by 70 µL DMC. Next, a stock solution of Haloperidol DMC (70 µL, c = 0.020 M, 1.0 equiv.) was added to the glass vial. The vial was closed with a screw cap. The resulting reaction mixture was heated at 80 °C for 2 hours. After 2 hours, the reaction mixture was cooled to 23 °C and the DMC was removed under a gentle stream of argon and 140 µL of DMSO were added to result an in situ formed stock solution of arene-ruthenium complex **C48** (140 µL, c = 0.01 M, assuming quantitative arene coordination to ruthenium).

Under an ambient atmosphere, the stock solution **SD-01** of DNA-AOP-NH_2 (**2**) in water (1.0 µL, c = 2.0 mM,

2.0 nmol, 1.0 equiv.) was added to a 1.5 mL Eppendorf tube, followed by sodium borate buffer (1.0 µL, pH 9.4, c = 0.50 M). To this mixture, 16 µL of DMSO was added and the solution was vortexed for 5 seconds. Next, the freshly prepared stock solution **C48** (2.0 µL, c = 0.01 M, 0.02 µmol, 10 equiv.) in DMSO was added. The resulting reaction mixture was vortexed for 5 seconds, transferred to a thermocycler at 40 °C, and incubated for 16 hours at 800 rpm to yield the DNA-conjugate **SRu-60**. Next, the reaction mixture was diluted with 10 µL of Milli-Q water. To the reaction mixture was added the stock solution of NaCl in water (**SR-06** , 3.0 µL, c = 5.0 M, 10% volume of the total reaction volume), followed by cold ethanol (−20 °C, 99 µL) to precipitate the *N*-arylated ruthenium DNA conjugate **SRu-60**. The Eppendorf tube was placed in a freezer (−20 °C) for at least 1 hour, and then it was centrifuged at 4 °C and 11000 × g for at least 30 minutes. The supernatant was removed and the pellet was dried under air, then dissolved in 20 µL water to obtain the DNA-conjugate **SRu-60** (20 µL, c = 0.10 mM). Then, 1.0 µL of the above solution was diluted to 40 µL with water for LC–MS analysis.

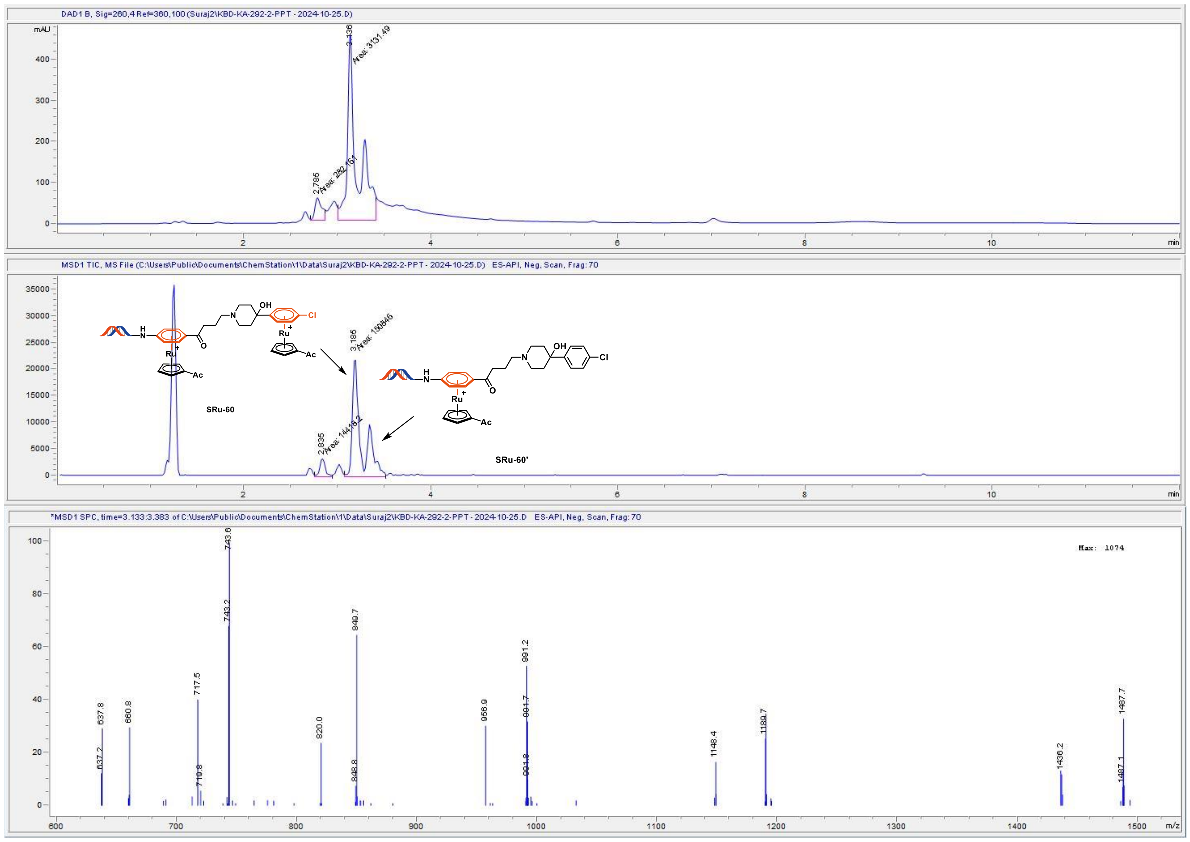

Figure S115. Analytical HPLC trace of **SRu-60** and **SRu-60’** with HPLC method A. (Top) DAD chromatogram at 260 nm. (Middle) TIC chromatogram. (Bottom) Ionization of peak at 3.18**–**3.50 min. containing DNA-conjugate **SRu-60** and **SRu-60’**.

Decomplexation of SRu-60 and SRu-60’ to obtain product 60: Under an ambient atmosphere, the solution containing the mixture of DNA-conjugate **SRu-60** and **SRu-60’** stock solution in water (c = 0.10 mM, 20 µL) was irradiated with a 390 nm (40 W) Kessil lamp for 2 hours, while maintaining the temperature at

approximately 30 °C through cooling with a fan. To the reaction mixture was added the stock solution of NaCl in water (**SR-06**, 2.0 µL, c = 5.0 M, 10% volume of the total reaction volume), followed by cold ethanol (−20 °C, 66 µL) to precipitate the DNA conjugate **60**. The Eppendorf tube was placed in the freezer (−20 °C) for at least 1 hour, and then it was centrifuged at 4 °C and 11000 × g for at least 30 minutes. The supernatant was removed, the pellet dried under air and dissolved in Milli-Q water to obtain the purified DNA-conjugate **60**. Then, 1 µL of the above solution was diluted to 40 µL with water for LC–MS analysis. The yield of the DNA conjugate was calculated by measuring the integration of the peaks of the diode array detection (DAD) UV absorbance at 260 nm of the LC–MS trace, assuming complete DNA recovery and identical UV absorbance.

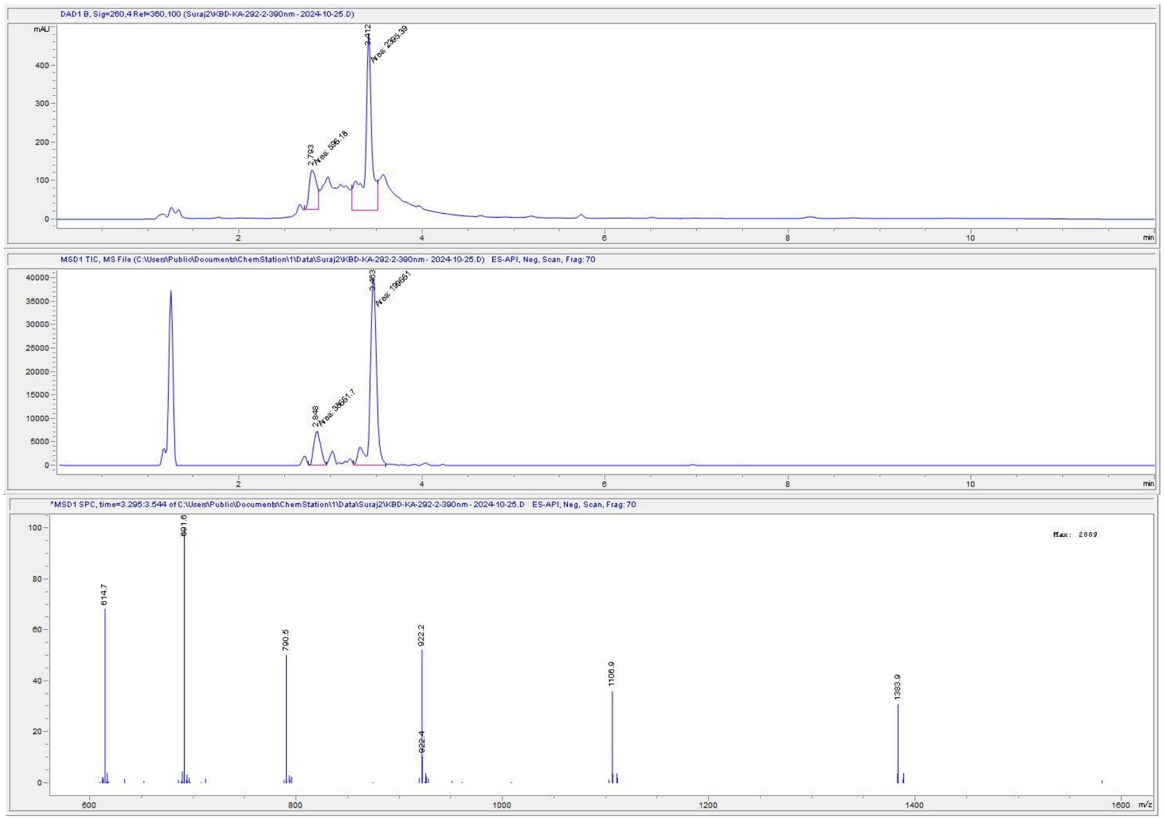

Figure S116. Analytical HPLC trace of **60** with HPLC method A. (Top) DAD chromatogram at 260 nm. (Middle) TIC chromatogram. (Bottom) Ionization of peak at 3.46 min. containing reaction product (yield was determined by integrating the TIC chromatogram).

N-arylation of amine-DNA conjugates

In situ formation of fluorobenzene-ruthenium stock solution:

Under an ambient atmosphere, a 1 mL glass GC vial equipped with a 6 mm Teflon-coated stirring bar was charged with ruthenium complex **1** (1.4 mg, 2.9 µmol, 1.0 equiv.). Next, a stock solution of fluorobenzene in DMC (294 µL, c = 0.10 M, 29 µmol, 10 equiv.) was added. The resulting reaction mixture was heated at 80 °C for 2 hours. After 2 hours, the reaction mixture was cooled to 23 °C. Next, the DMC was removed under a

gentle stream of argon and 294 µL of DMSO were added to result an in situ formed stock solution of arene-ruthenium complex **C1** (294 µL, c = 0.01 M, assuming quantitative arene coordination to ruthenium). The stock solution **C1** was used for performing *N*-arylation of all the amine DNA-conjugates (**S15**–**S30**).

N-arylation of DNA-conjugate S15

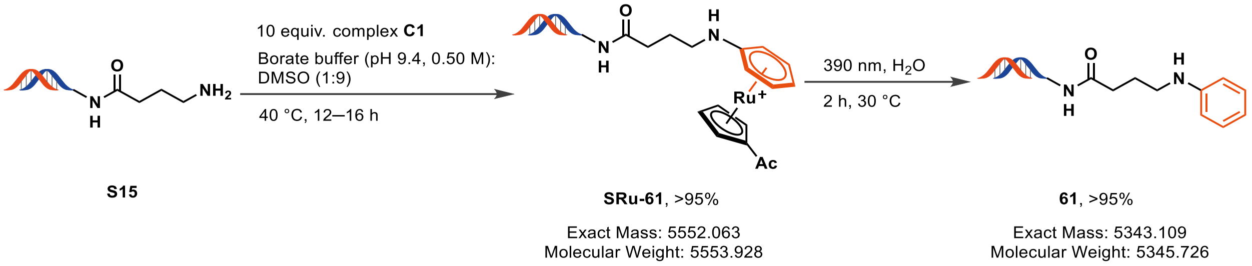

At 20–25 °C, the stock solution of DNA-conjugate **S15** (1.0 µL, c = 2.0 mM, 2.0 nmol, 1.0 equiv.) in water was added to a 1.5 mL Eppendorf tube, followed by sodium borate buffer (1.0 µL, pH 9.4, c = 0.50 M). To this mixture, 16 µL of DMSO was added and the solution was vortexed for 5 seconds. Next, the freshly prepared stock solution **C1** (2.0 µL, c = 0.01 M, 0.02 µmol, 10 equiv.) in DMSO was added. The resulting reaction mixture was vortexed for 5 seconds, transferred to a thermocycler at 40 °C, and incubated for 16 hours at 800 rpm to yield the DNA-conjugate **SRu-61**. Next, the reaction mixture was diluted with 10 µL of Milli-Q water. To the reaction mixture was added the stock solution of NaCl in water (**SR-06** , 3.0 µL, c = 5.0 M, 10% volume of the total reaction volume), followed by cold ethanol (−20 °C, 99 µL) to precipitate the *N*-arylated ruthenium DNA conjugate **SRu-61**. The Eppendorf tube was placed in a freezer (−20 °C) for at least 1 hour, and then it was centrifuged at 4 °C and 11000 × g for at least 30 minutes. The supernatant was removed and the pellet was dried under air, then dissolved in 20 µL water to obtain the DNA-conjugate **SRu-61** (20 µL, c = 0.10 mM). Then, 1.0 µL of the above solution was diluted to 40 µL with water for LC–MS analysis.

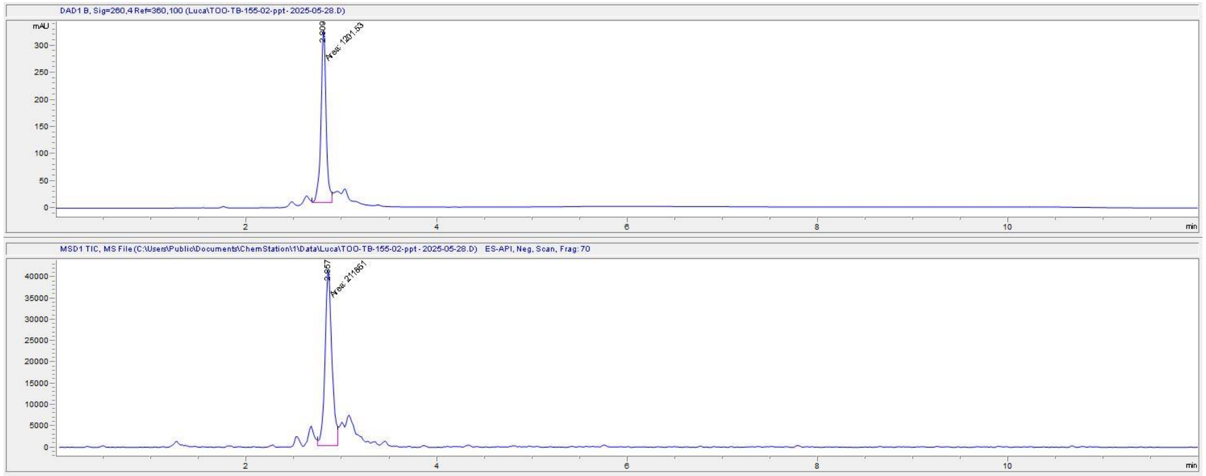

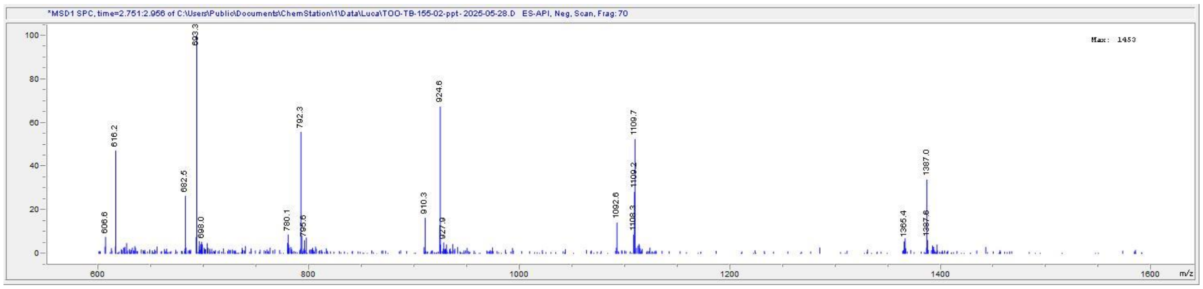

Figure S117. Analytical HPLC trace of **SRu-61** with HPLC method A. (Top) DAD chromatogram at 260 nm. (Middle) TIC chromatogram. (Bottom) Ionization of peak at 2.86 min. containing reaction product.

Decomplexation of SRu-61 to obtain product 61: Under an ambient atmosphere, the DNA-conjugate **SRu-61** stock solution in water (c = 0.10 mM, 20 µL) was irradiated with a 390 nm (40 W) Kessil lamp for 2 hours, while maintaining the temperature at approximately 30 °C through cooling with a fan. To the reaction mixture was added the stock solution of NaCl in water (**SR-06**, 2.0 µL, c = 5.0 M, 10% volume of the total reaction volume), followed by cold ethanol (−20 °C, 66 µL) to precipitate the DNA conjugate **61**. The Eppendorf tube was placed in the freezer (−20 °C) for at least 1 hour, and then it was centrifuged at 4 °C and 11000 × g for at least 30 minutes. The supernatant was removed, the pellet dried under air and dissolved in Milli-Q water to obtain the purified DNA-conjugate **61**. Then, 1 µL of the above solution was diluted to 40 µL with water for LC–MS analysis. The yield of the DNA conjugate was calculated by measuring the integration of the peaks of the diode array detection (DAD) UV absorbance at 260 nm of the LC–MS trace, assuming complete DNA recovery and identical UV absorbance. In this case the unreacted DNA-AOP-NH_2 (**2**) from the starting material also gave the arylated product. Therefore, the yield was determined by the integration of the DAD peak, where the unreacted DNA-conjugate **2** from the starting material was not included in yield determination.

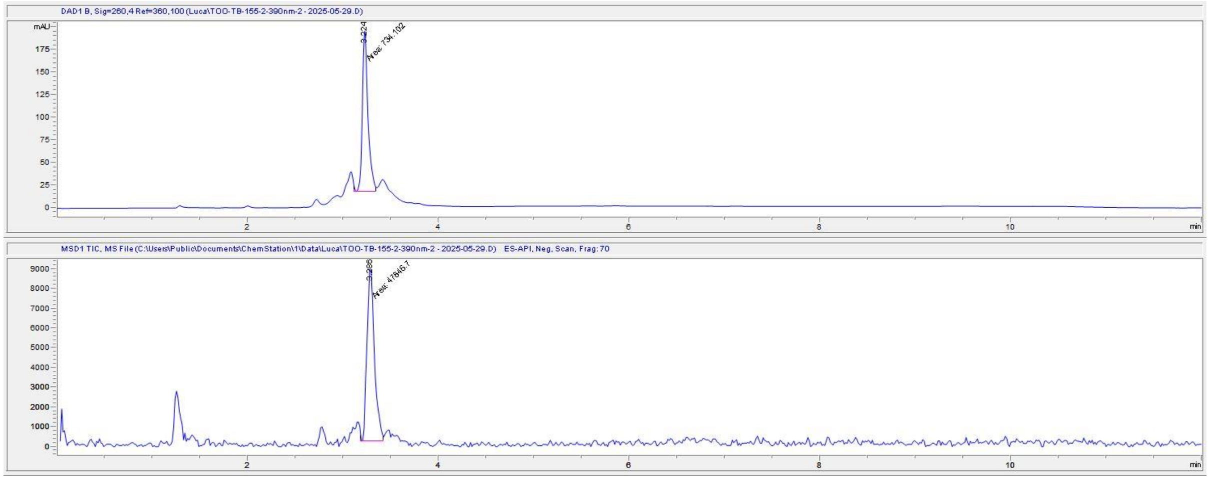

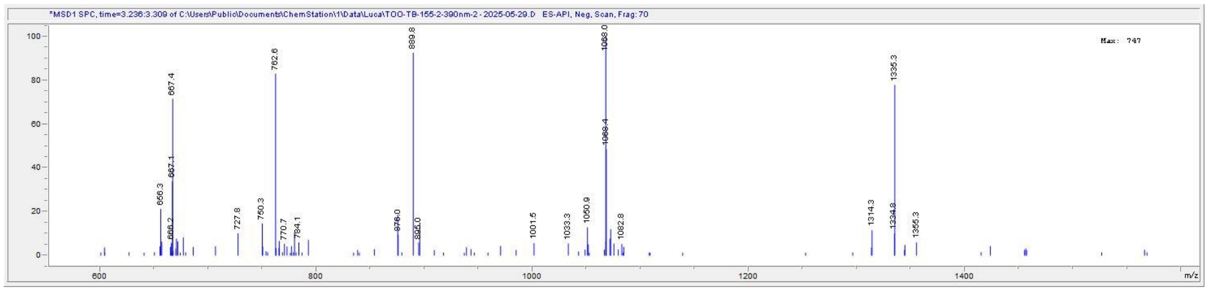

Figure S118. Analytical HPLC trace of **61** with HPLC method A. (Top) DAD chromatogram at 260 nm. (Middle) TIC chromatogram. (Bottom) Ionization of peak at 3.29 min. containing reaction product.

N-arylation of DNA-conjugate S16

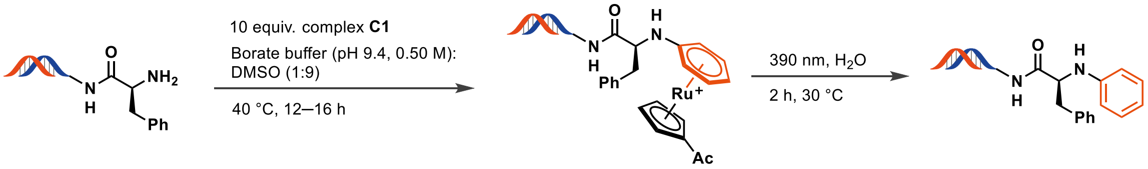

S16 **SRu-62** **62**, 66%

Exact Mass: 5614.078
Molecular Weight: 5615.999

Exact Mass: 5405.124
Molecular Weight: 5407.797

At 20–25 °C, the stock solution of DNA-conjugate **S16** (1.0 µL, c = 2.0 mM, 2.0 nmol, 1.0 equiv.) in water was added to a 1.5 mL Eppendorf tube, followed by sodium borate buffer (1.0 µL, pH 9.4, c = 0.50 M). To this mixture, 16 µL of DMSO was added and the solution was vortexed for 5 seconds. Next, the freshly prepared stock solution **C1** (2.0 µL, c = 0.01 M, 0.02 µmol, 10 equiv.) in DMSO was added. The resulting reaction mixture was vortexed for 5 seconds, transferred to a thermocycler at 40 °C, and incubated for 16 hours at 800 rpm to yield the DNA-conjugate **SRu-62**. Next, the reaction mixture was diluted with 10 µL of Milli-Q water. To the reaction mixture was added the stock solution of NaCl in water (**SR-06** , 3.0 µL, c = 5.0 M, 10% volume of the total reaction volume), followed by cold ethanol (−20 °C, 99 µL) to precipitate the *N*-arylated ruthenium DNA conjugate **SRu-62**. The Eppendorf tube was placed in a freezer (−20 °C) for at least 1 hour, and then it was centrifuged at 4 °C and 11000 × g for at least 30 minutes. The supernatant was removed and the pellet was dried under air, then dissolved in 20 µL water to obtain the DNA-conjugate **SRu-62** (20 µL, c = 0.10 mM). Then, 1.0 µL of the above solution was diluted to 40 µL with water for LC–MS analysis.

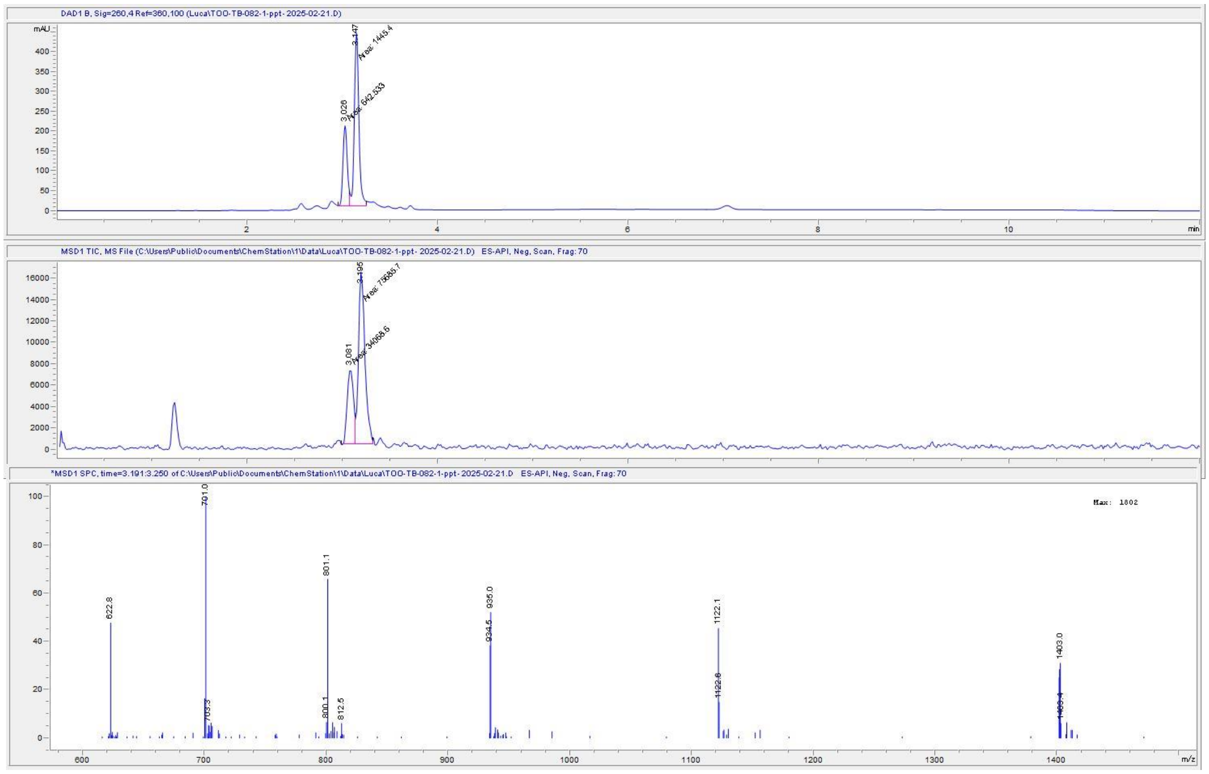

Figure S119. Analytical HPLC trace of **SRu-62** with HPLC method A. (Top) DAD chromatogram at 260 nm. (Middle) TIC chromatogram. (Bottom) Ionization of peak at 3.19 min. containing reaction product.

Decomplexation of SRu-62 to obtain product 62: Under an ambient atmosphere, the DNA-conjugate **SRu-62** stock solution in water (c = 0.10 mM, 20 µL) was irradiated with a 390 nm (40 W) Kessil lamp for 2 hours, while maintaining the temperature at approximately 30 °C through cooling with a fan. To the reaction mixture was added the stock solution of NaCl in water (**SR-06**, 2.0 µL, c = 5.0 M, 10% volume of the total reaction volume), followed by cold ethanol (−20 °C, 66 µL) to precipitate the DNA conjugate **62**. The Eppendorf tube was placed in the freezer (−20 °C) for at least 1 hour, and then it was centrifuged at 4 °C and 11000 × g for at least 30 minutes. The supernatant was removed, the pellet dried under air and dissolved in Milli-Q water to obtain the purified DNA-conjugate **62**. Then, 1 µL of the above solution was diluted to 40 µL with water for LC–MS analysis. The yield of the DNA conjugate was calculated by measuring the integration of the peaks of the diode array detection (DAD) UV absorbance at 260 nm of the LC–MS trace, assuming complete DNA recovery and identical UV absorbance.

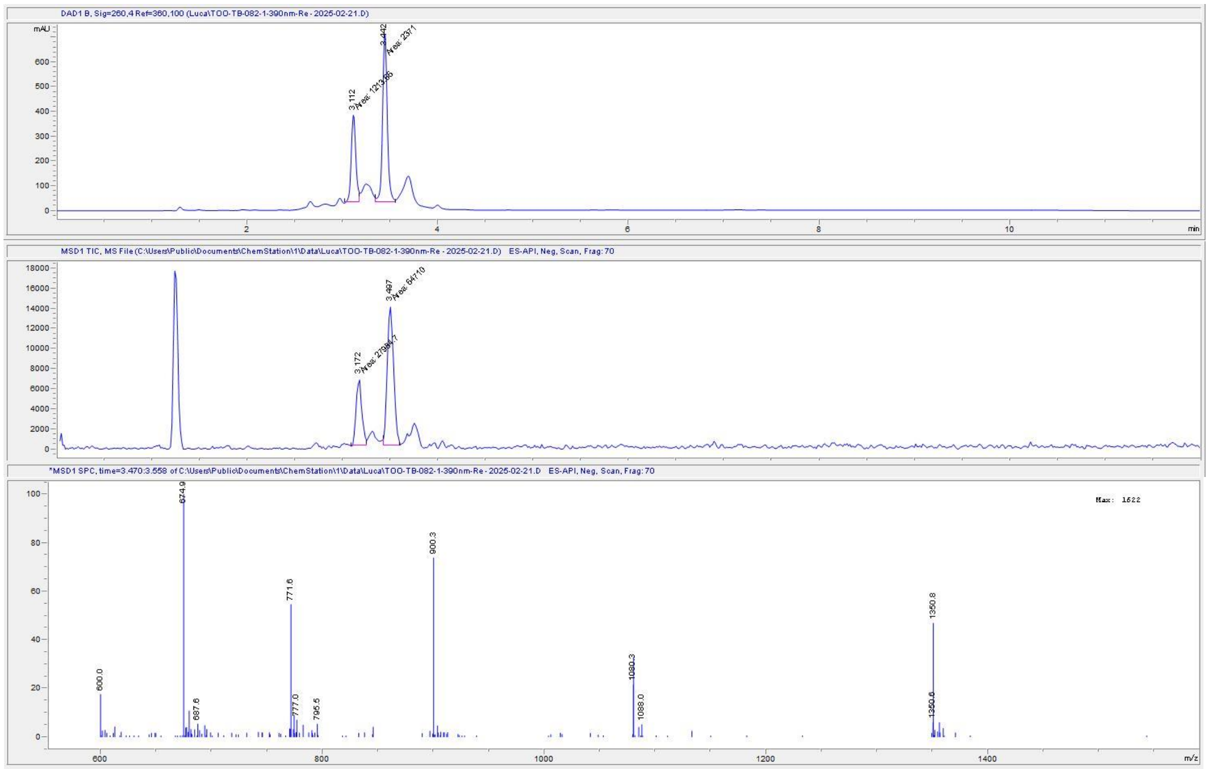

Figure S120. Analytical HPLC trace of **62** with HPLC method A. (Top) DAD chromatogram at 260 nm. (Middle) TIC chromatogram. (Bottom) Ionization of peak at 3.50 min. containing reaction product.

N-arylation of DNA-conjugate S17

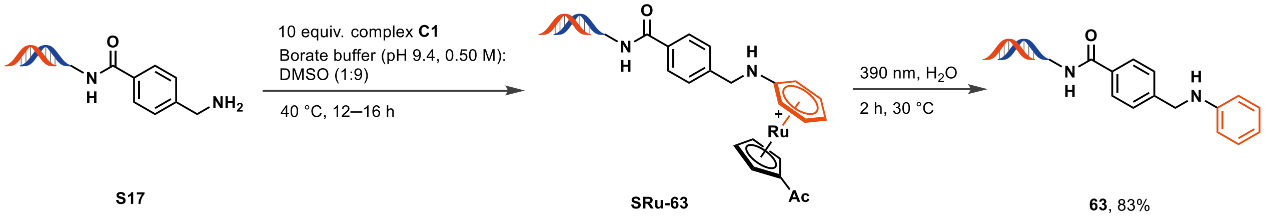

S17

SRu-63

Exact Mass: 5600.063
Molecular Weight: 5601.972

63, 83%

Exact Mass: 5391.109
Molecular Weight: 5393.770

At 20–25 °C, the stock solution of DNA-conjugate **S17** (1.0 µL, c = 2.0 mM, 2.0 nmol, 1.0 equiv.) in water was added to a 1.5 mL Eppendorf tube, followed by sodium borate buffer (1.0 µL, pH 9.4, c = 0.50 M). To this mixture, 16 µL of DMSO was added and the solution was vortexed for 5 seconds. Next, the freshly prepared stock solution **C1** (2.0 µL, c = 0.01 M, 0.02 µmol, 10 equiv.) in DMSO was added. The resulting reaction mixture was vortexed for 5 seconds, transferred to a thermocycler at 40 °C, and incubated for 16 hours at 800 rpm to yield the DNA-conjugate **SRu-63**. Next, the reaction mixture was diluted with 10 µL of Milli-Q water. To the reaction mixture was added the stock solution of NaCl in water (**SR-06** , 3.0 µL, c = 5.0 M, 10% volume of the total reaction volume), followed by cold ethanol (−20 °C, 99 µL) to precipitate the *N*-arylated ruthenium DNA conjugate **SRu-63**. The Eppendorf tube was placed in a freezer (−20 °C) for at least 1 hour, and then it was centrifuged at 4 °C and 11000 × g for at least 30 minutes. The supernatant was removed and

the pellet was dried under air, then dissolved in 20 µL water to obtain the DNA-conjugate **SRu-63** (20 µL, c = 0.10 mM). Then, 1.0 µL of the above solution was diluted to 40 µL with water for LC–MS analysis.

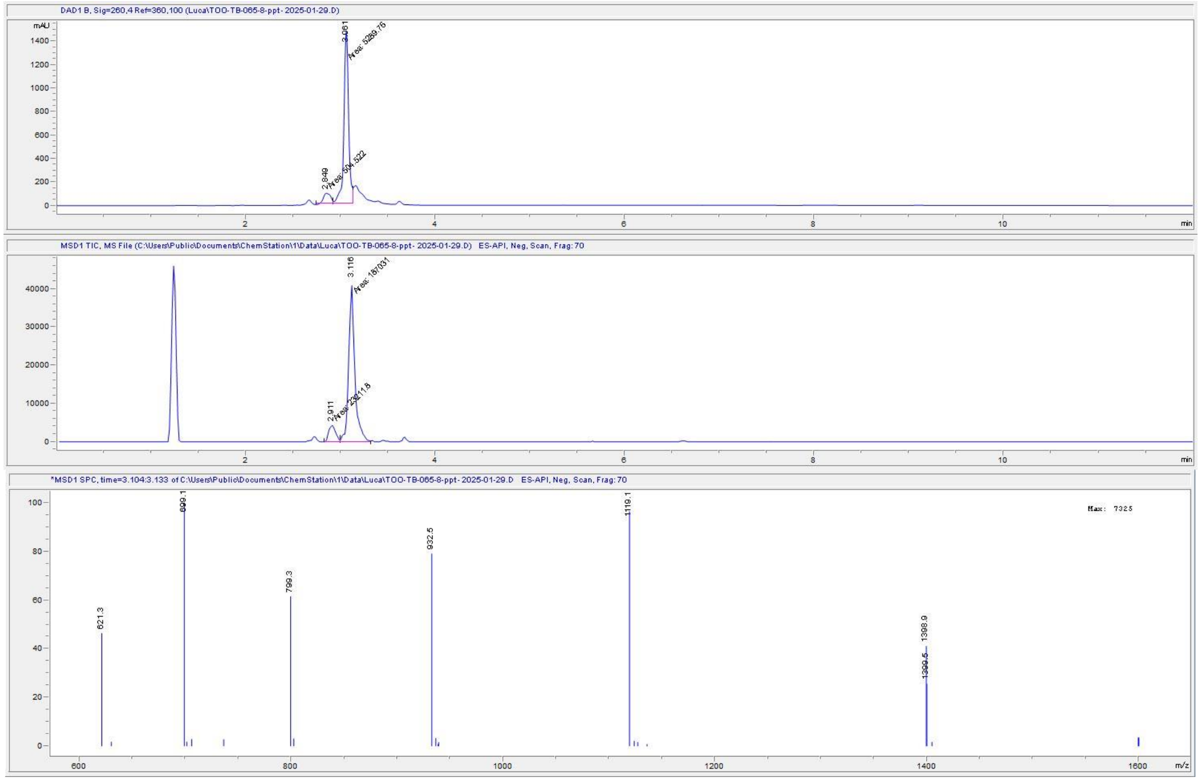

Figure S121. Analytical HPLC trace of **SRu-63** with HPLC method A. (Top) DAD chromatogram at 260 nm. (Middle) TIC chromatogram. (Bottom) Ionization of peak at 3.12 min. containing reaction product.

Decomplexation of SRu-63 to obtain product 63: Under an ambient atmosphere, the DNA-conjugate **SRu-63** stock solution in water (c = 0.10 mM, 20 µL) was irradiated with a 390 nm (40 W) Kessil lamp for 2 hours, while maintaining the temperature at approximately 30 °C through cooling with a fan. To the reaction mixture was added the stock solution of NaCl in water (**SR-06**, 2.0 µL, c = 5.0 M, 10% volume of the total reaction volume), followed by cold ethanol (−20 °C, 66 µL) to precipitate the DNA conjugate **63**. The Eppendorf tube was placed in the freezer (−20 °C) for at least 1 hour, and then it was centrifuged at 4 °C and 11000 × g for at least 30 minutes. The supernatant was removed, the pellet dried under air and dissolved in Milli-Q water to obtain the purified DNA-conjugate **63**. Then, 1 µL of the above solution was diluted to 40 µL with water for LC–MS analysis. The yield of the DNA conjugate was calculated by measuring the integration of the peaks of the diode array detection (DAD) UV absorbance at 260 nm of the LC–MS trace, assuming complete DNA recovery and identical UV absorbance.

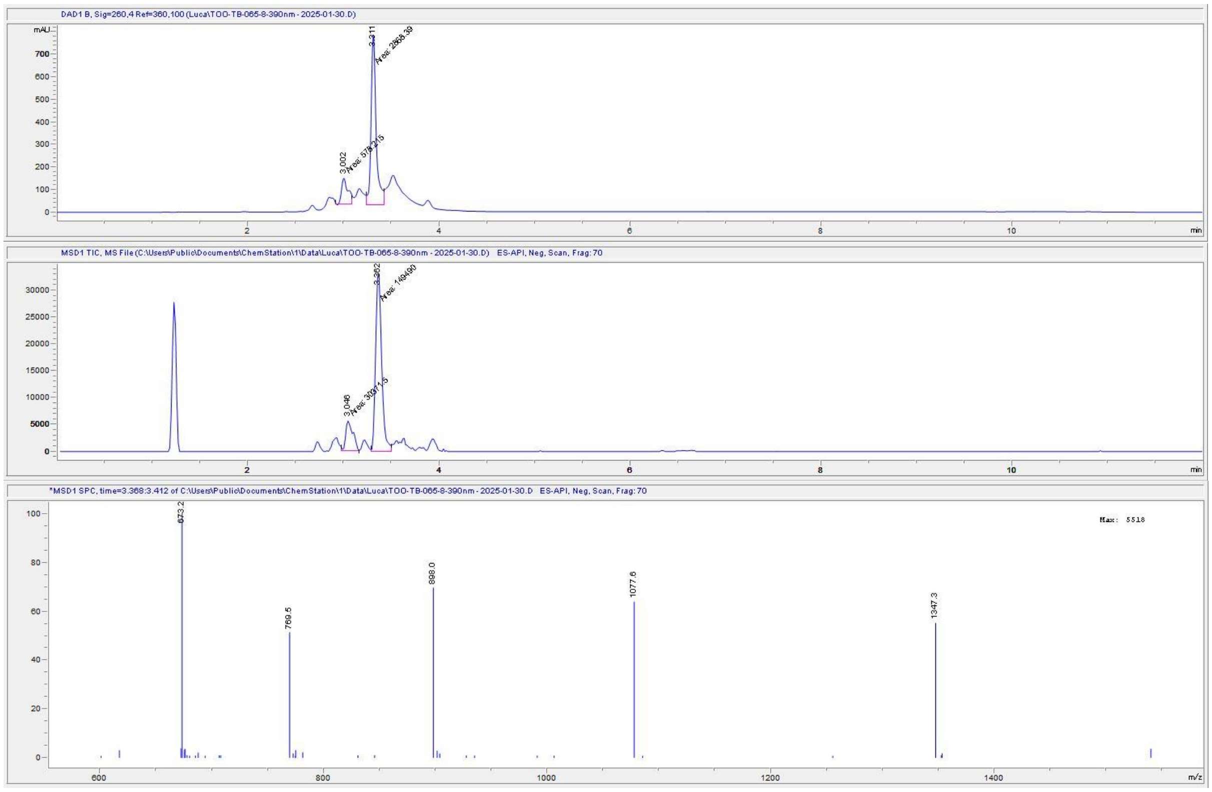

Figure S122. Analytical HPLC trace of **63** with HPLC method A. (Top) DAD chromatogram at 260 nm. (Middle) TIC chromatogram. (Bottom) Ionization of peak at 3.36 min. containing reaction product.

N-arylation of DNA-conjugate S18

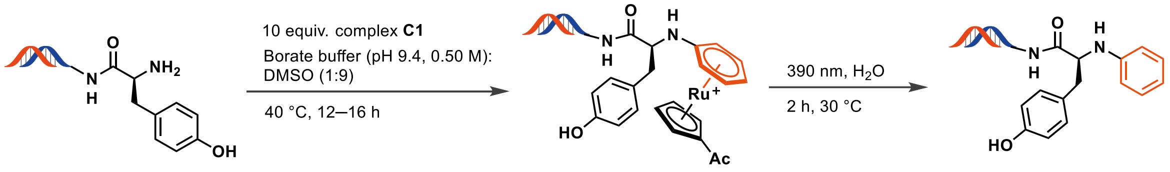

S18 **SRu-64** **64**, >95%

Exact Mass: 5630.073
Molecular Weight: 5631.007

Exact Mass: 5421.119
Molecular Weight: 5423.796

At 20–25 °C, the stock solution of DNA-conjugate **S18** (1.0 µL, c = 2.0 mM, 2.0 nmol, 1.0 equiv.) in water was added to a 1.5 mL Eppendorf tube, followed by sodium borate buffer (1.0 µL, pH 9.4, c = 0.50 M). To this mixture, 16 µL of DMSO was added and the solution was vortexed for 5 seconds. Next, the freshly prepared stock solution **C1** (2.0 µL, c = 0.01 M, 0.02 µmol, 10 equiv.) in DMSO was added. The resulting reaction mixture was vortexed for 5 seconds, transferred to a thermocycler at 40 °C, and incubated for 16 hours at 800 rpm to yield the DNA-conjugate **SRu-64**. Next, the reaction mixture was diluted with 10 µL of Milli-Q water. To the reaction mixture was added the stock solution of NaCl in water (**SR-06** , 3.0 µL, c = 5.0 M, 10% volume of the total reaction volume), followed by cold ethanol (−20 °C, 99 µL) to precipitate the *N*-arylated ruthenium DNA conjugate **SRu-64**. The Eppendorf tube was placed in a freezer (−20 °C) for at least 1 hour, and then it was centrifuged at 4 °C and 11000 × g for at least 30 minutes. The supernatant was removed and

the pellet was dried under air, then dissolved in 20 µL water to obtain the DNA-conjugate **SRu-64** (20 µL, c = 0.10 mM). Then, 1.0 µL of the above solution was diluted to 40 µL with water for LC–MS analysis.

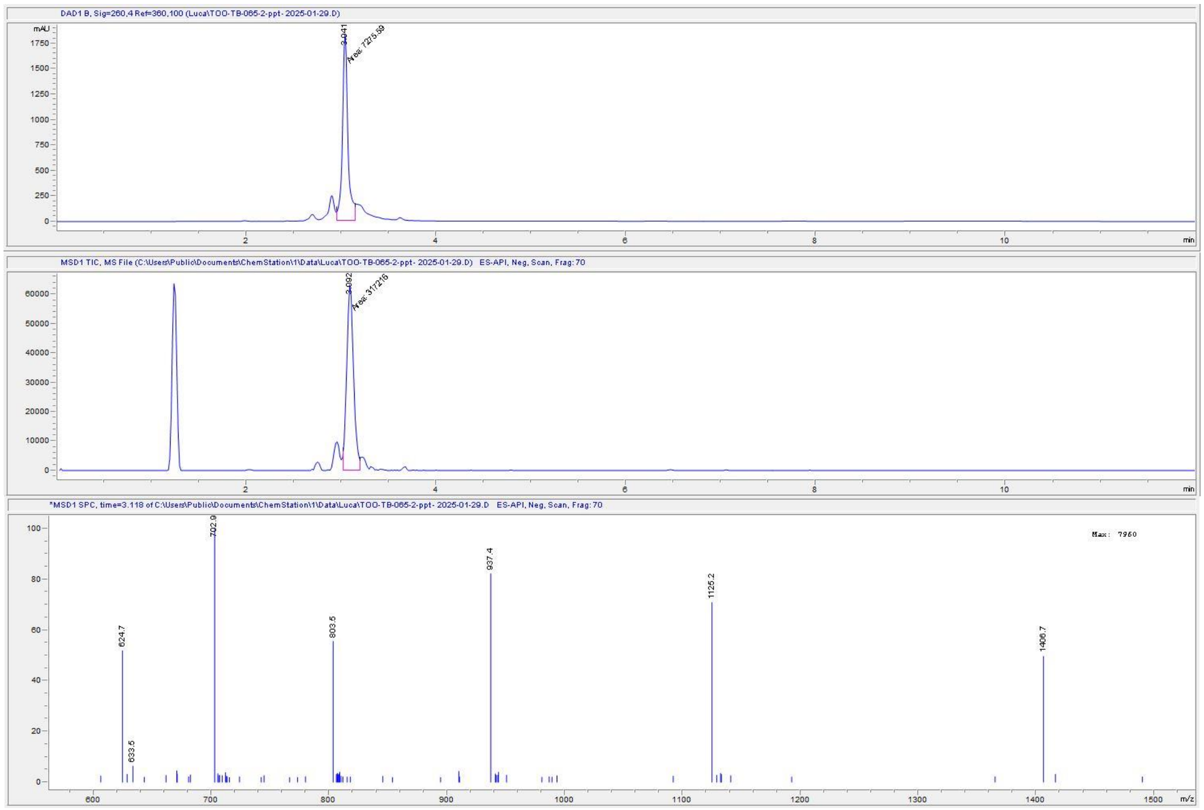

Figure S123. Analytical HPLC trace of **SRu-64** with HPLC method A. (Top) DAD chromatogram at 260 nm. (Middle) TIC chromatogram. (Bottom) Ionization of peak at 3.09 min. containing reaction product.

Decomplexation of SRu-64 to obtain product 64: Under an ambient atmosphere, the DNA-conjugate **SRu-64** stock solution in water (c = 0.10 mM, 20 µL) was irradiated with a 390 nm (40 W) Kessil lamp for 2 hours, while maintaining the temperature at approximately 30 °C through cooling with a fan. To the reaction mixture was added the stock solution of NaCl in water (**SR-06**, 2.0 µL, c = 5.0 M, 10% volume of the total reaction volume), followed by cold ethanol (−20 °C, 66 µL) to precipitate the DNA conjugate **64**. The Eppendorf tube was placed in the freezer (−20 °C) for at least 1 hour, and then it was centrifuged at 4 °C and 11000 × g for at least 30 minutes. The supernatant was removed, the pellet dried under air and dissolved in Milli-Q water to obtain the purified DNA-conjugate **64**. Then, 1 µL of the above solution was diluted to 40 µL with water for LC–MS analysis. The yield of the DNA conjugate was calculated by measuring the integration of the peaks of the diode array detection (DAD) UV absorbance at 260 nm of the LC–MS trace, assuming complete DNA recovery and identical UV absorbance.

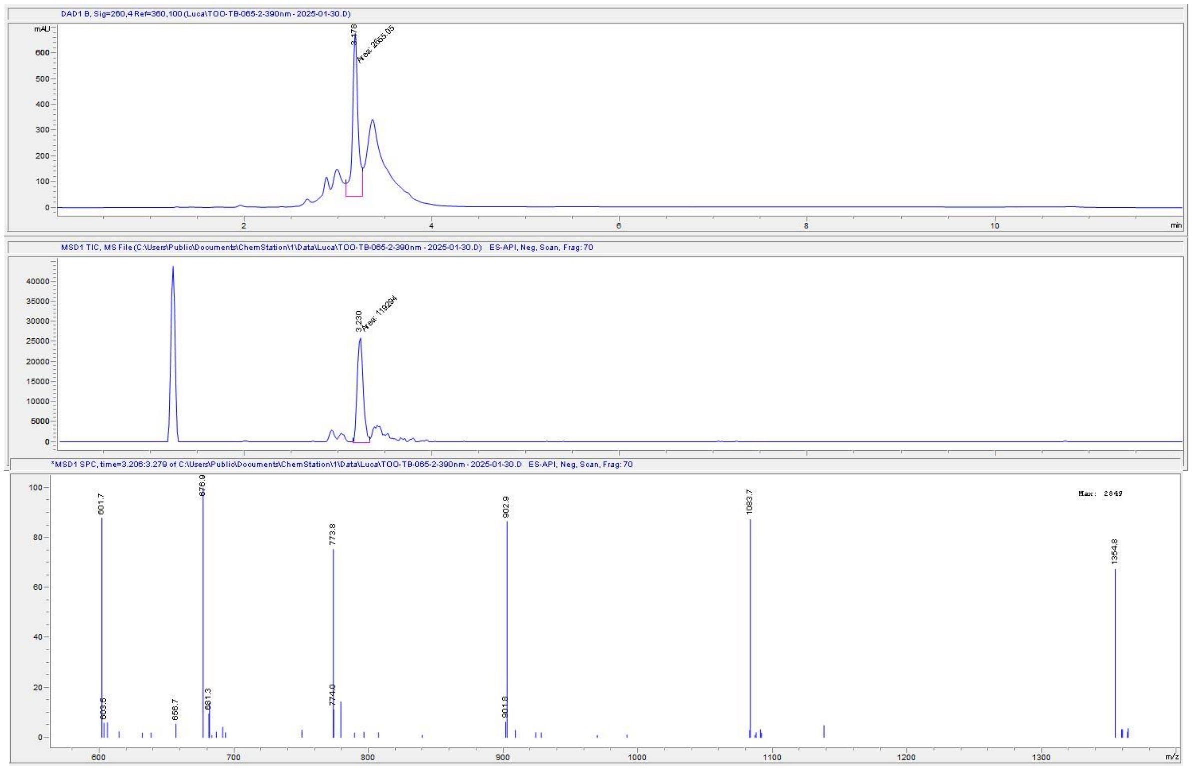

Figure S124. Analytical HPLC trace of **64** with HPLC method A. (Top) DAD chromatogram at 260 nm. (Middle) TIC chromatogram. (Bottom) Ionization of peak at 3.23 min. containing reaction product.

N-arylation of DNA-conjugate S20

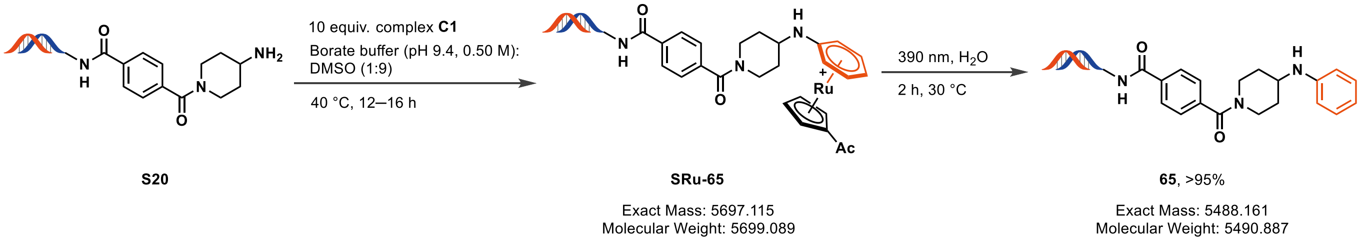

At 20–25 °C, the stock solution of DNA-conjugate **S20** (1.0 µL, c = 2.0 mM, 2.0 nmol, 1.0 equiv.) in water was added to a 1.5 mL Eppendorf tube, followed by sodium borate buffer (1.0 µL, pH 9.4, c = 0.50 M). To this mixture, 16 µL of DMSO was added and the solution was vortexed for 5 seconds. Next, the freshly prepared stock solution **C1** (2.0 µL, c = 0.01 M, 0.02 µmol, 10 equiv.) in DMSO was added. The resulting reaction mixture was vortexed for 5 seconds, transferred to a thermocycler at 40 °C, and incubated for 16 hours at 800 rpm to yield the DNA-conjugate **SRu-65**. Next, the reaction mixture was diluted with 10 µL of Milli-Q water. To the reaction mixture was added the stock solution of NaCl in water (**SR-06** , 3.0 µL, c = 5.0 M, 10% volume of the total reaction volume), followed by cold ethanol (−20 °C, 99 µL) to precipitate the *N*-arylated ruthenium DNA conjugate **SRu-65**. The Eppendorf tube was placed in a freezer (−20 °C) for at least 1 hour, and then it was centrifuged at 4 °C and 11000 × g for at least 30 minutes. The supernatant was removed and the pellet was dried under air, then dissolved in 20 µL water to obtain the DNA-conjugate **SRu-65** (20 µL, c =

0.10 mM). Then, 1.0 µL of the above solution was diluted to 40 µL with water for LC–MS analysis.

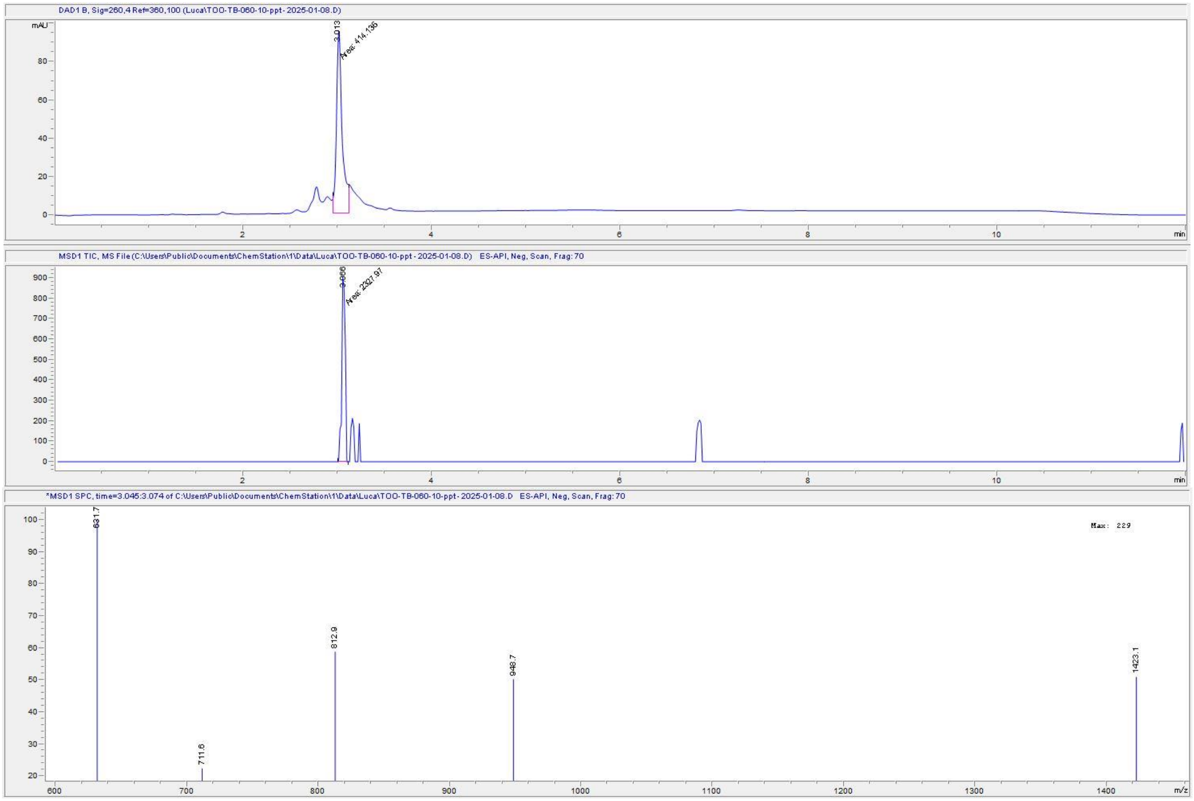

Figure S125. Analytical HPLC trace of **SRu-65** with HPLC method A. (Top) DAD chromatogram at 260 nm. (Middle) TIC chromatogram. (Bottom) Ionization of peak at 3.07 min. containing reaction product.

Decomplexation of SRu-65 to obtain product 65: Under an ambient atmosphere, the DNA-conjugate **SRu-65** stock solution in water (c = 0.10 mM, 20 µL) was irradiated with a 390 nm (40 W) Kessil lamp for 2 hours, while maintaining the temperature at approximately 30 °C through cooling with a fan. To the reaction mixture was added the stock solution of NaCl in water (**SR-06**, 2.0 µL, c = 5.0 M, 10% volume of the total reaction volume), followed by cold ethanol (−20 °C, 66 µL) to precipitate the DNA conjugate **65**. The Eppendorf tube was placed in the freezer (−20 °C) for at least 1 hour, and then it was centrifuged at 4 °C and 11000 × g for at least 30 minutes. The supernatant was removed, the pellet dried under air and dissolved in Milli-Q water to obtain the purified DNA-conjugate **65**. Then, 1 µL of the above solution was diluted to 40 µL with water for LC–MS analysis. The yield of the DNA conjugate was calculated by measuring the integration of the peaks of the diode array detection (DAD) UV absorbance at 260 nm of the LC–MS trace, assuming complete DNA recovery and identical UV absorbance.

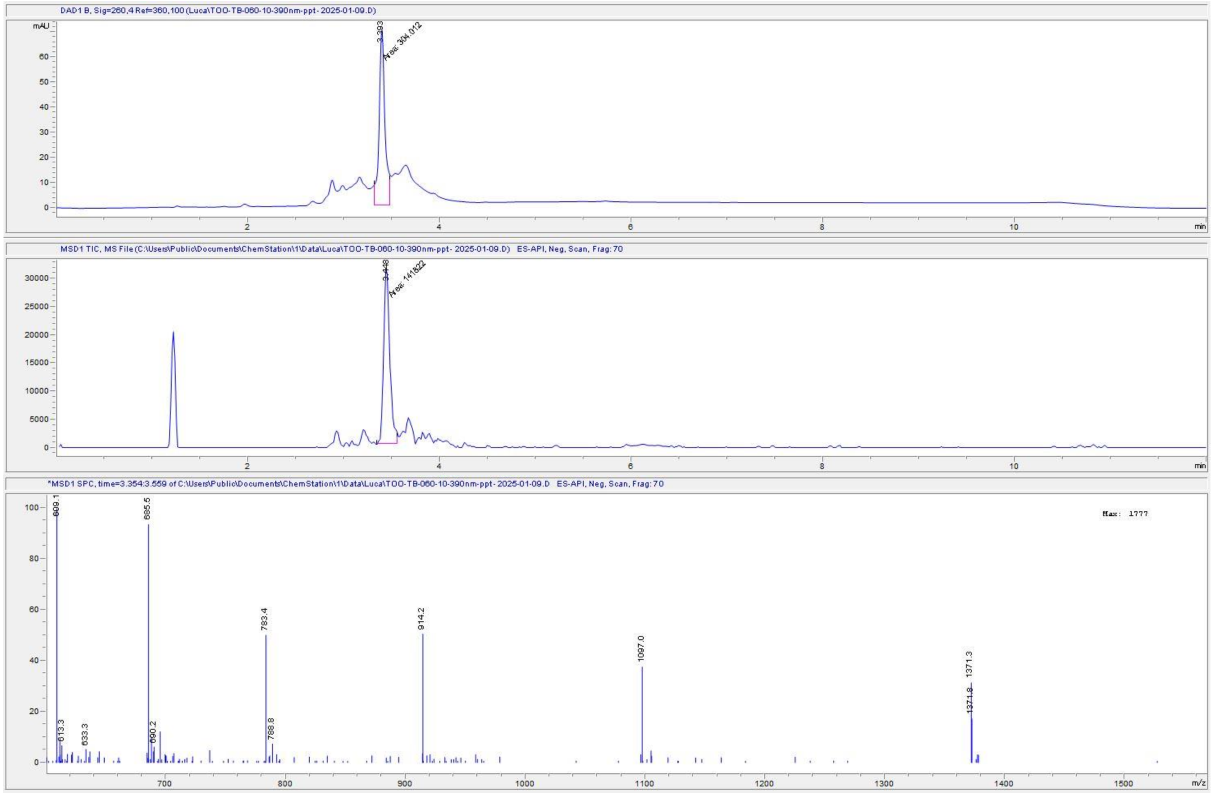

Figure S126. Analytical HPLC trace of **65** with HPLC method A. (Top) DAD chromatogram at 260 nm. (Middle) TIC chromatogram. (Bottom) Ionization of peak at 3.45 min. containing reaction product.

N-arylation of DNA-conjugate S21

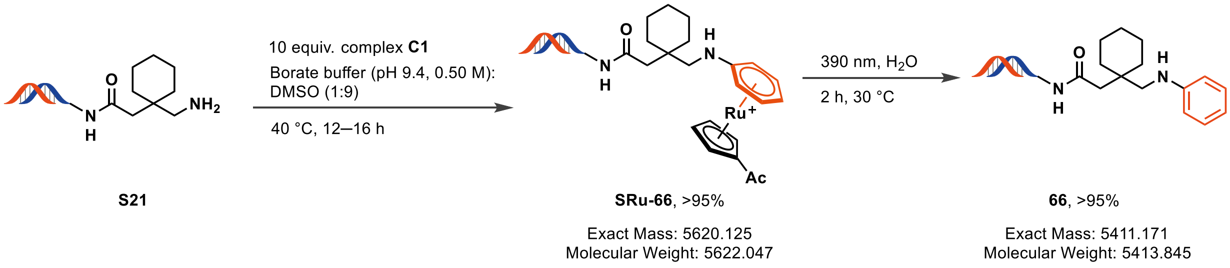

At 20–25 °C, the stock solution of DNA-conjugate **S21** (1.0 µL, c = 2.0 mM, 2.0 nmol, 1.0 equiv.) in water was added to a 1.5 mL Eppendorf tube, followed by sodium borate buffer (1.0 µL, pH 9.4, c = 0.50 M). To this mixture, 16 µL of DMSO was added and the solution was vortexed for 5 seconds. Next, the freshly prepared stock solution **C1** (2.0 µL, c = 0.01 M, 0.02 µmol, 10 equiv.) in DMSO was added. The resulting reaction mixture was vortexed for 5 seconds, transferred to a thermocycler at 40 °C, and incubated for 16 hours at 800 rpm to yield the DNA-conjugate **SRu-66**. Next, the reaction mixture was diluted with 10 µL of Milli-Q water. To the reaction mixture was added the stock solution of NaCl in water (**SR-06** , 3.0 µL, c = 5.0 M, 10% volume of the total reaction volume), followed by cold ethanol (−20 °C, 99 µL) to precipitate the *N*-arylated ruthenium DNA conjugate **SRu-66**. The Eppendorf tube was placed in a freezer (−20 °C) for at least 1 hour, and then it was centrifuged at 4 °C and 11000 × g for at least 30 minutes. The supernatant was removed and

the pellet was dried under air, then dissolved in 20 µL water to obtain the DNA-conjugate **SRu-66** (20 µL, c = 0.10 mM). Then, 1.0 µL of the above solution was diluted to 40 µL with water for LC–MS analysis.

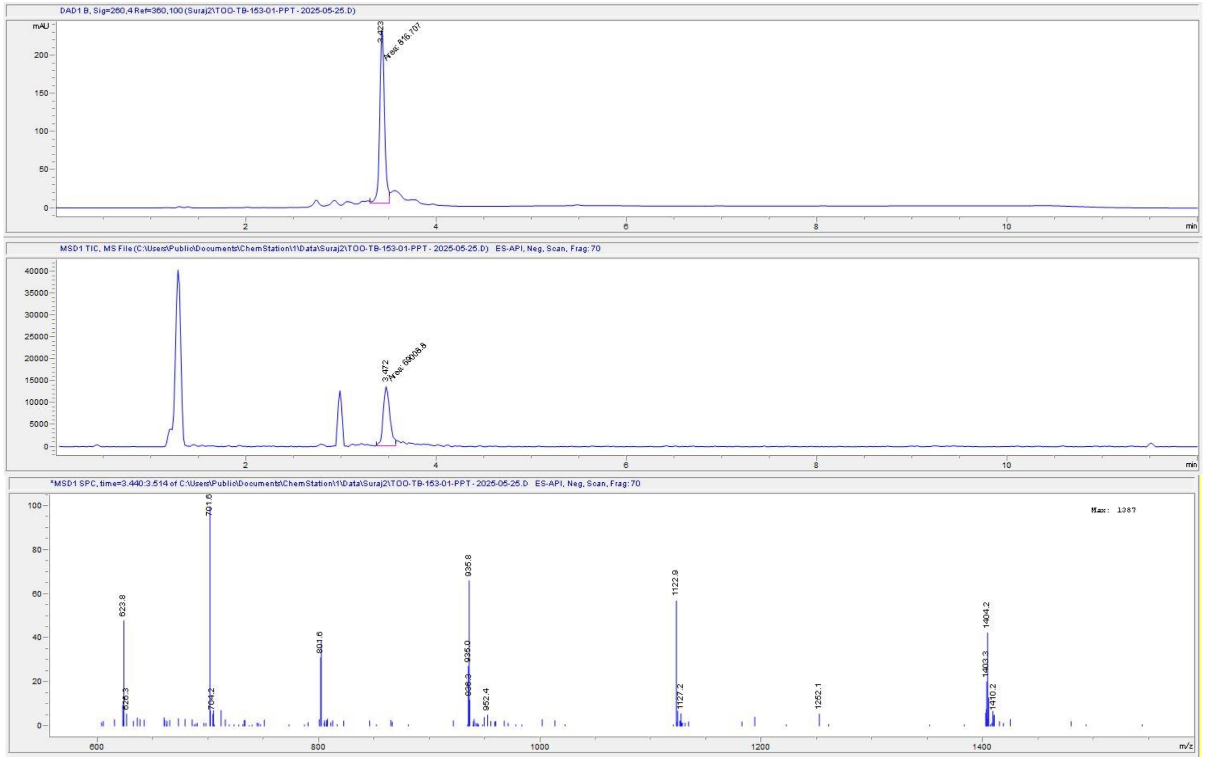

Figure S127. Analytical HPLC trace of **SRu-66** with HPLC method A. (Top) DAD chromatogram at 260 nm. (Middle) TIC chromatogram. (Bottom) Ionization of peak at 3.47 min. containing reaction product.

Decomplexation of SRu-66 to obtain product 66: Under an ambient atmosphere, the DNA-conjugate **SRu-66** stock solution in water (c = 0.10 mM, 20 µL) was irradiated with a 390 nm (40 W) Kessil lamp for 2 hours, while maintaining the temperature at approximately 30 °C through cooling with a fan. To the reaction mixture was added the stock solution of NaCl in water (**SR-06**, 2.0 µL, c = 5.0 M, 10% volume of the total reaction volume), followed by cold ethanol (−20 °C, 66 µL) to precipitate the DNA conjugate **66**. The Eppendorf tube was placed in the freezer (−20 °C) for at least 1 hour, and then it was centrifuged at 4 °C and 11000 × g for at least 30 minutes. The supernatant was removed, the pellet dried under air and dissolved in Milli-Q water to obtain the purified DNA-conjugate **66**. Then, 1 µL of the above solution was diluted to 40 µL with water for LC–MS analysis. The yield of the DNA conjugate was calculated by measuring the integration of the peaks of the diode array detection (DAD) UV absorbance at 260 nm of the LC–MS trace, assuming complete DNA recovery and identical UV absorbance.

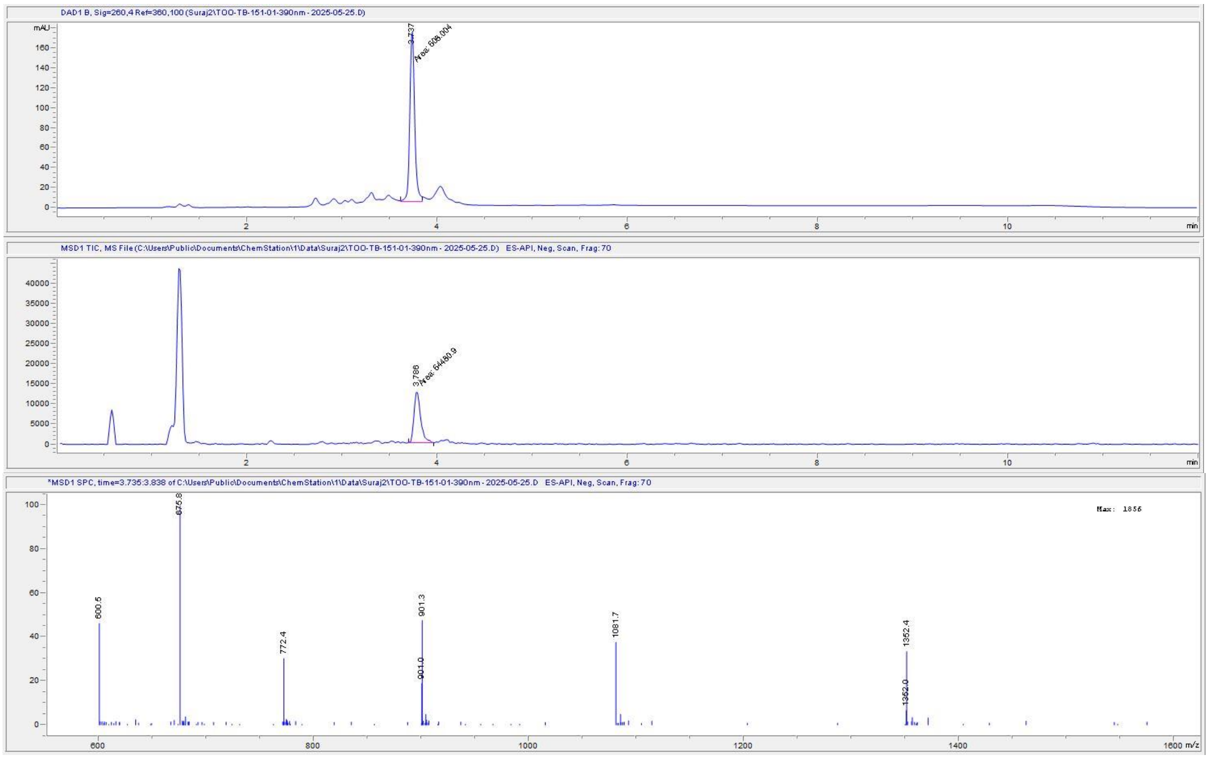

Figure S128. Analytical HPLC trace of **66** with HPLC method A. (Top) DAD chromatogram at 260 nm. (Middle) TIC chromatogram. (Bottom) Ionization of peak at 3.79 min. containing reaction product.

N-arylation of DNA-conjugate S22

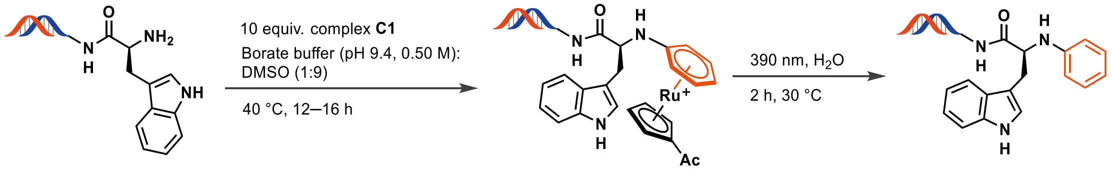

At 20–25 °C, the stock solution of DNA-conjugate **S22** (1.0 µL, c = 2.0 mM, 2.0 nmol, 1.0 equiv.) in water was added to a 1.5 mL Eppendorf tube, followed by sodium borate buffer (1.0 µL, pH 9.4, c = 0.50 M). To this mixture, 16 µL of DMSO was added and the solution was vortexed for 5 seconds. Next, the freshly prepared stock solution **C1** (2.0 µL, c = 0.01 M, 0.02 µmol, 10 equiv.) in DMSO was added. The resulting reaction mixture was vortexed for 5 seconds, transferred to a thermocycler at 40 °C, and incubated for 16 hours at 800 rpm to yield the DNA-conjugate **SRu-67**. Next, the reaction mixture was diluted with 10 µL of Milli-Q water. To the reaction mixture was added the stock solution of NaCl in water (**SR-06** , 3.0 µL, c = 5.0 M, 10% volume of the total reaction volume), followed by cold ethanol (−20 °C, 99 µL) to precipitate the *N*-arylated ruthenium DNA conjugate **SRu-67**. The Eppendorf tube was placed in a freezer (−20 °C) for at least 1 hour, and then it was centrifuged at 4 °C and 11000 × g for at least 30 minutes. The supernatant was removed and the pellet was dried under air, then dissolved in 20 µL water to obtain the DNA-conjuagte **SRu-67** (20 µL, c =

0.10 mM). Then, 1.0 µL of the above solution was diluted to 40 µL with water for LC–MS analysis.

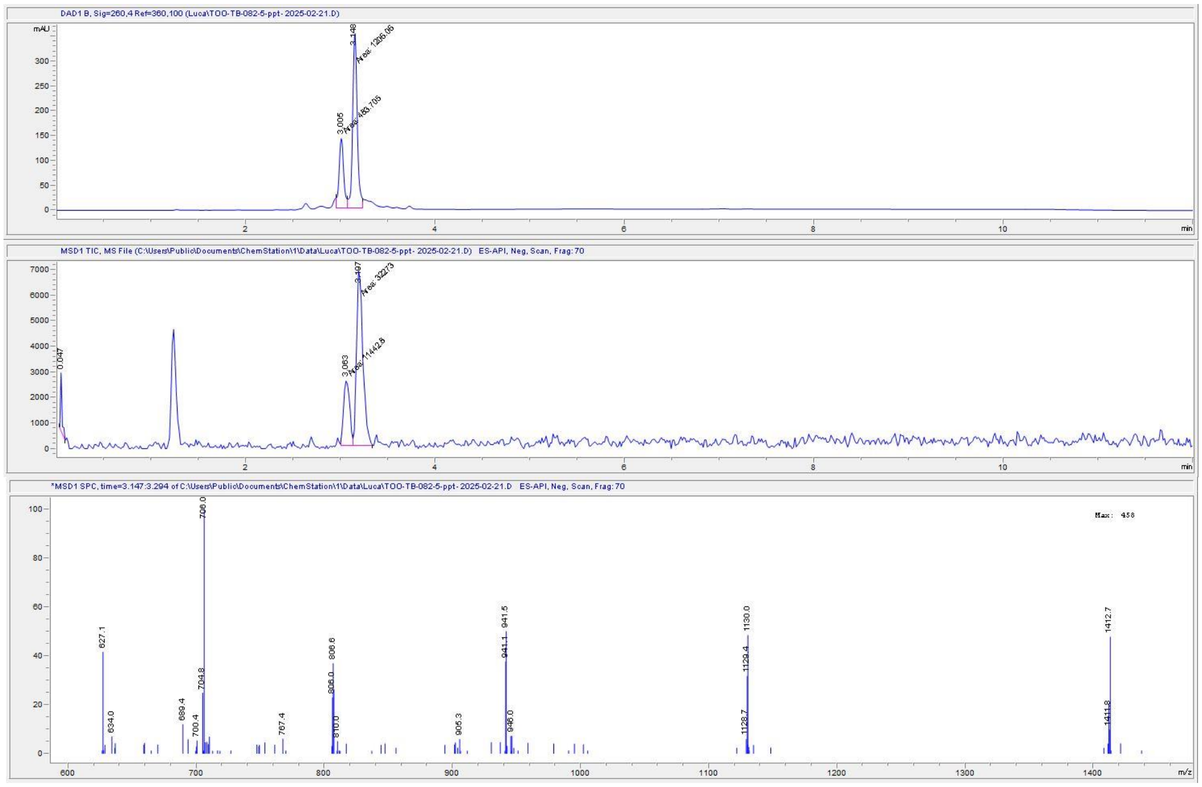

Figure S129. Analytical HPLC trace of **Ru-54** with HPLC method A. (Top) DAD chromatogram at 260 nm. (Middle) TIC chromatogram. (Bottom) Ionization of peak at 3.20 min. containing reaction product.

Decomplexation of SRu-67 to obtain product 67: Under an ambient atmosphere, the DNA-conjugate **SRu-67** stock solution in water (c = 0.10 mM, 20 µL) was irradiated with a 390 nm (40 W) Kessil lamp for 2 hours, while maintaining the temperature at approximately 30 °C through cooling with a fan. To the reaction mixture was added the stock solution of NaCl in water (**SR-06**, 2.0 µL, c = 5.0 M, 10% volume of the total reaction volume), followed by cold ethanol (−20 °C, 66 µL) to precipitate the DNA conjugate **67**. The Eppendorf tube was placed in the freezer (−20 °C) for at least 1 hour, and then it was centrifuged at 4 °C and 11000 × g for at least 30 minutes. The supernatant was removed, the pellet dried under air and dissolved in Milli-Q water to obtain the purified DNA-conjugate **67**. Then, 1 µL of the above solution was diluted to 40 µL with water for LC–MS analysis. The yield of the DNA conjugate was calculated by measuring the integration of the peaks of the diode array detection (DAD) UV absorbance at 260 nm of the LC–MS trace, assuming complete DNA recovery and identical UV absorbance.

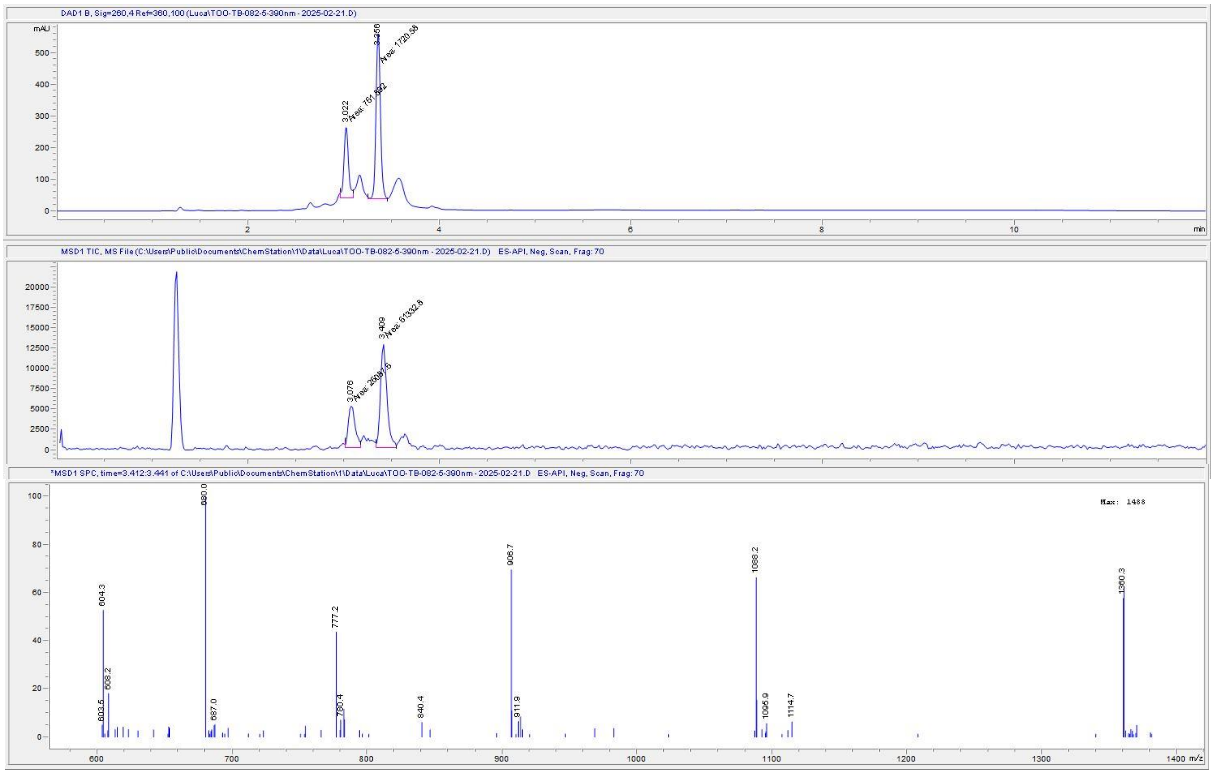

Figure S130. Analytical HPLC trace of **55** with HPLC method A. (Top) DAD chromatogram at 260 nm. (Middle) TIC chromatogram. (Bottom) Ionization of peak at 3.41 min. containing reaction product.

N-arylation of DNA-conjugate S23

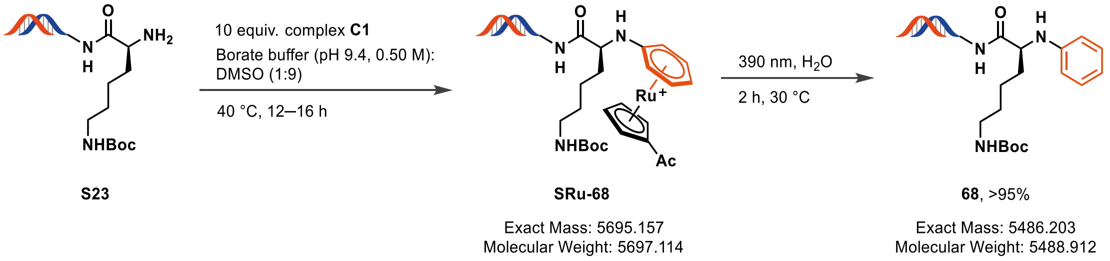

At 20–25 °C, the stock solution of DNA-conjugate **S23** (1.0 µL, c = 2.0 mM, 2.0 nmol, 1.0 equiv.) in water was added to a 1.5 mL Eppendorf tube, followed by sodium borate buffer (1.0 µL, pH 9.4, c = 0.50 M). To this mixture, 16 µL of DMSO was added and the solution was vortexed for 5 seconds. Next, the freshly prepared stock solution **C1** (2.0 µL, c = 0.01 M, 0.02 µmol, 10 equiv.) in DMSO was added. The resulting reaction mixture was vortexed for 5 seconds, transferred to a thermocycler at 40 °C, and incubated for 16 hours at 800 rpm to yield the DNA-conjugate **SRu-68**. Next, the reaction mixture was diluted with 10 µL of Milli-Q water. To the reaction mixture was added the stock solution of NaCl in water (**SR-06** , 3.0 µL, c = 5.0 M, 10% volume of the total reaction volume), followed by cold ethanol (−20 °C, 99 µL) to precipitate the *N*-arylated ruthenium DNA conjugate **SRu-68**. The Eppendorf tube was placed in a freezer (−20 °C) for at least 1 hour, and then it was centrifuged at 4 °C and 11000 × g for at least 30 minutes. The supernatant was removed and

the pellet was dried under air, then dissolved in 20 µL water to obtain the DNA-conjuagte **SRu-68** (20 µL, c = 0.10 mM). Then, 1.0 µL of the above solution was diluted to 40 µL with water for LC–MS analysis.

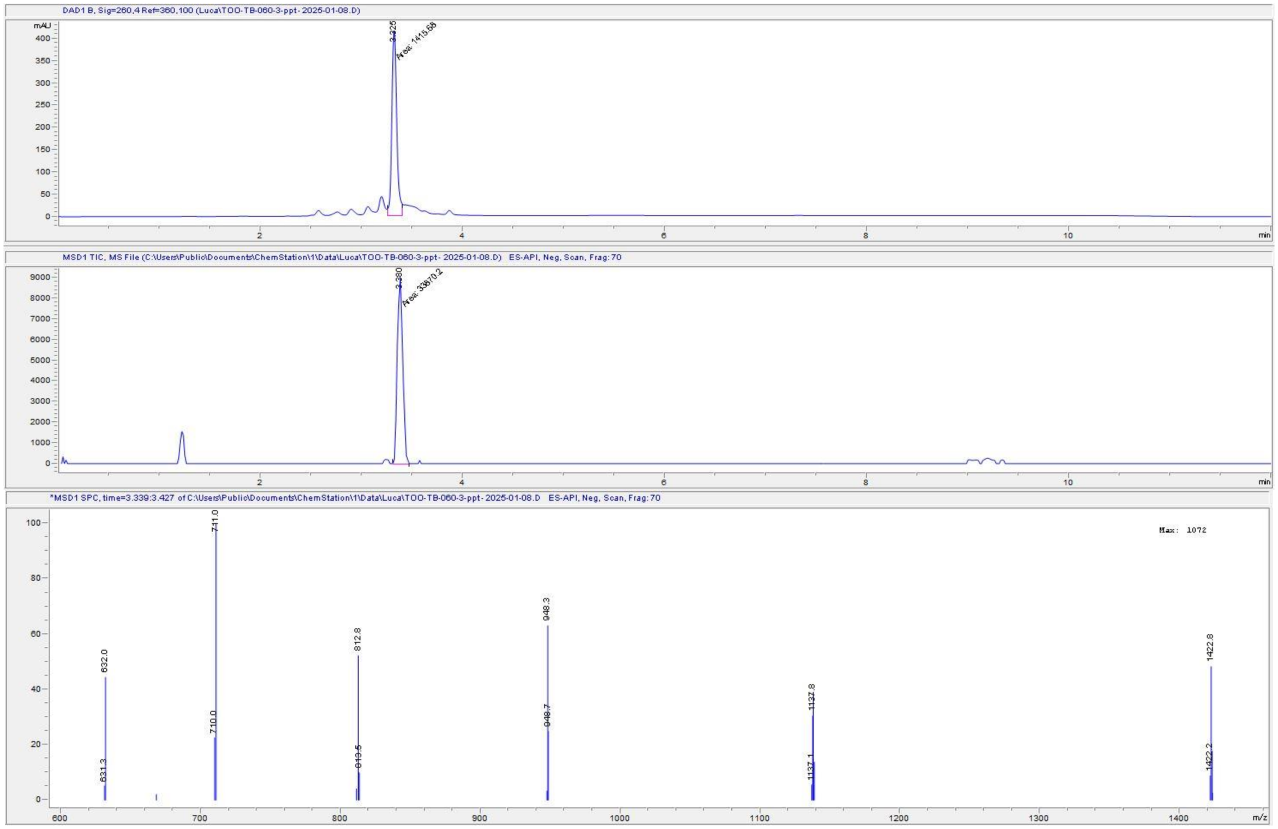

Figure S131. Analytical HPLC trace of **Ru-68** with HPLC method A. (Top) DAD chromatogram at 260 nm. (Middle) TIC chromatogram. (Bottom) Ionization of peak at 3.38 min. containing reaction product.

Decomplexation of SRu-68 to obtain product 68: Under an ambient atmosphere, the DNA-conjugate **SRu-68** stock solution in water (c = 0.10 mM, 20 µL) was irradiated with a 390 nm (40 W) Kessil lamp for 2 hours, while maintaining the temperature at approximately 30 °C through cooling with a fan. To the reaction mixture was added the stock solution of NaCl in water (**SR-06**, 2.0 µL, c = 5.0 M, 10% volume of the total reaction volume), followed by cold ethanol (−20 °C, 66 µL) to precipitate the DNA conjugate **68**. The Eppendorf tube was placed in the freezer (−20 °C) for at least 1 hour, and then it was centrifuged at 4 °C and 11000 × g for at least 30 minutes. The supernatant was removed, the pellet dried under air and dissolved in Milli-Q water to obtain the purified DNA-conjugate **68**. Then, 1 µL of the above solution was diluted to 40 µL with water for LC–MS analysis. The yield of the DNA conjugate was calculated by measuring the integration of the peaks of the diode array detection (DAD) UV absorbance at 260 nm of the LC–MS trace, assuming complete DNA recovery and identical UV absorbance.

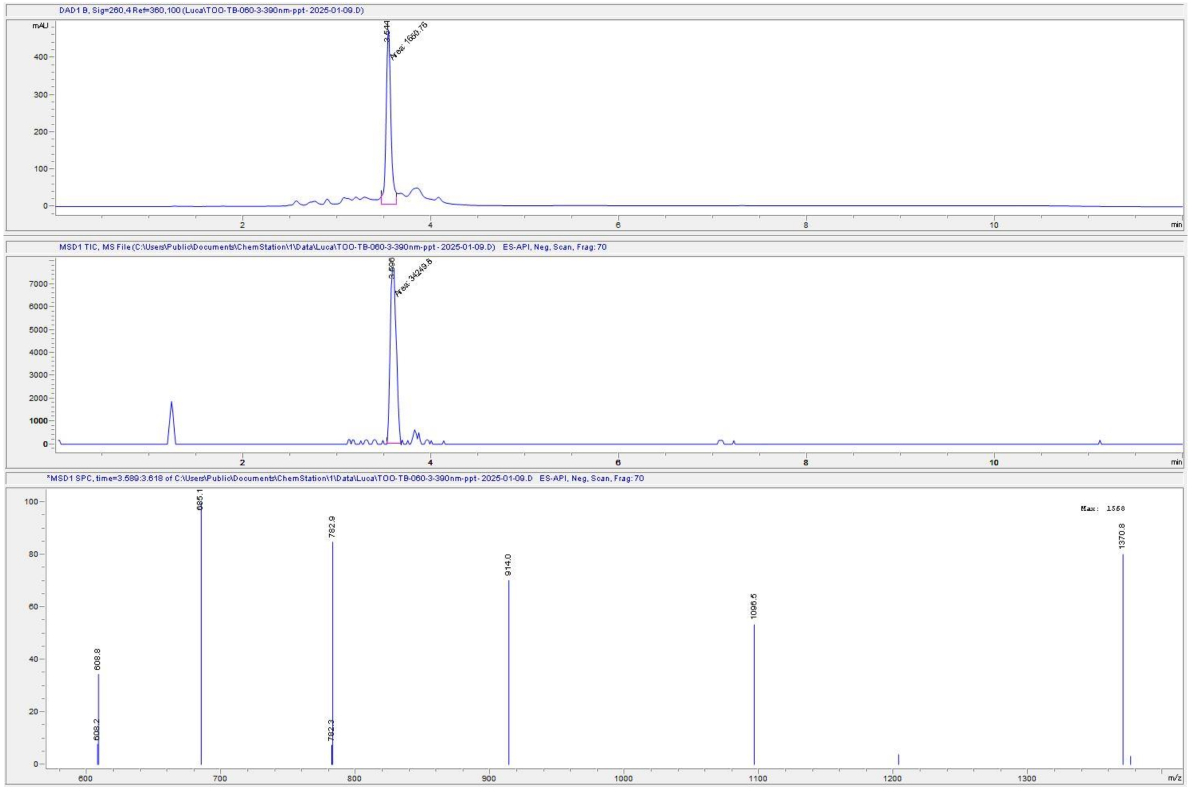

Figure S132. Analytical HPLC trace of **68** with HPLC method A. (Top) DAD chromatogram at 260 nm. (Middle) TIC chromatogram. (Bottom) Ionization of peak at 3.60 min. containing reaction product.

N-arylation of DNA-conjugate S24

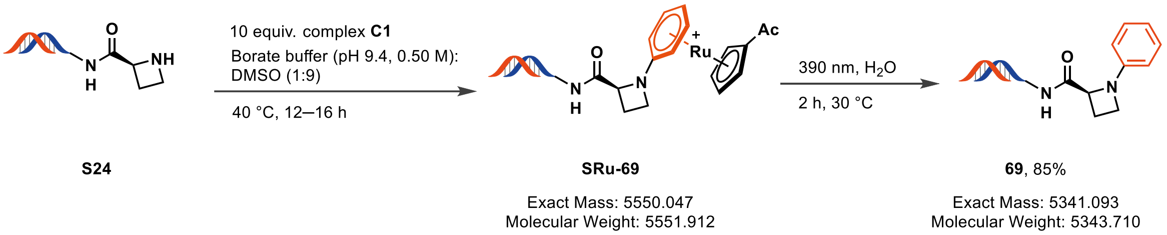

At 20–25 °C, the stock solution of DNA-conjugate **S24** (1.0 µL, c = 2.0 mM, 2.0 nmol, 1.0 equiv.) in water was added to a 1.5 mL Eppendorf tube, followed by sodium borate buffer (1.0 µL, pH 9.4, c = 0.50 M). To this mixture, 16 µL of DMSO was added and the solution was vortexed for 5 seconds. Next, the freshly prepared stock solution **C1** (2.0 µL, c = 0.01 M, 0.02 µmol, 10 equiv.) in DMSO was added. The resulting reaction mixture was vortexed for 5 seconds, transferred to a thermocycler at 40 °C, and incubated for 16 hours at 800 rpm to yield the DNA-conjugate **SRu-69**. Next, the reaction mixture was diluted with 10 µL of Milli-Q water. To the reaction mixture was added the stock solution of NaCl in water (**SR-06** , 3.0 µL, c = 5.0 M, 10% volume of the total reaction volume), followed by cold ethanol (−20 °C, 99 µL) to precipitate the *N*-arylated ruthenium DNA conjugate **SRu-69**. The Eppendorf tube was placed in a freezer (−20 °C) for at least 1 hour, and then it was centrifuged at 4 °C and 11000 × g for at least 30 minutes. The supernatant was removed and the pellet was dried under air, then dissolved in 20 µL water to obtain the DNA-conjugate **SRu-69** (20 µL, c =

0.10 mM). Then, 1.0 µL of the above solution was diluted to 40 µL with water for LC–MS analysis.

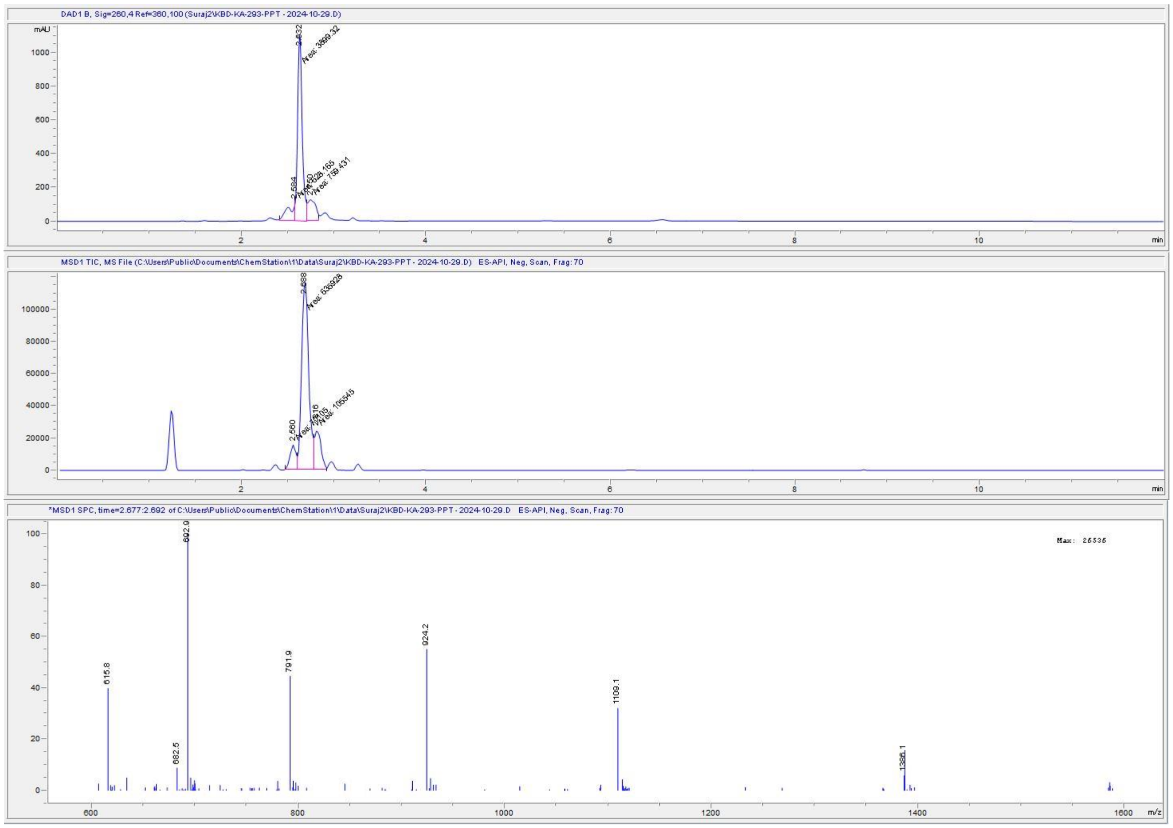

Figure S133. Analytical HPLC trace of **Ru-57** with HPLC method A. (Top) DAD chromatogram at 260 nm. (Middle) TIC chromatogram. (Bottom) Ionization of peak at 2.69 min. containing reaction product.

Decomplexation of SRu-69 to obtain product 69: Under an ambient atmosphere, the DNA-conjugate **SRu-69** stock solution in water (c = 0.10 mM, 20 µL) was irradiated with a 390 nm (40 W) Kessil lamp for 2 hours, while maintaining the temperature at approximately 30 °C through cooling with a fan. To the reaction mixture was added the stock solution of NaCl in water (**SR-06**, 2.0 µL, c = 5.0 M, 10% volume of the total reaction volume), followed by cold ethanol (−20 °C, 66 µL) to precipitate the DNA conjugate **69**. The Eppendorf tube was placed in the freezer (−20 °C) for at least 1 hour, and then it was centrifuged at 4 °C and 11000 × g for at least 30 minutes. The supernatant was removed, the pellet dried under air and dissolved in Milli-Q water to obtain the purified DNA-conjugate **69**. Then, 1 µL of the above solution was diluted to 40 µL with water for LC–MS analysis. The yield of the DNA conjugate was calculated by measuring the integration of the peaks of the diode array detection (DAD) UV absorbance at 260 nm of the LC–MS trace, assuming complete DNA recovery and identical UV absorbance.

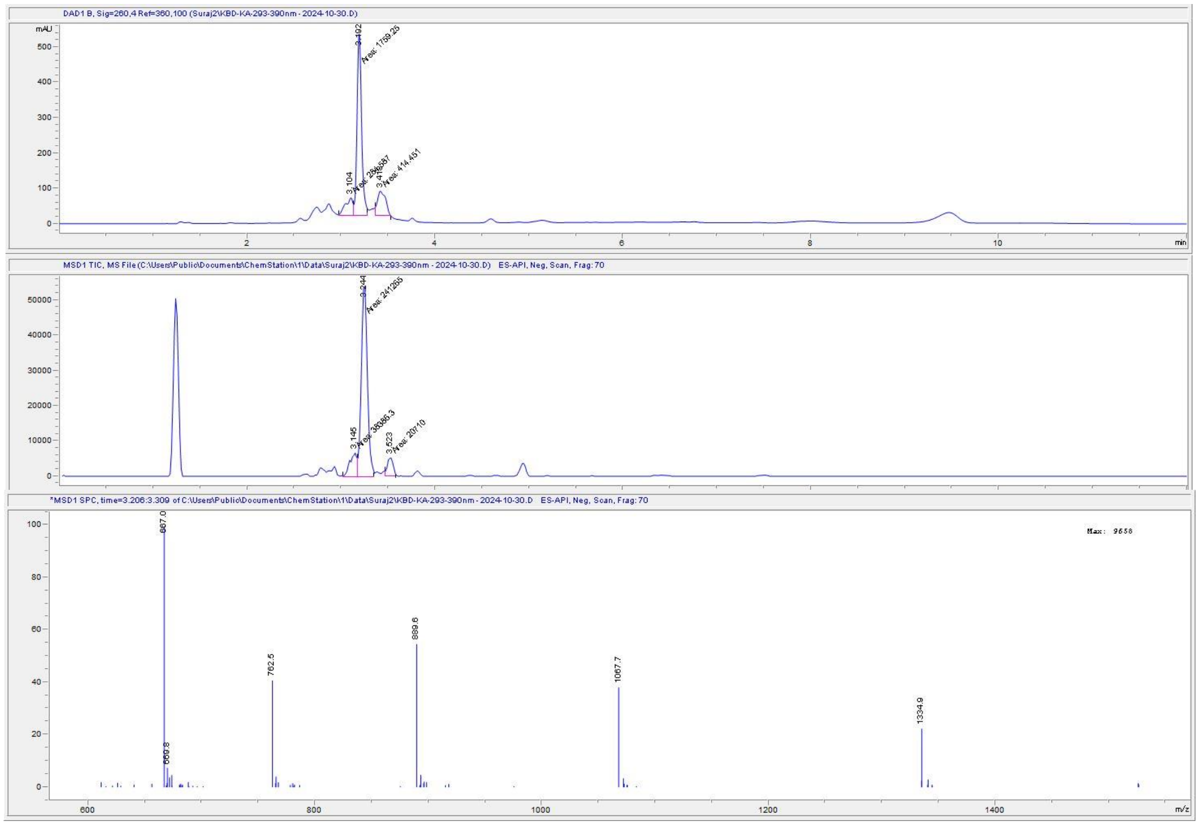

Figure S134. Analytical HPLC trace of **69** with HPLC method A. (Top) DAD chromatogram at 260 nm. (Middle) TIC chromatogram. (Bottom) Ionization of peak at 3.24 min. containing reaction product.

N-arylation of DNA-conjugate S25

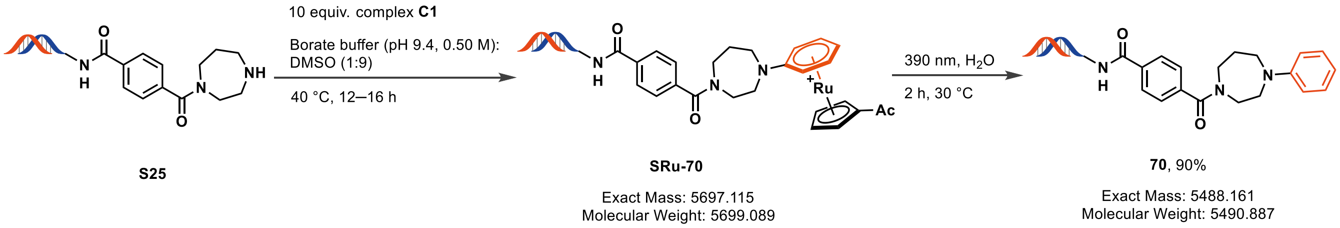

At 20–25 °C, the stock solution of DNA-conjugate **S25** (1.0 µL, c = 2.0 mM, 2.0 nmol, 1.0 equiv.) in water was added to a 1.5 mL Eppendorf tube, followed by sodium borate buffer (1.0 µL, pH 9.4, c = 0.50 M). To this mixture, 16 µL of DMSO was added and the solution was vortexed for 5 seconds. Next, the freshly prepared stock solution **C1** (2.0 µL, c = 0.01 M, 0.02 µmol, 10 equiv.) in DMSO was added. The resulting reaction mixture was vortexed for 5 seconds, transferred to a thermocycler at 40 °C, and incubated for 16 hours at 800 rpm to yield the DNA-conjugate **SRu-70**. Next, the reaction mixture was diluted with 10 µL of Milli-Q water. To the reaction mixture was added the stock solution of NaCl in water (**SR-06** , 3.0 µL, c = 5.0 M, 10% volume of the total reaction volume), followed by cold ethanol (−20 °C, 99 µL) to precipitate the *N*-arylated ruthenium DNA conjugate **SRu-70**. The Eppendorf tube was placed in a freezer (−20 °C) for at least 1 hour, and then it was centrifuged at 4 °C and 11000 × g for at least 30 minutes. The supernatant was removed and

the pellet was dried under air, then dissolved in 20 µL water to obtain the DNA-conjugate **SRu-70** (20 µL, c = 0.10 mM). Then, 1.0 µL of the above solution was diluted to 40 µL with water for LC–MS analysis.

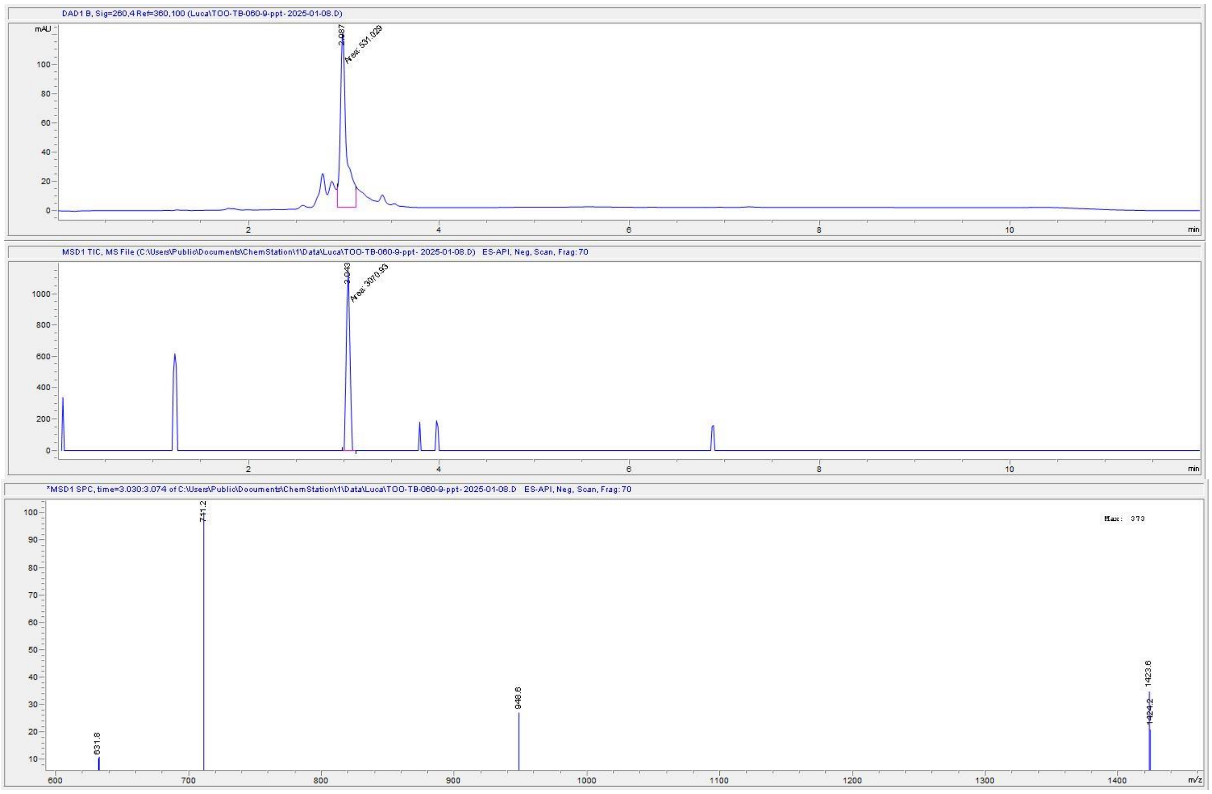

Figure S135. Analytical HPLC trace of **SRu-70** with HPLC method A. (Top) DAD chromatogram at 260 nm. (Middle) TIC chromatogram. (Bottom) Ionization of peak at 3.04 min. containing reaction product.

Decomplexation of SRu-70 to obtain product 70: Under an ambient atmosphere, the DNA-conjugate **SRu-73** stock solution in water (c = 0.10 mM, 20 µL) was irradiated with a 390 nm (40 W) Kessil lamp for 2 hours, while maintaining the temperature at approximately 30 °C through cooling with a fan. To the reaction mixture was added the stock solution of NaCl in water (**SR-06**, 2.0 µL, c = 5.0 M, 10% volume of the total reaction volume), followed by cold ethanol (−20 °C, 66 µL) to precipitate the DNA conjugate **70**. The Eppendorf tube was placed in the freezer (−20 °C) for at least 1 hour, and then it was centrifuged at 4 °C and 11000 × g for at least 30 minutes. The supernatant was removed, the pellet dried under air and dissolved in Milli-Q water to obtain the purified DNA-conjugate **70**. Then, 1 µL of the above solution was diluted to 40 µL with water for LC–MS analysis. The yield of the DNA conjugate was calculated by measuring the integration of the peaks of the diode array detection (DAD) UV absorbance at 260 nm of the LC–MS trace, assuming complete DNA recovery and identical UV absorbance.

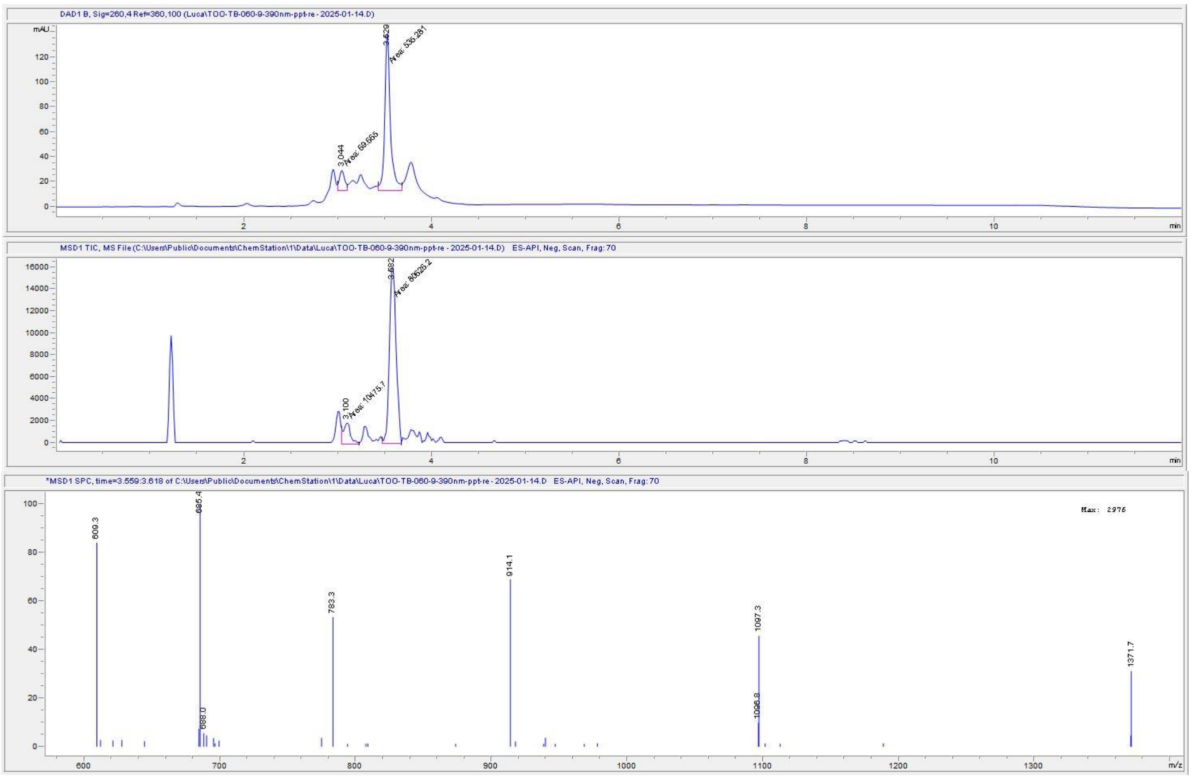

Figure S136. Analytical HPLC trace of **70** with HPLC method A. (Top) DAD chromatogram at 260 nm. (Middle) TIC chromatogram. (Bottom) Ionization of peak at 3.58 min. containing reaction product.

N-arylation of DNA-conjugate S26

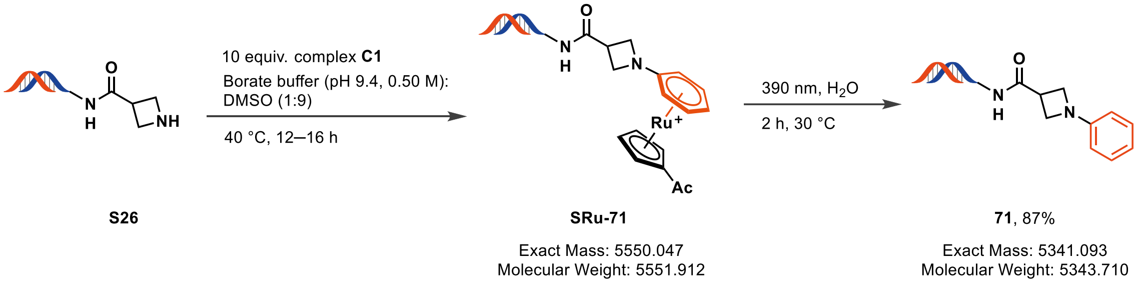

At 20–25 °C, the stock solution of DNA-conjugate **S26** (1.0 µL, c = 2.0 mM, 2.0 nmol, 1.0 equiv.) in water was added to a 1.5 mL Eppendorf tube, followed by sodium borate buffer (1.0 µL, pH 9.4, c = 0.50 M). To this mixture, 16 µL of DMSO was added and the solution was vortexed for 5 seconds. Next, the freshly prepared stock solution **C1** (2.0 µL, c = 0.01 M, 0.02 µmol, 10 equiv.) in DMSO was added. The resulting reaction mixture was vortexed for 5 seconds, transferred to a thermocycler at 40 °C, and incubated for 16 hours at 800 rpm to yield the DNA-conjugate **SRu-71**. Next, the reaction mixture was diluted with 10 µL of Milli-Q water. To the reaction mixture was added the stock solution of NaCl in water (**SR-06** , 3.0 µL, c = 5.0 M, 10% volume of the total reaction volume), followed by cold ethanol (−20 °C, 99 µL) to precipitate the *N*-arylated ruthenium DNA conjugate **SRu-71**. The Eppendorf tube was placed in a freezer (−20 °C) for at least 1 hour, and then it was centrifuged at 4 °C and 11000 × g for at least 30 minutes. The supernatant was removed and

the pellet was dried under air, then dissolved in 20 µL water to obtain the DNA-conjugate **SRu-71** (20 µL, c = 0.10 mM). Then, 1.0 µL of the above solution was diluted to 40 µL with water for LC–MS analysis.

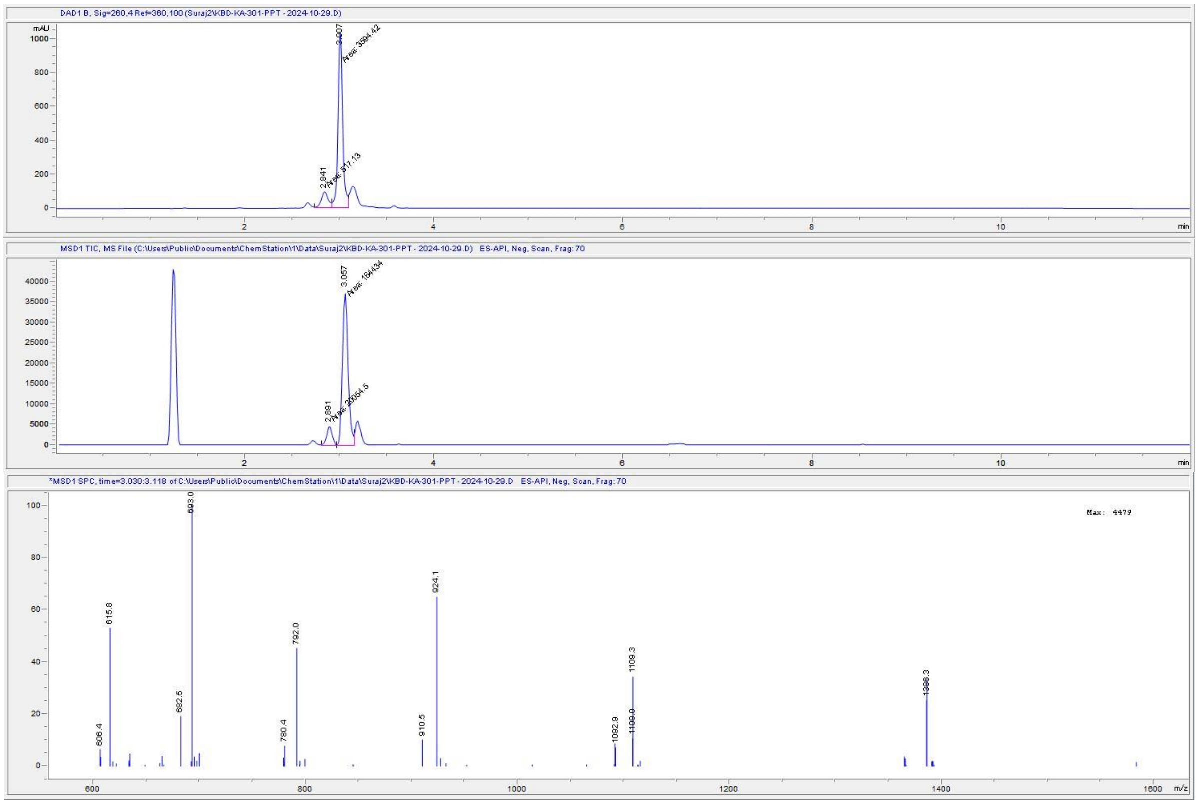

Figure S137. Analytical HPLC trace of **SRu-71** with HPLC method A. (Top) DAD chromatogram at 260 nm. (Middle) TIC chromatogram. (Bottom) Ionization of peak at 3.06 min. containing reaction product.

Decomplexation of SRu-71 to obtain product 71: Under an ambient atmosphere, the DNA-conjugate **SRu-71** stock solution in water (c = 0.10 mM, 20 µL) was irradiated with a 390 nm (40 W) Kessil lamp for 2 hours, while maintaining the temperature at approximately 30 °C through cooling with a fan. To the reaction mixture was added the stock solution of NaCl in water (**SR-06**, 2.0 µL, c = 5.0 M, 10% volume of the total reaction volume), followed by cold ethanol (−20 °C, 66 µL) to precipitate the DNA conjugate **71**. The Eppendorf tube was placed in the freezer (−20 °C) for at least 1 hour, and then it was centrifuged at 4 °C and 11000 × g for at least 30 minutes. The supernatant was removed, the pellet dried under air and dissolved in Milli-Q water to obtain the purified DNA-conjugate **71**. Then, 1 µL of the above solution was diluted to 40 µL with water for LC–MS analysis. The yield of the DNA conjugate was calculated by measuring the integration of the peaks of the diode array detection (DAD) UV absorbance at 260 nm of the LC–MS trace, assuming complete DNA recovery and identical UV absorbance.

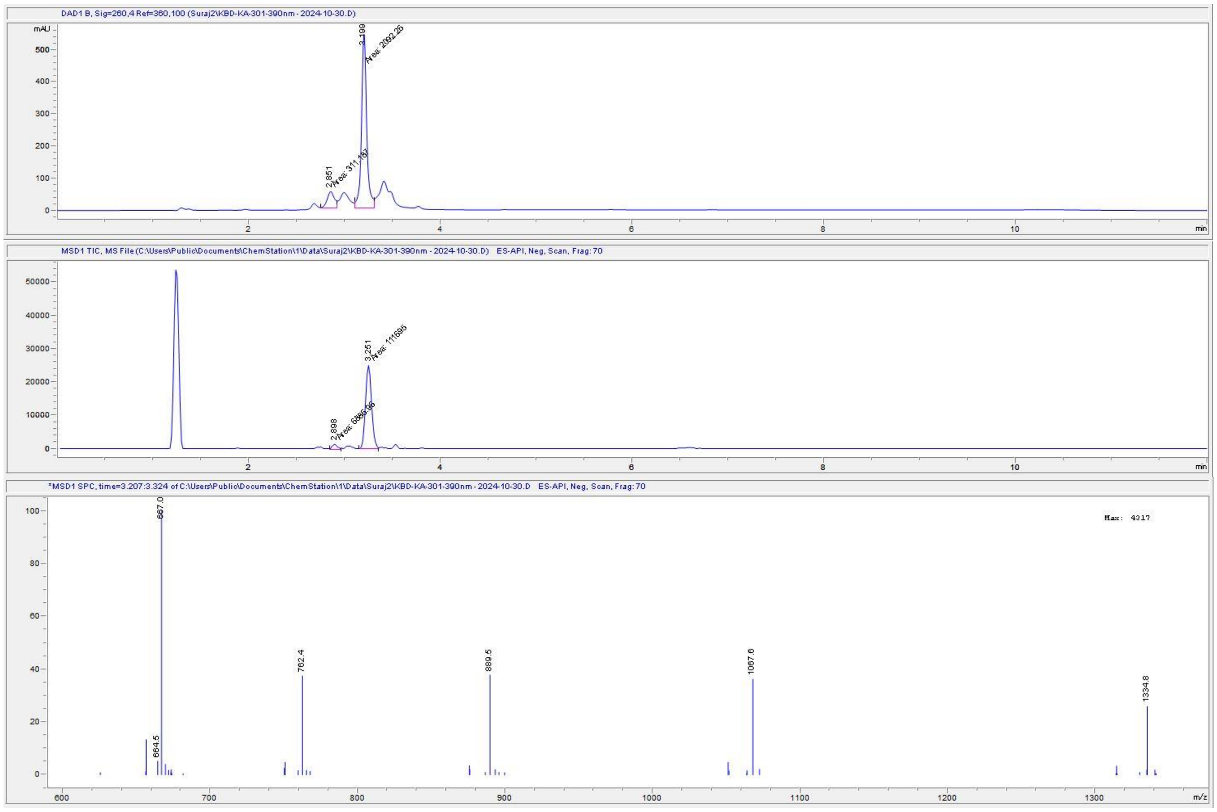

Figure S138. Analytical HPLC trace of **71** with HPLC method A. (Top) DAD chromatogram at 260 nm. (Middle) TIC chromatogram. (Bottom) Ionization of peak at 3.25 min. containing reaction product.

N-arylation of DNA-conjugate S27

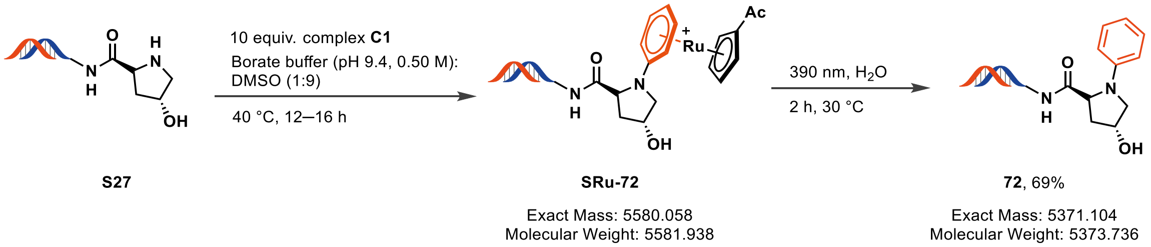

At 20–25 °C, the stock solution of DNA-conjugate **S27** (1.0 µL, c = 2.0 mM, 2.0 nmol, 1.0 equiv.) in water was added to a 1.5 mL Eppendorf tube, followed by sodium borate buffer (1.0 µL, pH 9.4, c = 0.50 M). To this mixture, 16 µL of DMSO was added and the solution was vortexed for 5 seconds. Next, the freshly prepared stock solution **C1** (2.0 µL, c = 0.01 M, 0.02 µmol, 10 equiv.) in DMSO was added. The resulting reaction mixture was vortexed for 5 seconds, transferred to a thermocycler at 40 °C, and incubated for 16 hours at 800 rpm to yield the DNA-conjugate **SRu-72**. Next, the reaction mixture was diluted with 10 µL of Milli-Q water. To the reaction mixture was added the stock solution of NaCl in water (**SR-06** , 3.0 µL, c = 5.0 M, 10% volume of the total reaction volume), followed by cold ethanol (−20 °C, 99 µL) to precipitate the *N*-arylated ruthenium DNA conjugate **SRu-72**. The Eppendorf tube was placed in a freezer (−20 °C) for at least 1 hour, and then it was centrifuged at 4 °C and 11000 × g for at least 30 minutes. The supernatant was removed and

the pellet was dried under air, then dissolved in 20 µL water to obtain the DNA-conjugate **SRu-72** (20 µL, c = 0.10 mM). Then, 1.0 µL of the above solution was diluted to 40 µL with water for LC–MS analysis.

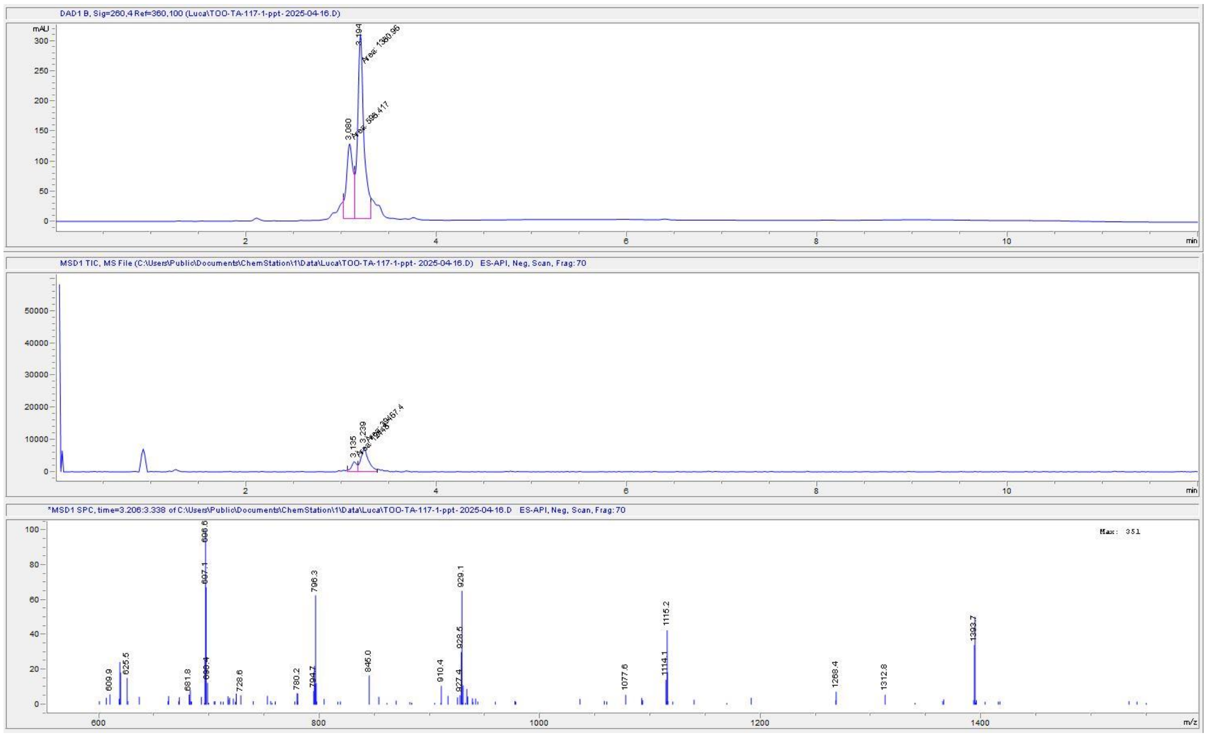

Figure S139. Analytical HPLC trace of **SRu-72** with HPLC method A. (Top) DAD chromatogram at 260 nm. (Middle) TIC chromatogram. (Bottom) Ionization of peak at 3.24 min. containing reaction product.

Decomplexation of SRu-72 to obtain product 72: Under an ambient atmosphere, the DNA-conjugate **SRu-72** stock solution in water (c = 0.10 mM, 20 µL) was irradiated with a 390 nm (40 W) Kessil lamp for 2 hours, while maintaining the temperature at approximately 30 °C through cooling with a fan. To the reaction mixture was added the stock solution of NaCl in water (**SR-06**, 2.0 µL, c = 5.0 M, 10% volume of the total reaction volume), followed by cold ethanol (−20 °C, 66 µL) to precipitate the DNA conjugate **72**. The Eppendorf tube was placed in the freezer (−20 °C) for at least 1 hour, and then it was centrifuged at 4 °C and 11000 × g for at least 30 minutes. The supernatant was removed, the pellet dried under air and dissolved in Milli-Q water to obtain the purified DNA-conjugate **72**. Then, 1 µL of the above solution was diluted to 40 µL with water for LC–MS analysis. The yield of the DNA conjugate was calculated by measuring the integration of the peaks of the diode array detection (DAD) UV absorbance at 260 nm of the LC–MS trace, assuming complete DNA recovery and identical UV absorbance.

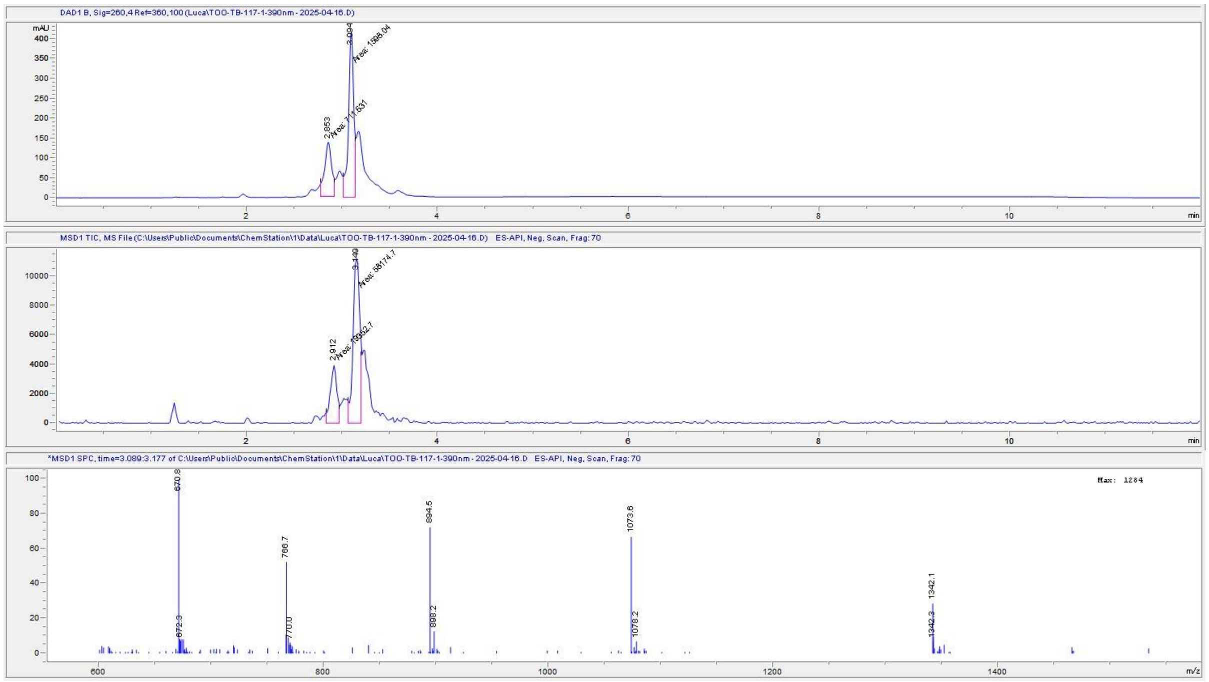

Figure S140. Analytical HPLC trace of **72** with HPLC method A. (Top) DAD chromatogram at 260 nm. (Middle) TIC chromatogram. (Bottom) Ionization of peak at 3.15 min. containing reaction product.

N-arylation of DNA-conjugate S28

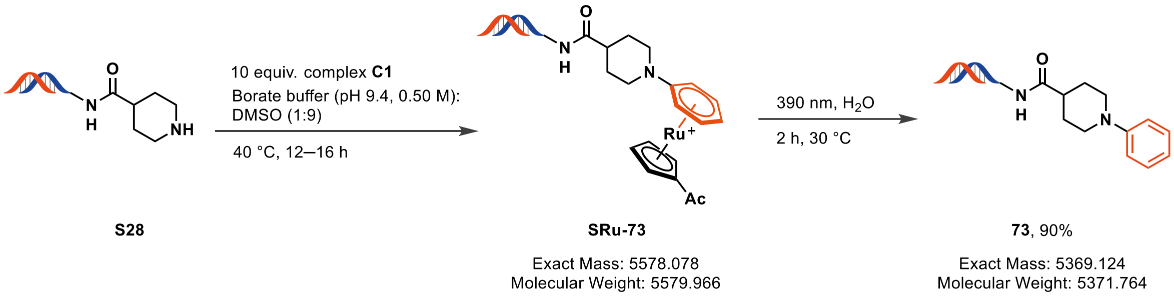

At 20–25 °C, the stock solution of DNA-conjugate **S28** (1.0 µL, c = 2.0 mM, 2.0 nmol, 1.0 equiv.) in water was added to a 1.5 mL Eppendorf tube, followed by sodium borate buffer (1.0 µL, pH 9.4, c = 0.50 M). To this mixture, 16 µL of DMSO was added and the solution was vortexed for 5 seconds. Next, the freshly prepared stock solution **C1** (2.0 µL, c = 0.01 M, 0.02 µmol, 10 equiv.) in DMSO was added. The resulting reaction mixture was vortexed for 5 seconds, transferred to a thermocycler at 40 °C, and incubated for 16 hours at 800 rpm to yield the DNA-conjugate **SRu-73**. Next, the reaction mixture was diluted with 10 µL of Milli-Q water. To the reaction mixture was added the stock solution of NaCl in water (**SR-06** , 3.0 µL, c = 5.0 M, 10% volume of the total reaction volume), followed by cold ethanol (−20 °C, 99 µL) to precipitate the *N*-arylated ruthenium DNA conjugate **SRu-73**. The Eppendorf tube was placed in a freezer (−20 °C) for at least 1 hour, and then it was centrifuged at 4 °C and 11000 × g for at least 30 minutes. The supernatant was removed and the pellet was dried under air, then dissolved in 20 µL water to obtain the DNA-conjugate **SRu-73** (20 µL, c = 0.10 mM). Then, 1.0 µL of the above solution was diluted to 40 µL with water for LC–MS analysis.

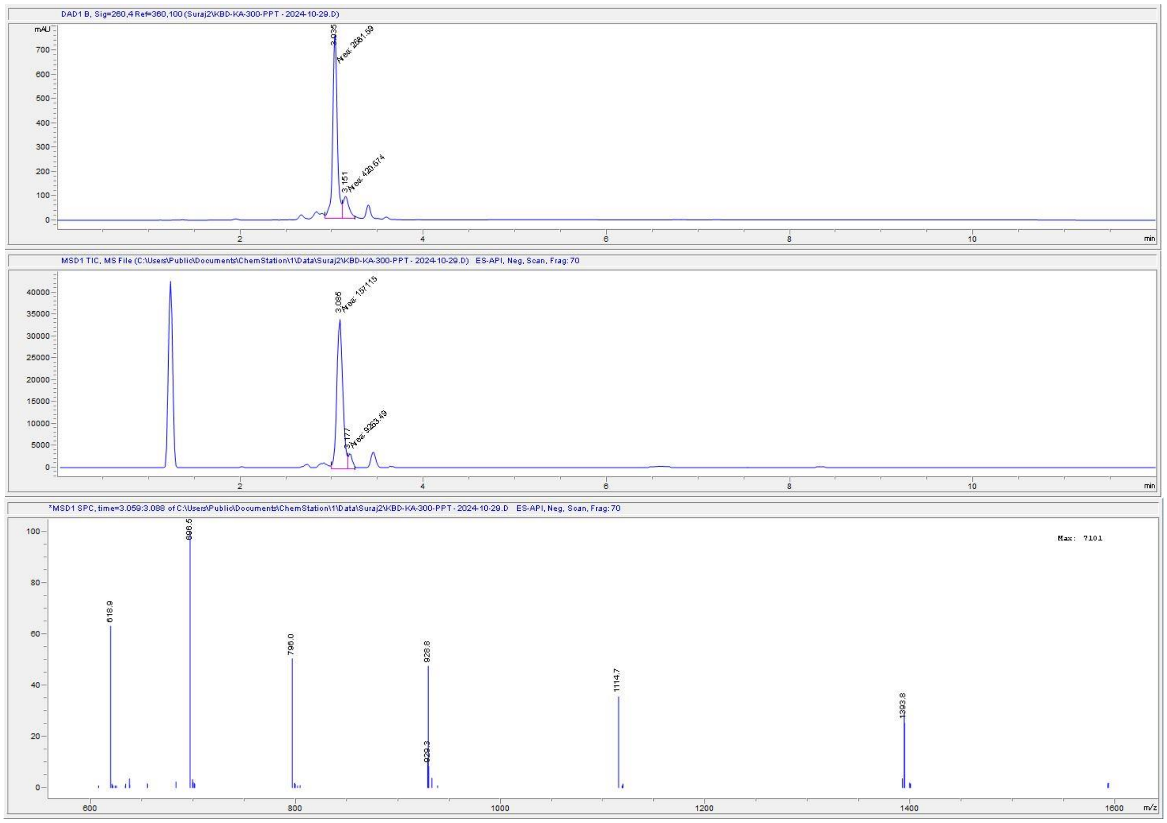

Figure S141. Analytical HPLC trace of **Ru-73** with HPLC method A. (Top) DAD chromatogram at 260 nm. (Middle) TIC chromatogram. (Bottom) Ionization of peak at 3.08 min. containing reaction product.

Decomplexation of SRu-73 to obtain product 73: Under an ambient atmosphere, the DNA-conjugate **SRu-73** stock solution in water (c = 0.10 mM, 20 µL) was irradiated with a 390 nm (40 W) Kessil lamp for 2 hours, while maintaining the temperature at approximately 30 °C through cooling with a fan. To the reaction mixture was added the stock solution of NaCl in water (**SR-06**, 2.0 µL, c = 5.0 M, 10% volume of the total reaction volume), followed by cold ethanol (−20 °C, 66 µL) to precipitate the DNA conjugate **73**. The Eppendorf tube was placed in the freezer (−20 °C) for at least 1 hour, and then it was centrifuged at 4 °C and 11000 × g for at least 30 minutes. The supernatant was removed, the pellet dried under air and dissolved in Milli-Q water to obtain the purified DNA-conjugate **73**. Then, 1 µL of the above solution was diluted to 40 µL with water for LC–MS analysis. The yield of the DNA conjugate was calculated by measuring the integration of the peaks of the diode array detection (DAD) UV absorbance at 260 nm of the LC–MS trace, assuming complete DNA recovery and identical UV absorbance.

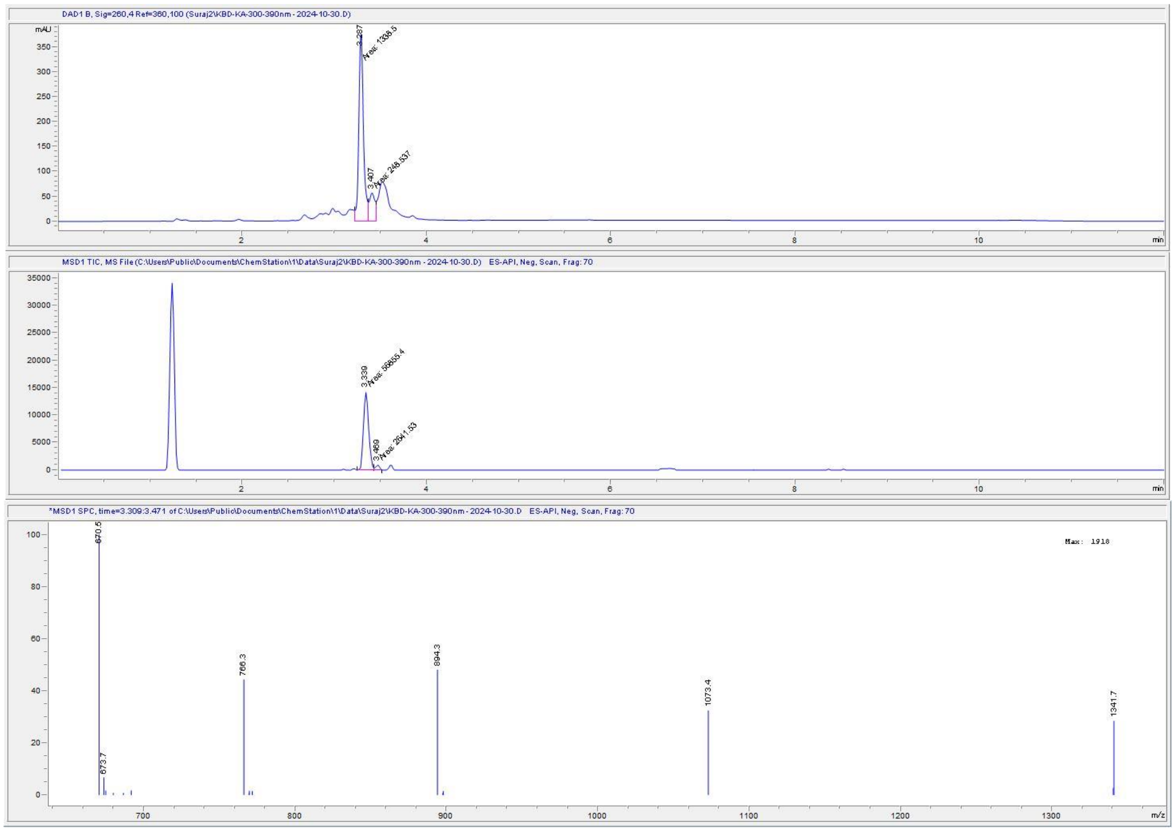

Figure S142. Analytical HPLC trace of **73** with HPLC method A. (Top) DAD chromatogram at 260 nm. (Middle) TIC chromatogram. (Bottom) Ionization of peak at 3.34 min. containing reaction product.

N-arylation of DNA-conjugate S29

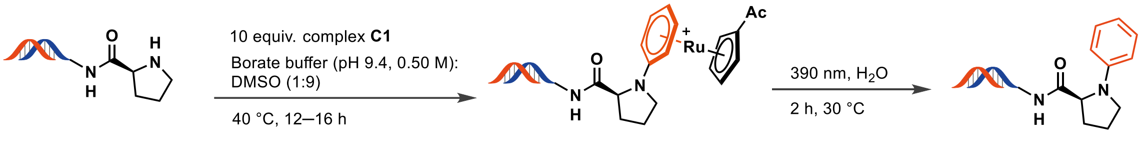

S29

SRu-74

Exact Mass: 5564.063
Molecular Weight: 5565.939

74, 81%

Exact Mass: 5355.109
Molecular Weight: 5357.737

At 20–25 °C, the stock solution of DNA-conjugate **S29** (1.0 µL, c = 2.0 mM, 2.0 nmol, 1.0 equiv.) in water was added to a 1.5 mL Eppendorf tube, followed by sodium borate buffer (1.0 µL, pH 9.4, c = 0.50 M). To this mixture, 16 µL of DMSO was added and the solution was vortexed for 5 seconds. Next, the freshly prepared stock solution **C1** (2.0 µL, c = 0.01 M, 0.02 µmol, 10 equiv.) in DMSO was added. The resulting reaction mixture was vortexed for 5 seconds, transferred to a thermocycler at 40 °C, and incubated for 16 hours at 800 rpm to yield the DNA-conjugate **SRu-74**. Next, the reaction mixture was diluted with 10 µL of Milli-Q water. To the reaction mixture was added the stock solution of NaCl in water (**SR-06** , 3.0 µL, c = 5.0 M, 10% volume of the total reaction volume), followed by cold ethanol (−20 °C, 99 µL) to precipitate the *N*-arylated ruthenium DNA conjugate **SRu-74**. The Eppendorf tube was placed in a freezer (−20 °C) for at least 1 hour, and then it was centrifuged at 4 °C and 11000 × g for at least 30 minutes. The supernatant was removed and

the pellet was dried under air, then dissolved in 20 µL water to obtain the DNA-conjugate **SRu-74** (20 µL, c = 0.10 mM). Then, 1.0 µL of the above solution was diluted to 40 µL with water for LC–MS analysis.

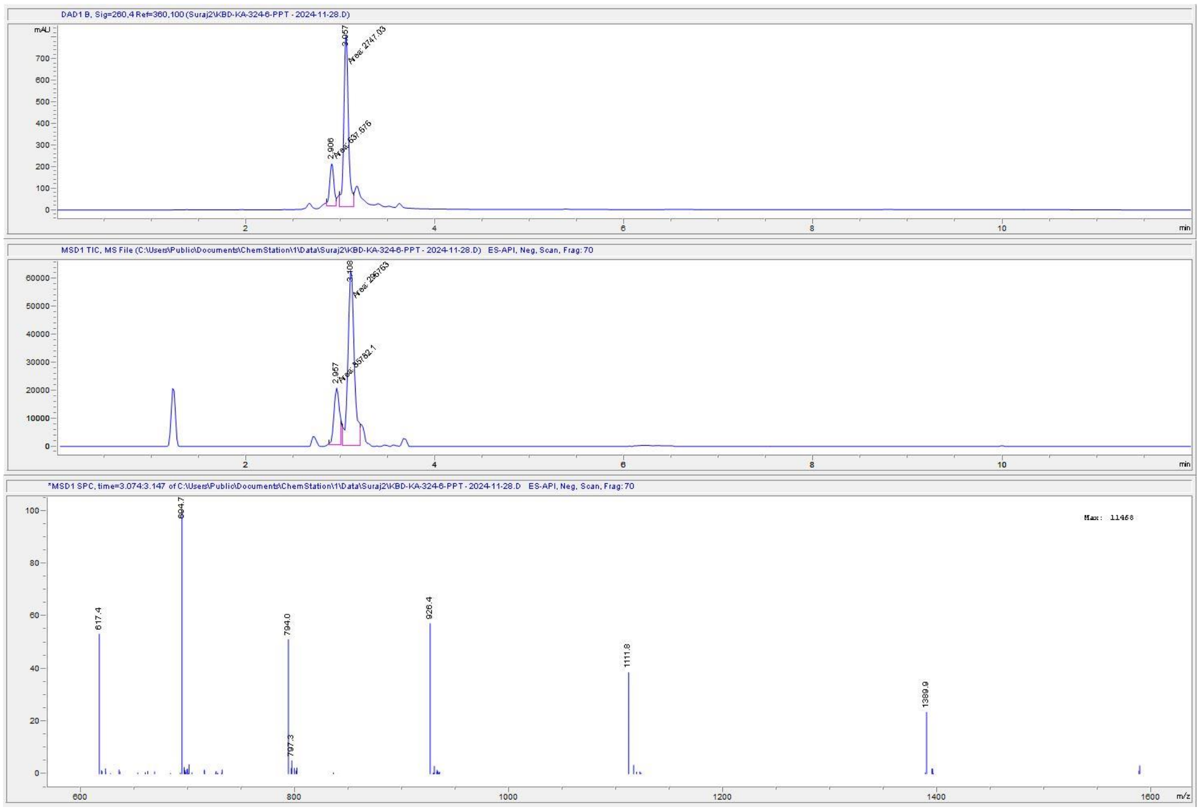

Figure S143. Analytical HPLC trace of **SRu-74** with HPLC method A. (Top) DAD chromatogram at 260 nm. (Middle) TIC chromatogram. (Bottom) Ionization of peak at 3.11 min. containing reaction product.

Decomplexation of SRu-74 to obtain product 74: Under an ambient atmosphere, the DNA-conjugate **SRu-74** stock solution in water (c = 0.10 mM, 20 µL) was irradiated with a 390 nm (40 W) Kessil lamp for 2 hours, while maintaining the temperature at approximately 30 °C through cooling with a fan. To the reaction mixture was added the stock solution of NaCl in water (**SR-06**, 2.0 µL, c = 5.0 M, 10% volume of the total reaction volume), followed by cold ethanol (−20 °C, 66 µL) to precipitate the DNA conjugate **74**. The Eppendorf tube was placed in the freezer (−20 °C) for at least 1 hour, and then it was centrifuged at 4 °C and 11000 × g for at least 30 minutes. The supernatant was removed, the pellet dried under air and dissolved in Milli-Q water to obtain the purified DNA-conjugate **74**. Then, 1 µL of the above solution was diluted to 40 µL with water for LC–MS analysis. The yield of the DNA conjugate was calculated by measuring the integration of the peaks of the diode array detection (DAD) UV absorbance at 260 nm of the LC–MS trace, assuming complete DNA recovery and identical UV absorbance.

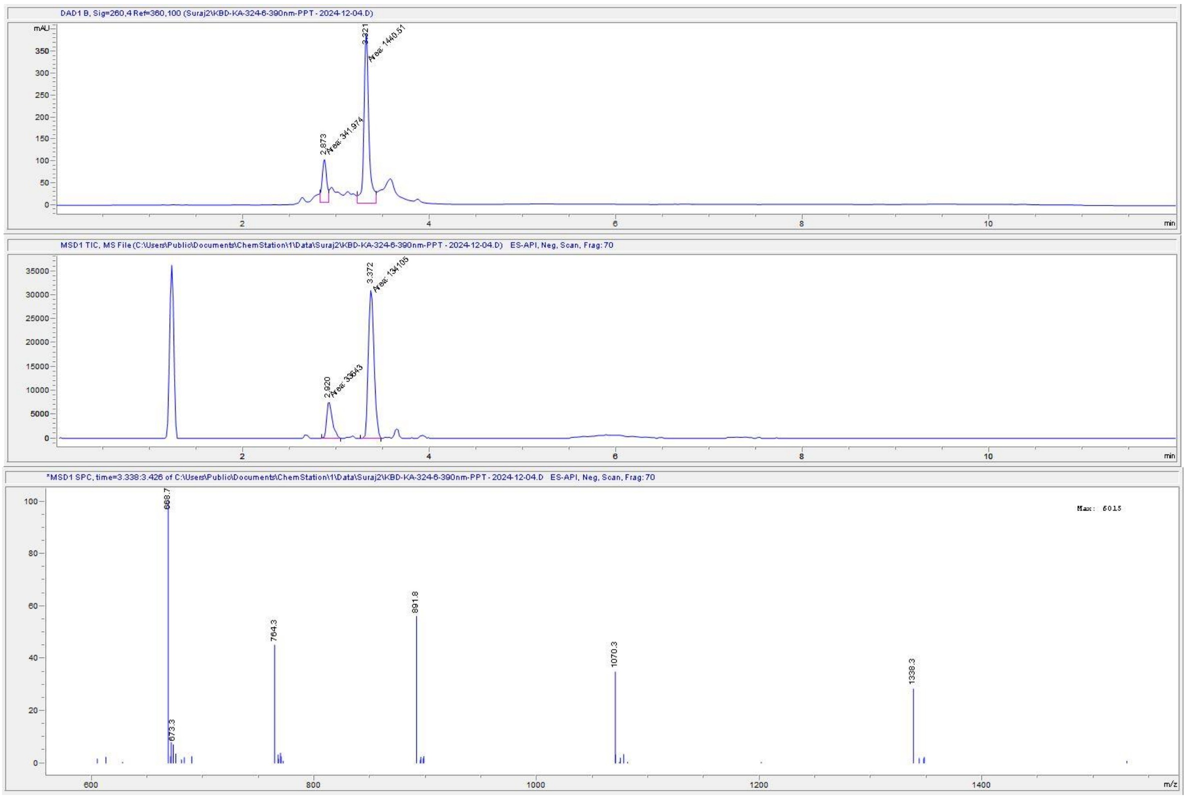

Figure S144. Analytical HPLC trace of **74** with HPLC method A. (Top) DAD chromatogram at 260 nm. (Middle) TIC chromatogram. (Bottom) Ionization of peak at 3.37 min. containing reaction product.

N-arylation of DNA-conjugate S30

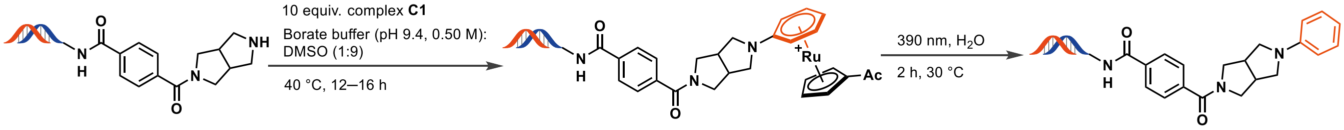

S30

SRu-75

Exact Mass: 5709.115
Molecular Weight: 5711.100

75, 90%

Exact Mass: 5500.161
Molecular Weight: 5502.898

At 20–25 °C, the stock solution of DNA-conjugate **S30** (1.0 µL, c = 2.0 mM, 2.0 nmol, 1.0 equiv.) in water was added to a 1.5 mL Eppendorf tube, followed by sodium borate buffer (1.0 µL, pH 9.4, c = 0.50 M). To this mixture, 16 µL of DMSO was added and the solution was vortexed for 5 seconds. Next, the freshly prepared stock solution **C1** (2.0 µL, c = 0.01 M, 0.02 µmol, 10 equiv.) in DMSO was added. The resulting reaction mixture was vortexed for 5 seconds, transferred to a thermocycler at 40 °C, and incubated for 16 hours at 800 rpm to yield the DNA-conjugate **SRu-75**. Next, the reaction mixture was diluted with 10 µL of Milli-Q water. To the reaction mixture was added the stock solution of NaCl in water (**SR-06** , 3.0 µL, c = 5.0 M, 10% volume of the total reaction volume), followed by cold ethanol (−20 °C, 99 µL) to precipitate the *N*-arylated ruthenium DNA conjugate **SRu-75**. The Eppendorf tube was placed in a freezer (−20 °C) for at least 1 hour, and then it was centrifuged at 4 °C and 11000 × g for at least 30 minutes. The supernatant was removed and the pellet was dried under air, then dissolved in 20 µL water to obtain the DNA-conjugate **SRu-75** (20 µL, c =

0.10 mM). Then, 1.0 µL of the above solution was diluted to 40 µL with water for LC–MS analysis.

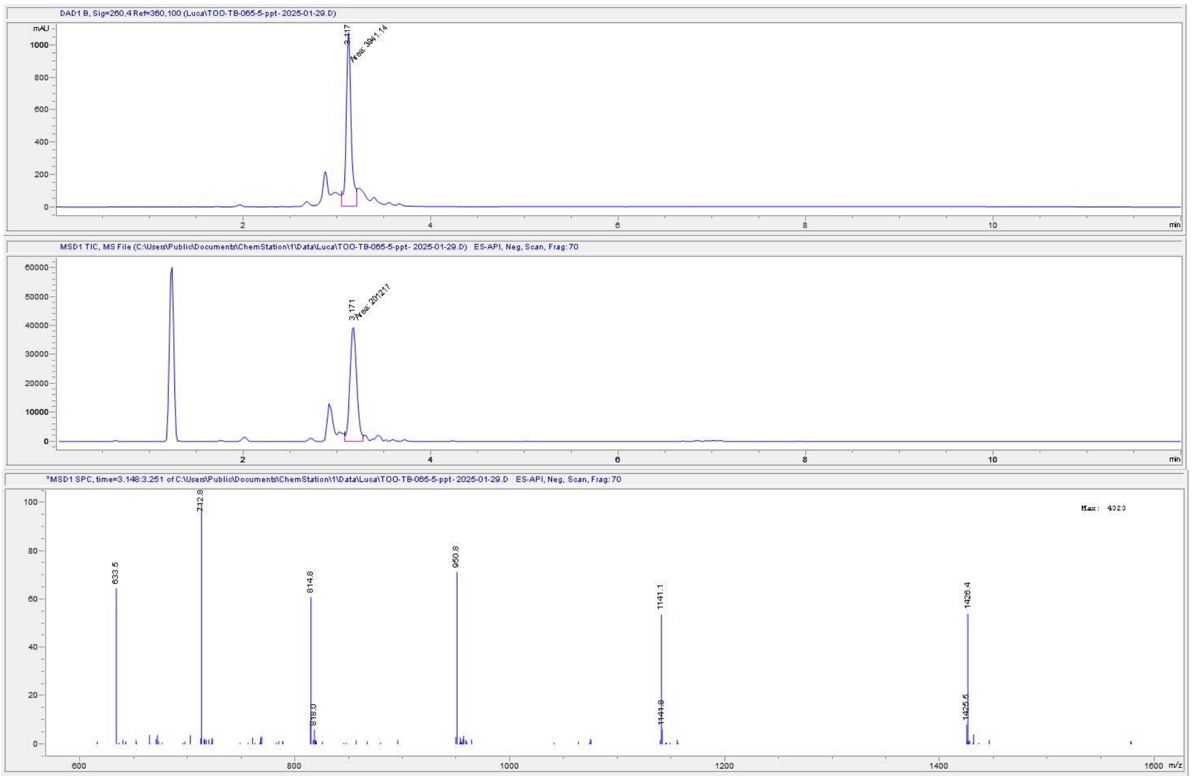

Figure S145. Analytical HPLC trace of **Ru-75** with HPLC method A. (Top) DAD chromatogram at 260 nm. (Middle) TIC chromatogram. (Bottom) Ionization of peak at 3.17 min. containing reaction product

Decomplexation of SRu-75 to obtain product 75: Under an ambient atmosphere, the DNA-conjugate **SRu-75** stock solution in water (c = 0.10 mM, 20 µL) was irradiated with a 390 nm (40 W) Kessil lamp for 2 hours, while maintaining the temperature at approximately 30 °C through cooling with a fan. To the reaction mixture was added the stock solution of NaCl in water (**SR-06**, 2.0 µL, c = 5.0 M, 10% volume of the total reaction volume), followed by cold ethanol (−20 °C, 66 µL) to precipitate the DNA conjugate **75**. The Eppendorf tube was placed in the freezer (−20 °C) for at least 1 hour, and then it was centrifuged at 4 °C and 11000 × g for at least 30 minutes. The supernatant was removed, the pellet dried under air and dissolved in Milli-Q water to obtain the purified DNA-conjugate **75**. Then, 1 µL of the above solution was diluted to 40 µL with water for LC–MS analysis. The yield of the DNA conjugate was calculated by measuring the integration of the peaks of the diode array detection (DAD) UV absorbance at 260 nm of the LC–MS trace, assuming complete DNA recovery and identical UV absorbance.

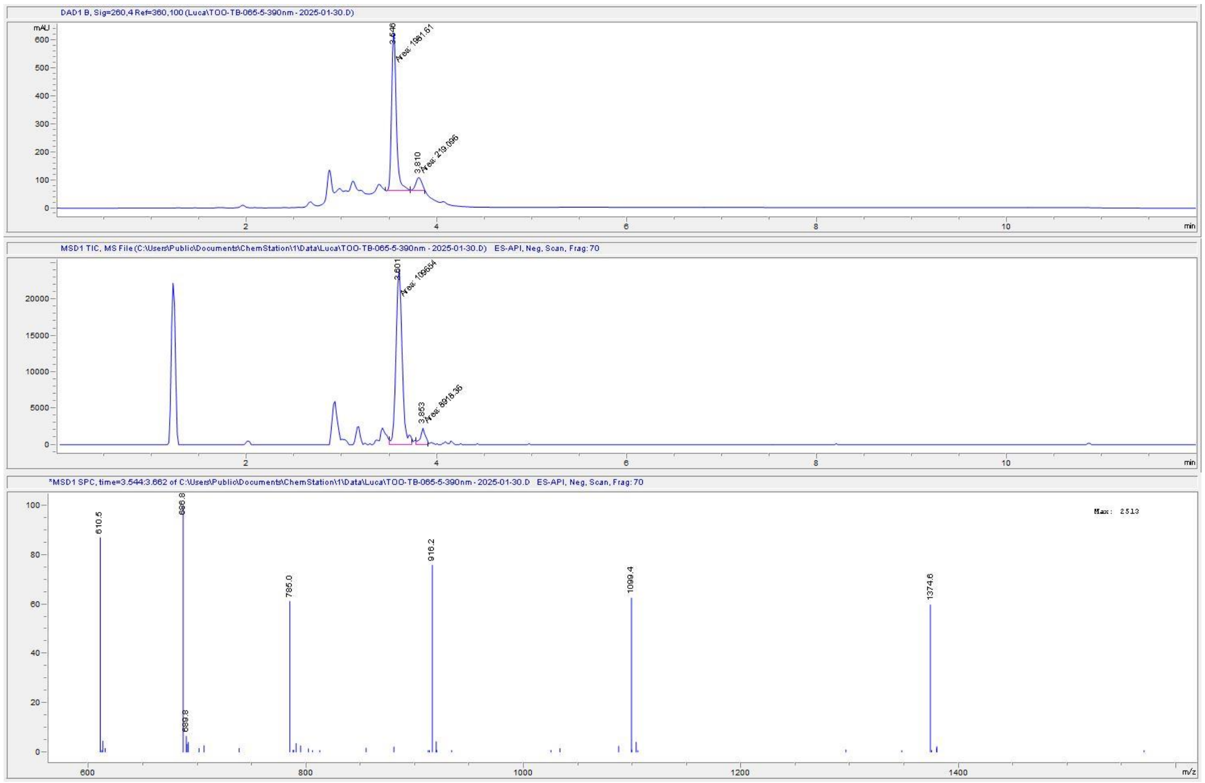

Figure S146. Analytical HPLC trace of **75** with HPLC method A. (Top) DAD chromatogram at 260 nm. (Middle) TIC chromatogram. (Bottom) Ionization of peak at 3.60 min. containing reaction product.

Synthesis of DNA-conjugate 76

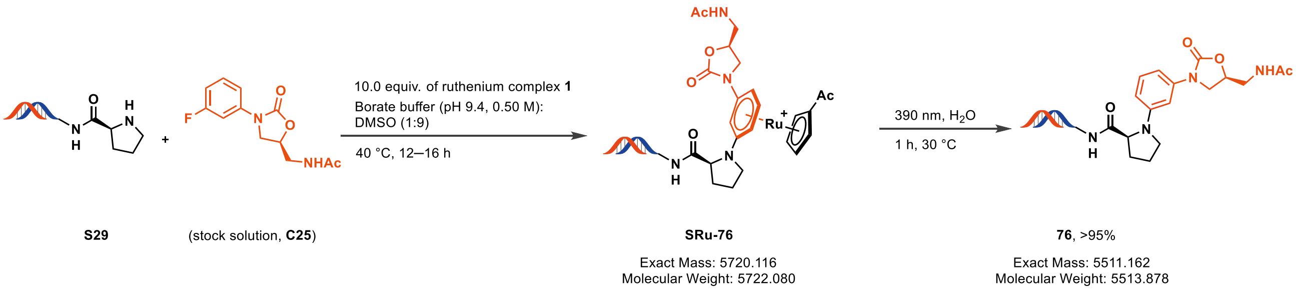

In situ formation of arene-ruthenium stock solution: Under an ambient atmosphere, a 1 mL glass GC vial equipped with a 6 mm Teflon-coated stirring bar was charged with ruthenium complex **1** (1.4 mg, 2.9 µmol, 1.0 equiv.). Next, a stock solution of (*S*)-N-((3-(3-fluorophenyl)-2-oxooxazolidin-5-yl)methyl)acetamide in DMC (294 µL, c = 0.10 M, 29 µmol, 10 equiv.) was added. The resulting reaction mixture was heated at 80 °C for 2 hours. After 2 hours, the reaction mixture was cooled to 23 °C. Next, the DMC was removed under a gentle stream of argon and 294 µL of DMSO were added to result an in situ formed stock solution of arene-ruthenium complex **C25** (294 µL, c = 0.01 M, assuming quantitative arene coordination to ruthenium).

At 20–25 °C, the stock solution of DNA-conjugate **S29** (1.0 µL, c = 2.0 mM, 2.0 nmol, 1.0 equiv.) in water was added to a 1.5 mL Eppendorf tube, followed by sodium borate buffer (1.0 µL, pH 9.4, c = 0.50 M). To this

mixture, 16 µL of DMSO was added and the solution was vortexed for 5 seconds. Next, the freshly prepared stock solution **C25** (2.0 µL, c = 0.01 M, 0.02 µmol, 10 equiv.) in DMSO was added. The resulting reaction mixture was vortexed for 5 seconds, transferred to a thermocycler at 40 °C, and incubated for 16 hours at 800 rpm to yield the DNA-conjugate **SRu-76**. Next, the reaction mixture was diluted with 10 µL of Milli-Q water. To the reaction mixture was added the stock solution of NaCl in water (**SR-06** , 3.0 µL, c = 5.0 M, 10% volume of the total reaction volume), followed by cold ethanol (−20 °C, 99 µL) to precipitate the *N*-arylated ruthenium DNA conjugate **SRu-76**. The Eppendorf tube was placed in a freezer (−20 °C) for at least 1 hour, and then it was centrifuged at 4 °C and 11000 × g for at least 30 minutes. The supernatant was removed and the pellet was dried under air, then dissolved in 20 µL water to obtain the DNA-conjugate **SRu-76** (20 µL, c = 0.10 mM). Then, 1.0 µL of the above solution was diluted to 40 µL with water for LC–MS analysis.

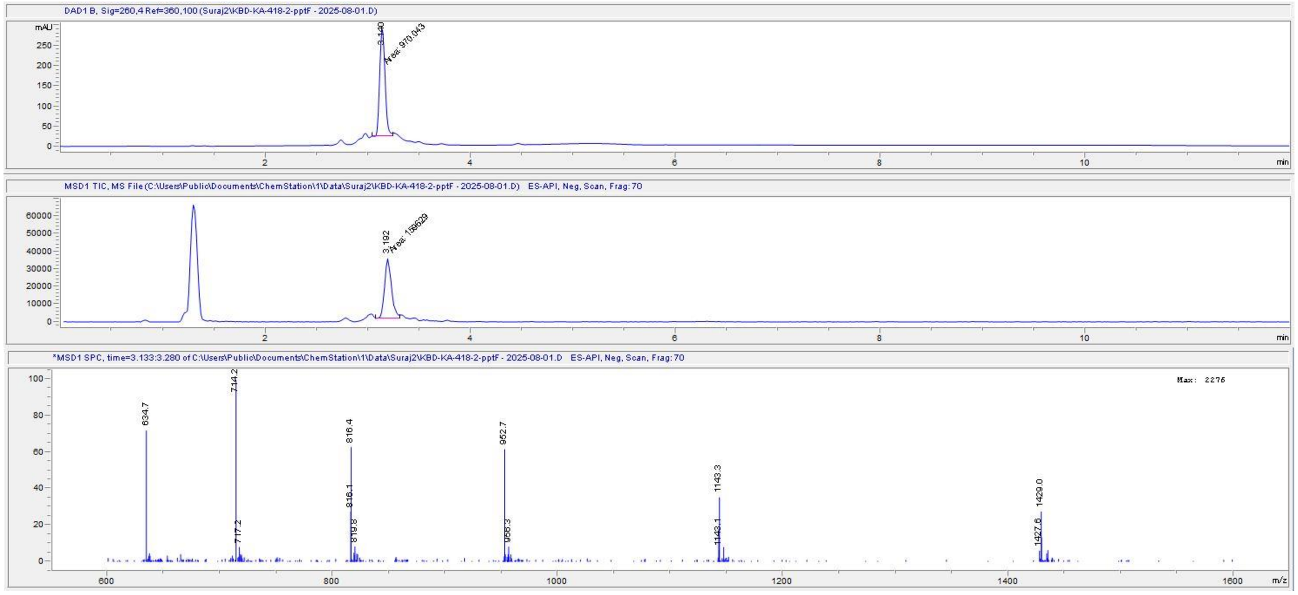

Figure S147. Analytical HPLC trace of **SRu-76** with HPLC method A. (Top) DAD chromatogram at 260 nm. (Middle) TIC chromatogram. (Bottom) Ionization of peak at 3.19 min. containing reaction product.

Decomplexation of SRu-76 to obtain product 76: Under an ambient atmosphere, the DNA-conjugate **SRu-76** stock solution in water (c = 0.10 mM, 20 µL) was irradiated with a 390 nm (40 W) Kessil lamp for 1 hours, while maintaining the temperature at approximately 30 °C through cooling with a fan. To the reaction mixture was added the stock solution of NaCl in water (**SR-06**, 2.0 µL, c = 5.0 M, 10% volume of the total reaction volume), followed by cold ethanol (−20 °C, 66 µL) to precipitate the DNA conjugate **76**. The Eppendorf tube was placed in the freezer (−20 °C) for at least 1 hour, and then it was centrifuged at 4 °C and 11000 × g for at least 30 minutes. The supernatant was removed, the pellet dried under air and dissolved in Milli-Q water to obtain the purified DNA-conjugate **76**. Then, 1 µL of the above solution was diluted to 40 µL with water for LC–MS analysis. The yield of the DNA conjugate was calculated by measuring the integration of the peaks of the diode array detection (DAD) UV absorbance at 260 nm of the LC–MS trace, assuming complete DNA recovery and identical UV absorbance.

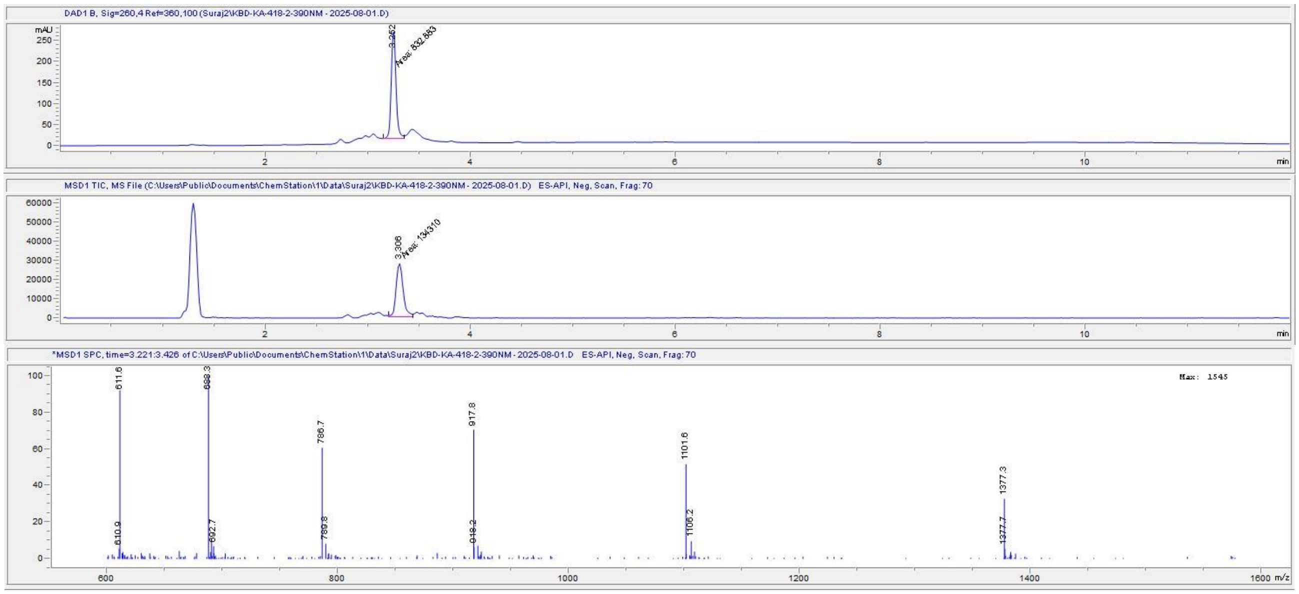

Figure S148. Analytical HPLC trace of **76** with HPLC method A. (Top) DAD chromatogram at 260 nm. (Middle) TIC chromatogram. (Bottom) Ionization of peak at 3.30 min. containing reaction product.

Synthesis of DNA-conjugate 77

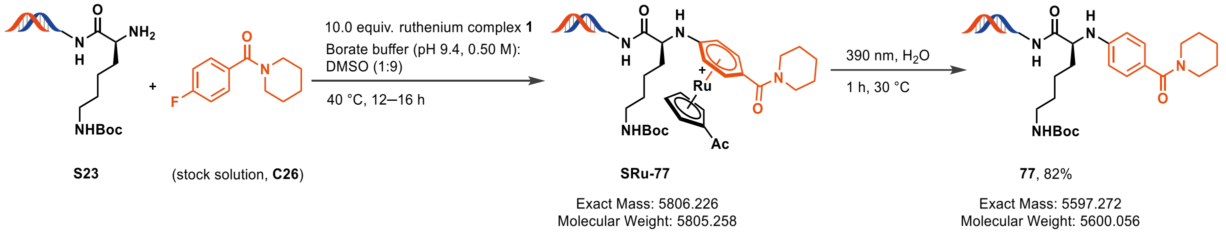

In situ formation of arene-ruthenium stock solution: Under an ambient atmosphere, a 1 mL glass GC vial equipped with a 6 mm Teflon-coated stirring bar was charged with ruthenium complex **1** (1.4 mg, 2.9 µmol, 1.0 equiv.). Next, a stock solution of (4-fluorophenyl)(piperidin-1-yl)methanone in DMC (294 µL, c = 0.10 M, 29 µmol, 10 equiv.) was added. The resulting reaction mixture was heated at 80 °C for 2 hours. After 2 hours, the reaction mixture was cooled to 23 °C. Next, the DMC was removed under a gentle stream of argon and 294 µL of DMSO were added to result an in situ formed stock solution of arene-ruthenium complex **C26** (294 µL, c = 0.01 M, assuming quantitative arene coordination to ruthenium).

At 20–25 °C, the stock solution of DNA-conjugate **S23** (1.0 µL, c = 2.0 mM, 2.0 nmol, 1.0 equiv.) in water was added to a 1.5 mL Eppendorf tube, followed by sodium borate buffer (1.0 µL, pH 9.4, c = 0.50 M). To this mixture, 16 µL of DMSO was added and the solution was vortexed for 5 seconds. Next, the freshly prepared stock solution **C26** (2.0 µL, c = 0.01 M, 0.02 µmol, 10 equiv.) in DMSO was added. The resulting reaction mixture was vortexed for 5 seconds, transferred to a thermocycler at 40 °C, and incubated for 16 hours at 800 rpm to yield the DNA-conjugate **SRu-77**. Next, the reaction mixture was diluted with 10 µL of Milli-Q water. To the reaction mixture was added the stock solution of NaCl in water (**SR-06** , 3.0 µL, c = 5.0 M, 10% volume of the total reaction volume), followed by cold ethanol (−20 °C, 99 µL) to precipitate the *N*-arylated

ruthenium DNA conjugate **SRu-77**. The Eppendorf tube was placed in a freezer (−20 °C) for at least 1 hour, and then it was centrifuged at 4 °C and 11000 × g for at least 30 minutes. The supernatant was removed and the pellet was dried under air, then dissolved in 20 µL water to obtain the DNA-conjugate **SRu-77** (20 µL, c = 0.10 mM). Then, 1.0 µL of the above solution was diluted to 40 µL with water for LC–MS analysis.

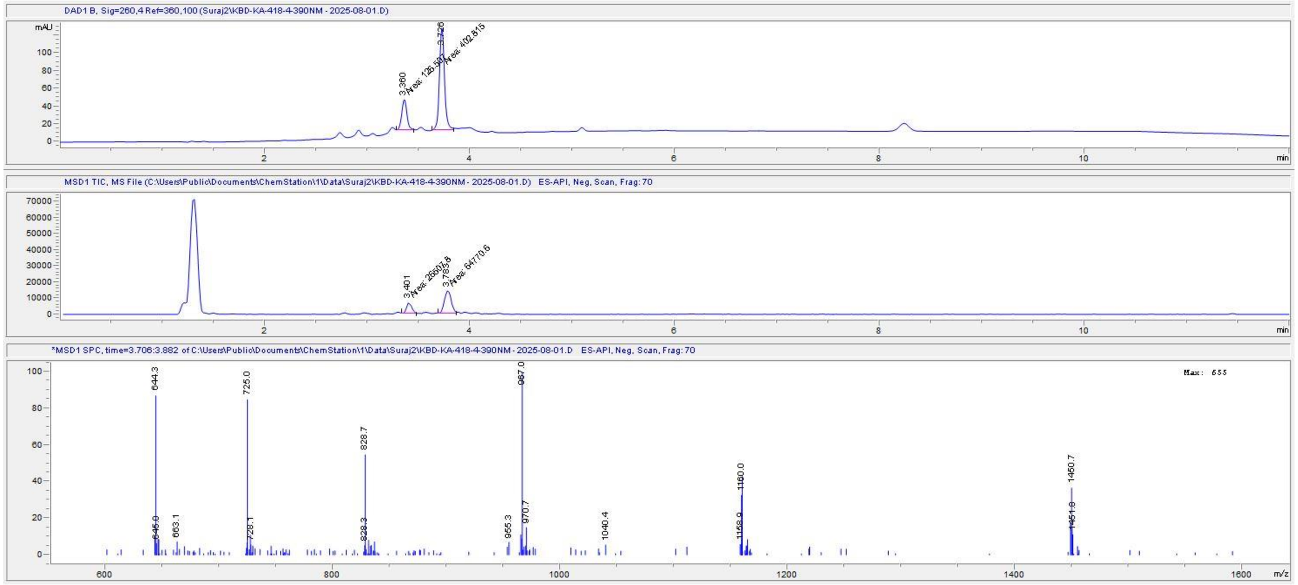

Figure S149. Analytical HPLC trace of **SRu-77** with HPLC method A. (Top) DAD chromatogram at 260 nm. (Middle) TIC chromatogram. (Bottom) Ionization of peak at 3.78 min. containing reaction product.

Decomplexation of SRu-77 to obtain product 77: Under an ambient atmosphere, the DNA-conjugate **SRu-77** stock solution in water (c = 0.10 mM, 20 µL) was irradiated with a 390 nm (40 W) Kessil lamp for 1 hours, while maintaining the temperature at approximately 30 °C through cooling with a fan. To the reaction mixture was added the stock solution of NaCl in water (**SR-06**, 2.0 µL, c = 5.0 M, 10% volume of the total reaction volume), followed by cold ethanol (−20 °C, 66 µL) to precipitate the DNA conjugate **77**. The Eppendorf tube was placed in the freezer (−20 °C) for at least 1 hour, and then it was centrifuged at 4 °C and 11000 × g for at least 30 minutes. The supernatant was removed, the pellet dried under air and dissolved in Milli-Q water to obtain the purified DNA-conjugate **77**. Then, 1 µL of the above solution was diluted to 40 µL with water for LC–MS analysis. The yield of the DNA conjugate was calculated by measuring the integration of the peaks of the diode array detection (DAD) UV absorbance at 260 nm of the LC–MS trace, assuming complete DNA recovery and identical UV absorbance.

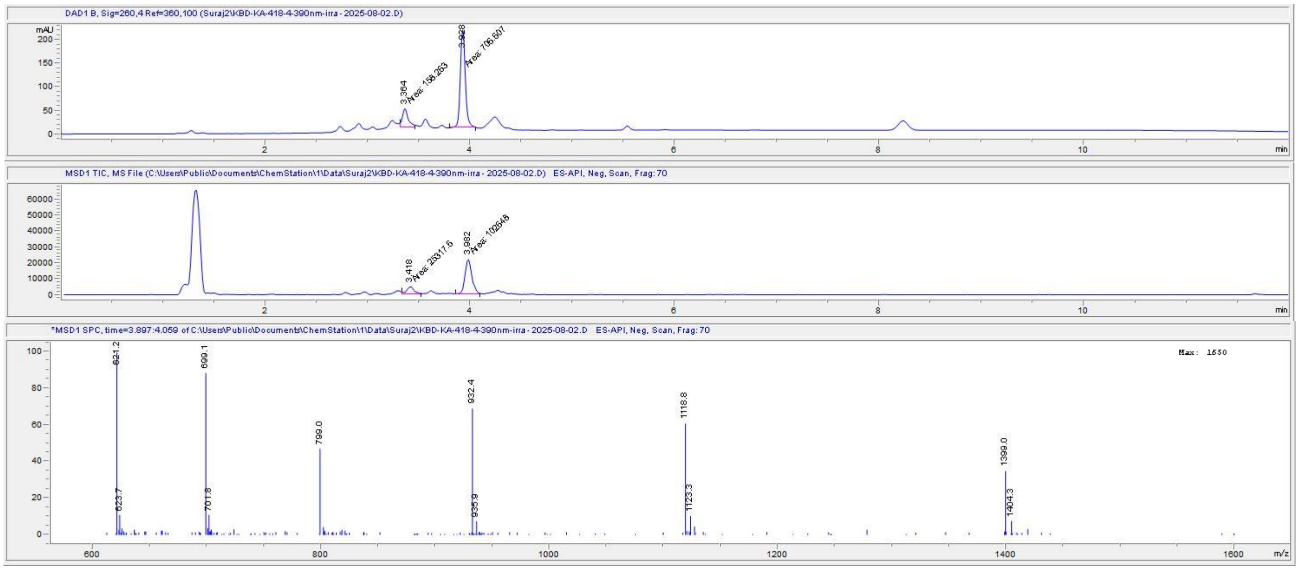

Figure S150. Analytical HPLC trace of **77** with HPLC method A. (Top) DAD chromatogram at 260 nm. (Middle) TIC chromatogram. (Bottom) Ionization of peak at 3.98 min. containing reaction product.

Synthesis of DNA-conjugate 78

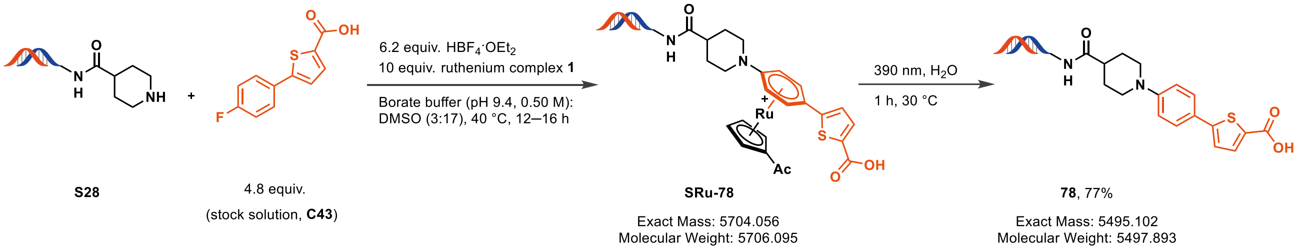

In situ formation of fluoroarene-ruthenium stock solution: Under an ambient atmosphere, a 1 mL glass GC vial equipped with a 6 mm Teflon-coated stirring bar was charged with ruthenium complex **1** (1.0 mg, 2.1 µmol, 1.0 equiv.) and then 100 µL of DMC was added to the reaction vial. In a separate Eppendorf tube was added a stock solution of $HBF_4 \cdot OEt_2$ (50 µL, c = 0.26 M, 13 µmol, 6.2 equiv.), followed by a stock solution of 5-(4-fluorophenyl)thiophene-2-carboxylic acid in DMC (50 µL, c = 0.20 M, 10 µmol, 4.8 equiv.). The contents of the Eppendorf tube was then transferred to the reaction vial. The vial was then closed with a screw cap and the resulting reaction mixture was heated at 80 °C for 2 hours. After 2 hours, the reaction mixture was cooled to 23 °C and the DMC was removed under a gentle stream of argon and 200 µL of DMSO was added to result in an in situ formed stock solution of arene-ruthenium complex **C43** in DMSO (200 µL, c = 0.01 M, assuming quantitative arene coordination to ruthenium).

At 20–25 °C, the stock solution of the amine-DNA conjugate **S28** (1.0 µL, c = 2.0 mM, 2.0 nmol, 1.0 equiv.) in water was added to a 1.5 mL Eppendorf tube, followed by sodium borate buffer (2.0 µL, pH 9.4, c = 0.50 M). To this mixture, 15 µL of DMSO was added and the solution was vortexed for 5 seconds. Next, the freshly prepared stock solution **C43** (2.0 µL, c = 0.01 M, 0.02 µmol, 10 equiv.) in DMSO was added. The resulting reaction mixture was vortexed for 5 seconds, transferred to a thermocycler at 40 °C, and incubated for 16

hours at 800 rpm to yield the DNA-conjugate **SRu-78**. To the reaction mixture was added the stock solution of NaCl in water (**SR-06** , 2.0 µL, c = 5.0 M, 10% volume of the total reaction volume), followed by cold ethanol (−20 °C, 66 µL) to precipitate the *N*-arylated ruthenium DNA conjugate **SRu-78**. The Eppendorf tube was placed in a freezer (−20 °C) for at least 1 hour, and then it was centrifuged at 4 °C and 11000 × g for at least 30 minutes. The supernatant was removed and the pellet was dried under air, then dissolved in 20 µL water to obtain the DNA-conjugate **SRu-78** (10 µL, c = 0.10 mM). Then, 1.0 µL of the above solution was diluted to 40 µL with water for LC–MS analysis.

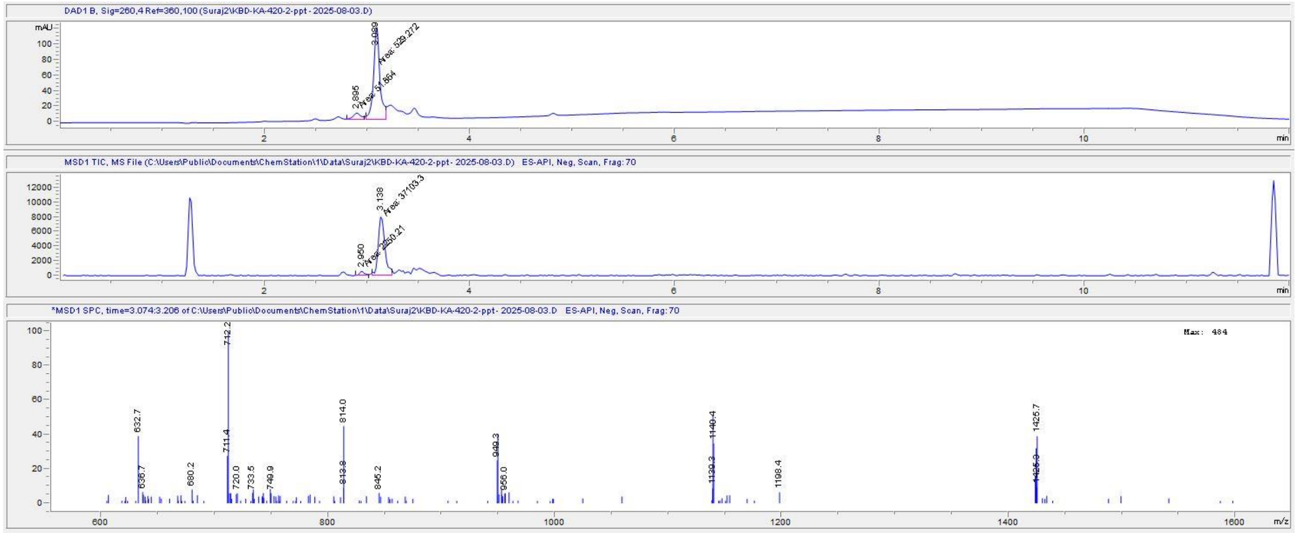

Figure S151. Analytical HPLC trace of **SRu-78** with HPLC method A. (Top) DAD chromatogram at 260 nm. (Middle) TIC chromatogram. (Bottom) Ionization of peak at 3.13 min. containing reaction product.

Decomplexation of SRu-78 to obtain product 78: Under an ambient atmosphere, the DNA-conjugate **SRu-78** stock solution in water (c = 0.10 mM, 20 µL) was irradiated with a 390 nm (40 W) Kessil lamp for 1 hours, while maintaining the temperature at approximately 30 °C through cooling with a fan. To the reaction mixture was added the stock solution of NaCl in water (**SR-06**, 2.0 µL, c = 5.0 M, 10% volume of the total reaction volume), followed by cold ethanol (−20 °C, 66 µL) to precipitate the DNA conjugate **78**. The Eppendorf tube was placed in the freezer (−20 °C) for at least 1 hour, and then it was centrifuged at 4 °C and 11000 × g for at least 30 minutes. The supernatant was removed, the pellet dried under air and dissolved in Milli-Q water to obtain the purified DNA-conjugate **78**. Then, 1 µL of the above solution was diluted to 40 µL with water for LC–MS analysis. The yield of the DNA conjugate was calculated by measuring the integration of the peaks of the diode array detection (DAD) UV absorbance at 260 nm of the LC–MS trace, assuming complete DNA recovery and identical UV absorbance.

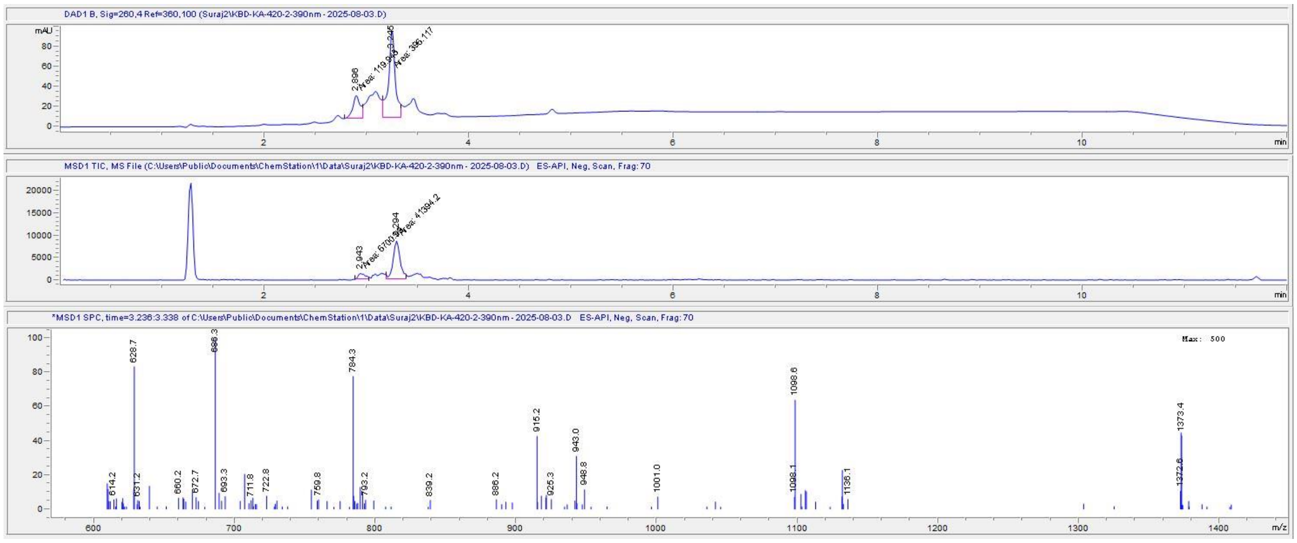

Figure S152. Analytical HPLC trace of **78** with HPLC method A. (Top) DAD chromatogram at 260 nm. (Middle) TIC chromatogram. (Bottom) Ionization of peak at 3.29 min. containing reaction product.

Synthesis of DNA-conjugate 79

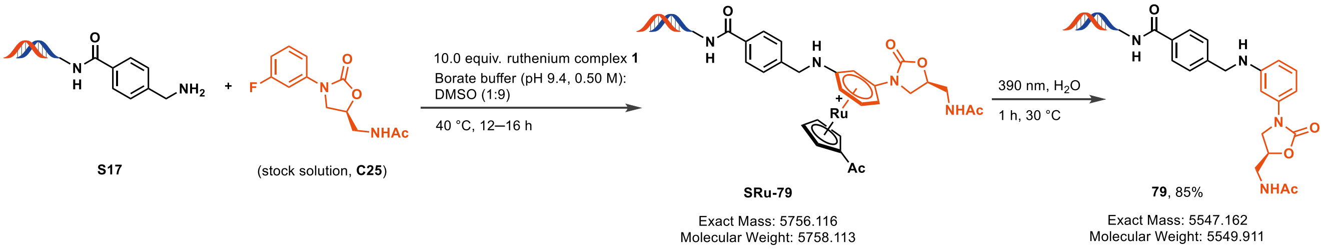

In situ formation of arene-ruthenium stock solution: Under an ambient atmosphere, a 1 mL glass GC vial equipped with a 6 mm Teflon-coated stirring bar was charged with ruthenium complex **1** (1.4 mg, 2.9 µmol, 1.0 equiv.). Next, a stock solution of (*S*)-N-((3-(3-fluorophenyl)-2-oxooxazolidin-5-yl)methyl)acetamide in DMC (294 µL, c = 0.10 M, 29 µmol, 10 equiv.) was added. The resulting reaction mixture was heated at 80 °C for 2 hours. After 2 hours, the reaction mixture was cooled to 23 °C. Next, the DMC was removed under a gentle stream of argon and 294 µL of DMSO were added to result an in situ formed stock solution of arene-ruthenium complex **C25** (294 µL, c = 0.01 M, assuming quantitative arene coordination to ruthenium).

At 20–25 °C, the stock solution of DNA-conjugate **S17** (1.0 µL, c = 2.0 mM, 2.0 nmol, 1.0 equiv.) in water was added to a 1.5 mL Eppendorf tube, followed by sodium borate buffer (1.0 µL, pH 9.4, c = 0.50 M). To this mixture, 16 µL of DMSO was added and the solution was vortexed for 5 seconds. Next, the freshly prepared stock solution **C25** (2.0 µL, c = 0.01 M, 0.02 µmol, 10 equiv.) in DMSO was added. The resulting reaction mixture was vortexed for 5 seconds, transferred to a thermocycler at 40 °C, and incubated for 16 hours at 800 rpm to yield the DNA-conjugate **SRu-79**. Next, the reaction mixture was diluted with 10 µL of Milli-Q water. To the reaction mixture was added the stock solution of NaCl in water (**SR-06** , 3.0 µL, c = 5.0 M, 10% volume of the total reaction volume), followed by cold ethanol (−20 °C, 99 µL) to precipitate the *N*-arylated ruthenium DNA conjugate **SRu-79**. The Eppendorf tube was placed in a freezer (−20 °C) for at least 1 hour,

and then it was centrifuged at 4 °C and 11000 × g for at least 30 minutes. The supernatant was removed and the pellet was dried under air, then dissolved in 20 µL water to obtain the DNA-conjugate **SRu-79** (20 µL, c = 0.10 mM). Then, 1.0 µL of the above solution was diluted to 40 µL with water for LC–MS analysis.

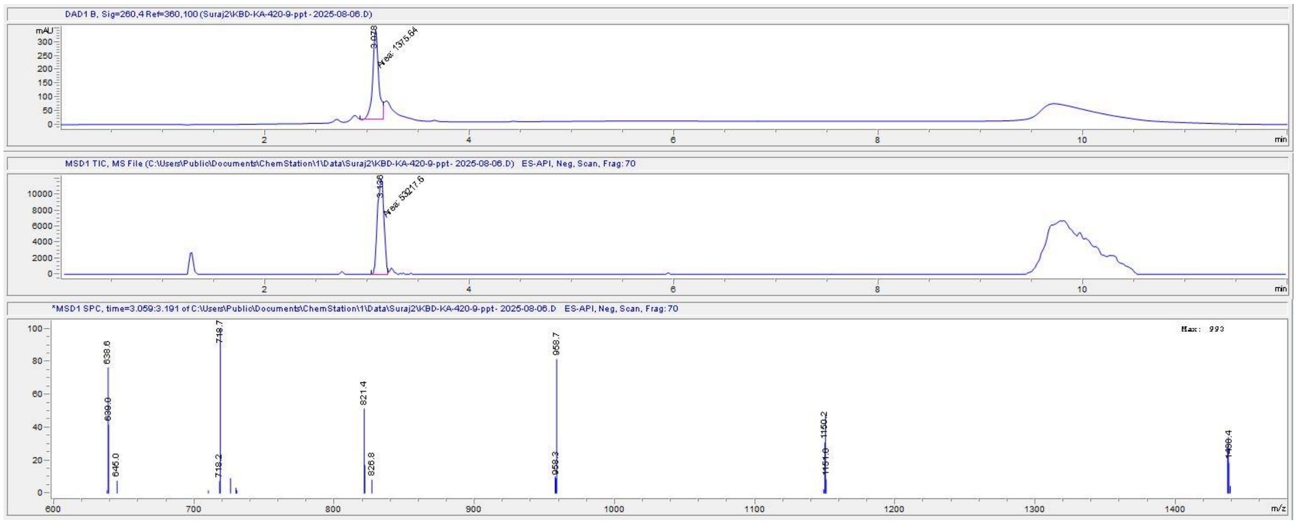

Figure S153. Analytical HPLC trace of **SRu-79** with HPLC method A. (Top) DAD chromatogram at 260 nm. (Middle) TIC chromatogram. (Bottom) Ionization of peak at 3.13 min. containing reaction product.

Decomplexation of SRu-79 to obtain product 79: Under an ambient atmosphere, the DNA-conjugate **SRu-79** stock solution in water (c = 0.10 mM, 20 µL) was irradiated with a 390 nm (40 W) Kessil lamp for 1 hours, while maintaining the temperature at approximately 30 °C through cooling with a fan. To the reaction mixture was added the stock solution of NaCl in water (**SR-06**, 2.0 µL, c = 5.0 M, 10% volume of the total reaction volume), followed by cold ethanol (−20 °C, 66 µL) to precipitate the DNA conjugate **79**. The Eppendorf tube was placed in the freezer (−20 °C) for at least 1 hour, and then it was centrifuged at 4 °C and 11000 × g for at least 30 minutes. The supernatant was removed, the pellet dried under air and dissolved in Milli-Q water to obtain the purified DNA-conjugate **79**. Then, 1 µL of the above solution was diluted to 40 µL with water for LC–MS analysis. The yield of the DNA conjugate was calculated by measuring the integration of the peaks of the diode array detection (DAD) UV absorbance at 260 nm of the LC–MS trace, assuming complete DNA recovery and identical UV absorbance.

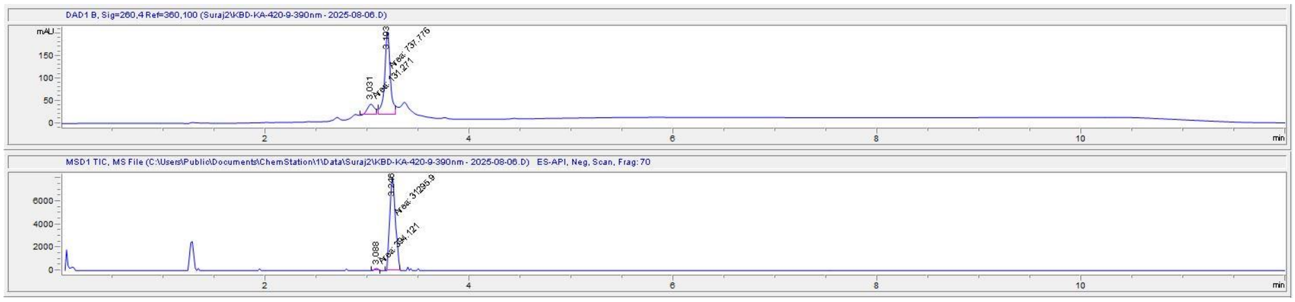

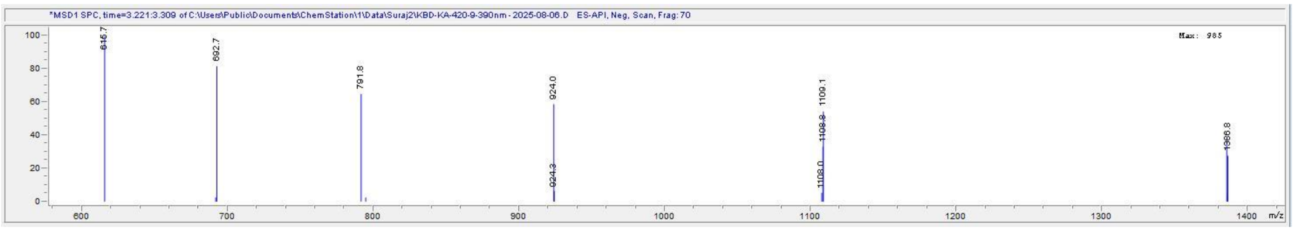

Figure S154. Analytical HPLC trace of **79** with HPLC method A. (Top) DAD chromatogram at 260 nm. (Middle) TIC chromatogram. (Bottom) Ionization of peak at 3.24 min. containing reaction product.

Synthesis of DNA-conjugate 80

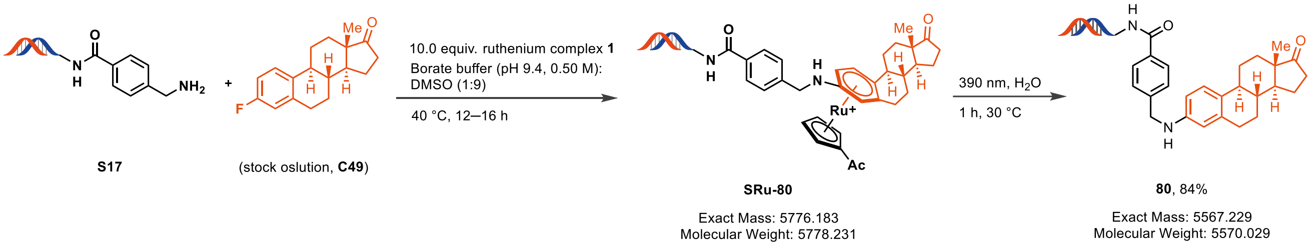

In situ formation of arene-ruthenium stock solution: Under an ambient atmosphere, a 1 mL glass GC vial equipped with a 6 mm Teflon-coated stirring bar was charged with ruthenium complex **1** (1.4 mg, 2.9 µmol, 1.0 equiv.). Next, a stock solution of the estrone derivative in DMC (294 µL, c = 0.10 M, 29 µmol, 10 equiv.) was added. The resulting reaction mixture was heated at 80 °C for 2 hours. After 2 hours, the reaction mixture was cooled to 23 °C. Next, the DMC was removed under a gentle stream of argon and 294 µL of DMSO were added to result an in situ formed stock solution of arene-ruthenium complex **C49** (294 µL, c = 0.01 M, assuming quantitative arene coordination to ruthenium).

At 20–25 °C, the stock solution of DNA-conjugate **S17** (1.0 µL, c = 2.0 mM, 2.0 nmol, 1.0 equiv.) in water was added to a 1.5 mL Eppendorf tube, followed by sodium borate buffer (1.0 µL, pH 9.4, c = 0.50 M). To this mixture, 16 µL of DMSO was added and the solution was vortexed for 5 seconds. Next, the freshly prepared stock solution **C49** (2.0 µL, c = 0.01 M, 0.02 µmol, 10 equiv.) in DMSO was added. The resulting reaction mixture was vortexed for 5 seconds, transferred to a thermocycler at 40 °C, and incubated for 16 hours at 800 rpm to yield the DNA-conjugate **SRu-80**. Next, the reaction mixture was diluted with 10 µL of Milli-Q water. To the reaction mixture was added the stock solution of NaCl in water (**SR-06** , 3.0 µL, c = 5.0 M, 10% volume of the total reaction volume), followed by cold ethanol (−20 °C, 99 µL) to precipitate the *N*-arylated ruthenium DNA conjugate **SRu-80**. The Eppendorf tube was placed in a freezer (−20 °C) for at least 1 hour, and then it was centrifuged at 4 °C and 11000 × g for at least 30 minutes. The supernatant was removed and the pellet was dried under air, then dissolved in 20 µL water to obtain the DNA-conjugate **SRu-80** (20 µL, c = 0.10 mM). Then, 1.0 µL of the above solution was diluted to 40 µL with water for LC–MS analysis.

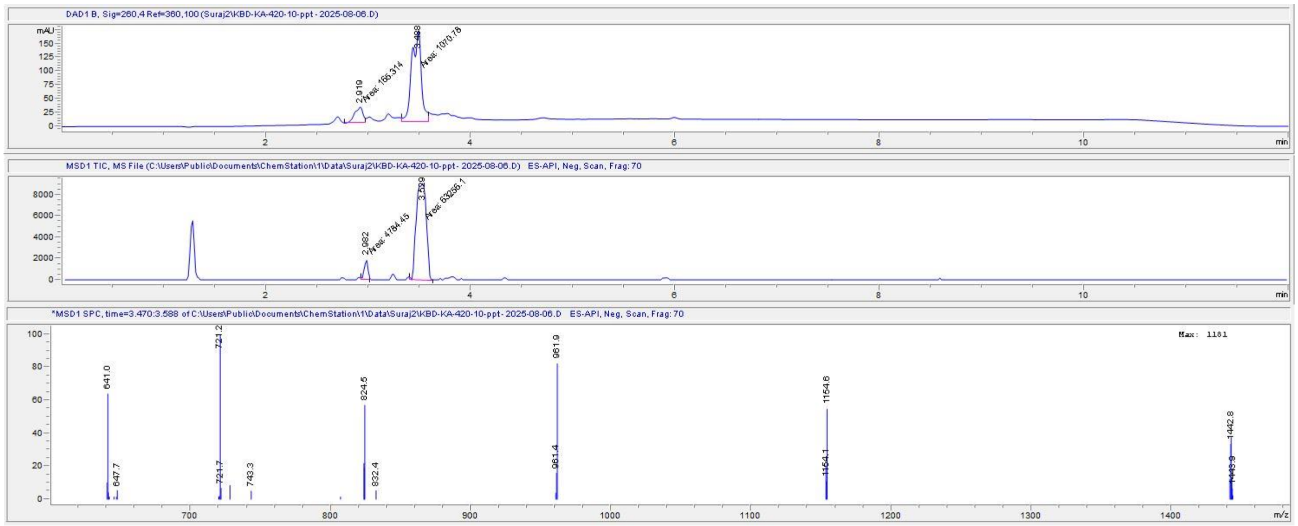

Figure S155. Analytical HPLC trace of **SRu-80** with HPLC method A. (Top) DAD chromatogram at 260 nm. (Middle) TIC chromatogram. (Bottom) Ionization of peak at 3.59 min. containing reaction product.

Decomplexation of SRu-80 to obtain product 80: Under an ambient atmosphere, the DNA-conjugate **SRu-80** stock solution in water (c = 0.10 mM, 20 µL) was irradiated with a 390 nm (40 W) Kessil lamp for 1 hours, while maintaining the temperature at approximately 30 °C through cooling with a fan. To the reaction mixture was added the stock solution of NaCl in water (**SR-06**, 2.0 µL, c = 5.0 M, 10% volume of the total reaction volume), followed by cold ethanol (−20 °C, 66 µL) to precipitate the DNA conjugate **80**. The Eppendorf tube was placed in the freezer (−20 °C) for at least 1 hour, and then it was centrifuged at 4 °C and 11000 × g for at least 30 minutes. The supernatant was removed, the pellet dried under air and dissolved in Milli-Q water to obtain the purified DNA-conjugate **80**. Then, 1 µL of the above solution was diluted to 40 µL with water for LC–MS analysis. The yield of the DNA conjugate was calculated by measuring the integration of the peaks of the diode array detection (DAD) UV absorbance at 260 nm of the LC–MS trace, assuming complete DNA recovery and identical UV absorbance.

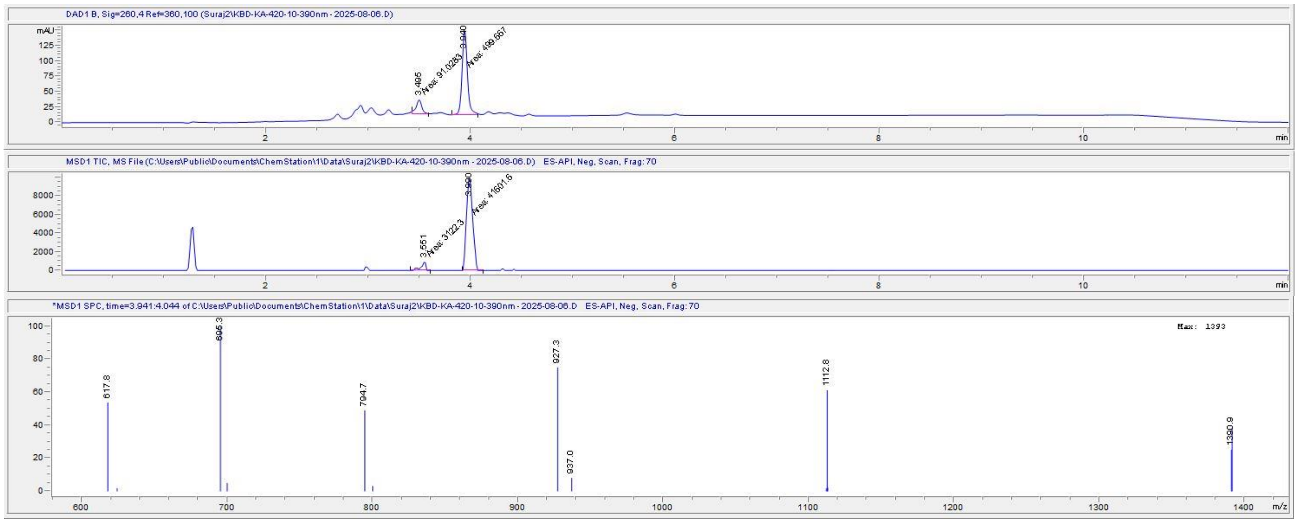

Figure S156. Analytical HPLC trace of **80** with HPLC method A. (Top) DAD chromatogram at 260 nm. (Middle) TIC chromatogram. (Bottom) Ionization of peak at 3.99 min. containing reaction product.

Synthesis of DNA-conjugate 81

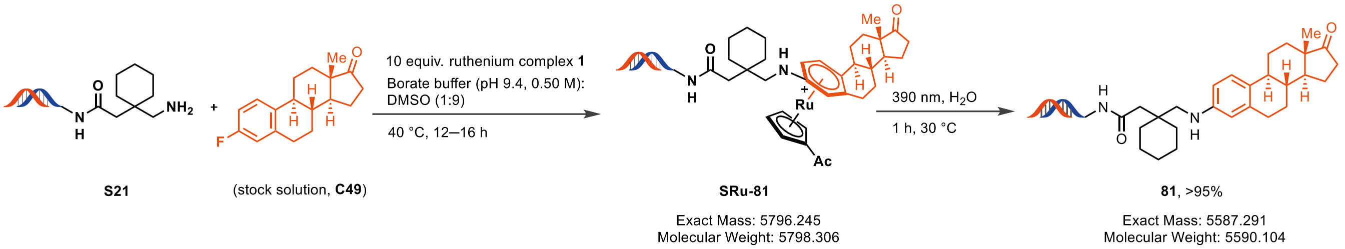

In situ formation of arene-ruthenium stock solution: Under an ambient atmosphere, a 1 mL glass GC vial equipped with a 6 mm Teflon-coated stirring bar was charged with ruthenium complex **1** (1.4 mg, 2.9 µmol, 1.0 equiv.). Next, a stock solution of the estrone derivative in DMC (294 µL, c = 0.10 M, 29 µmol, 10 equiv.) was added. The resulting reaction mixture was heated at 80 °C for 2 hours. After 2 hours, the reaction mixture was cooled to 23 °C. Next, the DMC was removed under a gentle stream of argon and 294 µL of DMSO were added to result an in situ formed stock solution of arene-ruthenium complex **C49** (294 µL, c = 0.01 M, assuming quantitative arene coordination to ruthenium).

At 20–25 °C, the stock solution of DNA-conjugate **S21** (1.0 µL, c = 2.0 mM, 2.0 nmol, 1.0 equiv.) in water was added to a 1.5 mL Eppendorf tube, followed by sodium borate buffer (1.0 µL, pH 9.4, c = 0.50 M). To this mixture, 16 µL of DMSO was added and the solution was vortexed for 5 seconds. Next, the freshly prepared stock solution **C49** (2.0 µL, c = 0.01 M, 0.02 µmol, 10 equiv.) in DMSO was added. The resulting reaction mixture was vortexed for 5 seconds, transferred to a thermocycler at 40 °C, and incubated for 16 hours at 800 rpm to yield the DNA-conjugate **SRu-81**. Next, the reaction mixture was diluted with 10 µL of Milli-Q water. To the reaction mixture was added the stock solution of NaCl in water (**SR-06** , 3.0 µL, c = 5.0 M, 10% volume of the total reaction volume), followed by cold ethanol (−20 °C, 99 µL) to precipitate the *N*-arylated ruthenium DNA conjugate **SRu-81**. The Eppendorf tube was placed in a freezer (−20 °C) for at least 1 hour, and then it was centrifuged at 4 °C and 11000 × g for at least 30 minutes. The supernatant was removed and the pellet was dried under air, then dissolved in 20 µL water to obtain the DNA-conjugate **SRu-81** (20 µL, c = 0.10 mM). Then, 1.0 µL of the above solution was diluted to 40 µL with water for LC–MS analysis.

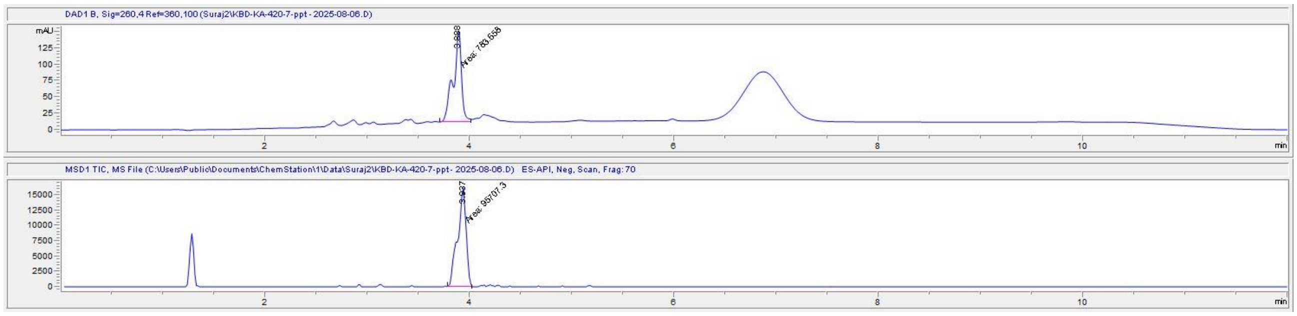

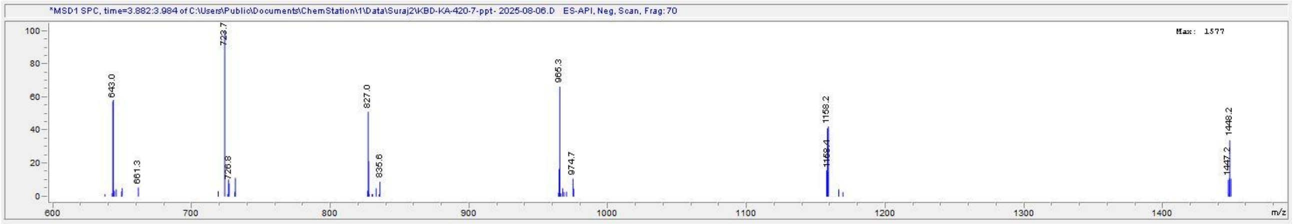

Figure S157. Analytical HPLC trace of **SRu-81** with HPLC method A. (Top) DAD chromatogram at 260 nm. (Middle) TIC chromatogram. (Bottom) Ionization of peak at 3.94 min. containing reaction product.

Decomplexation of SRu-81 to obtain product 81: Under an ambient atmosphere, the DNA-conjugate **SRu-81** stock solution in water (c = 0.10 mM, 20 µL) was irradiated with a 390 nm (40 W) Kessil lamp for 1 hours, while maintaining the temperature at approximately 30 °C through cooling with a fan. To the reaction mixture was added the stock solution of NaCl in water (**SR-06**, 2.0 µL, c = 5.0 M, 10% volume of the total reaction volume), followed by cold ethanol (−20 °C, 66 µL) to precipitate the DNA conjugate **81**. The Eppendorf tube was placed in the freezer (−20 °C) for at least 1 hour, and then it was centrifuged at 4 °C and 11000 × g for at least 30 minutes. The supernatant was removed, the pellet dried under air and dissolved in Milli-Q water to obtain the purified DNA-conjugate **81**. Then, 1 µL of the above solution was diluted to 40 µL with water for LC–MS analysis. The yield of the DNA conjugate was calculated by measuring the integration of the peaks of the diode array detection (DAD) UV absorbance at 260 nm of the LC–MS trace, assuming complete DNA recovery and identical UV absorbance.

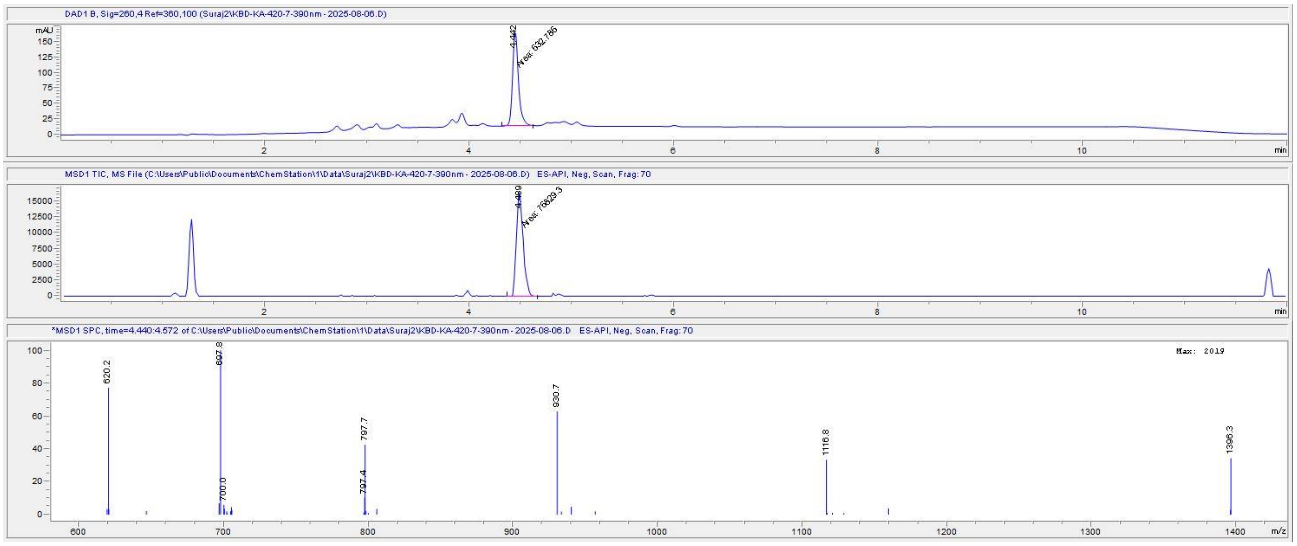

Figure S158. Analytical HPLC trace of **81** with HPLC method A. (Top) DAD chromatogram at 260 nm. (Middle) TIC chromatogram. (Bottom) Ionization of peak at 4.49 min. containing reaction product.

Synthesis of DNA-conjugate 82

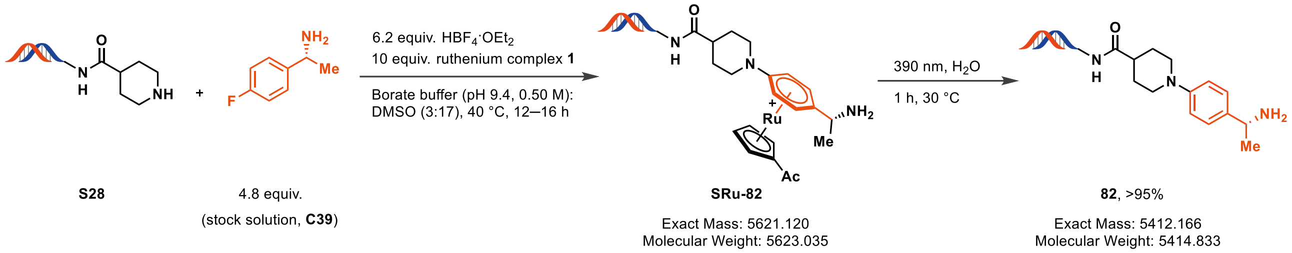

In situ formation of fluoroarene-ruthenium stock solution: Under an ambient atmosphere, a 1 mL glass GC vial equipped with a 6 mm Teflon-coated stirring bar was charged with ruthenium complex **1** (1.0 mg, 2.1 µmol, 1.0 equiv.) and then 100 µL of DMC was added to the reaction vial. In a separate Eppendorf tube was added a stock solution of $HBF_4{\cdot}OEt_2$ (50 µL, c = 0.26 M, 13 µmol, 6.2 equiv.), followed by a stock solution of (*R*)-1-(4-fluorophenyl)ethan-1-amine in DMC (50 µL, c = 0.20 M, 10 µmol, 4.8 equiv.). The contents of the Eppendorf tube was then transferred to the reaction vial. The vial was then closed with a screw cap and the resulting reaction mixture was heated at 80 °C for 2 hours. After 2 hours, the reaction mixture was cooled to 23 °C and the DMC was removed under a gentle stream of argon and 200 µL of DMSO was added to result in an in situ formed stock solution of arene-ruthenium complex **C39** in DMSO (200 µL, c = 0.01 M, assuming quantitative arene coordination to ruthenium).

At 20–25 °C, the stock solution of the amine-DNA conjugate **S28** (1.0 µL, c = 2.0 mM, 2.0 nmol, 1.0 equiv.) in water was added to a 1.5 mL Eppendorf tube, followed by sodium borate buffer (2.0 µL, pH 9.4, c = 0.50 M). To this mixture, 15 µL of DMSO was added and the solution was vortexed for 5 seconds. Next, the freshly prepared stock solution **C39** (2.0 µL, c = 0.01 M, 0.02 µmol, 10 equiv.) in DMSO was added. The resulting reaction mixture was vortexed for 5 seconds, transferred to a thermocycler at 40 °C, and incubated for 16 hours at 800 rpm to yield the DNA-conjugate **SRu-82**. To the reaction mixture was added the stock solution of NaCl in water (**SR-06** , 2.0 µL, c = 5.0 M, 10% volume of the total reaction volume), followed by cold ethanol (−20 °C, 66 µL) to precipitate the *N*-arylated ruthenium DNA conjugate **SRu-82**. The Eppendorf tube was placed in a freezer (−20 °C) for at least 1 hour, and then it was centrifuged at 4 °C and 11000 × g for at least 30 minutes. The supernatant was removed and the pellet was dried under air, then dissolved in 20 µL water to obtain the DNA-conjugate **SRu-82** (10 µL, c = 0.10 mM). Then, 1.0 µL of the above solution was diluted to 40 µL with water for LC–MS analysis.

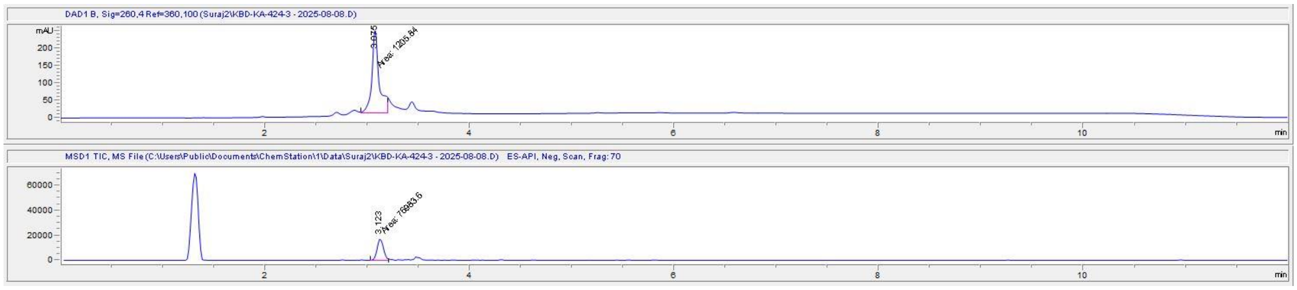

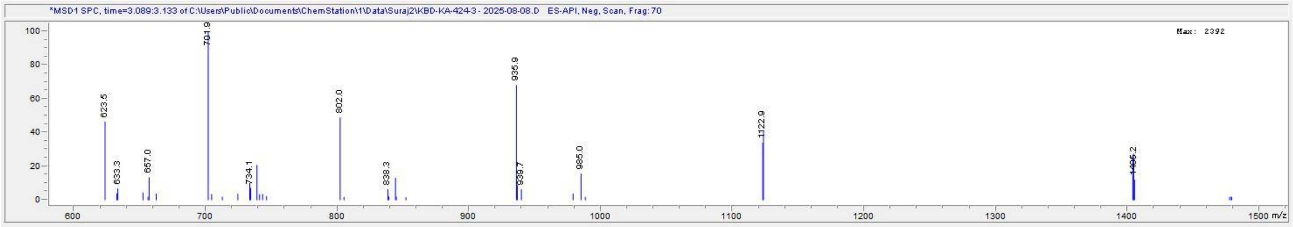

Figure S159. Analytical HPLC trace of **Ru-82** with HPLC method A. (Top) DAD chromatogram at 260 nm. (Middle) TIC chromatogram. (Bottom) Ionization of peak at 3.12 min. containing reaction product.

Decomplexation of SRu-82 to obtain product 82: Under an ambient atmosphere, the DNA-conjugate **SRu-82** stock solution in water (c = 0.10 mM, 20 µL) was irradiated with a 390 nm (40 W) Kessil lamp for 1 hours, while maintaining the temperature at approximately 30 °C through cooling with a fan. To the reaction mixture was added the stock solution of NaCl in water (**SR-06**, 2.0 µL, c = 5.0 M, 10% volume of the total reaction volume), followed by cold ethanol (−20 °C, 66 µL) to precipitate the DNA conjugate **82**. The Eppendorf tube was placed in the freezer (−20 °C) for at least 1 hour, and then it was centrifuged at 4 °C and 11000 × g for at least 30 minutes. The supernatant was removed, the pellet dried under air and dissolved in Milli-Q water to obtain the purified DNA-conjugate **82**. Then, 1 µL of the above solution was diluted to 40 µL with water for LC–MS analysis. The yield of the DNA conjugate was calculated by measuring the integration of the peaks of the diode array detection (DAD) UV absorbance at 260 nm of the LC–MS trace, assuming complete DNA recovery and identical UV absorbance.

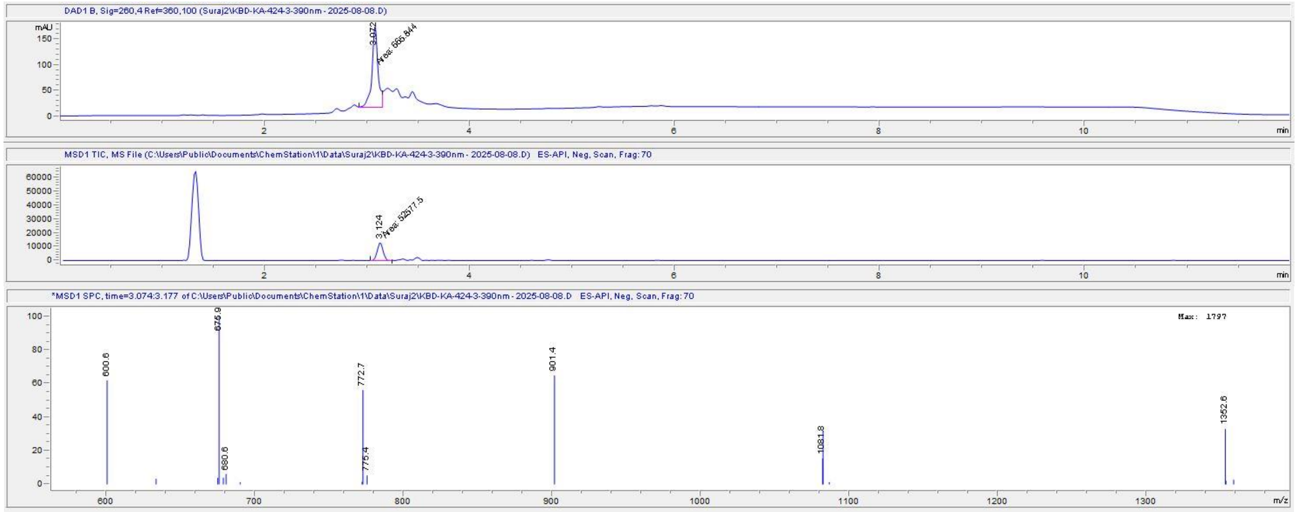

Figure S160. Analytical HPLC trace of **82** with HPLC method A. (Top) DAD chromatogram at 260 nm. (Middle) TIC chromatogram. (Bottom) Ionization of peak at 3.12 min. containing reaction product.

Synthesis of DNA-conjugate 83

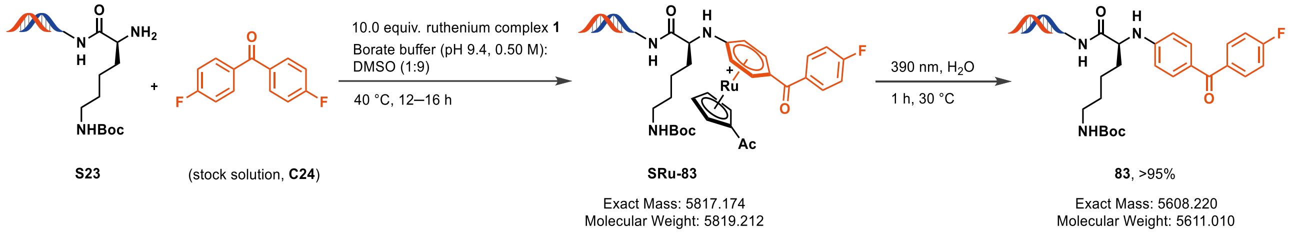

In situ formation of arene-ruthenium stock solution: Under an ambient atmosphere, a 1 mL glass GC vial equipped with a 6 mm Teflon-coated stirring bar was charged with ruthenium complex **1** (1.4 mg, 2.9 µmol, 1.0 equiv.). Next, a stock solution of bis(4-fluorophenyl)methanone in DMC (294 µL, c = 0.10 M, 29 µmol, 10 equiv.) was added. The resulting reaction mixture was heated at 80 °C for 2 hours. After 2 hours, the reaction mixture was cooled to 23 °C. Next, the DMC was removed under a gentle stream of argon and 294 µL of DMSO were added to result an in situ formed stock solution of arene-ruthenium complex **C24** (294 µL, c = 0.01 M, assuming quantitative arene coordination to ruthenium).

At 20–25 °C, the stock solution of DNA-conjugate **S23** (1.0 µL, c = 2.0 mM, 2.0 nmol, 1.0 equiv.) in water was added to a 1.5 mL Eppendorf tube, followed by sodium borate buffer (1.0 µL, pH 9.4, c = 0.50 M). To this mixture, 16 µL of DMSO was added and the solution was vortexed for 5 seconds. Next, the freshly prepared stock solution **C24** (2.0 µL, c = 0.01 M, 0.02 µmol, 10 equiv.) in DMSO was added. The resulting reaction mixture was vortexed for 5 seconds, transferred to a thermocycler at 40 °C, and incubated for 16 hours at 800 rpm to yield the DNA-conjugate **SRu-83**. Next, the reaction mixture was diluted with 10 µL of Milli-Q water. To the reaction mixture was added the stock solution of NaCl in water (**SR-06** , 3.0 µL, c = 5.0 M, 10% volume of the total reaction volume), followed by cold ethanol (−20 °C, 99 µL) to precipitate the *N*-arylated ruthenium DNA conjugate **SRu-83**. The Eppendorf tube was placed in a freezer (−20 °C) for at least 1 hour, and then it was centrifuged at 4 °C and 11000 × g for at least 30 minutes. The supernatant was removed and the pellet was dried under air, then dissolved in 20 µL water to obtain the DNA-conjugate **SRu-83** (20 µL, c = 0.10 mM). Then, 1.0 µL of the above solution was diluted to 40 µL with water for LC–MS analysis.

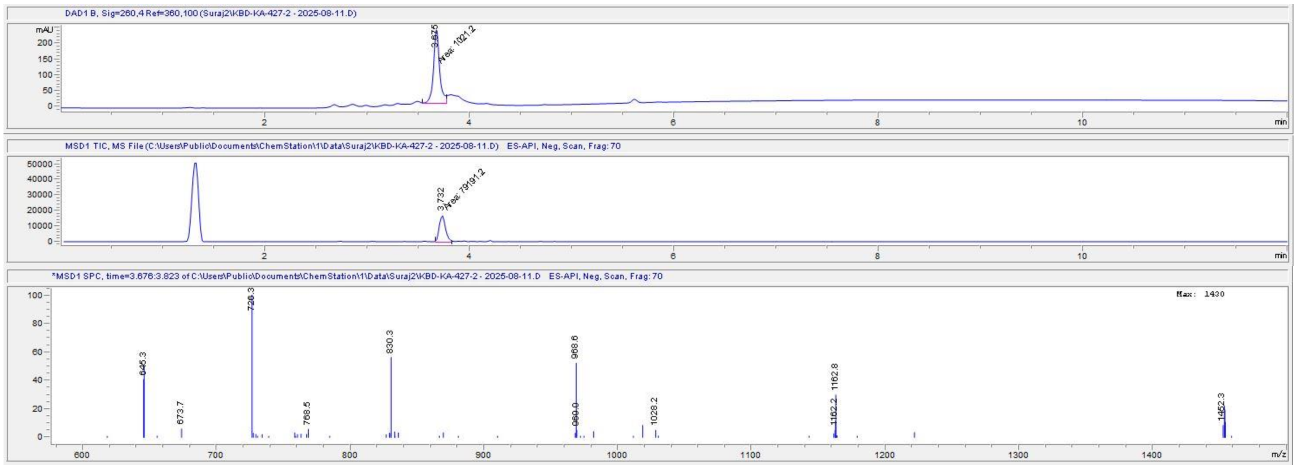

Figure S161. Analytical HPLC trace of **SRu-83** with HPLC method A. (Top) DAD chromatogram at 260 nm. (Middle) TIC chromatogram. (Bottom) Ionization of peak at 3.73 min. containing reaction product.

Decomplexation of SRu-83 to obtain product 83: Under an ambient atmosphere, the DNA-conjugate **SRu-83** stock solution in water (c = 0.10 mM, 20 µL) was irradiated with a 390 nm (40 W) Kessil lamp for 1 hours, while maintaining the temperature at approximately 30 °C through cooling with a fan. To the reaction mixture was added the stock solution of NaCl in water (**SR-06**, 2.0 µL, c = 5.0 M, 10% volume of the total reaction volume), followed by cold ethanol (−20 °C, 66 µL) to precipitate the DNA conjugate **83**. The Eppendorf tube was placed in the freezer (−20 °C) for at least 1 hour, and then it was centrifuged at 4 °C and 11000 × g for at least 30 minutes. The supernatant was removed, the pellet dried under air and dissolved in Milli-Q water to obtain the purified DNA-conjugate **83**. Then, 1 µL of the above solution was diluted to 40 µL with water for LC–MS analysis. The yield of the DNA conjugate was calculated by measuring the integration of the peaks of the diode array detection (DAD) UV absorbance at 260 nm of the LC–MS trace, assuming complete DNA recovery and identical UV absorbance.

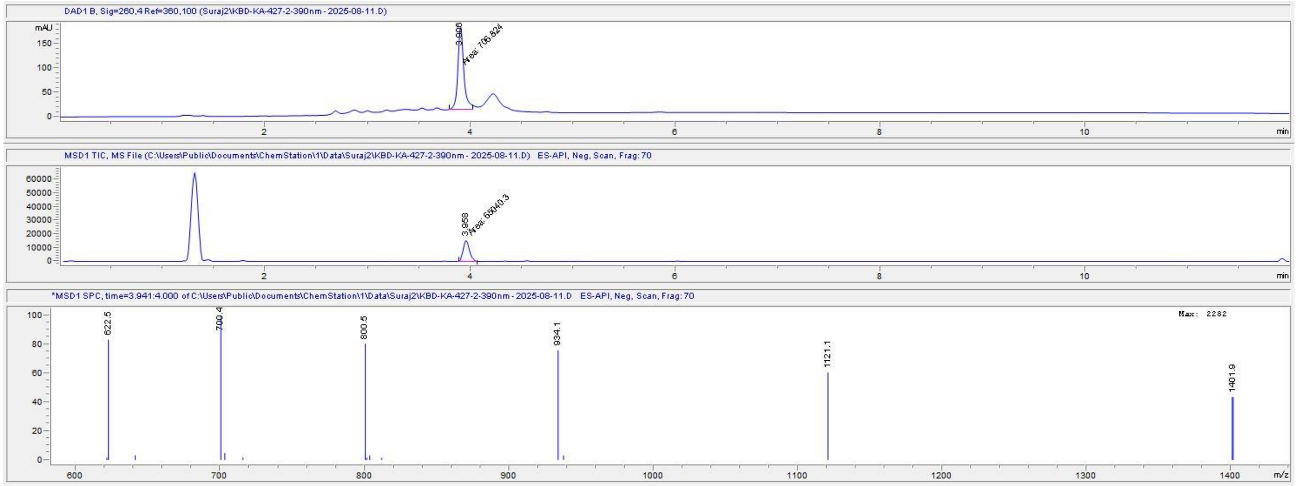

Figure S162. Analytical HPLC trace of **83** with HPLC method A. (Top) DAD chromatogram at 260 nm. (Middle) TIC chromatogram. (Bottom) Ionization of peak at 3.95 min. containing reaction product

Synthesis of DNA-conjugate 84

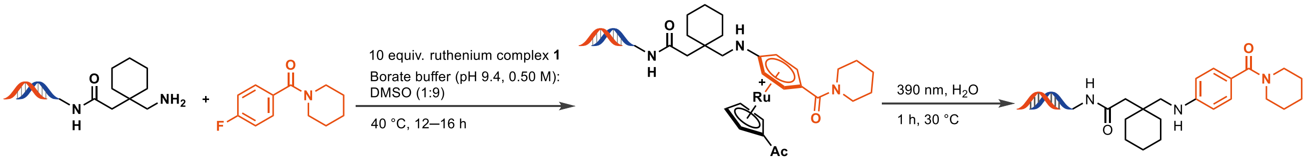

S21 (stock solution, **C26**) **SRu-84**, >95% **84**, >95%

Exact Mass: 5731.194
Molecular Weight: 5733.191

Exact Mass: 5522.240
Molecular Weight: 5514.989

In situ formation of arene-ruthenium stock solution: Under an ambient atmosphere, a 1 mL glass GC vial equipped with a 6 mm Teflon-coated stirring bar was charged with ruthenium complex **1** (1.4 mg, 2.9 µmol, 1.0 equiv.). Next, a stock solution of (4-fluorophenyl)(piperidin-1-yl)methanone in DMC (294 µL, c = 0.10 M,

29 µmol, 10 equiv.) was added. The resulting reaction mixture was heated at 80 °C for 2 hours. After 2 hours, the reaction mixture was cooled to 23 °C. Next, the DMC was removed under a gentle stream of argon and 294 µL of DMSO were added to result an in situ formed stock solution of arene-ruthenium complex **C26** (294 µL, c = 0.01 M, assuming quantitative arene coordination to ruthenium).

At 20–25 °C, the stock solution of DNA-conjugate **S21** (1.0 µL, c = 2.0 mM, 2.0 nmol, 1.0 equiv.) in water was added to a 1.5 mL Eppendorf tube, followed by sodium borate buffer (1.0 µL, pH 9.4, c = 0.50 M). To this mixture, 16 µL of DMSO was added and the solution was vortexed for 5 seconds. Next, the freshly prepared stock solution **C26** (2.0 µL, c = 0.01 M, 0.02 µmol, 10 equiv.) in DMSO was added. The resulting reaction mixture was vortexed for 5 seconds, transferred to a thermocycler at 40 °C, and incubated for 16 hours at 800 rpm to yield the DNA-conjugate **SRu-84**. Next, the reaction mixture was diluted with 10 µL of Milli-Q water. To the reaction mixture was added the stock solution of NaCl in water (**SR-06** , 3.0 µL, c = 5.0 M, 10% volume of the total reaction volume), followed by cold ethanol (−20 °C, 99 µL) to precipitate the *N*-arylated ruthenium DNA conjugate **SRu-84**. The Eppendorf tube was placed in a freezer (−20 °C) for at least 1 hour, and then it was centrifuged at 4 °C and 11000 × g for at least 30 minutes. The supernatant was removed and the pellet was dried under air, then dissolved in 20 µL water to obtain the DNA-conjugate **SRu-84** (20 µL, c = 0.10 mM). Then, 1.0 µL of the above solution was diluted to 40 µL with water for LC–MS analysis.

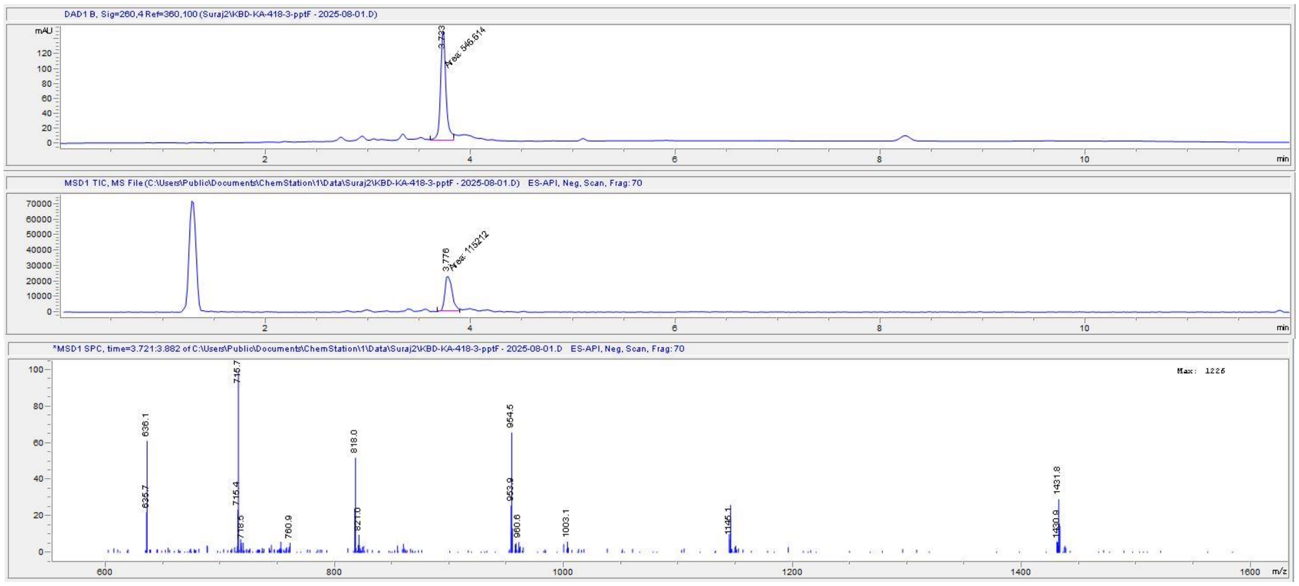

Figure S163. Analytical HPLC trace of **SRu-84** with HPLC method A. (Top) DAD chromatogram at 260 nm. (Middle) TIC chromatogram. (Bottom) Ionization of peak at 3.78 min. containing reaction product.

Decomplexation of SRu-84 to obtain product 84: Under an ambient atmosphere, the DNA-conjugate **SRu-84** stock solution in water (c = 0.10 mM, 20 µL) was irradiated with a 390 nm (40 W) Kessil lamp for 1 hours, while maintaining the temperature at approximately 30 °C through cooling with a fan. To the reaction mixture was added the stock solution of NaCl in water (**SR-06**, 2.0 µL, c = 5.0 M, 10% volume of the total reaction volume), followed by cold ethanol (−20 °C, 66 µL) to precipitate the DNA conjugate **84**. The Eppendorf tube was placed in the freezer (−20 °C) for at least 1 hour, and then it was centrifuged at 4 °C and 11000 × g for at

least 30 minutes. The supernatant was removed, the pellet dried under air and dissolved in Milli-Q water to obtain the purified DNA-conjugate **84**. Then, 1 µL of the above solution was diluted to 40 µL with water for LC–MS analysis. The yield of the DNA conjugate was calculated by measuring the integration of the peaks of the diode array detection (DAD) UV absorbance at 260 nm of the LC–MS trace, assuming complete DNA recovery and identical UV absorbance.

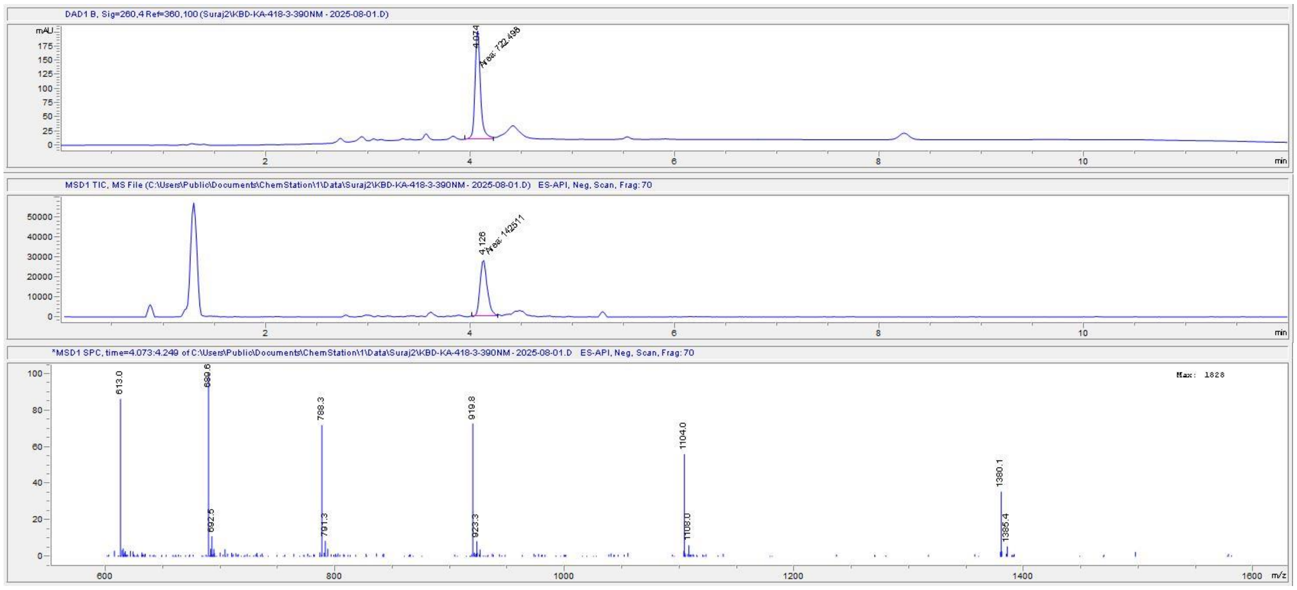

Figure S164. Analytical HPLC trace of **84** with HPLC method A. (Top) DAD chromatogram at 260 nm. (Middle) TIC chromatogram. (Bottom) Ionization of peak at 4.12 min. containing reaction product.

Synthesis of DNA-conjugate 85

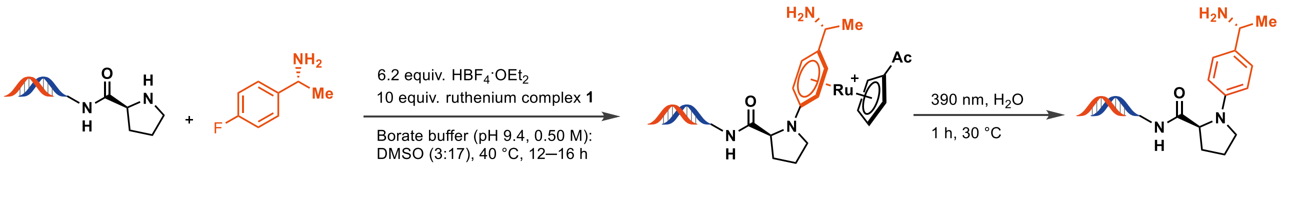

S29 4.8 equiv. (stock solution, **C39**) **SRu-85** Exact Mass: 5607.105 Molecular Weight: 5609.008 **85**, 84% Exact Mass: 5398.151 Molecular Weight: 5400.806

In situ formation of fluoroarene-ruthenium stock solution: Under an ambient atmosphere, a 1 mL glass GC vial equipped with a 6 mm Teflon-coated stirring bar was charged with ruthenium complex **1** (1.0 mg, 2.1 µmol, 1.0 equiv.) and then 100 µL of DMC was added to the reaction vial. In a separate Eppendorf tube was added a stock solution of $HBF_4{\cdot}OEt_2$ (50 µL, c = 0.26 M, 13 µmol, 6.2 equiv.), followed by a stock solution of (*R*)-1-(4-fluorophenyl)ethan-1-amine in DMC (50 µL, c = 0.20 M, 10 µmol, 4.8 equiv.). The contents of the Eppendorf tube was then transferred to the reaction vial. The vial was then closed with a screw cap and the resulting reaction mixture was heated at 80 °C for 2 hours. After 2 hours, the reaction mixture was cooled to 23 °C and the DMC was removed under a gentle stream of argon and 200 µL of DMSO was added to result

in an in situ formed stock solution of arene-ruthenium complex **C39** in DMSO (200 µL, c = 0.01 M, assuming quantitative arene coordination to ruthenium).

At 20–25 °C, the stock solution of the amine-DNA conjugate **S29** (1.0 µL, c = 2.0 mM, 2.0 nmol, 1.0 equiv.) in water was added to a 1.5 mL Eppendorf tube, followed by sodium borate buffer (2.0 µL, pH 9.4, c = 0.50 M). To this mixture, 15 µL of DMSO was added and the solution was vortexed for 5 seconds. Next, the freshly prepared stock solution **C39** (2.0 µL, c = 0.01 M, 0.02 µmol, 10 equiv.) in DMSO was added. The resulting reaction mixture was vortexed for 5 seconds, transferred to a thermocycler at 40 °C, and incubated for 16 hours at 800 rpm to yield the DNA-conjugate **SRu-85**. To the reaction mixture was added the stock solution of NaCl in water (**SR-06** , 2.0 µL, c = 5.0 M, 10% volume of the total reaction volume), followed by cold ethanol (−20 °C, 66 µL) to precipitate the *N*-arylated ruthenium DNA conjugate **SRu-85**. The Eppendorf tube was placed in a freezer (−20 °C) for at least 1 hour, and then it was centrifuged at 4 °C and 11000 × g for at least 30 minutes. The supernatant was removed and the pellet was dried under air, then dissolved in 20 µL water to obtain the DNA-conjugate **SRu-85** (10 µL, c = 0.10 mM). Then, 1.0 µL of the above solution was diluted to 40 µL with water for LC–MS analysis.

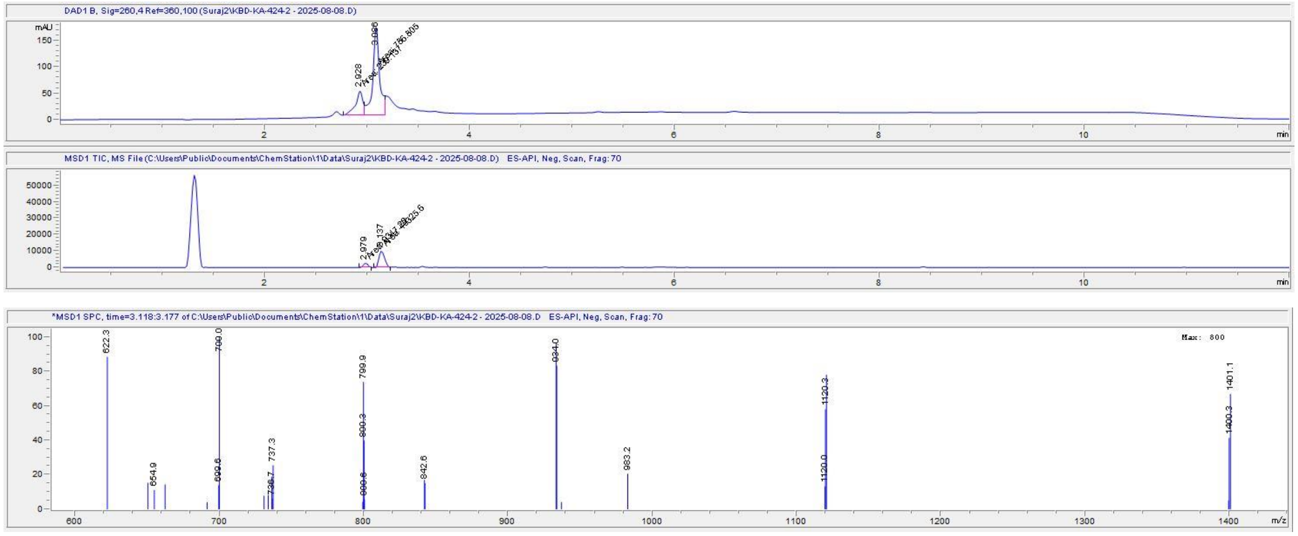

Figure S165. Analytical HPLC trace of **SRu-85** with HPLC method A. (Top) DAD chromatogram at 260 nm. (Middle) TIC chromatogram. (Bottom) Ionization of peak at 3.14 min. containing reaction product.

Decomplexation of SRu-85 to obtain product 85: Under an ambient atmosphere, the DNA-conjugate **SRu-85** stock solution in water (c = 0.10 mM, 20 µL) was irradiated with a 390 nm (40 W) Kessil lamp for 1 hours, while maintaining the temperature at approximately 30 °C through cooling with a fan. To the reaction mixture was added the stock solution of NaCl in water (**SR-06**, 2.0 µL, c = 5.0 M, 10% volume of the total reaction volume), followed by cold ethanol (−20 °C, 66 µL) to precipitate the DNA conjugate **85**. The Eppendorf tube was placed in the freezer (−20 °C) for at least 1 hour, and then it was centrifuged at 4 °C and 11000 × g for at least 30 minutes. The supernatant was removed, the pellet dried under air and dissolved in Milli-Q water to obtain the purified DNA-conjugate **85**. Then, 1 µL of the above solution was diluted to 40 µL with water for LC–MS analysis. The yield of the DNA conjugate was calculated by measuring the integration of the peaks of

the diode array detection (DAD) UV absorbance at 260 nm of the LC–MS trace, assuming complete DNA recovery and identical UV absorbance.

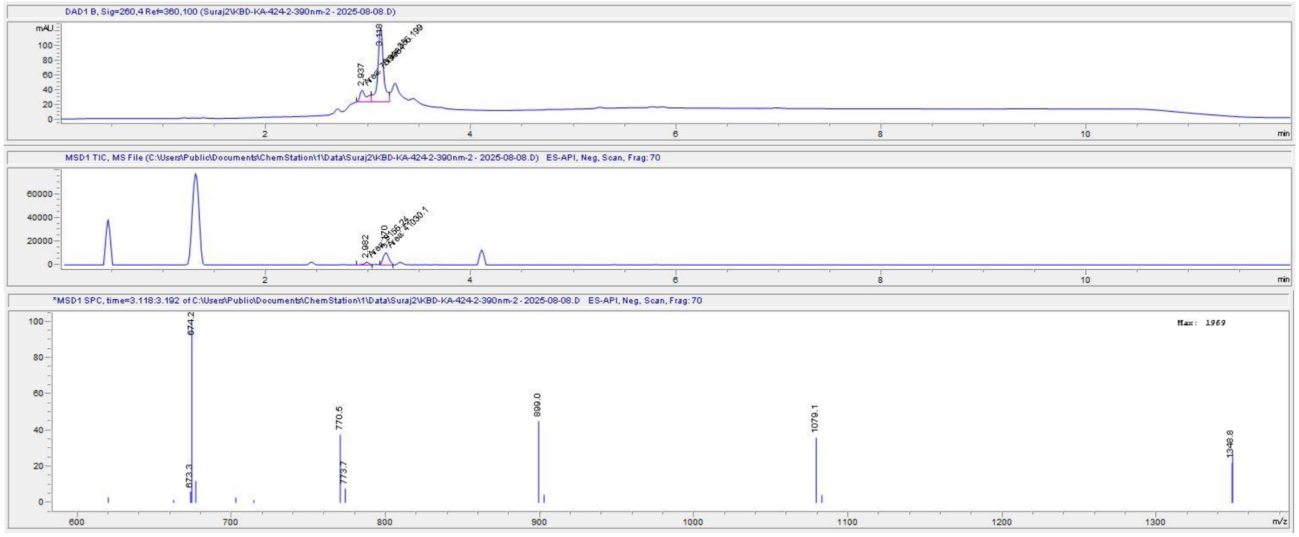

Figure S166. Analytical HPLC trace of **85** with HPLC method A. (Top) DAD chromatogram at 260 nm. (Middle) TIC chromatogram. (Bottom) Ionization of peak at 3.17 min. containing reaction product.

COMPUTATIONAL STUDIES

Electrophilicity of ruthenium complexes

Computational details

All geometry optimizations and electronic energy calculations were performed in gas phase using the ORCA program package, version 6.0.2[7–8]. Geometry optimizations were carried out at the PBE0[9]-D4[10]/x2c-TZVPall[11] level with the x2c Hamiltonian[12]. Electronic energies were calculated at the DLPNO-CCSD(T)[7]/x2c-TZVPall level with TightPNO settings and x2c Hamiltonian.

The Multiwfn 3.7[14] program was used to obtain the electrophilicity data from the ORCA single point electronic energy output files. First, ORCA output files for the same species with varying number of electrons (N, N+1, N−1, N−2) were converted to wfn files which are read by the Multiwfn program to analyze the different electronic states i.e. cationic, neutral and anionic states. Multiwfn produces a CDFT.txt file which contains the local electrophilicities as well as the global electrophilicity index of a species.

Computational data

Electrophilicity values

We present the electrophilicity data listed in the CDFT.txt files from the Multiwfn 3.7 program for the seven complexes. The wfn file inputs for the Multiwfn program were obtained from the single point energy calculations in gas phase from the ORCA 6.0[7–8] quantum chemistry package, at the domain-based local pair natural orbital CCSD(T) (DLPNO-CCSD(T)[13]) level of theory, with the x2c Hamiltonian[12] for scalar relativistic effects and the x2c-TZVPall[11] basis set.

Note: The single point energy calculations for generating electrophilicity data from Multiwfn 3.7 were initially performed with PBE0-D4/x2c-TZVPall level of theory with the x2c hamiltonian which failed to reproduce the expected electrophilicity trend. The same calculations were repeated with the more reliable coupled cluster theory, at the DLPNO-CCSD(T)/x2c-TZVPall level of theory with T = T1 and TightPNO settings, which resulted in the expected electrophilicity trend.

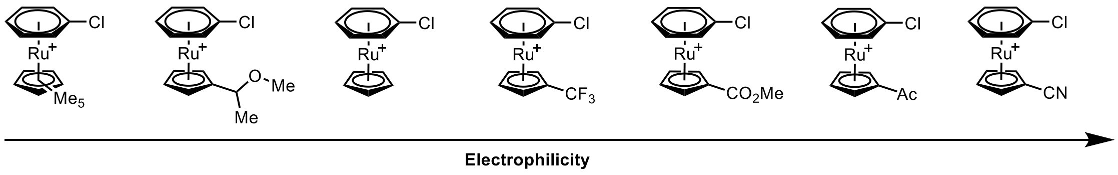

Global electrophilicity index [Hartree]:	0.0965	0.1104	0.1131	0.1197	0.1327	0.1343	0.1413
$\bar{\Phi}$ [0.5*((Ø of PhCl)+Ru)]:	0.081	0.127	0.134	0.139	0.274	0.274	0.300

Fig S167. Trends of global electrophilicity index and average electrophilicity of chlorobenzene carbons and the Ru atom together across the set of species with data from the electrophilicity table.

Table S1. The average electrophilicity of the six C atoms of the chlororobenzene ($\Phi_{Chlorobenzene}$), electrophilicity of the Ru atom (Φ_{Ru}) are given in column 2 and 3 respectively. Column 4 represents an average of column 2 and 3, i.e. $\bar{\Phi}_{(Chlorobenzene + Ru)} = 0.5\times(\Phi_{Chlorobenzene} + \Phi_{Ru})$. Global electrophilicity indices (in hartree) are listed in column 5.

Species	ΦPhCl	ΦRu	$\bar{\Phi}$(PhCl + Ru)	Global Electrophilicity index (hartree)
**[η^6(PhCl)RuCpMe$_5$]$^+$**	0.177	−0.016	0.081	0.096534
**[η^6(PhCl)RuCpCH(Me)(OMe)]$^+$**	0.223	0.030	0.127	0.110429
**[η^6(PhCl)RuCp]$^+$**	0.230	0.037	0.134	0.113054
**[η^6(PhCl)RuCpCF$_3$]$^+$**	0.243	0.034	0.139	0.119702
**[η^6(PhCl)RuCpCN]$^+$**	0.145	0.454	0.300	0.141348
**[η^6(PhCl)RuCpAc]$^+$**	0.136	0.411	0.274	0.134315
**[η^6(PhCl)RuCpCO$_2$Me]$^+$**	0.136	0.412	0.274	0.132732

Optimized structures with x, y, z coordinates (Å)

[η⁶(PhCl)RuCpMe₅]⁺

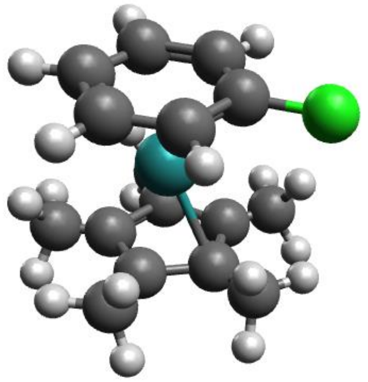

Fig S168. Optimized structure of the **[η^6(PhCl)RuCpMe$_5$]$^+$** cation obtained at the PBE0-D4/ x2c-TZVPall level of theory in the gas phase.

Coordinates

1 1

C	4.86895	0.94938	0.14101
H	2.78209	1.54279	-0.05904
C	3.51506	1.01842	0.55651
C	5.83451	0.32686	0.97255
C	3.12674	0.46665	1.80312
C	5.44531	-0.22610	2.21911
C	4.09149	-0.15581	2.63410
Ru	4.79090	1.87440	2.13782
Cl	1.57872	0.61234	2.28491
H	6.88492	0.31787	0.67616
H	3.80200	-0.53250	3.61674
H	5.17709	1.41948	-0.79454
H	6.19648	-0.65886	2.88214
C	5.44182	4.44121	0.88502
C	4.29203	3.19505	3.79044
C	4.05013	3.87507	2.55805
C	3.54767	2.96520	4.55218
C	3.08899	4.25663	2.21521
C	5.68216	2.87245	3.85094
C	5.29043	3.97203	1.85652

C	6.29923	3.35306	2.65609
C	6.18431	2.35498	4.66777
C	7.35529	3.26734	2.40257
H	4.50894	4.81957	0.52242
H	5.82565	3.71836	0.19577
H	6.13956	5.24651	0.98270
H	3.20967	4.71858	1.25764
H	2.72121	4.97752	2.91518
H	2.39212	3.44854	2.13610
H	2.59112	3.32611	4.23651
H	3.82129	3.43780	5.47233
H	3.49961	1.90597	4.69576
H	7.53031	3.71623	1.44718
H	7.63082	2.23404	2.36701
H	7.94195	3.76757	3.14453
H	7.22443	2.25191	4.43880
H	5.74973	1.38574	4.79666
H	6.07191	2.91911	5.57001

[η^6(PhCl)RuCpCH(Me)(OMe)]$^+$

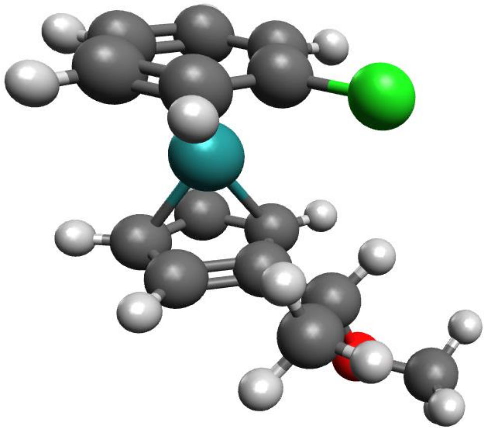

Fig S169. Optimized structure of the **[η^6(PhCl)RuCpCH(Me)(OMe)]$^+$** cation obtained at the PBE0-D4/x2c-TZVPall level of theory in the gas phase.

Coordinates

1 1

C	4.78581739295239	0.92822681371342	0.02706500731463

H	3.04014722083072	2.15237893737888	-0.31007335751178
C	3.48121286344217	1.38247565472126	0.30902306666237
C	5.38454246285909	-0.03012016493641	0.87302208410813
C	2.78740696305160	0.89223287460518	1.43382333695270
C	4.70551853354169	-0.51104459660271	2.01313895968214
C	3.39986154684412	-0.05001467009403	2.28556186419284
Ru	4.73429882585179	1.68952777184933	2.09984382988881
H	2.89476870774686	-0.38129106923263	3.18315156341923
H	5.34239972202343	1.33304227948659	-0.80772690688448
H	5.19951273207119	-1.20047993910470	2.68458303699462
H	5.61483555972857	4.32642694409443	1.25210297613943
C	4.63947583887482	2.71512079823304	4.01479395936388
C	4.36861459259508	3.64556351288326	2.97234646945423
H	3.95811989224659	2.42577669100836	4.80087205622072
H	3.44603478805598	4.18799118933913	2.82895158482249
C	5.95306187817768	2.21091482404416	3.81824758104039
C	5.51749133780929	3.71670073568519	2.13754896583187
C	6.50624807575824	2.82851266511308	2.65679524841537
H	6.45074212642886	1.47448478966982	4.43224708997026
C	7.90418961364556	2.61089857402898	2.16249969477459
C	8.12751788368922	3.10969712211592	0.75002231358787
O	8.73953269389460	3.27172772713743	3.10291310182086
C	10.07338189124612	2.81190178690668	3.08909219957599
H	10.59165783649141	3.31275754442070	3.90672255530351
H	10.58366257012054	3.05127239827583	2.14996332242445
H	10.11551542003189	1.72718751006154	3.24721121939878
H	8.11548803159993	1.53323580213766	2.20869612474516
H	7.45085264937813	2.61198267046833	0.05354146311439
H	9.14787967206797	2.89987371309219	0.42807099033658
H	7.96042603271889	4.18724161697098	0.69208994869717
H	1.81253797926588	1.29031051672216	1.68186734735052
Cl	6.96625466495933	-0.60105302419321	0.52415330279217

[η6(PhCl)RuCp]+

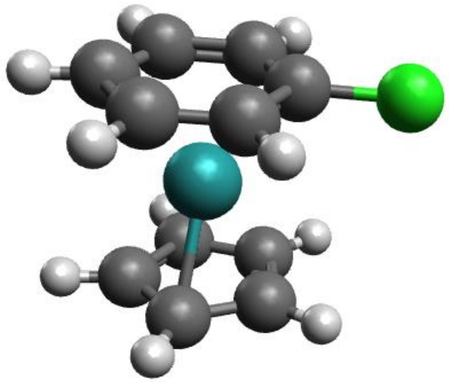

Fig S170. Optimized structure of the **[η6(PhCl)RuCp]+** cation obtained at the PBE0-D4/x2c-TZVPall level of theory in the gas phase.

Coordinates

1 1

C	4.877140000000	0.949639000000	0.140211000000
H	2.783060000000	1.553573000000	-0.054314000000
C	3.523871000000	1.032372000000	0.554076000000
C	5.840101000000	0.319662000000	0.968040000000
C	3.135382000000	0.472958000000	1.800013000000
C	5.448901000000	-0.227242000000	2.215818000000
C	4.098001000000	-0.149437000000	2.637787000000
Ru	4.805456000000	1.875717000000	2.134742000000
Cl	1.506138000000	0.598228000000	2.319855000000
H	6.889878000000	0.304967000000	0.670571000000
H	3.794230000000	-0.526734000000	3.615588000000
H	5.182615000000	1.417027000000	-0.797707000000
H	6.195504000000	-0.666476000000	2.879925000000
H	5.444147000000	4.446247000000	0.886243000000
C	4.292333000000	3.191104000000	3.786469000000
C	4.050620000000	3.870463000000	2.555166000000
H	3.543965000000	2.949014000000	4.540657000000
H	3.086135000000	4.238816000000	2.206753000000
C	5.684855000000	2.876279000000	3.851466000000
C	5.293170000000	3.975643000000	1.857213000000
C	6.303506000000	3.362938000000	2.659659000000

H	6.186654000000	2.359504000000	4.668944000000
H	7.360662000000	3.283677000000	2.408202000000

[η[6](PhCl)RuCpCF$_3$]$^+$

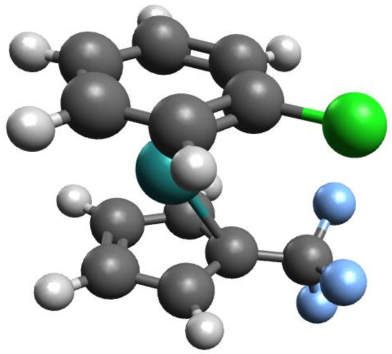

Fig S171. Optimized structure of the **[η[6](PhCl)RuCpCF$_3$]$^+$** cation obtained at the PBE0-D4/x2c-TZVPall level of theory in the gas phase.

Coordinates

1 1

C	4.41022608056510	1.09572640779503	-0.07244108787613
H	2.37306744934617	1.77115534000550	0.16709062000163
C	3.16678508421680	1.16987614875597	0.59011912316213
C	5.46325784284444	0.36190565965255	0.51268836732342
C	2.98383414644424	0.52458758768297	1.83063219438417
C	5.29737548254979	-0.26062152435707	1.76933870338266
C	4.04918868014129	-0.18581093451479	2.41990292382549
Ru	4.72786807340901	1.86437917467180	1.97478407928682
H	3.93421626649542	-0.62338084527158	3.40271267175006
H	4.57249635108716	1.62232691704549	-1.00331392287284
H	6.13465083344243	-0.76096803418575	2.23741379354511
H	5.83492532829361	4.31844074062889	0.85146519495497
C	4.27004238400770	3.23381574748168	3.60691917875273
C	4.24172493244342	3.95294612060800	2.37902193625925
H	3.43529852639216	3.08754127146886	4.27578003355615
H	3.38197533209443	4.44789595726130	1.95312561407571
C	5.57673679947731	2.70869123792656	3.78661021758898
C	5.53124630126918	3.88056387714963	1.79031685997962

C	6.35349820528187	3.10503949943810	2.65810478041143
H	5.92116885107048	2.11083674882120	4.61705284384527
C	7.81287119431605	2.85575006513174	2.48124471212524
F	8.18120819471874	2.95466566612588	1.20235479923250
F	8.17393141204820	1.64671955460359	2.92193334697964
F	8.53244969241086	3.75446899905381	3.17055636950595
Cl	6.96692586193821	0.24126673104065	-0.30090466947903
H	2.04937169369581	0.63664488597995	2.36402531629904

[η^6(PhCl)RuCpCN]$^+$

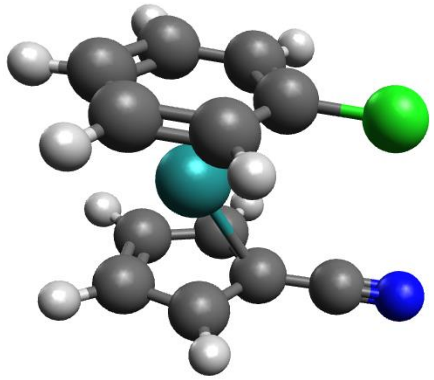

Fig S172. Optimized structure of the **[η^6(PhCl)RuCpCN]$^+$** cation obtained at the PBE0-D4/x2c-TZVPall level of theory in the gas phase.

Coordinates

1 1

C	4.68954701276834	0.98586041451183	-0.00865121284312
H	2.69464333343391	1.80319141912900	-0.13043667705894
C	3.36761887196808	1.17984292871475	0.44336462269399
C	5.58519781558901	0.22532068686656	0.77454780920636
C	2.95108709329879	0.63393844270683	1.67435425767002
C	5.18241625316002	-0.30241374851175	2.02108493685693
C	3.85808433170648	-0.10279123111105	2.46239841977141
Ru	4.75417965412493	1.86138386536845	2.01414365738781
H	3.56242871890802	-0.46380362261075	3.43862206048128
H	5.03130880290954	1.44276563174019	-0.92779288252549
H	5.89870255720286	-0.82313036110760	2.64247460300854
H	5.54885071310102	4.40172334355956	0.82761667839655

C	4.41387645433596	3.17071936343857	3.73112214604497
C	4.18638169909331	3.90724245679941	2.53488453667607
H	3.68125017409947	2.96255446484940	4.49629345074433
H	3.25130661472857	4.35537001418074	2.23431712197789
C	5.75952229552191	2.72643267485129	3.73694047130063
C	5.38820891425336	3.92863608315360	1.78438874549699
C	6.36960721800469	3.19923033032250	2.53026614262860
H	6.24757188596162	2.14034738553267	4.50074301060416
C	7.71889170859963	2.99749026375491	2.14872206994241
N	8.81714928780972	2.84257077449443	1.84277160991833
Cl	7.19052331935143	-0.01875508014400	0.23103524623563
H	1.95808627006907	0.84374649951031	2.04900717538457

[η⁶(PhCl)RuCpAc]⁺

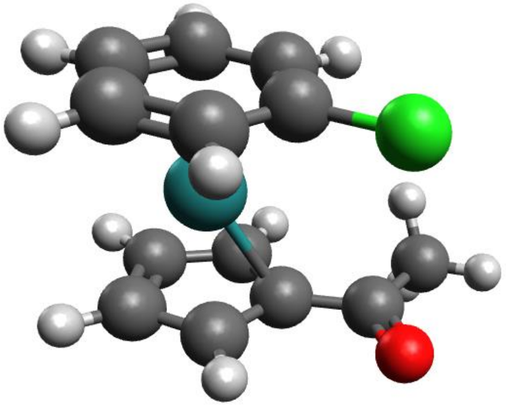

Fig S173. Optimized structure of the **[η⁶(PhCl)RuCpAc]⁺** cation obtained at the PBE0-D4/x2c-TZVPall level of theory in the gas phase.

Coordinates

1 1

C	4.58680224776911	1.03569402991067	0.00718864825631
H	2.52469705300497	1.66938160318137	0.10771503779924
C	3.30553233292105	1.10084941187212	0.59519534045562
C	5.62108618623377	0.34980253105251	0.67866252683102
C	3.06511625990832	0.49280762836427	1.84472785916291
C	5.39403444377770	-0.24048803447294	1.94213107310331
C	4.10835727112631	-0.17635303790873	2.51624958871438
Ru	4.77475086522018	1.87282451838812	2.03868066136349

H	3.94592137184127	-0.58587404622630	3.50443203264603
H	4.79109821480976	1.53757788674114	-0.92902300705505
H	5.70196668963031	4.35106606058621	0.83570119428792
C	4.30669766689677	3.24512541672210	3.66992344535743
C	4.19256436043981	3.94184959008752	2.43314019124203
H	3.50808810190735	3.07237115650713	4.37586087732225
H	3.29138536718663	4.38672155759747	2.03835609288404
C	5.64425244143974	2.79033934610514	3.80084755630782
C	5.45844164668324	3.92211004897456	1.79565367912454
C	6.36688759686709	3.20984380906172	2.63836152829962
H	6.04067160238370	2.22020695951475	4.62759630073038
C	7.78849105928276	2.94076733822742	2.30595154189573
O	8.26608453005874	3.41434750308479	1.29665178618971
C	8.56788637045440	2.09195611328485	3.25442063685871
H	8.06751908210948	1.13156659813116	3.40372325071217
H	8.61741944106239	2.58306045060869	4.23074329642088
H	9.57322055524879	1.93306585417488	2.87021071864945
Cl	7.18162513354570	0.26504048739762	-0.02699086736477
H	6.21419430885224	-0.70305728771646	2.47514386019524
H	2.09949441203827	0.60083922204814	2.32028635920953

[η^6(PhCl)RuCpCO$_2$Me]$^+$

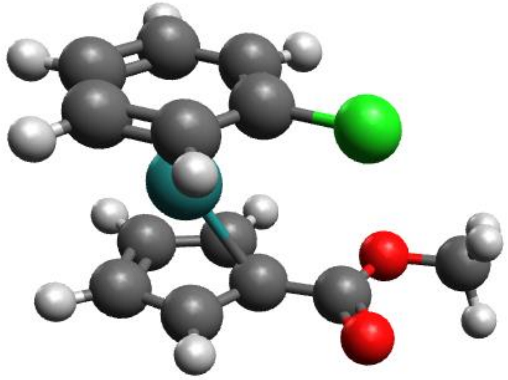

Fig S174. Optimized structure of the **[η^6(PhCl)RuCpCO$_2$Me]$^+$** cation obtained at the PBE0-D4/x2c-TZVPall level of theory in the gas phase.

Coordinates

1 1

C	4.75714791268616	1.01899292523831	0.01093647144409
H	2.70573239235937	1.68763846844825	-0.08272479673999
C	3.43038212536888	1.11552731628090	0.48136203146785
C	5.71588043105722	0.32770632837557	0.78281032393993
C	3.07062411309700	0.53623076061750	1.71533547959664
C	5.36889772105161	-0.23448129584645	2.03119220674236
C	4.03880253805511	-0.13527312614479	2.48864171865554
Ru	4.78523214922162	1.89023034500026	2.03484781390715
H	3.78246247368684	-0.52173731150897	3.46623016873433
H	5.05264989911252	1.49994108417883	-0.91188711687767
H	6.12975192406864	-0.70227619945515	2.64163207329152
H	5.60613401283523	4.41508065671905	0.84517762233467
C	4.30834197554912	3.22705698282217	3.69421837473029
C	4.14421092174936	3.93367333484090	2.46873959339974
H	3.53135008074780	3.01990571367737	4.41479543200662
H	3.22124497868670	4.35405412347993	2.09848724114085
C	5.66238132236212	2.81468610147751	3.79204979393165
C	5.39606486530779	3.96463974423580	1.80323394856509
C	6.33983353549451	3.27171626986137	2.61948007276771
H	6.10418249402479	2.24970738797266	4.59824368355455
C	7.75738199943891	3.06598872140335	2.25874255733682
O	8.26648320082297	3.53759687241411	1.27193726255885
O	8.38333962506591	2.30834539175493	3.14571435413604
C	9.76557158613780	2.04300573158792	2.88068256061307
H	10.10329505728448	1.41223642119078	3.69833801578805
H	10.32932775983949	2.97564360275645	2.85867866801118
H	9.87262025743280	1.52460596345417	1.92794579457250
H	2.06923908866694	0.66969593054920	2.10226826928767
Cl	7.33103255878795	0.20516975461798	0.21846038110277

Note: As shown above, our computational data indicates that the cyano-substituted Cp (CpCN) complex exhibits higher electrophilicity than the acetyl-substituted Cp (CpAc) complex. Therefore, his increased electrophilicity could potentially enhance the efficiency of the S_NAr reaction. Then why didn't we use this complex for performing on-DNA reactions? We would like to clarify our rationale for not employing the CpCN complex in the on-DNA reaction by outlining the following key considerations:

1. **Design constraints and stability:** Our design criteria required that the metal complex should not only possess sufficient electrophilicity to facilitate the S_NAr reaction but also be easy to synthesize, stable in air and aqueous media, and free from undesired side reactivity. The cyano group, while increasing electrophilicity, is also known to be reactive under nucleophilic conditions. This could lead to side reactions, such as nucleophilic attack by the amine on the cyano group. Such pathways would compromise both the selectivity and efficiency of the desired transformation.

2. **Potential coordination issues:** Another concern with the CpCN complex is the possibility of the cyano group coordinating to the metal center itself, which could interfere with or prevent proper arene coordination—an essential feature for the S_NAr reaction to proceed efficiently.

3. **Synthetic accessibility:** To the best of our knowledge, there are no reported synthetic procedures for the CpCN ligand. Considering the potential stability and reactivity issues discussed above, investing significant effort into the synthesis of a ligand that might inherently pose problems was not aligned with our design priorities.

4. **Computational benchmarking:** The computational studies involving the CpCN complex were primarily carried out to validate the expected electrophilicity trend—namely, that CpAc is more electrophilic than the parent Cp complex. Given that the cyano group is more electron-withdrawing than the acetyl group, we anticipated a higher global electrophilicity index for the CpCN complex, which was indeed observed. However, this evaluation was intended to serve as a theoretical benchmark rather than a guide for direct experimental application.

EXPERIMENTAL STUDIES

Reaction of DNA-AOP-NH2 (2) with different complexes

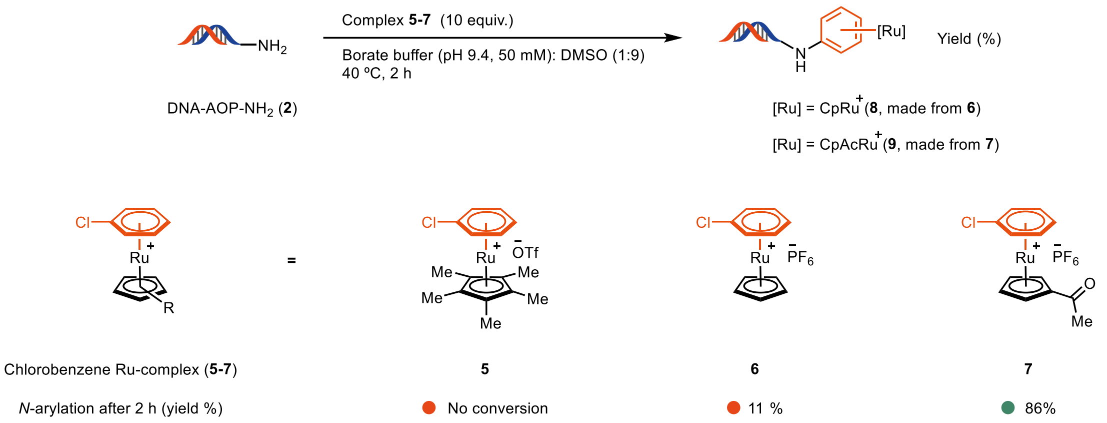

At 20–25 °C, the stock solution **SD-01** of DNA-AOP-NH2 (**2**) (1.0 µL, c = 2.0 mM, 2.0 nmol, 1.0 equiv.) in water was added to a 1.5 mL Eppendorf tube, followed by sodium borate buffer (1.0 µL, pH 9.4, c = 0.50 M). To this, 16 µL of DMSO was added, followed by the stock solution of the complexes (**5–7)** in DMSO (2.0 µL, c = 10 mM, 20 nmol, 10 equiv.). The resulting reaction mixture was vortexed for 5 seconds, transferred into a thermocycler, and incubated at 40 °C for 2 hours at 800 rpm to yield the *N*-arylated product. Next, the reaction mixture was diluted with 10 µL of Milli-Q water. To the above reaction mixture was added the stock solution of NaCl in water (**SR-06**, 3.0 µL, c = 5.0 M, 10% volume of the total reaction volume), followed by cold ethanol (−20 °C, 99 µL) to precipitate the DNA conjugate. The Eppendorf tube was placed in a freezer (−20 °C) for at least 1 hour, and then it was centrifuged at 4 °C and 10000 x g for at least 30 minutes. The supernatant was removed and the pellet was dried under air, then dissolved in 20 µL water to obtain the *N*-arylated product (20 µL, c = 0.10 mM). Then, 2.0 µL of the above solution was diluted to 40 µL with water for LC–MS analysis

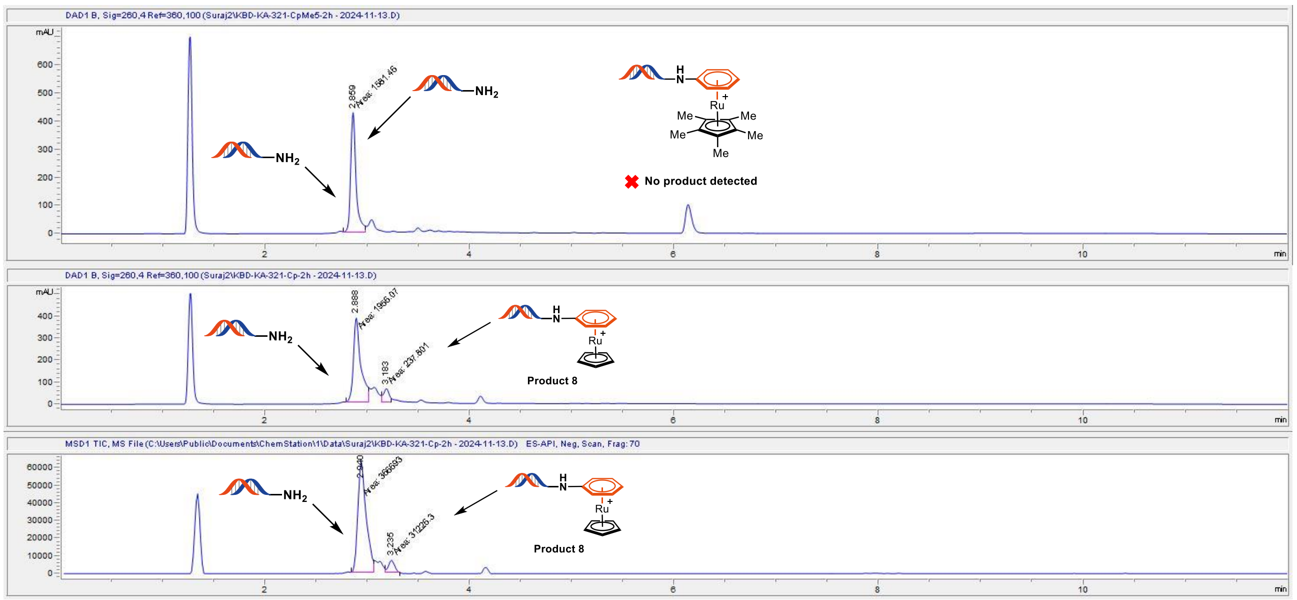

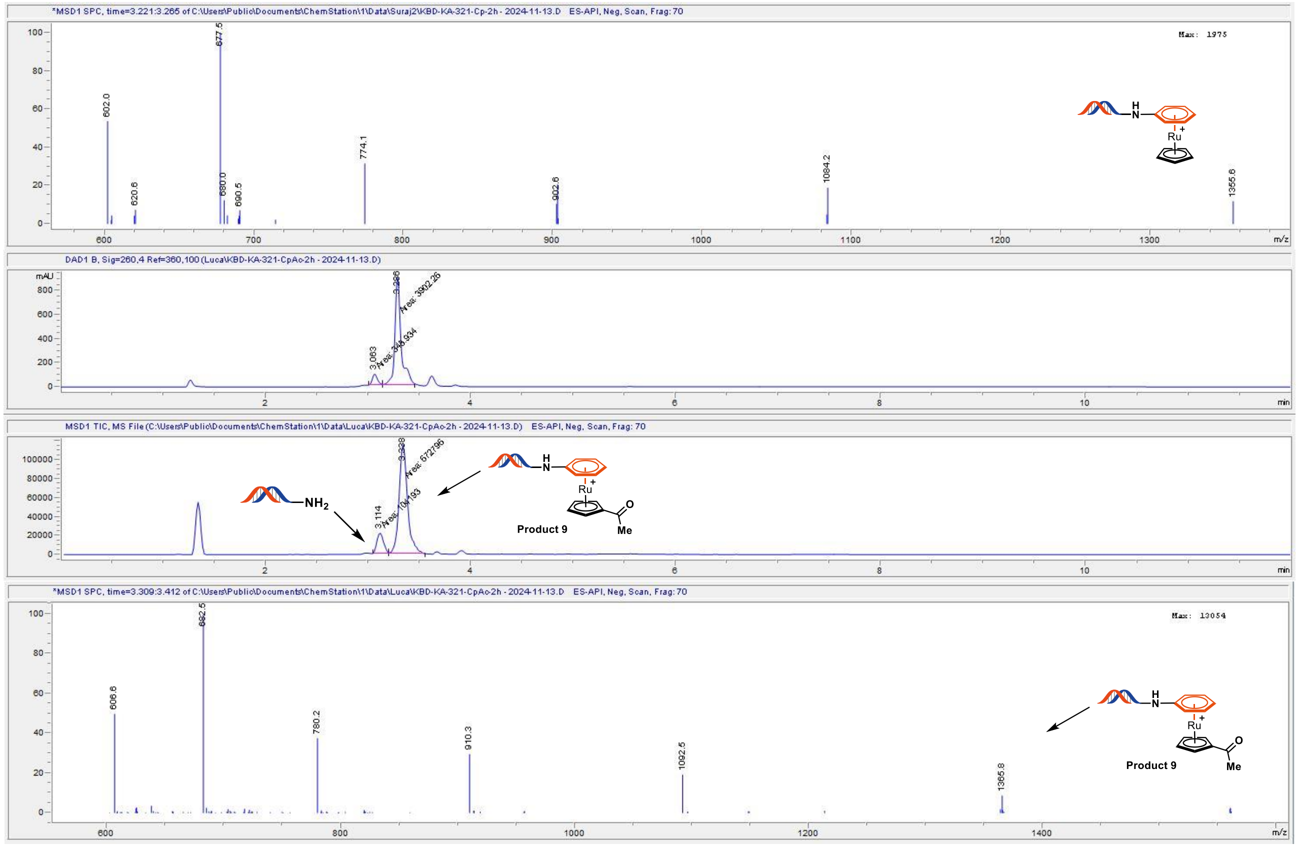

Figure S175. Analytical HPLC traces for the reaction of DNA-AOP-NH_2 (**2**) with various ruthenium complexes.

Result and discussion: The reaction of DNA-AOP-NH_2 (**2**) with the isolated ruthenium Cp* complex **5**, resulted in no product formation, whereas the reaction of **2** with ruthenium Cp complex **6** resulted in only 11% formation of the desired *N*-arylated product **8.** The reaction of DNA-AOP-NH_2 (**2**) with the ruthenium CpAc complex **7**, resulted in the formation of the *N*-arylated product in 86% yield, within 2 hours.

Chemoselectivity of DNA-AOP-NH_2 (2) towards C–N cross coupling reaction

Preparation of oxidative addition complex (OAC, 13) stock solution SA-18: Under an ambient atmosphere, a 1.5 mL Eppendorf tube was charged with the oxidative addition complex **13** (7.5 mg, 10 µmol). Next, 200 µL of DMSO was added to the Eppendorf tube and the vortexed for 10 seconds, resulting in the stock solution **SA-18** of the OAC (**13**) in DMSO (200 µL, c = 50 mM).

At 20–25 °C, the stock solution **SD-01** of DNA-AOP-NH_2 (**2**) (1.0 µL, 2.0 mM, 2.0 nmol, 1.0 equiv.) in water was added to a 1.5 mL Eppendorf tube, followed by sodium borate buffer (1.0 µL, pH 9.4, c = 0.50 M).To the Eppendorf tube 16 µL of DMSO was added, followed by the stock solution of the oxidative addition complex (OAC) (**SA-18**, 2.0 µL, 50 mM, 0.100 µmol, 50 equiv.) in DMSO. The resulting reaction mixture was vortexed for 5 seconds, transferred into a thermocycler, and incubated at 40 °C for 16 hours at 800 rpm. The palladium in the reaction mixture was then quenched with a stock solution of sodium diethyldithiocarbaminate trihydrate (DTC) stock solution (2.0 µL, 150 mM). The supernatant was transferred to another Eppendorf tube and then

added the stock solution of NaCl in water (**SR-06** , 2.0 µL, c = 5.0 M, 10% volume of the total reaction volume), followed by cold ethanol (−20 °C, 66 µL) to precipitate the *N*-arylated ruthenium DNA conjugate **16**. The Eppendorf tube was placed in a freezer (−20 °C) for at least 1 hour, and then it was centrifuged at 4 °C and 10000 x g for at least 30 minutes. The supernatant was removed and the pellet was dried under air, then dissolved in 20 µL water to obtain the DNA-conjugate **16** (20 µL, c = 0.10 mM). Next, 2.0 µL of the above solution was diluted to 40 µL with water for LC–MS analysis (yield was calculated by integrating the DNA ionization peaks in the TIC chromatogram).

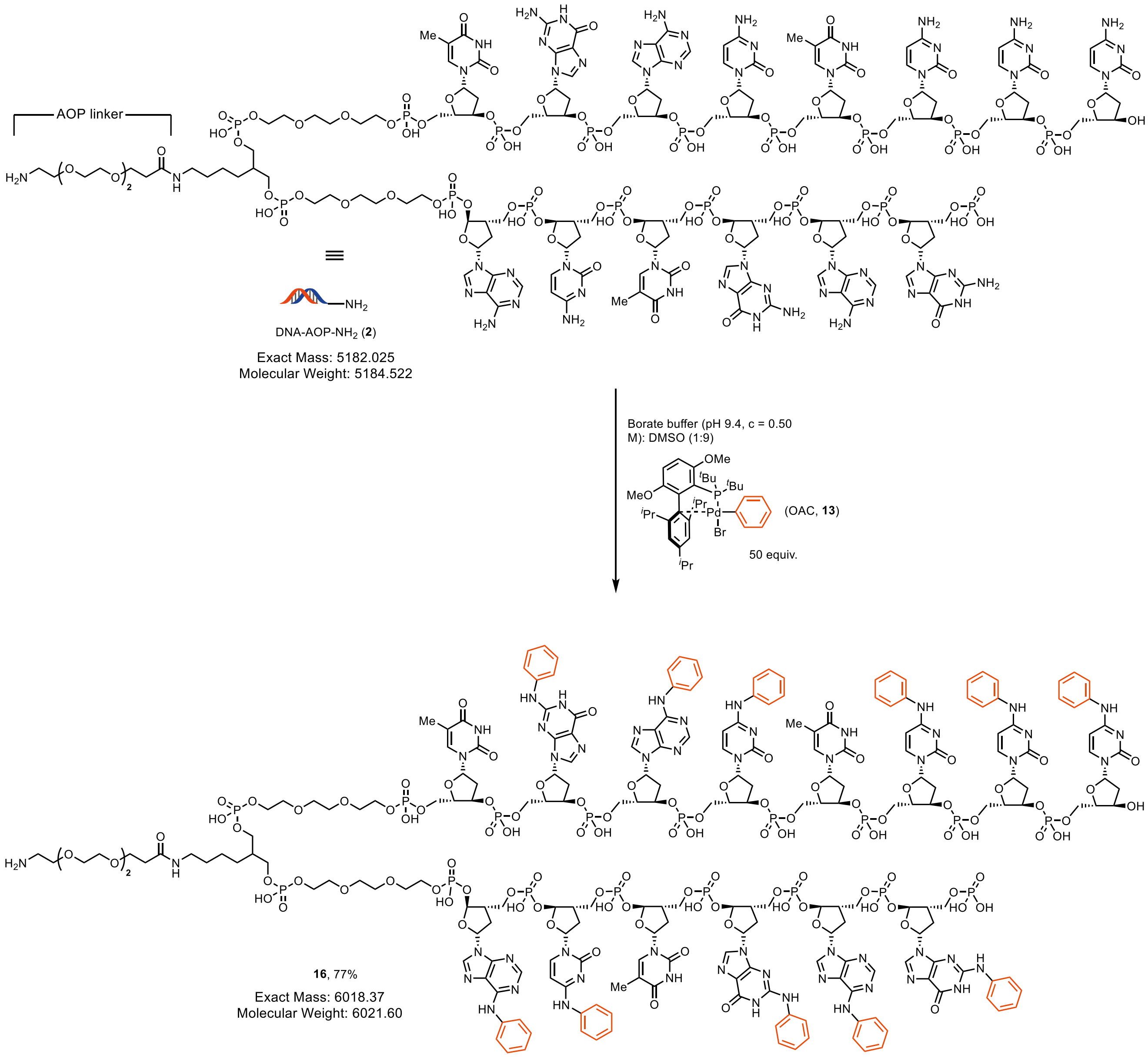

Scheme S1. Structure of polyarylated DNA-AOP-NH₂ (**2**) with five cytosine arylations, three adenine arylations, and three guanine arylations.

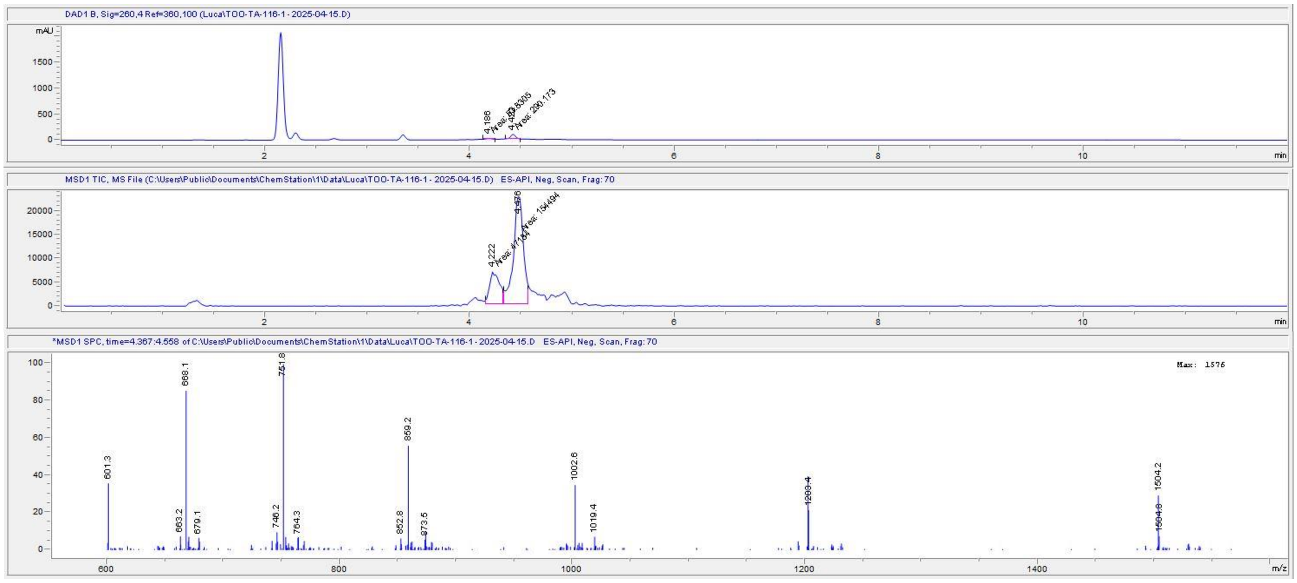

Figure S176. Analytical HPLC trace of **16** with HPLC method A. (Top) DAD chromatogram at 260 nm. (Middle) TIC chromatogram. (Bottom) Ionization of peak at 4.47 min. containing polyarylated DNA-conjugate **16**.

Control experiment

Synthesis of benzoic acid-DNA conjugate

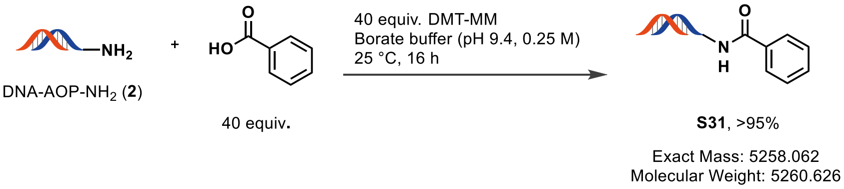

Preparation of benzoic acid stock solution (SA-19): Under an ambient atmosphere, a 1.5 mL Eppendorf tube was charged with benzoic acid (4.5 mg, 40 µmol). Next, 100 µL of DMA was added to the above 1.5 mL Eppendorf tube and the mixture was vortexed for 10 seconds. Next, the Eppendorf tube was sonicated for 10 seconds to ensure that benzoic acid is completely dissolved, resulting in the formation of benzoic acid stock solution **SA-04** in DMA (c = 0.40 M).

Under an ambient atmosphere, the stock solution **SD-01** of DNA-AOP-NH_2 (**2**) in water (10 µL, c = 2.0 mM, 20 nmol, 1.0 equiv.) was added to a 1.5 mL Eppendorf tube, followed by sodium borate buffer (10 µL, pH 9.4, c = 0.50 M). Next, the stock solution of benzoic acid in DMA (**SA-19**, 2.0 µL, c = 0.40 M, 0.80 µmol, 40 equiv.) was added. The reaction mixture was vortexed for 5 seconds. Then, the stock solution of DMT-MM in water (**SR-02**, 2.0 µL, c = 0.40 M, 0.80 µmol, 40 equiv.) was added. The mixture was vortexed for 5 seconds, transferred to a thermocycler at 25 °C, and incubated for 16 hours at 600 rpm. After 16 hours, an aliquot of 1.0 µL of the reaction mixture was diluted to 40 µL with water for LC–MS analysis. Next, to the above reaction mixture was added the stock solution of NaCl in water (**SR-06**, 2.4 µL, c = 5.0 M, 10% volume of the total reaction volume), followed by cold ethanol (−20 °C, 80 µL) to precipitate the *N*-Fmoc amine DNA conjugate

S31. The Eppendorf tube was placed in a freezer (−20 °C) for at least 1 hour, and then it was centrifuged at 4 °C and 11000 × g for at least 30 minutes. The supernatant was removed and the pellet was dried under air, then dissolved in 10 µL water (c = 2.0 mM).

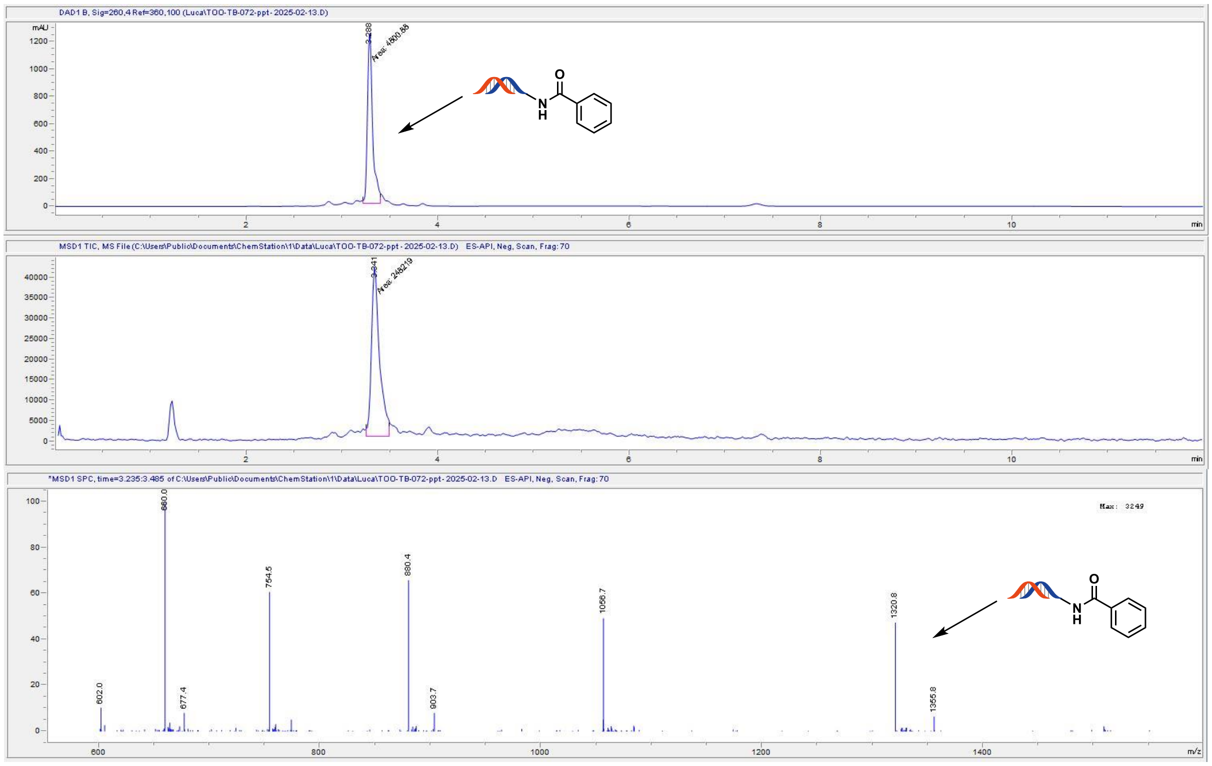

Figure S177. Analytical HPLC trace of **S31** with HPLC method A. (Top) DAD chromatogram at 260 nm. (Middle) TIC chromatogram. (Bottom) Ionization of peak at 3.34 min. containing reaction product.

Reaction of DNA conjugate S31 with Ru-fluorobenzene complex C1

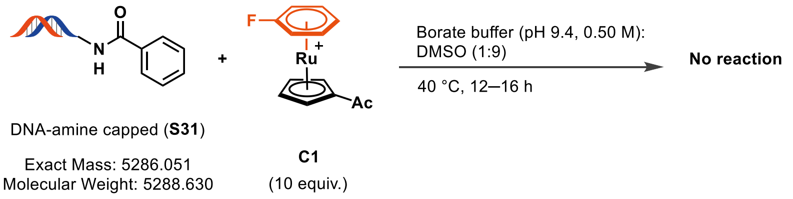

In situ formation of arene-ruthenium stock solution: Under an ambient atmosphere, a 1 mL glass GC vial equipped with a 6 mm Teflon-coated stirring bar was charged with ruthenium complex **1** (1.4 mg, 2.9 µmol, 1.0 equiv.). Next, a stock solution of fluorobenzene in DMC (294 µL, c = 0.10 M, 29 µmol, 10 equiv.) was added. The resulting reaction mixture was heated at 80 °C for 2 hours. After 2 hours, the reaction mixture was cooled to 23 °C. Next, the DMC was removed under a gentle stream of argon and 294 µL of DMSO were added to result an in situ formed stock solution of arene-ruthenium complex **C1** (294 µL, c = 0.01 M, assuming quantitative arene coordination to ruthenium).

Under an ambient atmosphere, the stock solution of DNA-conjugate **S31** in water (1.0 µL, c = 2.0 mM, 2.0

nmol, 1.0 equiv.) was added to a 1.5 mL Eppendorf tube, followed by sodium borate buffer (1.0 µL, pH 9.4, c = 0.50 M). To this mixture, 16 µL of DMSO was added and the solution was vortexed for 5 seconds. Next, the freshly prepared stock solution **C1** (2.0 µL, c = 0.01 M, 0.02 µmol, 10 equiv.) in DMSO was added. The resulting reaction mixture was vortexed for 5 seconds, transferred to a thermocycler at 40 °C, and incubated for 16 hours at 800 rpm to yield the DNA-conjugate. Next, the reaction mixture was diluted with 10 µL of Milli-Q water. To the reaction mixture was added the stock solution of NaCl in water (**SR-06** , 3.0 µL, c = 5.0 M, 10% volume of the total reaction volume), followed by cold ethanol (−20 °C, 99 µL) to precipitate the *N*-arylated ruthenium DNA conjugate. The Eppendorf tube was placed in a freezer (−20 °C) for at least 1 hour, and then it was centrifuged at 4 °C and 11000 × g for at least 30 minutes. The supernatant was removed and the pellet was dried under air, then dissolved in 20 µL water to obtain the DNA-conjugate (20 µL, c = 0.10 mM). Then, 1.0 µL of the above solution was diluted to 40 µL with water for LC–MS analysis.

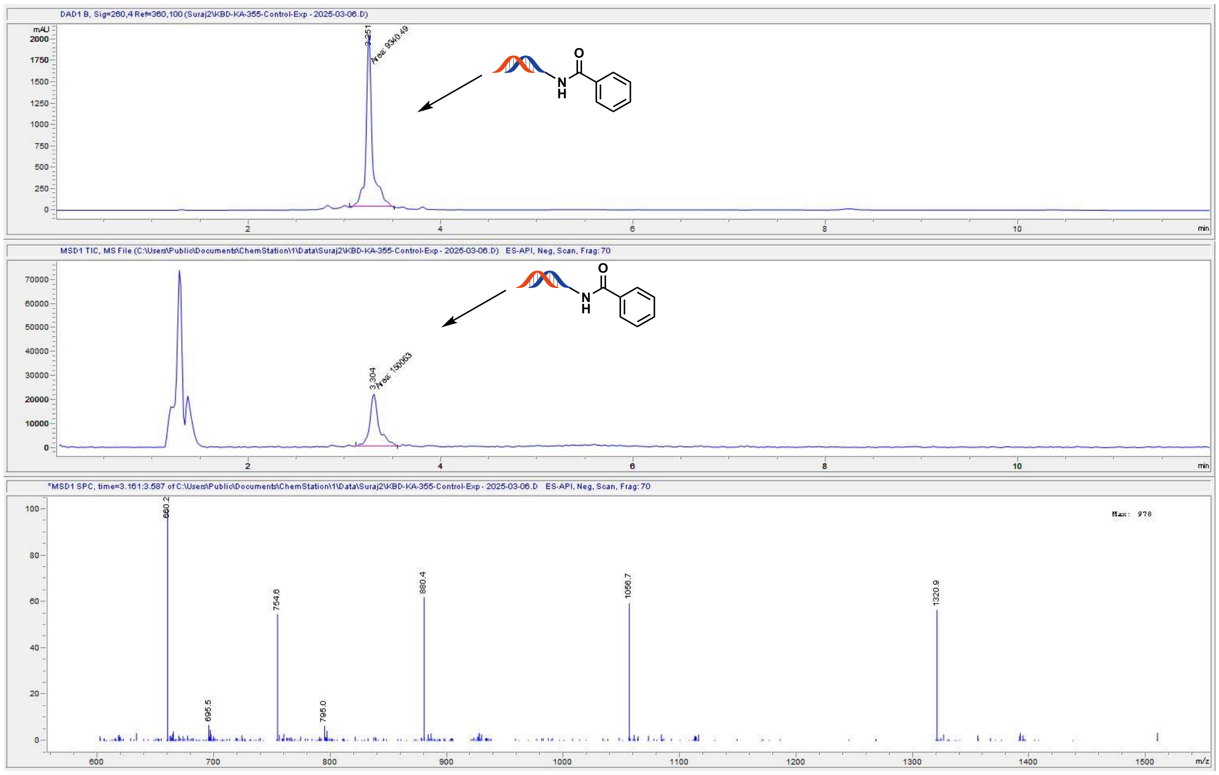

Figure S178. Analytical HPLC trace of unreacted DNA-conjugate **S31** with HPLC method A. (Top) DAD chromatogram at 260 nm. (Middle) TIC chromatogram. (Bottom) Ionization of peak at 3.30 min. containing the starting material.

Co-injection experiment

Synthesis of DNA conjugate 73 via amide coupling

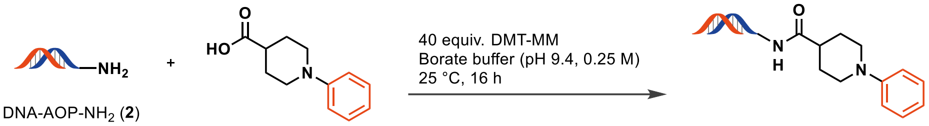

DNA-AOP-NH_2 (**2**)

40 equiv.

(stock solution, **SA-20**)

73, >95%

Exact Mass: 5384.148
Molecular Weight: 5386.799

Preparation of 1-phenylpiperidine-4-carboxylic acid stock solution (SA-20): At 20–25 °C, a 1.5 mL Eppendorf tube was charged with 1-phenylpiperidine-4-carboxylic acid (8.2 mg, 0.040 mmol). Next, 100 µL of DMA was added to the above 1.5 mL Eppendorf tube and the mixture was vortexed for 10 seconds. Next, the Eppendorf vial was sonicated for 10 seconds to make sure that the acid is completely dissolved, which resulted in the formation of the 1-phenylpiperidine-4-carboxylic acid stock solution (**SA-20**) (c = 0.40 M) in DMA.

At 20–25 °C, the stock solution **SD-01** of DNA-AOP-NH_2 (**2**) (10 µL, c = 2.0 mM, 20 nmol, 1.0 equiv.) in water was added to a 1.5 mL Eppendorf tube, followed by sodium borate buffer (10 µL, pH 9.4, c = 0.50 M). Next, the stock solution of 1-phenylpiperidine-4-carboxylic acid stock solution (**SA-20**, 2.0 µL, c = 0.40 M, 0.80 µmol, 40 equiv.) in DMA was added. The reaction mixture was vortexed for 5 seconds. Then, the stock solution of DMT-MM (**SR-02**, 2.0 µL, c = 0.40 M, 0.80 µmol, 40 equiv.) in water was added. The mixture was vortexed for 5 seconds, transferred to a thermocycler at 25 °C, and incubated for 16 hours at 600 rpm. After 16 hours, an aliquot of 1.0 µL of the reaction mixture was diluted to 40 µL with water for LC–MS analysis. Next, to the above reaction mixture was added the stock solution of NaCl in water (**SR-06**, 2.4 µL, c = 5.0 M, 10% volume of the total reaction volume), followed by cold ethanol (−20 °C, 80 µL) to precipitate the DNA conjugate. The Eppendorf tube was placed in a freezer (−20 °C) for at least 1 hour, and then it was centrifuged at 4 °C and 11000 × g for at least 30 minutes. The supernatant was removed and the pellet was dried under air, then dissolved in 20 µL water (c = 1.0 mM).

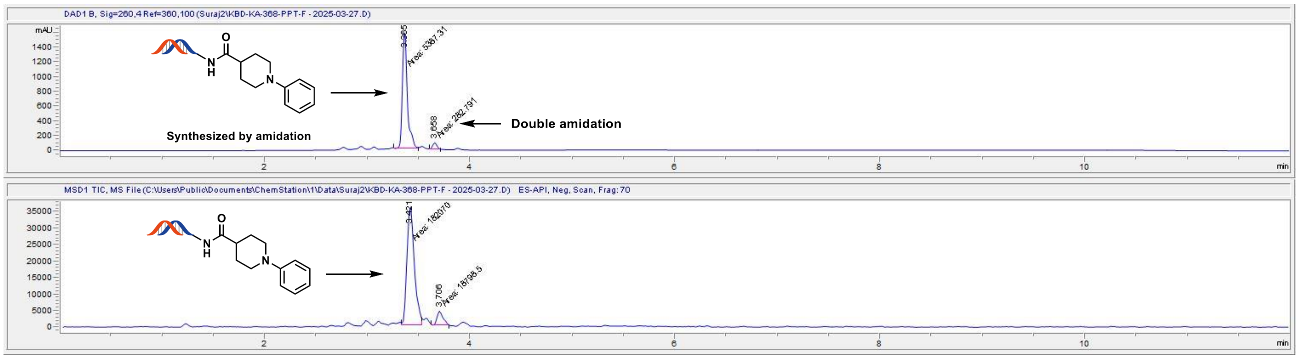

DMT-MM adduct

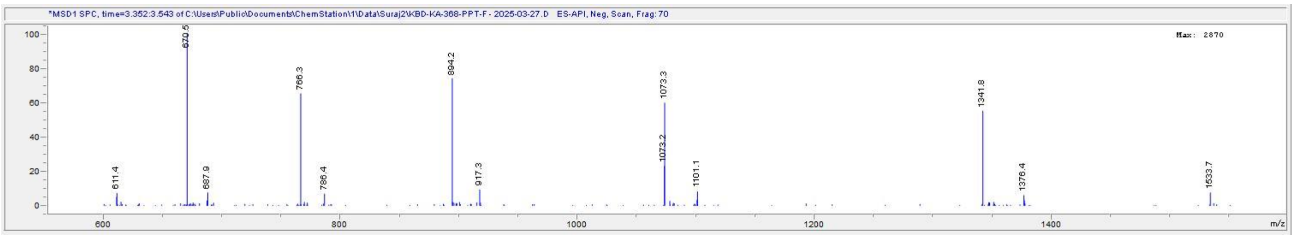

Figure S179. Analytical HPLC trace of **73** with HPLC method A. (Top) DAD chromatogram at 260 nm. (Middle) TIC chromatogram. (Bottom) Ionization of peak at 3.42 min. containing reaction product.

Synthesis of DNA conjugate 73 via ruthenium-mediated S_NAr

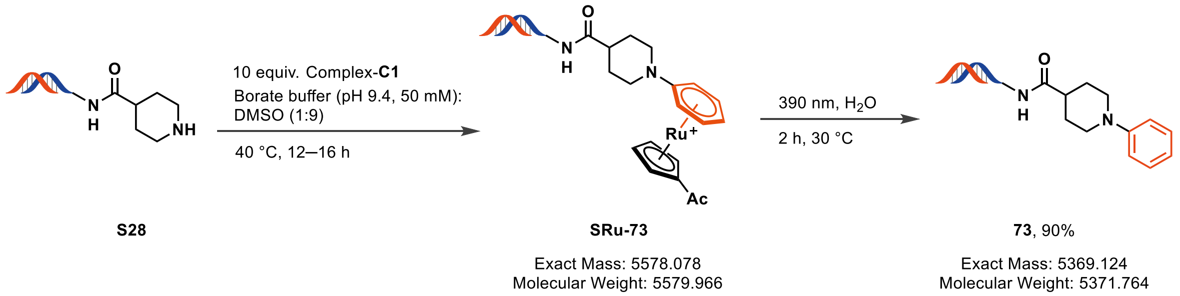

In situ formation of arene-ruthenium stock solution: Under an ambient atmosphere, a 1 mL glass GC vial equipped with a 6 mm Teflon-coated stirring bar was charged with ruthenium complex **1** (1.4 mg, 2.9 µmol, 1.0 equiv.). Next, a stock solution of fluorobenzene in DMC (294 µL, c = 0.10 M, 29 µmol, 10 equiv.) was added. The resulting reaction mixture was heated at 80 °C for 2 hours. After 2 hours, the reaction mixture was cooled to 23 °C. Next, the DMC was removed under a gentle stream of argon and 294 µL of DMSO were added to result an in situ formed stock solution of arene-ruthenium complex **C1** (294 µL, c = 0.01 M, assuming quantitative arene coordination to ruthenium).

At 20–25 °C, the stock solution of DNA-conjugate **S28** (1.0 µL, c = 2.0 mM, 2.0 nmol, 1.0 equiv.) in water was added to a 1.5 mL Eppendorf tube, followed by sodium borate buffer (1.0 µL, pH 9.4, c = 0.50 M). To this mixture, 16 µL of DMSO was added and the solution was vortexed for 5 seconds. Next, the freshly prepared stock solution **C1** (2.0 µL, c = 0.01 M, 0.02 µmol, 10 equiv.) in DMSO was added. The resulting reaction mixture was vortexed for 5 seconds, transferred to a thermocycler at 40 °C, and incubated for 16 hours at 800 rpm to yield the DNA-conjugate **SRu-73**. Next, the reaction mixture was diluted with 10 µL of Milli-Q water. To the reaction mixture was added the stock solution of NaCl in water (**SR-06** , 3.0 µL, c = 5.0 M, 10% volume of the total reaction volume), followed by cold ethanol (−20 °C, 99 µL) to precipitate the *N*-arylated ruthenium DNA conjugate **SRu-73**. The Eppendorf tube was placed in a freezer (−20 °C) for at least 1 hour, and then it was centrifuged at 4 °C and 11000 × g for at least 30 minutes. The supernatant was removed and the pellet was dried under air, then dissolved in 20 µL water to obtain the DNA-conjugate **SRu-73** (20 µL, c = 0.10 mM). Then, 1.0 µL of the above solution was diluted to 40 µL with water for LC–MS analysis.

Decomplexation of SRu-73 to obtain product 73: Under an ambient atmosphere, the DNA-conjugate **SRu-73** stock solution in water (c = 0.10 mM, 20 µL) was irradiated with a 390 nm (40 W) Kessil lamp for 2 hours,

while maintaining the temperature at approximately 30 °C through cooling with a fan. To the reaction mixture was added the stock solution of NaCl in water (**SR-06**, 2.0 µL, c = 5.0 M, 10% volume of the total reaction volume), followed by cold ethanol (−20 °C, 66 µL) to precipitate the DNA conjugate **73**. The Eppendorf tube was placed in the freezer (−20 °C) for at least 1 hour, and then it was centrifuged at 4 °C and 11000 × g for at least 30 minutes. The supernatant was removed, the pellet dried under air and dissolved in Milli-Q water to obtain the purified DNA-conjugate **73**. Then, 1 µL of the above solution was diluted to 40 µL with water for LC–MS analysis.

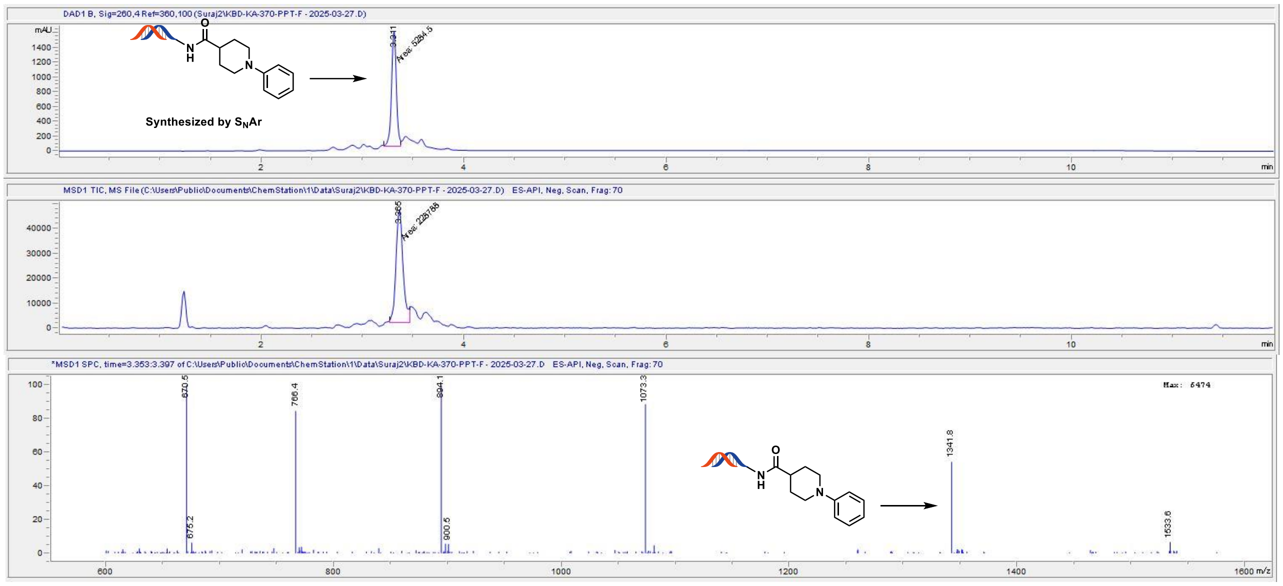

Figure S180. Analytical HPLC trace of **73** with HPLC method A. (Top) DAD chromatogram at 260 nm. (Middle) TIC chromatogram. (Bottom) Ionization of peak at 3.36 min. containing reaction product.

Co-injected samples of DNA conjugate 73 synthesized by two different methodS

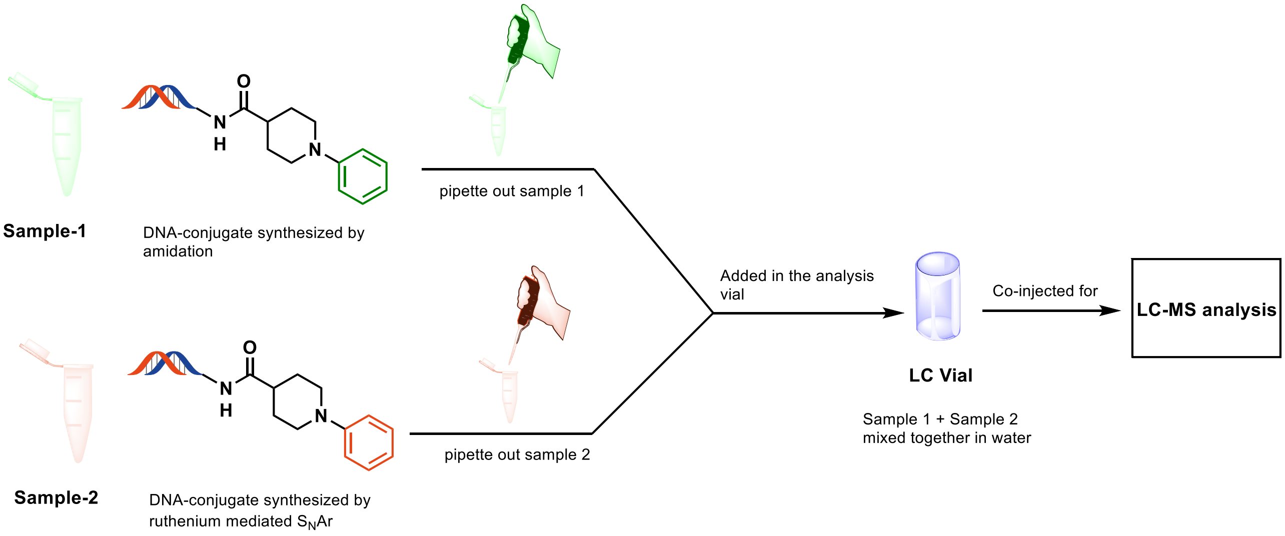

Scheme S2. Representation of co-injection experiment. Sample-1 and sample-2 mixed together and co-

injected for the LC-MS analysis.

To perform the co-injection experiment, 2.0 µL of the 0.1 mM stock solutions of the DNA-conjugate **73**, synthesized by the two different methods, were added to 40 µL of Milli-Q water. The solution was mixed and injected for the LC-MS analysis. The analysis confirmed the formation of the same product with the same chemoselectivity.

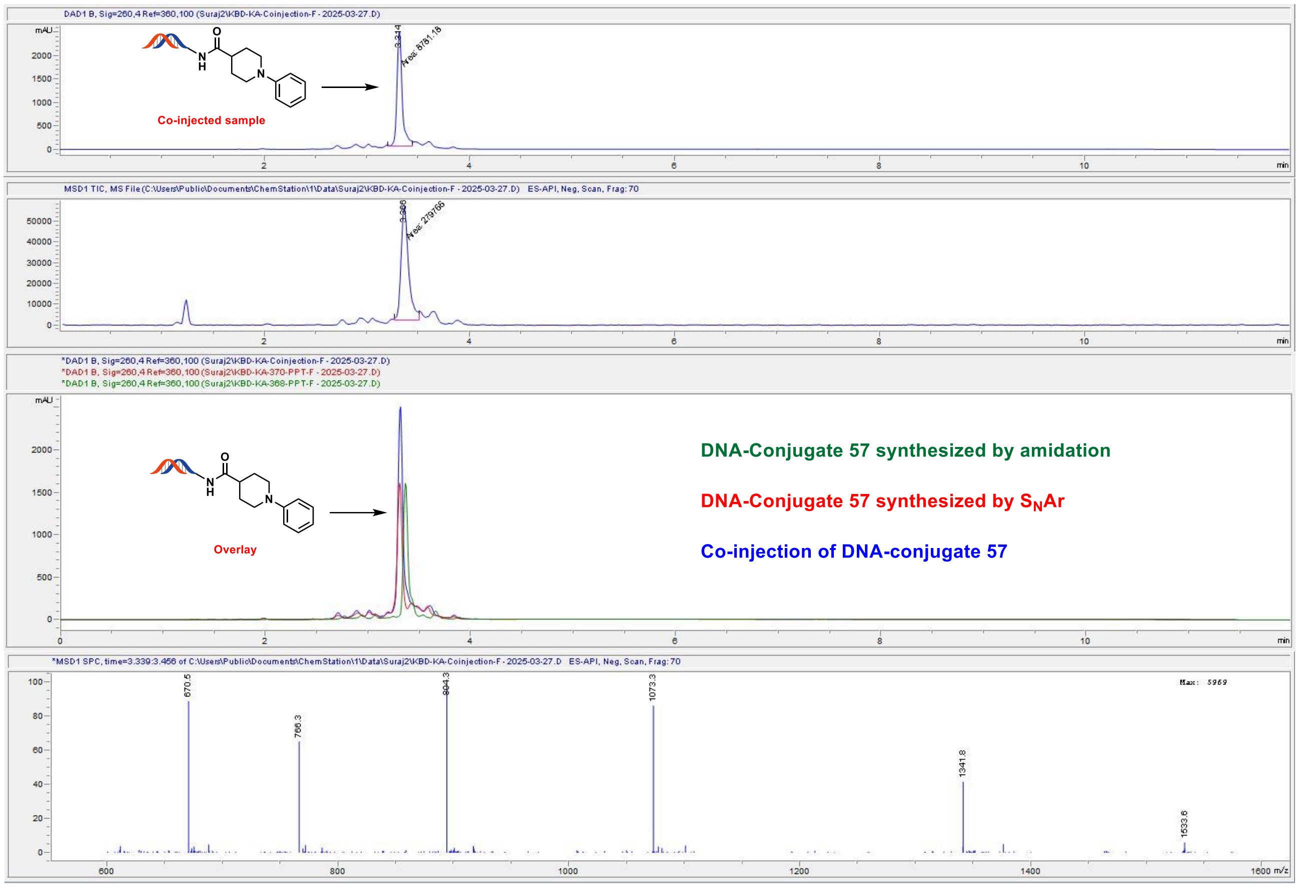

Figure S181. Analytical HPLC trace of **73** (after co-injection) with HPLC method A. (Top) DAD chromatogram at 260 nm. (Middle top) TIC chromatogram containing the reaction product at 3.36 min. (Middle bottom) co-injected sample. (Bottom) Ionization of peak at 3.36 min. containing reaction product.

N-arylation of ligated DNA-conjugate S33

Step-1: DNA-AOP-NH_2 (**2**) ligation with tags

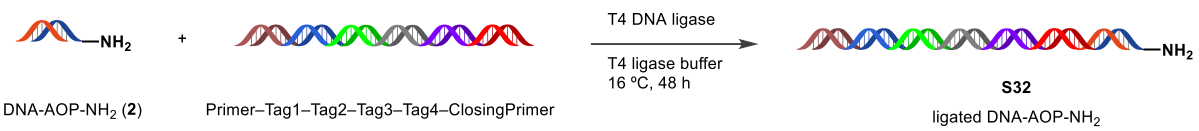

Step-2: *N*-arylation of ligated DNA-AOP-NH_2

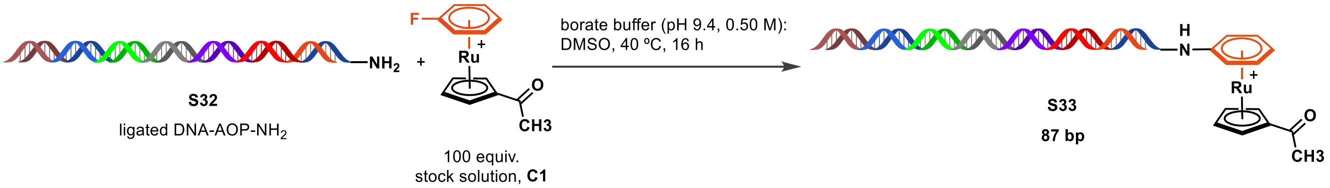

At 4 °C, 5.0 µL of DNA-AOP-NH_2 (**2**) (1.0 mM, 5.0 nmol, 1.0 equiv.) in water was added to a 1.5 mL Eppendorf tube. Next, 13 µL of a stock solution of the DNA fragment Primer–Tag1–Tag2–Tag3–Tag4–ClosingPrimer (DNA tags for ligation) (0.47 mM, 6.1 nmol, 1.2 equiv.) in water was added and cooled to 16 °C. In another Eppendorf tube at 4 °C, 2.0 µL of 10X T4 DNA ligase buffer and 0.20 µL of T4 DNA ligase were premixed. The mixture was added over the solution of DNA-AOP-NH_2 (**2**), vortexed for 5 seconds, transferred into a Thermocycler pre-cooled at 16 °C, and incubated at 16 °C for 48 hours without stirring. After 48 hours, the reaction mixture was centrifuged and diluted with 20 µL of water, and added the stock solution of NaCl in water (**SR-06**, 4.0 µL, c = 5.0 M, 10% volume of the total reaction volume), followed by cold ethanol (−20 °C, 66 µL) to precipitate the DNA conjugate **S32**. The Eppendorf tube was placed in the freezer (−20 °C) for at least 1 hour, and then it was centrifuged at 4 °C and 11000 × g for at least 30 minutes. The supernatant was removed, the pellet dried under air and dissolved in Milli-Q water to obtain the purified DNA-conjugate **S32** (50 µL, c = 0.10 mM).

Under an ambient atmosphere, a 1 mL glass GC vial equipped with a 6 mm Teflon coated magnetic stirring bar was charged with a stock solution of fluorobenzene (100 µL, c = 100 mM), followed by a stock solution of ruthenium complex **1** (100 µL, c = 10 mM). The vial was closed using a screw cap and the reaction mixture was heated at 80 °C for 1 hours. After 1 hour, the DMC was removed under a gentle stream of argon and then added 200 µL of DMSO to form the stock solution of fluorobenzene ruthenium complex **C1** (c = 5.0 mM).

In a 1.5 mL Eppendorf tube was added the stock solution of the ligated DNA-conjugate **S32** (2.0 µL, c = 0.10 mM, 0.20 nmol, 1.0 equiv.) and dried. In another 1.5 mL Eppendorf tube the stock solution of ruthenium complex **C1** (4.0 µL, c = 5.0 mM, 0.02 µmol, 100 equiv.) in DMSO was added, followed by sodium borate buffer (2.0 µL, pH 9.4, c = 0.50 M,). The solution was then vortexed for 5 seconds and then transferred to the first Eppendorf tube containing the dried ligated DNA-conjugate **S32**. The mixture was vortexed for 5 seconds, transferred to a Thermocycler at 40 °C and incubated for 2 hours at 600 rpm. After 16 hours, to the Eppendorf tube was added 200 µL of Milli-Q water, followed by a stock solution of NaCl in water (**SR-06**, 30 µL, c = 5.0 M) and cold ethanol (−20 °C, 800 µL), to precipitate the DNA-conjugate **S33**. The Eppendorf was

then placed in freezer at −20 °C for 1 hour and then was centrifuged at 11000 × g for 1 hour. The supernatant was decanted, and the pellet was dissolved in 300 µL of water and, the same procedure was repeated one more time. The remaining pellet was then dried under a flow of argon and dissolved again in 400 µL of Milli-Q water and filtered through an YM10K filter. The procedure for filtering was repeated three more times. The DNA-conjugate **S33** was then diluted to 100 ng/ µL of Milli-Q water and stored in the freezer at −20 °C. The ligation was analyzed by GEL electrophoresis with SYBR Green as dye, 4% agarose gel and 75 V and 150 V for 1 hour to 1.5 hours.

Note: The reaction was repeated three times and each of the products were analysed by GEL electrophoresis and qPCR separately.

GEL electrophoresis protocol

- 4% agarose gel (1.2 g) to 30 mL of buffer

8 lanes:

- Lane 1: DNA ladder (Bio-Rad Laboratories EZ Load 20 bp Molecular Ruler)
- Lane 2: DNA-AOP-NH_2 (**2**, 120 ng).
- Lane 3: Primer–Tag1–Tag2–Tag3–Tag4–ClosingPrimer (60 ng).
- Lane 4: Ruthenium-DNA conjugate **S33.** 1st repetition
- Lane 5: Ruthenium-DNA conjugate **S33.** 2nd repetition
- Lane 6: Ruthenium-DNA conjugate **S33.** 3rd repetition
- Lane 7: Ligated DNA-AOP-NH_2 (**S32**, 60 ng).
- Lane 8: DNA ladder (Bio-Rad Laboratories EZ Load 20 bp Molecular Ruler).

Gel images were processed using Bio-Rad Laboratories, Inc. Image Lab Version 6.1.0 build 7, Standard Edition.

After loading the samples, the gel was run at 75V or 150 V for 15 min. to 1.5 hours, as mentioned below.

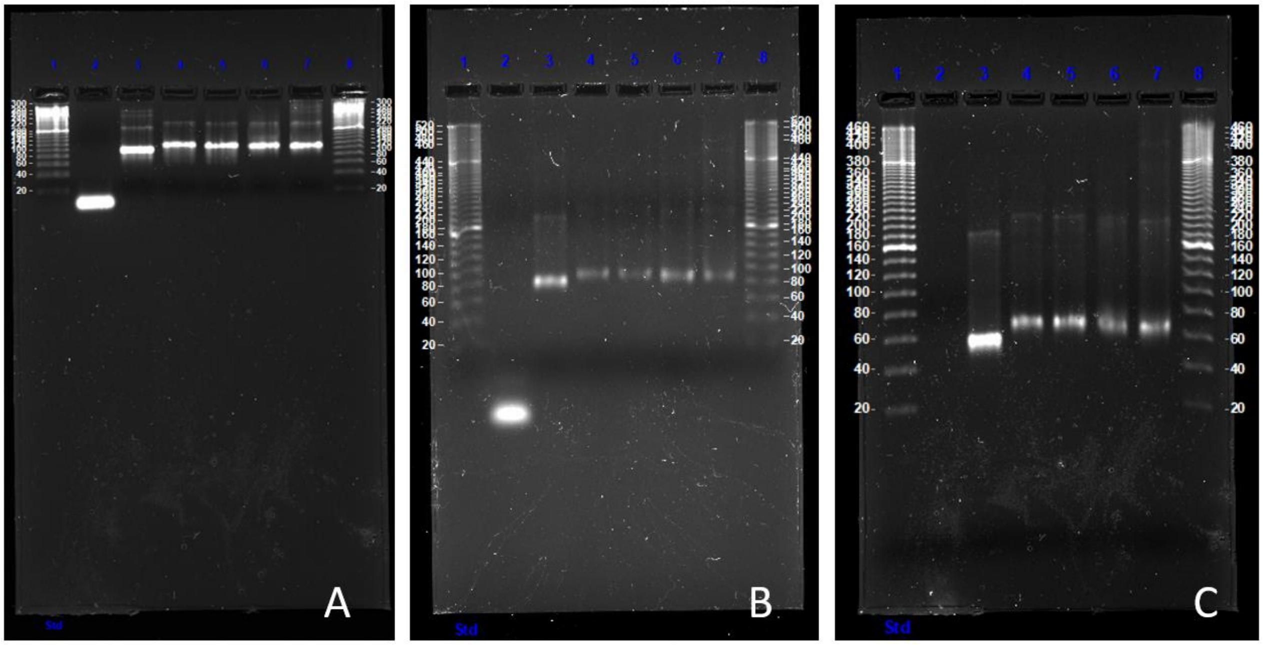

Figure S182. Images of the DNA gel after the voltage 75-150 V. A. Gel running at 75 V after 15 min. B. Gel running at 75 V for 1.5 hours. C. Gel running at 150 V for 1.5 hours to observe a better separation between the ligated samples and the tags. In the above image Lane 1 and 8 contains DNA ladder (Bio-Rad Laboratories EZ Load 20 bp Molecular Ruler). Lane 2 contains the DNA-AOP-NH_2 (**2**). Lane 3 contains only the tags (Primer–Tag1–Tag2–Tag3–Tag4–ClosingPrimer). Lane 4-6 contains Ruthenium-DNA conjugate **S33-X**. Lane 7 contains the Ligated DNA-AOP-NH_2 (**S32**).

qPCR analysis of DNA conjugates

qPCR was performed with the KAPA SYBR® FASTqPCR Master Mix on a qPCR cycler MyiQTM Optics Module-Thermocycler (Bio-Rad Laboratories). Preparation of PCR-Mastermix:

- 18 µL of PCR-Mastermix contain 10 µL KAPA SYBR® FASTqPCR Master Mix, 0.5 µL 10 µM forward primer, 0.5 µl 10 µM reverse primer, and 7 µL ddH_2O.

All samples were subjected to PCR cycles as follows:

- 391 pM DNA samples were diluted as follows:

**Sample**	**dilution**	**C (DNA) / fM**	**m (DNA) / pg**	**Log_{10} [Conc.]**
01	1:4	98000	10.94	4.99
02	1:16	24000	2.73	4.38
03	1:64	6100	0.68	3.79

04	1:256	1530	0.17	3.18
05	1:1024	381	4.3×10^{-2}	2.58
06	1:4096	95	1.1×10^{-2}	1.98
07	1:16384	24	0.27×10^{-2}	1.38
08	1:65536	6	6.7×10^{-4}	0.76

- 2.0 µL of the samples were added to a well in a 96 well plate. Afterwards, 18 µL of the PCR-Mastermix were added to the DNA. The PCR was conducted utilizing the following settings:

  2 min. – 95 °C

  15 s. – 95 °C } 40x

  1 min. – 60 °C }

  1 min. – 95 °C

  30 s. – 60–90 °C in 1 °C steps (melting curve)

The fluorescence raw data was processed with LinRegPCR 11.0. The C_t values were determined and plotted against $\log_{10}$ [concentration] (fM). The measurements were conducted six times, and all values are reported as mean ± SD.

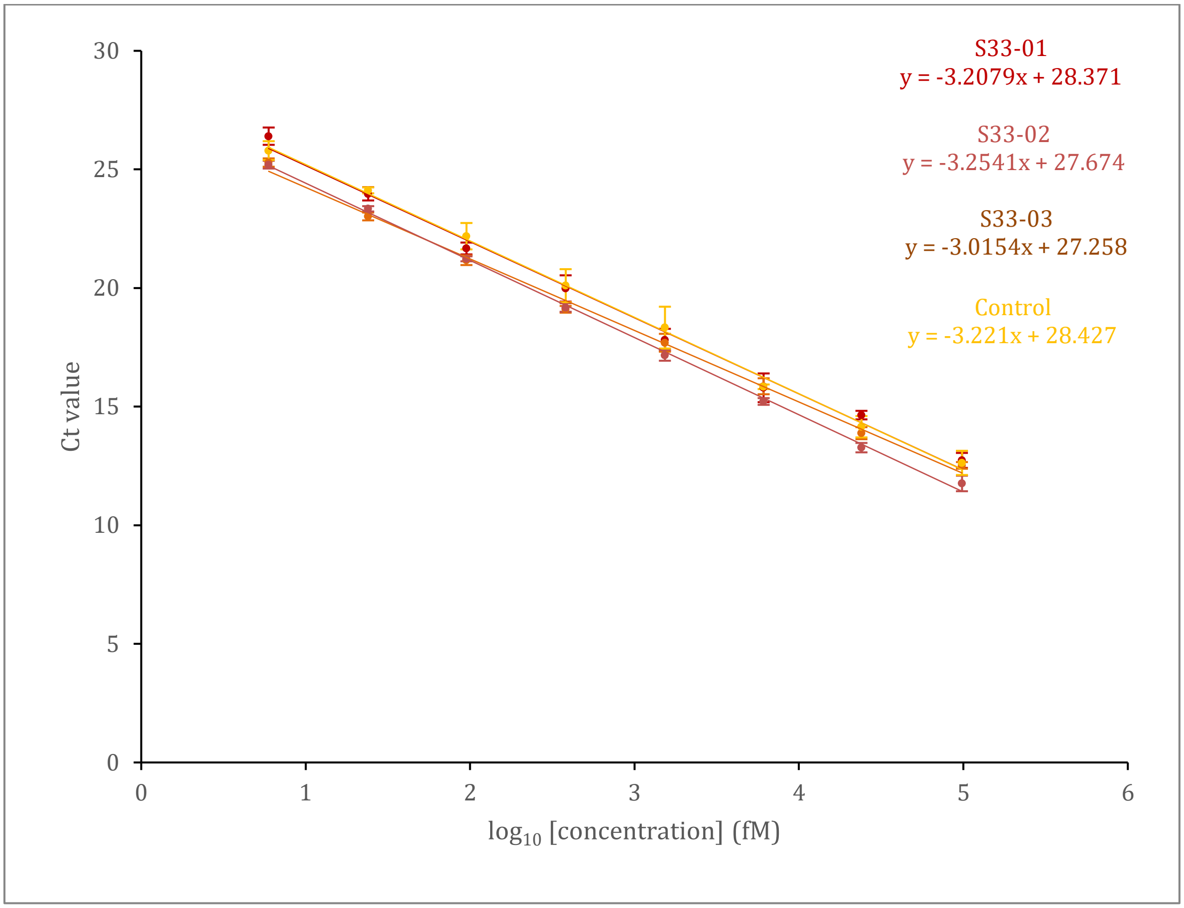

Figure S183. Quantitative PCR (qPCR) efficiency comparison between control and ruthenium-mediated *N*-arylated product reactions **S33-X** (X= sample number from 01–03).

Ligation efficiency

Ligation with excess of the Tags

Step-1: Synthesis of DNA-conjugate **37**

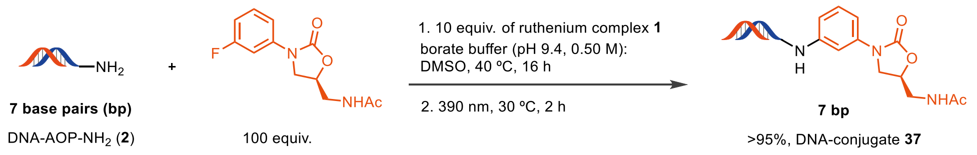

Step-2: Ligation of DNA-conjugate **37** with tags

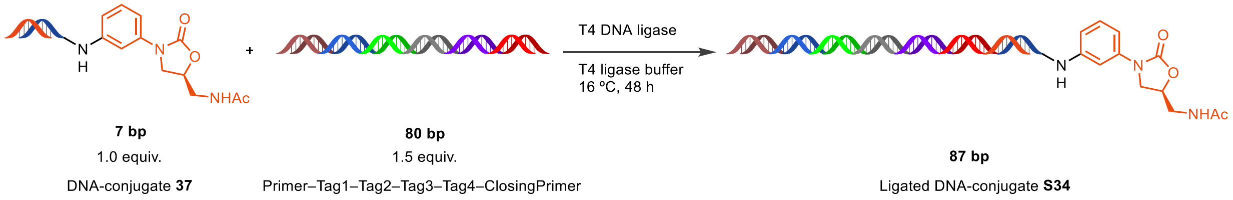

In situ formation of arene-ruthenium stock solution: Under an ambient atmosphere, a 1 mL glass GC vial equipped with a 6 mm Teflon-coated stirring bar was charged with ruthenium complex **1** (2.0 mg, 4.2 µmol, 1.0 equiv.). Next, a stock solution of (*S*)-N-((3-(3-fluorophenyl)-2-oxooxazolidin-5-yl)methyl)acetamide in DMC (420 µL, c = 0.10 M, 42 µmol, 10 equiv.) was added. The resulting reaction mixture was heated at 80 °C for 2 hours. After 2 hours, the reaction mixture was cooled to 23 °C. Next, the DMC was removed under a gentle stream of argon and 420 µL of DMSO were added to result an in situ formed stock solution of arene-ruthenium complex **C25** (420 µL, c = 0.01 M, assuming quantitative arene coordination to ruthenium).

Under an ambient atmosphere, the stock solution **SD-01** of DNA-AOP-NH_2 (**2**) in water (10 µL, c = 2.0 mM, 20 nmol, 1.0 equiv.) was added to a 1.5 mL Eppendorf tube, followed by sodium borate buffer (10 µL, pH 9.4, c = 0.50 M). To this mixture, 160 µL of DMSO was added and the solution was vortexed for 5 seconds. Next, the freshly prepared stock solution **C25** (20 µL, c = 0.01 M, 0.2 µmol, 10 equiv.) in DMSO was added. The resulting reaction mixture was vortexed for 5 seconds, transferred to a thermocycler at 40 °C, and incubated for 16 hours at 800 rpm. To the reaction mixture was added the stock solution of NaCl in water (**SR-06** , 20 µL, c = 5.0 M, 10% volume of the total reaction volume), followed by cold ethanol (−20 °C, 660 µL) to precipitate the *N*-arylated ruthenium DNA conjugate. The Eppendorf tube was placed in a freezer (−20 °C) for at least 1 hour, and then it was centrifuged at 4 °C and 11000 × g for at least 30 minutes. The supernatant was removed and the pellet was dried under air, then dissolved in 200 µL water and irradiated with a 390 nm (40 W) Kessil lamp for 2 hours, while maintaining the temperature at approximately 30 °C through cooling with a fan. The above reaction mixture was then filtered using a 3 KDa MWCO Amicon ultra centrifugal filter for desalting the sample. Next, the concentration of the DNA-conjugate **37** was adjusted to a final concentration of c = 1.0 mM using Thermo Scientific™ NanoDrop™ One^{C}.

At 4 °C, 2.0 µL of the DNA-conjugate (**37**, c = 50 µM, 0.10 nmol, 1.0 equiv.) in water was added to a 1.5 mL Eppendorf tube. Next, 3 µL of a stock solution of the DNA fragment Primer–Tag1–Tag2–Tag3–Tag4–ClosingPrimer (DNA tags for ligation) (c = 50 µM, 0.15 nmol, 1.5 equiv.) in water was added, followed by 4.0 µL of Milli-Q water and cooled to 16 °C. In another Eppendorf tube at 4 °C, 1.0 µL of 10X T4 DNA ligase buffer and 0.50 µL of T4 DNA ligase were mixed. The mixture was added to the Eppendorf tube containing the solution of amine-DNA conjugate **37** and the DNA fragment Primer–Tag1–Tag2–Tag3–Tag4–ClosingPrimer. The resulting mixture was vortexed for 5 seconds, transferred to a Thermocycler pre-cooled at 16 °C, and incubated at 16 °C for 42 hours without stirring. After 42 hours, the reaction mixture was diluted with 10 µL of Milli-Q water, and added the stock solution of NaCl in water (**SR-06**, 2.0 µL, c = 5.0 M, 10% volume of the total reaction volume), followed by cold ethanol (−20 °C, 66 µL) to precipitate the ligated DNA-conjugate **S34**. The Eppendorf tube was placed in the freezer (−20 °C) for at least 1 hour, and then it was centrifuged at 4 °C and 11000 × g for at least 30 minutes. The supernatant was removed, the pellet dried under air and dissolved in Milli-Q water to obtain the ligated DNA-conjugate **S34**. The concentration of the DNA-conjugate **S34** was adjusted to a final concentration of c = 50 µM using Thermo Scientific™ NanoDrop™ One^{C}. The ligation was analyzed by GEL electrophoresis with SYBR Green as dye, 4% agarose gel and 150 V for 15 min to 1.25 hours.

Gel electrophoresis protocol (before 50 KDa MWCO filtration)

- 4% agarose gel (1.2 g) to 30 mL of buffer

6 lanes:

- Lane 1: DNA ladder (Bio-Rad Laboratories EZ Load 20 bp Molecular Ruler)
- Lane 2: Ligated DNA-conjugate **S34** (60 ng)
- Lane 3: Primer–Tag1–Tag2–Tag3–Tag4–ClosingPrimer (60 ng)
- Lane 4: Primer–Tag1–Tag2–Tag3–Tag4–ClosingPrimer (60 ng)
- Lane 5: DNA-conjugate **37** (120 ng)
- Lane 6: DNA ladder (Bio-Rad Laboratories EZ Load 20 bp Molecular Ruler)

Gel images were processed using Bio-Rad Laboratories, Inc. Image Lab Version 6.1.0 build 7, Standard Edition.

Note: *The DNA gel was run with the sample after ethanol precipitation and before Amicon filtration. Therefore, excess of the unligated tags are visible in the DNA gel (Fig. S184, image B and C, Lane 2). Also, the absence of DNA-conjugate* ***37*** *in Lane 2 suggests that the ligation is almost quantitative with the tags. It also can be seen from the DNA gel (Fig. S184, B and C) that the ligated DNA-conjugate* ***S34*** *travels less distance compared to the unligated Tags. The ligated DNA conjugate* ***S34*** *in the Lane 2 falls within the range of 80–100 bp (reference to the DNA-ladder in Lane 1 and 8), rightly aligning with the 87 bp count corresponding to the ligated DNA conjugate* ***S34****.*

- After loading the samples, the gel was run at 150V for 15 minutes to 1.25 hours.

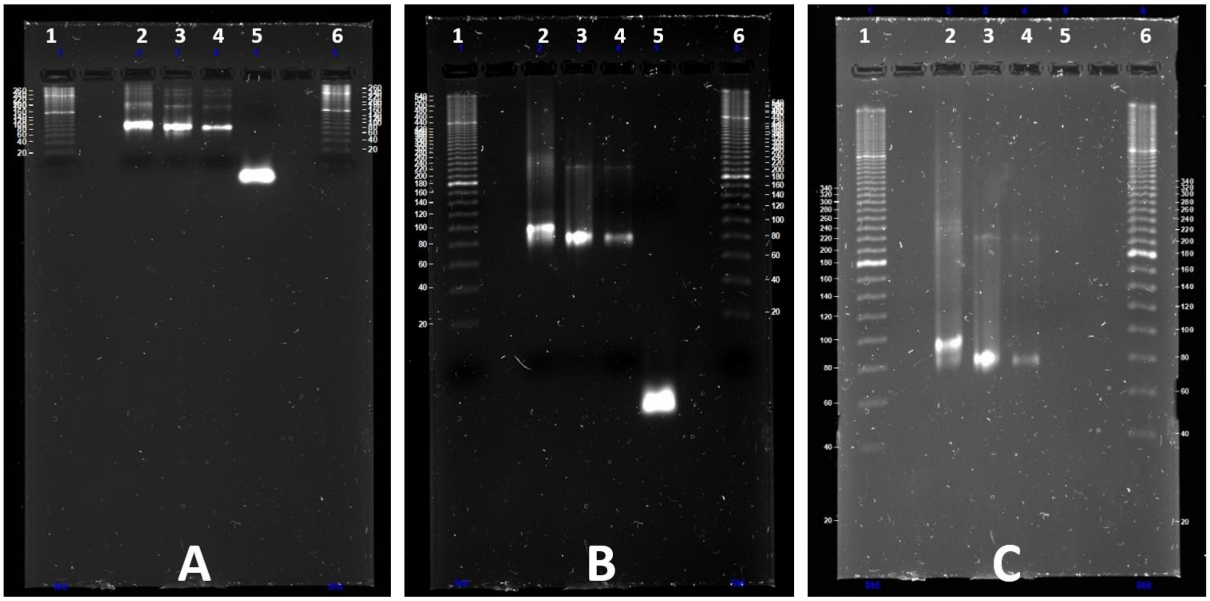

Figure S184. Images of the DNA gel. A. DNA gel running at 150 V for 15 min. B. DNA gel running at 150 V for 45 min. C. DNA gel running at 150 V for 1.25 hours to observe a better separation between the ligated samples and the tags. In the above image Lane 1 and 6 contains DNA ladder (Bio-Rad Laboratories EZ Load

20 bp Molecular Ruler). Lane 2 contains the ligated DNA-conjugate **S34**. Lane 3 and 4 contains only the tags (Primer–Tag1–Tag2–Tag3–Tag4–ClosingPrimer). Lane 5 contains the DNA-conjugate **37**.

Gel electrophoresis protocol (after 50 KDa MWCO filtration)

- 4% agarose gel (1.2 g) to 30 mL of buffer

8 lanes:

- Lane 1: DNA ladder (Bio-Rad Laboratories EZ Load 20 bp Molecular Ruler)
- Lane 2: DNA-conjugate **37** (120 ng)
- Lane 3: Primer–Tag1–Tag2–Tag3–Tag4–ClosingPrimer (45 ng)
- Lane 4: Ligated DNA-conjugate **S34** (45 ng)
- Lane 5: Ligated DNA-conjugate **S34** (45 ng)
- Lane 6: Primer–Tag1–Tag2–Tag3–Tag4–ClosingPrimer (45 ng)
- Lane 7: DNA-conjugate **37** (120 ng)
- Lane 8: DNA ladder (Bio-Rad Laboratories EZ Load 20 bp Molecular Ruler)

Gel images were processed using Bio-Rad Laboratories, Inc. Image Lab Version 6.1.0 build 7, Standard Edition.

Note: *The DNA gel was run with a sample after filtration with 50 KDa MWCO Amicon ultra centrifugal filter. Therefore, excess of the tags have been removed from the ligation mixture and hence it is not visible in the DNA gel (Fig. S185, Lane 4 and 5). The ligated DNA conjugate* ***S34*** *in the Lane 4 and 5 falls within the range of 80–100 bp (reference to the DNA-ladder in Lane 1 and 8), rightly aligning with the 87 bp count corresponding to the ligated DNA conjugate* ***S34****.*

- After loading the samples, the gel was run at 150V for 45 minutes.

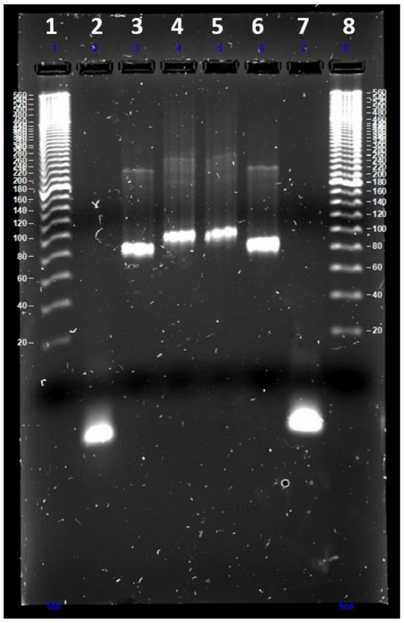

Figure S185. Image of the DNA gel running at 150 V for 45 min. In the above image Lane 1 and 8 contains DNA ladder (Bio-Rad Laboratories EZ Load 20 bp Molecular Ruler). Lane 2 and 7 contains the DNA-conjugate **37**. Lane 3 and 6 contains only the tags (Primer–Tag1–Tag2–Tag3–Tag4–ClosingPrimer). Lane 4

and 5 contains the ligated DNA-conjugate **S34**.

Ligation with an equimolar proportion of DNA-conjugate amine and Tags:

Step-1: Synthesis of DNA-conjugate **37**

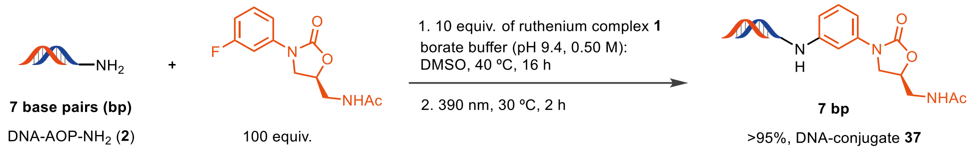

Step-2: Ligation of DNA-conjugate **37** with tags

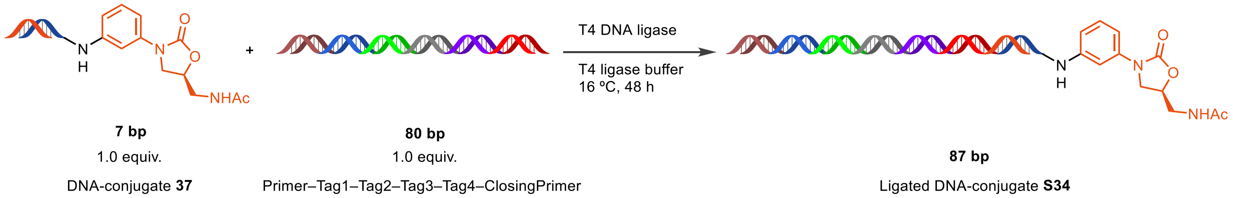

At 4 °C, 5.0 µL of the DNA-conjugate (**37**, c = 0.10 mM, 0.50 nmol, 1.0 equiv.) in water was added to a 1.5 mL Eppendorf tube. Next, 5.0 µL of a stock solution of the DNA fragment Primer–Tag1–Tag2–Tag3–Tag4–ClosingPrimer (DNA tags for ligation) (c = 0.10 mM, 0.50 nmol, 1.0 equiv.) in water was added and cooled to 16 °C. In another Eppendorf tube at 4 °C, 1.0 µL of 10X T4 DNA ligase buffer and 0.50 µL of T4 DNA ligase were mixed. The mixture was added to the Eppendorf tube containing the solution of amine-DNA-conjugate **37** and the DNA fragment Primer–Tag1–Tag2–Tag3–Tag4–ClosingPrimer. The resulting mixture was vortexed for 5 seconds, transferred to a Thermocycler pre-cooled at 16 °C, and incubated at 16 °C for 42 hours without stirring. After 42 hours, the reaction mixture was diluted with 10 µL of Milli-Q water, and added the stock solution of NaCl in water (**SR-06**, 2.0 µL, c = 5.0 M, 10% volume of the total reaction volume), followed by cold ethanol (−20 °C, 66 µL) to precipitate the ligated DNA-conjugate **S34**. The Eppendorf tube was placed in the freezer (−20 °C) for at least 1 hour, and then it was centrifuged at 4 °C and 11000 × g for at least 30 minutes. The supernatant was removed, the pellet dried under air and dissolved in 100 µL Milli-Q water to obtain the ligated DNA-conjugate **S34**. Next, the solution of the DNA-conjugate **S34** was filtered using a 10 kDa MWCO Amicon ultra centrifugal filter for desalting the solution. The concentration of the DNA-conjugate **S34** was adjusted to a concentration of c = 0.10 mM using Thermo Scientific™ NanoDrop™ One^{C}. The ligation was analyzed by GEL electrophoresis with SYBR Green as dye, 4% agarose gel and 150 V for 15 min to 1.25 hours.

Gel electrophoresis protocol (After 10 KDa MWCO filtration)

- 4% agarose gel (1.2 g) to 30 mL of buffer

8 lanes:

- Lane 1: DNA ladder (Bio-Rad Laboratories EZ Load 20 bp Molecular Ruler)
- Lane 2: DNA-conjugate **37** (120 ng)
- Lane 3: Primer–Tag1–Tag2–Tag3–Tag4–ClosingPrimer (60 ng)

- Lane 4: Ligated DNA-conjugate **S34** (60 ng)
- Lane 5: Ligated DNA-conjugate **S34** (60 ng)
- Lane 6: Primer–Tag1–Tag2–Tag3–Tag4–ClosingPrimer (60 ng)
- Lane 7: DNA-conjugate **37** (120 ng)
- Lane 8: DNA ladder (Bio-Rad Laboratories EZ Load 20 bp Molecular Ruler)

Gel images were processed using Bio-Rad Laboratories, Inc. Image Lab Version 6.1.0 build 7, Standard Edition.

Note: *The DNA gel was run with the above sample after filtration with 10 KDa MWCO Amicon ultra centrifugal filter. The ligation was performed with 1:1 ratio of tags and the DNA-conjugate* ***37*** *and hence no detectable unligated tags are visible in the DNA gel, implying almost quantitative ligation of the tags with the DNA-conjugate* ***37*** *(Fig. S186, image B and C, Lane 4 and 5). The ligated DNA conjugate* ***S34*** *in the Lane 4 and 5 falls within the range of 80–100 bp (reference to the DNA-ladder in Lane 1 and 8), rightly aligning with the 87 bp count corresponding to the ligated DNA conjugate* ***S34****.*

- After loading the samples, the gel was run at 150V for 15 minutes to 1.25 hours.

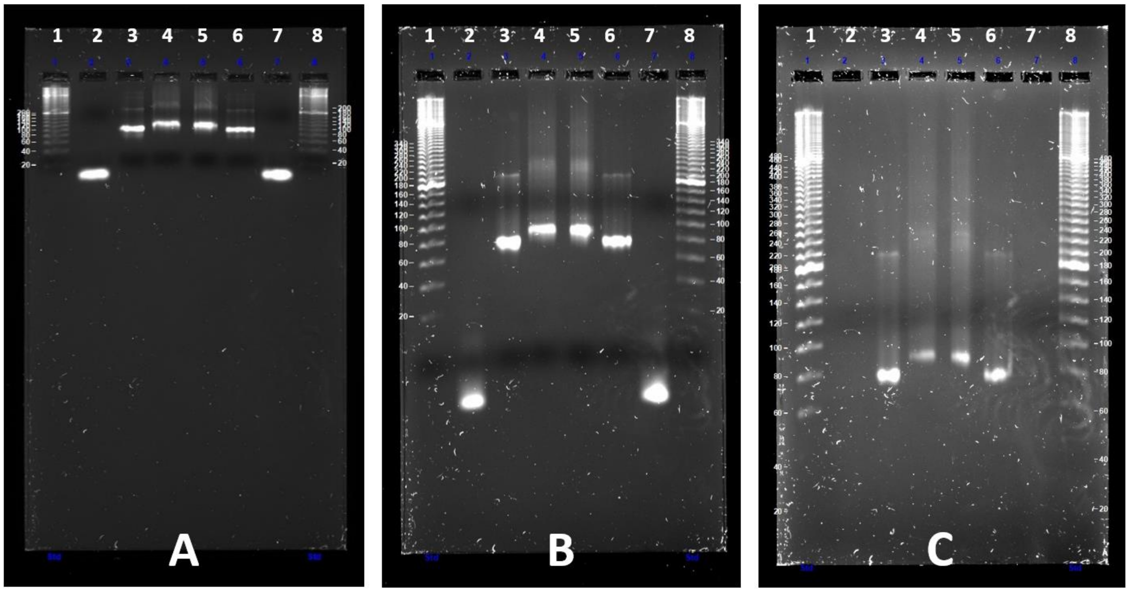

Figure S186. Images of the DNA gel. A. DNA gel running at 150 V for 15 min. B. DNA gel running at 150 V for 45 min. C. DNA gel running at 150 V for 1.25 hours to observe a better separation between the ligated samples and the tags. Image of the DNA gel running at 150 V after 45 min. In the above image Lane 1 and 8 contains DNA ladder (Bio-Rad Laboratories EZ Load 20 bp Molecular Ruler). Lane 2 and 7 contains the DNA-conjugate **37**. Lane 3 and 6 contains only the tags (Primer–Tag1–Tag2–Tag3–Tag4–ClosingPrimer). Lane 4 and 5 contains the ligated DNA-conjugate **S34**.

NMR reaction monitoring

NMR reaction monitoring was conducted to evaluate the reactivity of different haloarene–ruthenium complexes in nucleophilic aromatic substitution (S_NAr) reactions. The study involved fluorobenzene-ruthenium complex **11**, chlorobenzene-ruthenium complex **7**, bromobenzene-ruthenium complex **S7**, and iodobenzene-ruthenium complexe **S8** as electrophiles, with benzylamine serving as the amine nucleophile. Reactions were monitored using a 600 MHz NMR spectrometer at 40 °C.

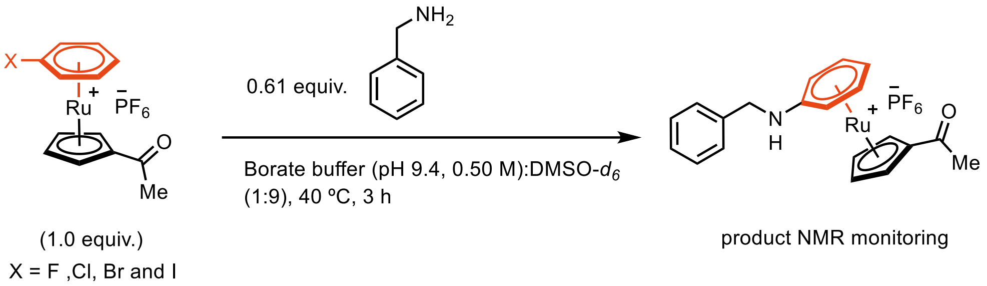

For the study, a mixture of DMSO-d_6 and sodium borate buffer (pH 9.4, c = 0.50 M in water) in the ratio 1:9 was used. The following equivalents were used for performing the NMR analysis:

- Benzylamine (2.1 µL, 19 µmol, 0.61 equiv.)
- Fluorobenzene complex **11** (13.9 mg, 31.2 µmol, 1.0 equiv.)
- Chlorobenzene complex **7** (14.5 mg, 31.2 µmol, 1.0 equiv.)
- Bromobenzene complex **S7** (15.9 mg, 31.2 µmol, 1.0 equiv.)
- Iodobenzene complex **S8** (17.4 mg, 31.2 µmol, 1.0 equiv.)

Under an ambient atmosphere, a 4 mL borosilicate vial equipped with a Teflon coated magnetic stirring bar was charged with the halobenzene ruthenium complex (solid (mg), 31.2 µmol, 1.0 equiv.), followed by 0.9 mL of DMSO-d_6. The mixture was properly mixed, resulting in a solution using a 1 mL syringe. Subsequently, 0.10 mL of sodium borate buffer was added and the solution was thoroughly mixed. Benzylamine (2.1 µL, 19 µmol, 0.61 equiv.) was then added to the reaction mixture using a micropipette, followed by additional mixing. Immediately after, 0.50 mL of the resulting reaction mixture was transferred to an NMR tube for analysis.

This study was conducted to qualitatively assess the reactivity of aliphatic amines with various haloarenes in the presence of a buffered aqueous environment. In the case of the fluorobenzene–ruthenium complex, complete product conversion was observed within 2–5 minutes. Therefore, for clarity, the product outcome from the fluorinated arene (ArF) system was extrapolated.

Due to the complexity of the NMR spectra, reaction conversion was referenced against the initial concentration of benzylamine (19 mM).

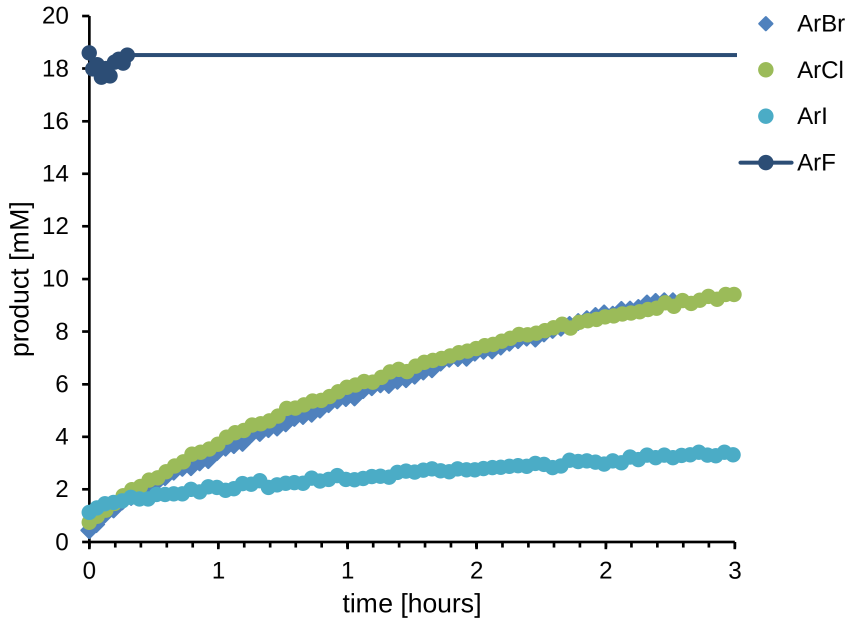

Figure S187. Product vs time plot.

Proof-of-concept mock-library synthesis

Library design: For the synthesis of the mock-library, three amine–DNA conjugates (**S21**, **S17**, and **S23**) were pooled in a 1.5 mL Eppendorf tube and mixed in a 1:1:1 ratio. The resulting mixture was divided into six wells, to which six different *in situ*–activated aryl halides were added (according to **Scheme S3**). The reactions were incubated at 40 °C for 3 h with shaking at 800 rpm, followed by irradiation with 390 nm Kessil lamp (40 W) for 1 h, affording the 18-member mock-library of *N*-arylation products.

Procedure for Library synthesis: Under an ambient atmosphere, the solution of amine–DNA conjugates **S21** (10 µL, c = 2.0 mM, 20 nmol), **S17** (10 µL, c = 2.0 mM, 20 nmol), and **S23** (10 µL, c = 2.0 mM, 20 nmol) in water were combined in a 1.5 mL Eppendorf tube in a 1:1:1 ratio to give a pooled mixture of amine–DNA conjugates (30 µL, c = 2.0 mM, 60 nmol). Next, 3.0 µL of this pool were added to six separate Eppendorf tubes, followed by sodium borate buffer (3.0 µL, pH 9.4, c = 0.50 M) and 48 µL DMSO. The mixtures were vortexed for 5 seconds and then freshly prepared stock solutions of ruthenium–arene complexes (6.0 µL, c = 0.01 M, 0.06 µmol, 10 equiv.) in DMSO were added to each Eppendorf tubes according to Scheme-**1**. The mixtures were vortexed for 5 seconds, transferred to a thermocycler, and incubated at 40 °C for 3 h with shaking at 800 rpm. After 3 hours, a stock solution of cysteine (10 µL, c = 60 mM, 0.60 µmol, 10 equiv.) in water was added to each of the Eppendorf tubes and left stirring at 40 °C for 15 min. Next, the stock solution of NaCl in water (**SR-06**, 7.0 µL, c = 5.0 M, 10% volume of the total reaction volume), followed by cold ethanol (−20 °C, 231 µL) were added to each of the Eppendorf tubes to precipitate the DNA-conjugates. The Eppendorf tubes were placed in a freezer (−20 °C) for at least 1 hour, and then it was centrifuged at 4 °C and 11000 × g for at least 30 minutes. The supernatants were removed and the pellets were dried under air, then to each of the Eppendorf tubes were

added 60 µL Milli-Q water and irradiated with a 390 nm (40 W) Kessil lamp for 1 hours, while maintaining the temperature at approximately 30 °C through cooling with a fan. The above reaction mixtures were then filtered using a 3 KDa MWCO Amicon ultra centrifugal filter. Next, 10 µL of the DNA-conjugates were diluted to 40 µL of Milli-Q water for LC–MS analysis.

Note: For wells 4 and 5, where the acid method was used for the arene complexation, 6.0 µL sodium borate buffer (pH 9.4, c = 0.50 M) was added instead of 3.0 µL.

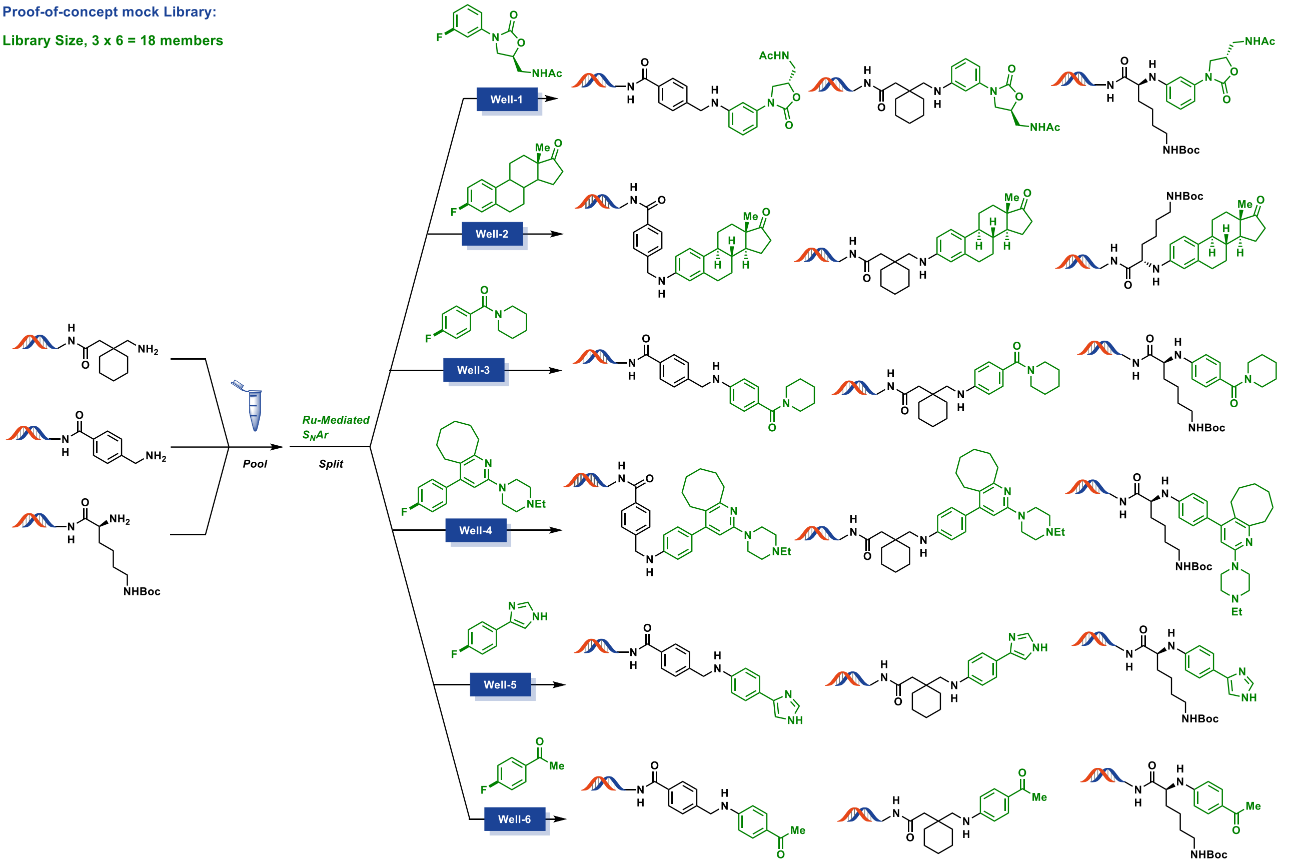

Scheme S3. Synthesis of an 18-membered proof-of-concept mock library.

Well-1:

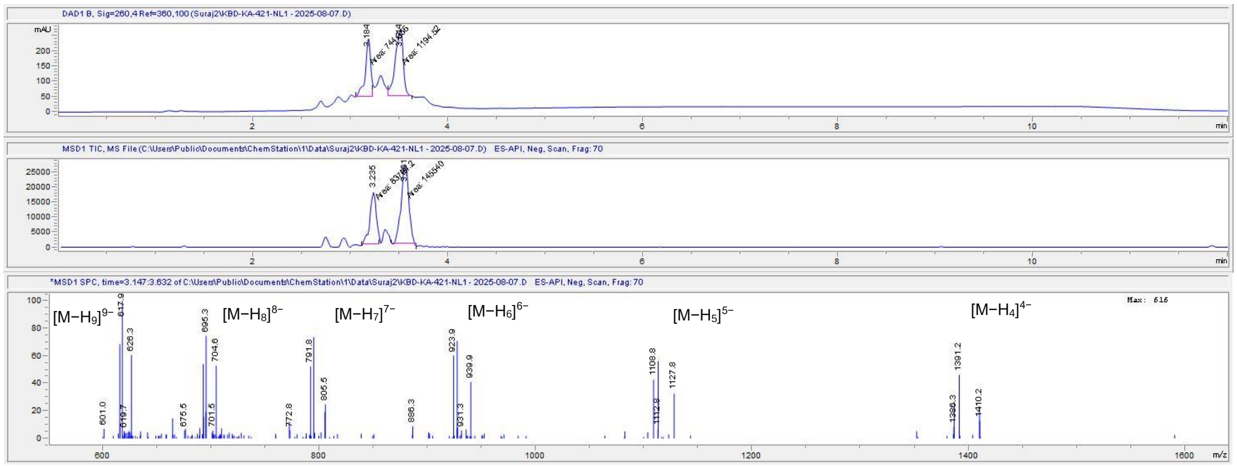

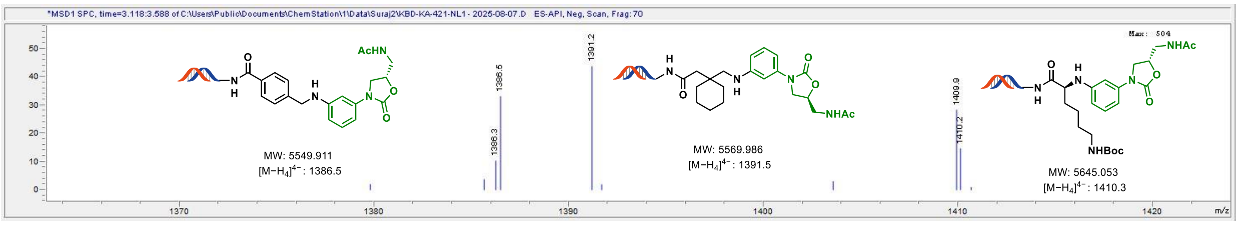
*MSD1 SPC, time=3.118:3.588 of C:\Users\Public\Documents\ChemStation\1\Data\Suraj2\KBD-KA-421-NL1 - 2025-08-07.D ES-API, Neg, Scan, Frag: 70
AcHN
MW: 5549.911
[M−H4]4−: 1386.5
MW: 5569.986
[M−H4]4−: 1391.5
NHAc
NHBoc
MW: 5645.053
[M−H4]4−: 1410.3
1386.3
1386.5
1391.2
1409.9
1410.2
m/z

Well-2:

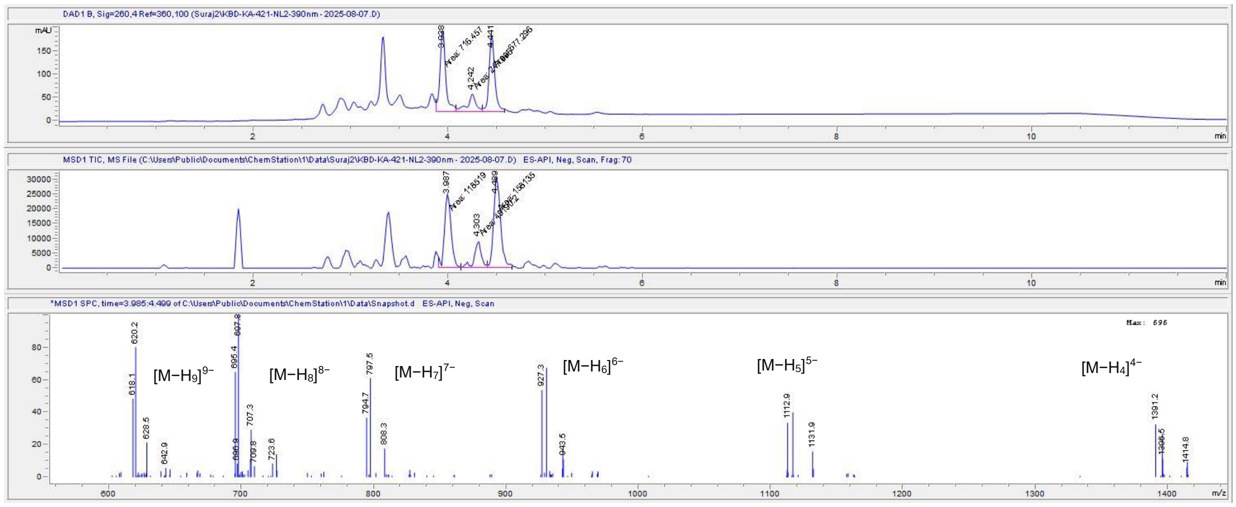
DAD1 B, Sig=260,4 Ref=360,100 (Suraj2\KBD-KA-421-NL2-390nm - 2025-08-07.D)
MSD1 TIC, MS File (C:\Users\Public\Documents\ChemStation\1\Data\Suraj2\KBD-KA-421-NL2-390nm - 2025-08-07.D) ES-API, Neg, Scan, Frag: 70
*MSD1 SPC, time=3.985:4.499 of C:\Users\Public\Documents\ChemStation\1\Data\Snapshot.d ES-API, Neg, Scan
[M−H9]9−
[M−H8]8−
[M−H7]7−
[M−H6]6−
[M−H5]5−
[M−H4]4−
620.2
618.1
628.5
695.4
697.8
707.3
794.7
797.5
808.3
927.3
943.5
1112.9
1131.9
1391.2
1414.8

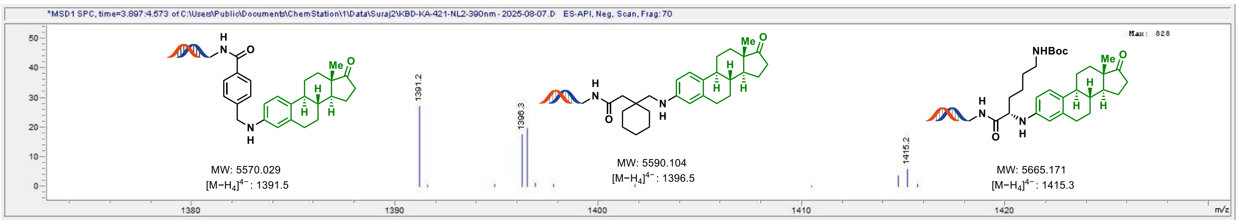
*MSD1 SPC, time=3.897:4.573 of C:\Users\Public\Documents\ChemStation\1\Data\Suraj2\KBD-KA-421-NL2-390nm - 2025-08-07.D ES-API, Neg, Scan, Frag: 70
MW: 5570.029
[M−H4]4−: 1391.5
MW: 5590.104
[M−H4]4−: 1396.5
NHBoc
MW: 5665.171
[M−H4]4−: 1415.3
1391.2
1396.3
1415.2
m/z

Well-3:

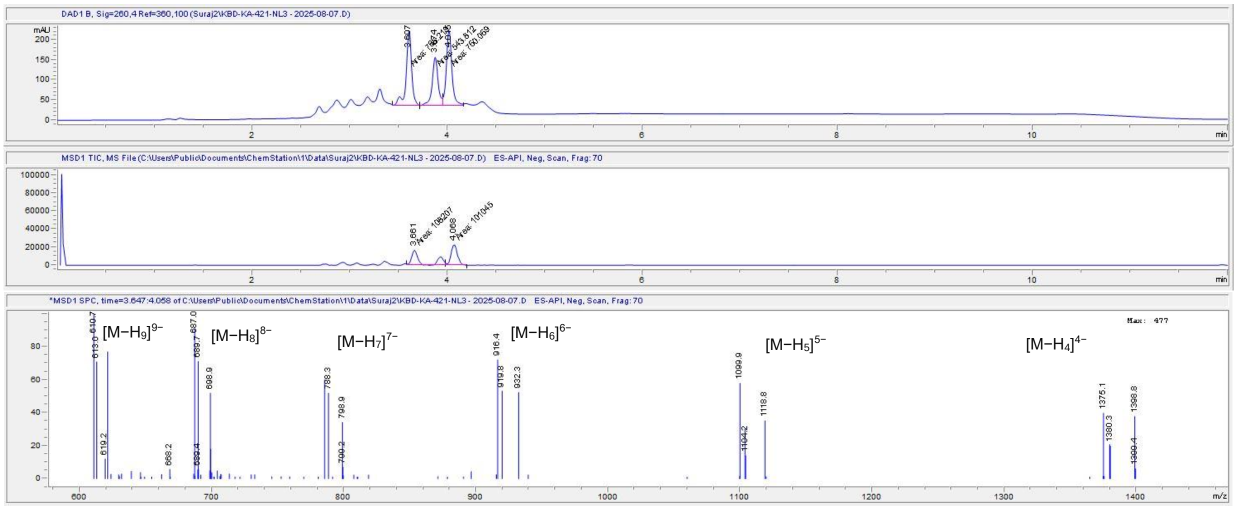
DAD1 B, Sig=260,4 Ref=360,100 (Suraj2\KBD-KA-421-NL3 - 2025-08-07.D)
MSD1 TIC, MS File (C:\Users\Public\Documents\ChemStation\1\Data\Suraj2\KBD-KA-421-NL3 - 2025-08-07.D) ES-API, Neg, Scan, Frag: 70
*MSD1 SPC, time=3.647:4.058 of C:\Users\Public\Documents\ChemStation\1\Data\Suraj2\KBD-KA-421-NL3 - 2025-08-07.D ES-API, Neg, Scan, Frag: 70
[M−H9]9−
[M−H8]8−
[M−H7]7−
[M−H6]6−
[M−H5]5−
[M−H4]4−
619.2
668.2
698.9
788.3
798.9
916.4
919.8
932.3
1099.9
1104.2
1118.8
1375.1
1380.3
1398.8

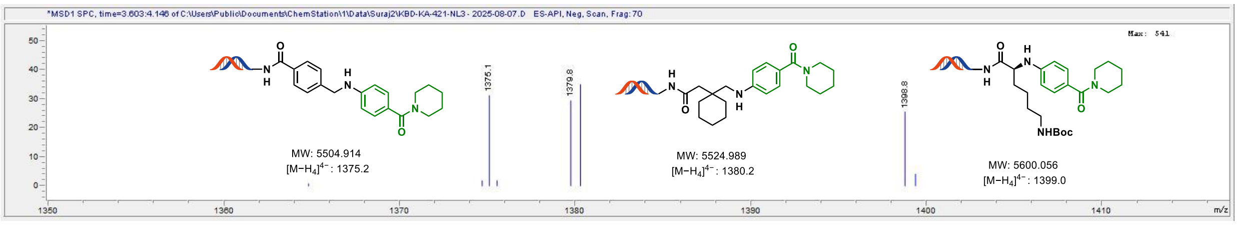
*MSD1 SPC, time=3.603:4.146 of C:\Users\Public\Documents\ChemStation\1\Data\Suraj2\KBD-KA-421-NL3 - 2025-08-07.D ES-API, Neg, Scan, Frag: 70
Max: 541
MW: 5504.914
[M−H4]4− : 1375.2
MW: 5524.989
[M−H4]4− : 1380.2
MW: 5600.056
[M−H4]4− : 1399.0
NHBoc
1375.1
1379.8
1398.8
m/z

Well-4:

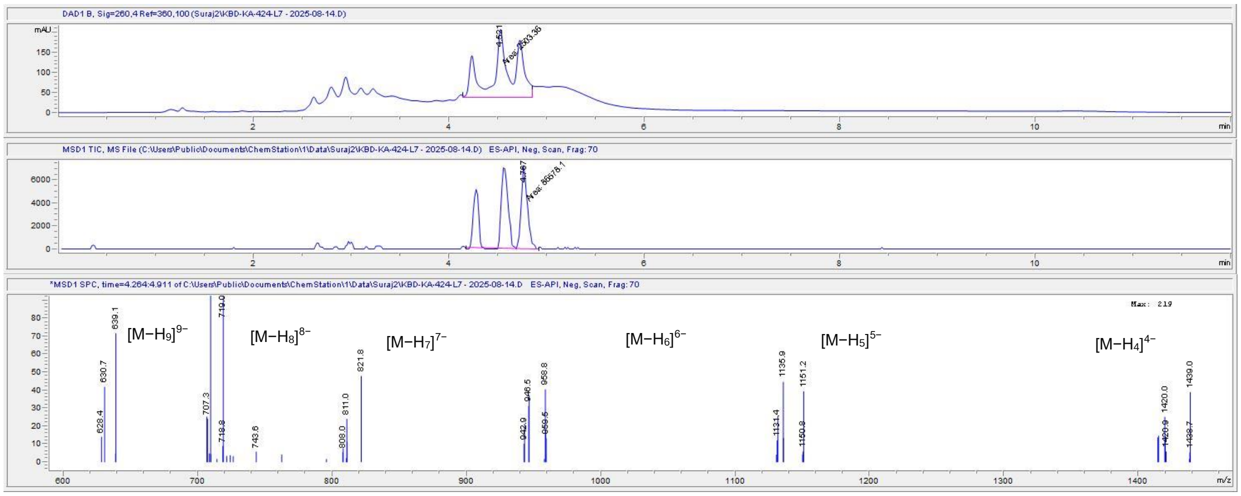
DAD1 B, Sig=260,4 Ref=360,100 (Suraj2\KBD-KA-424-L7 - 2025-08-14.D)
MSD1 TIC, MS File (C:\Users\Public\Documents\ChemStation\1\Data\Suraj2\KBD-KA-424-L7 - 2025-08-14.D) ES-API, Neg, Scan, Frag: 70
*MSD1 SPC, time=4.264:4.911 of C:\Users\Public\Documents\ChemStation\1\Data\Suraj2\KBD-KA-424-L7 - 2025-08-14.D ES-API, Neg, Scan, Frag: 70
Max: 219
[M−H9]9−
[M−H8]8−
[M−H7]7−
[M−H6]6−
[M−H5]5−
[M−H4]4−
min
m/z

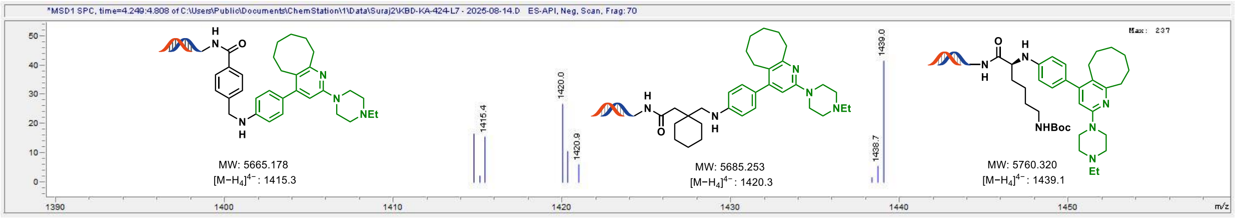
*MSD1 SPC, time=4.249:4.808 of C:\Users\Public\Documents\ChemStation\1\Data\Suraj2\KBD-KA-424-L7 - 2025-08-14.D ES-API, Neg, Scan, Frag: 70
Max: 237
MW: 5665.178
[M−H4]4− : 1415.3
MW: 5685.253
[M−H4]4− : 1420.3
MW: 5760.320
[M−H4]4− : 1439.1
NEt
NHBoc
1415.4
1420.0
1420.9
1438.7
1439.0
m/z

Well-5:

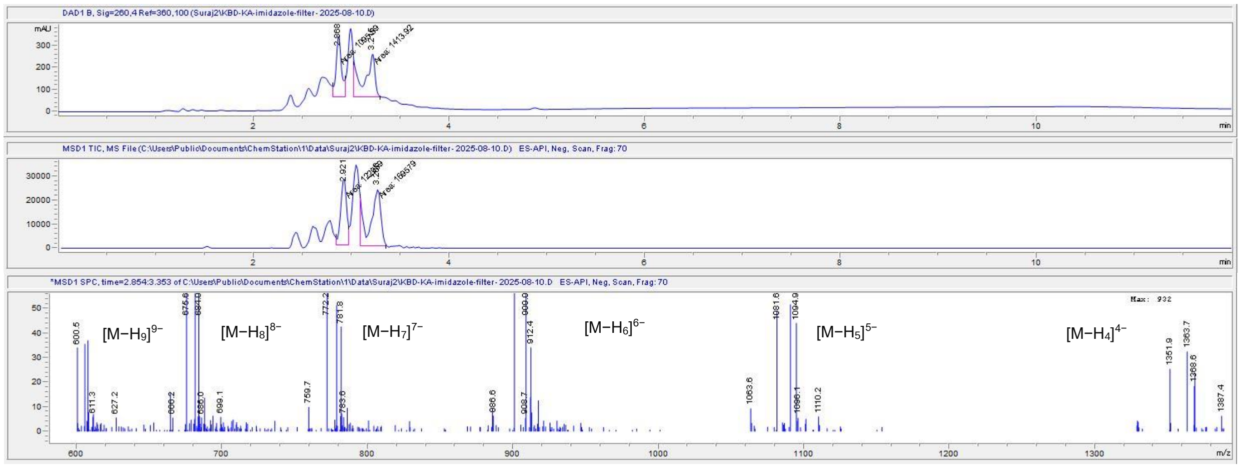
DAD1 B, Sig=260,4 Ref=360,100 (Suraj2\KBD-KA-imidazole-filter - 2025-08-10.D)
MSD1 TIC, MS File (C:\Users\Public\Documents\ChemStation\1\Data\Suraj2\KBD-KA-imidazole-filter - 2025-08-10.D) ES-API, Neg, Scan, Frag: 70
*MSD1 SPC, time=2.854:3.353 of C:\Users\Public\Documents\ChemStation\1\Data\Suraj2\KBD-KA-imidazole-filter - 2025-08-10.D ES-API, Neg, Scan, Frag: 70
Max: 932
[M−H9]9−
[M−H8]8−
[M−H7]7−
[M−H6]6−
[M−H5]5−
[M−H4]4−
min
m/z

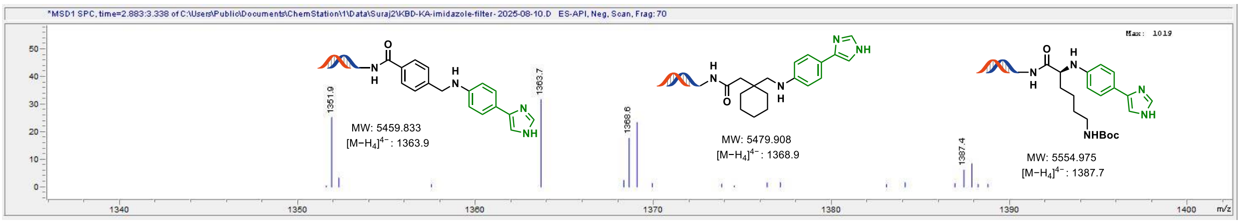

Well-6:

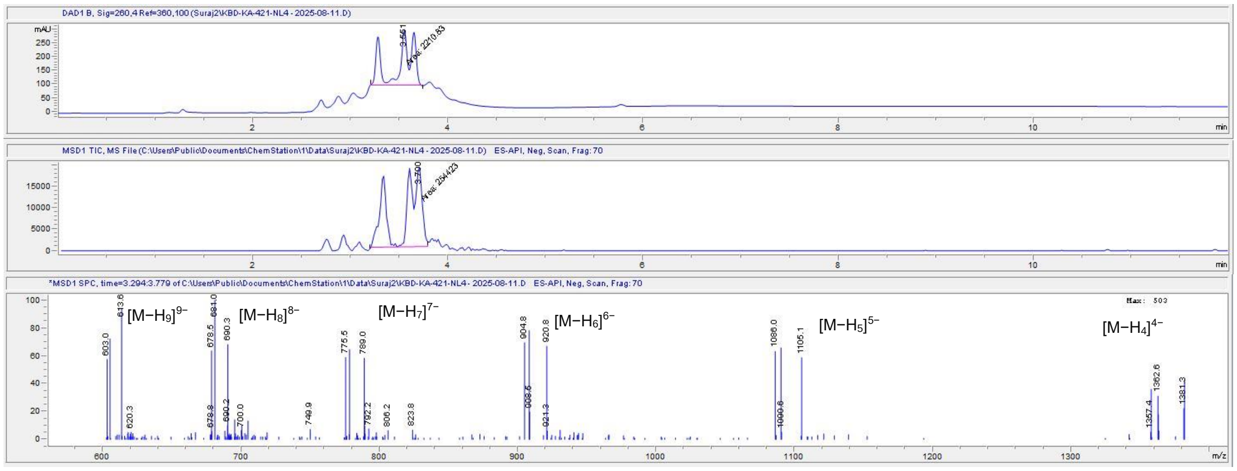

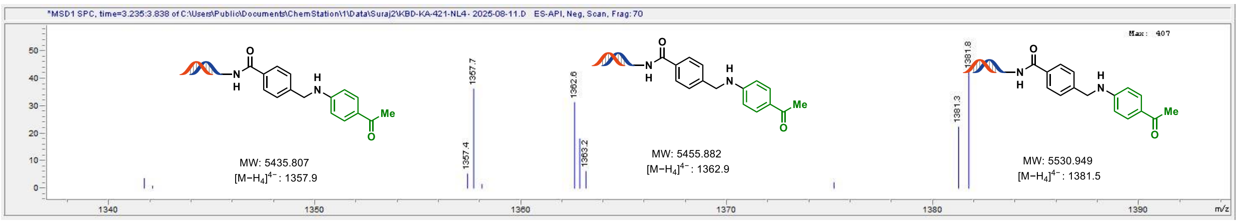

Note: The mass fragments corresponding to all 18 members of the mock library were detected. The molecular weights and mass fragments of each library member have been assigned in the LC–MS chromatograms shown above. For better clarity, zoomed-in views of the mass spectra ($[M{-}H_4]^{4-}$ ionization fragments) corresponding to each well have been provided.

Quantitative NMR (qNMR) of ruthenium complex 1

Details:

Relaxation delay: 35 sec

Number Scans: 16

Size of FID: 65536

Spectra offset 6.175 (3089.62 Hz) ppm, width 19.9875 ppm

Frequency offset (2nd nucleus): 6.175 (3089.62 Hz)

Complex with dodecylchloride:

$$Molar\ ratio = \frac{\frac{I(cpd)}{nH(cpd)}}{\frac{I(std)}{nH(std)}} = \frac{\frac{0.175}{2}}{\frac{1.000}{2}} = 0.175$$

$$wt\% = \frac{mg(std) \times MW(cpd) \times molar\ ratio \times P(std)}{mg(cpd) \times MW(std)} \times 100$$

$$wt\% = \frac{27.47 \times 476.32 \times 0.175 \times 1.00}{11.48 \times 204.78} \times 100$$

$$wt\% = 97.4\%$$

X-RAY CRYSTALLOGRAPHIC ANALYSIS

Single crystals suitable for X-ray crystallographic analysis were obtained by vial in vial gas phase diffusion of diethyl ether into a concentrated MeCN solution. The atoms are depicted with 50% probability ellipsoids. The crystallographic data is summarized in the following tables.

Ruthenium complex 1 (CCDC = 2432119)

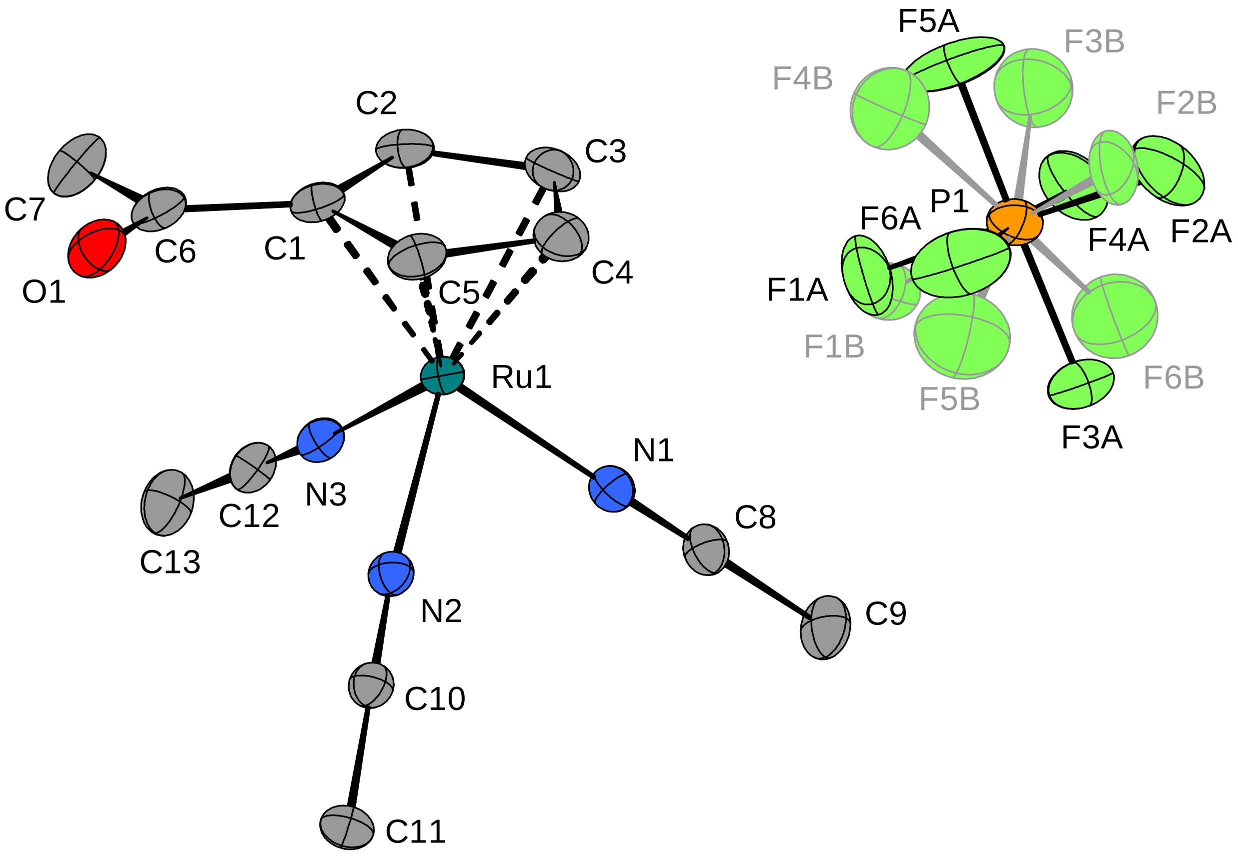

Fig. S188. X-ray structure of compound **1**.

Table S2 Crystal data and structure refinement of compound **1**:

Identification code	15741
Empirical formula	$C_{13}H_{16}F_6N_3OPRu$
Color	yellow
Formula weight	476.33 g mol^{-1}
Temperature	100(2) K
Wavelength	0.71073 Å

Crystal system	Monoclinic	
Space group	***P*2₁/*c*, (no. 14)**	
Unit cell dimensions	a = 14.4779(8) Å	α = 90°.
	b = 5.8637(3) Å	β = 90.578(3)°.
	c = 20.3635(11) Å	γ = 90°.
Volume	1728.65(16) $Å^3$	
Z	4	
Density (calculated)	1.830 $Mg \cdot m^{-3}$	
Absorption coefficient	1.067 mm^{-1}	
F(000)	944 e	
Crystal size	0.58 x 0.101 x 0.06 mm^3	
θ range for data collection	2.434 to 33.244°.	
Index ranges	$-22 \le h \le 22$, $-9 \le k \le 9$, $-31 \le l \le 31$	
Reflections collected	169338	
Independent reflections	6619 [R_{int} = 0.0470]	
Reflections with I>2σ (I)	6156	
Completeness to θ = 25.242°	99.8 %	
Absorption correction	Numerical	
Max. and min. transmission	0.9951 and 0.7051	
Refinement method	Full-matrix least-squares on F^2	
Data / restraints / parameters	6619 / 24 / 284	
Goodness-of-fit on F^2	1.070	
Final R indices [I>2σ (I)]	R_1 = 0.0215	wR^2 = 0.0522
R indices (all data)	R_1 = 0.0242	wR^2 = 0.0541
Extinction coefficient	n/a	
Largest diff. peak and hole	0.751 and −0.675 $e \cdot Å^{-3}$	

SPECTROSCOPIC DATA

^{1}H NMR spectrum of S4

600 MHz, CD_3CN, 298 K

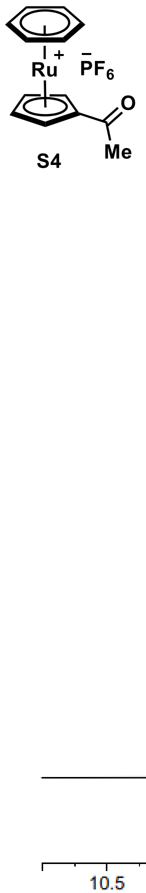

S4

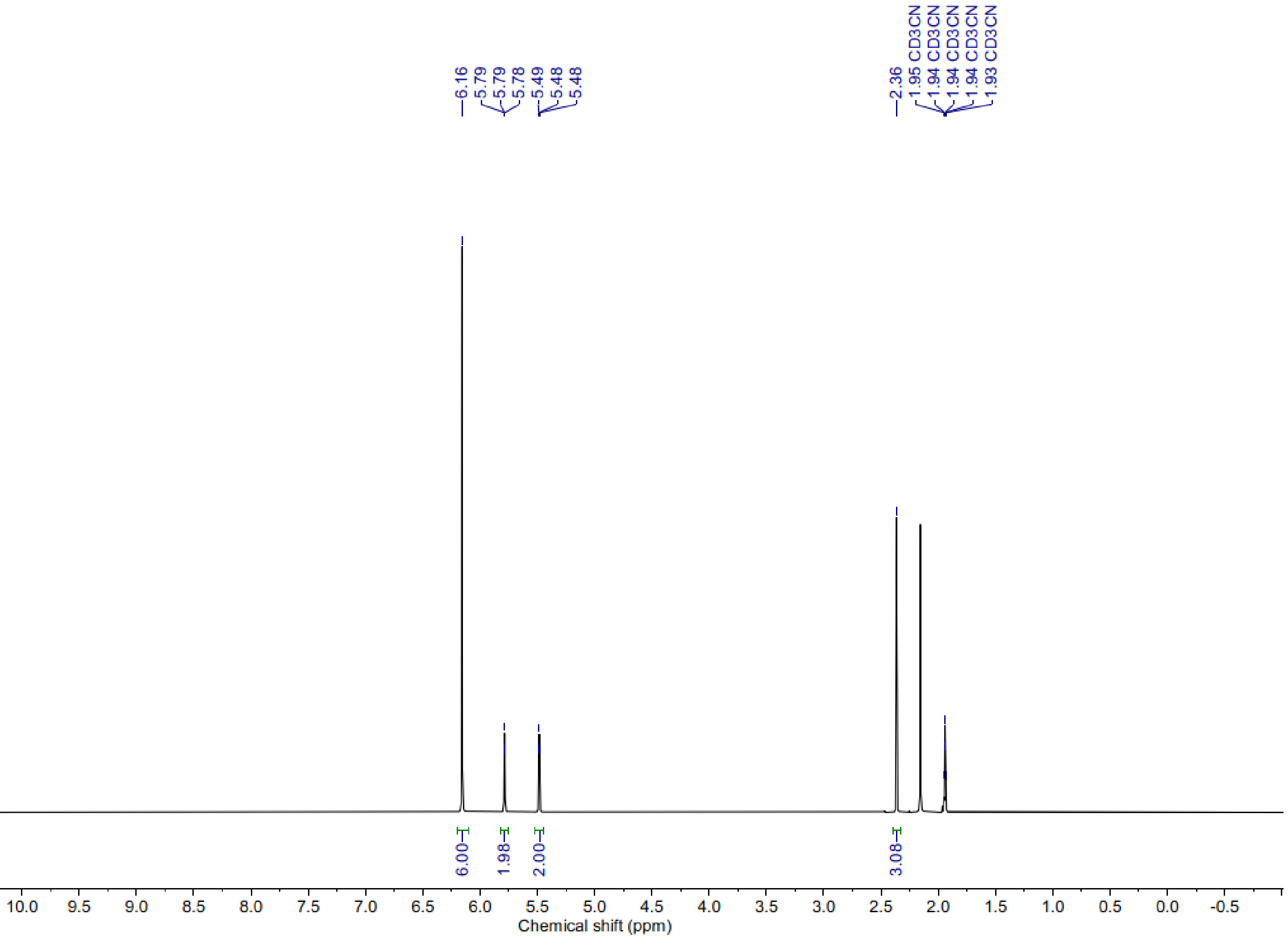

^{13}C NMR spectrum of S4

151 MHz, CD_3CN, 298 K

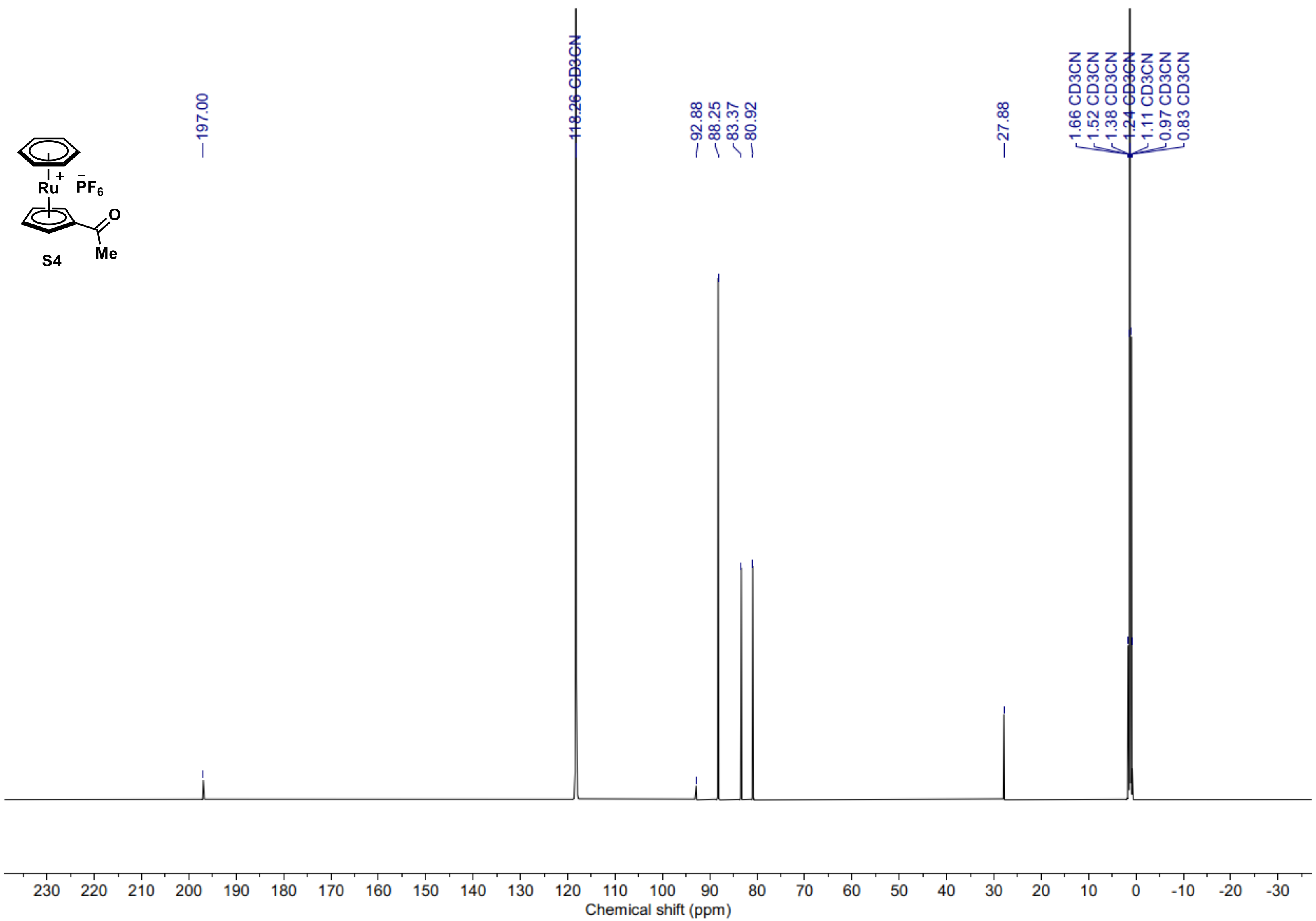

^{19}F NMR spectrum of S4

565 MHz, CD_3CN, 298 K

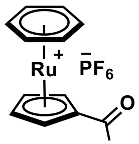

S4

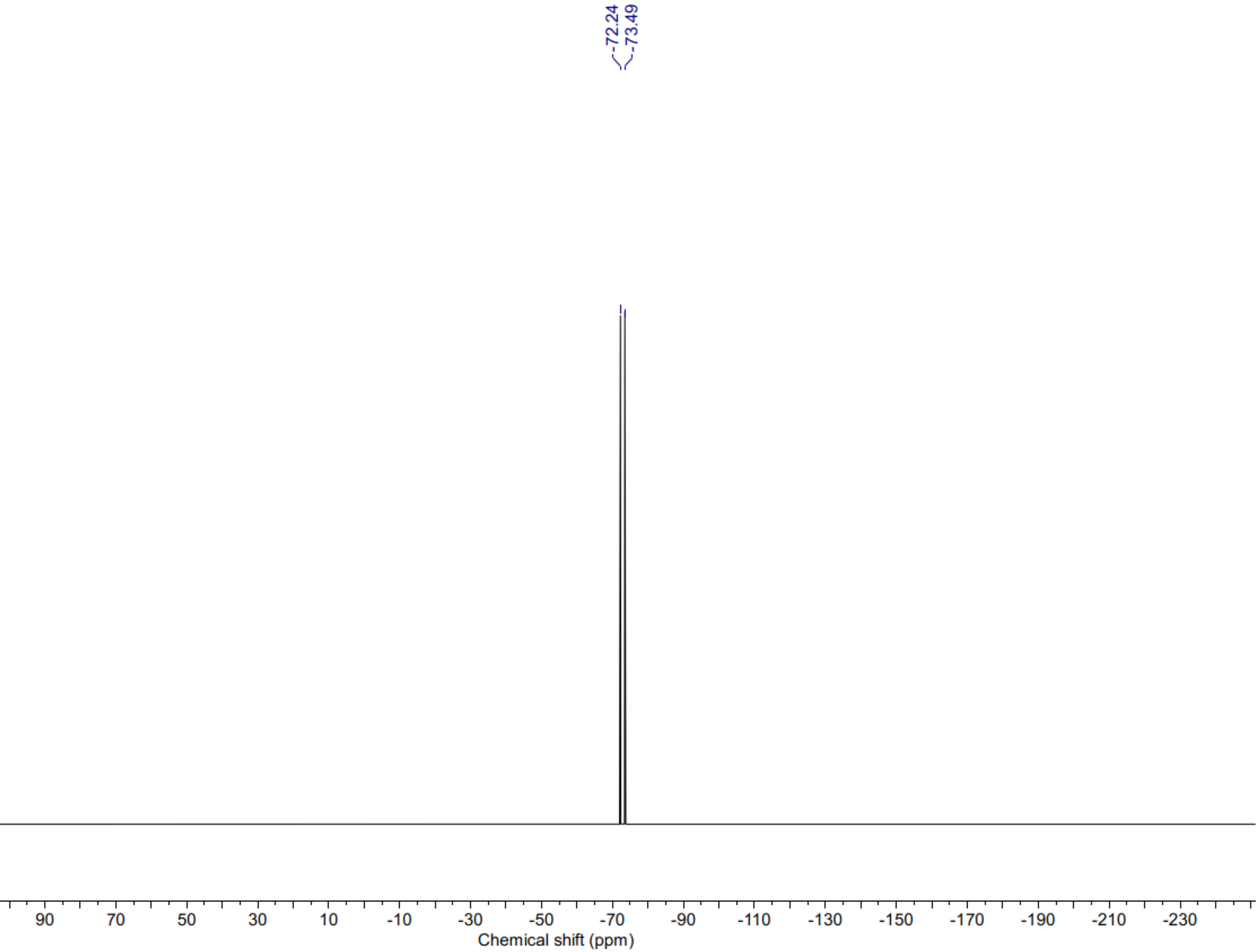

^{31}P NMR spectrum of S4

243 MHz, CD_3CN, 298 K

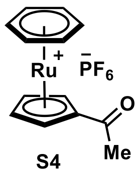

S4

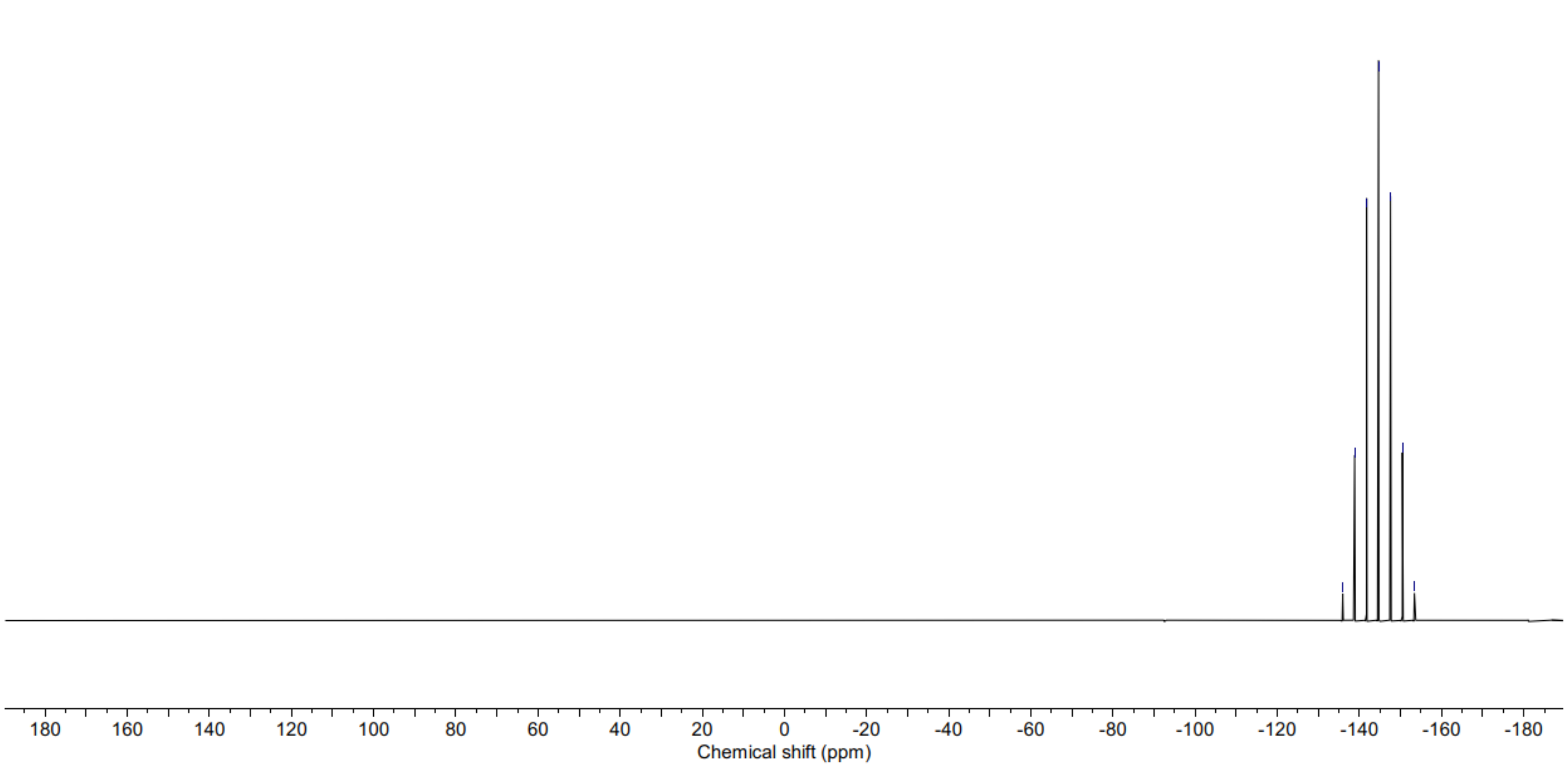

^{1}H NMR spectrum of 1

600 MHz, CD_3CN, 298 K

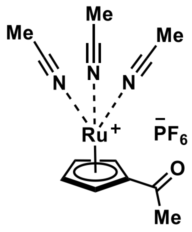

1

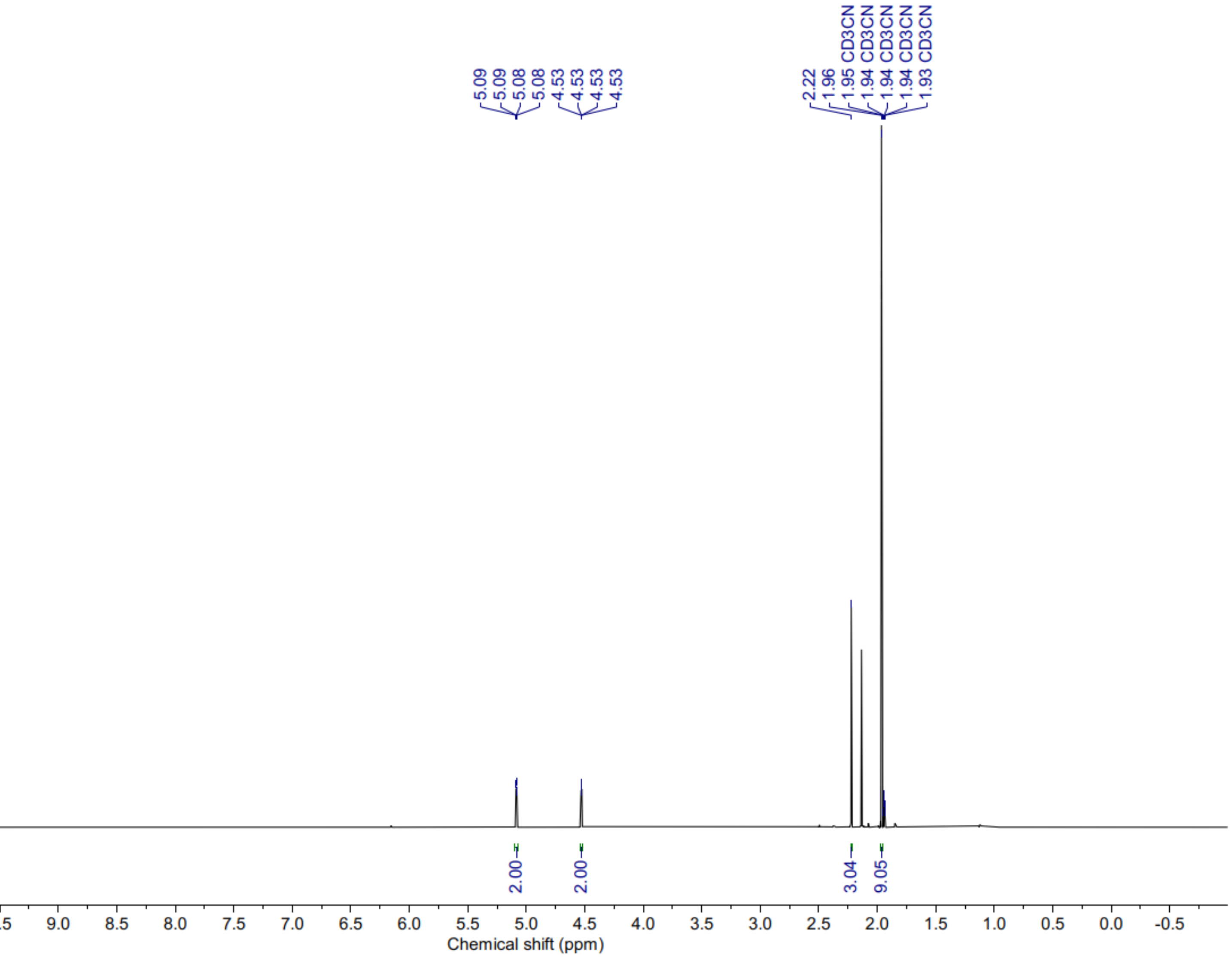

^{13}C NMR spectrum of 1

151 MHz, CD_3CN, 298 K

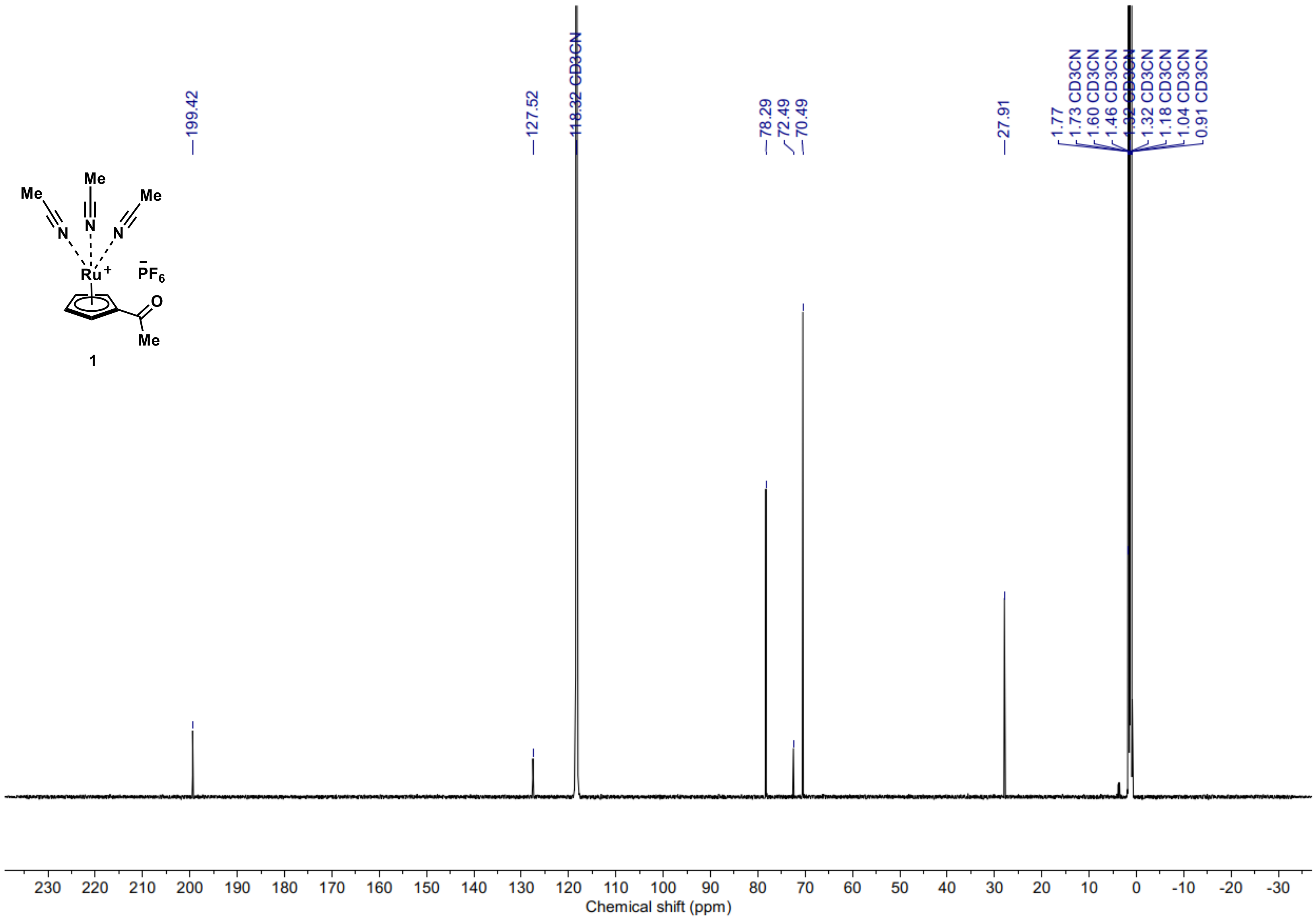

^{19}F NMR spectrum of 1

565 MHz, CD_3CN, 298 K

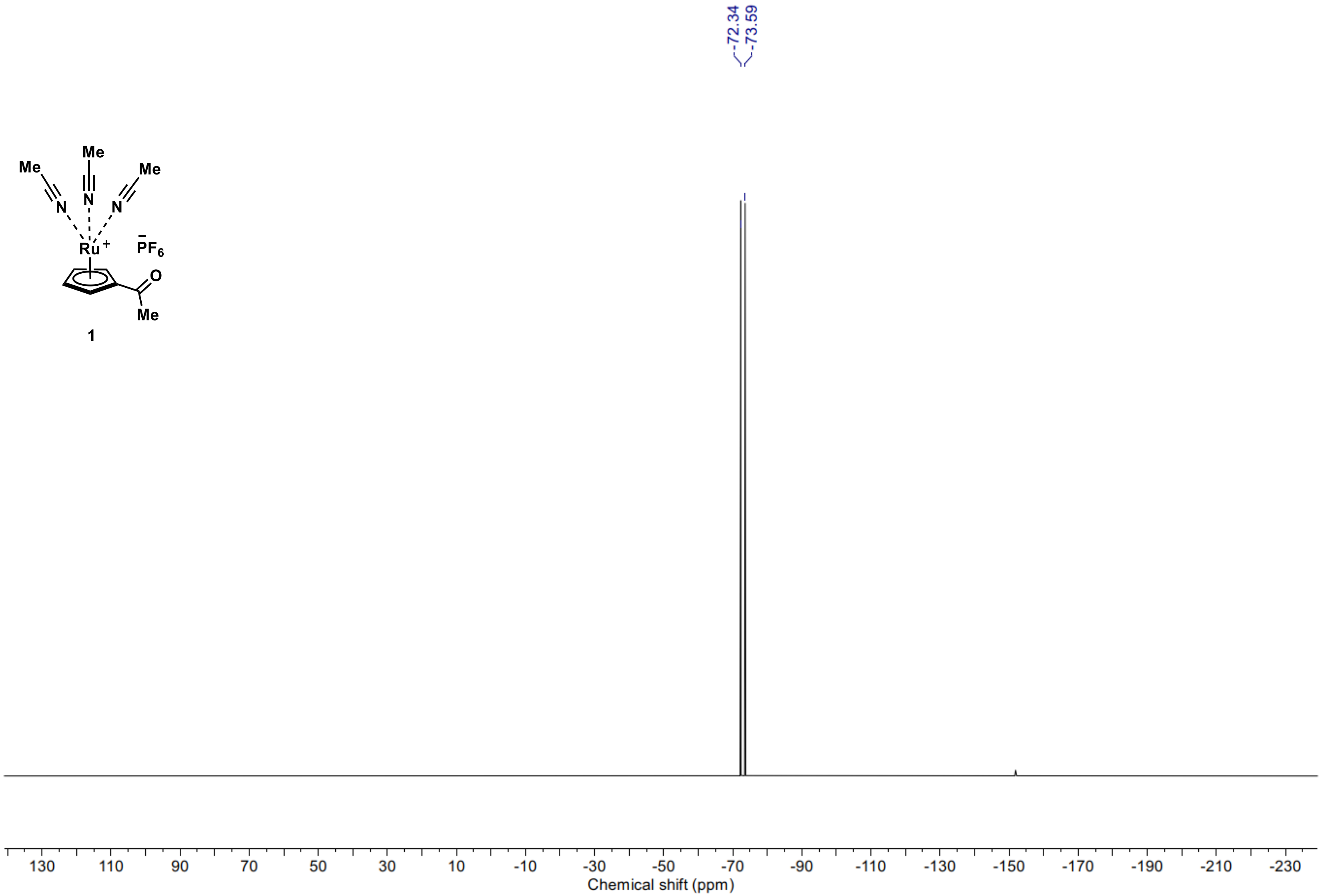

^{31}P NMR spectrum of 1

243 MHz, CD_3CN, 298 K

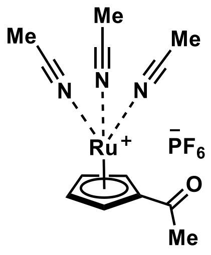

1

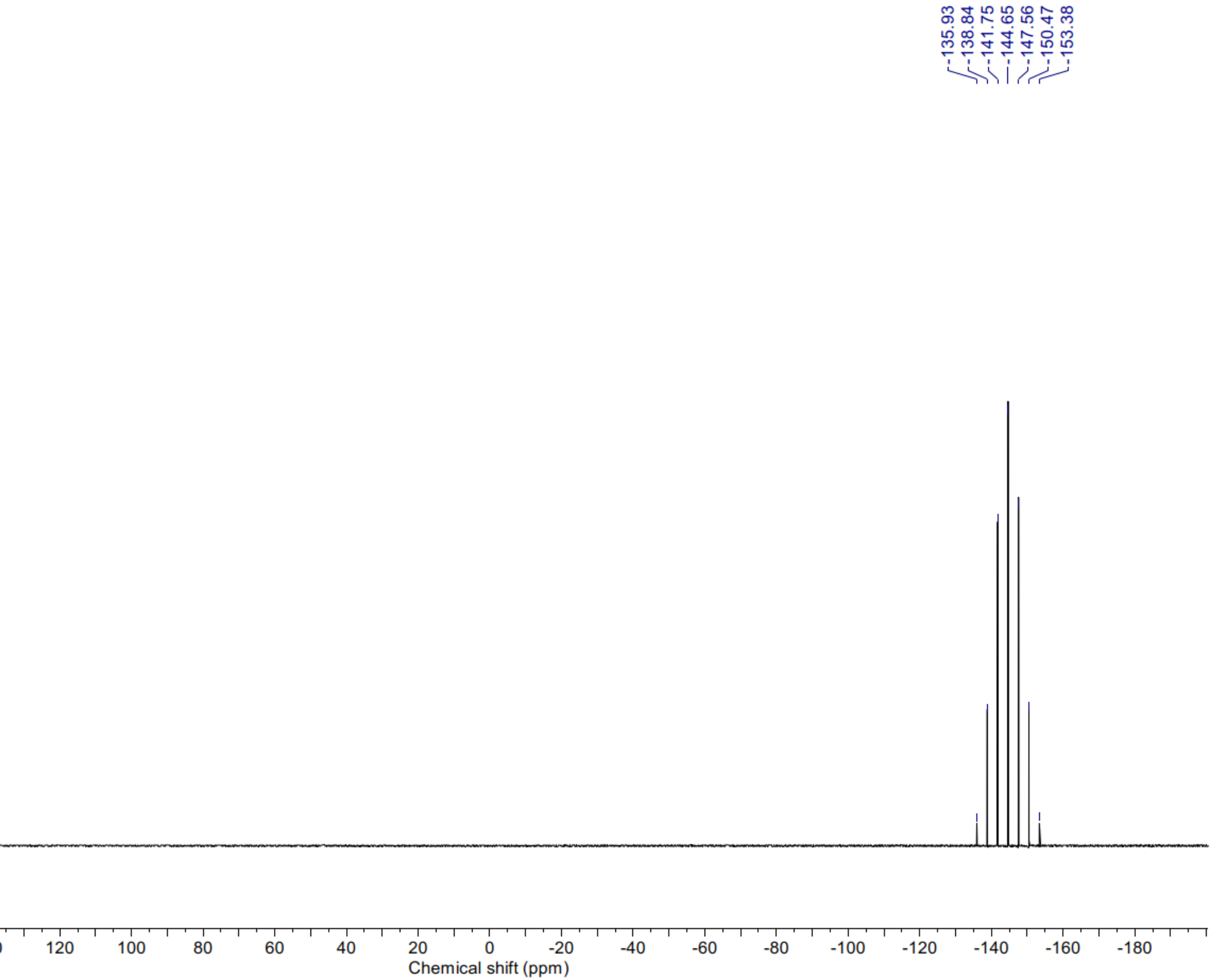

^{1}H NMR spectrum of 5

600 MHz, CD_3CN, 298 K

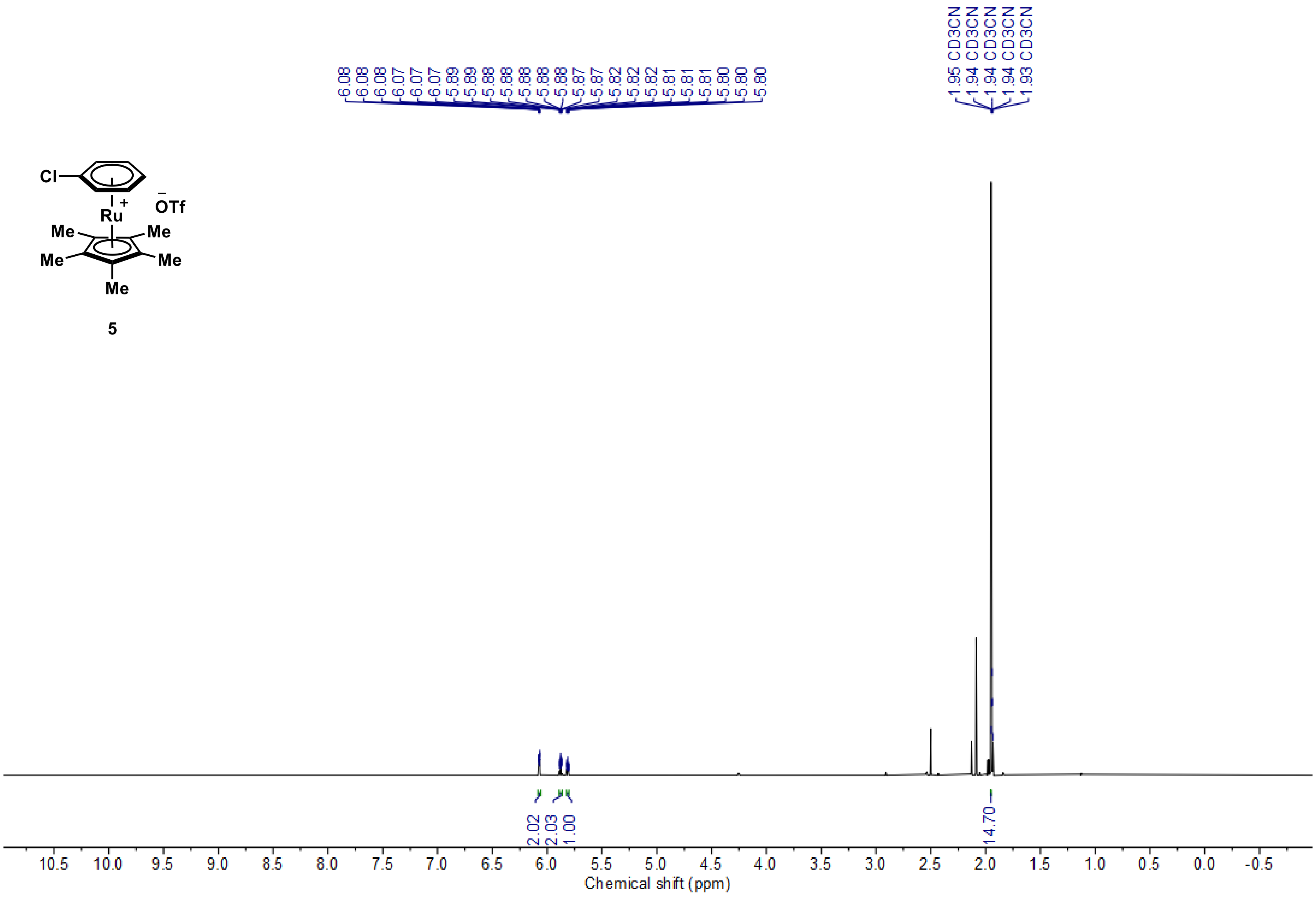

^{13}C NMR spectrum of 5

151 MHz, CD_3CN, 298 K

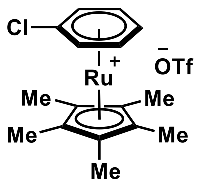

5

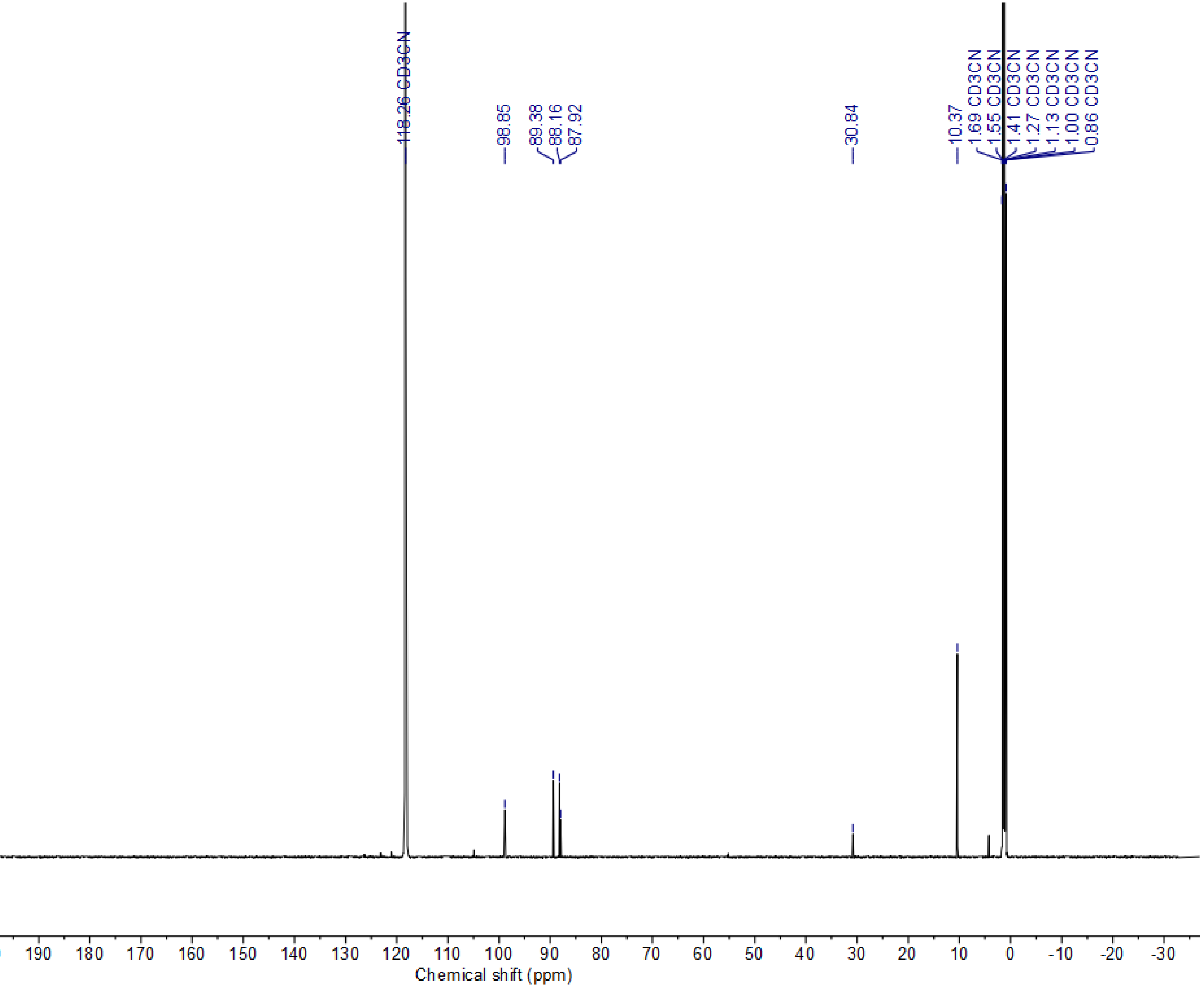

^{19}F NMR spectrum of 5

565 MHz, CD_3CN, 298 K

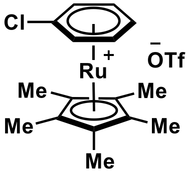

5

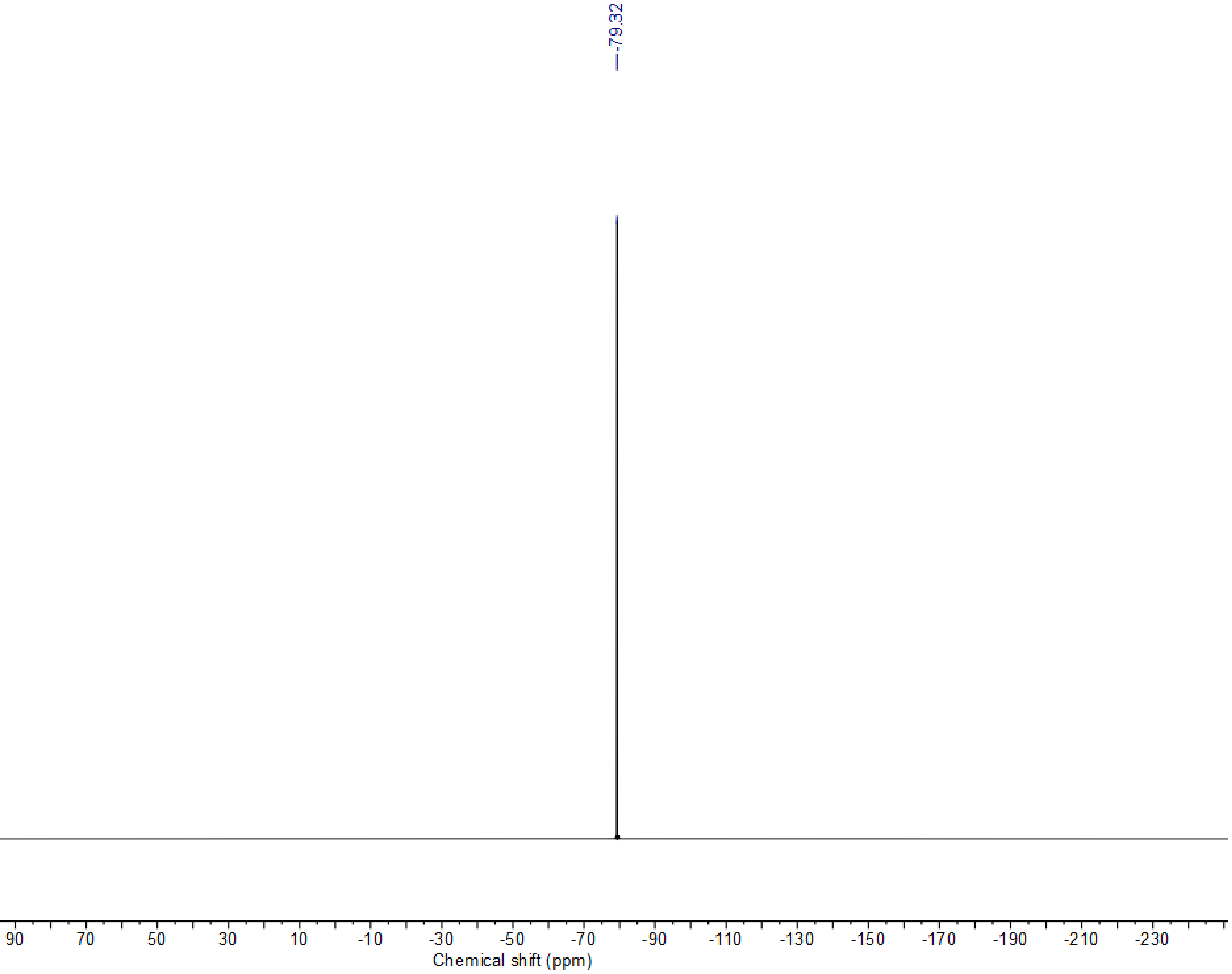

^{1}H NMR spectrum of 6

600 MHz, CD_3CN, 298 K

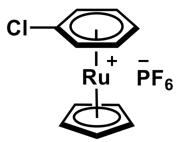

6

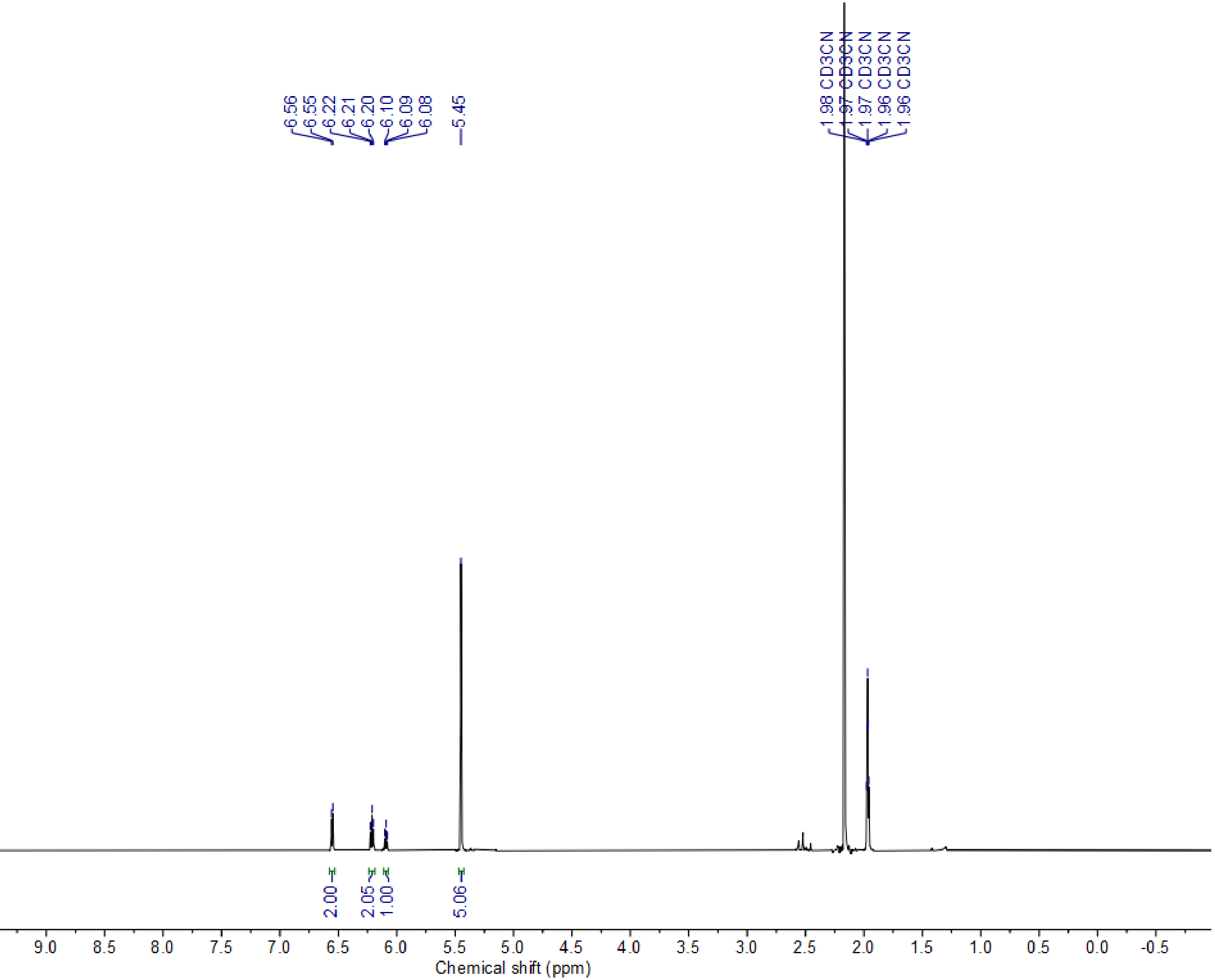

^{13}C NMR spectrum of 6

151 MHz, CD_3CN, 298 K

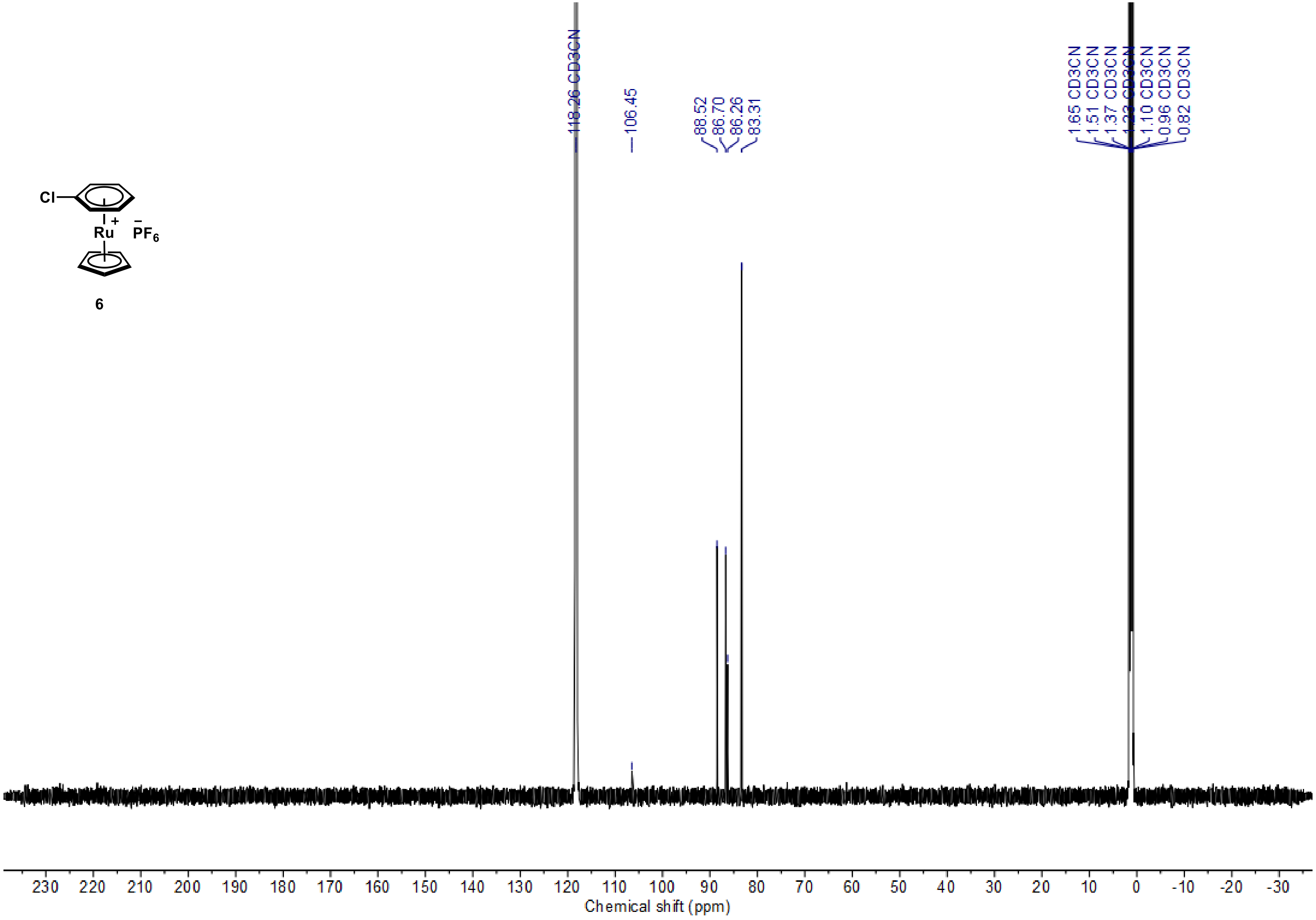

^{19}F NMR spectrum of 6

565 MHz, CD_3CN, 298 K

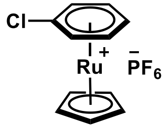

6

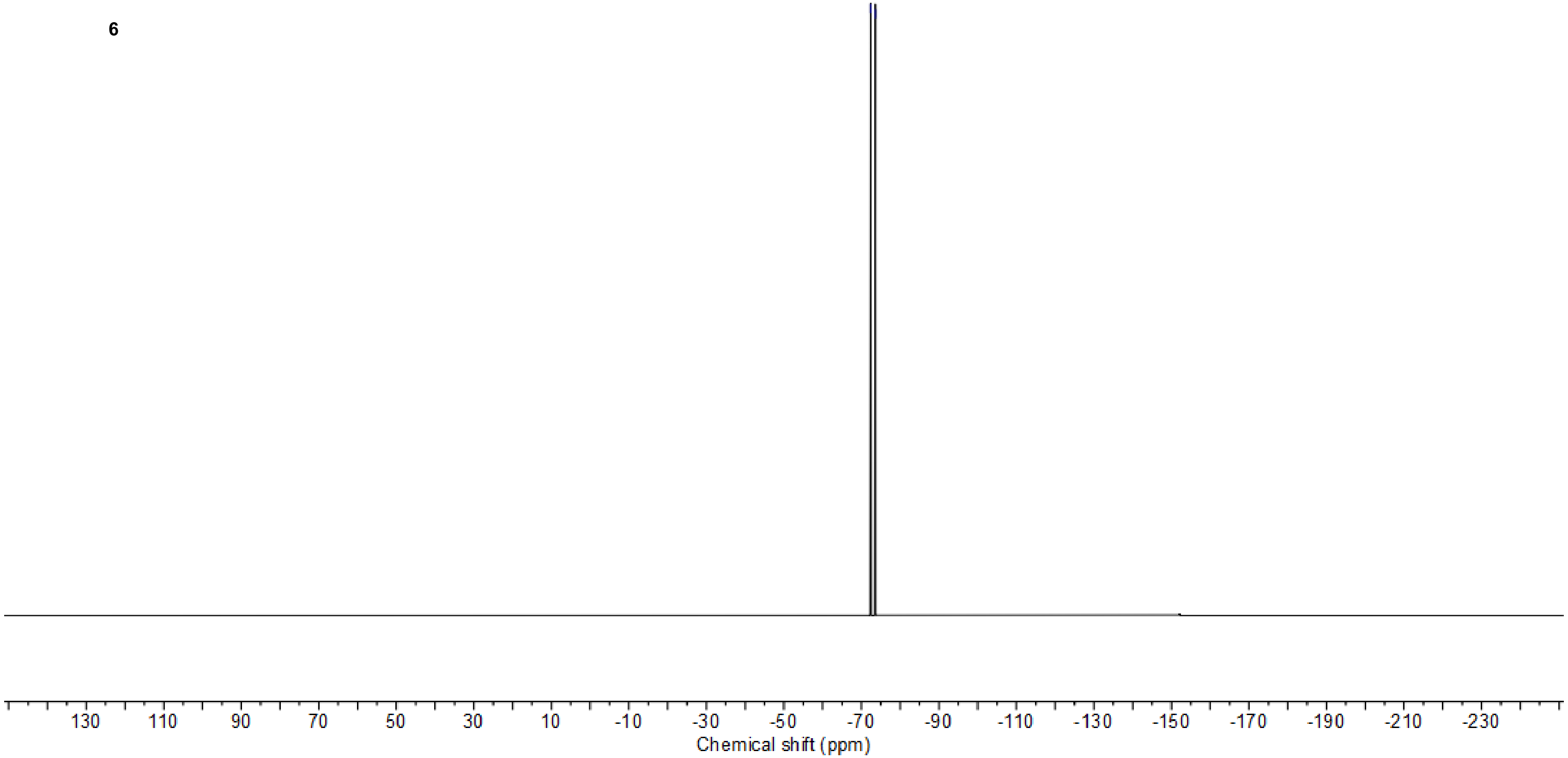

^{31}P NMR spectrum of 6

243 MHz, CD_3CN, 298 K

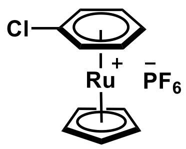

6

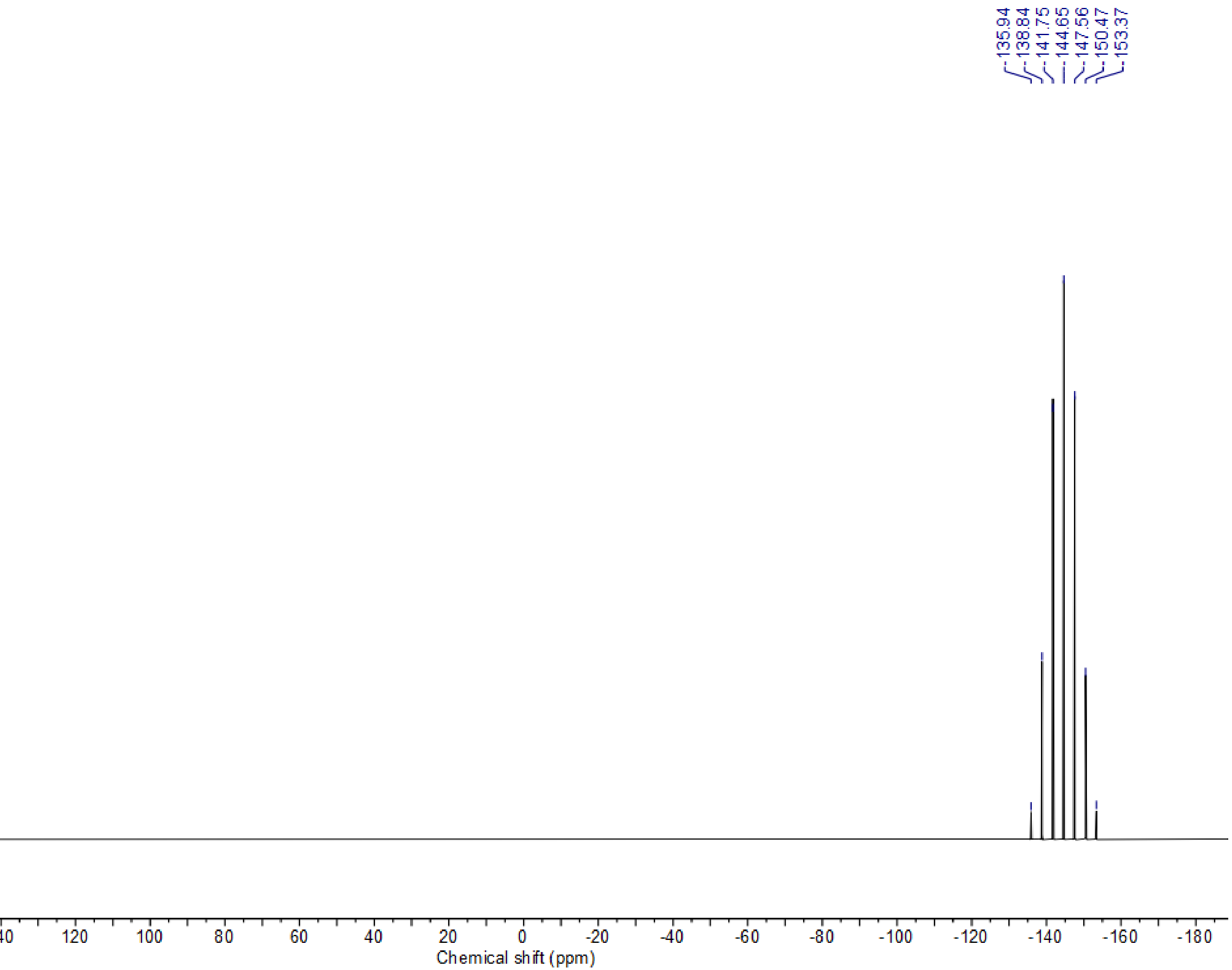

^{1}H NMR spectrum of 7

600 MHz, CD_3CN, 298 K

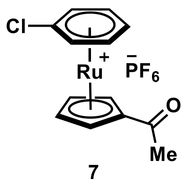

7

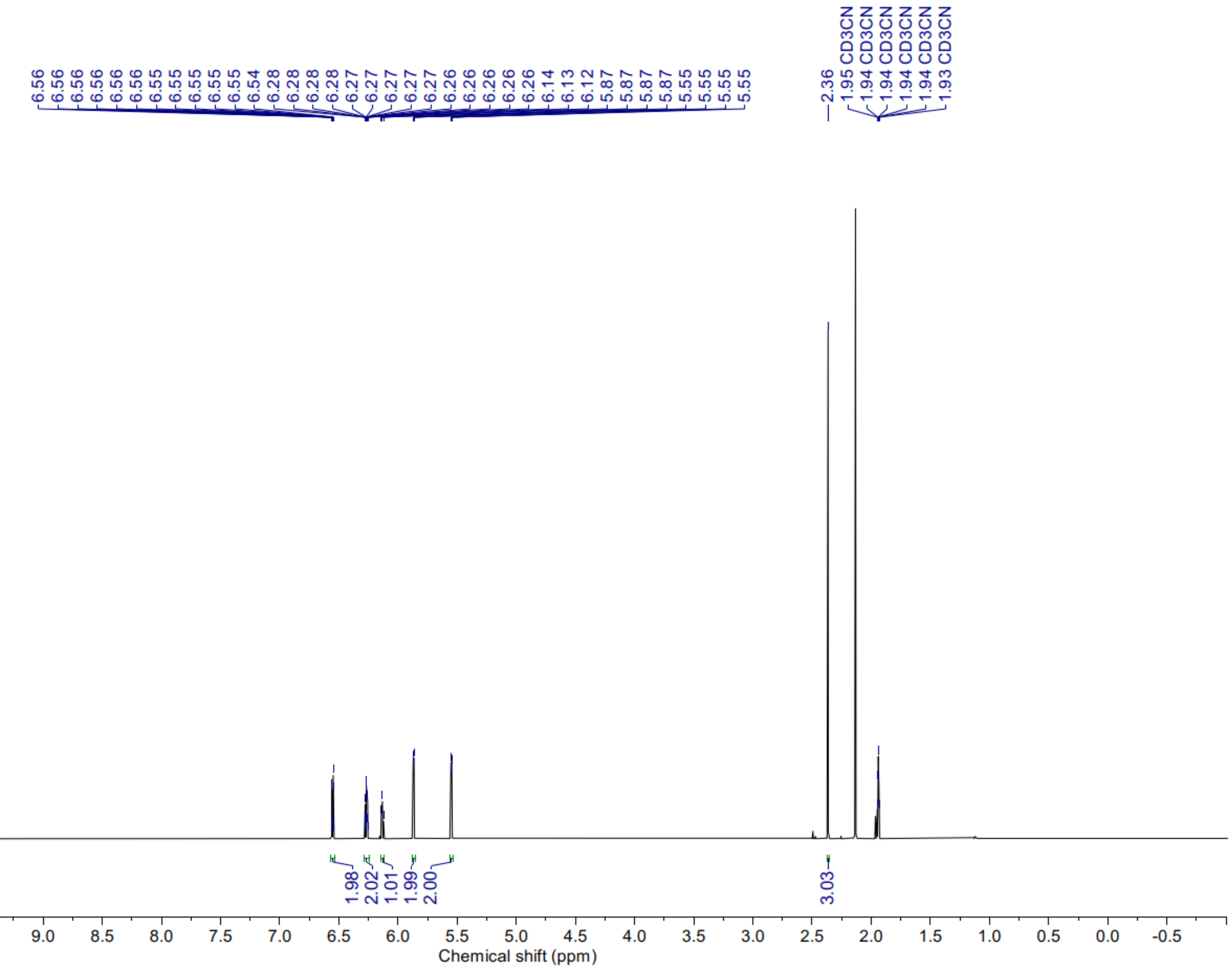

^{13}C NMR spectrum of 7

151 MHz, CD_3CN, 298 K

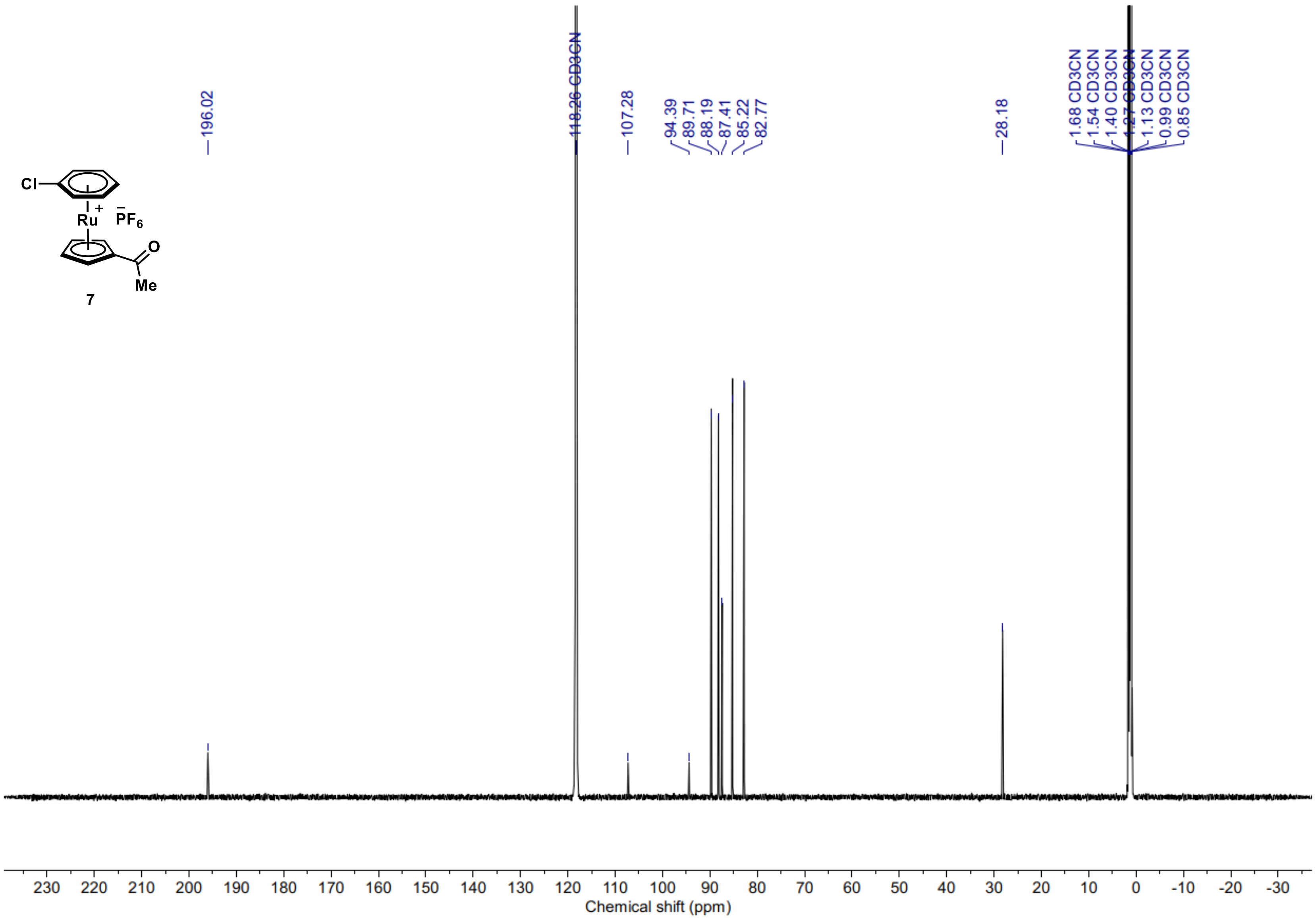

^{19}F NMR spectrum of 7

565 MHz, CD_3CN, 298 K

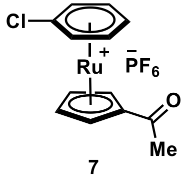

7

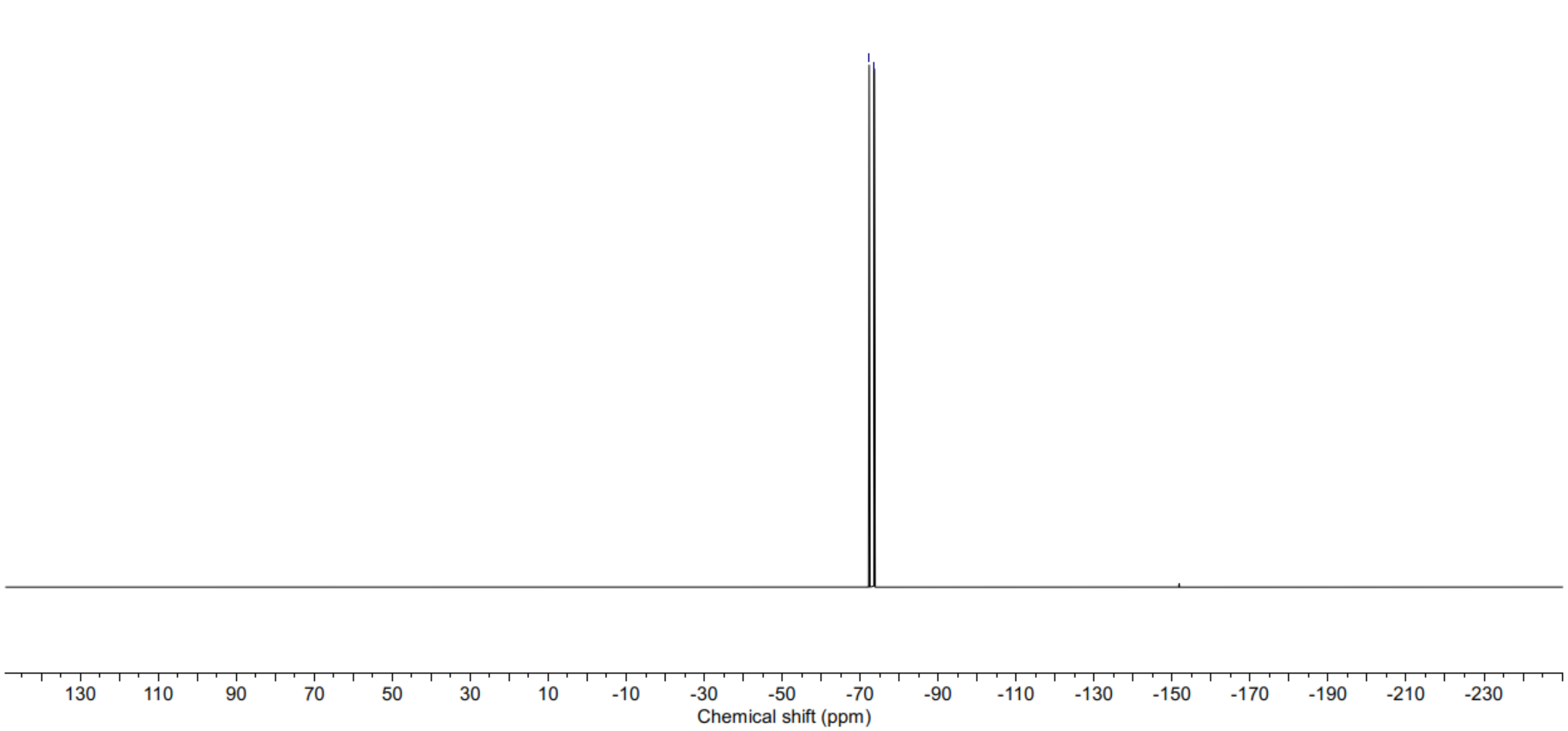

^{31}P NMR spectrum of 7

243 MHz, CD_3CN, 298 K

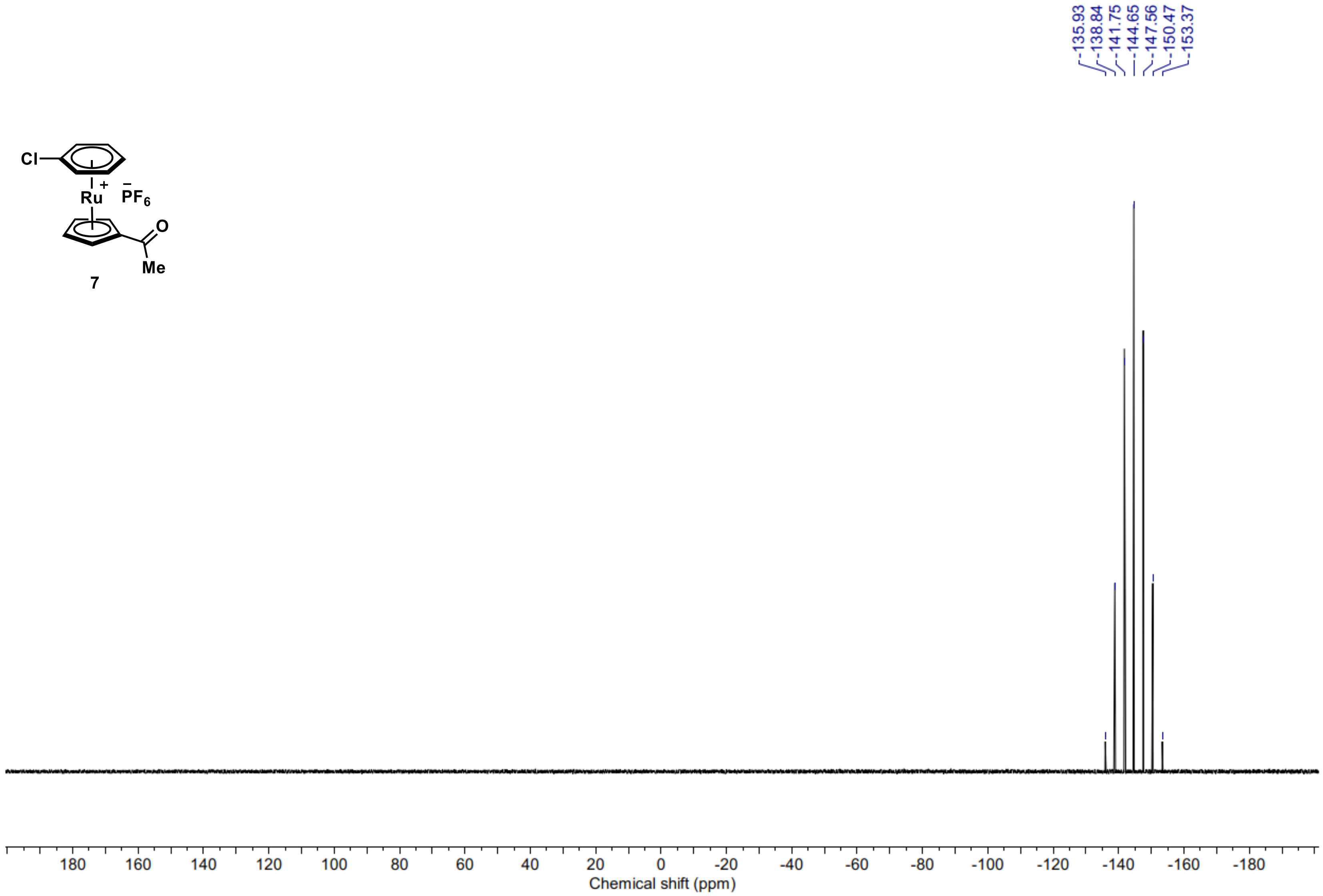

^{1}H NMR spectrum of S7

600 MHz, CD_3CN, 298 K

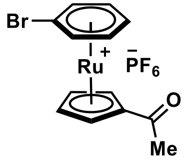

S7

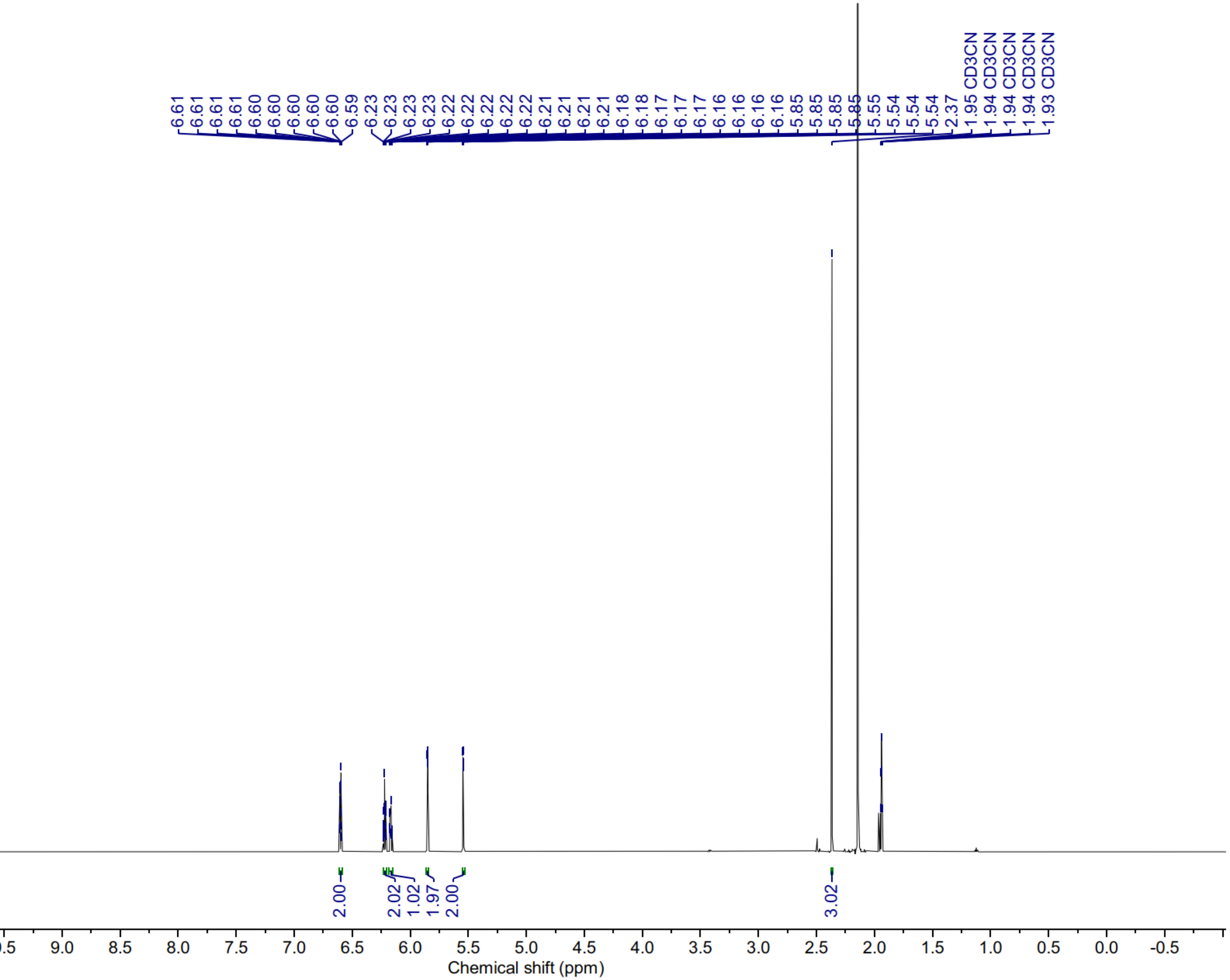

^{13}C NMR spectrum of S7

151 MHz, CD_3CN, 298 K

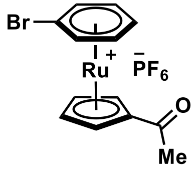

S7

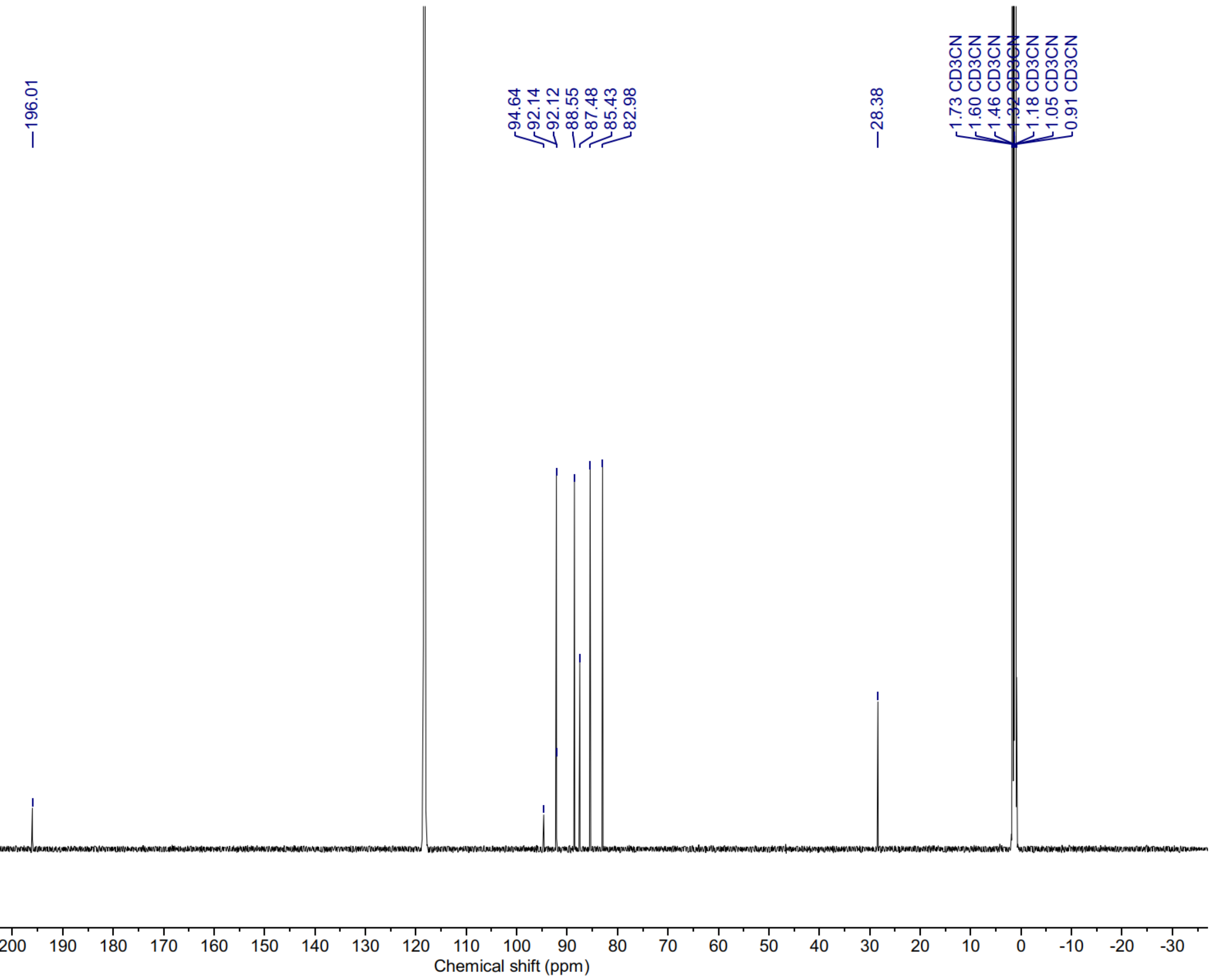

^{19}F NMR spectrum of S7

565 MHz, CD_3CN, 298 K

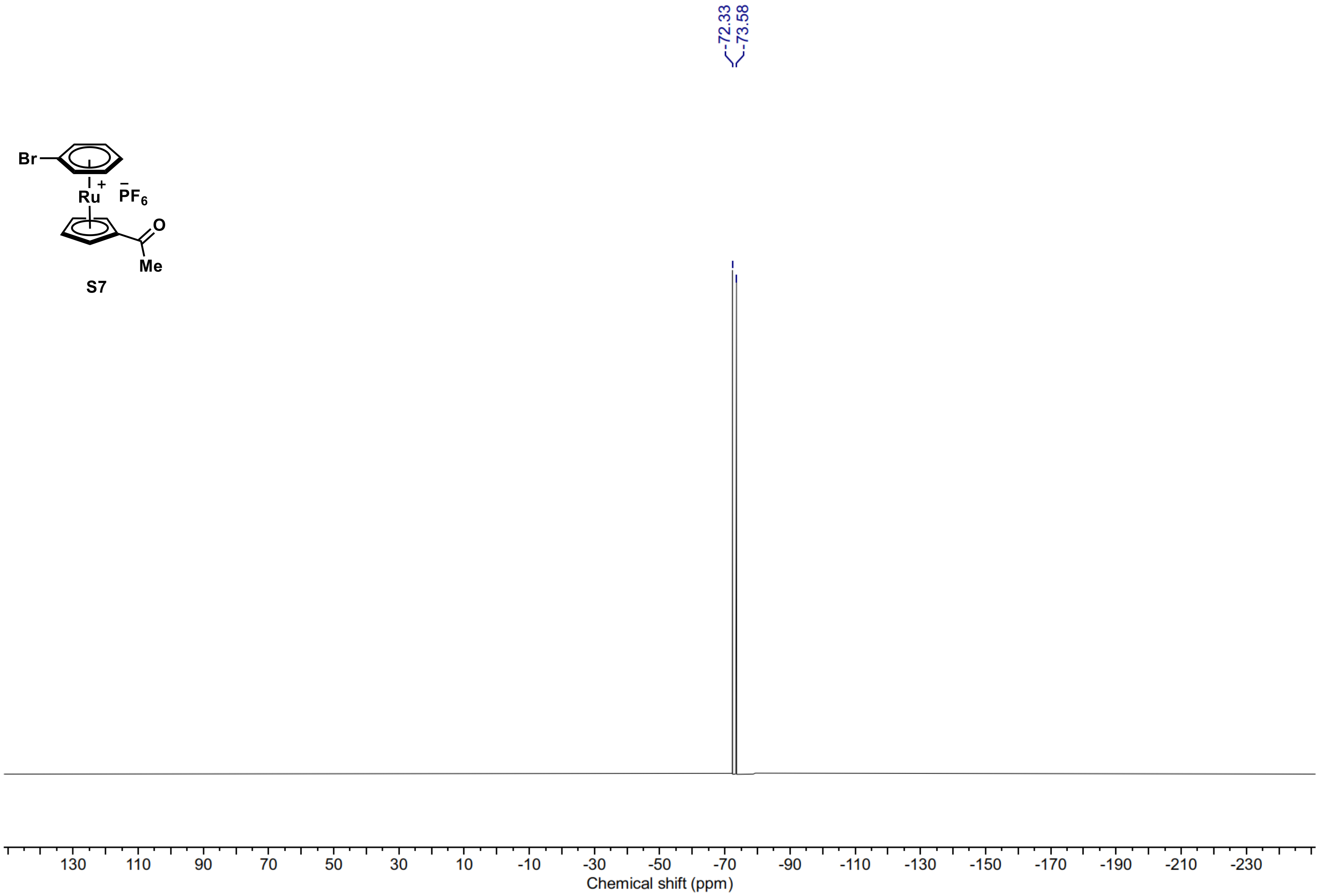

^{31}P NMR spectrum of S7

243 MHz, CD_3CN, 298 K

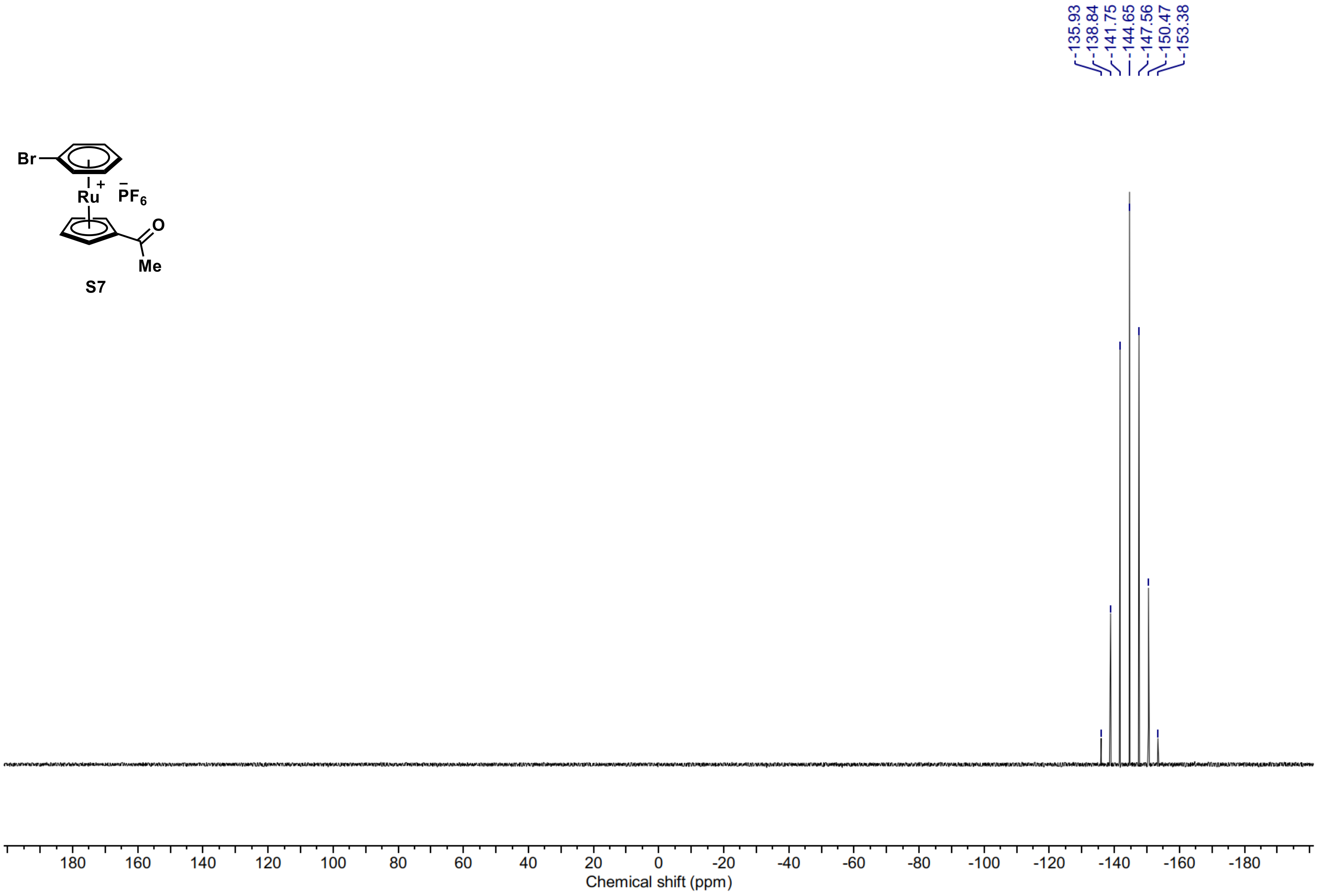

S7

^{1}H NMR spectrum of S8

600 MHz, CD_3CN, 298 K

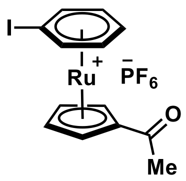

S8

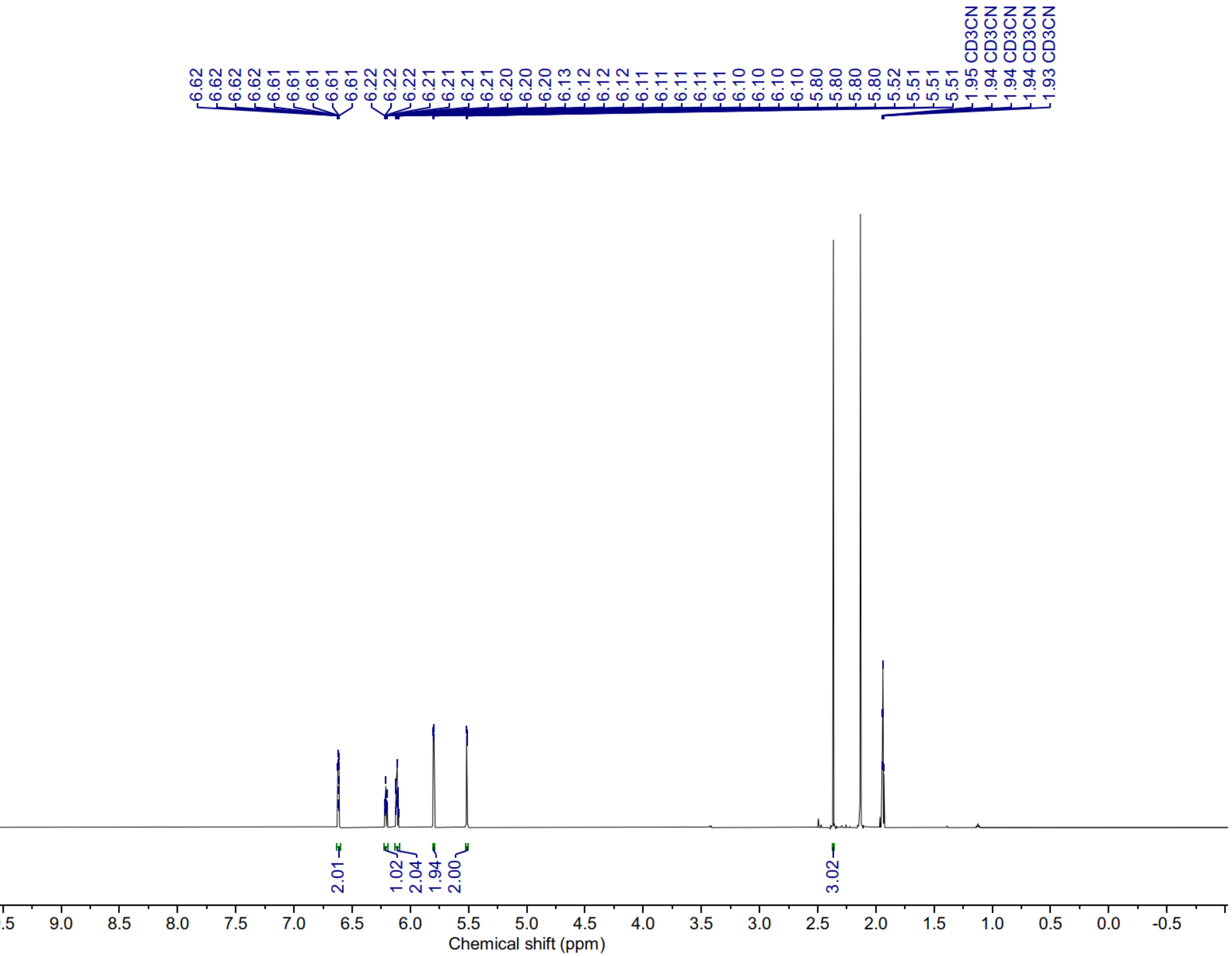

^{13}C NMR spectrum of S8

151 MHz, CD_3CN, 298 K

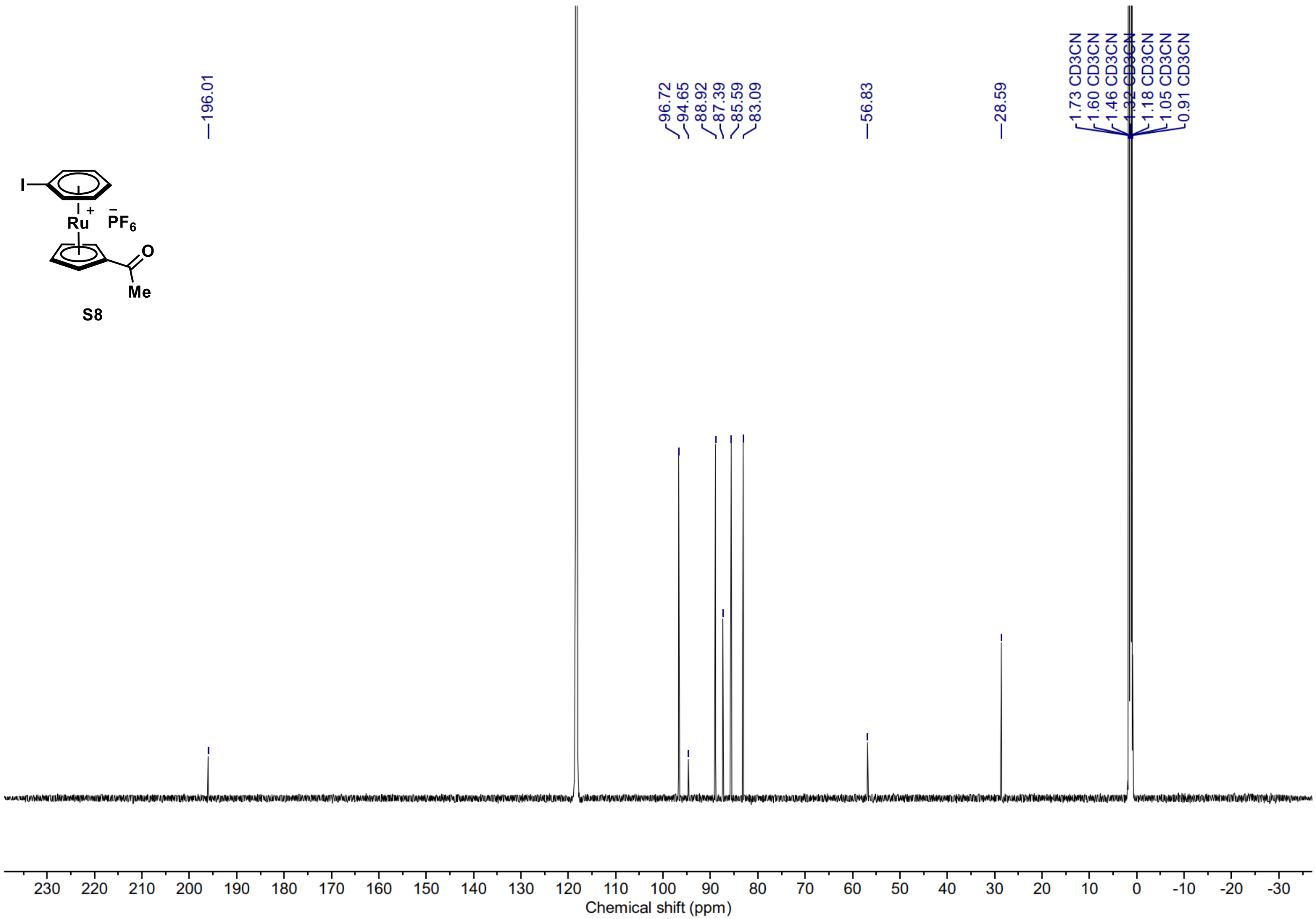

^{19}F NMR spectrum of S8

565 MHz, CD_3CN, 298 K

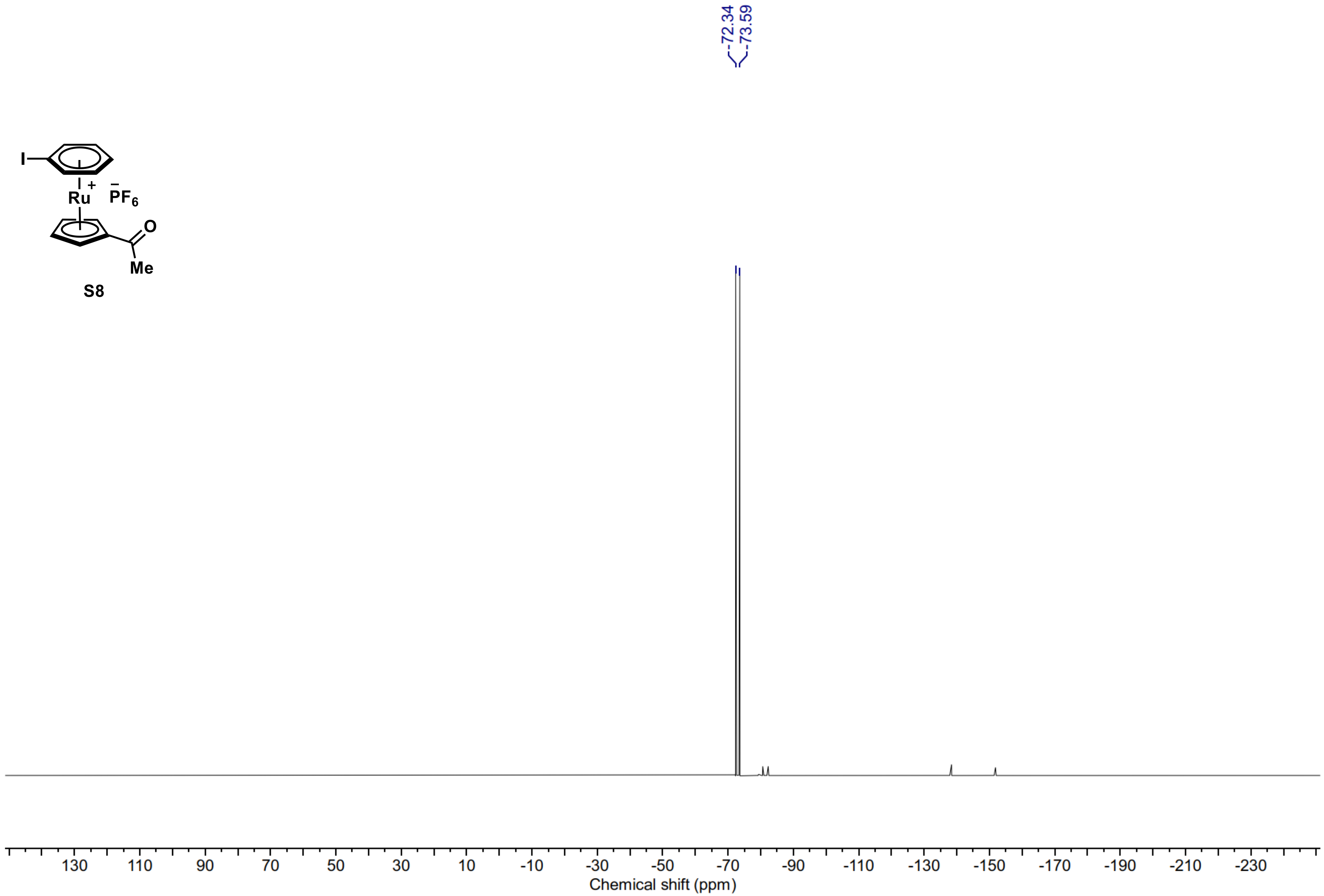

^{31}P NMR spectrum of S8

243 MHz, CD_3CN, 298 K

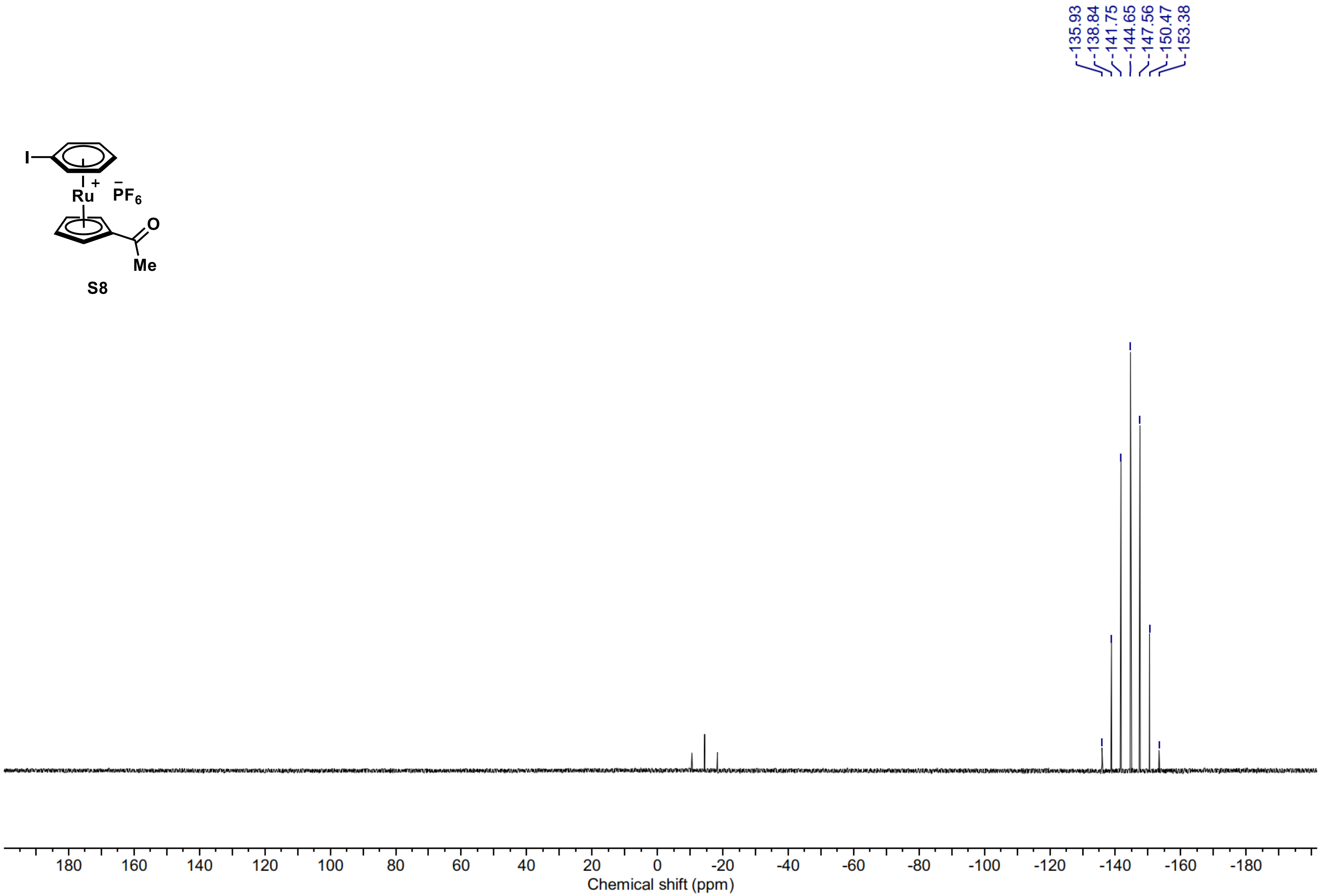

^{1}H NMR spectrum of S9

600 MHz, CD_3CN, 298 K

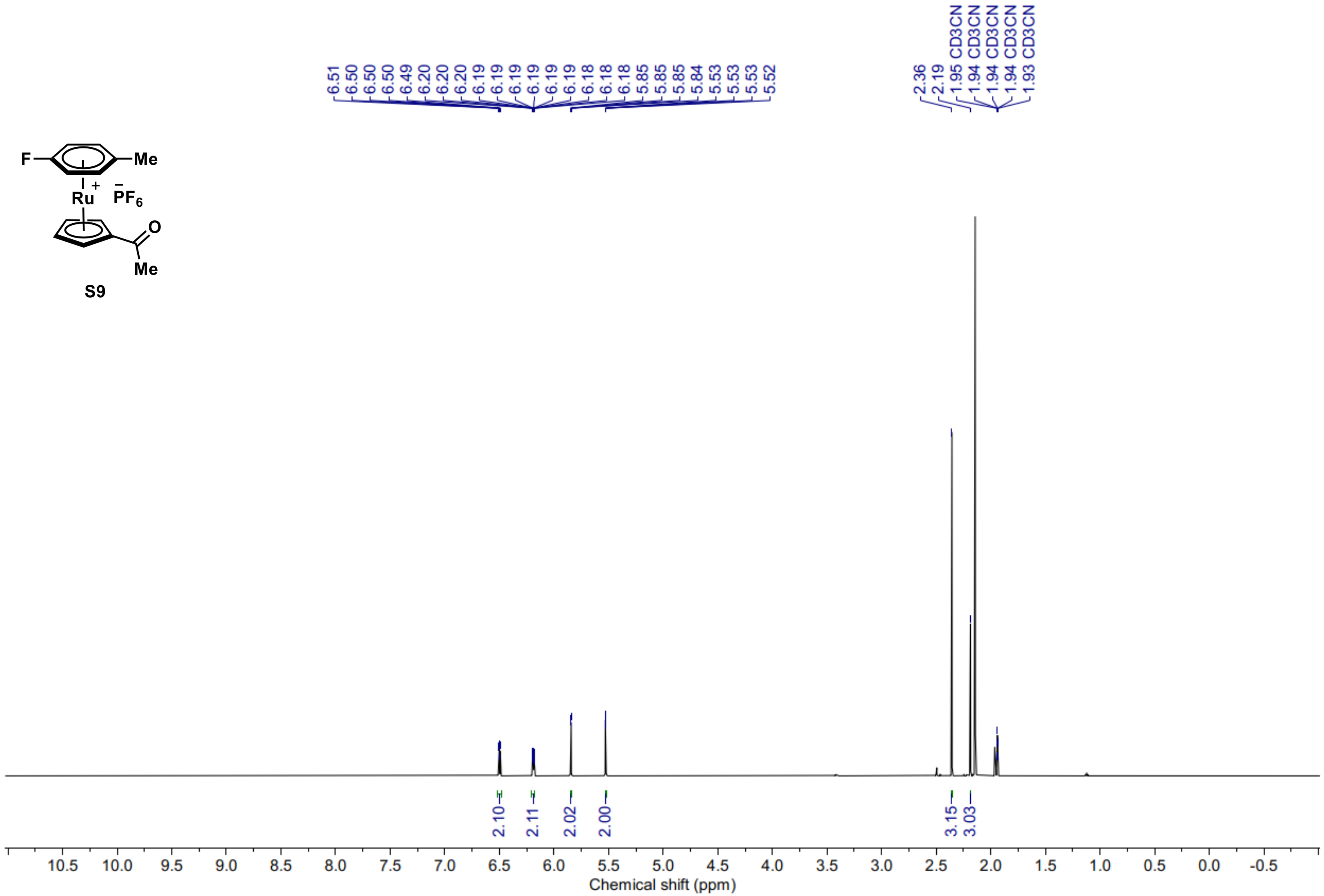

S9

^{13}C NMR spectrum of S9

151 MHz, CD_3CN, 298 K

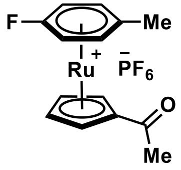

S9

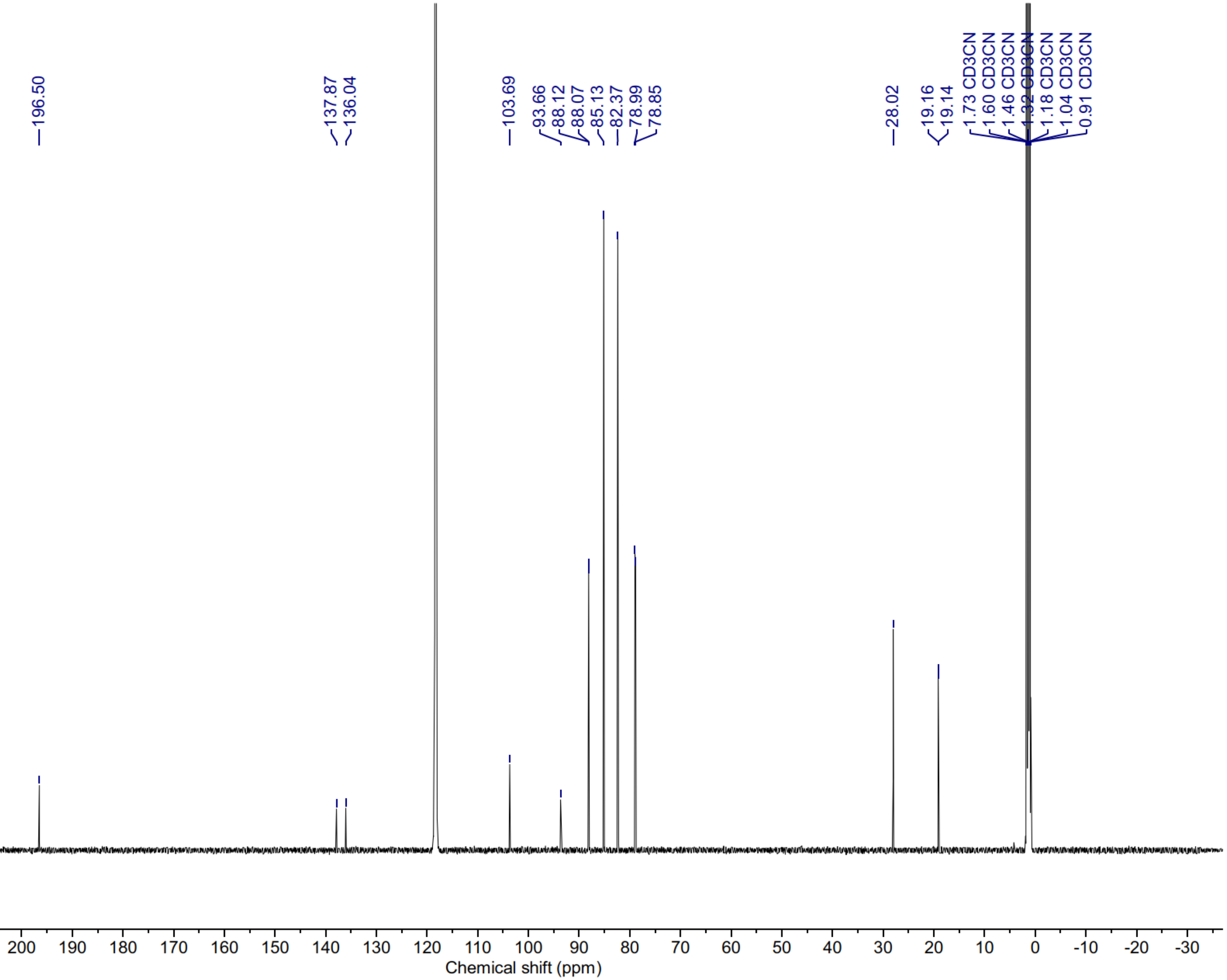

^{19}F NMR spectrum of S9

565 MHz, CD_3CN, 298 K

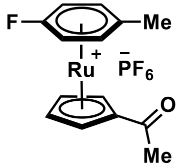

S9

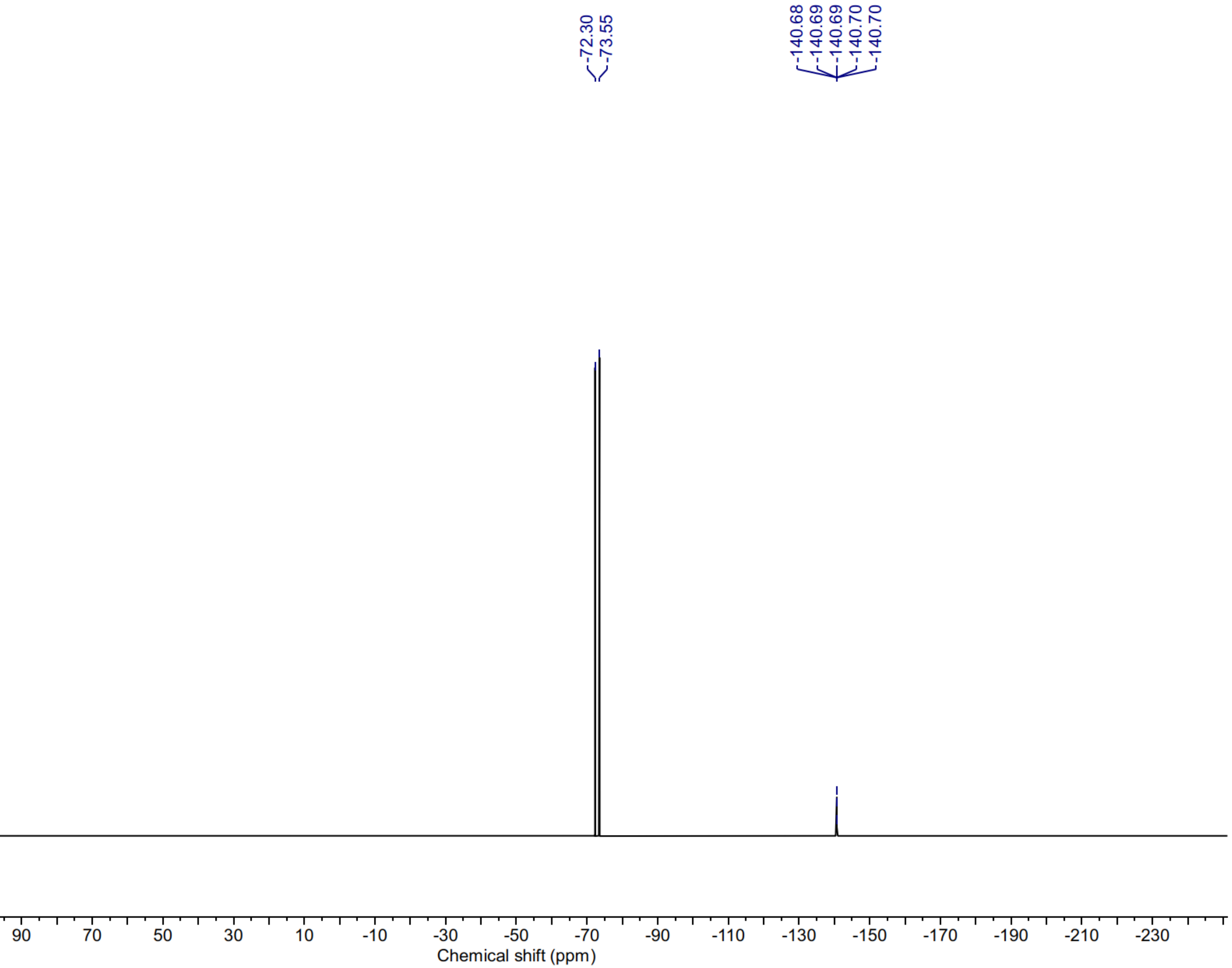

^{31}P NMR spectrum of S9

243 MHz, CD_3CN, 298 K

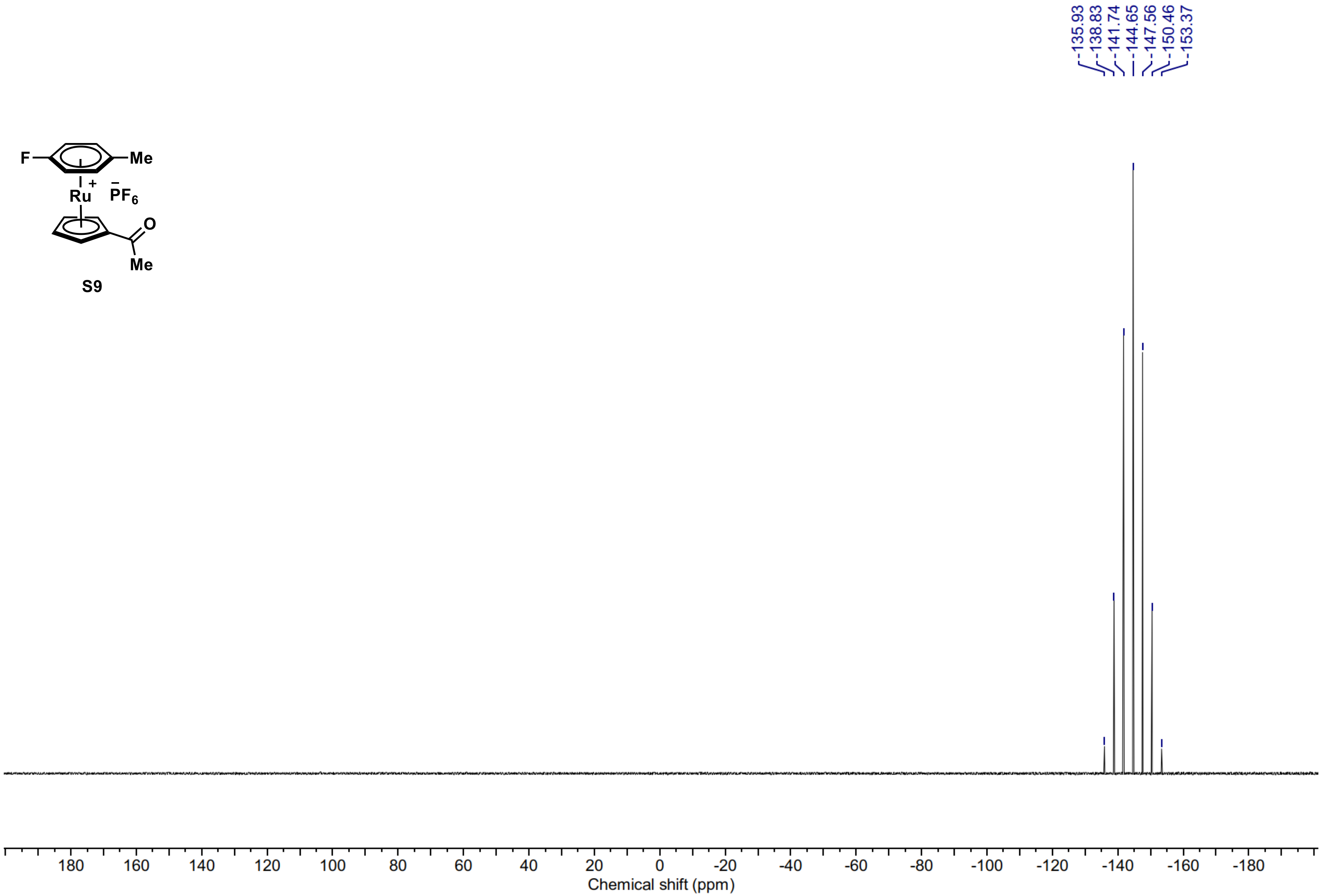

^{1}H NMR spectrum of S10

600 MHz, CD_3CN, 298 K

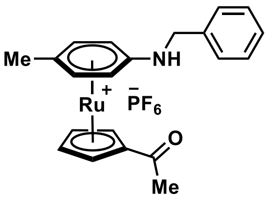

S10

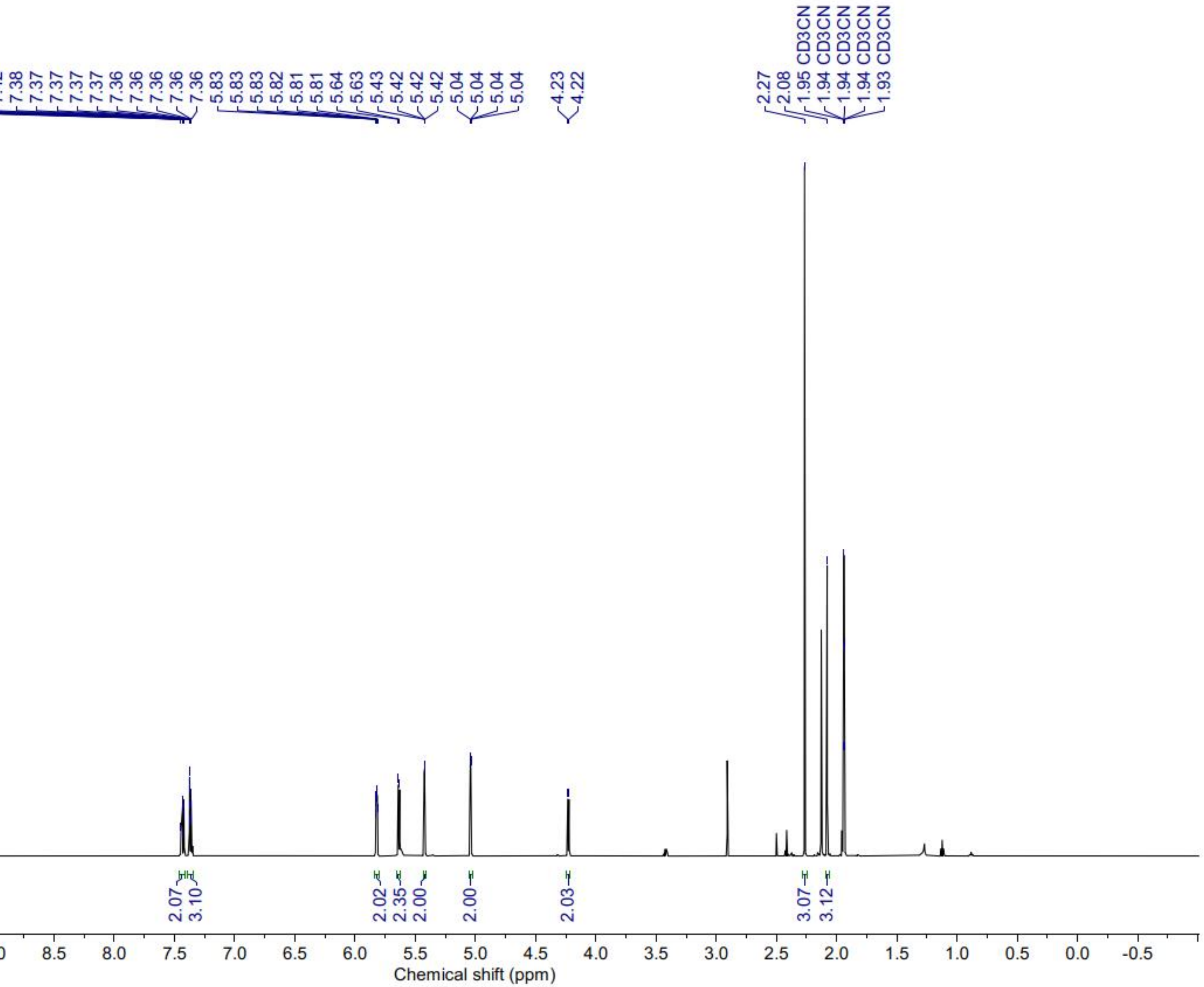

^{13}C NMR spectrum of S10

151 MHz, CD_3CN, 298 K

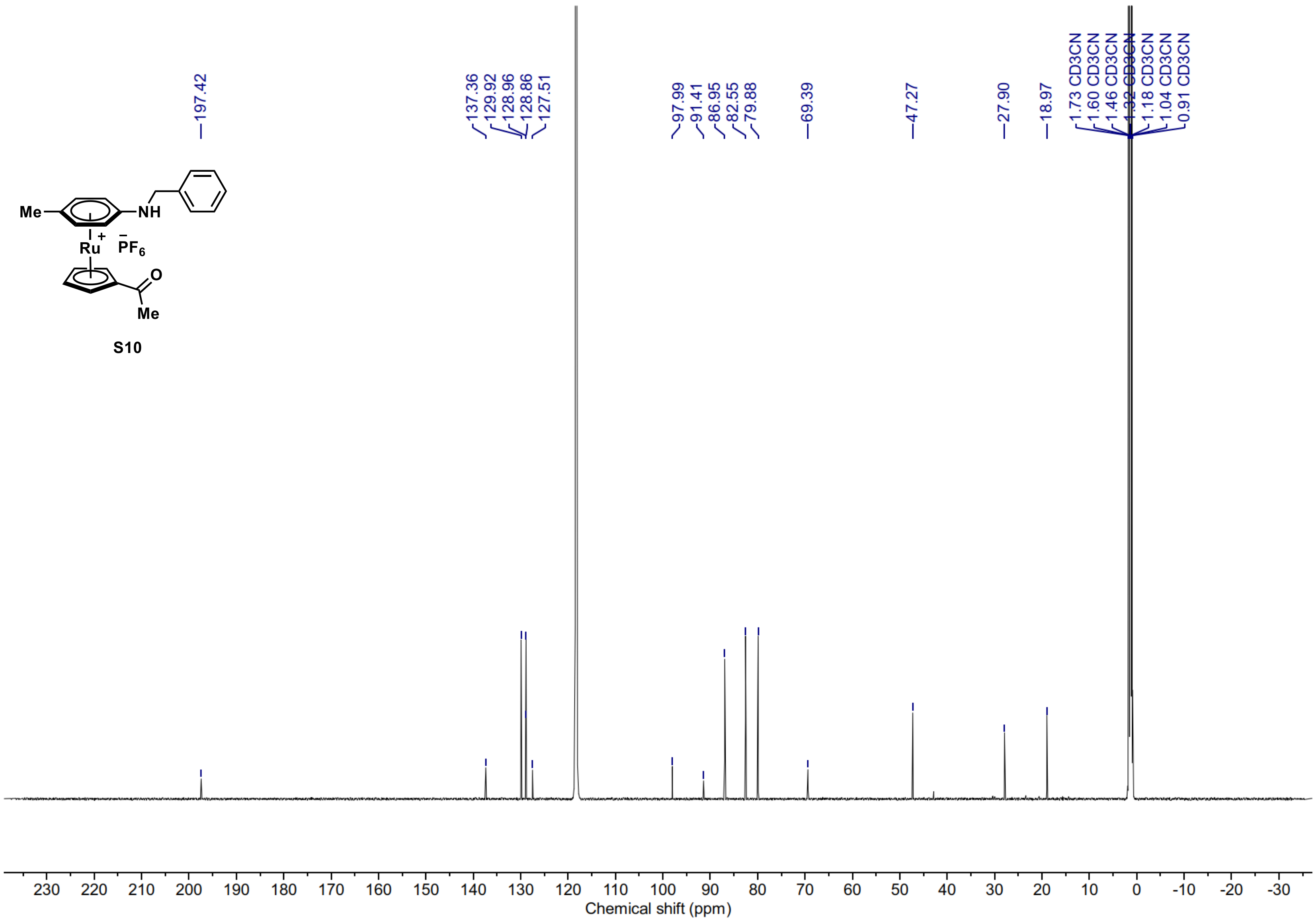

^{19}F NMR spectrum of S10

471 MHz, CD_3CN, 298 K

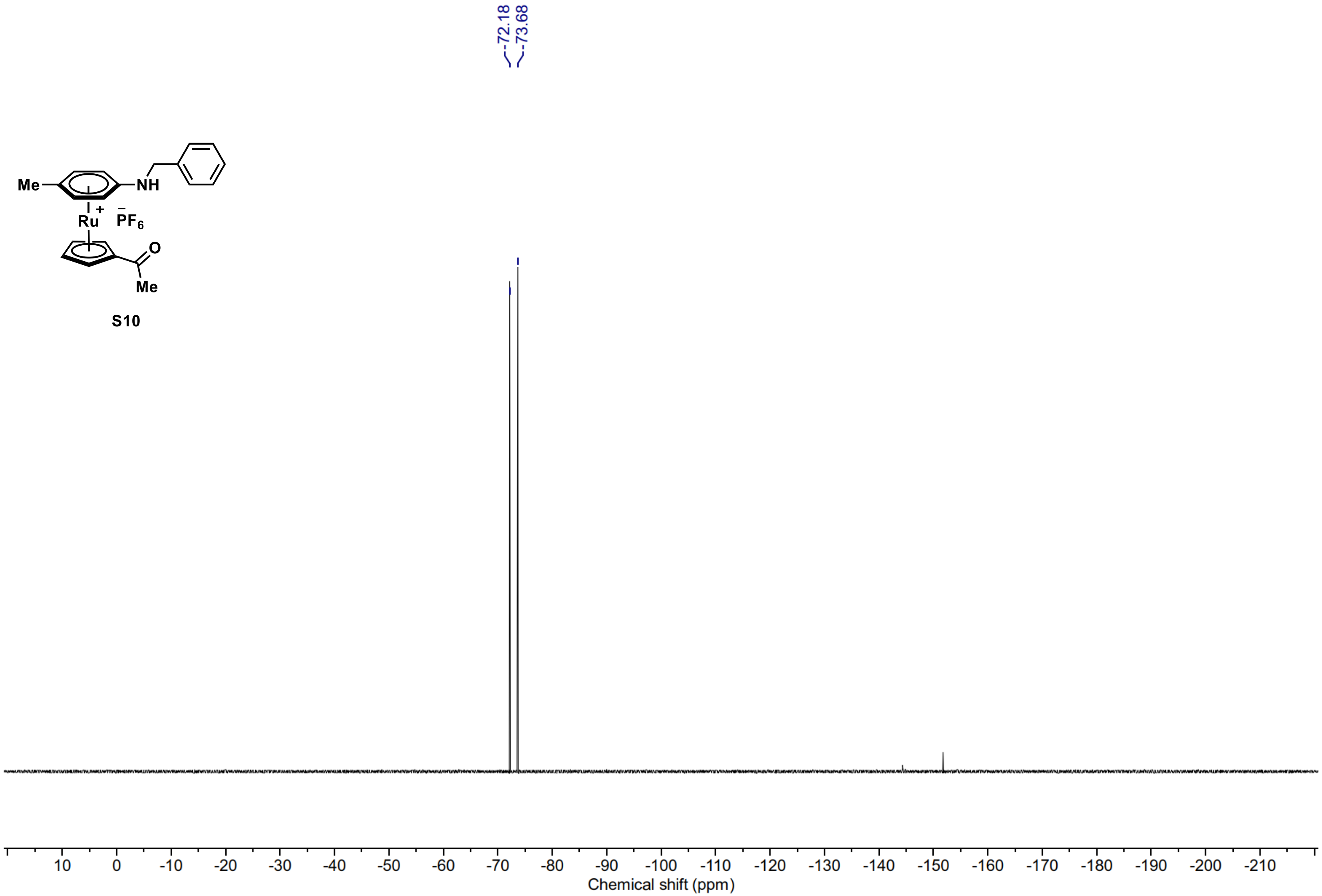

f

^{31}P NMR spectrum of S10

243 MHz, CD_3CN, 298 K

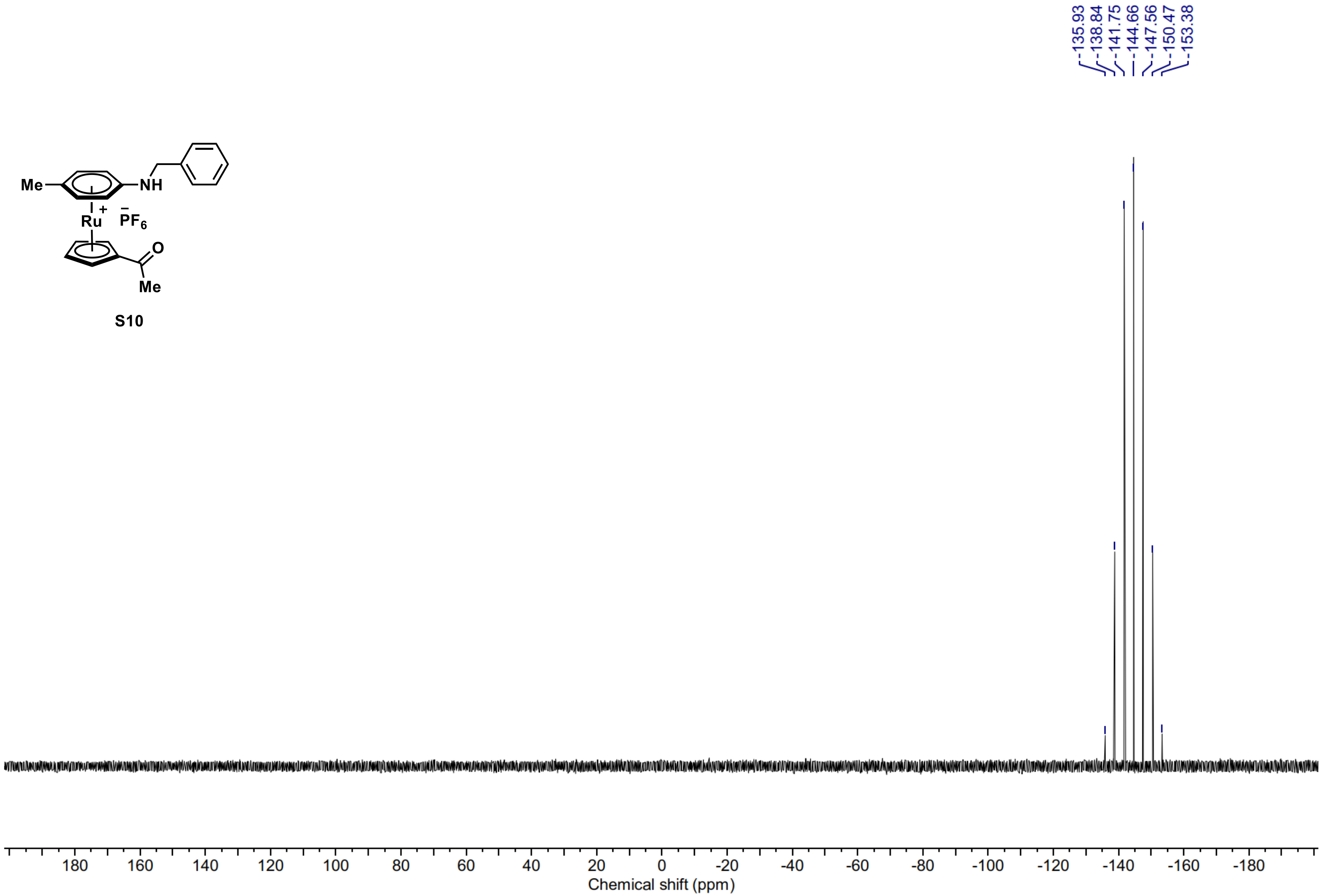

^{1}H NMR spectrum of S14

600 MHz, CD_3CN, 298 K

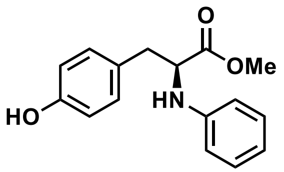

S14

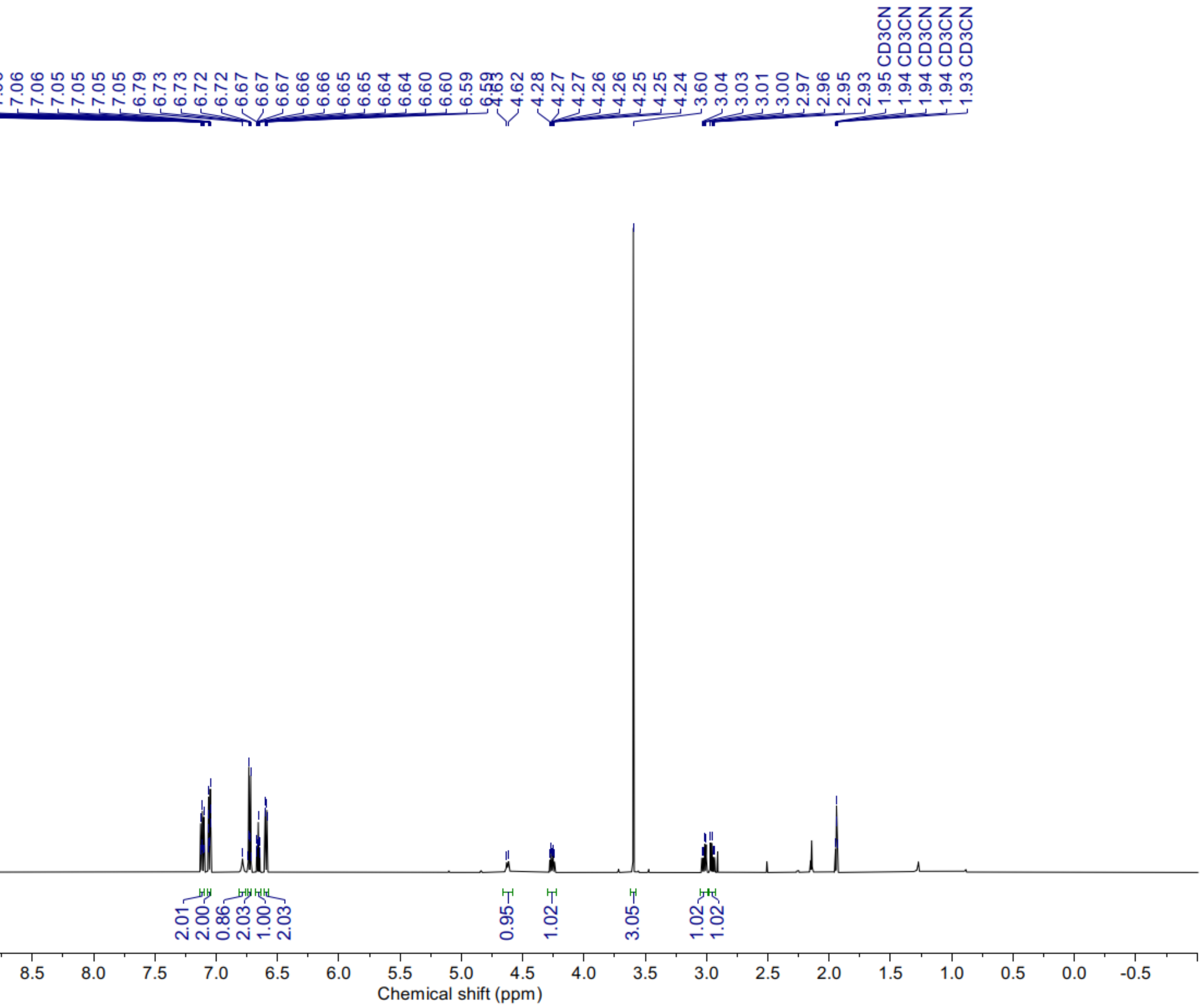

^{13}C NMR spectrum of S14

151 MHz, CD_3CN, 298 K

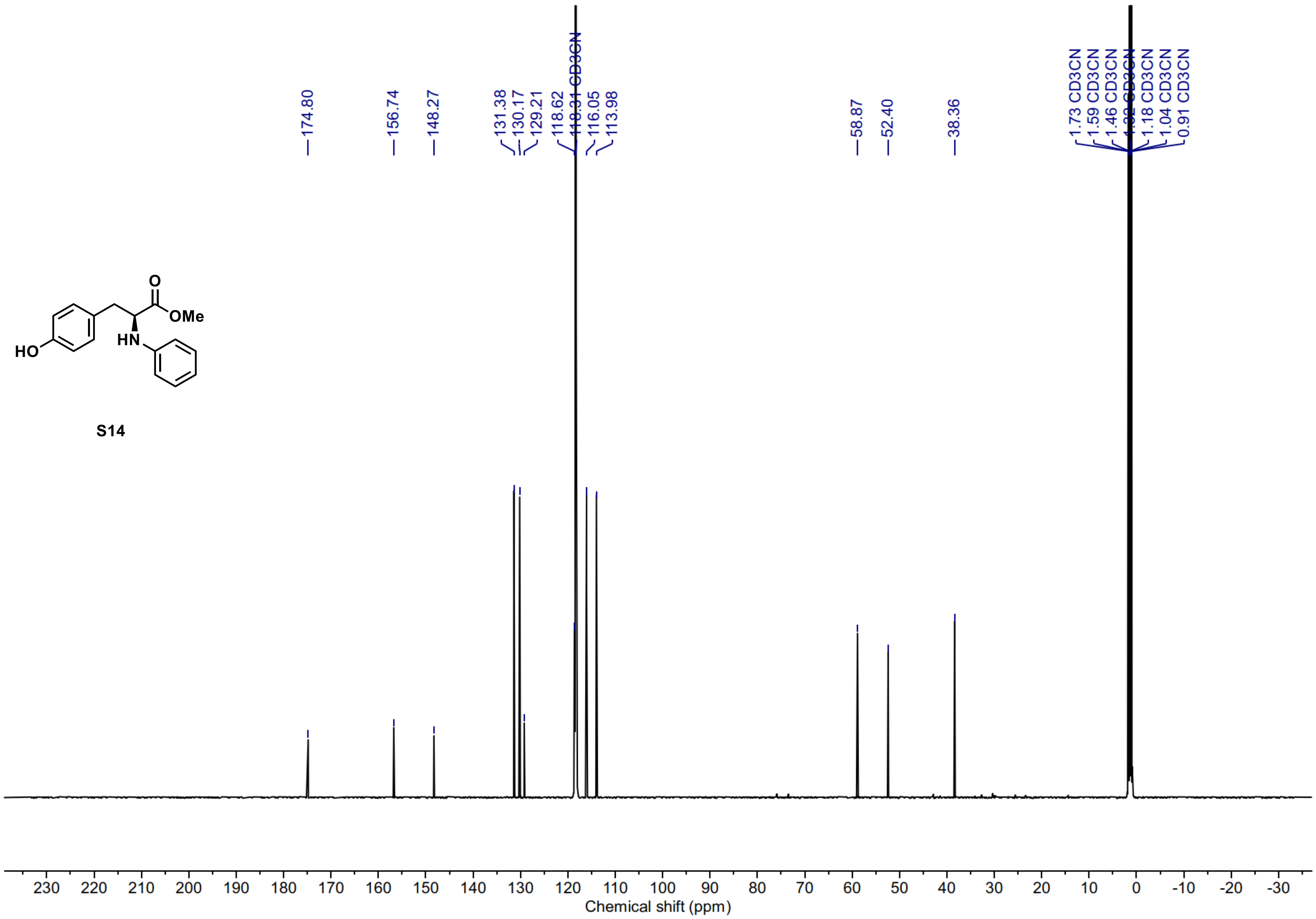

^{1}H NOESY NMR spectrum of S14

600 MHz, CD_3CN, 298 K

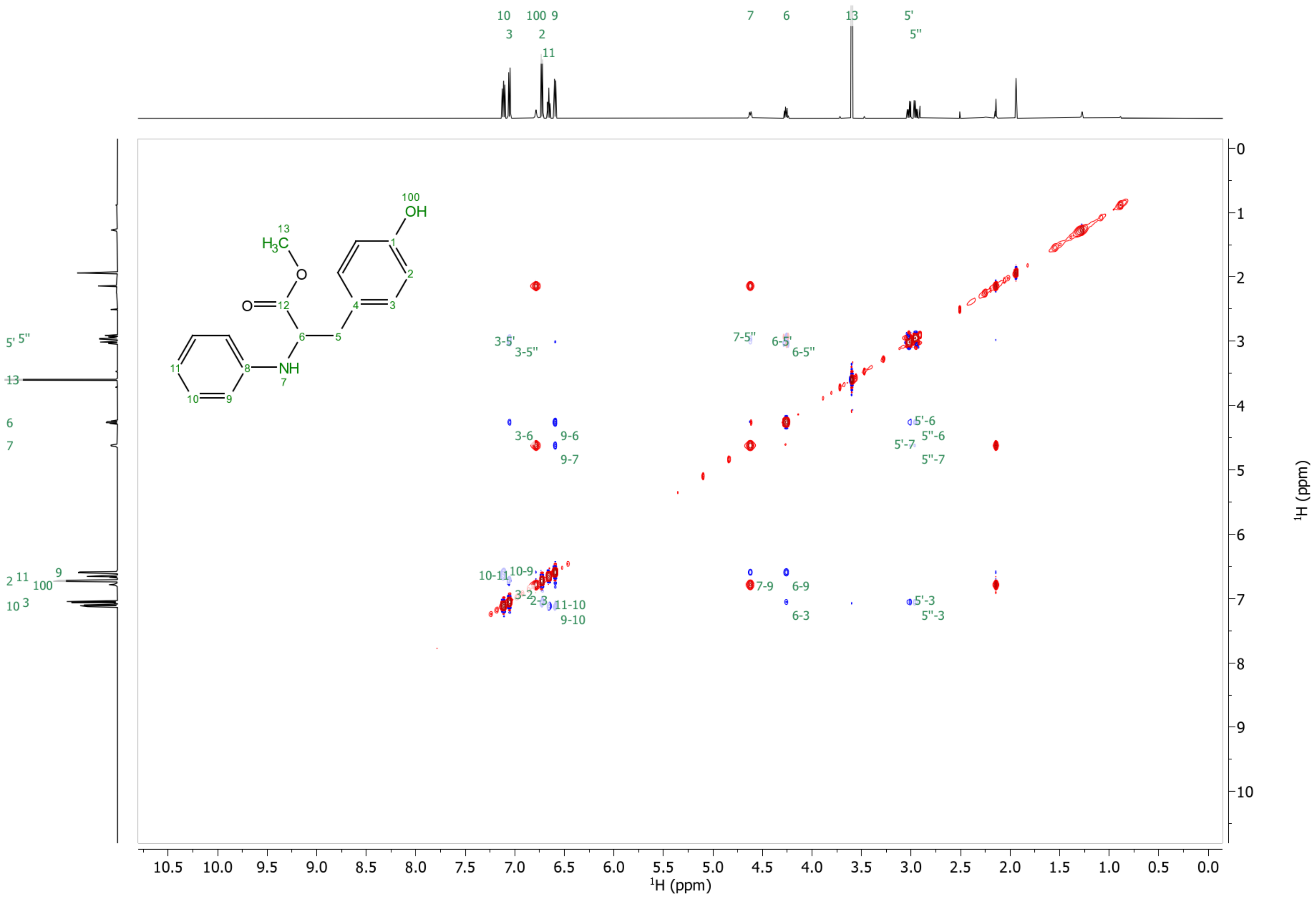

^{1}H {^{15}N} HMBC NMR spectrum of S14

600 MHz, CD_3CN, 298 K

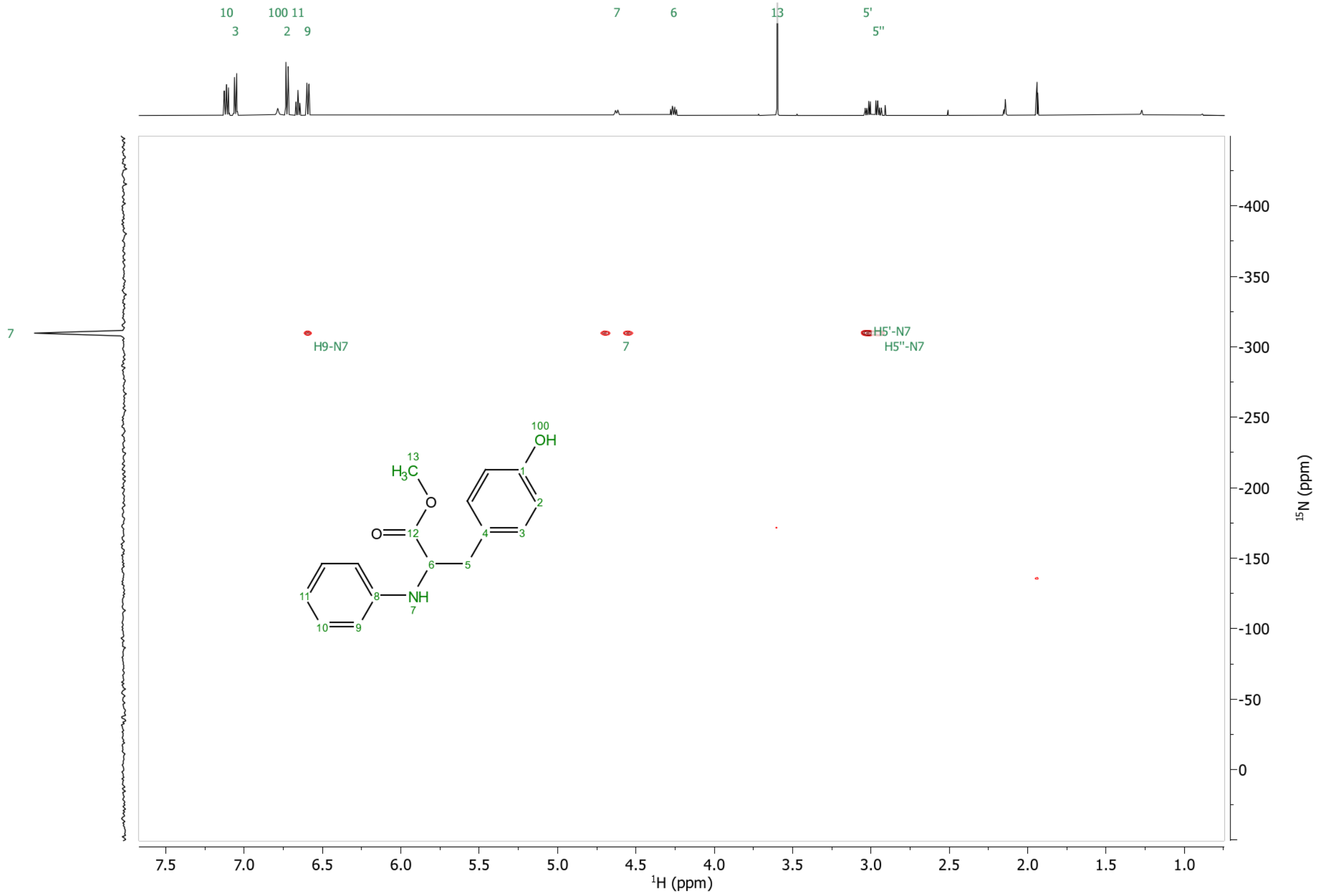

^{1}H NMR spectrum of S12

600 MHz, Methanol-d_4, 298 K

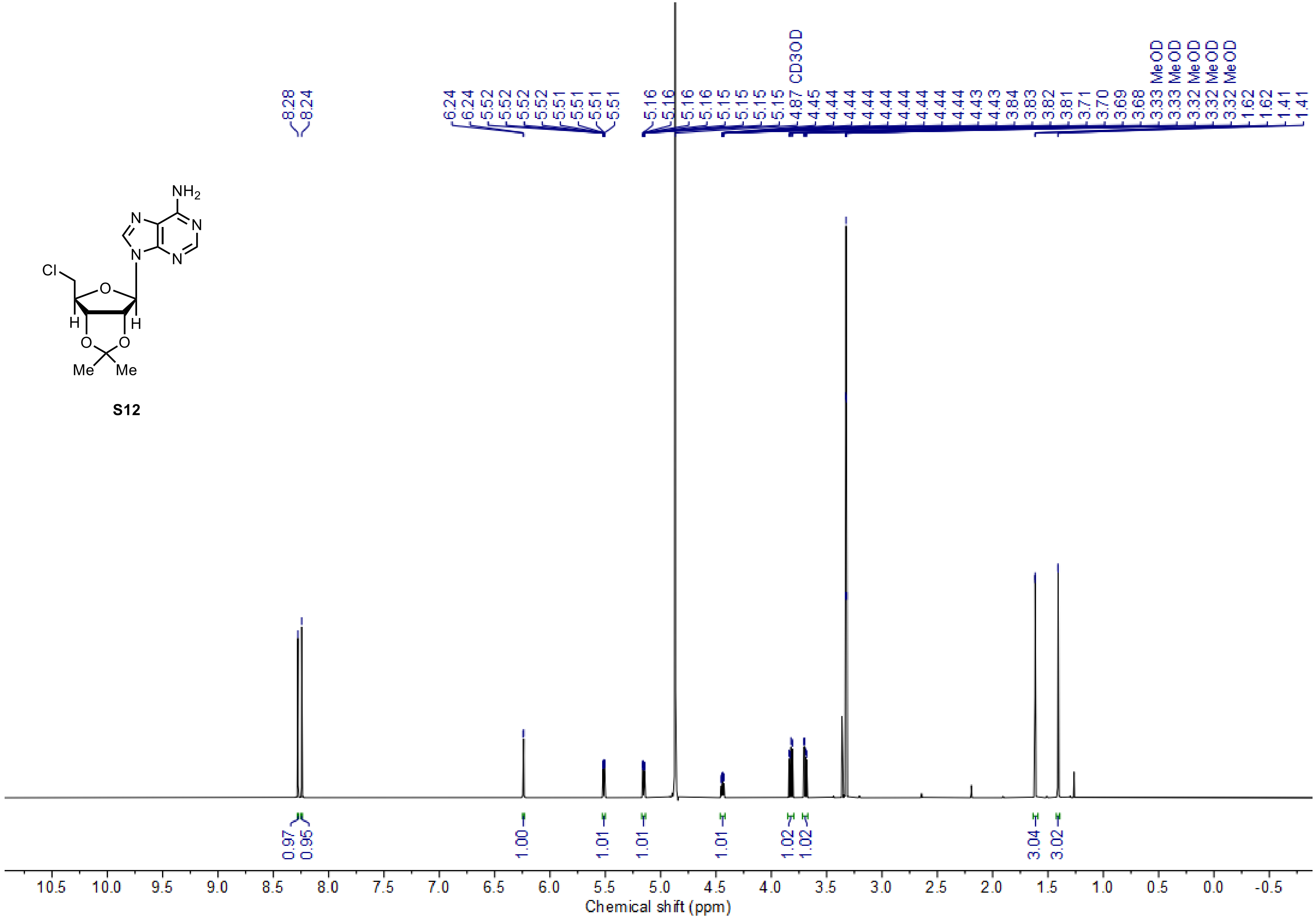

S12

^{13}C NMR spectrum of S12

151 MHz, Methanol-d_4, 298 K

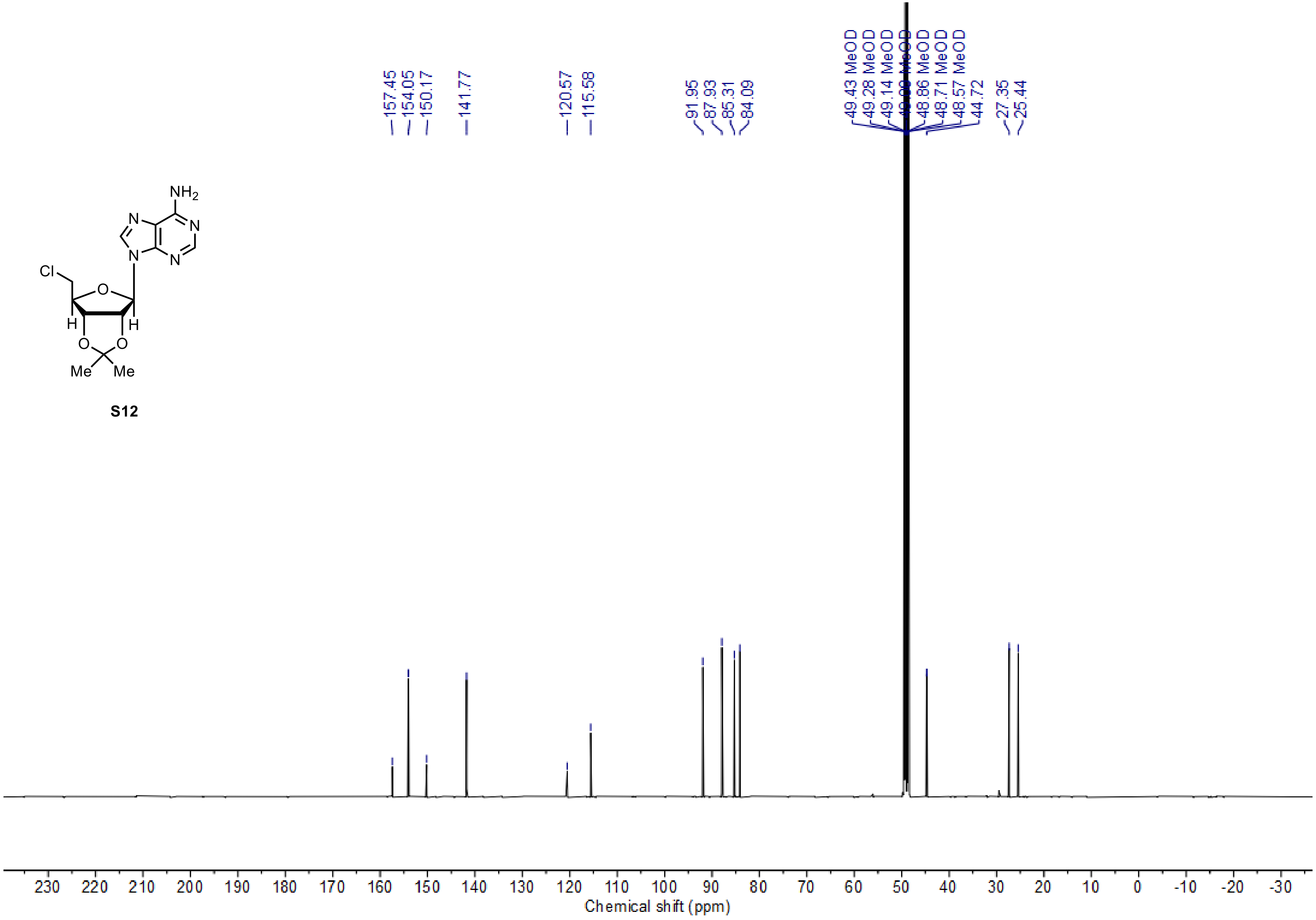

^{1}H NMR spectrum of 10

600 MHz, Methanol-d_4, 298 K

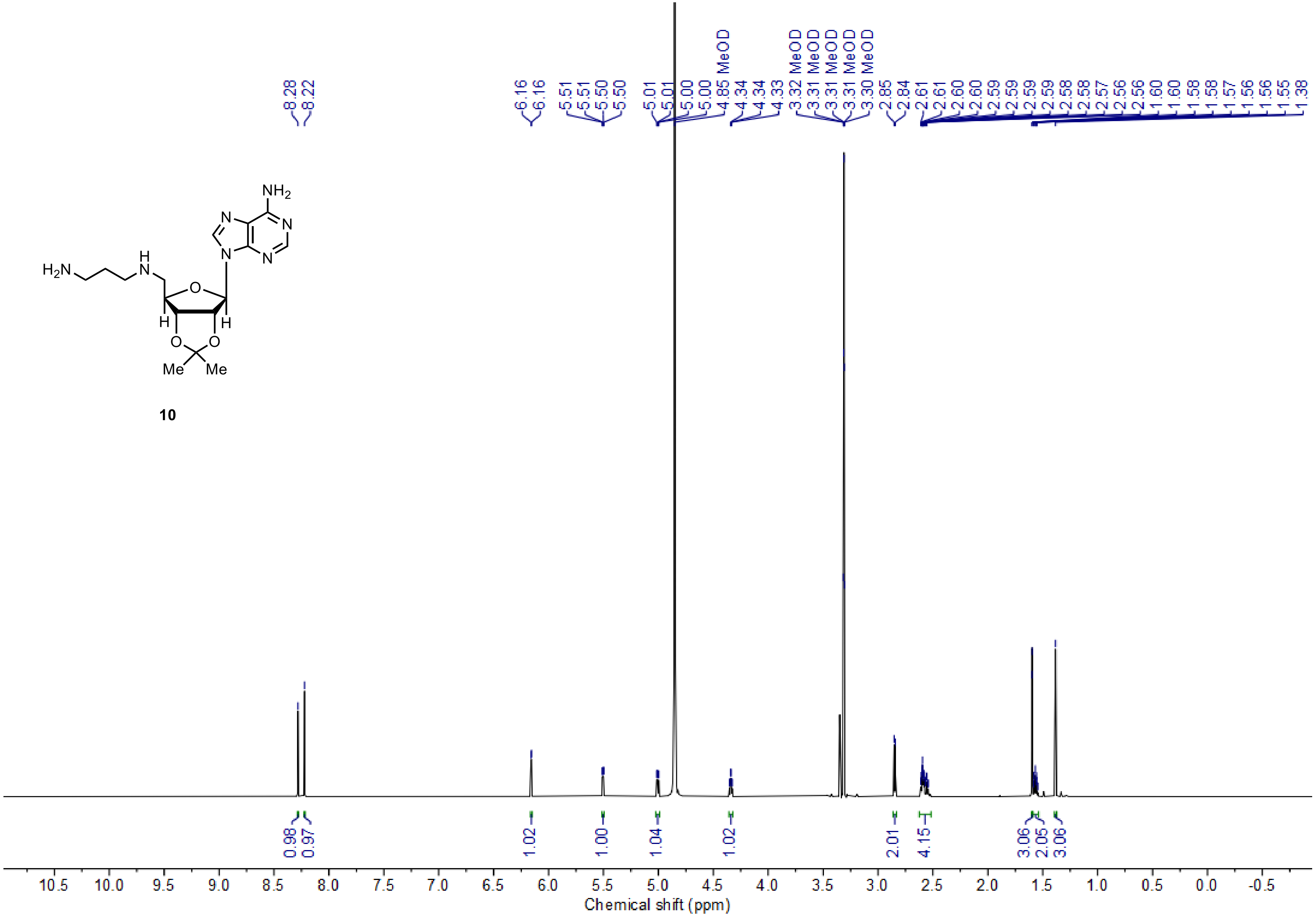

10

^{13}C NMR spectrum of 10

151 MHz, Methanol-d_4, 298 K

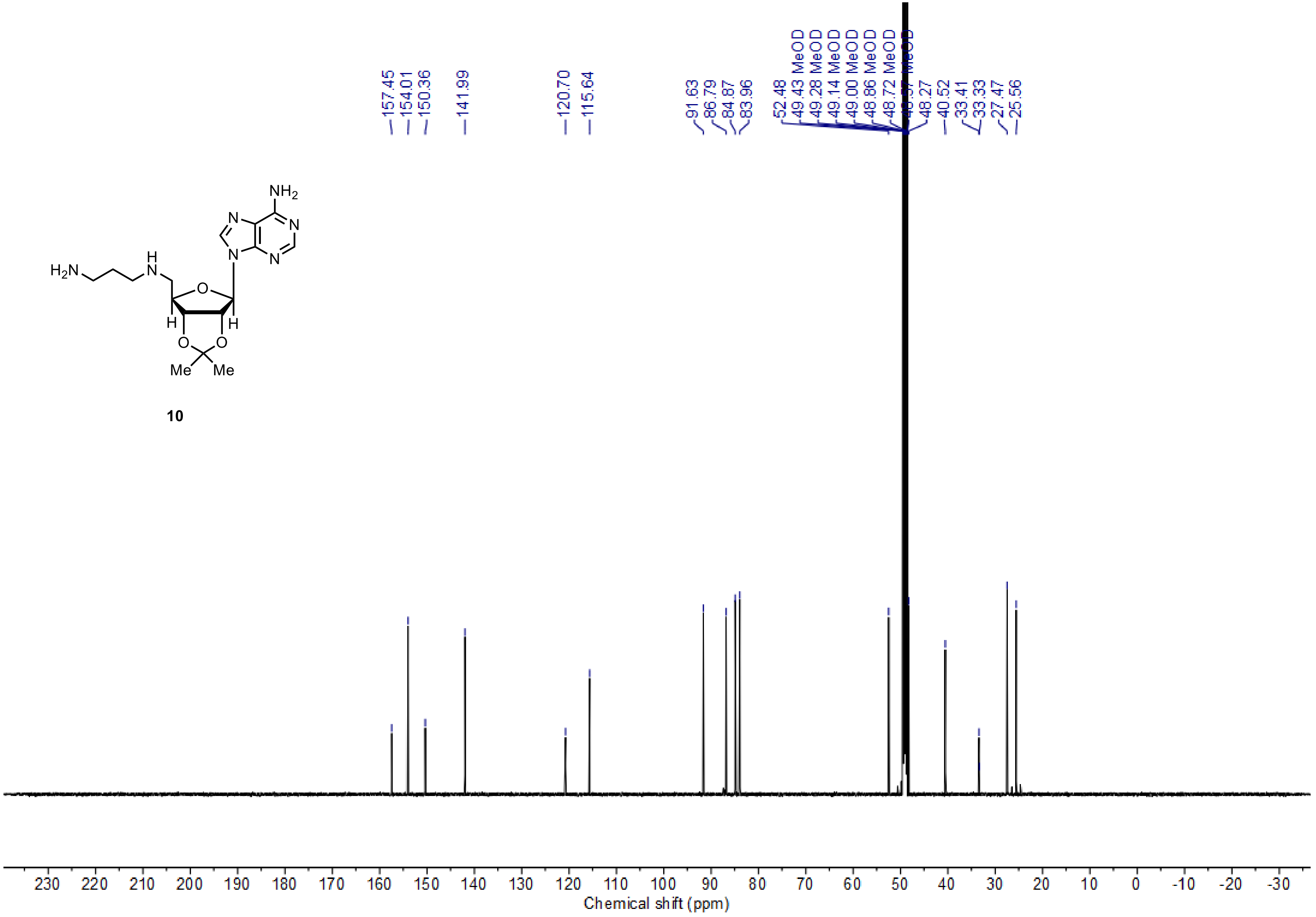

^{1}H NMR spectrum of 11

600 MHz, CD_3CN, 298 K

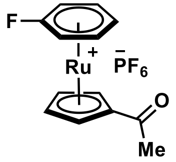

11

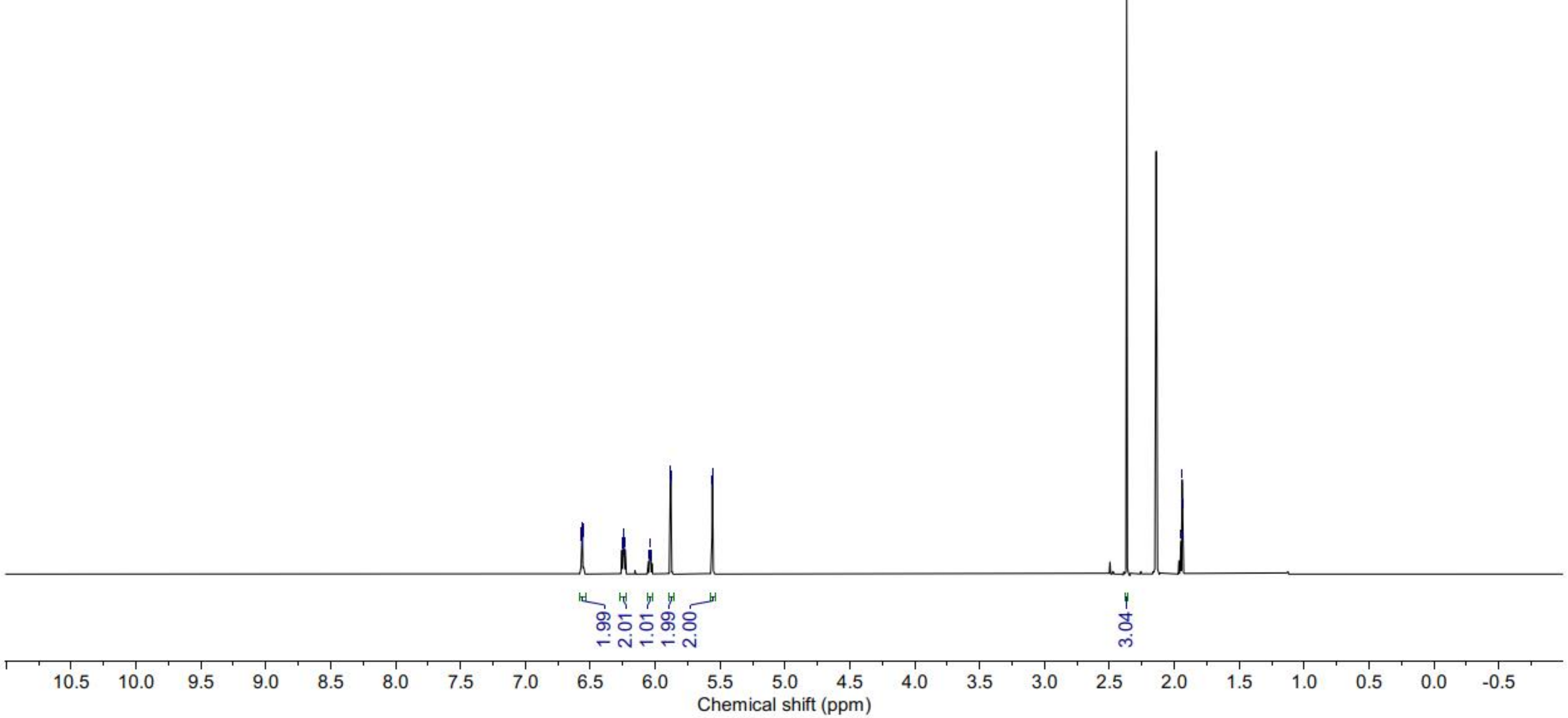

^{13}C NMR spectrum of 11

151 MHz, CD_3CN, 298 K

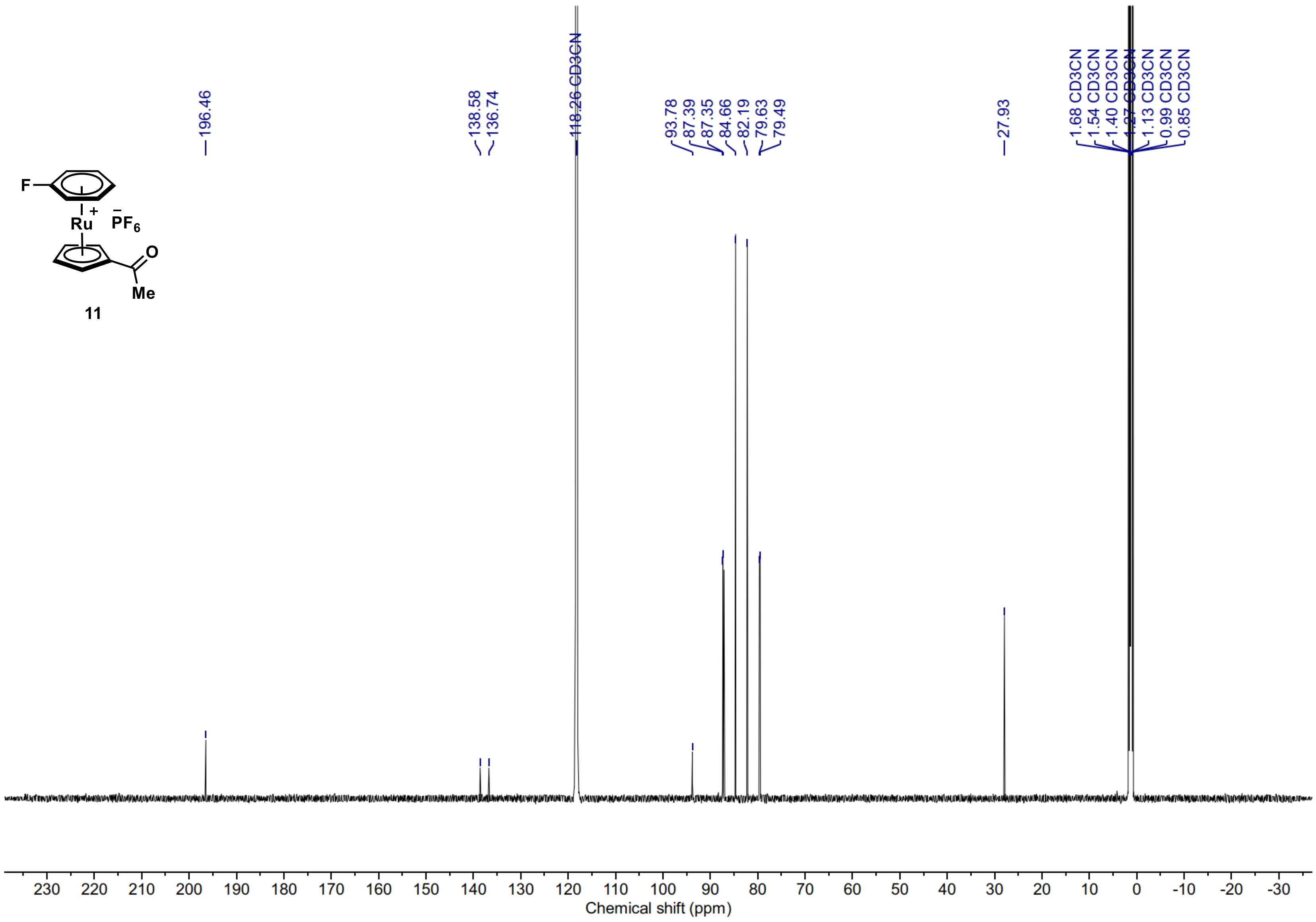

^{19}F NMR spectrum of 11

565 MHz, CD_3CN, 298 K

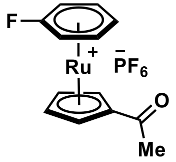

11

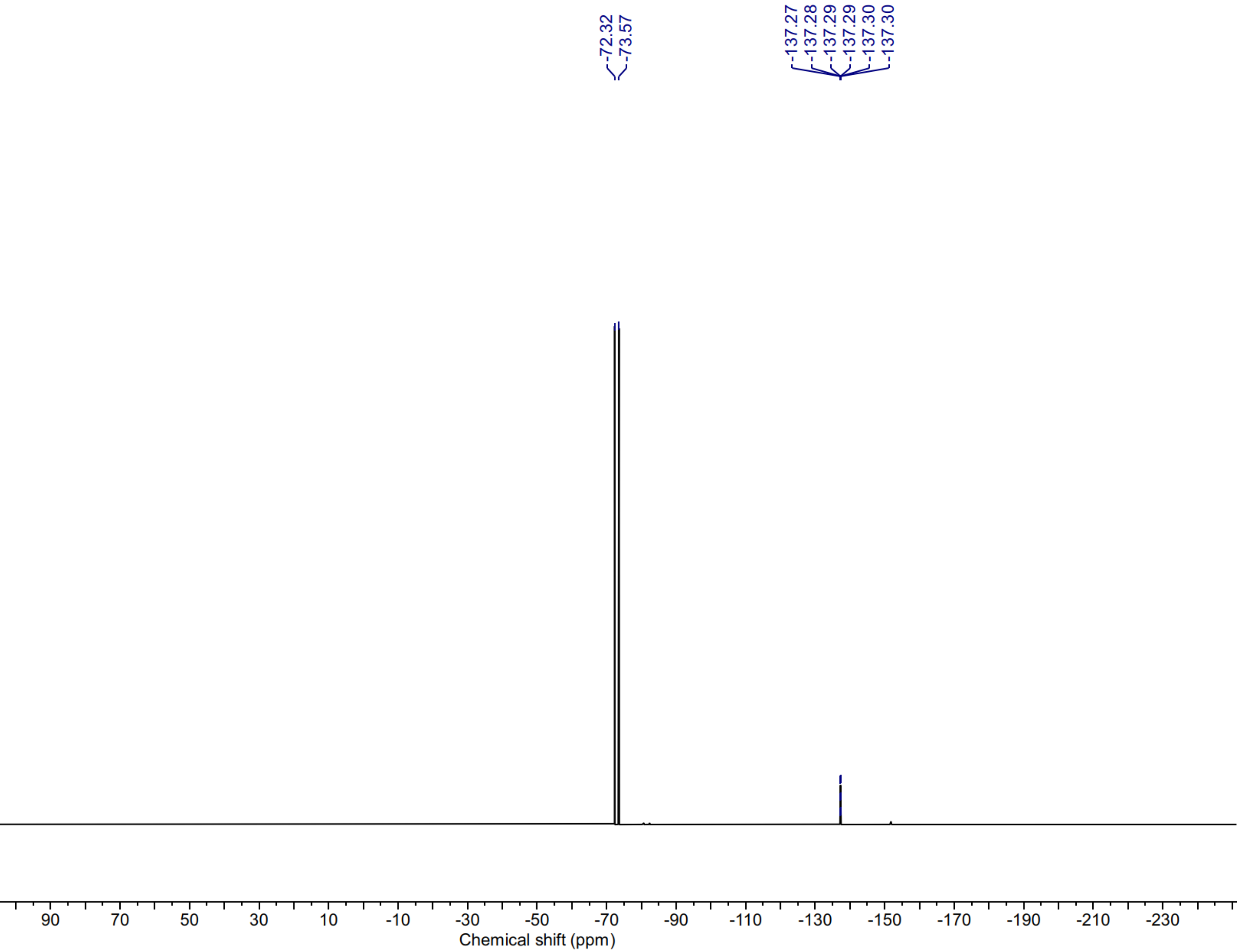

^{31}P NMR spectrum of 11

243 MHz, CD_3CN, 298 K

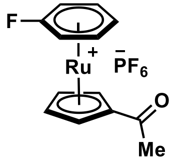

11

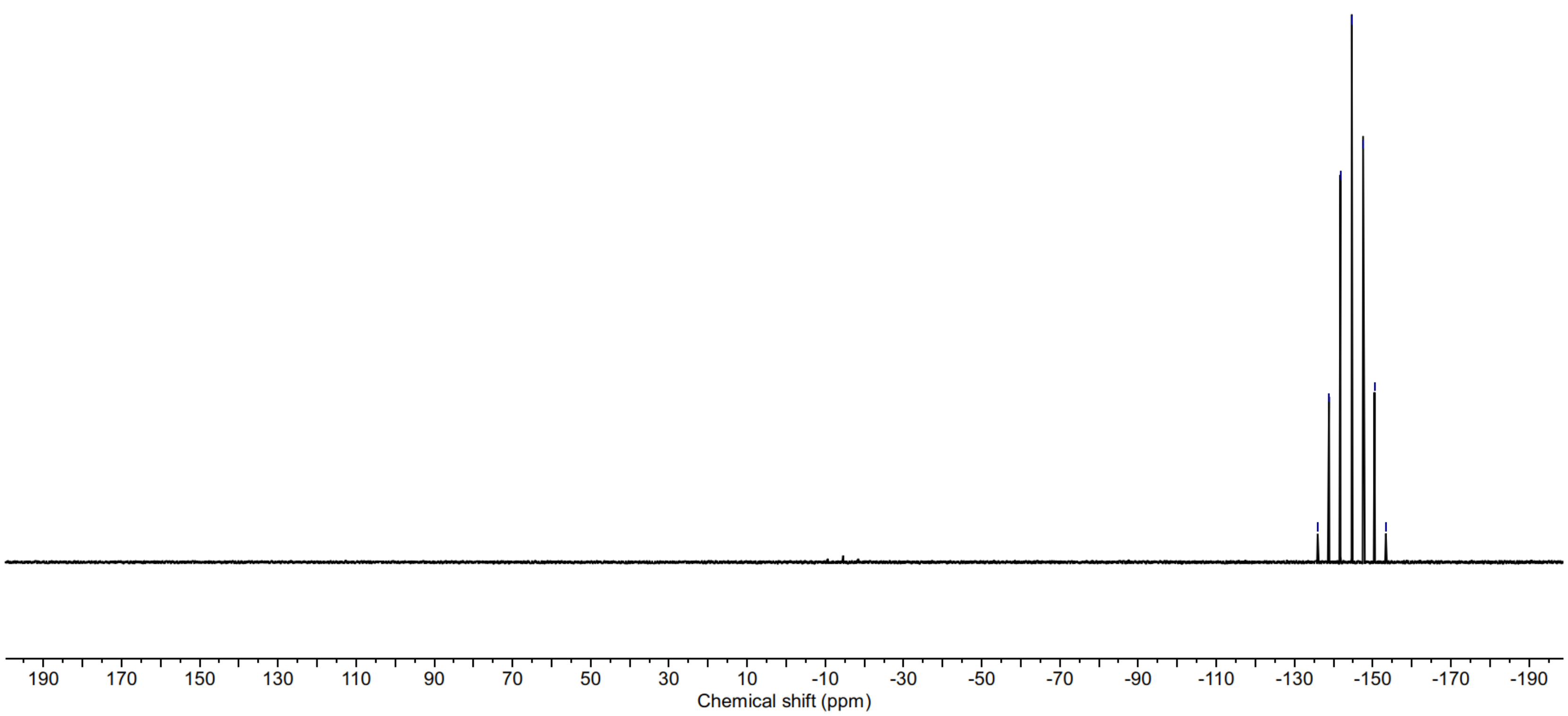

^{1}H NMR spectrum of 12

600 MHz, DMSO-d_6, 298 K

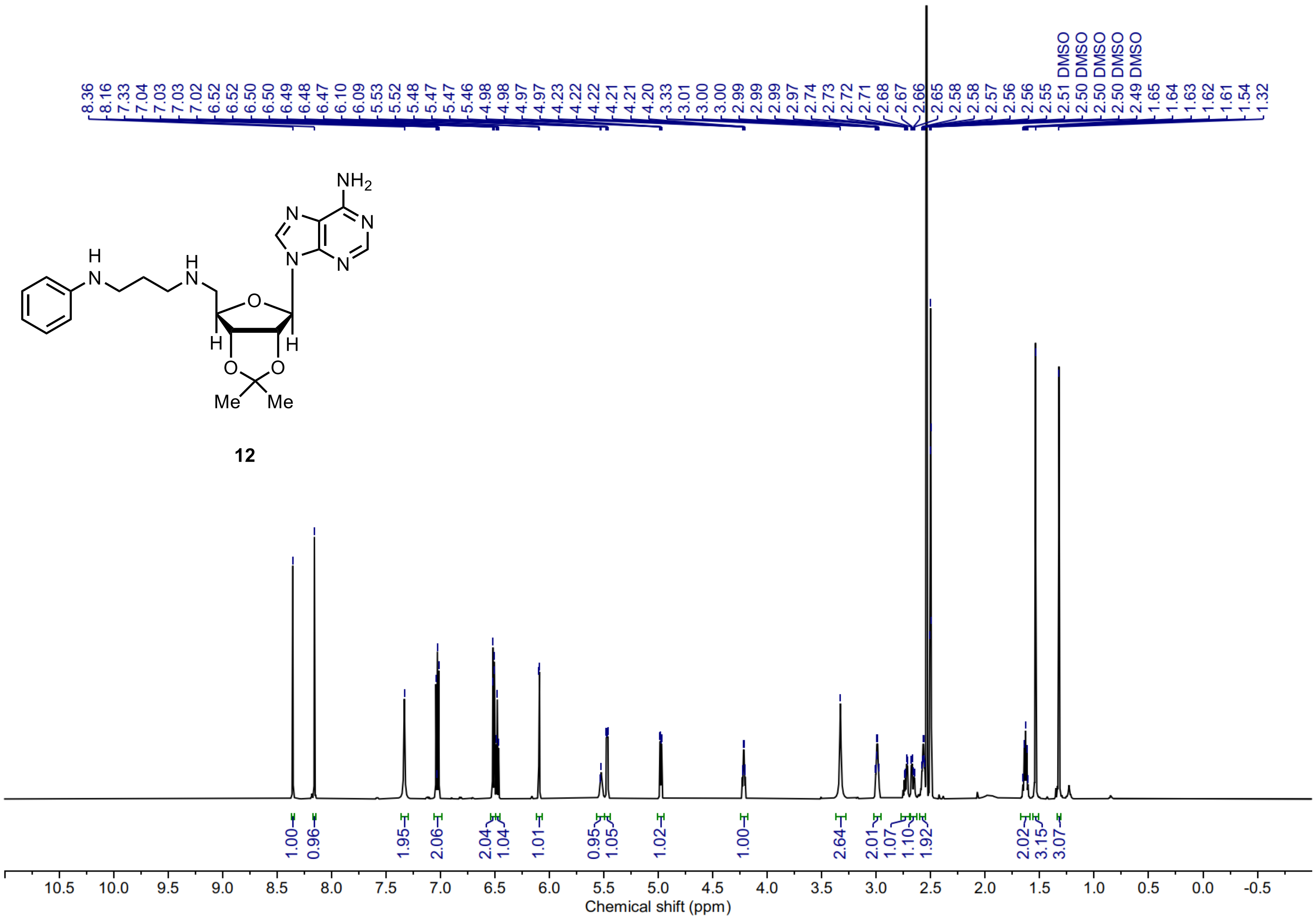

^{13}C NMR spectrum of 12

151 MHz, DMSO-d_6, 298 K

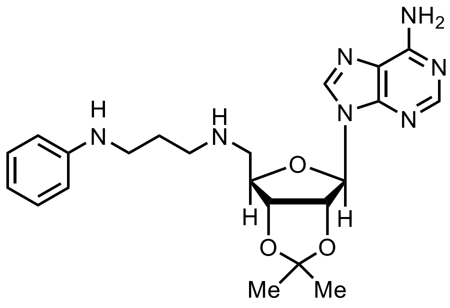

12

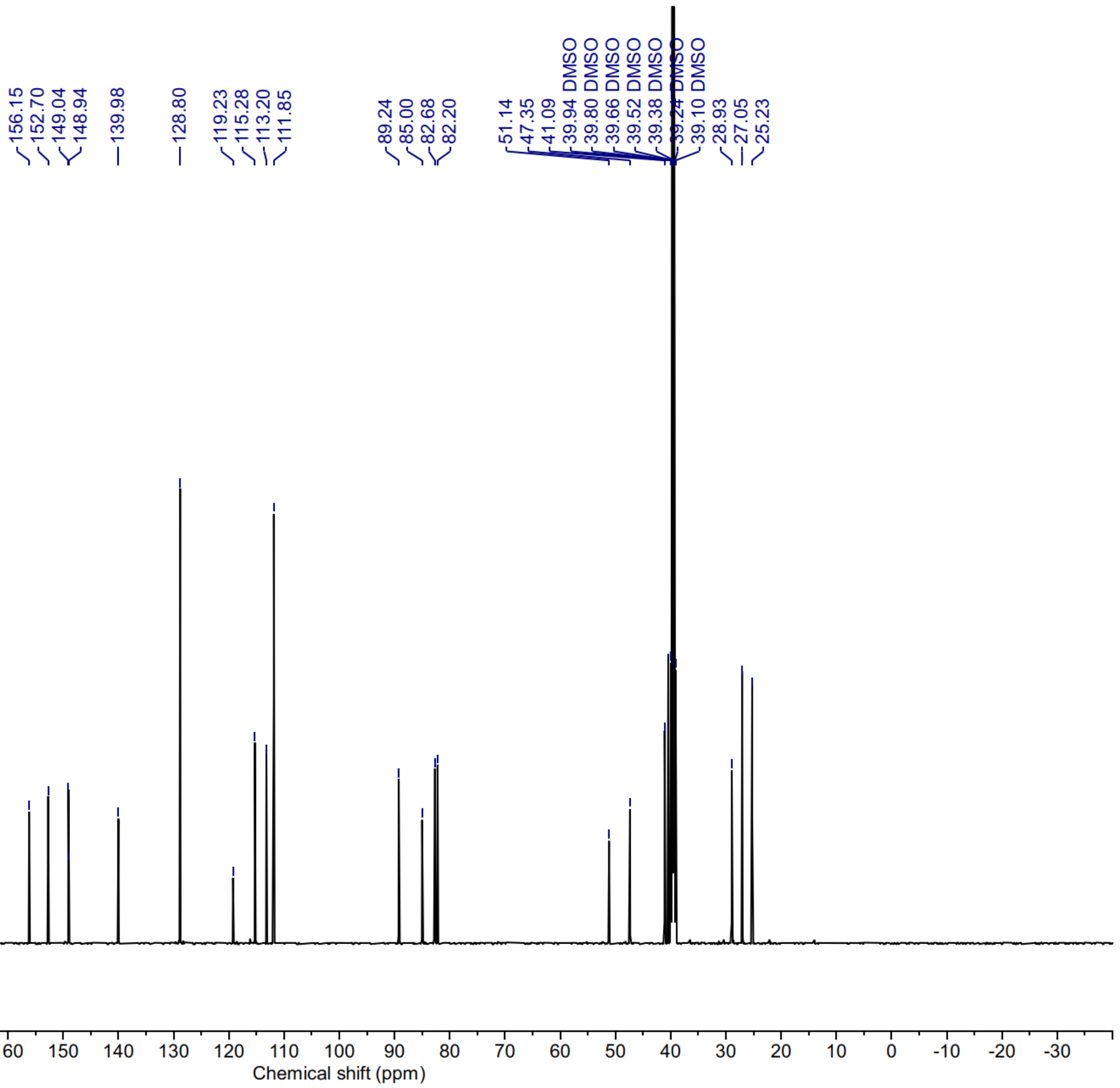

^{1}H NMR spectrum of 12 (Deuterated sample)

600 MHz, DMSO-d_6, 298 K (sample with partial deuteration from CD_3OD)

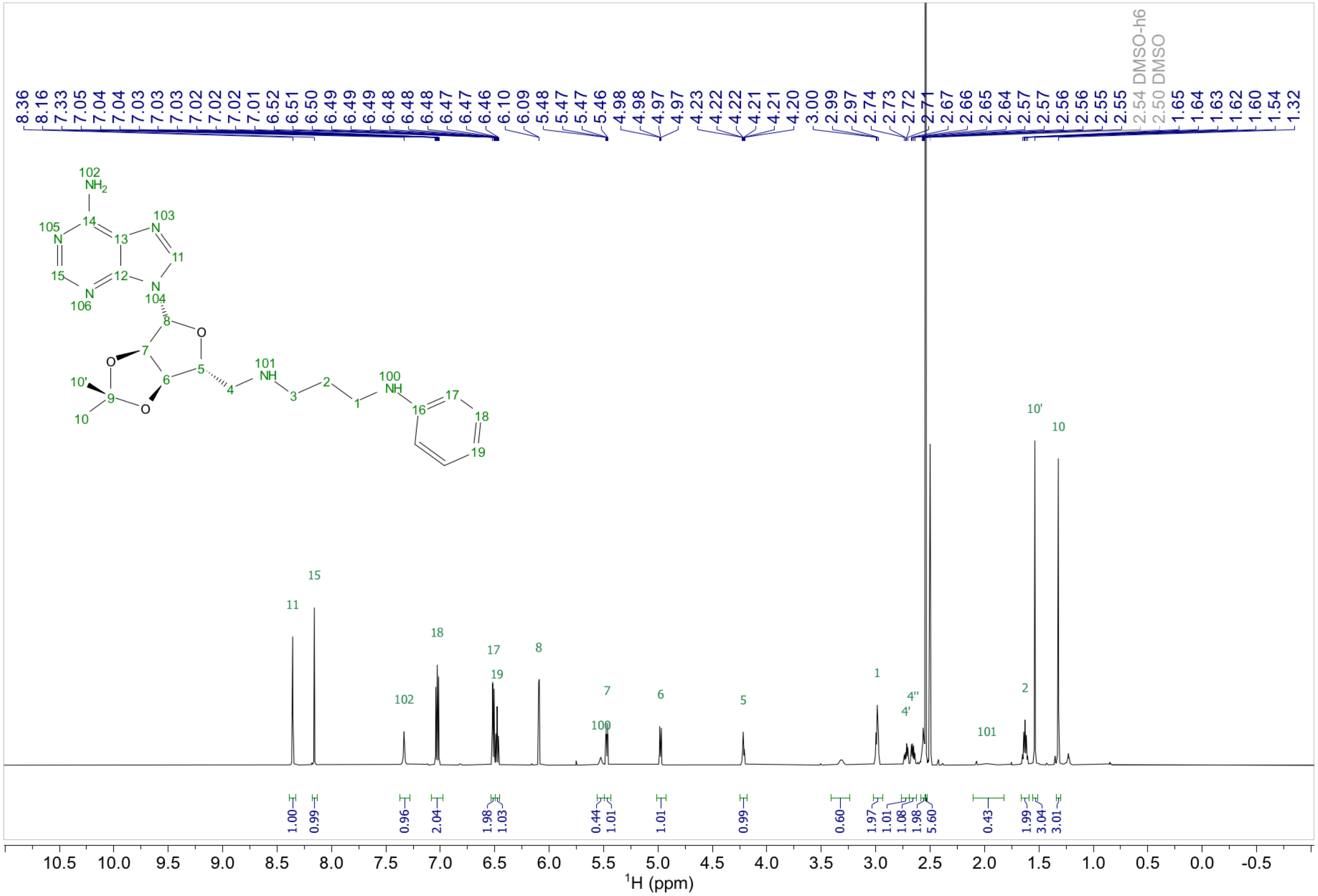

^{13}C NMR spectrum of 12 (Deuterated sample)

151 MHz, DMSO-d_6, 298 K

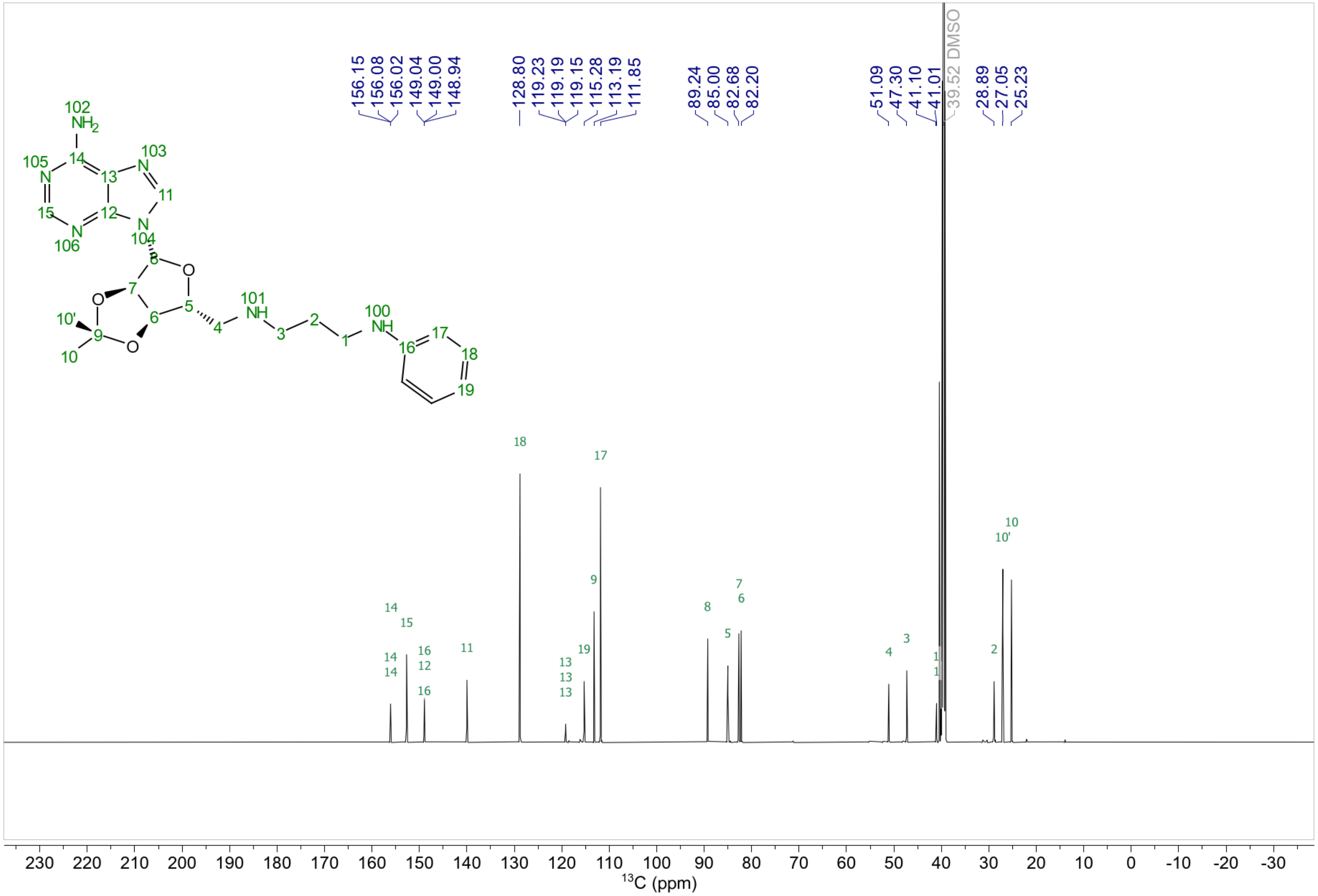

$^{1}H\ \{^{13}C\}$ HMBC NMR spectrum of 12

600 MHz, DMSO-d_6, 298 K; section from 3.25–4.25 ppm of ^{1}H NMR spectrum omitted for clarity due to lack of peaks in this section.(All the 2D NMR measurement of compound **12** was performed with the NMR sample with partial deuteration, from previously used CD_3OD solvent).

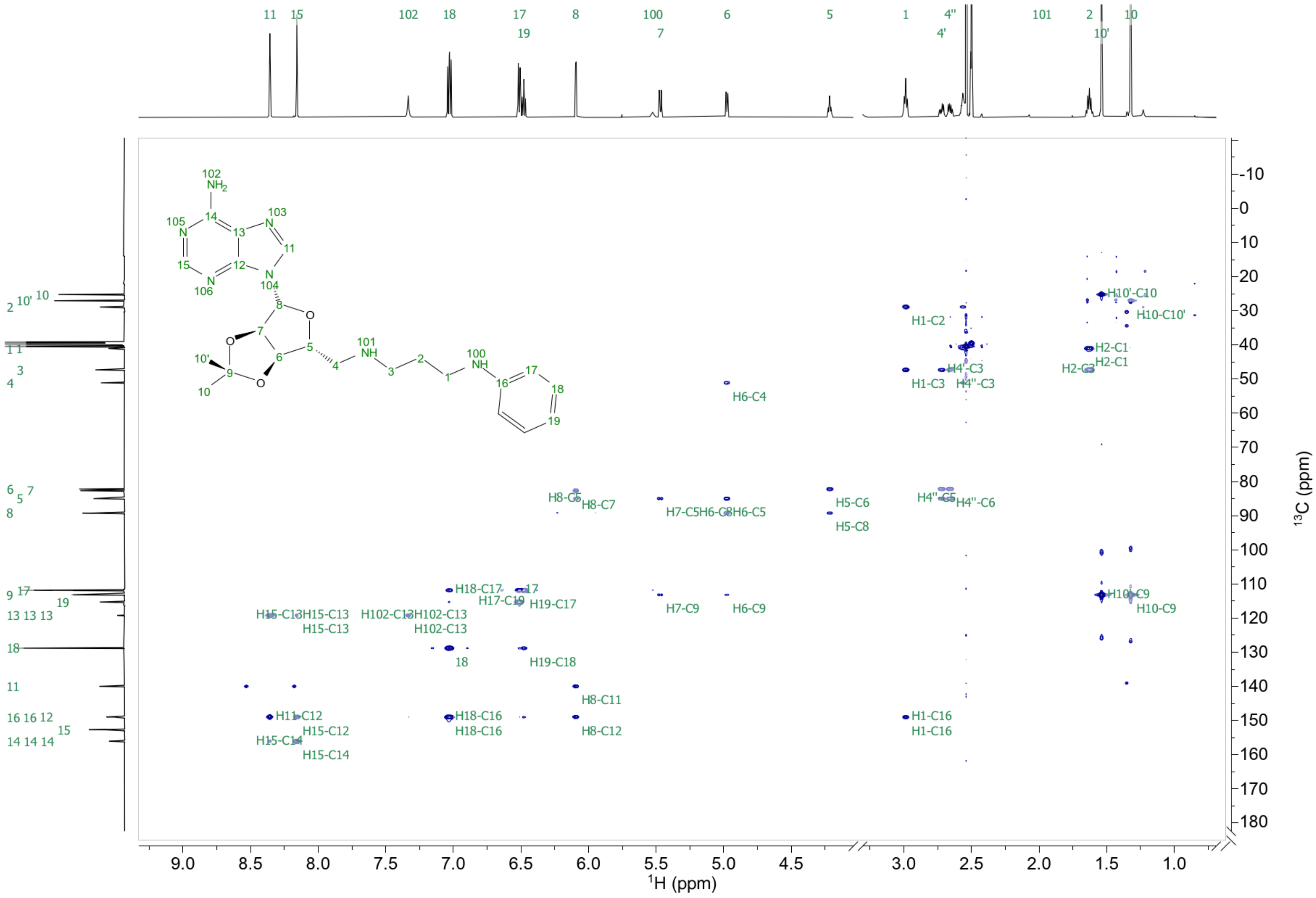

^{1}H {^{13}C} HSQC NMR spectrum of 12

600 MHz, DMSO-d_6, 298 K

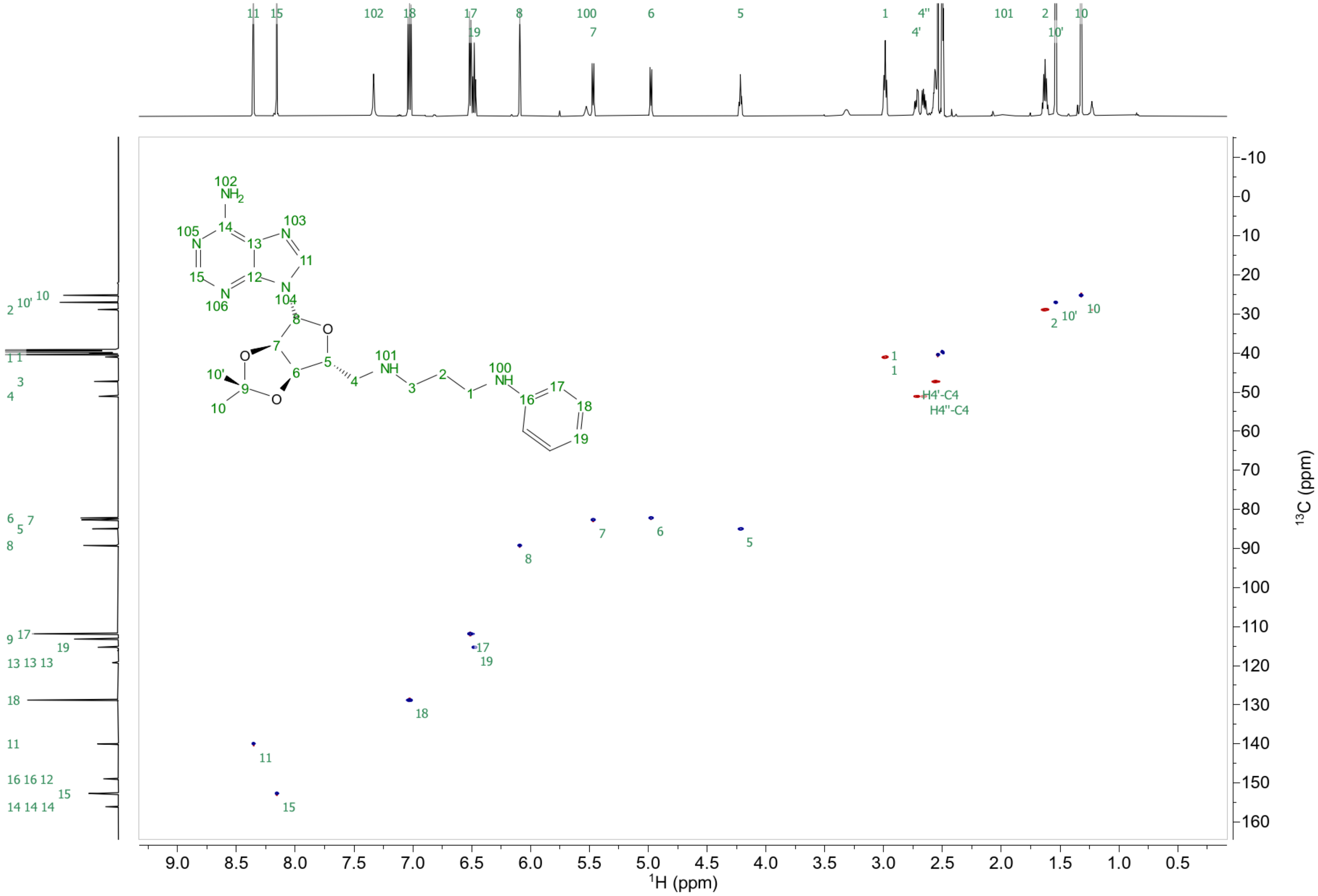

^{1}H ROESY NMR spectrum of 12

600 MHz, DMSO-d_6, 298 K

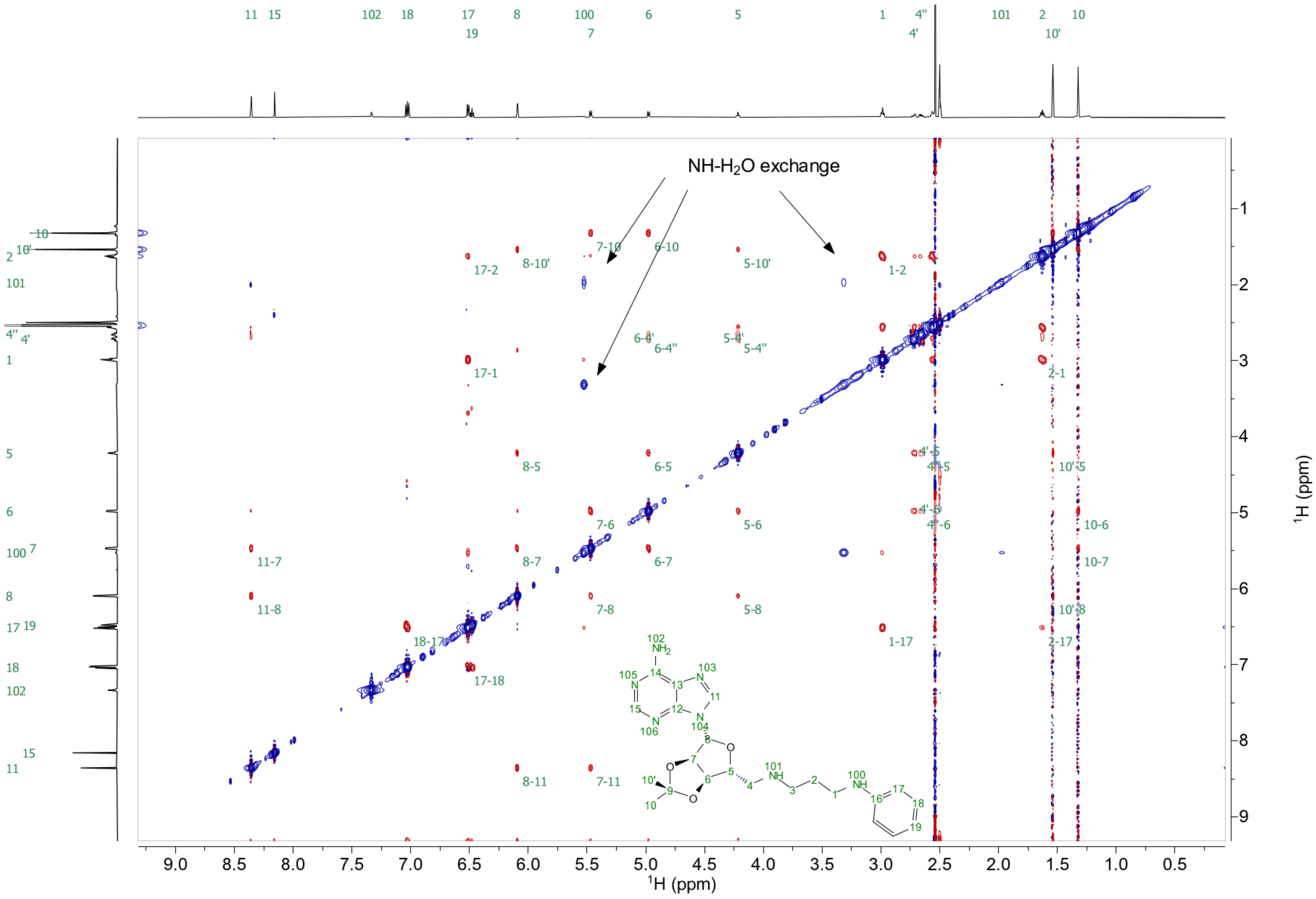

$^{1}H\{^{15}N\}$ HMBC NMR spectrum of 12

600 MHz, DMSO-d_6, 298 K

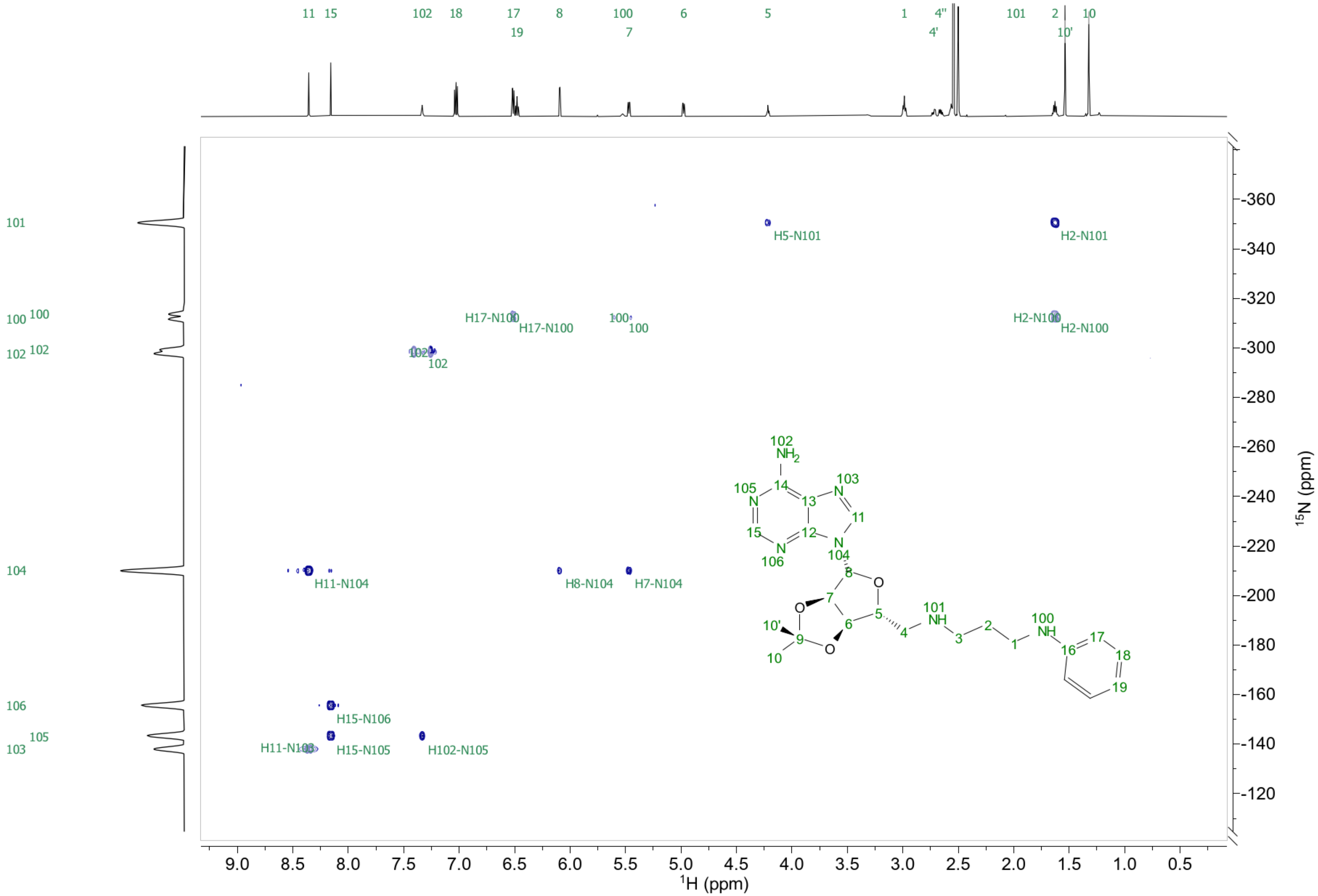

^{1}H COSY NMR spectrum of 12

600 MHz, DMSO-d_6, 298 K

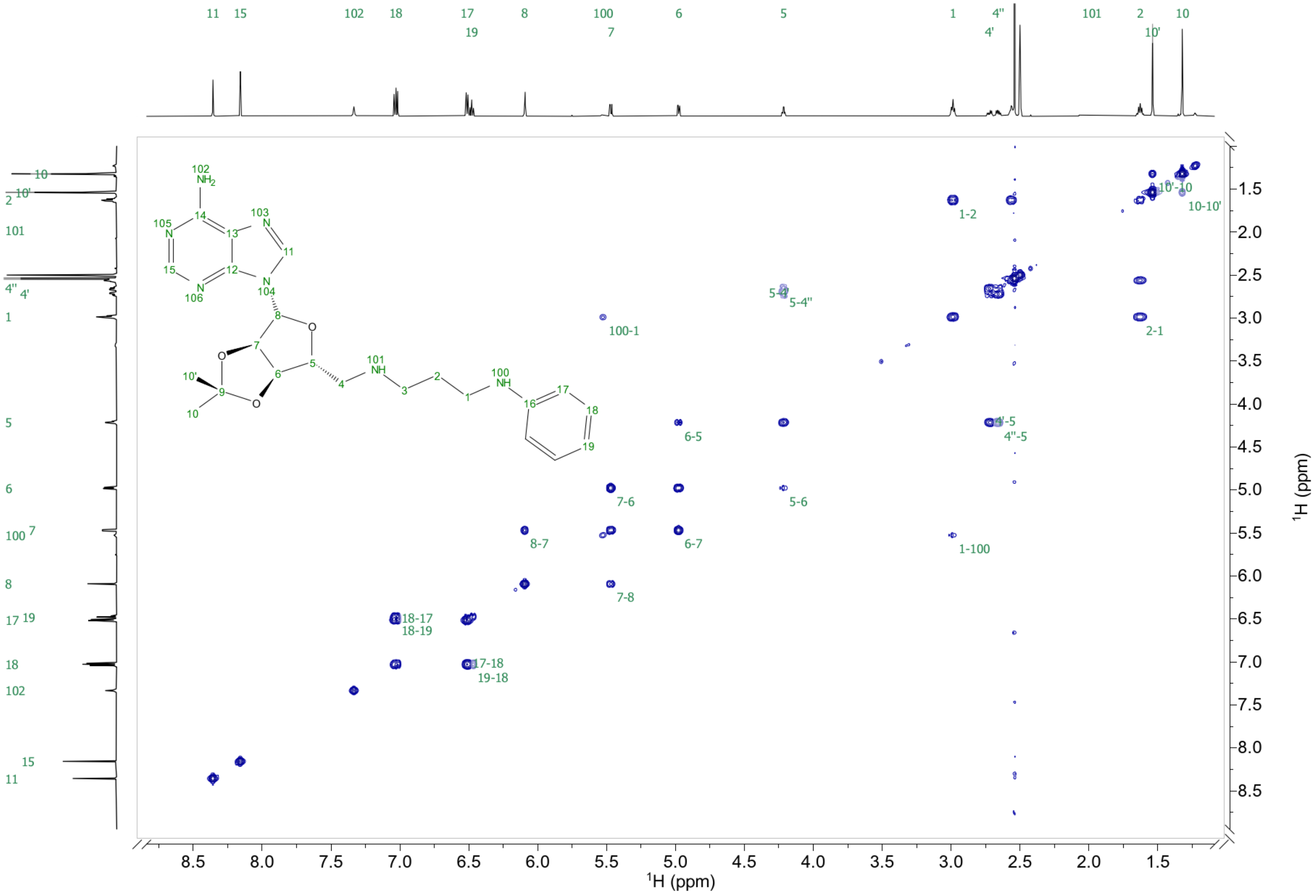

^{1}H NMR spectrum of 14

600 MHz, DMSO-d_6, 298 K

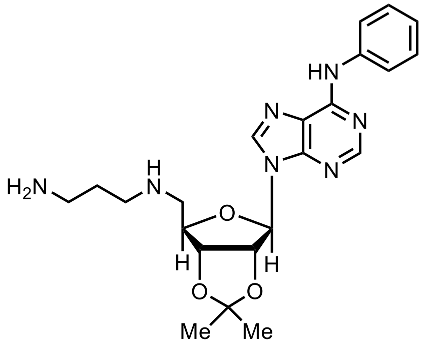

14

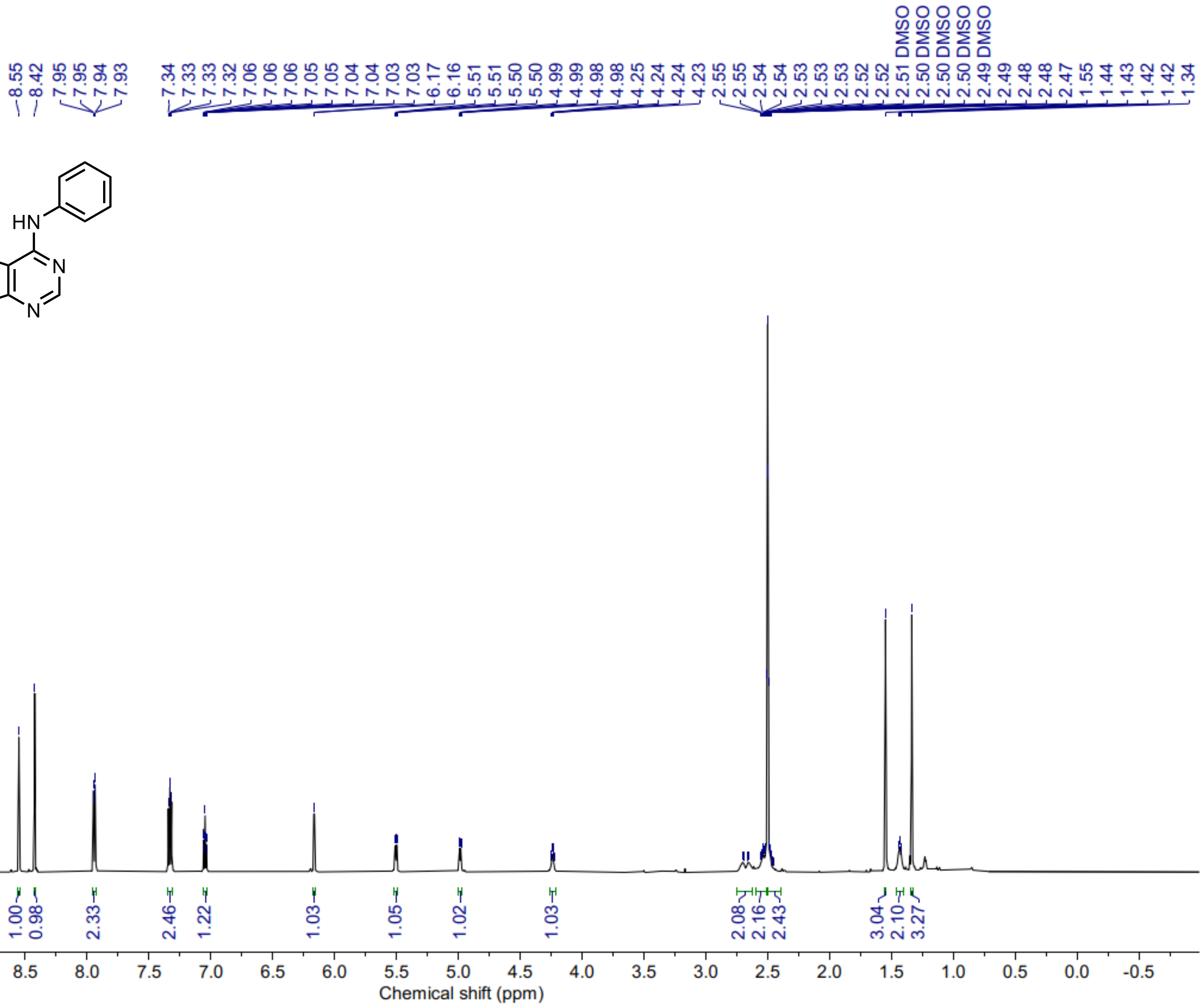

^{13}C NMR spectrum of 14

151 MHz, DMSO-d_6, 298 K

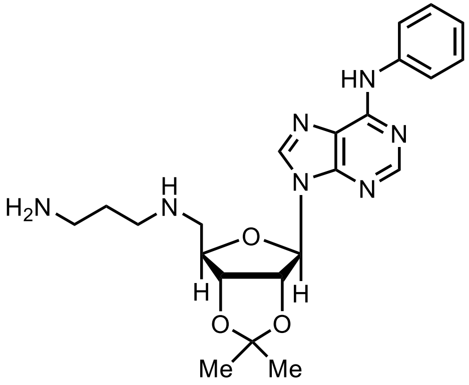

14

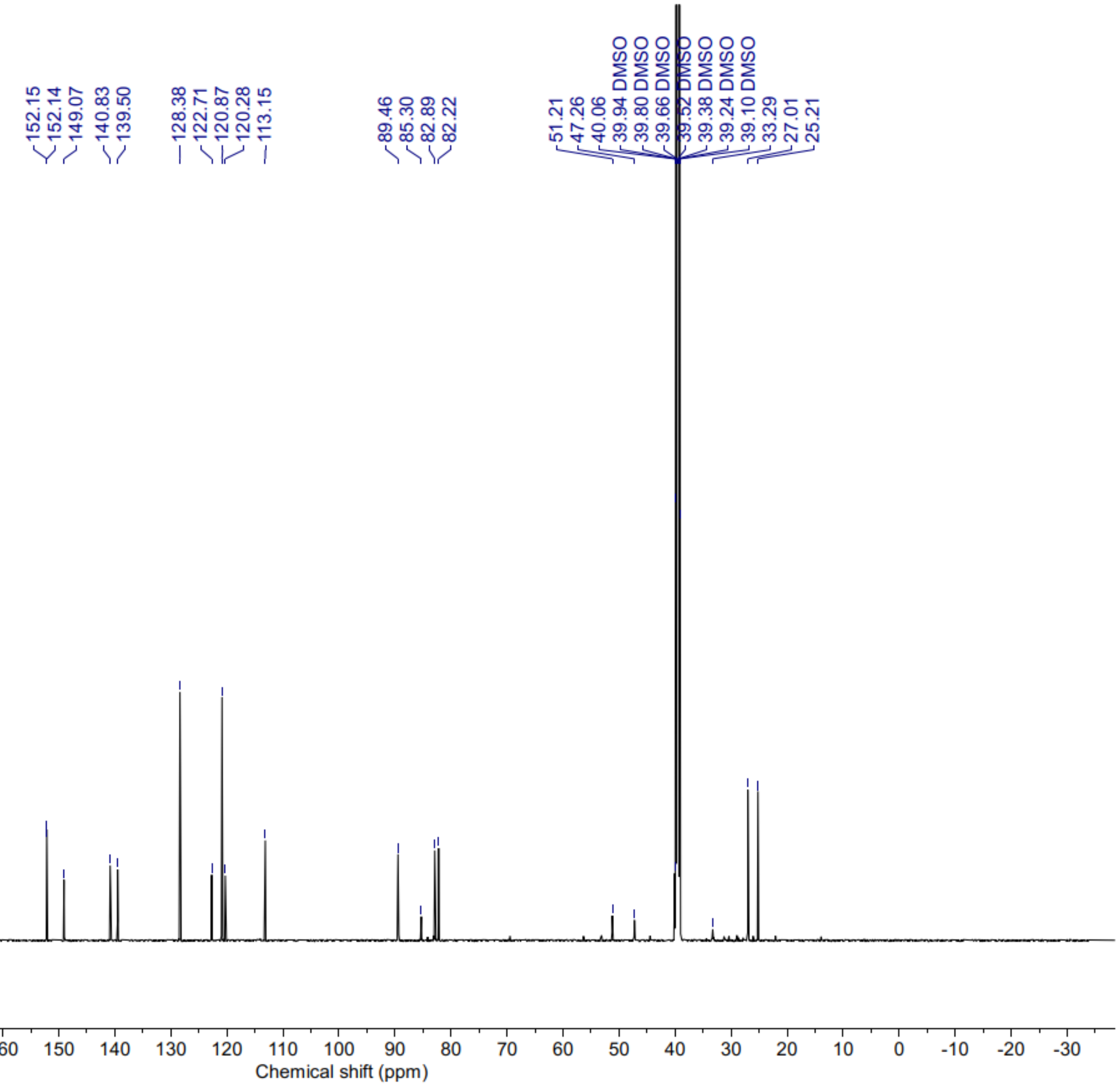

$^{1}H\ \{^{13}C\}$ HSQC NMR spectrum of 14

600 MHz, DMSO-d_6, 298 K

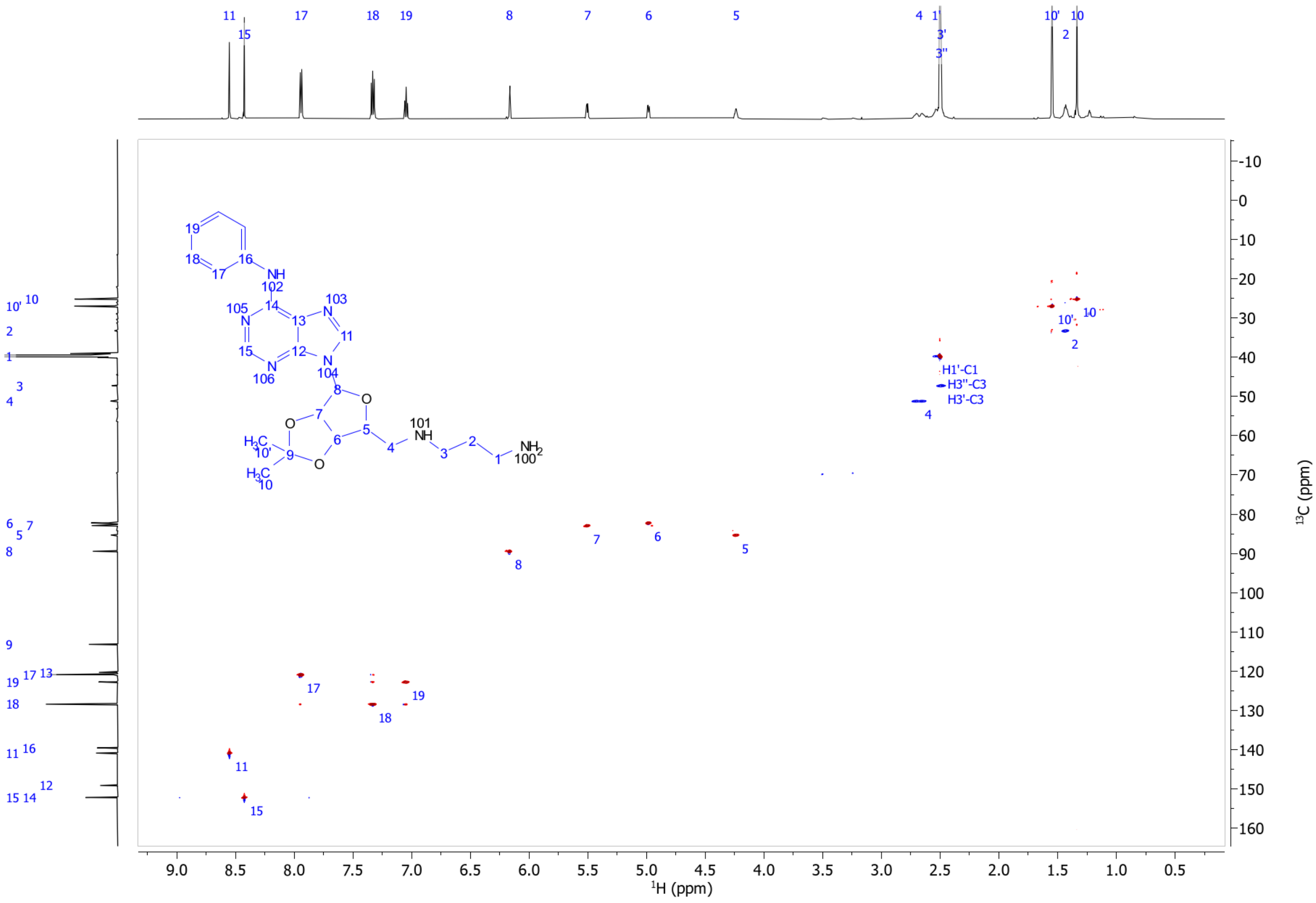

^{1}H {^{13}C} HMBC NMR spectrum of 14

600 MHz, DMSO-d_6, 298 K; section from 2.75–4.0 ppm of ^{1}H NMR spectrum omitted for clarity due to lack of peaks in this section.

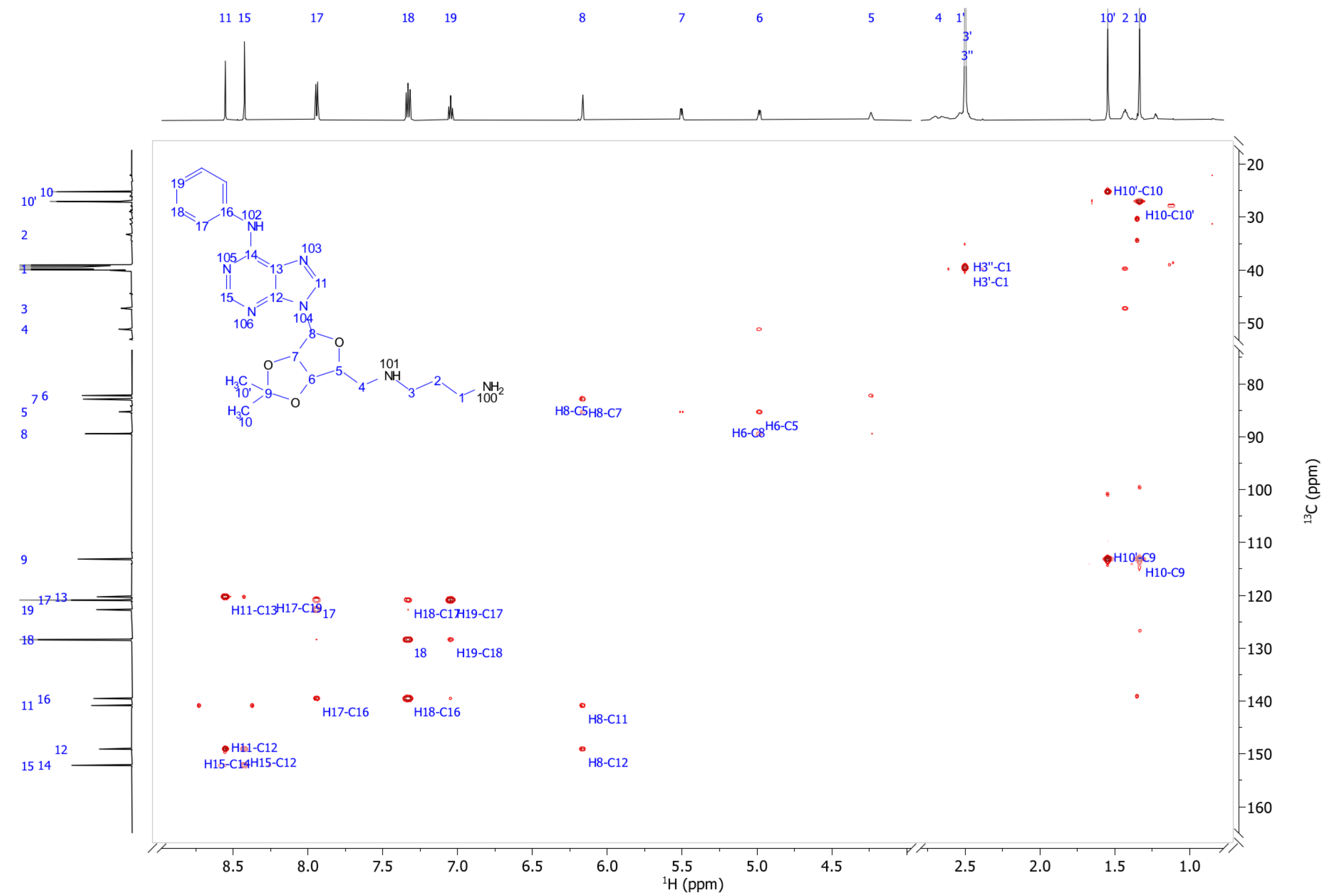

^{1}H {^{15}N} HMBC NMR spectrum of 14

600 MHz, DMSO-d_6, 298 K

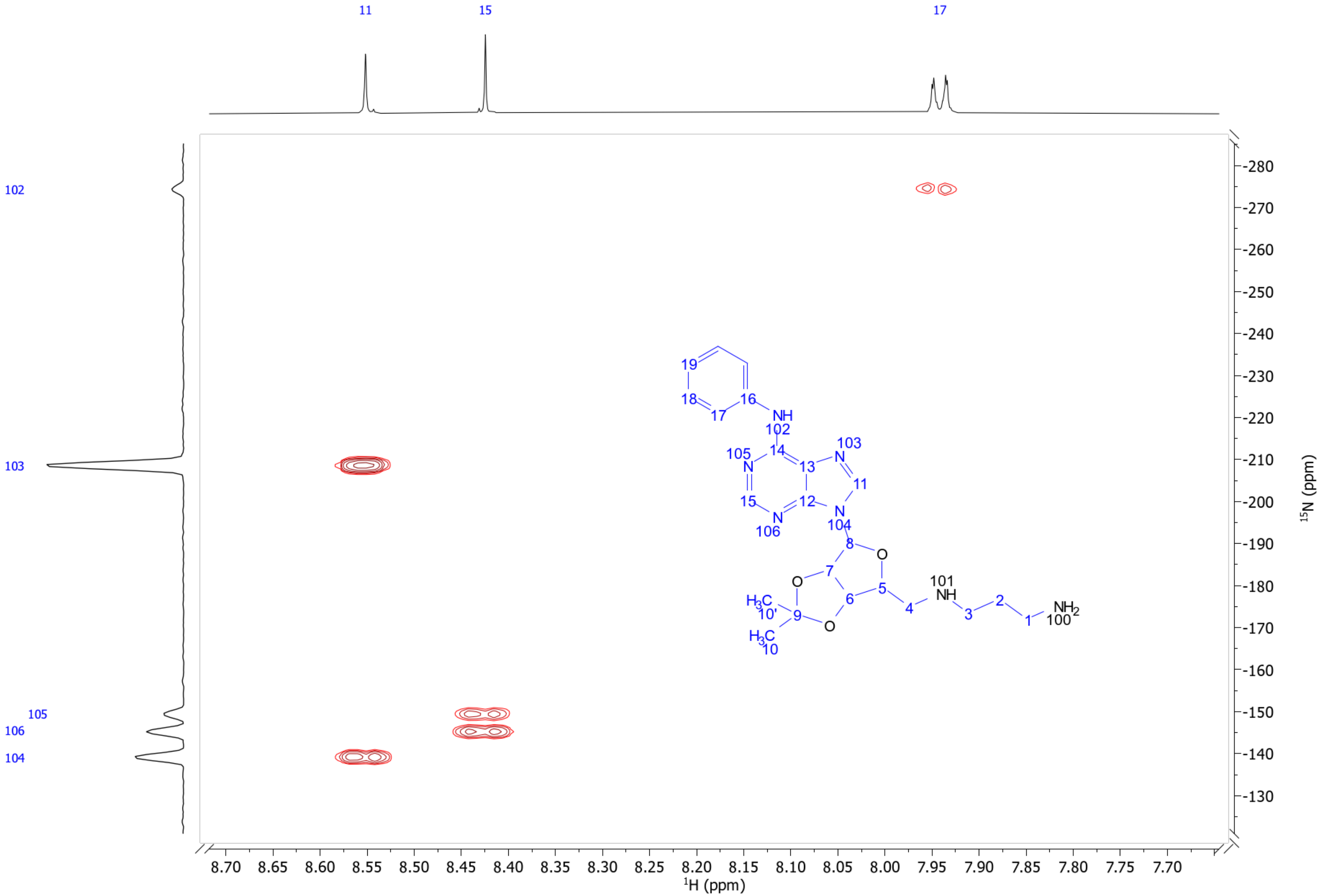

^{1}H COSY NMR spectrum of 14

600 MHz, DMSO-d_6, 298 K

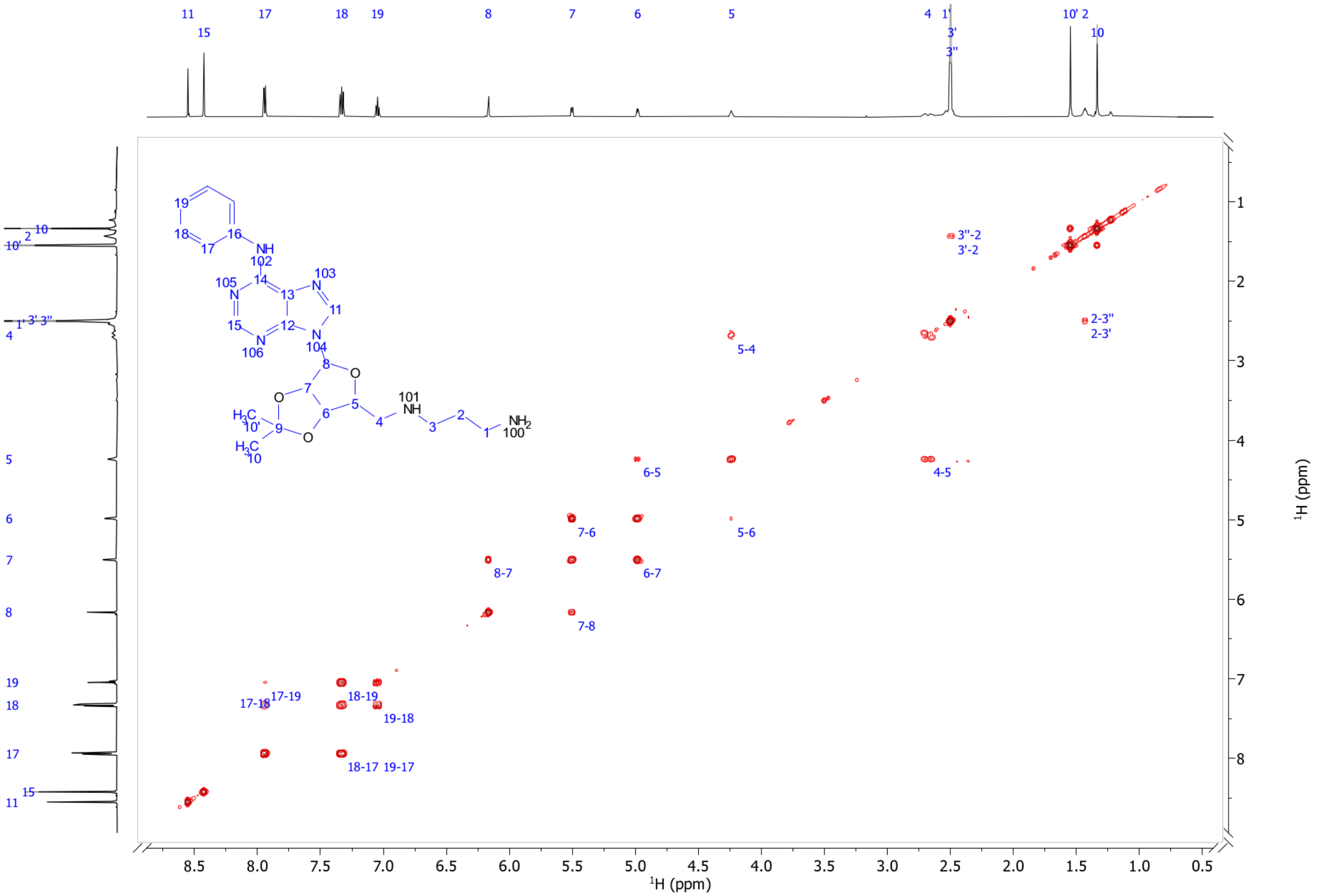

^{1}H ROESY NMR spectrum of 14

600 MHz, DMSO-d_6, 298 K

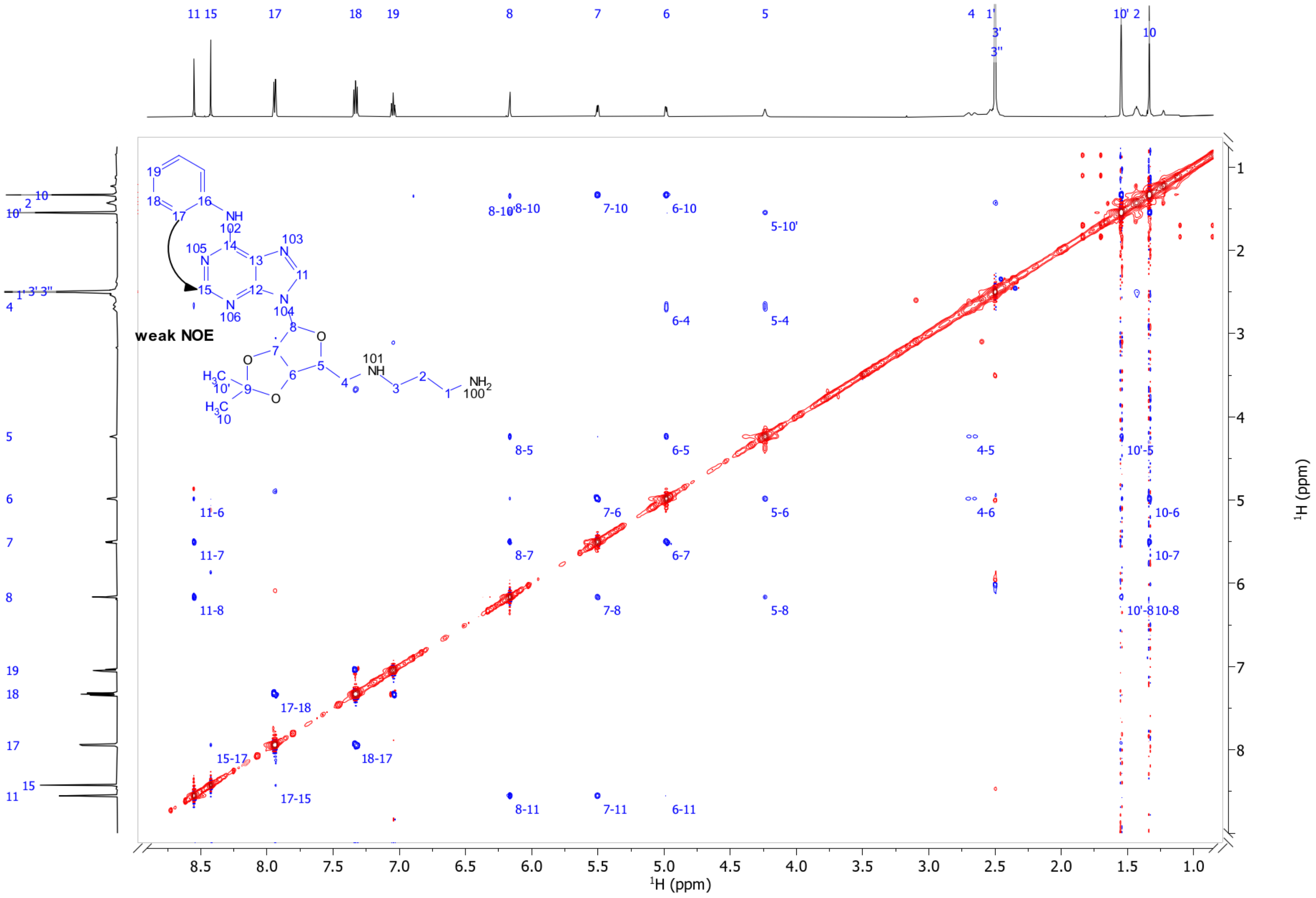

References

1. Fulmer, G. R. *et al.* NMR chemical shifts of trace impurities: common laboratory solvents, organics, and gases in deuterated solvents relevant to the organometallic chemist. *Organometallics* **29**, 2176–2179 (2010).
2. Hale, P., A Handbook for DNA-Encoded Chemistry. John Wiley & Sons, Inc. (2014).
3. Sambrook, J., Molecular Cloning: a Laboratory Manual. Cold Spring Harbor, N.Y.: Cold Spring Harbor Laboratory Press (2001).
4. Panda, T. K., Gamer, M. T. & Roesky, P. W. An improved synthesis of sodium and potassium cyclopentadienide. *Organometallics* **22**, 877–878 (2003).
5. Shaik, S. & Hoffmann, R. A new, general route to functionally substituted η^5-cyclopentadienyl metal compounds. *J. Am. Chem. Soc.* **102**, 1196–1198 (1980).
6. Milner, P. J., Maimone, T. J., Su, M., Chen, J., Müller, P. & Buchwald, S. L. Investigating the dearomative rearrangement of biaryl phosphine-ligated Pd(II) complexes. *J. Am. Chem. Soc.* **134**, 19922–19934 (2012).
7. Neese, F. The ORCA program system. *Wiley Interdiscip. Rev. Comput. Mol. Sci.* **2**, 73–78 (2012).
8. Neese, F. Software update: the ORCA program system—version 6.0. *Wiley Interdiscip. Rev. Comput. Mol. Sci.* **15**, e70019 (2025).
9. Adamo, C. & Barone, V. Toward reliable density functional methods without adjustable parameters: the PBE0 model. *J. Chem. Phys.* **110**, 6158–6170 (1999).
10. Caldeweyher, E. *et al.* A generally applicable atomic-charge dependent London dispersion correction. *J. Chem. Phys.* **150**, 154122 (2019).
11. Franzke, Y. J., Treß, R., Pazdera, T. M. & Weigend, F. Error-consistent segmented contracted all-electron relativistic basis sets of double- and triple-zeta quality for NMR shielding constants. *Phys. Chem. Chem. Phys.* **21**, 16658–16664 (2019).
12. Peng, D., Middendorf, N., Weigend, F. & Reiher, M. An efficient implementation of two-component relativistic exact-decoupling methods for large molecules. *J. Chem. Phys.* **138**, 184105 (2013).
13. Riplinger, C., Sandhoefer, B., Hansen, A. & Neese, F. Natural triple excitations in local coupled cluster calculations with pair natural orbitals. *J. Chem. Phys.* **139**, 134101 (2013).
14. Lu, T. & Chen, F. Multiwfn: a multifunctional wavefunction analyzer. *J. Comput. Chem.* **33**, 580–592 (2012).
